# Tether-directed synthesis of highly substituted oxasilacycles *via* an intramolecular allylation employing allylsilanes

**DOI:** 10.1186/1860-5397-3-6

**Published:** 2007-02-08

**Authors:** Peter J Jervis, Liam R Cox

**Affiliations:** 1 School of Chemistry, The University of Birmingham, Edgbaston, Birmingham, B15 2TT, UK

## Abstract

**Background:**

Using a silyl tether to unite an aldehyde electrophile and allylsilane nucleophile into a single molecule allows a subsequent Lewis-acid-mediated allylation to proceed in an intramolecular sense and therefore receive all the benefits associated with such processes. However, with the ability to cleave the tether *post* allylation, a product that is the result of a net intermolecular reaction can be obtained. In the present study, four diastereoisomeric β-silyloxy-α-methyl aldehydes, which contain an allylsilane tethered through the β-carbinol centre, have been prepared, in order to probe how the relative configuration of the two stereogenic centres affects the efficiency and selectivity of the intramolecular allylation.

**Results:**

*Syn*-aldehydes, ***syn*-4a** and ***syn*-4b**, both react poorly, affording all four possible diastereoisomeric oxasilacycle products. In contrast, the *anti* aldehydes ***anti*-4a** and ***anti*-4b** react analogously to substrates that lack substitution at the α-site, affording only two of the four possible allylation products.

**Conclusion:**

The outcome of the reaction with *anti*-aldehydes is in accord with reaction proceeding through a chair-like transition state (T.S.). In these systems, the sense of 1,3-stereoinduction can be rationalised by the aldehyde electrophile adopting a pseudoaxial orientation, which will minimise dipole-dipole interactions in the T.S. The 1,4-stereoinduction in these substrates is modest and seems to be modulated by the R substituent in the starting material. In the case of the *syn*-substrates, cyclisation through a chair T.S. is unlikely as this would require the methyl substituent α to the reacting carbonyl group to adopt an unfavourable pseudoaxial position. It is therefore proposed that these substrates react through poorly-defined T.S.s and consequently exhibit essentially no stereoselectivity.

## Background

*Intra*molecular reactions offer distinct advantages over their *inter*molecular counterparts providing the tethering unit, which connects the reacting functionalities, is neither too long such that the reaction resembles an intermolecular process, nor too short, in which case geometrical constraints can physically prevent the reaction. When these conditions on the tether are satisfied, however, the proximity of the reacting partners, combined with a reduction in the degrees of freedom in the system, render the intramolecular reaction more entropically and kinetically favourable. This can result in a more stereo-, regio- and chemoselective process, which is often reflected in an increased yield of the desired product.

**Scheme 1:**
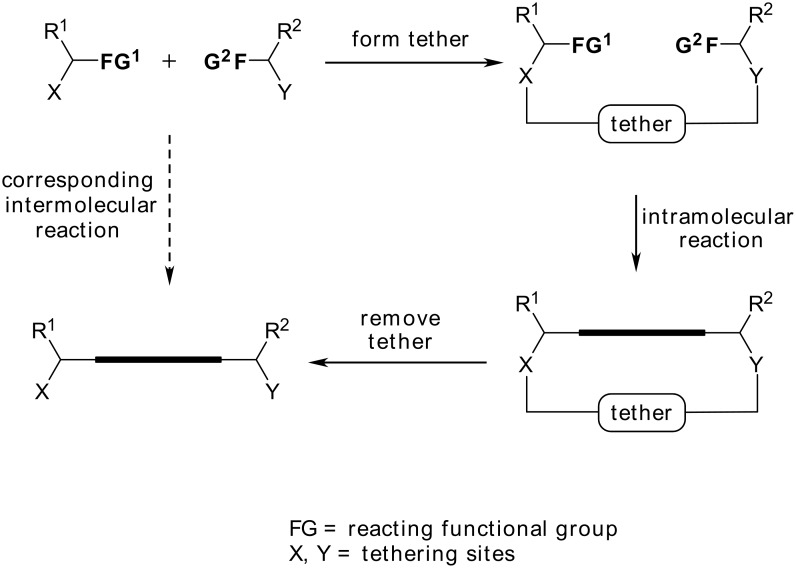
Synthesis using a Temporary Connection Strategy.

We have been investigating the use of a *temporary* tether to link two reacting partners. [1–3] By using such a transient linker, which can be cleaved *post* reaction, it is possible to accrue the benefits associated with an intramolecular process and yet still obtain a product that derives from a net intermolecular reaction (Scheme 1). [4] Silyl groups have proven to be particularly popular tethering units for this purpose. [4–6] They can be attached to carbon, oxygen and nitrogen functionalities in a variety of ways, and are often stable to a diverse array of reaction conditions. [4] Furthermore, the silyl tether can be manipulated *post* reaction in a range of ways. [7–8] The silyl reagents that are required to prepare the tether are also relatively cheap, exhibit low toxicity and are widely available.

**Scheme 2:**
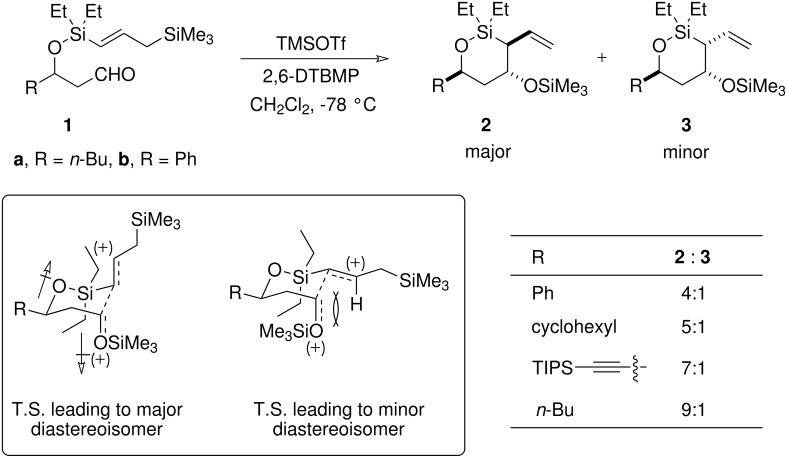
Intramolecular allylation of aldehyde **1** generates two out of the four possible oxasilacycles. The best 1,4-stereoinduction is achieved when less sterically demanding R groups are employed.

A number of groups have used the silyl group embedded in an allylsilane as the temporary connection for studying intramolecular allylation reactions. [9–14] We have taken a different approach, choosing to append an additional silyl group to the γ-position of the allylsilane nucleophile and use this as the tethering site instead (Scheme 2). This modification confers a number of advantages on the resulting system: first, it ensures that the allylsilane is exocyclic in the T.S. allowing a direct comparison with the analogous intermolecular reaction; second, the size of the cyclic T.S. is two atoms smaller – and should therefore be better defined – than if the silyl connection were contained within the allylsilane itself; third, the silyl tether remains intact *post* allylation, to provide a product that can be elaborated in a wide variety of ways.

We recently showed that this Temporary Silicon Connection strategy provides a useful method for the stereoselective allylation of aldehydes (Scheme 2). [3] In this study, Lewis acid-mediated allylation of aldehyde **1**, provided the oxasilacycle allylation products **2** and **3** in good yield. More significantly, owing to the complete 1,3-stereoinduction that is observed in this cyclisation, only these two – out of a possible four – oxasilacycles were obtained. We have rationalised the sense of 1,3-induction observed in this reaction on electrostatic grounds using a modification of Evans' dipole model, [15] in which the dipole moments across the polar C=O and C-O bonds oppose one another in a chair-like T.S. (Figure inset in Scheme 2). The levels of 1,4-stereoinduction in the reaction of aldehyde **1** are more modest. We have argued that the selectivity for the major product **2** arises from minimising steric interactions, principally those between the allylsilane and the ethyl substituents contained within the silyl tether (we have recently shown[16] that replacing the diethylsila-component for a methylene group reverses the sense of 1,4-stereoinduction). This is best achieved by placing the reacting allylsilane in a pseudoaxial orientation in a chair-like T.S. (Figure inset in Scheme 2).

It would be expected that large R groups in the cyclisation precursor **1**, such as phenyl and cyclohexyl substituents, would serve as the most effective conformational anchors for our proposed chair-like T.S. (A values:[17] cyclohexyl: 2.2 kcal mol^-1^; phenyl: 2.8 kcal mol^-1^). These groups should occupy a pseudoequatorial position in order to minimise 1,3-diaxial interactions across the ring. Interestingly, these substrates display some of the lowest levels of 1,4-stereoinduction (Entries 1,2, Table in Scheme 2); indeed, the highest levels of 1,4-induction are obtained when substrates containing *less* sterically demanding substituents, such as *n-*Bu and TIPS-C≡C-groups, are employed (Entries 3,4, Table in Scheme 2) (A values:[17] ethynyl = 0.41–0.52 kcal mol^-1^; ethyl = 1.79 kcal mol^-1^). We acknowledge that analysing steric interactions and predicting favoured conformations for such heavily substituted six-membered cyclic T.S.s is not straightforward, especially for substrates with substituents (*i.e*. small R groups) that are not strong conformational anchors. However, we postulate that when R is large (*e.g*. R = Ph), the reaction proceeds through a standard Zimmerman-Traxler chair T.S. For those substrates that possess small R substituents, however, the R group provides less of a conformational lock for a chair T.S. Consequently, this allows for small structural changes away from a chair conformation, which serve to alleviate the unfavourable interactions associated with placing the allylsilane in a pseudoaxial orientation and lead to the improved levels of 1,4-induction that are observed in these systems. The presence of relatively long C-Si and O-Si bonds and a relatively flexible O-Si-C bond angle, in the cyclic T.S. means that such deviations from the classical Zimmerman-Traxler T.S. are likely to be readily accommodated.

**Scheme 3:**
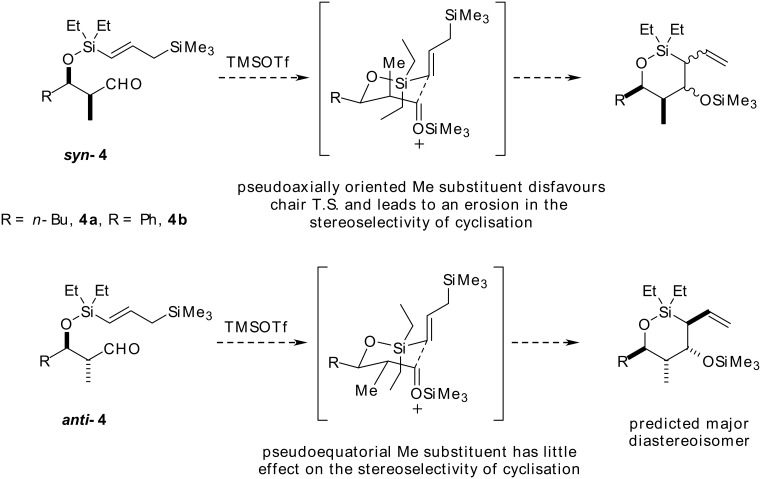
The effect of introducing a methyl group α- to the aldehyde in the cyclisation precursor will depend on the relative stereochemistry.

In light of the interesting substituent effect on 1,4-induction, we were keen to investigate how incorporating additional substituents into the substrate might influence the stereoselectivity of the reaction. Specifically we wanted to assess how incorporating an additional methyl group α to the aldehyde functionality would affect the stereoselectivity of the reaction. We hypothesised that if intramolecular allylation proceeds through a chair-like T.S., then the α-methyl group in *syn*-aldehyde ***syn*-4** will occupy a pseudoaxial position. Since this would lead to additional unfavourable 1,3-diaxial interactions, we postulated that cyclisation would likely proceed through alternative reactive conformations with a less predictable stereochemical outcome (Scheme 3). In contrast, a pseudoequatorially orientated methyl substituent, which would result from cyclisation of *anti*-aldehyde ***anti*-4**, might be expected to exert little impact on the stereoselectivity of the reaction (Scheme 3). To test our hypothesis, we chose to carry out these transformations on the *n-*Bu substrate, **4a**, as a representative of aldehydes possessing a substituent that imposes a relatively poor conformational lock, and compare the results with those for the Ph substrate, **4b**, which represents one of the more sterically demanding substituents.

## Results and discussion

**Scheme 4:**
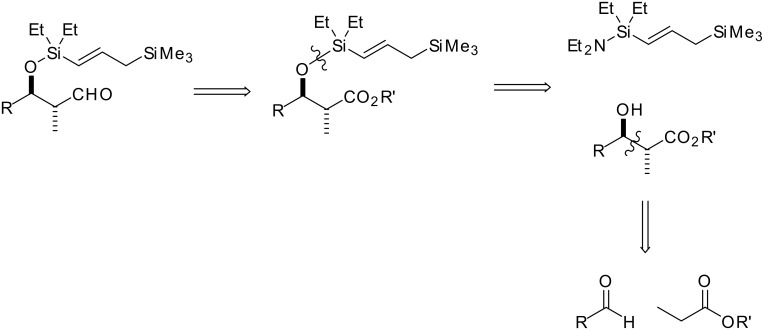
Retrosynthesis of aldehyde ***anti*-4**.

The desired cyclisation precursors **4a** and **4b** were prepared using our well-established method. [3] The retrosynthetic analysis for the *anti* series of products is outlined in Scheme 4.

**Scheme 5:**
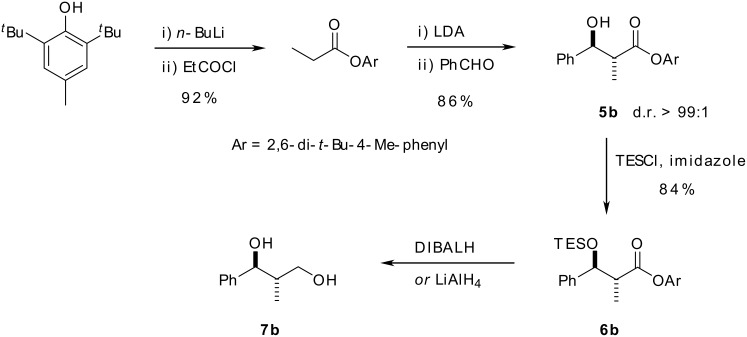
Attempts to reduce the bulky aryl ester resulted in Si-O bond cleavage and over-reduction to the primary alcohol.

We first required access to both *syn*- and *anti*-β-hydroxy ester diastereoisomers of our two test substrates. *Anti*-β-hydroxy ester, ***anti*-5b**, was prepared with complete diastereoselectivity (the minor diastereoisomer was not observed in the crude reaction mixture on analysis by 300 MHz ^1^H-NMR spectroscopy) by a method described by Heathcock *et al*. (Scheme 5). [18] We were concerned, however, that the steric bulk of the aryl ester in **5b**, which is required to impart the complete *anti* selectivity on the aldol reaction, would make unmasking of the aldehyde difficult owing to unfavourable steric clashes between the carbonyl group and one of the *tert*-butyl groups in the aryl ring forcing the aromatic group to rotate out of the plane of the ester, leaving the bulky *tert*-butyl groups to flank the faces of the carbonyl and block the Bürgi-Dunitz approach trajectory of the reducing agent. We therefore chose to investigate this reduction step on the model substrate **6b**, where the TES-ether would function as a cheaper mimic of the tethered allylsilane in our desired system. As expected, under the reaction conditions which had to be employed to effect reduction (LiAlH_4_ in Et_2_O or DIBALH in CH_2_Cl_2_ at reflux), it was neither possible to prevent Si-O bond cleavage, nor were we able to halt the reaction at the aldehyde stage, and consequently diol **7b** was the only product isolated (Scheme 5).

**Scheme 6:**
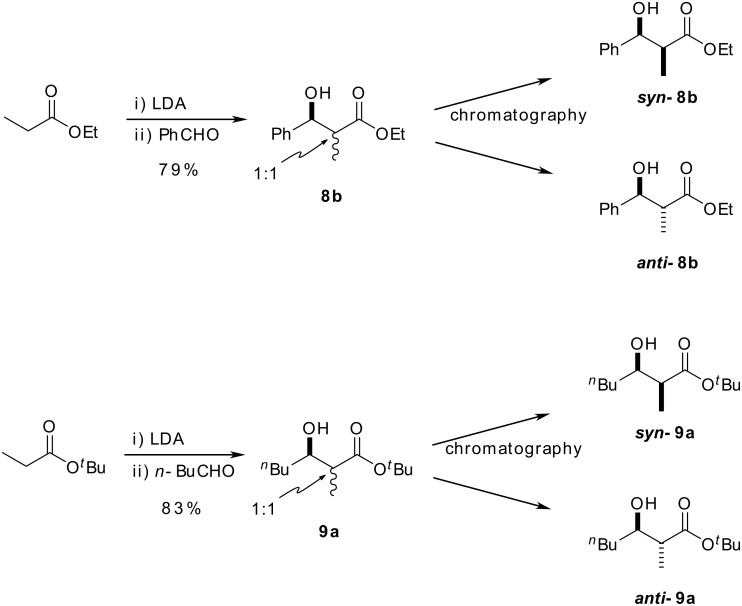
Preparation of *syn*- and *anti*-β-hydroxy esters.

We therefore switched our attention back to ethyl esters, which we knew from previous studies could be reduced directly to the required aldehyde with DIBALH at low temperature. [3] The reaction between the lithium enolate of ethyl propionate and benzaldehyde produced a 1:1 mixture of aldol products, ***syn*-8b** and ***anti*-8b**, in good yield (Scheme 6). [19] These were readily separated by flash column chromatography to afford the two required aldol diastereoisomers in gramme quantities. The same reaction employing valeraldehyde also led to the desired two diastereoisomeric products ***syn*-8a** and ***anti*-8a** in good yield (1:1 ratio); however this time, the two products proved to be inseparable by flash column chromatography. Fortunately, when *t*-butyl propionate was employed as the enolate precursor, we were able to access the readily separable *t*-butyl ester aldol products ***syn*-9a** and ***anti*-9a** in good yield (Scheme 6). The relative stereochemistry of these products was confirmed by comparison with literature ^1^H-NMR data. [20–21] The relative stereochemistry of ***anti*-8b** was further verified by comparing its diol reduction product with that obtained from the reduction of aryl ester ***anti-5*b** prepared earlier, which was of known *anti* configuration.

**Scheme 7:**
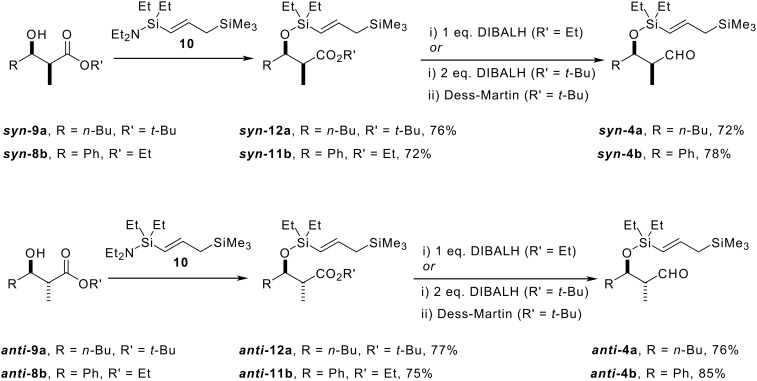
Preparation of the *syn-* and *anti*-aldehyde cyclisation precursors **4**.

γ-(Amino)silyl-substituted allylsilane **10** was synthesised according to our standard procedure, [3] and used to tether our allylsilane to the hydroxyl groups of both *syn-* and *anti*-β-hydroxy esters, **9a** and **8b**, by simply stirring the two reagents in the absence of solvent (Scheme 7). The by-product from this reaction is Et_2_NH, which can be easily removed by evaporation under reduced pressure at the end of the reaction. Subsequent DIBALH reduction of ethyl esters ***syn*-11b** and ***anti*-11b** produced the desired cyclisation precursors, aldehydes ***syn*-4b** and ***anti*-4b**, respectively. In the case of the two *t*-butyl esters ***syn*-12a**, and ***anti*-12a**, we were unable to effect direct reduction to the aldehyde in high yield owing to the propensity for the intermediate aldehyde to be reduced further to the corresponding primary alcohol. Presumably in the case of these *t*-butyl esters, increased steric compression in the initial tetrahedral intermediate causes this to collapse to the aldehyde, even at low temperature, allowing further reduction to the corresponding primary alcohols. Fortunately, the two alcohol products could be oxidised to the desired aldehydes ***syn*-4a** and ***anti*-4a**, using Dess-Martin periodinane[22–23] without epimerisation of the α-stereogenic centre (Scheme 7).

**Scheme 8:**
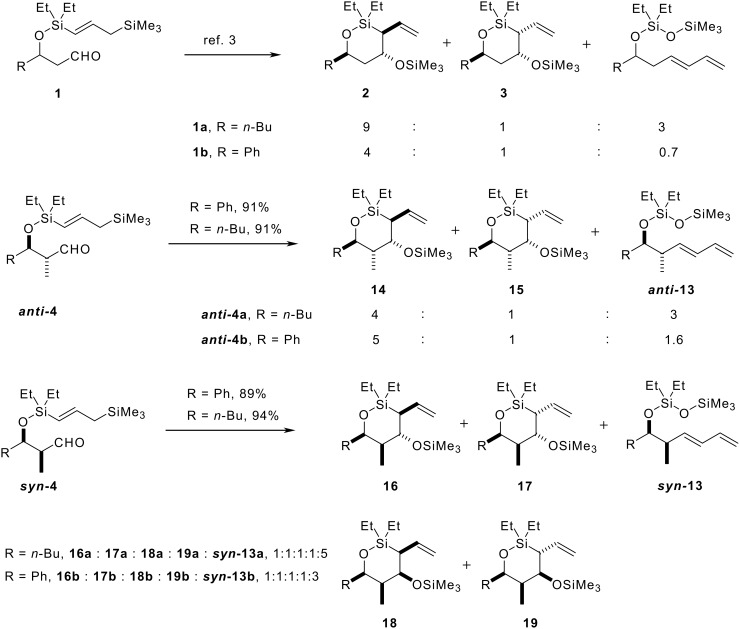
Intramolecular allylation results.

With all four cyclisation precursors in hand, we were ready to conduct our intramolecular allylation study. Each aldehyde substrate (>95:5 d.r. in all four cases) was treated with TMSOTf in the presence of 2,4,6-tri-*t*-butyl pyrimidine (TTBP), [24] which acts as a Brønsted acid scavenger, in CH_2_Cl_2_ as solvent, conditions that had proved successful in our previous cyclisation studies. [3] The results from these reactions are summarised in Scheme 8.

The first point to note is that the reactions of aldehydes ***syn*-4a** and ***syn*-4b** were poorly stereoselective; all four diastereoisomers were formed in both cases, as well as a significant amount of the corresponding side-product diene ***syn*-13a** and ***syn*-13b** (the diene may be formed in a variety of ways; we favour a mechanism involving a vinylogous silicon-mediated olefination as this best accounts for the excellent (*E*)-stereoselectivity observed). [25–27] The relative stereochemistry of each diastereoisomer in both cases was elucidated by extensive NMR experiments (see the Experimental Section in the Additional Files for full details). The two *syn*-aldehydes reacted not only with poor stereoselectivity, they also cyclised at a much slower reaction rate (24 h reaction time) than was observed with the corresponding α-unsubstituted aldehydes **1**. The results with both Ph and *n*-Bu substrates, ***syn*-4a** and ***syn*-4b**, respectively, are consistent with the *syn-*methyl group disfavouring chair-like T.S.s, owing to the fact that the additional methyl group would be forced to adopt a pseudoaxial orientation. Consequently we believe that cyclisation for these substrates proceeds through poorly defined T.S.s, resulting in the observed erosion in the stereoselectivity of the reaction.

**Figure 1:**
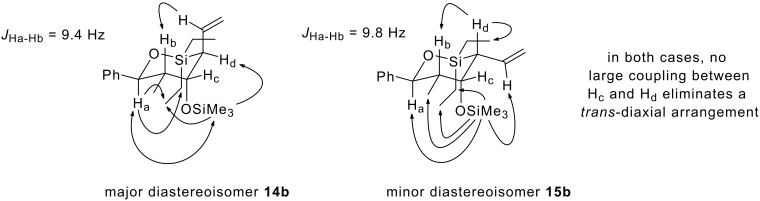
nOe data for the two oxasilacycles obtained from allylation of aldehyde ***anti*-4b**.

In marked contrast to the two *syn* aldehydes, cyclisation of *anti*-aldehydes, ***anti*-4a** and ***anti*-4b**, provided results which were more comparable with those obtained using the corresponding α-unsubstituted aldehydes **1a** and **1b**. The reaction times, 10 h for ***anti*-4a**, and 6 h for ***anti*-4b**, were much closer to those required for substrates lacking the α-Me substituent (8 h for both *n*-Bu and Ph substrates, **1a** and **1b**, respectively), and in line with our previous observations (Scheme 2), only two out of the possible four oxasilacycles were formed in both cases. Once again, extensive NMR experiments confirmed the relative stereochemistry in the two diastereoisomers and showed that complete 1,3-stereoinduction is obtained in both cyclisations. As expected, the sense of 1,3-induction was the same as was observed with the α-unsubstituted analogues **1a** and **1b** (Figure 1). The two allylation products in each case therefore arise from the modest level of 1,4-stereoinduction observed in both cyclisations.

Qualitatively, the observations with the two *anti*-aldehydes, ***anti*-4a** and ***anti*-4b**. are consistent with cyclisation proceeding through a chair-like T.S. in which the α-methyl group provides a further conformational lock by adopting a pseudoequatorial orientation. More careful analysis of the levels of 1,4-stereoinduction in these cyclisations, and comparison with the results obtained using the corresponding α-unsubstituted aldehydes, **1** (Scheme 2), reveals an erosion of stereoselectivity when cyclising the *n*-Bu substrate (9:1 for **1a** to 4:1 for ***anti*-4a**), whereas the stereoselectivity obtained when cyclising the phenyl substrate ***anti*-4b**, is essentially unchanged (4:1 for **1b**, 5:1 for ***anti*-4b**). We can interpret these results in two ways. One possibility is that the additional methyl group in ***anti*-4a** provides an additional conformational anchor for a chair-like T.S. The reactive conformation for ***anti*-4a** therefore deviates towards a more chair-like T.S., as is observed for substrates possessing bulkier substituents such as ***anti*-4b** and **1b**. This serves to bring down the stereoselectivity for ***anti*-4a** to similar levels to those observed for systems that react through more chair-like T.S.s. An alternative explanation is that ***anti*-4a** reacts through a similar T.S. to its α-unsubstituted analogue **1a**, which deviates from a chair-like conformation. The additional α-methyl group in ***anti*-4a** then introduces additional unfavourable interactions in this favoured T.S., which leads to the erosion in the level of 1,4-induction.

## Summary

We have previously shown that allylsilanes tethered through a γ-silyl substituent to a series of β-hydroxy aldehydes cyclise with complete 1,3-stereoinduction but afford two diastereoisomeric products owing to the more modest levels of 1,4-stereoinduction. In the present study we have incorporated an α-methyl substituent into the substrate to probe how this affects the stereoselectivity of the reaction. We have shown that the relative stereochemistry of the two stereogenic centres in the starting aldehyde **4** has a profound effect on the efficiency of the reaction. *Syn*-aldehydes react poorly, affording mixtures of all four possible oxasilacycles in addition to appreciable quantities of a diene side-product. The results with *anti*-aldehydes are more interesting. In line with our prediction, substrates possessing this relative stereochemistry provide results which are comparable to those from aldehydes that lack a substituent at the α-site. That a slight reduction in 1,4-stereoinduction is observed with the *n*-Bu substrate ***anti*-4b** supports the idea that substrates, which lack substituents that provide a strong conformational anchor on the reactive conformation, react through a T.S. that deviates from a classical Zimmerman-Traxler chair conformation. Studies are now focusing on how the geometry of the double bond in the tethered allylsilane also influences the stereoselectivity of this reaction.

## Supporting Information

File 1 Experimental details and characterisation data.

File 2 ^1^H-NMR and ^13^C-NMR Spectra for the following compounds: **5b, 6b, 7b,** ***syn*-8b,** ***anti*-8b,** ***syn*-11b,** ***anti*-11b,** ***syn*-4b,** ***anti*-4b,** ***syn*-9a,** ***anti*-9a,** ***syn*-12a,** ***anti*-12a,** ***syn*-4a,** ***anti*-4a,** ***syn*-13b,** ***anti*-13b,** ***syn*-13a,** ***anti*-13a**.

File 3 ^1^H-NMR and ^13^C-NMR Spectra for the following compounds: **16a**, **17a**.

File 4 ^1^H-NMR and ^13^C-NMR Spectra for the following compounds: **18a**, **19a**.

File 5 ^1^H-NMR and ^13^C-NMR Spectra for the following compounds: **14a**, **15a**.

File 6 ^1^H-NMR and ^13^C-NMR Spectra for the following compounds: **16b**, **17b**.

File 7 ^1^H-NMR and ^13^C-NMR Spectra for the following compounds: **18b**, **19b**.

File 8 ^1^H-NMR and ^13^C-NMR Spectra for the following compounds: **14b**, **15b**.
