# Supplementary material for: Tether-directed synthesis of highly substituted oxasilacycles via an intramolecular allylation employing allylsilanes
Source: Beilstein J Org Chem. 2007 Feb 8;3:6. doi: 10.1186/1860-5397-3-6 (PMC1810303; doi:10.1186/1860-5397-3-6)
Supplement: File 1 — Experimental details and characterisation data. [file Beilstein_J_Org_Chem-03-06-s001.pdf]

Additional File 1

Tether-directed synthesis of highly substituted oxasilacycles *via* an intramolecular allylation employing allylsilanes

Peter J. Jervis and Liam R. Cox*

email: l.r.cox@bham.ac.uk

Experimental Details and Characterisation Data

General Experimental

Infrared spectra were recorded either neat as thin films between sodium chloride discs, or as a Nujol mull between sodium chloride discs. The intensity of each band is described as s (strong), m (medium) or w (weak) and with the prefix v (very) and suffix br (broad) where appropriate. ^{1}H-NMR and ^{13}C-NMR spectra were recorded in $CDCl_3$ unless stated otherwise, at 500 and 125 MHz, 400 and 100 MHz, or 300 and 75 MHz, respectively. Chemical shifts are reported as δ values (ppm) referenced to the following solvent signals: $CHCl_3$, δ_H 7.26; $CDCl_3$, δ_C 77.0. The term, 'stack' is used to describe a region where resonances arising from non-equivalent nuclei are coincident, and multiplet, m, to describe a region where a resonance arises from a single nucleus (or equivalent nuclei) but coupling constants cannot be readily assigned. In analysing AB systems, where the resonance pattern forms two, well-separated groups, each of two lines, these are separately reported as "A of AB" and "B of AB", along with J_{AB}. In analysing ABX (and similar) systems, where the resonance pattern forms two, clearly separated groups of lines (two sets of four lines for an ABX system), these are reported as “A of ABX” and “B of ABX”, along with $J_{A\text{-}B}$, which can be directly measured from the spectra. Whilst $J_{A\text{-}X}$ cannot strictly be measured directly from the spectrum, the value obtained from the spectrum is sufficiently close to the actual value for it still to be useful; however it is acknowledged that the values quoted for $J_{A\text{-}X}$ and $J_{B\text{-}X}$ are not the true values. Connectivities were deduced from COSY90, HSQC and HMBC experiments. GOESY experiments were used to elucidate relative stereochemistry. Mass spectra were recorded on a LCT spectrometer utilising electrospray ionisation (and a methanol mobile phase), or electron impact ionisation, and are reported as (*m/z* (%)). HRMS were recorded on a LCT spectrometer using a lock mass incorporated

into the mobile phase. Melting points were determined using open capillaries and are uncorrected.

Preparative HPLC was performed on a reverse phase Phenomenex Luna 10u C18(2)100A 50 x 21, 20 mm x 10 micron fitted with a Dionex P580 pump and a Dionex UVD170S detector (used at 210 and 225 nm) using a helium-degassed HPLC grade water/acetonitrile gradient, without acidic additives. Elution was monitored and spectra were recorded on Dionex Chromeleon 6.11 software.

Reactions were monitored by thin layer chromatography using pre-coated glass-backed silica plates (60A F_{254}) and visualised by UV detection (at 254 nm) and with ammonium molybdate(IV) - cerium(IV) sulfate staining dip or potassium manganate(VII) staining dip or 2,4-dinitrophenylhydrazine staining dip (for aldehydes). Column chromatography was performed on silica gel (particle size 40-63 μm mesh).

All reactions were conducted in oven-dried (140 °C) or flame-dried glassware under a nitrogen atmosphere, and at ambient temperature (20 to 25 °C) unless otherwise stated, with magnetic stirring. Volumes of 1 mL or less were measured and dispensed with gastight syringes. Evaporation and concentration under reduced pressure was performed at 50 - 500 mbar. Residual solvent was removed under high vacuum (1 mbar).

All reagents were obtained from commercial sources and used without further purification unless stated otherwise. Trimethylsilyl trifluoromethanesulfonate (triflate) was stored in a Schlenk tube under nitrogen at 4 °C and used whilstever the

compound was colourless. Over time, a pink colouration was observed whereupon fresh TMSOTf was used. Dichloromethane and cyclohexane were freshly distilled under nitrogen from CaH_2. Diethyl ether was freshly distilled under nitrogen from sodium benzophenone ketyl. Toluene was freshly distilled from sodium. $^{i}Pr_2NH$ was distilled under a nitrogen atmosphere from KOH and stored under nitrogen at room temperature over activated 4 Å molecular sieves. All solutions are aqueous and saturated unless stated otherwise. TMSCl was distilled under nitrogen from CaH_2. 2,4,6-Tri-*tert*-butylpyrimidine (TTBP) was synthesised following a known procedure.[1] Dess Martin Periodinane was synthesised according to a known procedure.[2,3]

2'-6'-di-*t*-butyl-4'-methylphenyl propionate (20)

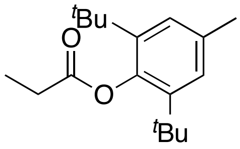

^{n}BuLi (4.72 mL, 11.85 mmol, 2.5 M in hexane) was added dropwise over 5 min to a solution of 2,6-di-*t*-butyl-4-methylphenol (2.60 g, 11.85 mmol) in THF (12 mL) at 0 ^{o}C. The reaction mixture was stirred at this temperature for 15 min before propionyl chloride (1.54 mL, 17.7 mmol) was added dropwise over 10 min. The solution was allowed to warm to rt and then stirred overnight. NH_4Cl solution (24 mL) and EtOAc (12 mL) were added and the layers were separated. The aqueous phase was extracted with EtOAc (2 x 24 mL) and the combined organic extracts were washed with brine (24 mL), dried ($MgSO_4$) and concentrated under reduced pressure. Reduced pressure distillation (0.5 mmHg) afforded **20** as a colourless viscous oil (3.02 g, 92%); b.p. 122 ^{o}C at 0.5 mmHg (lit.[4] 120 ^{o}C at 0.5 mmHg); ν_{max}(film)/cm^{-1} 3583w, 3075w, 2963s, 2874s, 1760s (C=O), 1599m, 1483m, 1462m 1422s,1395m, 1393m, 1364s, 1348m, 1269m, 1219m, 1199s, 1188s, 1148s, 1110s, 1075m, 1005w, 983m, 949w, 927w, 889m, 860m, 805w, 752w, 666w; δ_H (300 MHz) 1.27 (3H, t, *J* 7.7, C*H*$_3$CH$_2$C=O), 1.31 (18H, s, 2 x C(C*H*$_3$)$_3$), 2.31 (3H, s, ArC*H*$_3$), 2.64 (2H, q, *J* 7.7, CH$_3$C*H*$_2$C=O), 7.10 (2H, s, Ar*H*); δ_C (75 MHz) 8.6 (CH$_3$, *C*H$_3$CH$_2$C=O), 21.4 (CH$_3$, Ar*C*H$_3$), 29.0 (quat. C, *C*(CH$_3$)$_3$), 31.4 (CH$_3$, C(*C*H$_3$)$_3$), 35.2 (CH$_2$, CH$_3$*C*H$_2$C=O), 126.9 (CH, 2 x Ar *C*-H), 134.3 (quat. C, *ipso*Ph), 141.8 (quat. C, *ipso*Ph), 145.9 (quat. C, *ipso*Ph), 174.1 (quat. C, *C*=O); *m/z* (TOF ES+) 299.5 ([M+Na]$^+$, 100%); HRMS *m/z* (TOF ES+) 299.1985. $C_{18}H_{28}NaO_2$ requires 299.1987. Data were in agreement with those reported in the literature.[4]

(2*R, 3*S**)-2'-6'-di-*t*-butyl-4'-methylphenyl 2-methyl-3-hydroxy-3-phenyl-propanoate (5b)**

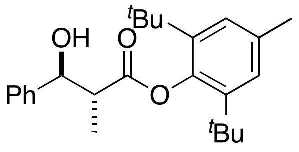

^{n}BuLi (7.33 mL, 18.33 mmol, 2.5 M in hexane) was added dropwise over 5 min to a stirred solution of di*iso*propylamine (2.55 mL, 18.33 mmol) in THF (115 mL) at 0 ^{o}C and the reaction mixture was stirred at this temperature for 20 min. The reaction mixture was then cooled to –78 ^{o}C before a solution of ester **20** (5.06 g, 18.33 mmol) in THF (5 mL) was added dropwise over 5 min. After 1 h at –78 ^{o}C, benzaldehyde (1.85 mL, 18.33 mmol) was added. After stirring for 15 min, NH_4Cl solution (100 mL) was added and the solution was warmed to rt. The layers were separated and the aqueous phase was extracted with EtOAc (3 x 100 mL). The combined organic extracts were washed with brine (50 mL), dried ($MgSO_4$) and concentrated under reduced pressure. Flash column chromatography (10% Et_2O in hexane) afforded β-hydroxy ester **5b** as a colourless, highly viscous oil (6.04 g, 86%); R_f: 0.23 (10% Et_2O in hexane); ν_{max}(film)/cm^{-1} 3472br m (OH), 3064w, 2962s, 1748m (C=O), 1599w, 1483w, 1455m, 1420m, 1395m, 1364m, 1301w, 1268m, 1199m, 1182s, 1107s, 1049m, 1022m, 914w, 889w, 859w, 799w, 763w, 748w, 702m, 666m; δ_H (300 MHz) 1.25 (3H, d, *J* 7.4, (C*H*$_3$)CHC=O), 1.32 (9H, s, 1 x C(C*H*$_3$)$_3$), 1.33 (9H, s, 1 x C(C*H*$_3$)$_3$), 2.33 (3H, s, ArCH$_3$), 3.07 (1H, app. quintet, *J* 7.4, (CH$_3$)C*H*C=O), 4.16 (1H, s, OH), 4.87 (1H, d, *J* 8.8, C*H*OH), 7.12-7.15 (2H, stack, Ar*H*), 7.31-7.42 (5H, stack, Ph*H*); δ_C (75 MHz) 13.7 (CH$_3$, (*C*H$_3$)CHC=O), 21.4 (CH$_3$, Ar*C*H$_3$), 31.3 (CH$_3$, 1 x C(*C*H$_3$)$_3$), 31.4 (CH$_3$, 1 x C(*C*H$_3$)$_3$), 35.0 (quat. C, 1 x *C*(CH$_3$)$_3$), 35.2 (quat. C, 1 x *C*(CH$_3$)$_3$), 47.6 (CH, (CH$_3$)*C*HC=O), 75.8 (CH, *C*HOH), 126.9 (CH, Ar), 127.0 (CH,

Ar), 127.2 (CH, Ar), 127.9 (CH, Ar), 128.3 (CH, Ar), 134.6 (quat. C, Ar), 140.9 (quat. C, Ar), 141.8 (quat. C, Ar), 145.7 (quat. C, Ar), 176.1 (quat. C, C=O); *m/z* (TOF ES+) 405.7 ($[M+Na]^+$, 100%); HRMS *m/z* (TOF ES+) 405.2420. $C_{25}H_{34}NaO_3$ requires 405.2406.

Data were in agreement with those reported in the literature.[4]

(2*R, 3*S**)-2'-6'-di-*t*-butyl-4'-methylphenyl 2-methyl-3-triethylsilanyloxy-3-phenyl-propanoate (6b)**

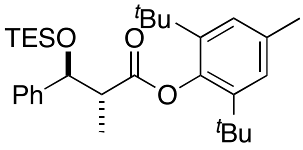

TESCl (0.60 mL, 3.42 mmol) was added over 15 min to a stirred solution of alcohol **5b** (1.23 g, 3.42 mmol) and imidazole (0.47 g, 6.84 mmol) in DMF (7 mL). The reaction mixture was stirred overnight and then poured into H_2O (30 mL) and extracted with hexane (3 x 30 mL). The combined organic extracts were washed with brine (30 mL), dried ($MgSO_4$), and concentrated under reduced pressure. Purification of the residue by flash column chromatography (1% Et_2O in hexane) afforded silylether **6b** as a colourless liquid (1.42 g, 84%); R_f = 0.2 (1% Et_2O in hexane); ν_{max}(film)/cm^{-1} 3501vw, 3075w, 2963s, 2916s, 2874s, 2290vw, 1760s (C=O), 1599m, 1483m, 1462m, 1422s, 1395m, 1382w, 1364s, 1348m, 1269m, 1219m, 1199s, 1188s, 1148s, 1110s, 1075m, 1005w, 983w, 949w, 927w, 889m, 860m, 805w, 752w; δ_H (300 MHz) 0.40-0.51 (6H, m, Si(C*H*$_2$CH$_3$)$_3$), 0.83 (9H, t, *J* 7.1, Si(CH$_2$C*H*$_3$)$_3$), 1.13 (9H, s, 1 x C(C*H*$_3$)$_3$), 1.22 (3H, d, *J* 7.3, (C*H*$_3$)CHC=O), 1.36 (9H, s, 1 x C(C*H*$_3$)$_3$), 2.29 (3H, s, ArC*H*$_3$), 3.11 (1H, app. quintet, *J* 7.1, (CH$_3$)C*H*C=O), 5.24 (1H, d, *J* 6.2, C*H*OSiEt$_3$), 7.06 (1H, s, Ar*H*), 7.11 (1H, s, Ar*H*), 7.17-7.41 (5H,

stack, Ph*H*); δ_C (75 MHz) 4.8 (CH_2, Si(CH_2CH_3)$_3$), 6.8 (CH_3, Si(CH_2CH_3)$_3$), 12.2 (CH_3, (CH_3)CHC=O), 21.4 (CH_3, ArCH_3), 31.2 (CH_3, C(CH_3)$_3$), 31.7 (CH_3, C(CH_3)$_3$), 35.1 (quat. C, *C*(CH_3)$_3$), 35.2 (quat. C, *C*(CH_3)$_3$), 49.0 (CH, (CH_3)*C*HC=O), 74.3 (CH, *C*HOSiEt$_3$), 126.8 (CH, Ar), 127.2 (CH, Ar) , 127.4 (CH, Ar), 127.6 (CH, Ar), 127.9 (CH, Ar), 134.3 (quat. C, Ar), 141.8 (quat. C, Ar), 142.1 (quat. C, Ar), 146.0 (quat. C, Ar), 173.4 (quat. C, C=O); *m/z* (TOF ES+) 519.5 ([M+Na]$^+$, 100%); HRMS *m/z* (TOF ES+) 519.3284. $C_{35}H_{34}NaO_3Si$ requires 519.3270.

(2*R**, 3*R**)-2-Methyl-1-phenyl-propane-1,3-diol (7b)

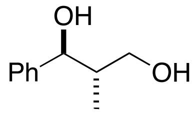

$LiAlH_4$ (162 mg, 4.30 mmol) was added portionwise to a stirred solution of ester **6b** (722 mg, 1.95 mmol) in THF (10 mL) at rt. The reaction mixture was then heated to reflux and stirred overnight. After cooling to rt, the reaction was quenched by the slow addition of EtOAc (10 mL). Aqueous HCl (20 mL of a 1 M solution) was added and the reaction mixture was stirred for 30 min. The layers were separated and the aqueous phase was extracted with EtOAc (2 x 20 mL). The combined organic extracts were washed with $NaHCO_3$ solution (20 mL) and brine (20 mL), dried ($MgSO_4$) and concentrated under reduced pressure to afford diol **7b** as a colourless oil (262 mg, 78%); ν_{max}(film)/cm^{-1} 4059w, 3358br s (OH), 3088s, 3064s, 3030s, 2931s, 2874s, 1953w, 1876w, 1811w, 1634m, 1607m, 1496s, 1454s, 1370m, 1209s, 1080m, 1016s, 913w, 816w, 735s, 670s, 666m; δ_H (300 MHz) 0.68 (3H, d, *J* 7.0, CHC*H*$_3$), 1.91-2.11 (1H, m, C*H*CH_3), 3.17 (1H, s, O*H*), 3.26 (1H, s, O*H*), 3.49-3.73 (2H, stack, C*H*$_2$OH), 4.52 (1H, d, *J* 8.4, C*H*Ph), 7.19-7.39 (5H, stack, Ph*H*); δ_C (75

MHz) 13.6 (CH_3, CH*C*H_3), 41.3 (CH, *C*HCH_3), 67.5 (CH_2, *C*H_2OH), 80.3 (CH, *C*HPh), 126.6 (CH, Ph), 127.4 (CH, Ph), 128.1 (CH, Ph), 143.3 (quat. C, *ipso*Ph); *m/z* (EI) 166 ($[M]^+$, 2%), 148 (14), 117 (3), 107 (100), 91 (7), 84 (40), 79 (42), 57 (2), 51 (7), 47 (9), 42 (13); HRMS *m/z* (EI) 166.0989. $C_{10}H_{14}O_2$ requires 166.0994.

(2*R**, 3*R**)-ethyl 3-hydroxy-2-methyl-3-phenylpropanoate (*syn*-8b) and (2*R**, 3*S**)-ethyl 3-hydroxy-2-methyl-3-phenylpropanoate (*anti*-8b)

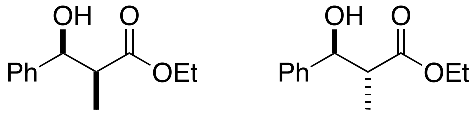

***syn*-8b** ***anti*-8b**

^{n}BuLi (7.33 mL, 18.33 mmol, 2.5 M in hexane) was added dropwise over 10 min to a stirred solution of di*iso*propylamine (2.55 mL, 18.33 mmol) in THF (115 mL) at 0 ^oC and the reaction mixture was stirred at this temperature for 20 min. The reaction mixture was then cooled to –78 ^oC before a solution of ethyl propionate (2.09 mL, 18.33 mmol) in THF (5 mL) was added dropwise over 5 min. After 1 h at –78 ^oC, benzaldehyde (1.85 mL, 18.33 mmol) was added. After stirring for 15 min, NH_4Cl solution (100 mL) was added and the solution was warmed to room temperature. The layers were separated and the aqueous phase was extracted with EtOAc (3 x 100 mL). The combined organic extracts were washed with brine (50 mL), dried ($MgSO_4$) and concentrated under reduced pressure to afford a 50:50 mixture of the two diastereoisomers which were separated by flash column chromotography (10% Et_2O in hexane) to afford the pure diastereoisomeric aldol products, both as colourless oils (3.02 g combined, 79%).

(2*R,3*R**)-ethyl 3-hydroxy-2-methyl-3-phenylpropanoate (*syn*-8b)**

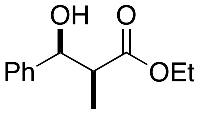

***syn*-8b**

R_f = 0.11 (10% EtOAc in hexane); ν_{max}(film)/cm^{-1} 3468s br (OH), 3087w, 3063w, 3031m, 2981s, 2939m, 2905m, 2883m, 2360w, 1955w, 1889w, 1732s (C=O), 1653w, 1636w, 1603w, 1586w, 1558w, 1559w, 1540w, 1522w, 1506w, 1494m, 1455s, 1394m, 1376s, 1336m, 1306s, 1249s, 1182s, 1120m, 1091m, 1073m, 1051s, 1026s, 930w, 916w, 869m, 767s, 703s; δ_H (300 MHz) 1.12 (3H, d, *J* 7.2, (CH_3)CHC=O), 1.21 (3H, t, *J* 7.0, OCH_2CH_3), 2.76 (1H, qd, *J* 7.0, 4.0, (CH_3)C*H*C=O), 2.97 (1H, s, OH) 4.12 (2H, q, *J* 7.0, OCH_2CH_3), 5.09 (1H, d, *J* 4.0, C*H*OH), 7.22-7.35 (5H, stack, Ph*H*); δ_C (75 MHz) 10.9 (CH_3, (CH_3)CHC=O), 13.9 (CH_3, OCH_2CH_3), 46.5 (CH, (CH_3)*C*HC=O), 60.5 (CH_2, OCH_2CH_3), 73.7 (CH, *C*HOH), 125.9 (CH, Ph), 127.3 (CH, Ph), 128.0 (CH, Ph), 141.6 (quat. C, *ipso*Ph), 175.5 (quat. C, *C*=O); *m/z* (TOF ES+) 231.1 ([M+Na]$^+$, 100%); HRMS *m/z* (TOF ES+) 231.1007. $C_{12}H_{16}NaO_3$ requires 231.0997.

(2*R**,3*S**)-ethyl 3-hydroxy-2-methyl-3-phenylpropanoate (*anti*-8b)

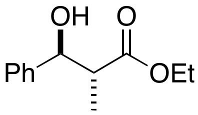

***anti*-8b**

R_f = 0.09 (10% EtOAc in hexane); ν_{max}(film)/cm^{-1} 3480s br (OH), 3088w, 3064m, 3031m, 2982s, 2939m, 2906m, 2881m, 2360w, 2340w, 1954w, 1889w, 1730s (C=O), 1653w, 1636w, 1604w, 1558w, 1540w, 1522w, 1494m, 1455s, 1375s, 1339s,1297m, 1252s, 1188s, 1125m, 1095m, 1060s, 1035s, 988m, 916w, 862w, 924w, 770s, 702s; δ_H (300 MHz) 1.02 (3H, d, *J* 7.0, (C*H*$_3$)CHC=O), 1.25 (3H, t, *J* 7.1, OCH$_2$C*H*$_3$), 2.66-2.74 (1H, m, (CH$_3$)C*H*C=O), 4.18 (2H, q, *J* 7.1, OC*H*$_2$CH$_3$), 4.75 (1H, d, *J* 8.4, C*H*OH), 7.25-7.36 (5H, stack, Ph*H*); δ_C (75 MHz) 13.9 (CH$_3$), 14.3 (CH$_3$), 47.1 (CH$_3$, (*C*H$_3$)CHC=O), 60.6 (CH$_2$, O*C*H$_2$CH$_3$), 76.1 (CH, *C*HOH), 126.5 (CH, Ph), 127.8 (CH, Ph), 128.2 (CH, Ph), 141.6 (quat. C, *ipso*Ph), 175.7 (quat. C, C=O); *m/z* (TOF ES+) 231.0 ([M+Na]$^+$, 100%); HRMS *m/z* (TOF ES+) 231.0994. $C_{12}H_{16}NaO_3$ requires 231.0997.

(*E*, 2*R,3*R**)-ethyl 3-[diethyl-(3'-trimethylsilanyl-prop-1'-enyl)-silanyloxy]-2-methyl-3-phenyl-propanoate (*syn*-11b)**

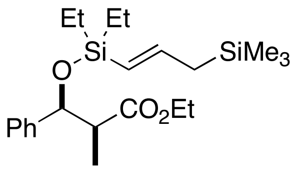

Alcohol ***syn*-8b** (800 mg, 3.85 mmol) and aminosilane **10** (1.05 g, 3.85 mmol) were stirred at 40 °C for 2 d. Evaporation of Et_2NH and purification of the residue by flash column chromotography (2% ether in hexane) afforded silyl ether ***syn*-11b** as a colourless oil (1.13 g, 72%); R_f = 0.21 (2% Et_2O in hexane); (Found: C, 65.20; H, 9.65. $C_{22}H_{38}O_3Si_2$ requires C, 64.97; H, 9.42); ν_{max}(film)/cm^{-1} 3064w, 3032w, 2955s, 2912m, 2877m, 2368w, 2340w, 2256w, 1733s (C=O), 1684w, 1653w, 1602m (C=C), 1559w, 1540w, 1506w, 1494w, 1456m, 1413w, 1372m, 1336w, 1312w, 1249s, 1179m, 1138m, 1066s, 1017m, 960w, 912w, 859s, 842s, 762m, 733m, 700s, 668w; δ_H (300 MHz) -0.01 (9H, s, Si(CH_3)$_3$), 0.41-0.59 (4H, stack, including [0.52 (2H, q, *J* 7.4, SiCH_2CH_3)], Si(CH_2CH_3)$_2$), 0.80 (3H, t, *J* 7.9, OSiCH_2CH_3), 0.88 (3H, t, *J* 7.9, OSiCH_2CH_3), 1.10 (3H, t, *J* 7.1, OCH_2CH_3), 1.19 (3H, d, *J* 6.9, (CH_3)CHC=O), 1.62 (2H, d, *J* 7.7, CH=CHCH_2), 2.68 (1H, app. quintet, *J* 7.0, (CH_3)C*H*C=O), 3.90-4.02 (2H, stack, OCH_2CH_3), 4.96 (1H, d, *J* 6.6, C*H*OSi), 5.25 (1H, d, *J* 18.4, C*H*=CHCH_2), 6.09 (1H, dt, *J* 18.4, 7.7, CH=C*H*CH_2), 7.19-7.30 (5H, stack, Ph*H*); δ_C (75 MHz) –2.0 (CH_3, Si(CH_3)$_3$), 5.42 (CH_2, SiCH_2CH_3), 5.43 (CH_2, SiCH_2CH_3), 6.6 (CH_3, SiCH_2CH_3), 6.7 (CH_3, SiCH_2CH_3), 12.4 (CH_3), 14.0 (CH_3), 28.8 (CH_2, CH=CHCH_2), 49.2 (CH, (CH_3)*C*HC=O), 60.0 (CH_2, OCH_2CH_3), 76.1 (CH, *C*HOSi), 122.6 (CH, *C*H=CH), 126.5 (CH, Ph), 127.2 (CH, Ph), 127.8 (CH, Ph), 143.3 (quat. C, *ipso*Ph), 147.2

(CH, *C*H=CH), 174.2 (quat. C, *C*=O); *m/z* (TOF ES+) 429.1 ($[M+Na]^+$, 100%); HRMS *m/z* (TOF ES+) 429.2264. $C_{22}H_{38}NaO_3Si_2$ requires 429.2257.

(*E,* 2*R,3*S**)-ethyl 3-[diethyl-(3'-trimethylsilanyl-prop-1'-enyl)-silanyloxy]-2-methyl-3-phenyl-propanoate (*anti*-11b)**

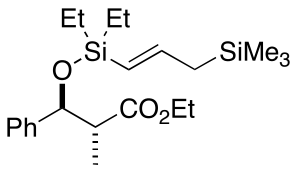

Alcohol ***anti*-8b** (800 mg, 3.85 mmol) and aminosilane **10** (1.05 g, 3.85 mmol) were stirred at 40 °C for 2 d. Evaporation of Et_2NH and purification of the residue by flash column chromotography (2 % ether in hexane) afforded silylether ***anti*-11b** as a colourless oil (1.13 g, 75 %); R_f = 0.20 (2% Et_2O in hexane); (Found: C, 64.71; H, 9.40. $C_{22}H_{38}O_3Si_2$ requires C, 64.97; H, 9.42%); ν_{max}(film)/cm^{-1} 3065w, 3031w, 2955s, 2912m, 2877m, 2360w, 2341w, 2555w, 1735s (C=O), 1684w, 1653w, 1602m (C=C), 1558w,1540w, 1506w, 1495w, 1456m, 1394w, 1375m, 1321w, 1249m, 1178, 1141m, 1069m, 1050m, 1016w, 960w, 912w, 859s, 842m, 826m, 764m, 734s, 701s; δ_H (300 MHz) –0.02 (9H, s, $Si(CH_3)_3$), 0.28-0.52 (4H, stack including [0.48 (2H, q, *J* 7.9, $SiCH_2CH_3$], $Si(CH_2CH_3)_2$), 0.73 (3H, t, *J* 7.9, $SiCH_2CH_3$), 0.77-0.85 (6H, stack, $SiCH_2CH_3$, (CH_3)CHC=O), 1.29 (3H, t, *J* 7.0, OCH_2CH_3), 1.60 (2H, d, *J* 7.7, CH=CHCH_2), 2.69-2.79 (1H, m, (CH_3)C*H*C=O), 4.10-4.22 (2H, stack, OCH_2CH_3), 4.76 (1H, d, *J* 9.2, C*H*OSi), 5.20 (1H, d, *J* 18.8, C*H*=CHCH_2), 6.04 (1H, dt, *J* 18.8, 7.7, CH=C*H*CH_2), 7.25-7.29 (5H, stack, Ph*H*); δ_C (75 MHz) –2.0 (CH_3, $Si(CH_3)_3$), 5.4 (CH_2, $Si(CH_2CH_3)_2$), 6.5 (CH_3, $Si(CH_2CH_3)_2$), 13.7 (CH_3), 14.2 (CH_3), 28.7 (CH_2, CH=CHCH_2), 49.1 (CH, (CH_3)*C*HC=O), 60.2 (CH_2, OCH_2CH_3), 77.4 (CH, *C*HOSi),

122.7 (CH, *C*H=CH), 127.1 (CH, Ph) 127.6, (CH, Ph), 130.0 (CH, Ph), 142.3 (quat. C, *ipso*Ph), 147.0 (CH, *C*H=CH), 175.2 (quat. C, *C*=O); *m/z* (TOF ES+) 429.2 ($[M+Na]^+$, 100%); HRMS *m/z* (TOF ES+) 429.2252. $C_{22}H_{38}NaO_3Si_2$ requires 429.2257.

(*E*, 2*R,3*R**)-3-[Diethyl-(3'-trimethylsilanyl-prop-1'-enyl)-silanyloxy]-2-methyl-3-phenyl-propanal (*syn*-4b)**

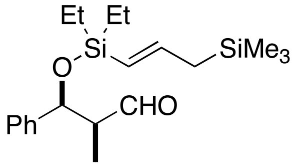

DIBALH (1.45 mL, 1.5 M in toluene, 2.18 mmol) was added dropwise over 30 min to a solution of ester ***syn*-11b** (0.72 g, 1.98 mmol) in CH_2Cl_2 (14 mL) at –78 °C. After 2 h, the reaction was quenched by the sequential addition of MeOH (87 μL, 2.18 mmol) and H_2O (235 μL, 13.08 mmol) at –78 °C. The resulting slurry was allowed to warm to rt and then filtered through $MgSO_4$ and Celite. The solvent was evaporated under reduced pressure to leave a yellow liquid which was purifed by flash column chromatography (2% Et_2O in hexane) to afford aldehyde ***syn*-4b** as a colourless liquid (0.56 g, 78%); R_f = 0.21 (2% Et_2O in hexane); ν_{max}(film)/cm^{-1} 3088w, 3064w, 2955s, 2913s, 2877s, 2712w, 2360w, 1948w, 1726s (C=O), 1684w, 1653w, 1602s, 1558w, 1540w, 1522w, 1507w, 1494m, 1455m, 1413m, 1363m, 1308w, 1249s, 1200m, 1142s, 1106s, 1089s, 1070s, 1035s, 959w, 943w, 912w, 859s, 764m, 749m, 725m, 701s; δ_H (300 MHz) 0.03 (9H, s, Si(CH_3)$_3$), 0.43-0.61 (4H, stack, including [0.57 (2H, q, *J* 7.7, SiCH_2CH$_3$)], Si(CH_2CH$_3$)$_2$), 0.80 (3H, t, *J* 7.7, OSiCH$_2$CH_3), 0.87 (3H, t, *J* 7.1, SiCH$_2$CH_3), 1.00 (3H, d, *J* 7.7, (CH_3)CHC=O), 1.62 (2H, d, *J* 7.7,

$CH{=}CHCH_2$), 2.54-2.64 (1H, m, (CH_3)C*H*C=O), 5.15 (1H, d, *J* 4.4, C*H*OSi), 5.25 (1H, d, *J* 18.7, C*H*=CHCH$_2$), 6.10 (1H, dt, *J* 18.7, 7.7, CH=C*H*CH$_2$), 7.21-7.30 (5H, stack, Ph*H*), 9.75 (1H, d, *J* 1.1, C*H*O); δ_C (75 MHz) -2.0, (CH_3, Si(CH_3)$_3$), 5.38 (CH_2, Si*C*H$_2$CH$_3$), 5.40 (CH_2, Si*C*H$_2$CH$_3$), 6.61 (CH_3, SiCH$_2$*C*H$_3$), 6.64 (CH_3, SiCH$_2$*C*H$_3$), 8.1 (CH_3, (*C*H$_3$)CHC=O) 28.8 (CH_2, CH=CH*C*H$_2$), 54.7 (CH, *C*HCH$_3$), 74.2 (CH, *C*HOSi), 122.2 (CH, *C*H=CH) 126.2 (CH, Ph), 127.3 (CH, Ph), 128.0 (CH, Ph), 142.4 (quat. C, *ipso*Ph), 147.8 (CH, C*H*=CH), 204.8 (CH, *C*=O); *m/z* (TOF ES+) 385.1 ([M+Na]$^+$, 100%); HRMS *m/z* (TOF ES+) 385.1982. $C_{20}H_{34}NaO_2Si_2$ requires 385.1995.

(*E*, 2*R,3*S**)-3-[Diethyl-(3-trimethylsilanyl-prop-1'-enyl)-silanyloxy]-2-methyl-3-phenyl-propanal (*anti*-4b)**

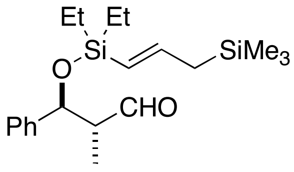

DIBALH (0.73 mL, 1.5 M in toluene, 1.09 mmol) was added dropwise over 30 min to a solution of ester ***anti*-11b** (0.36 g, 0.99 mmol) in CH_2Cl_2 (7 mL) at –78 ^oC. After 2 h, the reaction was quenched by the sequential addition of MeOH (44 μL, 1.09 mmol) and H_2O (120 μL, 6.90 mmol) at –78 ^oC. The resulting slurry was allowed to warm to rt and then filtered through $MgSO_4$ and Celite. The solvent was evaporated under reduced pressure to leave a yellow liquid which was purified by flash column chromatography (2% Et_2O in hexane) to afford aldehyde ***anti*-4b** as a colourless liquid (0.31 g, 85%); R_f = 0.20 (2% Et_2O in hexane); ν_{max}(film)/cm^{-1} 3065w, 3030w,

2955s, 2913s, 2877s, 2708w, 1944w, 1729s (C=O), 1684w, 1653w, 1636w, 1602s (C=C), 1559w, 1540w, 1522w, 1506w, 1493w, 1456m, 1413m, 1374w, 1361w, 1309w, 1249s, 1205w, 1140m, 1063s, 1028m, 1007s, 959w, 945w, 928w, 913w, 859s, 842s, 764m, 727m, 701s; δ_H (300 MHz) –0.02 (9H, s, $Si(CH_3)_3$), 0.34-0.57 (4H, stack, including [0.53, q, *J* 7.7, $SiCH_2CH_3$)], $Si(CH_2CH_3)_2$), 0.71-0.90 (9H, stack, $Si(CH_2CH_3)_2$, (CH_3)CHC=O), 1.62 (2H, d, *J* 8.1, $CH{=}CHCH_2$), 2.65-2.75 (1H, m, (CH_3)C*H*C=O), 4.79 (1H, d, *J* 7.7, C*H*OSi), 5.22 (1H, d, *J* 18.8, C*H*=$CHCH_2$), 6.08 (1H, dt, *J* 18.8, 7.7, CH=C*H*CH_2), 7.25-7.34 (5H, stack, Ph*H*), 9.80 (1H, d, *J* 2.9, C*H*O); δ_C (75 MHz) –1.9 (CH_3, $Si(CH_3)_3$), 5.4 (CH_2, $Si(CH_2CH_3)_2$), 6.5 (CH_3, $SiCH_2CH_3$), 6.6 (CH_3, $SiCH_2CH_3$), 11.0 (CH_3, (CH_3)CHC=O), 28.8 (CH_2, $CH{=}CHCH_2$), 54.4 (CH, (CH_3)*C*HC=O), 76.5 (CH, *C*HOSi), 122.3 (CH, *C*H=CH), 126.7 (CH, Ph), 127.7 (CH, Ph), 128.2 (CH, Ph), 149.3 (quat. C, *ipso*Ph), 147.8 (CH, *C*H=CH), 204.6 (CH, *C*=O); *m/z* (TOF ES+) 385.2 ($[M+Na]^+$, 100%); HRMS *m/z* (TOF ES+) 385.1992. $C_{20}H_{34}NaO_2Si_2$ requires 385.1995.

Allylation reaction of Aldehyde *syn*-4b

TMSOTf (80 µL, 0.47 mmol) was added to a solution of aldehyde ***syn*-4b** (150 mg, 0.41 mmol) and TTBP (134 mg, 0.54 mmol) in CH_2Cl_2 (5 mL) at –78 ^oC and the reaction was stirred for 24 h. The reaction was quenched by the addition of $NaHCO_3$ solution (5 mL) at –78 ^oC and then the reaction mixture was allowed to warm to room temperature over 30 min. The two phases were separated and the aqueous phase was extracted with CH_2Cl_2 (2 x 5 mL). The combined organic extracts were washed with water (5 mL) and brine (5 mL) and dried over $MgSO_4$. Filtration and evaporation under reduced pressure afforded a colourless oil. TTBP was removed by flash column chromatography (hexane) to afford diene ***syn*-13b** and 4 diastereoisomeric

oxasilacycles **16b**, **17b**, **18b** and **19b** (**16b**:**17b**:**18b**:**19b**:***syn*-13b**, 1:1:1:1:3) (134 mg, 89%). Flash column chromatography (10% toluene in hexane) afforded pure diene ***syn*-13b.** The oxasilacycles were inseparable by flash column chromatography. Analytically pure samples of each diastereoisomer were obtained by preparative HPLC.

(3*S, 4*R**, 5*R**, 6*S**)-2,2-Diethyl-5-methyl-6-phenyl-4-trimethylsilanyloxy-3-vinyl-[1,2]oxasilinane (16b)**

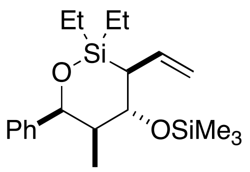

HPLC: t_R = 30.0 min; ν_{max}(film)/cm^{-1} 3583w, 3064w, 3027w, 2958s, 2913s, 2878s, 2361w, 1729w, 1629m (C=C), 1604w, 1496w, 1452m, 1413w, 1381m, 1310w, 1250s, 1204w, 1137m, 1105s, 1070s, 1001s, 947w, 891s, 841s, 742m, 700s, 666m, 620m; δ_H (500 MHz) 0.13 (9H, s, Si(CH_3)$_3$), 0.66-0.82 (7H, stack, including [0.72 (3H, d, *J* 7.3, CHCH_3)], Si(CH_2CH$_3$)$_2$, CHCH_3), 1.00 (3H, t, *J* 8.0, SiCH$_2$CH_3), 1.04 (3H, t, *J* 8.0, SiCH$_2$CH_3), 1.91-1.98 (1H, m, C*H*CH$_3$), 2.01 (1H, dd, *J* 10.5, 4.6, C*H*CH=CH$_2$), 3.98 (1H, app. t, *J* 4.6, C*H*OSiMe$_3$), 4.91 (1H, d with unresolved fine coupling, *J* 10.5, CH=CH_{cis}H$_{trans}$), 4.92 (1H, d with unresolved fine coupling, *J* 16.8, CH=CH$_{cis}$$H_{trans}$), 5.42 (1H, d, *J* 2.8, C*H*Ph), 5.87 (1H, dt, *J* 16.8, 10.5, C*H*=CH$_2$), 7.17-7.34 (5H, stack, Ph*H*); δ_C (100 MHz) 0.3 (CH$_3$, Si(CH$_3$)$_3$), 4.9 (CH$_2$, Si*C*H$_2$CH$_3$), 6.4 (CH$_3$, SiCH$_2$*C*H$_3$), 6.51 (CH$_2$, CH$_3$, Si*C*H$_2$*C*H$_3$, peaks coincident), 11.4 (CH$_3$, CH*C*H$_3$), 38.6 (CH, *C*HCH=CH$_2$), 45.4 (CH, *C*HCH$_3$), 72.1 (CH, *C*HPh), 77.5 (CH, *C*HOSiMe$_3$), 113.9 (CH$_2$, CH=*C*H$_2$), 125.7 (CH, Ph), 126.3 (CH, Ph), 127.8 (CH, Ph),

138.3 (CH, *C*H=CH$_2$), 143.5 (quat. C, *ipso*Ph); *m/z* (TOF ES+) 501.2 ([M+Ag+MeOH], 60%), 469.2 (100, [M+Ag]$^+$); HRMS *m/z* (TOF ES+) 469.1130. AgC$_{20}$H$_{34}$O$_2$Si$_2$ requires 469.1148.

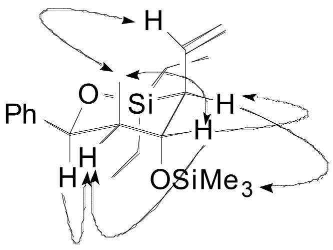

Selected nOes for oxasilacycle **16b**

(3*R, 4*R**, 5*R**, 6*S**)-2,2-Diethyl-5-methyl-6-phenyl-4-trimethylsilanyloxy-3-vinyl-[1,2]oxasilinane (17b)**

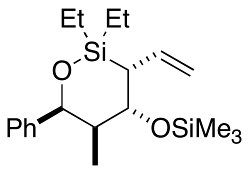

HPLC: t_R = 32.1 min; ν_{max}(film)/cm^{-1} 3583w, 3073w, 3028w, 2956s, 2878s, 2733w, 1946w, 1876w, 1806w, 1788w, 1625m (C=C), 1604w, 1495m, 1461m, 1452m, 1413m, 1379m, 1343m, 1309m, 1295w, 1251s, 1206m, 1152m, 1116m, 1062s, 997s, 958m, 923m, 872s, 841s, 754s, 729s, 700s, 666m, 646m, 624m; δ_H (300 MHz) 0.19 (9H, s, Si(CH$_3$)$_3$), 0.61-0.90 (7H, stack, including [0.64 (3H, d, *J* 7.3, CHC*H*$_3$)], Si(C*H*$_2$CH$_3$)$_2$, CHC*H*$_3$), 0.96 (3H, t, *J* 8.0, SiCH$_2$C*H*$_3$), 1.11 (3H, t, *J* 8.0, SiCH$_2$C*H*$_3$), 1.74-1.97 (1H, m, C*H*CH$_3$), 2.12 (1H, dd, *J* 9.8, 2.6, C*H*CH=CH$_2$), 4.08-4.12 (1H, m, C*H*OSiMe$_3$), 4.91 (1H, d, *J* 10.2, CH=C*H*$_{cis}$H$_{trans}$), 4.93 (1H, d, *J* 16.6,

CH=$CH_{cis}H_{trans}$), 5.47 (1H, d, *J* 1.8, C*H*Ph), 5.95 (1H, app. dt, *J* 16.6, 10.2, C*H*=CH_2), 7.16-7.35 (5H, stack, Ph*H*); δ_C (100 MHz) 0.4 (CH_3, Si(CH_3)$_3$), 4.6 (CH_2, SiCH_2CH_3), 5.5 (CH_2, SiCH_2CH_3), 6.7 (CH_3, SiCH_2CH_3), 6.8 (CH_3, SiCH_2CH_3), 9.5 (CH_3, CHCH_3), 32.5 (CH, *C*HCH=CH_2), 44.7 (CH, *C*HCH_3), 70.3 (CH, *C*HPh), 77.2 (CH, *C*HOSiMe$_3$), 112.9 (CH_2, CH=CH_2), 125.4 (CH, Ph), 126.2 (CH, Ph), 127.8 (CH, Ph), 137.9 (CH, *C*H=CH_2), 144.1 (quat. C, *ipso*Ph); *m/z* (TOF ES+) 469.4 ([M+Na]$^+$, 100%); HRMS *m/z* (TOF ES+) 469.1142. $AgC_{20}H_{34}O_2Si_2$ requires 469.1142.

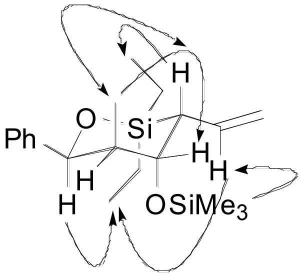

Selected nOes for oxasilacycle **17b**.

(3*S, 4*S**, 5*R**, 6*S**)-2,2-Diethyl-5-methyl-6-phenyl-4-trimethylsilanyloxy-3-vinyl-[1,2]oxasilinane (18b)**

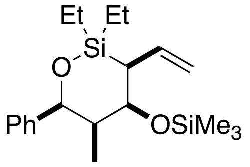

HPLC: t_R = 24.7 min; ν_{max}(film)/cm^{-1} 3852w, 3734w, 3648w, 3582w, 3419w, 3072w, 3027w, 2956s, 2877s, 1684w, 1652w, 1625m (C=C), 1604w, 1558w, 1540w, 1496w, 1456m, 1414m, 1384w, 1357m, 1322w, 1250s, 1204w, 1150m, 1102m, 1071s, 1003s, 947w, 882s, 843s, 742m, 700s, 666m, 634m, 619m; δ_H (500 MHz) 0.12 (9H,

s, Si(CH_3)$_3$), 0.70 (7H, stack, including [0.73 (3H, d, *J* 7.3, CHC*H*$_3$)], Si(C*H*$_2$CH$_3$)$_2$, CHC*H*$_3$), 1.02 (3H, t, *J* 7.8, SiCH$_2$C*H*$_3$), 1.06 (3H, t, *J* 8.0, SiCH$_2$C*H*$_3$), 1.99-2.09 (1H, m, C*H*CH$_3$), 2.22 (1H, dd, *J* 10.4, 6.6, C*H*CH=CH$_2$), 4.29 (1H, dd, *J* 6.6, 3.8, C*H*Ph), 4.80 (1H, d, *J* 16.7, CH=CH$_{cis}$*H*$_{trans}$), 4.90 (1H, d, *J* 10.4, CH=C*H*$_{cis}$H$_{trans}$), 5.01 (1H, d, *J* 2.0, C*H*Ph), 6.01 (1H, app. dt, *J* 16.7, 10.4, CH=C*H*$_2$), 7.17-7.36 (5H, stack, Ph*H*); δ_C (125 MHz) 0.2 (CH$_3$, Si(*C*H$_3$)$_3$), 4.7 (CH$_2$, Si*C*H$_2$CH$_3$), 5.7 (CH$_2$, Si*C*H$_2$CH$_3$), 6.3 (CH$_3$, SiCH$_2$*C*H$_3$), 6.9 (CH$_3$, SiCH$_2$*C*H$_3$), 7.6 (CH$_3$, CH*C*H$_3$), 38.3 (CH, *C*HCH=CH$_2$), 46.0 (CH, *C*HCH$_3$), 74.1 (CH, *C*HOSiMe$_3$), 75.7 (CH, *C*HPh), 114.0 (CH$_2$, CH=*C*H$_2$), 125.3 (CH, Ph), 126.5 (CH, Ph), 127.8 (CH, Ph), 137.3 (CH, *C*H=CH$_2$), 143.0 (quat. C, *ipso*Ph); *m/z* (TOF ES+) 501.2 ([M+Ag+MeOH]$^+$, 55%), 469.2 (100, [M+Ag]$^+$, 100%); HRMS *m/z* (TOF ES+) 469.1149. AgC$_{20}$H$_{34}$O$_2$Si$_2$ requires 469.1148.

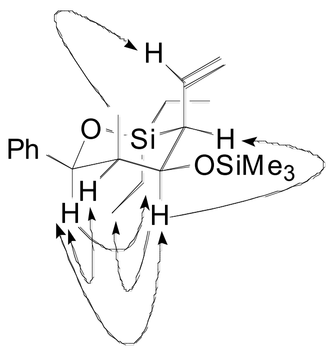

Selected nOes for oxasilacycle **18b**.

(3*R, 4*S**, 5*R**, 6*S**)-2,2-Diethyl-5-methyl-6-phenyl-4-trimethylsilanyloxy-3-vinyl-[1,2]oxasilinane (19b)**

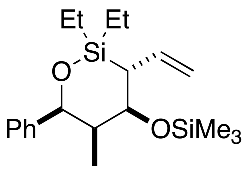

HPLC: t_R = 26.9 min; ν_{max}(film)/cm^{-1} 3583w, 3064w, 3072w, 2958s, 2913s, 2878s, 2630w, 1728w, 1630m (C=C), 1604w, 1496w, 1452m, 1413w, 1381m, 1310w, 1250s, 1204w, 1137m, 1105s, 1070s, 948w, 891s, 841s, 742m, 700s, 666m, 620m; δ_H (300 MHz) 0.13 (9H, s, Si(C*H*$_3$)$_3$), 0.64-0.87 (7H, stack, including [0.68 (3H, d, *J* 7.1, CHC*H*$_3$)], Si(C*H*$_2$CH$_3$)$_2$, CHC*H*$_3$), 0.99 (3H, t, *J* 8.0, SiCH$_2$C*H*$_3$), 1.13 (3H, t, *J* 8.0, SiCH$_2$C*H*$_3$), 1.18-2.06 (1H, m, C*H*CH$_3$), 2.24 (1H, app. t, *J* 10.6, C*H*CH=CH$_2$), 4.25 (1H, dd, *J* 11.5, 3.9, CHOSiMe$_3$), 4.92-5.01 (3H, stack, including [4.95 (1H, d, *J* 9.8, CH=C*H*$_{cis}$H$_{trans}$)], [4.97 (1H, d, *J* 16.8, CH=CH$_{cis}$*H*$_{trans}$)], C*H*Ph, CH=C*H*$_2$), 5.66 (1H, app. dt, *J* 16.8, 9.8, C*H*=CH$_2$), 7.19-7.37 (5H, stack, Ph*H*); δ_C (125 MHz) 0.4 (CH$_3$, Si(*C*H$_3$)$_3$), 3.8 (CH$_2$, Si*C*H$_2$CH$_3$), 4.0 (CH$_2$, Si*C*H$_2$CH$_3$), 4.5 (CH$_3$, CH*C*H$_3$), 6.6 (CH$_3$, SiCH$_2$*C*H$_3$), 7.0 (CH$_3$, SiCH$_2$*C*H$_3$), 35.6 (CH, *C*HCH=CH$_2$), 45.8 (CH, *C*HCH$_3$), 75.6 (CH, *C*HPh), 76.3 (CH, *C*HOSiMe$_3$), 113.9 (CH$_2$, CH=*C*H$_2$), 125.4 (CH, Ph), 126.5 (CH, Ph), 127.9 (CH, Ph), 137.7 (CH, *C*H=CH$_2$), 143.1 (quat. C, *ipso*Ph); *m/z* (TOF ES+) 501.2 ([M+Ag+MeOH]$^+$, 60%), 469.2 (100, [M+Ag]$^+$); HRMS *m/z* (TOF ES+) 469.1130. $AgC_{20}H_{34}O_2Si_2$ requires 469.1148.

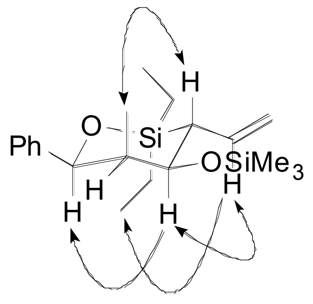

Selected nOes for oxasilacycle **19b**.

(*E*, 1*S**, 2*R**)-2-Methyl-1-phenyl-1-[(trimethylsilanyloxy)diethylsilanyloxy]-3,5-hexadiene (*syn*-13b)

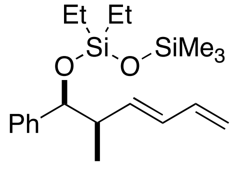

R_f = 0.20 (10 % toluene in hexane); ν_{max}(film)/cm^{-1} 3087w, 3084w, 3030w, 2958s, 2913s, 2877s, 2360w, 2248w, 1652w, 1603w, 1558w, 1540w, 1506w, 1494w, 1457m, 1414m, 1375w, 1252s, 1198w, 1066s, 955w, 909s, 842s, 736s, 701m; δ_H (300 MHz) –0.01 (9H, s, Si(C*H*$_3$)$_3$), 0.32-0.53 (4H, stack, including [0.51 (2H, q, *J* 7.7, SiC*H*$_2$CH$_3$)], Si(C*H*$_2$CH$_3$)$_2$), 0.77-1.00 (9H, stack, Si(CH$_2$C*H*$_3$)$_2$, CHC*H*$_3$), 2.47 (1H, app. sextet, *J* 6.2, C*H*CH$_3$), 4.63 (1H, d, *J* 6.0, C*H*OSi), 4.93 (1H, d, *J* 10.3, CH=C*H*$_{cis}$H$_{trans}$), 5.05 (1H, d, *J* 16.9, CH=C*H*$_{trans}$H$_{cis}$), 5.63 (1H, dd, *J* 15.4, 7.7, CH(CH$_3$)C*H*=CH), 5.91-5.99 (1H, m, C*H*CH=CH$_2$), 6.25 (1H, app. dt, *J* 16.9, 10.3, C*H*=CH$_2$), 7.19-7.29 (5H, stack, Ph*H*); δ_C (100 MHz) 1.7 (CH$_3$, Si(*C*H$_3$)$_3$), 6.1 (CH$_2$, Si*C*H$_2$CH$_3$), 6.2 (CH$_2$, Si*C*H$_2$CH$_3$), 6.4 (CH$_3$, SiCH$_2$*C*H$_3$), 6.5 (CH$_3$, SiCH$_2$*C*H$_3$), 14.8 (CH$_3$, CH*C*H$_3$), 45.0 (CH, *C*HCH$_3$), 77.8 (CH, *C*HOSi), 115.0 (CH$_2$, CH=*C*H$_2$), 126.7

(CH, Ph), 126.8 (CH, Ph), 127.6 (CH, Ph), 130.6 (CH, =*C*H), 137.4 (CH, =*C*H), 137.6 (CH, =*C*H), 133.5 (quat. C, *ipso*Ph); *m/z* (TOF ES+) 385.3 ([M+Na]$^+$, 100%); HRMS *m/z* (TOF ES+) 385.1979. $C_{20}H_{34}NaO_2Si_2$ requires 385.1995.

Allylation reaction of Aldehyde *anti*-4b

TMSOTf (160 μL, 0.94 mmol) was added to a solution of aldehyde ***anti*-4b** (300 mg, 0.83 mmol) and TTBP (268 mg, 1.08 mmol) in CH_2Cl_2 (10 mL) at –78 ^oC and the reaction was stirred for 10 h. The reaction was quenched by the addition of $NaHCO_3$ solution (10 mL) at –78 ^oC and then the reaction mixture was allowed to warm to room temperature over 30 min. The two phases were separated and the aqueous phase was extracted with CH_2Cl_2 (2 x 10 mL). The combined organic extracts were washed with water (10 mL) and brine (10 mL) and dried over $MgSO_4$. Filtration and evaporation under reduced pressure afforded a colourless oil. TTBP was removed by flash column chromatography (hexane) to afford diene ***anti*-13b** and 2 diastereoisomeric oxasilacycles **14b** and **15b** (**14b**:**15b**:***anti*-13b**, 4:1:3) (273 mg, 91%). Flash column chromatography (10% $PhCH_3$ in hexane) afforded pure diene ***anti*-13b.** The two oxasilacycles were inseparable by flash column chromatography. Analytically pure samples of each diastereoisomer were obtained by preparative HPLC.

(3*S, 4*R**, 5*S**, 6*S**)-2,2-Diethyl-5-methyl-6-phenyl-4-trimethylsilanyloxy-3-vinyl-[1,2]oxasilinane (14b)**

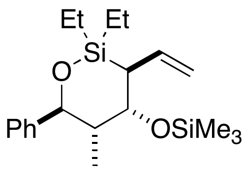

HPLC: t_R = 38.0 min; ν_{max}(film)/cm^{-1} 3074w, 3031w, 2958s, 2877s, 2362w, 1945w, 1874w, 1805w, 1805w, 1726w, 1710w, 1692w, 1658w, 1627m (C=C), 1604w, 1493w, 1454m, 1414m, 1378m, 1364m, 1332m, 1306w, 1286w, 1251s, 1209m, 1174m, 1133m, 1110m, 1084s, 1058s, 1021s, 971m, 932m, 887s, 875s, 841s, 746s, 724m, 699s, 665m, 645m, 610m; δ_H (500 MHz) 0.90 (9H, s, Si(C*H*$_3$)$_3$), 0.52-0.67 (5H, stack, including [0.54 (3H, d, *J* 6.8, CHC*H*$_3$)], SiC*H*$_2$CH$_3$, CHC*H*$_3$), 0.69-0.92 (8H, stack, including [0.89 (3H, t, *J* 8.0, SiCH$_2$C*H*$_3$)], [0.93 (3H, t, *J* 8.0, SiCH$_2$C*H*$_3$)], SiC*H*$_2$CH$_3$, Si(CH$_2$C*H*$_3$)$_2$), 1.87-1.96 (2H, stack, C*H*CH=CH$_2$, C*H*CH$_3$), 3.87 (1H, dd, *J* 4.1, 1.4, C*H*OSiMe$_3$), 5.75 (1H, d, *J* 9.4, C*H*Ph), 4.92 (1H, d, *J* 16.7, CH=CH$_{cis}$*H*$_{trans}$), 4.93 (1H, d, *J* 10.6, CH=C*H*$_{cis}$H$_{trans}$), 5.91 (1H, app. dt, *J* 16.7, 10.6, C*H*=CH$_2$), 7.18-7.31 (5H, stack, PhH); δ_C (125 MHz) 0.2 (CH$_3$, Si(*C*H$_3$)$_3$), 4.9 (CH$_2$, Si*C*H$_2$CH$_3$), 6.1 (CH$_3$, SiCH$_2$*C*H$_3$), 6.6 (CH$_3$, SiCH$_2$*C*H$_3$), 6.7 (CH$_2$, Si*C*H$_2$CH$_3$), 16.2 (CH$_3$, CH*C*H$_3$), 39.0 (CH, *C*HCH=CH$_2$), 40.8 (CH, *C*HCH$_3$), 76.1 (CH, *C*HPh), 77.8 (CH, *C*HOSiMe$_3$), 113.9 (CH$_2$, CH=*C*H$_2$), 127.0 (CH, Ph), 127.2 (CH, Ph), 128.1 (CH, Ph), 137.2 (CH, *C*H=CH$_2$), 144.3 (quat. C, *ipso*Ph); *m/z* (TOF ES+) 385.4 ([M+Na]$^+$, 100%); HRMS *m/z* (TOF ES+) 385.1992. $C_{20}H_{34}NaO_2Si_2$ requires 385.1995.

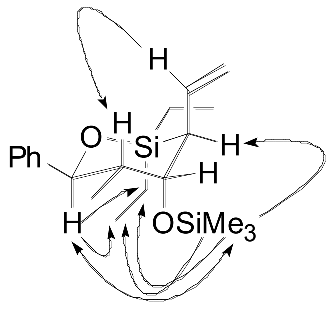

Selected nOes for oxasilacycle **14b**.

(3*R, 4*R**, 5*S**, 6*S**)-2,2-Diethyl-5-methyl-6-phenyl-4-trimethylsilanyloxy-3-vinyl-[1,2]oxasilinane (15b)**

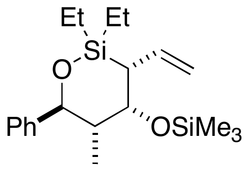

HPLC: t_R = 33.6 min; ν_{max}(film)/cm^{-1} 3074w, 3030w, 2959s, 2877s, 1940w, 1804w, 1754w, 1726w, 1710w, 1692w, 1658w, 1626m (C=C), 1604w, 1548w, 1536w, 1512w, 1492w, 1454m, 1415m, 1377w, 1361m, 1322m, 1284m, 1252s, 1209m, 1169w, 1145m, 1126m, 1094s, 1055s, 1026s, 948m, 882s, 840s, 792m, 735s, 699s, 666m, 653m, 617m; δ_H (500 MHz) 0.15 (9H, s, Si(C*H*$_3$)$_3$), 0.52-0.74 (6H, stack, including [0.54 (3H, d, *J* 7.1, CHC*H*$_3$)], 3H of Si(C*H*$_2$CH$_3$)$_2$, CHC*H*$_3$), 0.77-0.87 (1H, m, 1H of SiC*H*$_2$CH$_3$), 0.93 (3H, t, *J* 8.0, SiCH$_2$C*H*$_3$), 1.02 (3H, t, *J* 7.9, SiCH$_2$C*H*$_3$), 1.58-1.67 (1H, m, C*H*CH$_3$), 2.08 (1H, dd, *J* 10.3, 2.4, C*H*CH=CH$_2$), 4.03 (1H, d, *J* 1.9, C*H*OSiMe$_3$), 4.72 (1H, d, *J* 9.8, C*H*Ph), 4.89 (1H, d with unresolved fine coupling, *J* 10.3 , CH=C*H*$_{cis}$H$_{trans}$), 4.92 (1H, d with unresolved fine coupling, *J* 17.4, CH=CH$_{cis}$*H*$_{trans}$), 5.90 (1H, app. dt, *J* 17.4, 10.3, C*H*=CH$_2$), 7.18-7.31 (5H, stack, Ph*H*); δ_C (125 MHz) 0.8 (CH$_3$, Si(*C*H$_3$)$_3$), 4.5 (CH$_2$, Si*C*H$_2$CH$_3$), 6.0 (CH$_2$, Si*C*H$_2$CH$_3$),

6.5 (CH_3, $SiCH_2CH_3$), 6.6 (CH_3, $SiCH_2CH_3$), 16.6 (CH_3, $CHCH_3$), 40.0 (CH, *C*HCH=CH_2), 45.4 (CH, *C*HCH_3), 75.9 (CH, *C*HPh), 78.6 (CH, *C*HOSiMe_3), 113.0 (CH_2, CH=*C*H_2), 126.8 (CH, Ph), 127.1 (CH, Ph), 127.9 (CH, Ph), 137.9 (CH, *C*H=CH_2), 144.0 (quat. C, *ipso*Ph); *m/z* (TOF ES+) 385.4 ($[M+Na]^+$, 100%); HRMS *m/z* (TOF ES+) 385.1999. $C_{20}H_{34}NaO_2Si_2$ requires 385.1995.

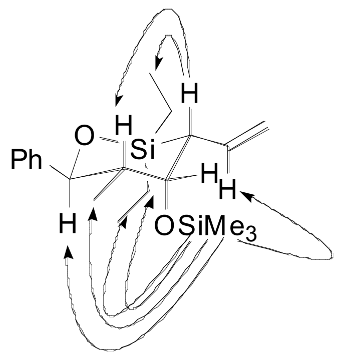

Selected nOes for oxasilacycle **15b**.

(*E*, 1*S, 2*S**)-2-Methyl-1-phenyl-1-[(trimethylsilanyloxy)diethylsilanyloxy]-3,5-hexadiene (*anti*-13b)**

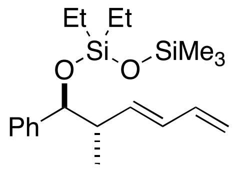

R_f = 0.22 (10 % toluene in hexane); ν_{max}(film)/cm^{-1} 3087w, 3065w, 3030m, 2958s, 2914s, 2878s, 2248w, 1944w, 1801w, 1734w, 1652w, 1604m, 1494m, 1454s, 1415m, 1374m, 1310w, 1252s, 1200w, 1062s, 1028s, 1007s, 954m, 909s, 842s, 736s, 701s; δ_H (300 MHz) 0.00 (9H, s, Si(CH_3)$_3$), 0.37 (2H, q, *J* 7.9, SiCH_2CH_3), 0.47 (2H, q, *J* 7.9, SiCH_2CH_3), 0.78 (3H, t, *J* 8.1, $SiCH_2CH_3$), 0.86-0.93 (6H, stack,

$SiCH_2CH_3$, $CHCH_3$), 2.48 (1H, app. sextet, *J* 7.0, C*H*CH_3), 4.57 (1H, d, *J* 6.2, C*H*OSi), 4.94 (1H, d, *J* 10.2, CH=C$H_{cis}H_{trans}$), 5.05 (1H, d, *J* 16.9, CH=$CH_{cis}H_{trans}$), 5.69 (1H, dd, *J* 15.1, 7.7, $CH(CH_3)CH$=CH), 5.92-6.01 (1H, m, C*H*CH=CH_2), 6.28 (1H, dt, *J* 16.9, 10.2, C*H*=CH_2), 7.20-7.31 (5H, stack, Ph*H*); δ_C (100 MHz) 1.7 (CH_3, $Si(CH_3)_3$), 6.1 (CH_2, $SiCH_2CH_3$), 6.2 (CH_2, $SiCH_2CH_3$), 6.4 (CH_3, $SiCH_2CH_3$), 6.5 (CH_3, $SiCH_2CH_3$), 16.2 (CH_3, $CHCH_3$), 45.1 (CH, *C*HCH_3), 78.2 (CH, *C*HOSi), 115.9 (CH_2, CH=CH_2), 126.8 (CH, Ph), 126.9 (CH, Ph), 127.6 (CH, Ph), 130.9 (CH, =*C*H), 137.4 (CH, =*C*H), 137.5 (CH, =*C*H), 143.6 (quat. C, *ipso*Ph); *m/z* (TOF ES+) 385.0 ($[M+Na]^+$, 100%).

(2*S,3*R**)-*tert*-butyl 3-hydroxy-2-methyl-heptanoate (*syn*-9a) and (2*R**, 3*R**)-*tert*-butyl 3-hydroxy-2-methyl-heptanoate (*anti*-9a)**

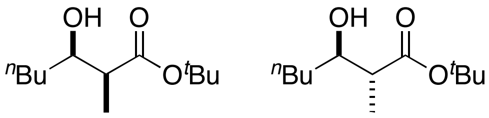

***syn*-9a** ***anti*-9a**

^{n}BuLi (5.86 mL, 14.66 mmol, 2.5 M in hexane) was added dropwise over 10 min to a stirred solution of di*iso*propylamine (2.04 mL, 14.66 mmol) in THF (90 mL) at 0 ^oC and the reaction mixture was stirred at this temperature for 20 min. The reaction mixture was then cooled to –78 ^oC before a solution of *tert*-butyl propionate (2.20 mL, 14.66 mmol) in THF (4 mL) was added dropwise over 5 min. After 1 h at –78 ^oC, valeraldehyde (1.56 mL, 14.66 mmol) was added. After stirring for 15 min, NH_4Cl solution (80 mL) was added and the solution was warmed to room temperature. The layers were separated and the aqueous phase was extracted with EtOAc (3 x 80 mL). The combined organic extracts were washed with brine (40 mL), dried

($MgSO_4$) and concentrated under reduced pressure to afford a 50:50 mixture of the two diastereoisomeric β-hydroxy esters which were separated by flash column chromotography (10% Et_2O in hexane) to provide the pure diastereoisomeric aldol products ***syn*-9a** and ***anti*-9a**, both as colourless oils (2.64 g combined, 83%).

(2*S**,3*R**)-*tert*-butyl 3-hydroxy-2-methyl-heptanoate (*syn*-9a)

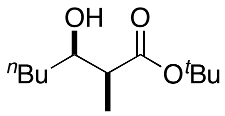

R_f = 0.12 (8% Et_2O in hexane); ν_{max}(film)/cm^{-1} 3451s br (OH), 2960s, 2995s, 2874s, 1729s (C=O), 1641w, 1548w, 1512w, 1460s, 1392m, 1368s, 1257m, 1212m, 1157s, 1124s, 1027m, 932w, 898w, 849m, 751w, 666m; δ_H (300 MHz) 0.89 (3H, t, *J* 7.0, $(CH_2)_3CH_3$), 1.17 (3H, d, *J* 7.4, $CHCH_3$), 1.19-1.53 (15H, stack, including [9H, s, $C(CH_3)_3$)], 3 x CH_2, $C(CH_3)_3$), 2.39 (1H, app. quintet, *J* 7.4, $CHCH_3$), 2.72 (1H, d, *J* 6.6, O*H*), 3.52-3.64 (1H, m, C*H*OH); δ_C (75 MHz) 14.0 (CH_3), 14.4 (CH_3), 22.6 (CH_2, CH_2CH_3), 27.8 (CH_2, $CH_2CH_2CH_3$), 28.1 (CH_3, $C(CH_3)_3$), 34.6 (CH_2, $CH_2(CH_2)_2CH_3$), 45.8 (CH, $CHCH_3$), 73.5 (CH, *C*HOH), 81.0 (quat. C, $C(CH_3)_3$), 175.6 (quat. C, *C*=O); *m/z* (TOF ES+) 239.1 ($[M+Na]^+$, 100%); HRMS *m/z* (TOF ES+) 239.1616. $C_{12}H_{24}NaO_3$ requires 239.1623.

(2*R**,3*R**)-*tert*-butyl 3-hydroxy-2-methyl-heptanoate (*anti*-9a)

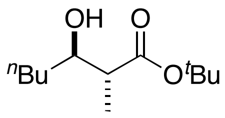

R_f = 0.10 (8% Et_2O in hexane); ν_{max}(film)/cm^{-1} 3442s br, 2959s, 2935s, 2874s, 2362w, 1726s (C=O), 1548w, 1530w, 1512w, 1459s, 1392m, 1368s, 1257s, 1215m, 1157s, 1028s, 975m, 932w, 900m, 850m, 755w, 731w, 666m; δ_H (300 MHz) 0.90 (3H, t, *J* 7.0, $(CH_2)_3CH_3$), 1.13 (3H, d, *J* 7.0, $CHCH_3$), 1.21-1.61 (15H, stack, including [9H, s, $C(CH_3)_3$)], 3 x CH_2, $C(CH_3)_3$), 2.39 (1H, qd, *J* 7.4, 3.3, $CHCH_3$), 2.64 (1H, s, O*H*), 3.78-3.86 (1H, m, C*H*OH); δ_C (75 MHz) 10.8 (CH_3), 13.9 (CH_3), 22.6 (CH_2), 27.9 (CH_3, $C(CH_3)_3$), 28.1 (CH_2), 33.5 (CH_2), 45.0 (CH, $(CH_3)CHC$=O), 71.8 (CH, *C*HOH), 80.7 (quat. C, $C(CH_3)_3$), 175.7 (quat. C, *C*=O); *m/z* (TOF ES+) 239.2 ($[M+Na]^+$, 100%); HRMS *m/z* (TOF ES+) 239.1621. $C_{12}H_{24}NaO_3$ requires 239.1623.

(*E*, 2*S**,3*R**)-*tert*-butyl 3-[diethyl-(3'-trimethylsilanyl-prop-1'-enyl)-silanyloxy]-2-methyl-heptanoate (*syn*-12a)

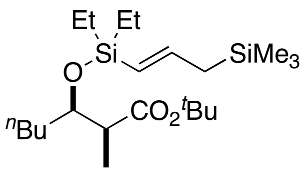

Alcohol ***syn*-9a** (700 mg, 3.24 mmol) and aminosilane **10** (882 mg, 3.24 mmol) were stirred at 40 ^oC for 2 d. Evaporation of Et_2NH and purification of the residue by flash column chromotography (2 % ether in hexane) afforded silyl ether ***syn*-12a** as a

colourless oil (1.021 g, 76%); R_f = 0.24 (2% Et_2O in hexane); (Found: C, 63.66; H, 11.16. $C_{22}H_{46}O_3Si_2$ requires C, 63.71; H, 11.18); ν_{max}(film)/cm^{-1} 3583w, 2956s, 2876s, 2732w, 2362w, 2257w, 1728s (C=O), 1602s (C=C), 1458s, 1414m, 1392m, 1367s, 1249s, 1157s, 1113s, 1057s, 1008s, 960w, 909m, 858s, 764m, 735s, 667m, 648w; δ_H (300 MHz) 0.00 (9H, s, Si(C*H*$_3$)$_3$), 0.58-0.66 (4H, stack, Si(C*H*$_2$CH$_3$)$_2$), 0.84-1.00 (9H, stack, including [0.88 (3H, t, *J* 7.0, (CH$_2$)$_3$C*H*$_3$)], [0.94 (6H, t, *J* 8.1, Si(CH$_2$C*H*$_3$)$_2$)], (CH$_2$)$_3$C*H*$_3$), Si(CH$_2$C*H*$_3$)$_2$), 1.09 (3H, d, *J* 7.0, CHC*H*$_3$), 1.12-1.51 (15H, stack, including [1.43 (9H, s, C(C*H*$_3$)$_3$)], 3 x C*H*$_2$, C(C*H*$_3$)$_3$), 1.66 (2H, d, *J* 7.9, C*H*$_2$SiMe$_3$), 2.39 (1H, app. quintet, *J* 7.0, C*H*CH$_3$), 3.79-3.91 (1H, m, C*H*OSi), 5.38 (1H, d, *J* 18.5, SiC*H*=CH), 6.14 (1H, dt, *J* 18.5, 7.9, SiCH=C*H*); δ_C (100 MHz) -2.0 (CH$_3$, Si(*C*H$_3$)$_3$), 5.7 (CH$_2$, Si*C*H$_2$CH$_3$), 5.9 (CH$_2$, Si*C*H$_2$CH$_3$), 6.9 (CH$_3$, Si(CH$_2$*C*H$_3$)$_2$), 13.2 (CH$_3$), 14.0 (CH$_3$), 22.9 (CH$_2$, CH=CH*C*H$_2$), 27.0 (CH$_2$), 28.0 (CH$_3$, C(*C*H$_3$)$_3$), 28.7 (CH$_2$), 35.0 (CH$_2$), 46.2 (CH, (CH$_3$)*C*HC=O), 73.8 (CH$_3$, *C*HOSi), 79.7 (quat. C, *C*(CH$_3$)$_3$), 123.5 (CH, *C*H=CH), 146.7 (CH, *C*H=CH), 174.6 (quat. C, *C*=O); *m/z* (TOF ES+) 437.3 ([M+Na]$^+$, 100%); HRMS *m/z* (TOF ES+) 437.2869. $C_{22}H_{46}NaO_3Si_2$ requires 437.2883.

(*E*, 2*R,3*R**)-*tert*-butyl 3-[diethyl-(3'-trimethylsilanyl-prop-1'-enyl)-silanyloxy]-2-methyl-heptanoate (*anti*-12a)**

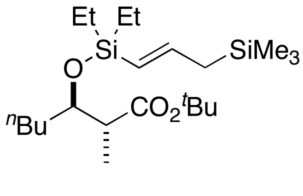

Alcohol ***anti*-9a** (500 mg, 2.41 mmol) and aminosilane **10** (656 mg, 2.41 mmol) were stirred at 40 °C for 2 d. Evaporation of Et_2NH and purification of the residue by flash column chromatography (2 % ether in hexane) afforded silyl ether ***anti*-12a** as a colourless, highly viscous oil (0.770 g, 77%); R_f = 0.24 (2% Et_2O in hexane); (Found: C, 63.49; H, 11.43. $C_{22}H_{46}O_3Si_2$ requires C, 63.71; H, 11.18); ν_{max}(film)/cm^{-1} 3583w, 2956s, 2876s, 2631w, 2341w, 2257w, 1732s (C=O), 1602m (C=C), 1459m, 1414w, 1392m, 1367m, 1313w, 1249s, 1158s, 1125m, 1084m, 1058m, 1000m, 959s, 933s, 910s, 859s, 815m, 764m, 734m, 667m; δ_H (300 MHz) 0.00 (9H, s, Si(CH_3)$_3$), 0.62 (4H, q, *J* 8.1, Si(CH_2CH$_3$)$_2$), 0.82-0.98 (9H, stack, including [0.87 (3H, t, *J* 6.2, (CH$_2$)$_3$CH_3)], [0.94 (6H, t, *J* 8.1, Si(CH$_2$CH_3)$_2$)], (CH$_2$)$_3$CH_3, Si(CH$_2$CH_3)$_2$), 1.03 (3H, d, *J* 7.0, CHCH_3), 1.11-1.42 (15H, stack, including [1.43 (9H, s, C(CH_3)$_3$)], 3 x CH_2, C(CH_3)$_3$), 1.66 (2H, d, *J* 8.1, CH_2SiMe$_3$), 2.39 (1H, qd, *J* 7.0, 5.5, C*H*CH$_3$), 3.94-4.25 (1H, m, C*H*OSi), 5.39 (1H, d, *J* 18.6, SiC*H*=CH), 6.14 (1H, dt, *J* 18.6, 8.1, SiCH=C*H*); δ_C (100 MHz) -2.0 (CH$_3$, Si(*C*H$_3$)$_3$), 5.5 (CH$_2$, Si*C*H$_2$CH$_3$), 5.6 (CH$_2$, Si*C*H$_2$CH$_3$), 6.8 (CH$_3$, Si(CH$_2$*C*H$_3$)$_2$), 11.0 (CH$_3$), 14.0 (CH$_3$), 22.7 (CH$_2$, CH=CH*C*H$_2$), 27.4 (CH$_2$), 28.0 (CH$_3$, C(*C*H$_3$)$_3$), 28.7 (CH$_2$), 32.6 (CH$_2$), 46.3 (CH, (CH$_3$)*C*HC=O), 73.2 (CH$_3$, *C*HOSi), 79.6 (quat. C, *C*(CH$_3$)$_3$), 123.4 (CH, *C*H=CH), 146.7 (CH, *C*H=CH), 173.7 (quat. C, *C*=O); *m/z* (TOF ES+) 437.0 ([M+Na]$^+$, 100%); HRMS *m/z* (TOF ES+) 437.2882. $C_{22}H_{46}NaO_3Si_2$ requires 437.2883.

(*E*, 2*S,3*R**)-3-[Diethyl-(3-trimethylsilanyl-prop-1'-enyl)-silanyloxy]-2-methyl-heptanal (*syn*-4a)**

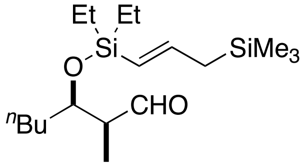

DIBALH (1.78 mL, 1.5 M in toluene, 2.66 mmol) was added dropwise over 30 min to a solution of ester ***syn*-12a** (500 mg, 1.21 mmol) in CH_2Cl_2 (9 mL) at –78 °C. After 2 h, the reaction was quenched by the sequential addition of MeOH (107 μL, 2.66 mmol) and H_2O (290 μL, 15.96 mmol) at –78 °C. The resulting slurry was allowed to warm to rt and then filtered through $MgSO_4$ and Celite. The solvent was evaporated under reduced pressure to leave a yellow liquid which was dissolved in CH_2Cl_2 (12 mL). Dess-Martin periodinane (0.774 g, 1.82 mmol) was added and the reaction mixture was stirred for 1 h. The reaction was then quenched by the addition of $NaHCO_3$ solution (6 mL) and sodium thiosulfate solution (6 mL) and stirred for a further 30 min. The phases were separated and the aqueous phase was extracted with CH_2Cl_2 (2 x 12 mL). The combined organic extracts were washed with water (12 mL) and brine (12 mL) and dried over $MgSO_4$. Filtration and evaporation under reduced pressure produced a yellow liquid which was purified by flash column chromatography (2% Et_2O in hexane) to afford aldehyde ***syn*-4a** as a colourless liquid (0.299 g, 72%); R_f = 0.19 (2% Et_2O in hexane); ν_{max}(film)/cm^{-1} 2956s, 2876s, 2706w, 1728s (C=O), 1602s (C=C), 1460m, 1414m, 1380s, 1249s, 1141m, 1100m, 1031m, 958w, 859s, 816m, 765m, 725m, 666m; δ_H (300 MHz) 0.00 (9H, s, Si(C*H*$_3$)$_3$), 0.52-0.67 (4H, stack, Si(C*H*$_2$CH$_3$)$_2$), 0.86-0.97 (9H, stack, (CH$_2$)$_3$C*H*$_3$, Si(CH$_2$C*H*$_3$)$_2$), 1.03 (3H, d, *J* 6.8, CHC*H*$_3$), 1.13-1.51 (6H, stack, 3 x C*H*$_2$), 1.67 (2H,

d, *J* 8.0, C*H*$_2$SiMe$_3$), 2.43 (1H, qd, *J* 6.8, 3.7, C*H*CH$_3$), 4.07-4.54 (1H, m, C*H*OSi), 5.35 (1H, d, *J* 18.8, SiC*H*=CH), 6.15 (1H, dt, *J* 18.8, 8.0, SiCH=C*H*), 9.75 (1H, s, C*H*O); δ_C (100 MHz) -2.0 (CH$_3$, Si(*C*H$_3$)$_3$), 5.6 (CH$_2$, Si*C*H$_2$CH$_3$), 5.7 (CH$_2$, Si*C*H$_2$CH$_3$), 6.7 (CH$_3$, SiCH$_2$*C*H$_3$), 6.8 (CH$_3$, SiCH$_2$*C*H$_3$), 7.7 (CH$_3$), 14.0 (CH$_3$), 22.6 (CH$_2$, CH=CH*C*H$_2$), 27.9 (CH$_2$), 28.8 (CH$_2$), 34.3 (CH$_2$), 51.4 (CH, (CH$_3$)*C*HC=O), 72.2 (CH, *C*HOSi), 122.9 (CH, *C*H=CH), 147.5 (CH, *C*H=CH), 205.3 (quat. C, *C*=O); *m/z* (TOF ES+) 381.2 ([M+K]$^+$, 100%); HRMS *m/z* (TOF ES+) 381.2058. $C_{18}KH_{38}O_2Si_2$ requires 381.2047.

(*E*, 2*R,3*R**)-3-[Diethyl-(3-trimethylsilanyl-prop-1'-enyl)-silanyloxy]-2-methyl-heptanal (*anti*-4a)**

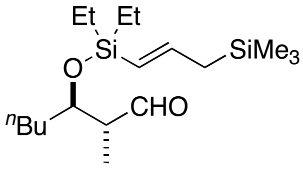

DIBALH (1.48 mL, 1.5 M in toluene, 2.22 mmol) was added dropwise over 30 min to a solution of ester ***anti*-12a** (0.417 g, 1.00 mmol) in CH$_2$Cl$_2$ (8 mL) at –78 °C. After 2 h, the reaction was quenched by the sequential addition of MeOH (89 μL, 2.22 mmol) and H$_2$O (242 μL, 13.30 mmol) at –78 °C. The resulting slurry was allowed to warm to rt and then filtered through MgSO$_4$ and Celite. The solvent was evaporated under reduced pressure to leave a yellow liquid which was dissolved in CH$_2$Cl$_2$ (10 mL). Dess-Martin periodinane (0.645 g, 1.52 mmol) was added and the reaction mixture was stirred for 1 h. The reaction was then quenched by the addition of NaHCO$_3$ solution (5 mL) and sodium thiosulfate solution (5 mL) and stirred for a further 30 min. The phases were separated and the aqueous phase was extracted

with CH_2Cl_2 (2 x 12 mL). The combined organic extracts were washed with water (10 mL) and brine (10 mL) and dried over $MgSO_4$. Filtration and evaporation under reduced pressure produced a yellow liquid which was purifed by flash column chromatography (2% Et_2O in hexane) to afford aldehyde ***anti*-4a** as a colourless liquid (0.578 g, 76%); R_f = 0.19 (2% Et_2O in hexane); ν_{max}(film)/cm^{-1} 3583w, 2956s, 2876s, 2707w, 2361w, 1727s (C=O), 1692w, 1658w, 1641w, 1602s (C=C), 1548w, 1530w, 1513w, 1461m, 1413m, 1379m, 1249s, 1141m, 1100m, 1032s, 958w, 859s, 816m, 765m, 725m, 666m; δ_H (300 MHz) 0.00 (9H, s, Si(CH_3)$_3$), 0.56-0.68 (4H, stack, Si(CH_2CH$_3$)$_2$), 0.85-0.98 (9H, stack, (CH$_2$)$_3$CH_3, Si(CH$_2$CH_3)$_2$), 1.05 (3H, d, *J* 7.0, CHCH_3), 1.15-1.53 (6H, stack, 3 x CH_2), 1.67 (2H, d with unresolved fine coupling, *J* 8.1, CH_2SiMe$_3$), 2.42-2.54 (1H, m, C*H*CH$_3$), 4.10 (1H, app q, *J* 5.5, C*H*OSi), 5.36 (1H, d with unresolved fine coupling, *J* 18.8, SiC*H*=CH), 6.16 (1H, dt, *J* 18.8, 8.1, SiCH=C*H*), 9.72 (1H, d, *J* 2.2, C*H*O); δ_C (100 MHz) -2.5 (CH$_3$, Si(*C*H$_3$)$_3$), 5.1 (CH$_2$, SiCH_2CH$_3$), 5.2 (CH$_2$, SiCH_2CH$_3$), 6.3 (CH$_3$, SiCH$_2$*C*H$_3$), 6.4 (CH$_3$, SiCH$_2$*C*H$_3$), 9.9 (CH$_3$), 13.6 (CH$_3$), 22.4 (CH$_2$, CH=CH*C*H$_2$), 26.7 (CH$_2$), 28.6 (CH$_2$), 34.3 (CH$_2$), 51.2 (CH, (CH$_3$)*C*HC=O), 73.4 (CH, *C*HOSi), 123.1 (CH, *C*H=CH), 148.0 (CH, *C*H=CH), 205.9 (quat. C, *C*=O); *m/z* (TOF ES+) 361.1 ([M+Na]$^+$, 100%); HRMS *m/z* (TOF ES+) 365.2299. $C_{18}H_{38}NaO_2Si_2$ requires 365.2308.

Allylation reaction of Aldehyde *syn*-4a

TMSOTf (90 μL, 0.53 mmol) was added to a solution of aldehyde ***syn*-4a** (158 mg, 0.46 mmol) and TTBP (151 mg, 0.61 mmol) in CH_2Cl_2 (6 mL) at –78 ^oC and the reaction mixture was stirred for 24 h. The reaction was then quenched by the addition of $NaHCO_3$ solution (6 mL) at –78 ^oC and then the resulting mixture was allowed to warm to room temperature over 30 min. The two phases were separated

and the aqueous phase was extracted with CH_2Cl_2 (2 x 6 mL). The combined organic extracts were washed with water (6 mL) and brine (6 mL) and dried over $MgSO_4$. Filtration and evaporation under reduced pressure afforded a colourless oil. TTBP was removed by flash column chromatography (hexane) to afford diene ***syn*-13a** and four diastereoisomeric oxasilacycles **16a**,**17a**,**18a** and **19a** (**16a**:**17a**:**18a**:**19a**:***syn*-13a**, 1:1:1:1:5) (148 mg, 94%). Flash column chromatography (10% toluene in hexane) afforded pure diene ***syn*-13a**. The oxasilacycles were inseparable by flash column chromatography. Analytically pure samples of each diastereoisomer were obtained by preparative HPLC.

(3*S**, 4*R**, 5*R**, 6*R**)-6-Butyl-2,2-diethyl-5-methyl-4-trimethylsilanyloxy-3-vinyl-[1,2]oxasilinane (16a)

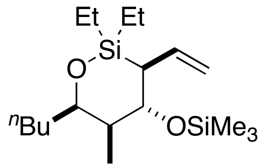

HPLC: t_R = 52.5 min; ν_{max}(film)/cm^{-1} 3583w, 3075w, 2957s, 2935s, 2877s, 2732w, 2361w, 2342w, 1792w, 1734w, 1670w, 1664w, 1653w, 1626m (C=C), 1559w, 1540w, 1507w, 1459m, 1415m, 1379m, 1323w, 1250s, 1134m, 1088s, 1064s, 1019s, 941w, 896m, 875m, 840s, 746m, 727m, 684m, 666m, 617m; δ_H (500 MHz) 0.11 (9H, s, Si(CH_3)$_3$), 0.55-0.73 (4H, m, Si(CH_2CH$_3$)$_2$), 0.88-0.93 (6H, stack, (CH$_2$)$_3$CH_3, CHCH_3), 0.96 (3H, t, *J* 7.8, SiCH$_2$CH_3), 0.99 (3H, t, *J* 7.8, SiCH$_2$CH_3), 1.21-1.54 (6H, stack, (CH_2)$_3$CH$_3$), 1.78-1.92 (1H, m, C*H*CH$_3$), 2.02 (1H, app. t, *J* 10.2, C*H*CH=CH$_2$), 3.82 (1H, app. t, *J* 8.9, C*H*OSiMe$_3$), 3.91 (1H, d with unresolved fine coupling, *J* 10.4, C*H*(CH$_2$)$_3$CH$_3$), 4.90 (1H, d, *J* 10.2, CH=CH_{cis}H$_{trans}$), 4.91 (1H,

d, *J* 16.7, CH=CH$_{cis}$*H*$_{trans}$), 5.67 (1H, app. dt, *J* 16.7, 10.2, C*H*=CH$_2$); δ$_C$ (125 MHz) 0.8 (CH$_3$, Si(*C*H$_3$)$_3$), 4.9 (CH$_2$, Si*C*H$_2$CH$_3$), 6.4 (CH$_2$, SiC*H*$_2$CH$_3$), 6.5 (CH$_3$, SiCH$_2$*C*H$_3$), 6.9 (CH$_3$, SiCH$_2$*C*H$_3$), 14.1 (CH$_3$, (CH$_2$)$_3$*C*H$_3$), 14.6 (CH$_3$, CH*C*H$_3$), 22.7 (CH$_2$, *C*H$_2$CH$_3$), 29.0 (CH$_2$, C*H*$_2$CH$_2$CH$_3$), 31.7 (CH$_2$, *C*H$_2$(CH$_2$)$_2$CH$_3$), 42.0 (CH, *C*HCH=CH$_2$), 44.5 (CH, *C*HCH$_3$), 75.1 (CH, *C*HOSiMe$_3$), 76.4 (CH, *C*H(CH$_2$)$_3$CH$_3$), 114.2 (CH$_2$, CH=*C*H$_2$), 138.3 (CH, *C*H=CH$_2$); *m/z* (TOF ES+) 365.2 ([M+Na]$^+$, 100%); HRMS *m/z* (TOF ES+) 365.2310. $C_{18}H_{38}NaO_2Si_2$ requires 365.2308.

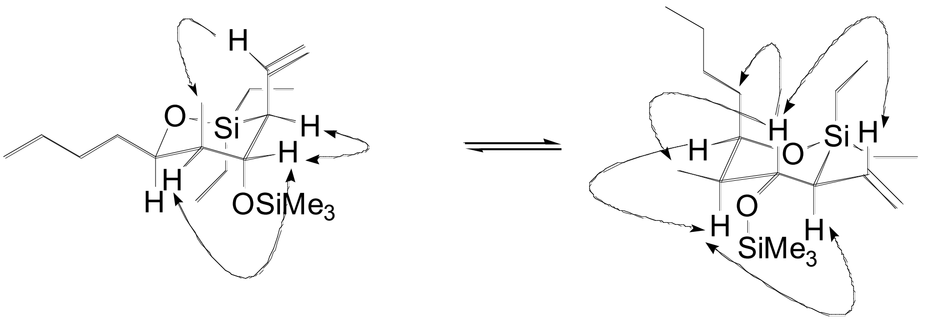

Selected nOes for oxasilacycle **16a** (conformationally very flexible).

(3*R**, 4*R**, 5*R**, 6*R**)-6-Butyl-2,2-diethyl-5-methyl-4-trimethylsilanyloxy-3-vinyl-[1,2]oxasilinane (17a)

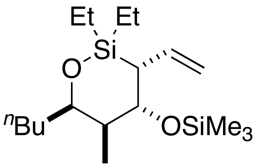

HPLC: t_R = 51.3 min; ν_{max}(film)/cm^{-1} 3583w, 3075w, 2957s, 2935s, 2876s, 2360w, 2342w, 1793w, 1734w, 1700w, 1684w, 1653w, 1625m (C=C), 1559w, 1540w, 1507w, 1458m, 1416w, 1380m, 1363w, 1342w, 1252s, 1176w, 1131m, 1070s, 1005s, 988s, 945m, 896m, 880s, 841s, 811m, 741s, 725s, 666m, 627m; δ$_H$ (500

MHz) 0.09 (9H, s, Si(C*H*$_3$)$_3$), 0.48-0.69 (4H, stack, Si(C*H*$_2$CH$_3$)$_2$), 0.84 (3H, d, *J* 7.1, CHC*H*$_3$), 0.89 (3H, t, *J* 7.1, (CH$_2$)$_3$C*H*$_3$), 0.94 (3H, t, *J* 8.0, SiCH$_2$C*H*$_3$), 0.98 (3H, t, *J* 8.0, SiCH$_2$C*H*$_3$), 1.18-1.60 (7H, stack, (C*H*$_2$)$_3$CH$_3$, C*H*CH$_3$), 2.00 (1H, dd, *J* 10.2, 2.7, C*H*CH=CH$_2$), 3.95 (1H, app. t, *J* 3.3, C*H*OSiMe$_3$), 4.20 (1H, app t. with unresolved fine coupling, *J* 6.0, C*H*(CH$_2$)$_3$CH$_3$), 4.85 (1H, d with unresolved fine coupling, *J* 10.2, CH=CH_{cis}H$_{trans}$), 4.88 (1H, d with unresolved fine coupling, *J* 17.6, CH=CH$_{cis}$$H_{trans}$), 5.87 (1H, app. dt, *J* 17.6, 10.2, C*H*=CH$_2$); δ_C (125 MHz) 0.3 (CH$_3$, Si(*C*H$_3$)$_3$), 4.6 (CH$_2$, Si*C*H$_2$CH$_3$), 5.1 (CH$_2$, SiC*H*$_2$CH$_3$), 6.6 (CH$_3$, SiCH$_2$*C*H$_3$), 6.8 (CH$_3$, SiCH$_2$*C*H$_3$), 9.4 (CH$_3$, CH*C*H$_3$), 14.1 (CH$_3$, (CH$_2$)$_3$*C*H$_3$), 22.7 (CH$_2$, *C*H$_2$CH$_3$), 28.3 (CH$_2$, C*H*$_2$CH$_2$CH$_3$), 32.8 (CH, *C*HCH=CH$_2$), 34.7 (CH$_2$, *C*H$_2$(CH$_2$)$_2$CH$_3$), 41.5 (CH, *C*HCH$_3$), 68.5 (CH, *C*H(CH$_2$)$_3$CH$_3$), 77.3 (CH, *C*HOSiMe$_3$), 112.6 (CH$_2$, CH=*C*H$_2$), 138.3 (CH, *C*H=CH$_2$); *m/z* (TOF ES+) 365.3 ([M+Na]$^+$, 100%); HRMS *m/z* (TOF ES+) 365.2322. $C_{18}H_{38}NaO_2Si_2$ requires 365.2308.

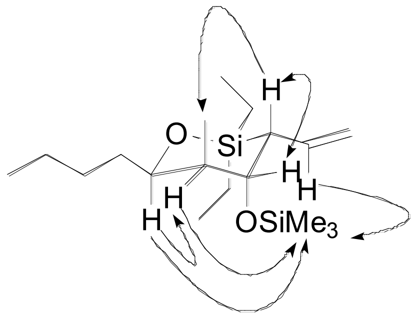

Selected nOes for oxasilacycle **17a**.

(3*S, 4*S**, 5*R**, 6*R**)-6-Butyl-2,2-diethyl-5-methyl-4-trimethylsilanyloxy-3-vinyl-[1,2]oxasilinane (18a)**

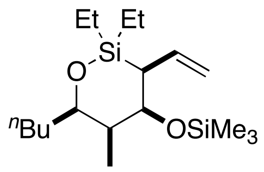

HPLC: t_R = 53.7 min; ν_{max}(film)/cm^{-1} 3583w, 3076w, 2957s, 2877s, 1628m (C=C), 1460m, 1413w, 1380m, 1295w, 1250s, 1150m, 1122m, 1076s, 1036s, 995m, 958w, 945w, 880s, 839s, 754m, 726m, 666m, 626m; δ_H (500 MHz) 0.11 (9H, s, Si(CH_3)$_3$), 0.55-0.79 (4H, stack, Si(CH_2CH$_3$)$_2$), 0.87-1.12 (12H, stack, including [0.90 (3H, t, *J* 7.5, (CH_2)$_3$CH$_3$)], [0.98 (3H, t, *J* 8.0, SiCH$_2$CH_3], CHCH_3, (CH$_2$)$_3$CH_3, Si(CH$_2$CH_3)$_2$), 1.16-1.58 (5H, stack, (CH_2)$_2$CH$_3$, CH_aH$_b$(CH$_2$)$_2$CH$_3$), 1.67-1.77 (1H, m, CH$_a$$H_b$(CH$_2$)$_2CH_3$), 1.77-1.84 (1H, m, C*H*CH$_3$), 2.02 (1H, dd, *J* 10.5, 4.8, C*H*CH=CH$_2$), 3.73 (1H, dt, *J* 10.0, 3.5, C*H*(CH$_2$)$_3$CH$_3$), 4.01 (1H, dd, *J* 4.6, 2.6, C*H*OSiMe$_3$), 4.83 (1H, d with unresolved fine coupling, *J* 16.9, CH=CH$_{cis}$$H_{trans}$), 4.87 (1H, d with unresolved fine coupling, *J* 10.5, CH=CH_{cis}H$_{trans}$), 5.91 (1H, dt, *J* 16.9, 10.5, C*H*=CH$_2$); δ_C (125 MHz) 0.5 (CH$_3$, Si(*C*H$_3$)$_3$), 4.8 (CH$_2$, Si*C*H$_2$CH$_3$), 6.1 (CH$_2$, Si*C*H$_2$CH$_3$), 6.5 (CH$_3$, SiCH$_2$*C*H$_3$), 6.8 (CH$_3$, SiCH$_2$*C*H$_3$), 11.9 (CH$_3$, CH*C*H$_3$), 14.1 (CH$_3$, (CH$_2$)$_3$*C*H$_3$), 22.7 (CH$_2$, CH_2CH$_3$), 29.2 (CH$_2$, *C*H$_2$CH$_2$CH$_3$), 33.7 (CH$_2$, *C*H$_2$(CH$_2$)$_2$CH$_3$), 39.4 (CH, *C*HCH=CH$_2$), 42.7 (CH, *C*HCH$_3$), 76.2 (CH, *C*HOSiMe$_3$), 76.6 (CH, *C*H(CH$_2$)$_3$CH$_3$), 113.5 (CH$_2$, CH=*C*H$_2$), 137.9 (CH, *C*H=CH$_2$); *m/z* (TOF ES+) 365.1 ([M+Na]$^+$, 100%); HRMS *m/z* (TOF ES+) 365.2323. $C_{18}H_{38}NaO_2Si_2$ requires 365.2308.

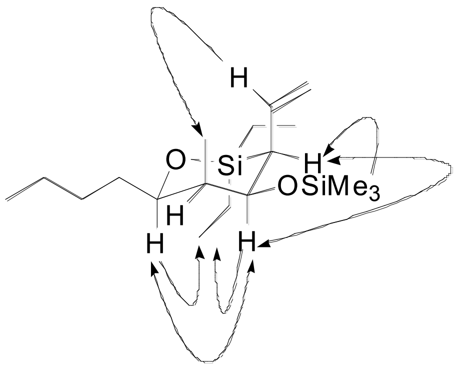

Selected nOes for oxasilacycle **18a**.

(3*R**, 4*S**, 5*R**, 6*R**)-6-Butyl-2,2-Diethyl-5-methyl-4-trimethylsilanyloxy-3-vinyl-[1,2]oxasilinane (19a)

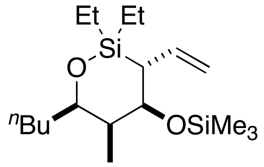

HPLC: t_R = 55.3 min; ν_{max}(film)/cm^{-1} 3608w, 3582w, 3074w, 2956s, 2933s, 2876s, 2348w, 1734w, 1626m (C=C), 1459m, 1415w, 1379w, 1322w, 1250s, 1133m, 1088s, 1064s, 1019s, 941w, 896m, 875m, 840s, 745m, 727m, 665m; δ_H (500 MHz) 0.09 (9H, s, Si(C*H*$_3$)$_3$), 0.51-0.76 (4H, stack, Si(C*H*$_2$CH$_3$)$_2$), 0.88 (3H, d, *J* 7.1, CHC*H*$_3$), 0.91 (3H, t, *J* 7.3, (CH$_2$)$_3$C*H*$_3$), 0.96 (3H, t, *J* 8.0, SiCH$_2$C*H*$_3$), 1.01 (3H, t, *J* 8.0, SiCH$_2$C*H*$_3$), 1.18-1.46 (6H, stack, (C*H*$_2$)$_3$CH$_3$), 1.68-1.75 (1H, m, C*H*CH$_3$), 2.14 (1H, app. t, *J* 10.2, C*H*CH=CH$_2$), 3.68 (1H, dd, *J* 7.3, 4.6, C*H*(CH$_2$)$_3$CH$_3$), 3.96 (1H, dd, *J* 11.5, 4.0, C*H*OSiMe$_3$), 4.89 (1H, d, *J* 16.8, CH=CH$_{cis}$*H*$_{trans}$), 4.92 (1H, d, *J* 10.2, CH=C*H*$_{cis}$H$_{trans}$), 5.57 (1H, app. dt, *J* 16.8, 10.2, C*H*=CH$_2$); δ_C (125 MHz) 0.4 (CH$_3$, Si(*C*H$_3$)$_3$), 3.4 (CH$_2$, Si*C*H$_2$CH$_3$), 3.9 (CH$_2$, Si*C*H$_2$CH$_3$), 4.5 (CH$_3$, CH*C*H$_3$), 6.6 (CH$_3$,

SiCH$_2$*C*H$_3$), 6.8 (CH$_3$, SiCH$_2$*C*H$_3$), 14.1 (CH$_3$, (CH$_2$)$_3$*C*H$_3$), 22.7 (CH$_2$, *C*H$_2$CH$_3$), 28.4 (CH$_2$, *C*H$_2$CH$_2$CH$_3$), 35.1 (CH$_2$, *C*H$_2$(CH$_2$)$_2$CH$_3$), 35.8 (CH, *C*HCH=CH$_2$), 42.9 (CH, *C*HCH$_3$), 74.3 (CH, *C*H(CH$_2$)$_3$CH$_3$), 76.5 (CH, *C*HOSiMe$_3$), 113.6 (CH$_2$, CH=*C*H$_2$), 138.1 (CH, *C*H=CH$_2$); *m/z* (TOF ES+) 365.2 ([M+Na]$^+$, 100%); HRMS *m/z* (TOF ES+) 365.2319. $C_{20}H_{34}NaO_2Si_2$ requires 365.2308.

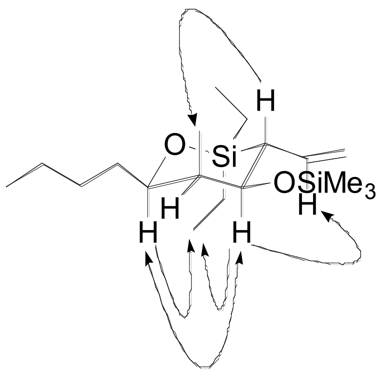

Selected nOes for oxasilacycle **19a**.

(*E*, 5*R, 6*R**)-5-Methyl-6-[(trimethylsilanyloxy)diethylsilanyloxy]-1,3-decadiene (*syn*-13a)**

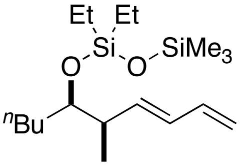

R$_f$ = 0.19 (10% toluene in hexane); ν_{max}(film)/cm^{-1} 3583w, 3087w, 2958s, 2876s, 2349w, 1797w, 1692w, 1650m (C=C), 1603 (C=C), 1552w, 1530w, 1513w, 1460m, 1414m, 1378m, 1252s, 1154m, 1065s, 1005s, 953m, 896m, 841m, 754m, 737m, 686w, 665w; δ_H (300 MHz) 0.07 (9H, s, Si(C*H*$_3$)$_3$), 0.49 (4H, q, *J* 8.1, Si(C*H*$_2$CH$_3$)$_2$),

0.81-1.01 (12H, stack, $CH_3(CH_2)_3$, $CHCH_3$, $Si(CH_2CH_3)_2$), 1.11-1.41 (6H, stack, $(CH_2)_3CH_3$), 2.31 (1H, app. sextet, *J* 6.2, $CHCH_3$), 3.56-3.66 (1H, m, *CH*OSi), 4.93 (1H, d, *J* 10.3, $CH{=}CH_{cis}H_{trans}$), 5.06 (1H, d, *J* 16.9, $CH{=}CH_{cis}H_{trans}$), 5.70 (1H, dd, *J* 15.4, 7.7, $CH(CH_3)CH{=}CH$), 5.94-6.07 (1H, m, $CH{=}CH{=}CH_2$), 6.29 (1H, app. dt, *J* 16.9, 10.3, $CH{=}CH_2$); δ_C (100 MHz) 1.9 (CH_3, $Si(CH_3)_3$), 6.3 (CH_2, $SiCH_2CH_3$), 6.4 (CH_2, $SiCH_2CH_3$), 6.7 (CH_3, $Si(CH_2CH_3)_2$), 14.1 (CH_3), 15.3 (CH_3), 22.9 (CH_2), 27.8 (CH_2), 33.8 (CH_2), 42.2 (CH, $CHCH_3$), 75.8 (CH, *C*HOSi), 114.8 (CH_2, $CH{=}CH_2$), 130.3 (CH, =CH), 138.6 (CH, =CH), 138.1 (CH, =CH); *m/z* (TOF ES+) 365.2 ($[M+Na]^+$, 100%); HRMS *m/z* (TOF ES+) 365.2304. $C_{18}H_{38}NaO_2Si_2$ requires 365.2308.

Allylation reaction of Aldehyde *anti*-4a

TMSOTf (100 µL, 0.66 mmol) was added to a solution of aldehyde ***anti*-4a** (175 mg, 0.51 mmol) and TTBP (168 mg, 0.68 mmol) in CH_2Cl_2 (7 mL) at –78 ^oC and the reaction was stirred for 6 h. The reaction was quenched by the addition of $NaHCO_3$ solution (7 mL) at –78 ^oC and then the reaction mixture was allowed to warm to room temperature over 30 min. The two phases were separated and the aqueous phase was extracted with CH_2Cl_2 (2 x 7 mL). The combined organic extracts were washed with water (7 mL) and brine (7 mL) and dried over $MgSO_4$. Filtration and evaporation under reduced pressure afforded a colourless oil. TTBP was removed by flash column chromatography (hexane) to afford diene ***anti*-13a** and 2 diastereoisomeric oxasilacycles **14a** and **15a** (**14a**:**15a**:***anti*-13a**, 4:1:3) (159 mg, 91%). Flash column chromatography (10% $PhCH_3$ in hexane) afforded pure diene ***anti*-13a.** The oxasilacycles were inseparable by flash column chromatography. Analytically pure samples of each diastereoisomer were obtained by preparative HPLC.

(3*S, 4*R**, 5*S**, 6*R**)-6-Butyl-2,2-diethyl-5-methyl-4-trimethylsilanyloxy-3-vinyl-[1,2]oxasilinane (14a)**

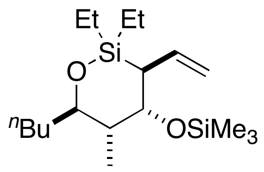

HPLC: t_R = 47.9 min; ν_{max}(film)/cm^{-1} 3608w, 3582w, 3074w, 2957s, 2935s, 2876s, 1780w, 1734w, 1625m (C=C), 1458m, 1415w, 1380m, 1362w, 1342w, 1251s, 1175w, 1131m, 1070s, 1005s, 988s, 945m, 896m, 880s, 841s, 811m, 741s, 726s, 665m, 627m; δ_H (500 MHz) 0.11 (9H, s, Si(C*H*$_3$)$_3$), 0.57 (2H, q, *J* 7.8, SiC*H*$_2$CH$_3$), 0.71-0.80 (2H, m, SiC*H*$_2$CH$_3$), 0.88-0.96 (9H, stack, SiCH$_2$C*H*$_3$, (CH$_2$)$_3$C*H*$_3$, CHC*H*$_3$), 0.99 (3H, t, *J* 8.0, SiCH$_2$C*H*$_3$), 1.24-1.39 (3H, stack, C*H*$_a$H$_b$C*H*$_2$CH$_3$), 1.26-1.51 (3H, stack, C*H*$_2$CH$_a$*H*$_b$CH$_2$CH$_3$), 1.69-1.76 (1H, m, C*H*CH$_3$), 2.01 (1H, dd, *J* 10.3, 7.6, C*H*CH=CH$_2$), 3.80 (1H, app q, *J* 5.8, C*H*(CH$_2$)$_3$CH$_3$), 3.98 (1H, dd, *J* 7.6, 2.6, C*H*OSiMe$_3$), 4.90 (1H, d, *J* 10.3, CH=C*H*$_{cis}$H$_{trans}$), 4.91 (1H, d, *J* 16.9, CH=CH$_{cis}$*H*$_{trans}$), 5.73 (1H, app. dt, *J* 16.9, 10.3, C*H*=CH$_2$); δ_C (125 MHz) 0.3 (CH$_3$, Si(*C*H$_3$)$_3$), 5.4 (CH$_2$, Si*C*H$_2$CH$_3$), 5.6 (CH$_2$, SiC*H*$_2$CH$_3$), 6.4 (CH$_3$, SiCH$_2$*C*H$_3$), 6.6 (CH$_3$, SiCH$_2$*C*H$_3$), 14.1 (CH$_3$, (CH$_2$)$_3$*C*H$_3$), 14.3 (CH$_3$, CH*C*H$_3$), 22.7 (CH$_2$, *C*H$_2$CH$_3$), 28.0 (CH$_2$, C*H*$_2$CH$_2$CH$_3$), 35.6 (CH$_2$, *C*H$_2$(CH$_2$)$_2$CH$_3$), 37.7 (CH, *C*HCH=CH$_2$), 40.5 (CH, *C*HCH$_3$), 75.0 (CH, *C*HOSiMe$_3$), 75.3 (CH, *C*H(CH$_2$)$_3$CH$_3$), 113.5 (CH$_2$, CH=*C*H$_2$), 137.8 (CH, *C*H=CH$_2$); *m/z* (TOF ES+) 413.4 ([M+K+MeOH]$^+$, 15%), 365.3 (100, [M+Na]$^+$); HRMS *m/z* (TOF ES+) 365.2314. $C_{18}H_{38}NaO_2Si_2$ requires 365.2308.

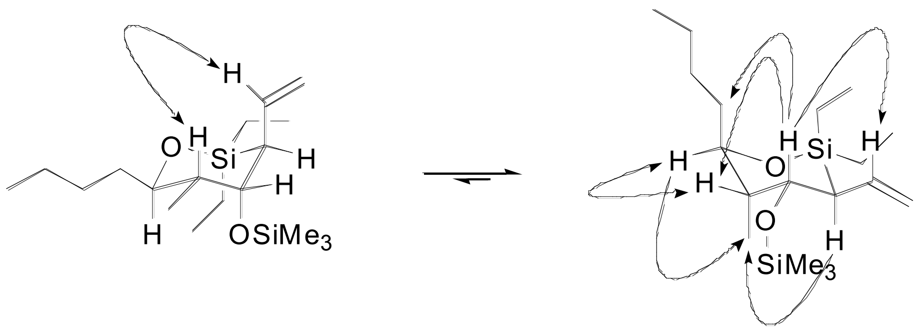

Selected nOes for oxasilacycle **14a** suggest a conformationally flexible structure.

(3*R**, 4*R**, 5*S**, 6*R**)-6-Butyl-2,2-diethyl-5-methyl-4-trimethylsilanyloxy-3-vinyl-[1,2]oxasilinane (15a)

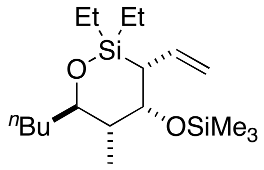

HPLC: t_R = 48.7 min; ν_{max}(film)/cm^{-1} 3583w, 3075w, 2957s, 2935s, 2876s, 1626m (C=C), 1459m, 1416w, 1380m, 1342w, 1252s, 1175w, 1131m, 1070s, 1005s, 988s, 945m, 896m, 880s, 841s, 811m, 741m, 726m, 666m, 627m; δ_H (500 MHz) 0.13 (9H, s, Si(CH_3)$_3$), 0.47-0.56 (1H, m, SiCH_aH$_b$CH$_3$), 0.57-0.67 (2H, stack, SiCH_2CH$_3$), 0.77-0.85 (4H, stack, including [0.82 (3H, d, *J* 6.9, CHCH_3)], SiCH$_a$$H_bCH_3$, CHC$H_3$), 0.90 (3H, t, *J* 7.1, (CH$_2$)$_3$CH_3), 0.95 (3H, t, *J* 7.9, SiCH$_2$CH_3), 0.96 (3H, t, *J* 7.9, SiCH$_2$CH_3), 1.12-1.54 (7H, stack, (CH_2)$_3$CH$_3$, C*H*CH$_3$), 1.91 (1H, dd, *J* 10.0, 2.2, C*H*CH=CH$_2$), 3.79-3.85 (1H, m, C*H*(CH$_2$)$_3$CH$_3$), 3.94 (1H, br s, C*H*OSiMe$_3$), 4.86 (1H, d with unresolved fine coupling, *J* 10.0, CH=CH_{cis}H$_{trans}$), 4.89 (1H, d with unresolved fine coupling, *J* 16.3, CH=CH$_{cis}$$H_{trans}$), 5.87 (1H, app. dt, *J* 16.3, 10.0, C*H*=CH$_2$); δ_C (125 MHz) 0.9 (CH$_3$, Si(*C*H$_3$)$_3$), 4.8 (CH$_2$, Si*C*H$_2$CH$_3$), 5.7 (CH$_2$,

SiCH_2CH_3), 6.6 (CH_3, SiCH_2*C*H_3), 6.7 (CH_3, SiCH_2*C*H_3), 14.2 (CH_3, $(CH_2)_3$*C*H_3), 16.7 (CH_3, CH*C*H_3), 22.9 (CH_2, *C*H_2CH_3), 26.9 (CH_2, C*H*$_2CH_2CH_3$), 34.7 (CH_2, *C*$H_2(CH_2)_2CH_3$), 40.6 (CH, *C*HCH=CH_2), 43.2 (CH, *C*HCH_3), 71.7 (CH, *C*H$(CH_2)_3CH_3$), 79.6 (CH, *C*HOSiMe_3), 112.8 (CH_2, CH=*C*H_2), 138.4 (CH, *C*H=CH_2); *m/z* (TOF ES+) 413.4 ($[M+K+MeOH]^+$, 25%), 365.4 (100, $[M+Na]^+$); HRMS *m/z* (TOF ES+) 365.2291. $C_{18}H_{38}NaO_2Si_2$ requires 365.2308.

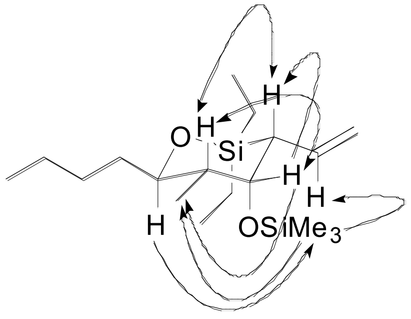

Selected nOes for oxasilacycle **15a**.

(*E*, 5*S**, 6*R**)-5-Methyl-6-[(trimethylsilanyloxy)diethylsilanyloxy]-1,3-decadiene (*anti*-13a)

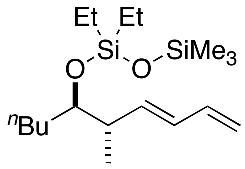

R_f = 0.18 (10% toluene in hexane); ν_{max}(film)/cm^{-1} 3848w, 3582w, 3086w, 2957s, 2876s, 2348w, 1797w, 1692w, 1650w (C=C), 1603m (C=C), 1552w, 1530w, 1460m, 1414m, 1378m, 1252s, 1067s, 1005s, 925w, 896m, 840s, 754m, 737m, 686w, 665w; δ_H (300 MHz) 0.10 (9H, s, Si(C*H*$_3$)$_3$), 0.52 (4H, q, *J* 8.5, Si(C*H*$_2CH_3$)$_2$), 0.88 (3H, t, *J*

6.6, $(CH_2)_3CH_3$), 0.94 (6H, t, *J* 7.7, $Si(CH_2CH_3)_2$), 1.02 (3H, d, *J* 6.7, $CHCH_3$), 1.14-1.42 (6H, stack, $(CH_2)_3CH_3$), 2.27-2.41 (1H, m, $CHCH_3$), 3.63-3.71 (1H, m, *CH*OSi), 4.97 (1H, dd, *J* 10.2, 1.1, $CH{=}CH_{cis}H_{trans}$), 5.10 (1H, d with unresolved fine coupling, *J* 16.9, $CH{=}CH_{cis}H_{trans}$), 5.68 (1H, dd, *J* 15.4, 8.1, $CH(CH_3)CH{=}CH$), 6.03 (1H, dd, *J* 15.4, 10.2, $CHCH{=}CH_2$), 6.33 (1H, app. dt, *J* 16.9, 10.2, $CH{=}CH_2$); δ_C (75 MHz) 1.9 (CH_3, $Si(CH_3)_3$), 6.3 (CH_2, $SiCH_2CH_3$), 6.5 (CH_2, $SiCH_2CH_3$), 6.71 (CH_3, $SiCH_2CH_3$), 6.72 (CH_3, $SiCH_2CH_3$), 14.1 (CH_3), 15.9 (CH_3), 22.8 (CH_2, CH_3CH_2), 28.1 (CH_2, $CH_3CH_2CH_2$), 33.6 (CH_2, $CH_3(CH_2)_2CH_2$), 42.3 (CH, $CHCH_3$), 75.7 (CH, *C*HOSi), 114.8 (CH_2, $CH{=}CH_2$), 130.7 (CH, $CH(CH_3)CH{=}CH$), 137.5 (CH, =*C*H), 137.6 (CH, =*C*H); *m/z* (TOF ES+) 365.4 ($[M+Na]^+$, 100%); HRMS *m/z* (TOF ES+) 365.2301. $C_{18}H_{38}NaO_2Si_2$ requires 365.2308.

References

1. Crich D, Smith M, Yao Q, Picione J: *Synthesis*, 2001, 323-326.
2. Ireland RE, Liu L: *J Org Chem* 1993, **58:**2899.
3. Dess DB, Martin JC: *J Am Chem Soc* 1991, **113:**7277-7287.
4. Heathcock CH, Pirrung MC, Montgomery SH, Lampe J: *Tetrahedron* 1981, **23:**4087-4095.
