# Supplementary material for: Tether-directed synthesis of highly substituted oxasilacycles via an intramolecular allylation employing allylsilanes
Source: Beilstein J Org Chem. 2007 Feb 8;3:6. doi: 10.1186/1860-5397-3-6 (PMC1810303; doi:10.1186/1860-5397-3-6)
Supplement: File 2 — 1H-NMR and 13C-NMR Spectra for the following compounds: 5b, 6b, 7b, syn-8b, anti-8b, syn-11b, anti-11b, syn-4b, anti-4b, syn-9a, anti-9a, syn-12a, anti-12a, syn-4a, anti-4a, syn-13b, anti-13b, syn-13a, anti-13a. [file Beilstein_J_Org_Chem-03-06-s002.pdf]

Additional File 2

Tether-directed synthesis of highly substituted oxasilacycles *via* an intramolecular allylation employing allylsilanes

Peter J. Jervis and Liam R. Cox*

email: l.r.cox@bham.ac.uk

^{1}H-NMR and ^{13}C-NMR Spectra for the following compounds:

5b, 6b, 7b, *syn*-8b, *anti*-8b, *syn*-11b, *anti*-11b, *syn*-4b, *anti*-4b, *syn*-9a, *anti*-9a,

***syn*-12a, *anti*-12a,*syn*-4a, *anti*-4a, *syn*-13b, *anti*-13b, *syn*-13a, *anti*-13a**

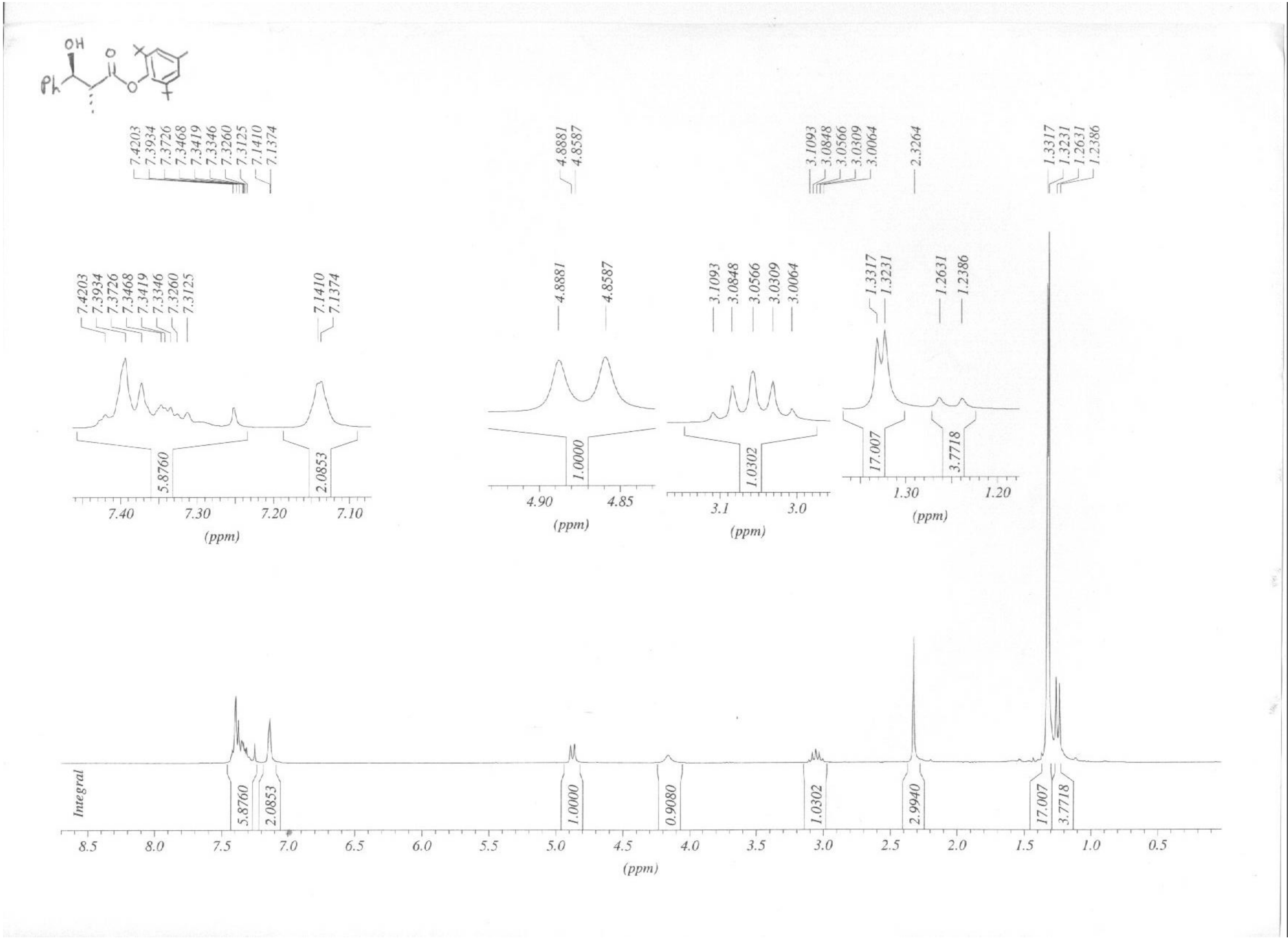
OH
Ph
7.4203
7.3934
7.3726
7.3468
7.3419
7.3346
7.3260
7.3125
7.1410
7.1374
4.8881
4.8587
3.1093
3.0848
3.0566
3.0309
3.0064
2.3264
1.3317
1.3231
1.2631
1.2386
5.8760
2.0853
1.0000
1.0302
17.007
3.7718
0.9080
2.9940
Integral
(ppm)

5b

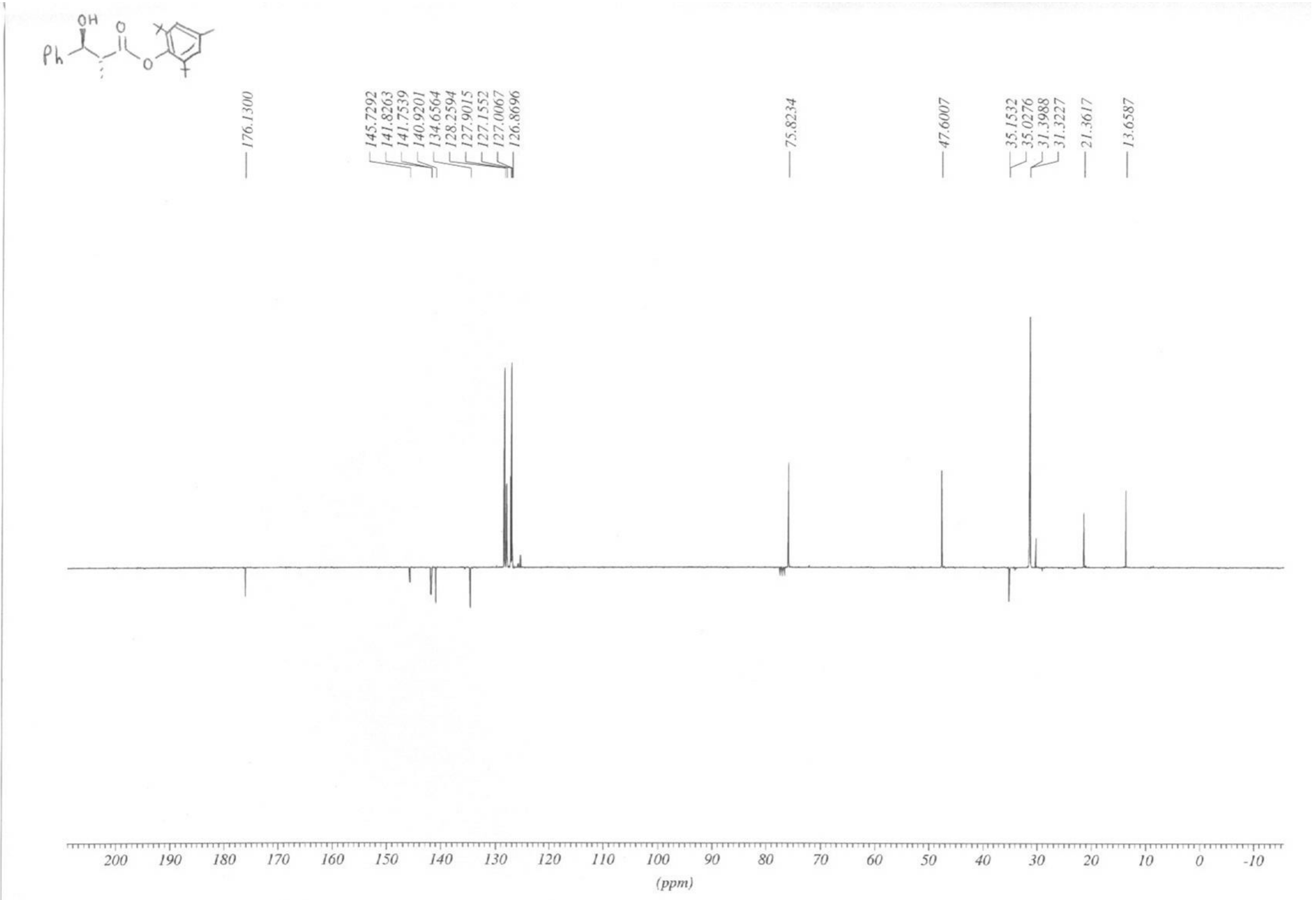

Ph
OH
176.1300
145.7292
141.8263
141.7539
140.9201
134.6564
128.2594
127.9015
127.1552
127.0067
126.8696
75.8234
47.6007
35.1532
35.0276
31.3988
31.3227
21.3617
13.6587
200 190 180 170 160 150 140 130 120 110 100 90 80 70 60 50 40 30 20 10 0 -10
(ppm)

5b

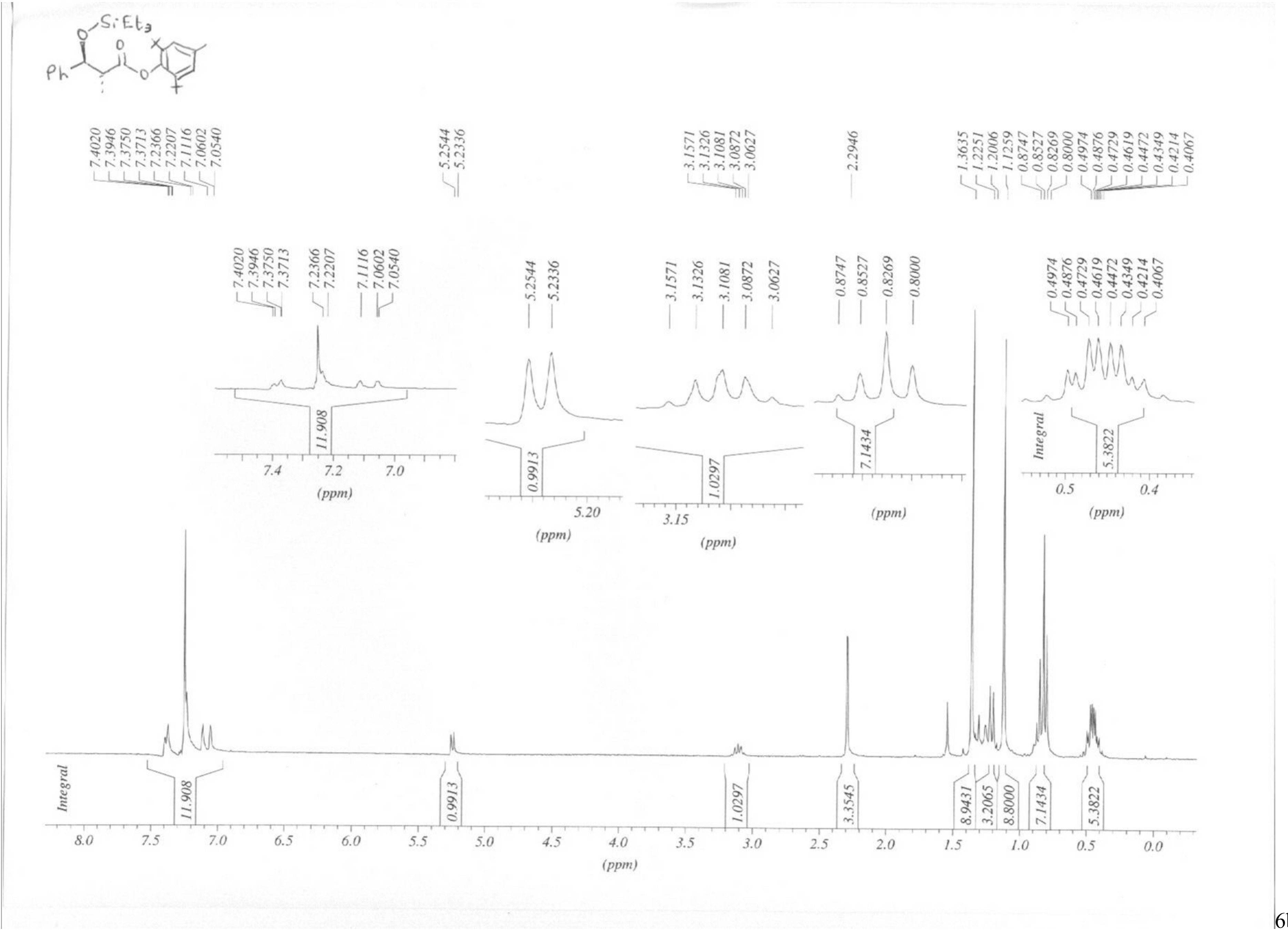

SiEt3
Ph
7.4020 7.3946 7.3750 7.3713 7.2366 7.2207 7.1116 7.0602 7.0540
5.2544 5.2336
3.1571 3.1326 3.1081 3.0872 3.0627
2.2946
1.3635 1.2251 1.2006 1.1259 0.8747 0.8527 0.8269 0.8000 0.4974 0.4876 0.4729 0.4619 0.4472 0.4349 0.4214 0.4067
11.908
0.9913
1.0297
7.1434
5.3822
Integral
(ppm)
Integral
11.908 0.9913 1.0297 3.3545 8.9431 3.2065 8.8000 7.1434 5.3822
8.0 7.5 7.0 6.5 6.0 5.5 5.0 4.5 4.0 3.5 3.0 2.5 2.0 1.5 1.0 0.5 0.0
(ppm)

6b

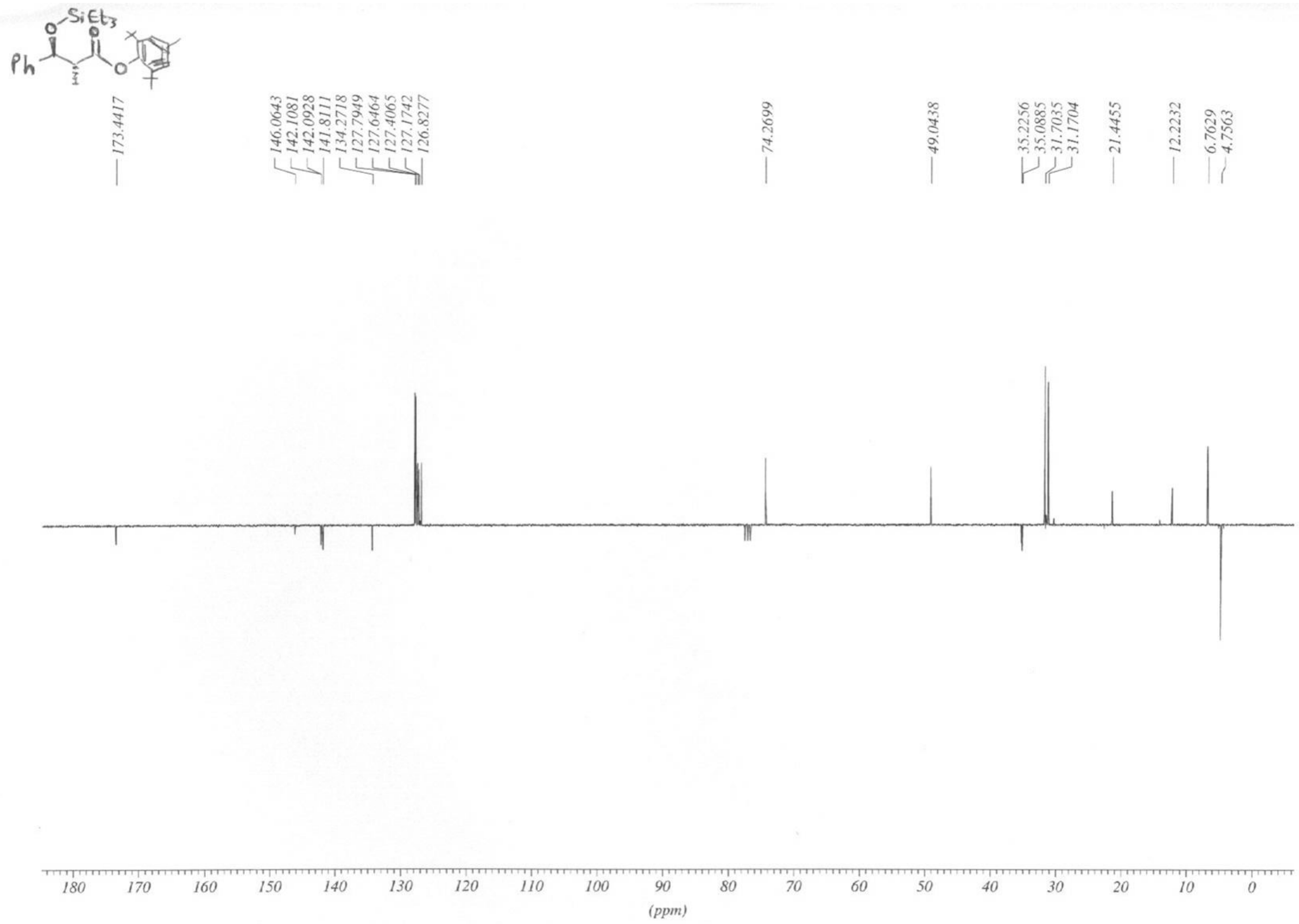

O-SiEt3
Ph
173.4417
146.0643
142.1081
142.0928
141.8111
134.2718
127.7949
127.6464
127.4065
127.1742
126.8277
74.2699
49.0438
35.2256
35.0885
31.7035
31.1704
21.4455
12.2232
6.7629
4.7563
180 170 160 150 140 130 120 110 100 90 80 70 60 50 40 30 20 10 0
(ppm)

6b

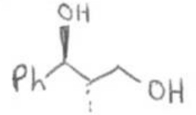
OH
Ph
OH

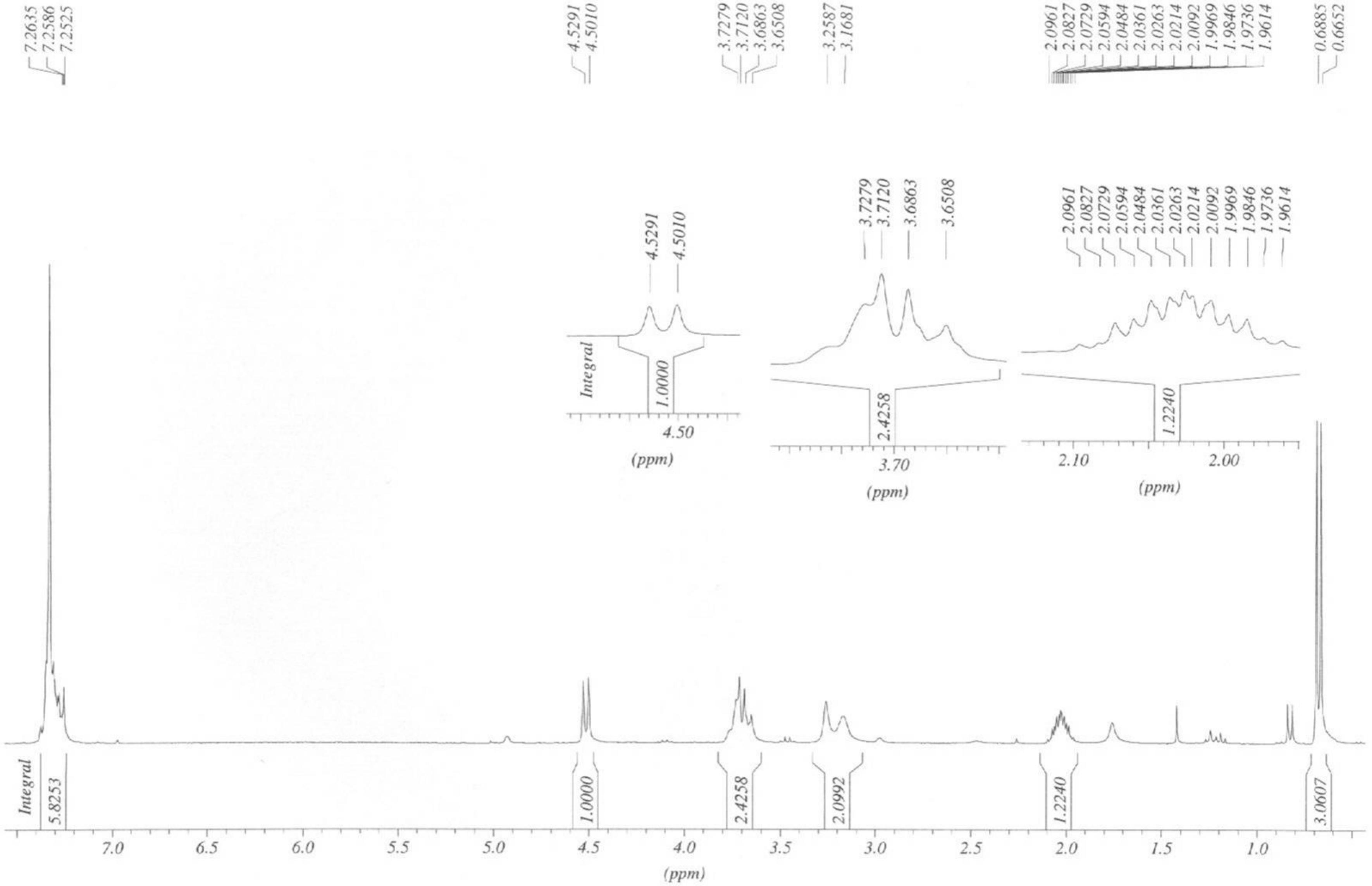
7.2635
7.2586
7.2525
4.5291
4.5010
3.7279
3.7120
3.6863
3.6508
3.2587
3.1681
2.0961
2.0827
2.0729
2.0594
2.0484
2.0361
2.0263
2.0214
2.0092
1.9969
1.9846
1.9736
1.9614
0.6885
0.6652
Integral
1.0000
4.50
(ppm)
2.4258
3.70
(ppm)
1.2240
2.10
2.00
(ppm)
Integral
5.8253
1.0000
2.4258
2.0992
1.2240
3.0607
7.0
6.5
6.0
5.5
5.0
4.5
4.0
3.5
3.0
2.5
2.0
1.5
1.0
(ppm)

7b

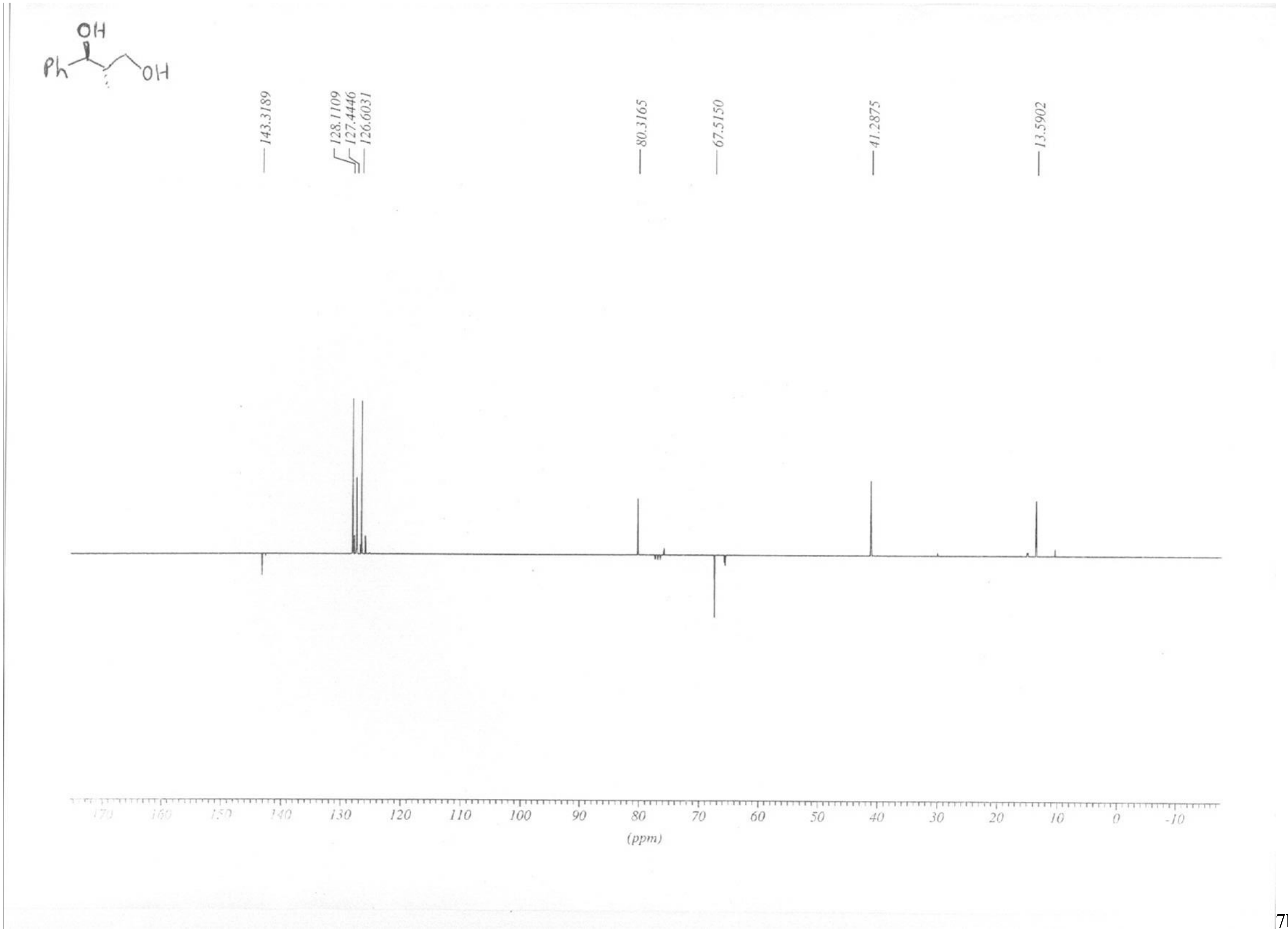

OH
Ph
OH
143.3189
128.1109
127.4446
126.6031
80.3165
67.5150
41.2875
13.5902
170
160
150
140
130
120
110
100
90
80
70
60
50
40
30
20
10
0
-10
(ppm)

7b

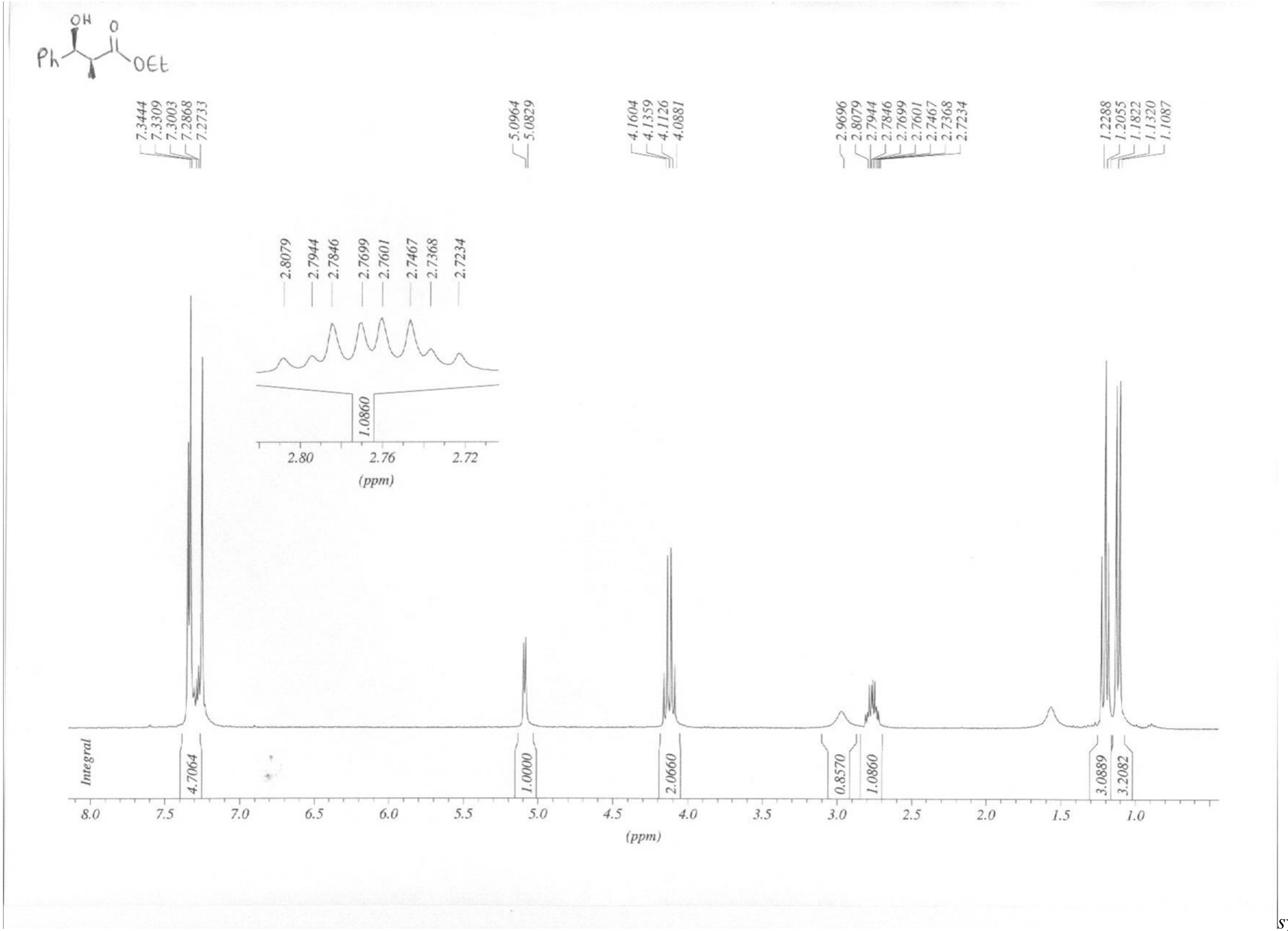
OH
O
Ph
OEt
7.3444
7.3309
7.3003
7.2868
7.2733
5.0964
5.0829
4.1604
4.1359
4.1126
4.0881
2.9696
2.8079
2.7944
2.7846
2.7699
2.7601
2.7467
2.7368
2.7234
1.2288
1.2055
1.1822
1.1320
1.1087
1.0860
2.80
2.76
2.72
(ppm)
Integral
4.7064
1.0000
2.0660
0.8570
1.0860
3.0889
3.2082
8.0
7.5
7.0
6.5
6.0
5.5
5.0
4.5
4.0
3.5
3.0
2.5
2.0
1.5
1.0
(ppm)

syn-8b

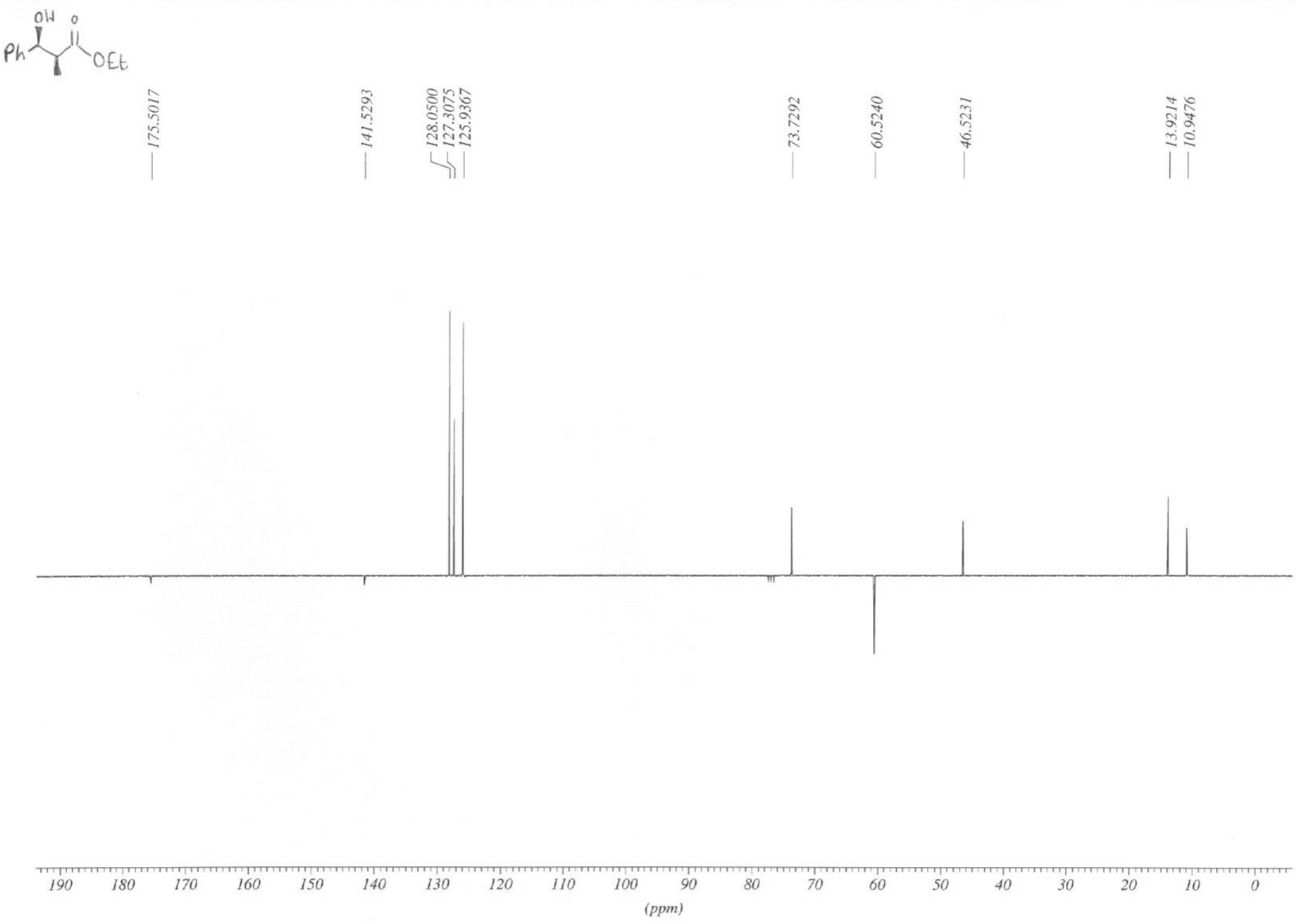
175.5017
141.5293
128.0500
127.3075
125.9367
73.7292
60.5240
46.5231
13.9214
10.9476
190 180 170 160 150 140 130 120 110 100 90 80 70 60 50 40 30 20 10 0
(ppm)

syn-8b

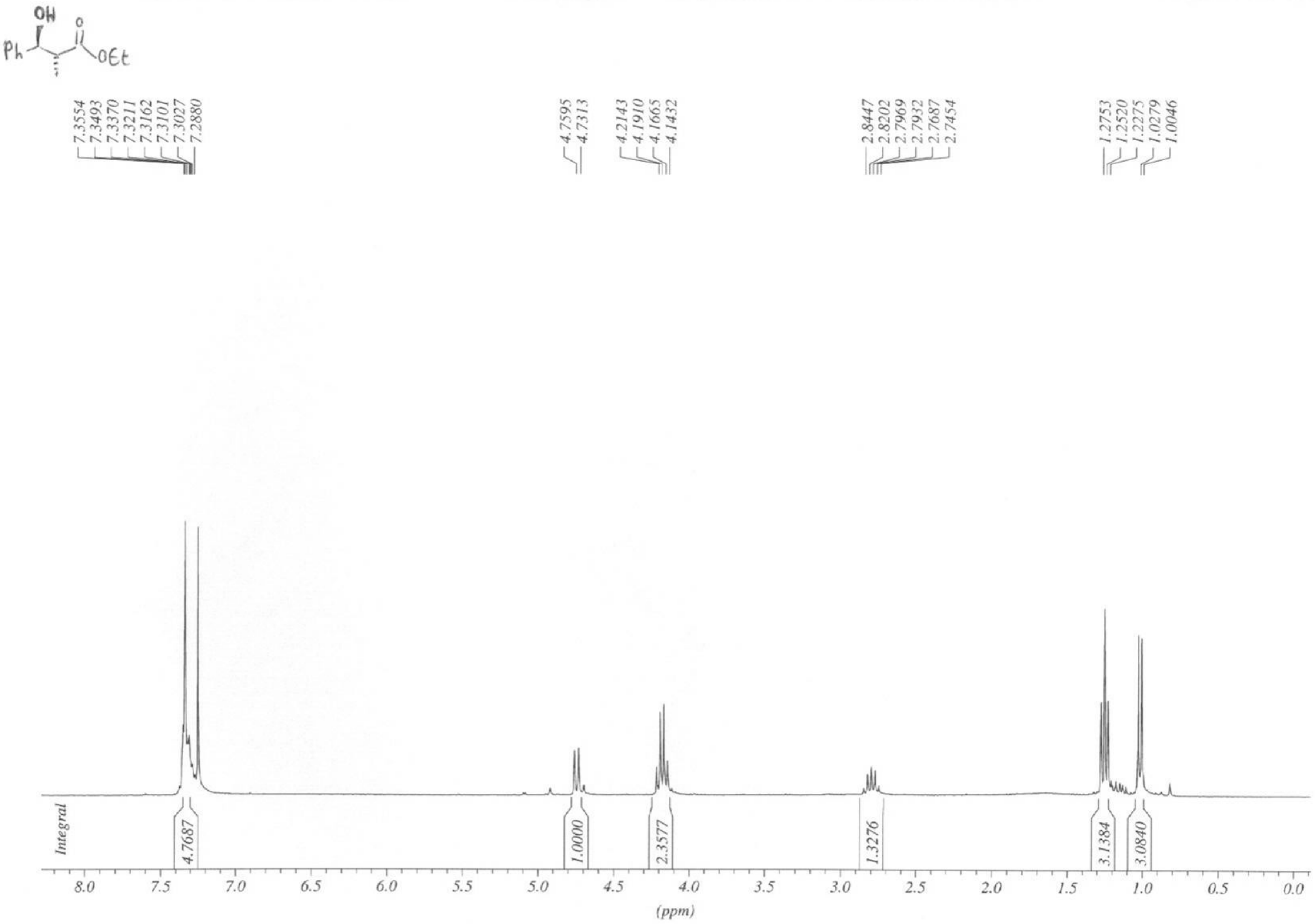
OH
O
Ph
OEt
7.3554
7.3493
7.3370
7.3211
7.3162
7.3101
7.3027
7.2880
4.7595
4.7313
4.2143
4.1910
4.1665
4.1432
2.8447
2.8202
2.7969
2.7932
2.7687
2.7454
1.2753
1.2520
1.2275
1.0279
1.0046
Integral
4.7687
1.0000
2.3577
1.3276
3.1384
3.0840
8.0
7.5
7.0
6.5
6.0
5.5
5.0
4.5
4.0
3.5
3.0
2.5
2.0
1.5
1.0
0.5
0.0
(ppm)

anti-8b

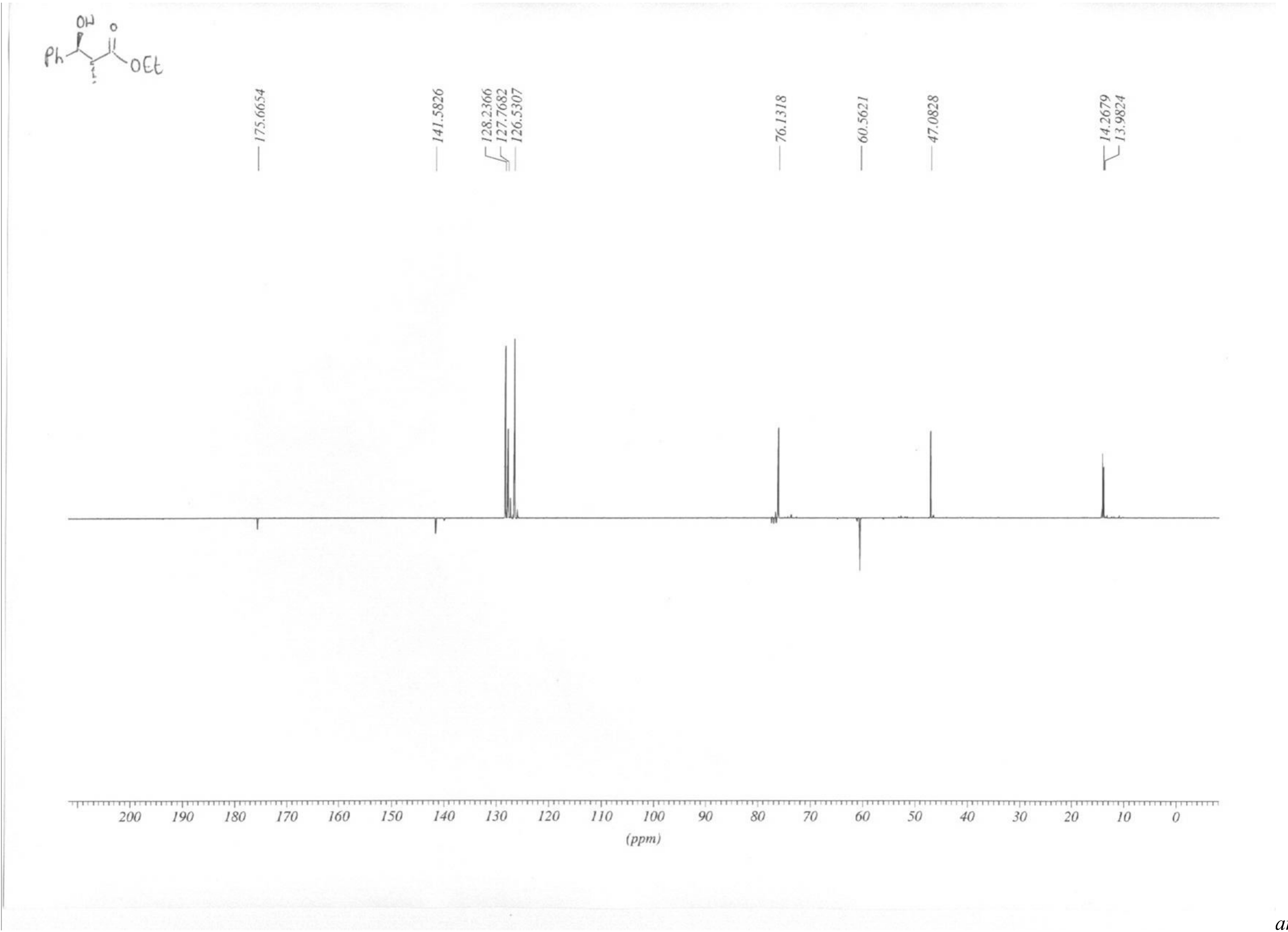
OH
O
Ph
OEt
175.6654
141.5826
128.2366
127.7682
126.5307
76.1318
60.5621
47.0828
14.2679
13.9824
200 190 180 170 160 150 140 130 120 110 100 90 80 70 60 50 40 30 20 10 0
(ppm)

anti-8b

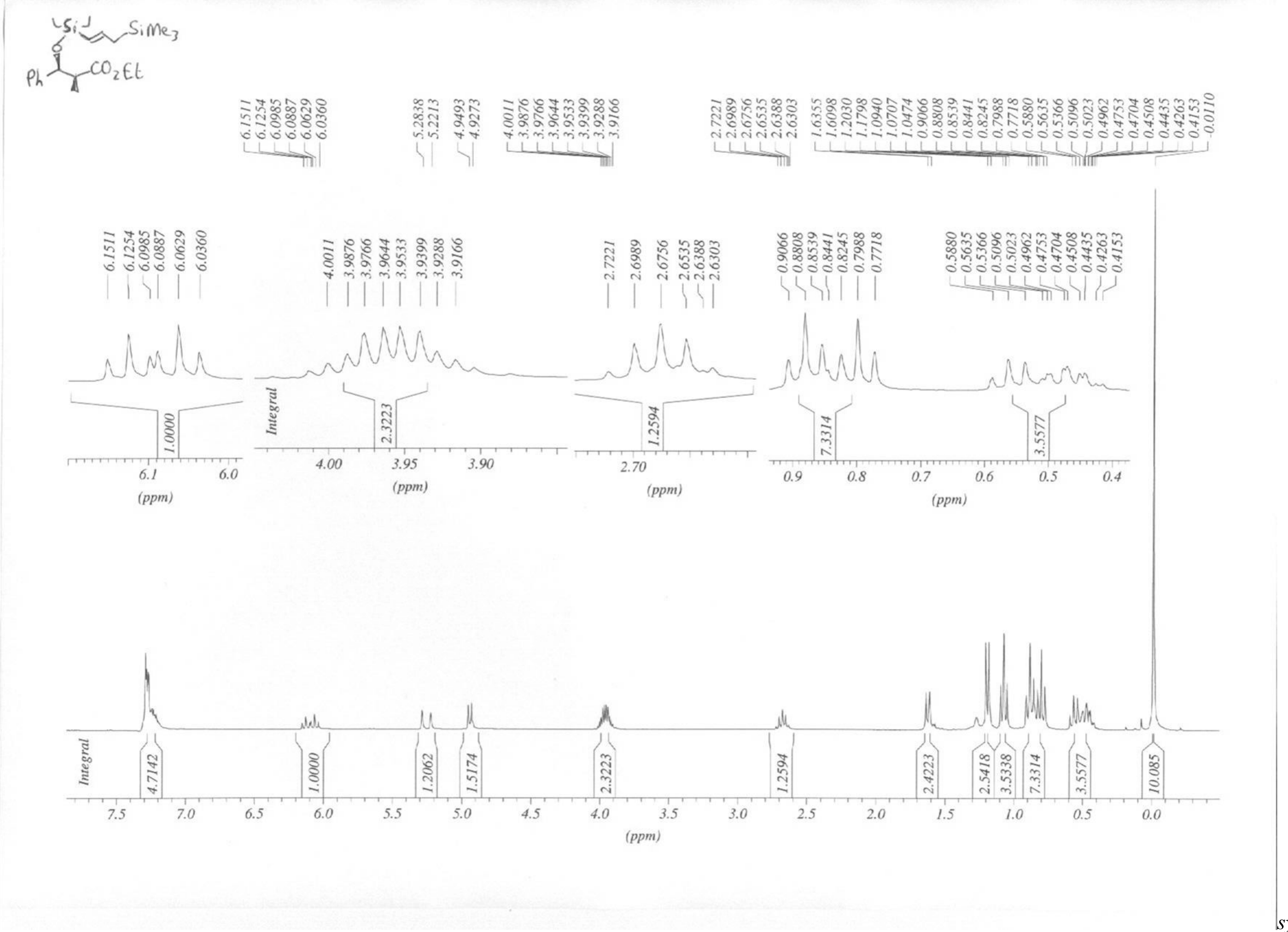

syn-11b

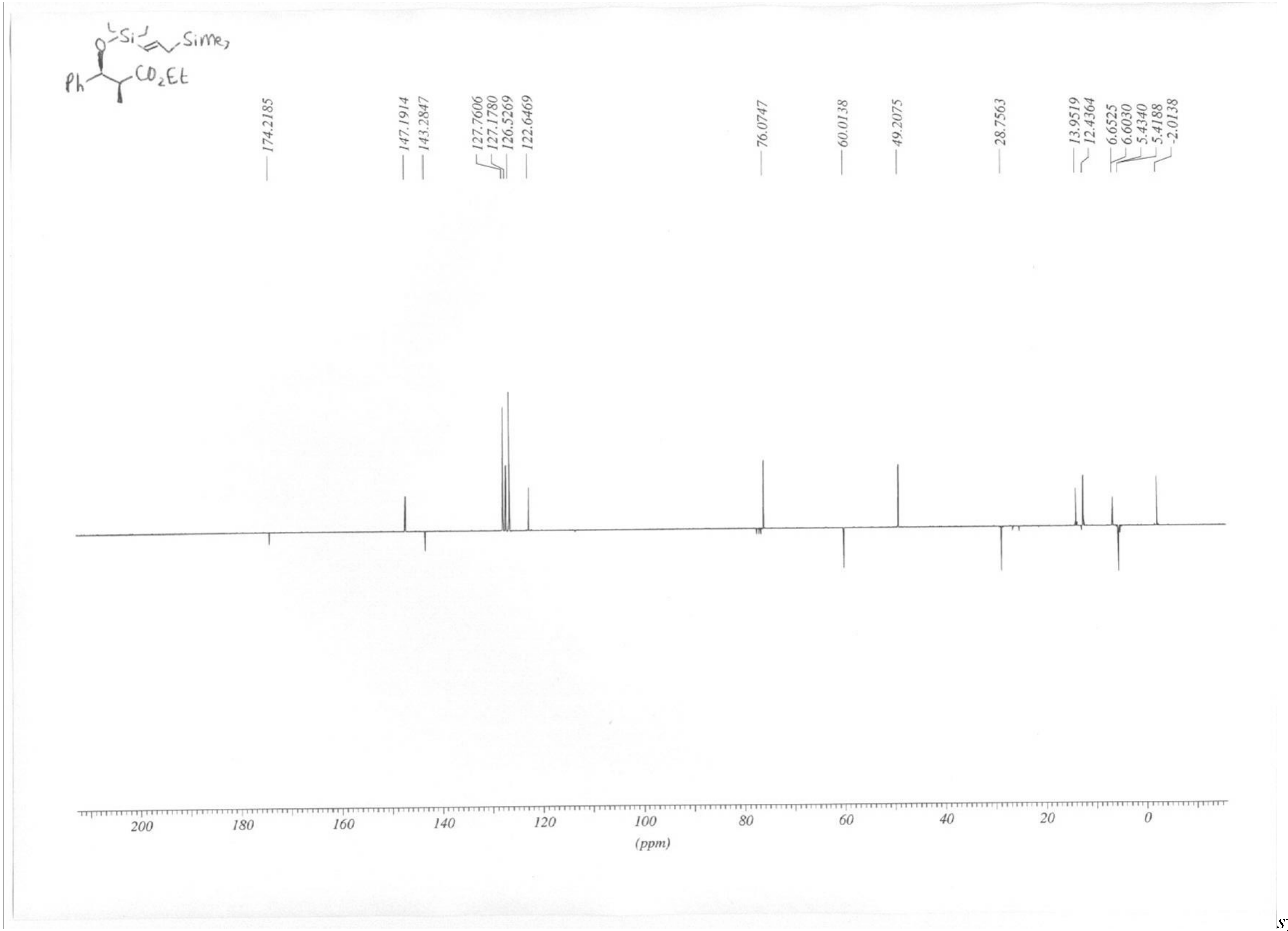
Si
SiMe3
Ph
CO2Et
174.2185
147.1914
143.2847
127.7606
127.1780
126.5269
122.6469
76.0747
60.0138
49.2075
28.7563
13.9519
12.4364
6.6525
6.6030
5.4340
5.4188
-2.0138
200
180
160
140
120
100
80
60
40
20
0
(ppm)

syn-11b

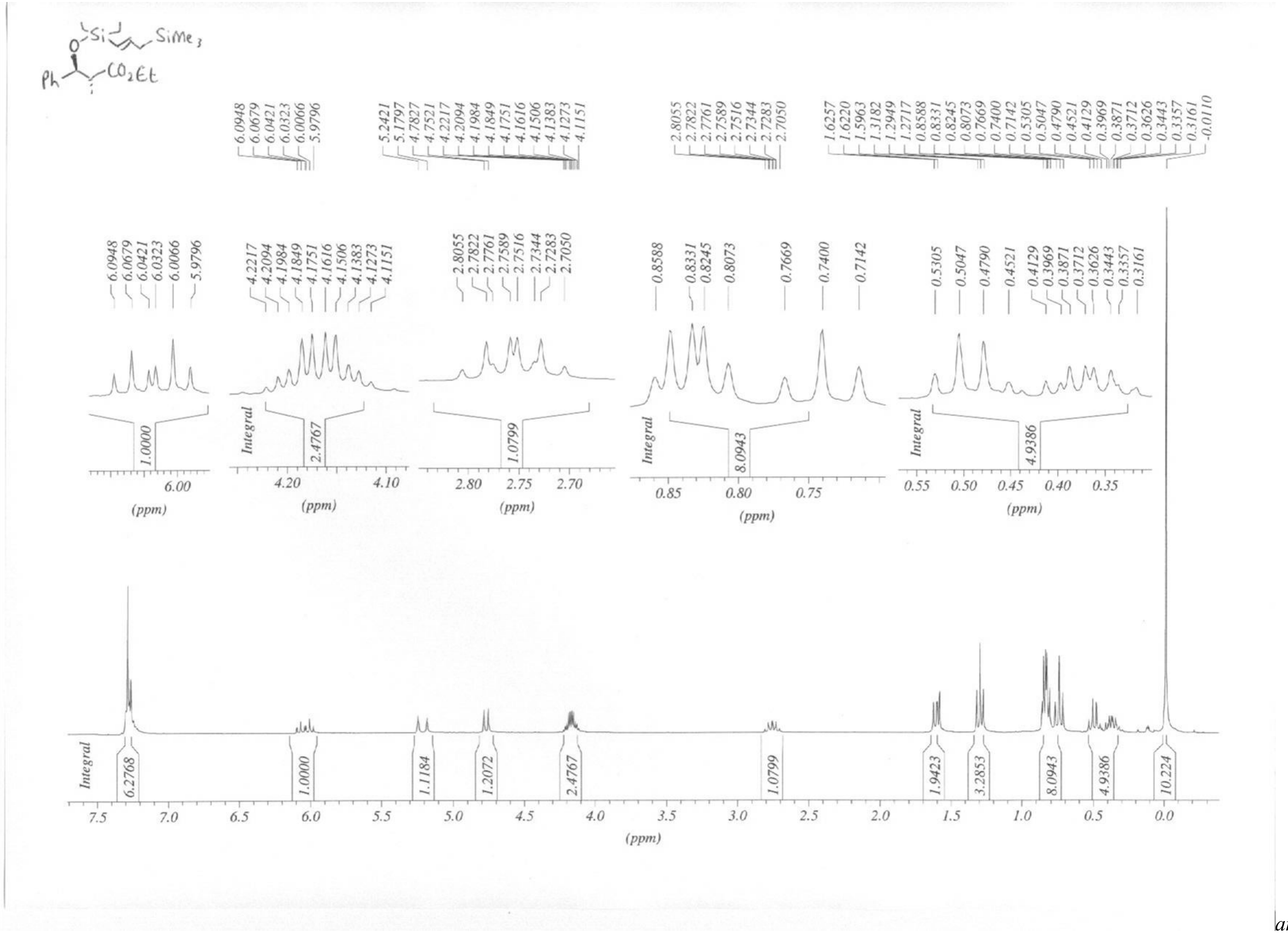

SiMe3
Ph
CO2Et
6.0948 6.0679 6.0421 6.0323 6.0066 5.9796
5.2421 5.1797 4.7827 4.7521 4.2217 4.2094 4.1984 4.1849 4.1751 4.1616 4.1506 4.1383 4.1273 4.1151
2.8055 2.7822 2.7761 2.7589 2.7516 2.7344 2.7283 2.7050
1.6257 1.6220 1.5963 1.3182 1.2949 1.2717 0.8588 0.8331 0.8245 0.8073 0.7669 0.7400 0.7142 0.5305 0.5047 0.4790 0.4521 0.4129 0.3969 0.3871 0.3712 0.3626 0.3443 0.3357 0.3161 -0.0110
Integral
1.0000
2.4767
1.0799
8.0943
4.9386
(ppm)
6.2768
1.1184
1.2072
1.9423
3.2853
10.224

anti-11b

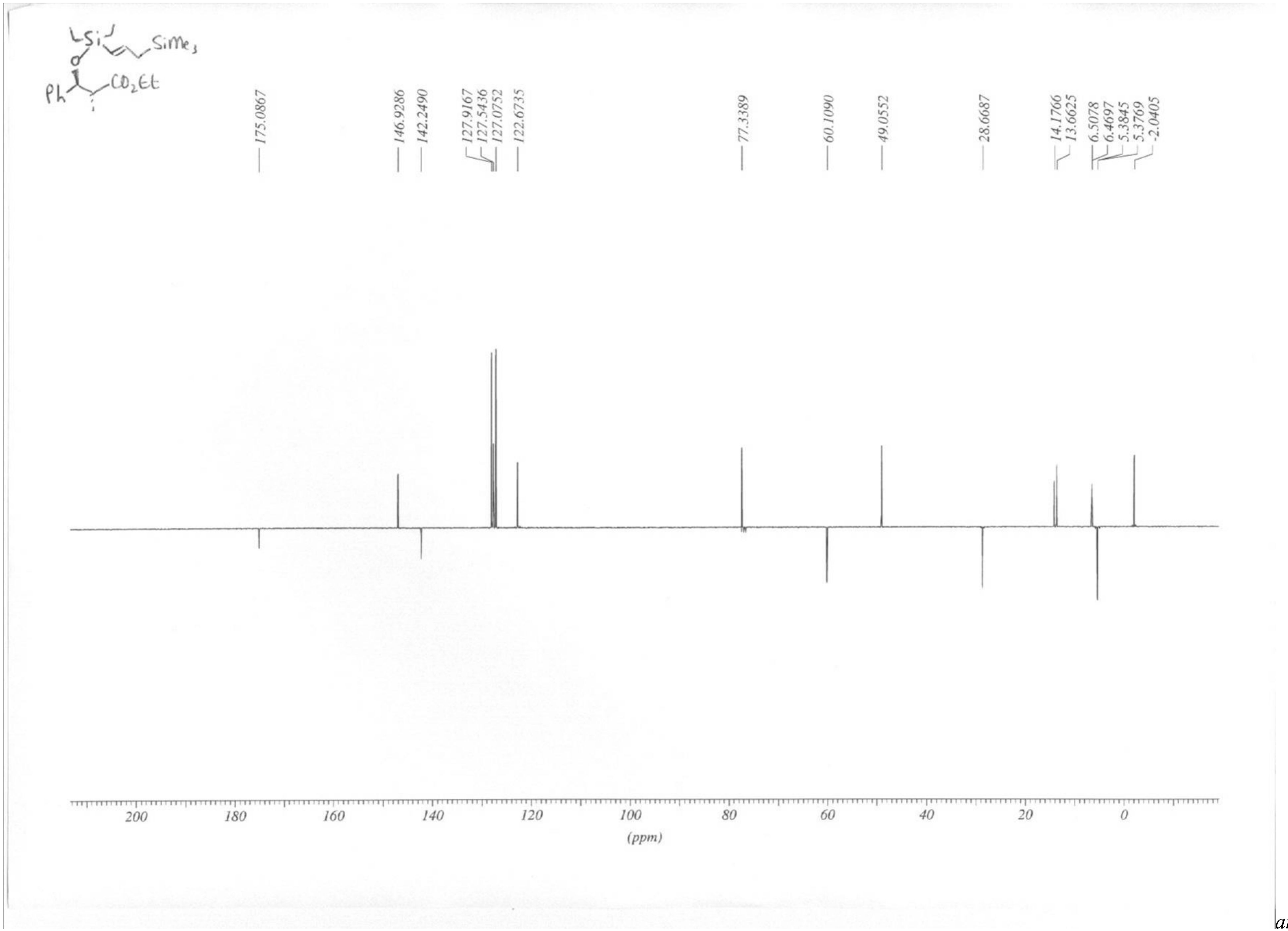
SiMe3
Ph
CO2Et
175.0867
146.9286
142.2490
127.9167
127.5436
127.0752
122.6735
77.3389
60.1090
49.0552
28.6687
14.1766
13.6625
6.5078
6.4697
5.3845
5.3769
-2.0405
200
180
160
140
120
100
80
60
40
20
0
(ppm)

anti-11b

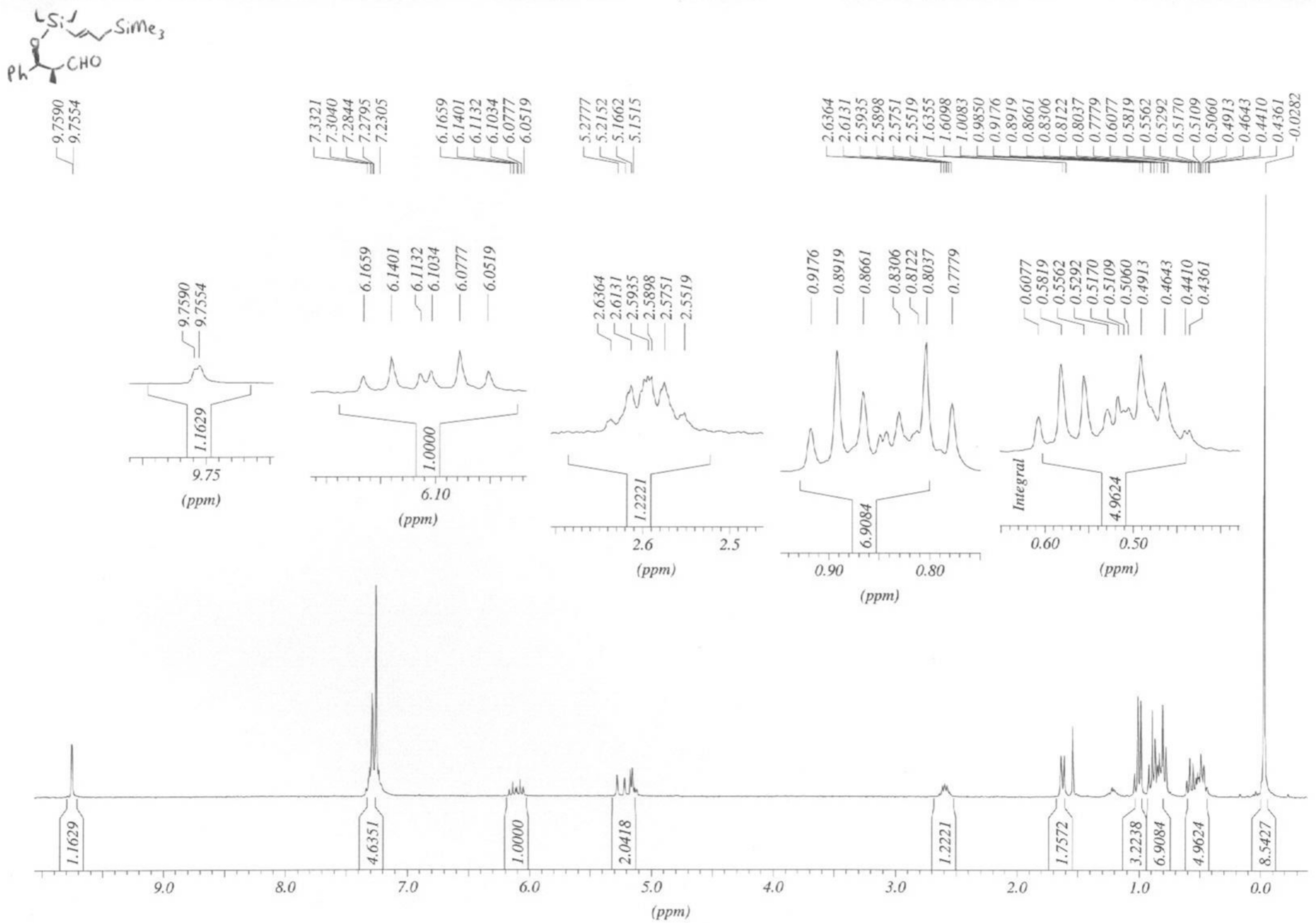
SiMe3
CHO
Ph
9.7590
9.7554
7.3321
7.3040
7.2844
7.2795
7.2305
6.1659
6.1401
6.1132
6.1034
6.0777
6.0519
5.2777
5.2152
5.1662
5.1515
2.6364
2.6131
2.5935
2.5898
2.5751
2.5519
1.6355
1.6098
1.0083
0.9850
0.9176
0.8919
0.8661
0.8306
0.8122
0.8037
0.7779
0.6077
0.5819
0.5562
0.5292
0.5170
0.5109
0.5060
0.4913
0.4643
0.4410
0.4361
-0.0282
1.1629
4.6351
1.0000
2.0418
1.2221
1.7572
3.2238
6.9084
4.9624
8.5427
Integral
(ppm)

syn-4b

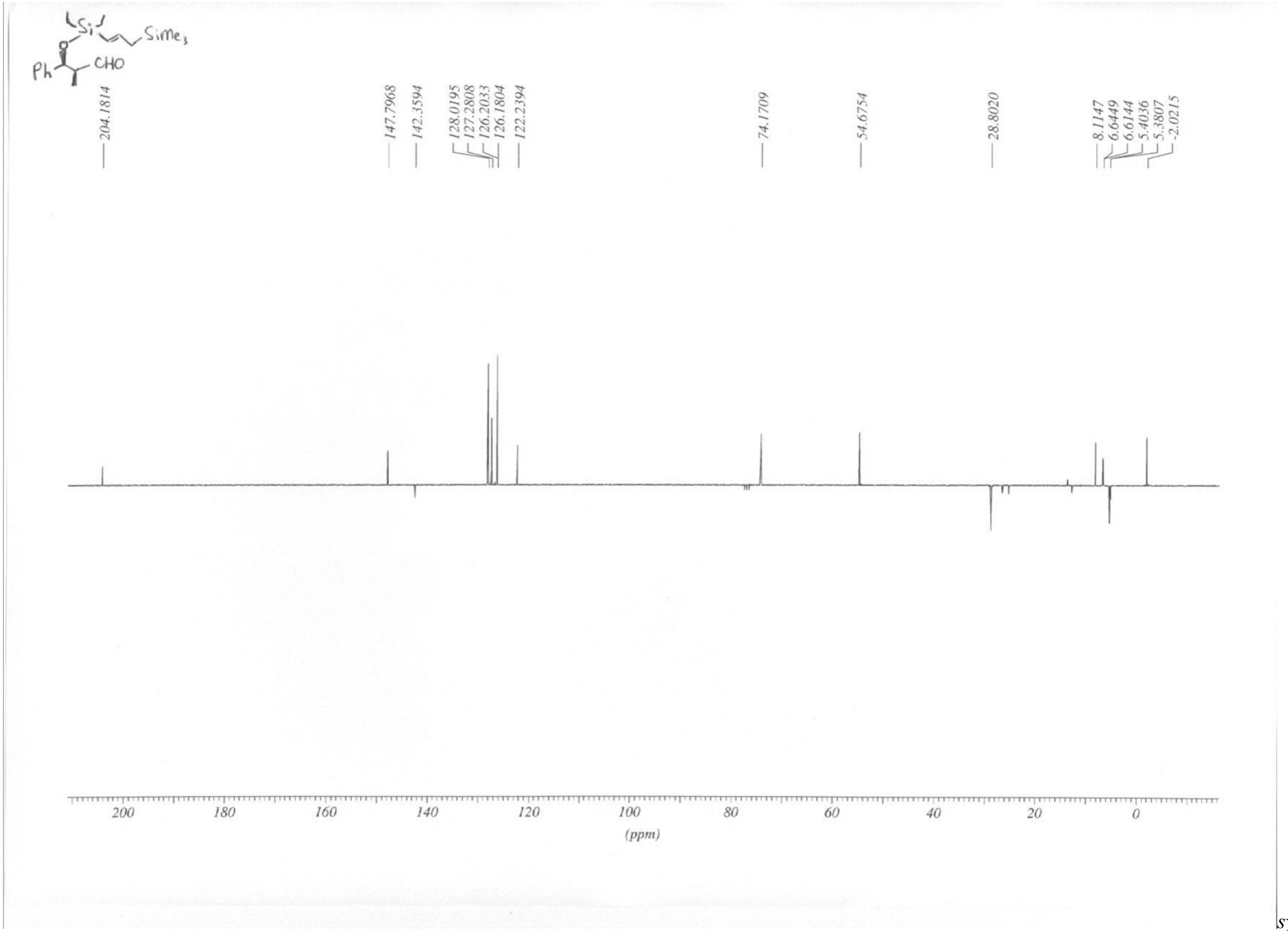

Si
SiMe3
O
Ph
CHO
204.1814
147.7968
142.3594
128.0195
127.2808
126.2033
126.1804
122.2394
74.1709
54.6754
28.8020
8.1147
6.6449
6.6144
5.4036
5.3807
-2.0215
200
180
160
140
120
100
80
60
40
20
0
(ppm)

syn-4b

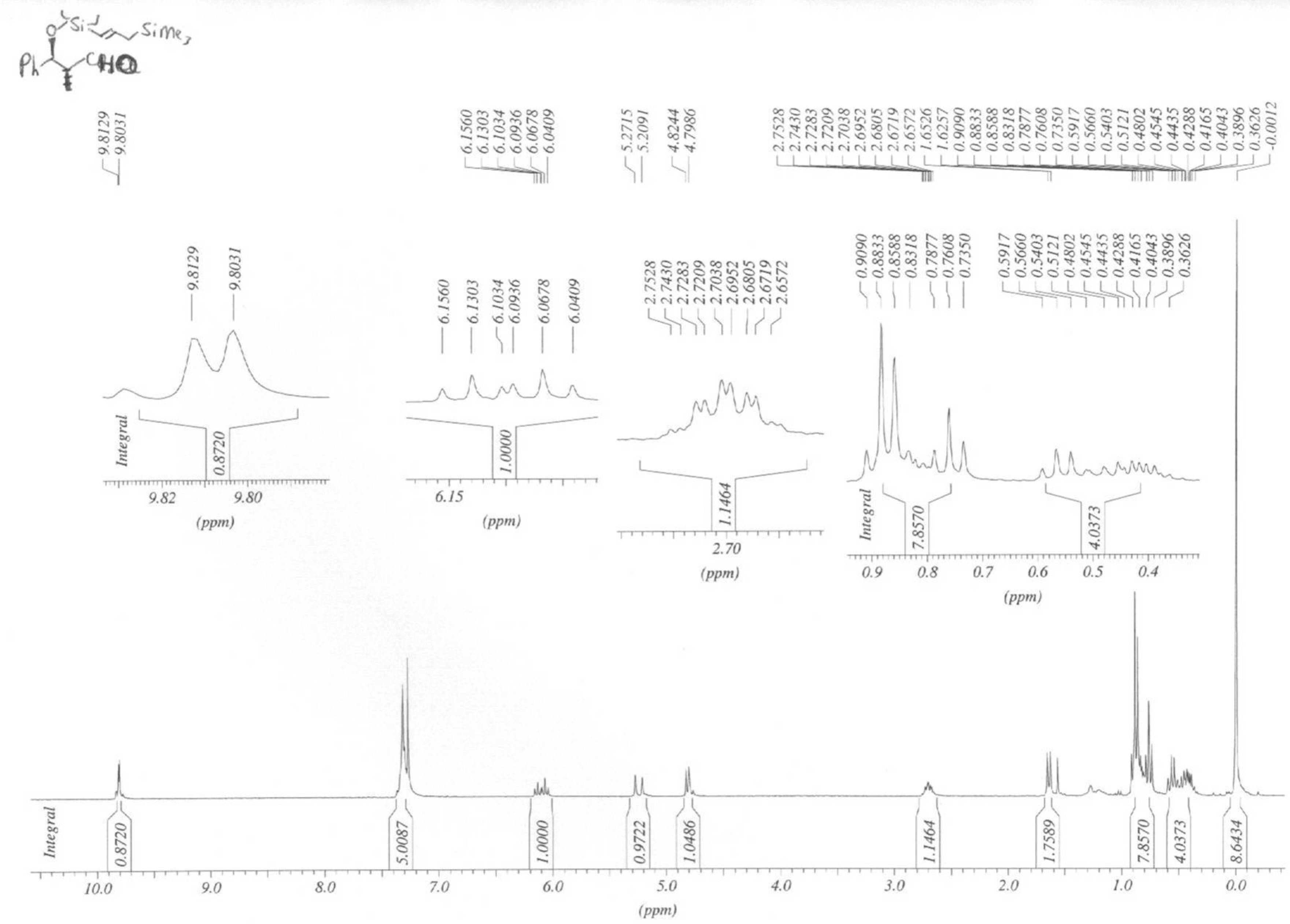
9.8129
9.8031
6.1560
6.1303
6.1034
6.0936
6.0678
6.0409
5.2715
5.2091
4.8244
4.7986
2.7528
2.7430
2.7283
2.7209
2.7038
2.6952
2.6805
2.6719
2.6572
1.6526
1.6257
0.9090
0.8833
0.8588
0.8318
0.7877
0.7608
0.7350
0.5917
0.5660
0.5403
0.5121
0.4802
0.4545
0.4435
0.4288
0.4165
0.4043
0.3896
0.3626
-0.0012
Integral
0.8720
1.0000
1.1464
7.8570
4.0373
5.0087
0.9722
1.0486
1.7589
8.6434
(ppm)

anti-4b

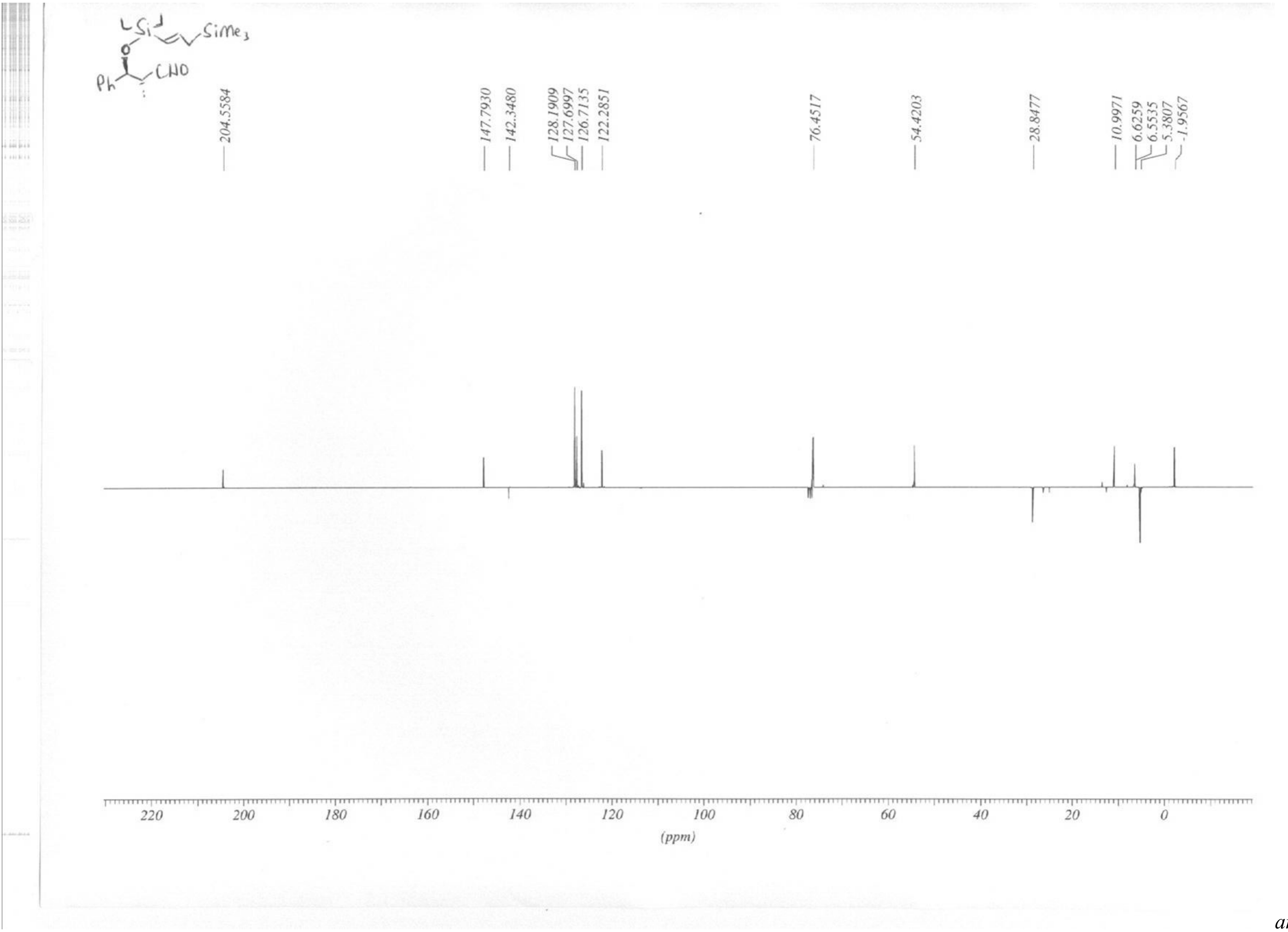

SiMe3
CHO
Ph
204.5584
147.7930
142.3480
128.1909
127.6997
126.7135
122.2851
76.4517
54.4203
28.8477
10.9971
6.6259
6.5535
5.3807
-1.9567
220
200
180
160
140
120
100
80
60
40
20
0
(ppm)

anti-4b

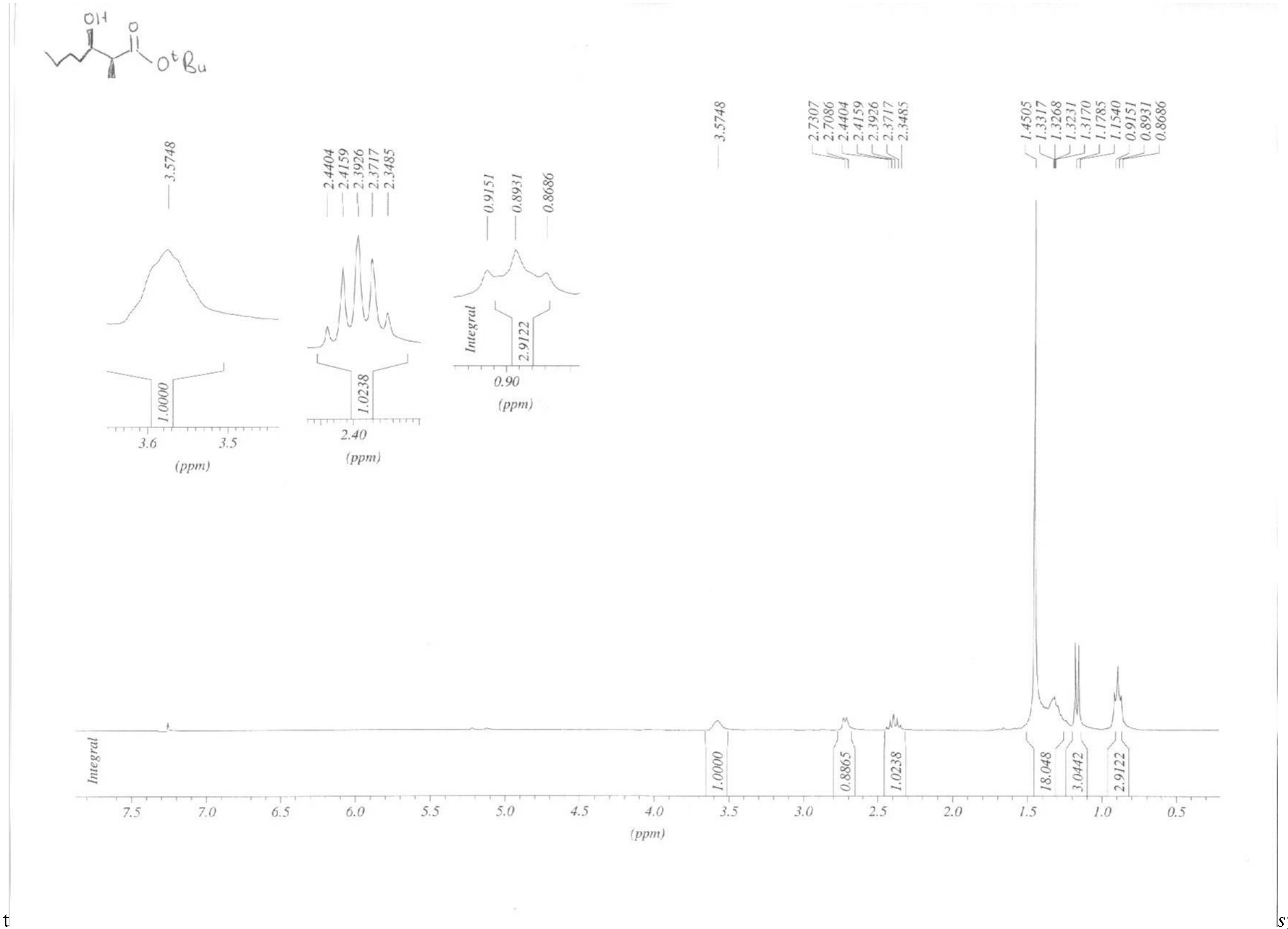

OH
O
O^{t}Bu
3.5748
2.4404
2.4159
2.3926
2.3717
2.3485
0.9151
0.8931
0.8686
Integral
2.9122
0.90
(ppm)
1.0000
3.6
3.5
(ppm)
1.0238
2.40
(ppm)
3.5748
2.7307
2.7086
2.4404
2.4159
2.3926
2.3717
2.3485
1.4505
1.3317
1.3268
1.3231
1.3170
1.1785
1.1540
0.9151
0.8931
0.8686
Integral
1.0000
0.8865
1.0238
18.048
3.0442
2.9122
7.5
7.0
6.5
6.0
5.5
5.0
4.5
4.0
3.5
3.0
2.5
2.0
1.5
1.0
0.5
(ppm)

t

syn-9a

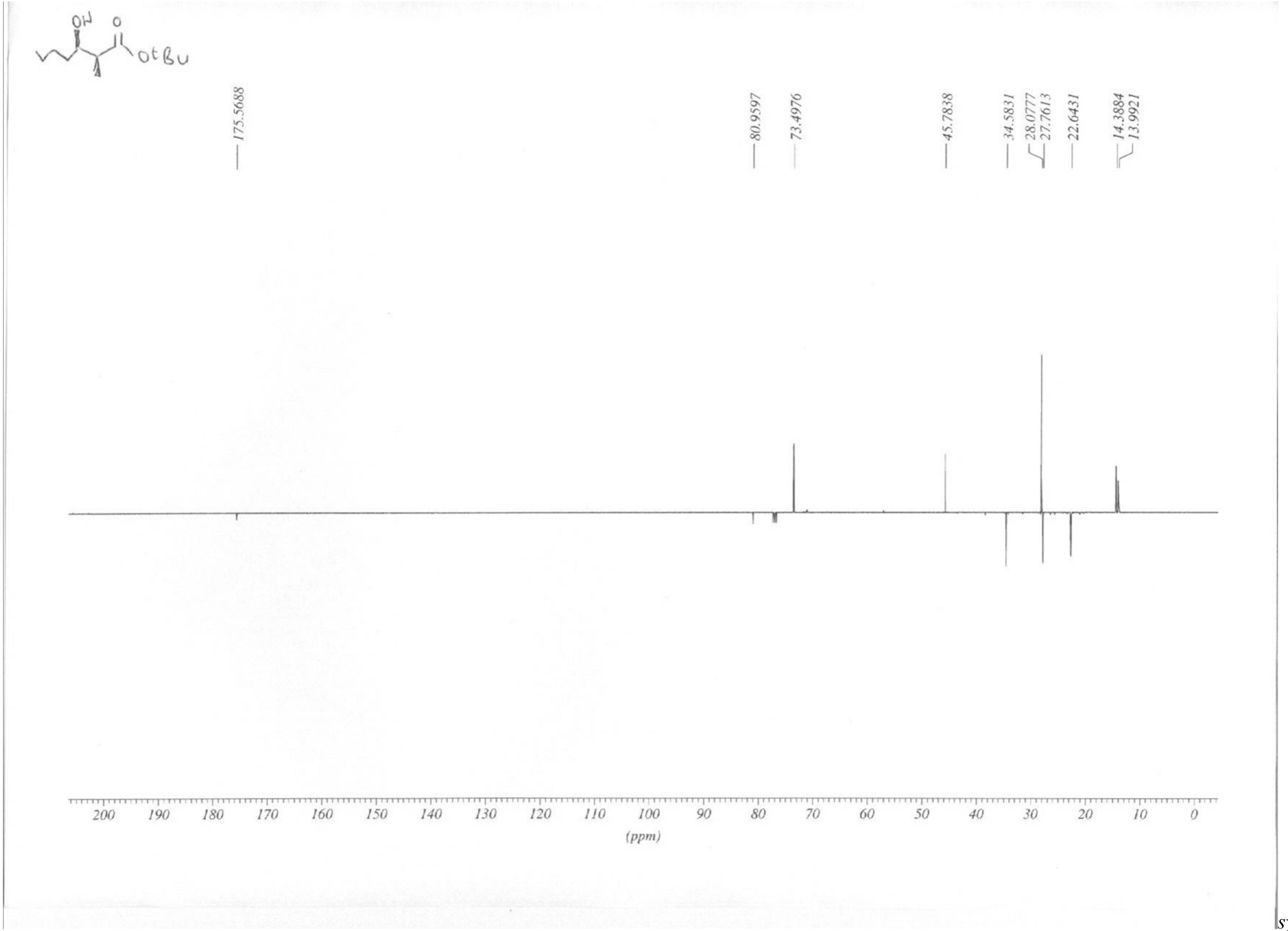
OH O
OtBu
175.5688
80.9597
73.4976
45.7838
34.5831
28.0777
27.7613
22.6431
14.3884
13.9921
200 190 180 170 160 150 140 130 120 110 100 90 80 70 60 50 40 30 20 10 0
(ppm)

syn-9a

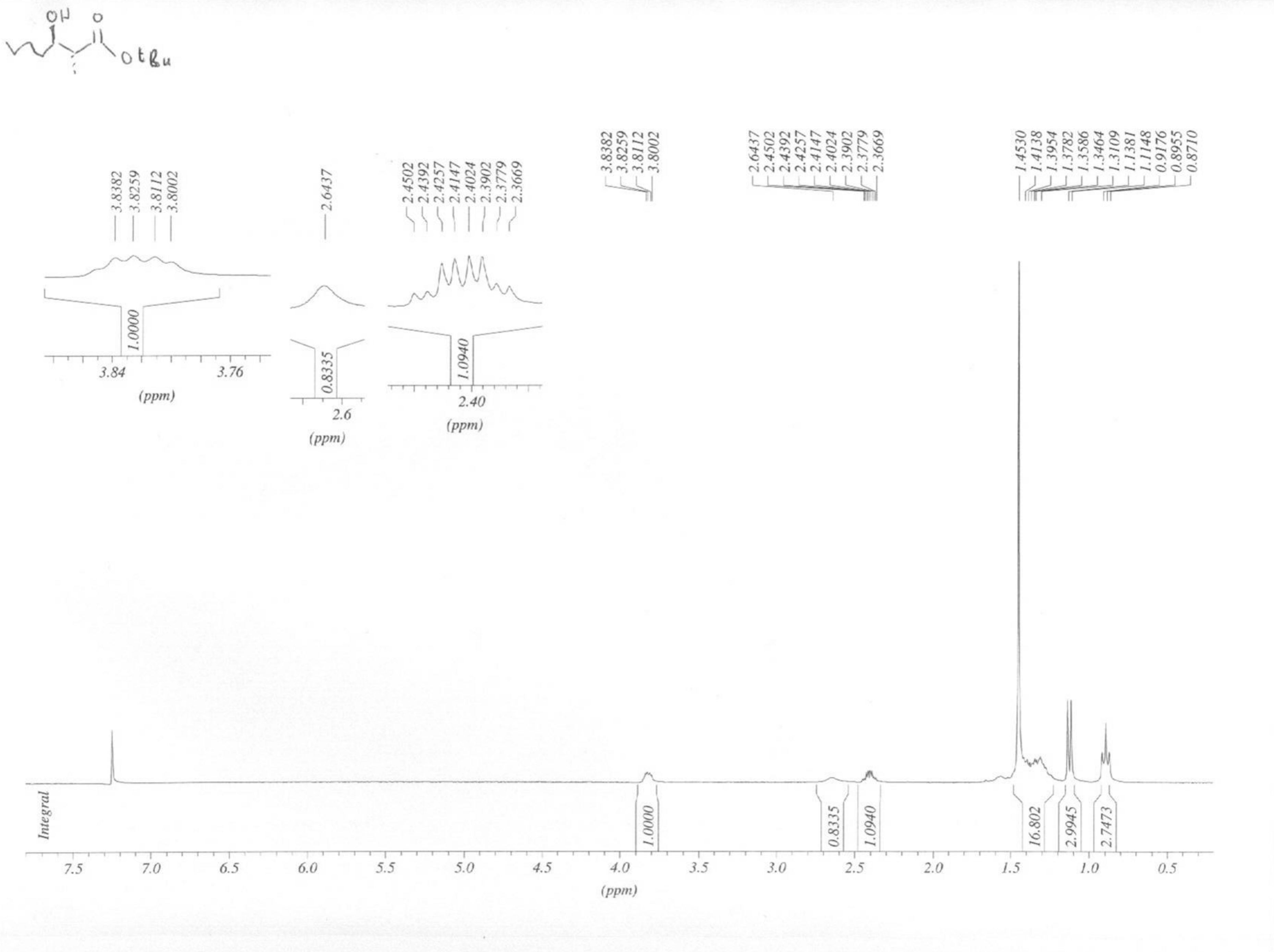
3.8382
3.8259
3.8112
3.8002
1.0000
3.84
3.76
(ppm)
2.6437
0.8335
2.6
(ppm)
2.4502
2.4392
2.4257
2.4147
2.4024
2.3902
2.3779
2.3669
1.0940
2.40
(ppm)
1.4530
1.4138
1.3954
1.3782
1.3586
1.3464
1.3109
1.1381
1.1148
0.9176
0.8955
0.8710
Integral
16.802
2.9945
2.7473
7.5
7.0
6.5
6.0
5.5
5.0
4.5
4.0
3.5
3.0
2.5
2.0
1.5
1.0
0.5
(ppm)

anti-9a

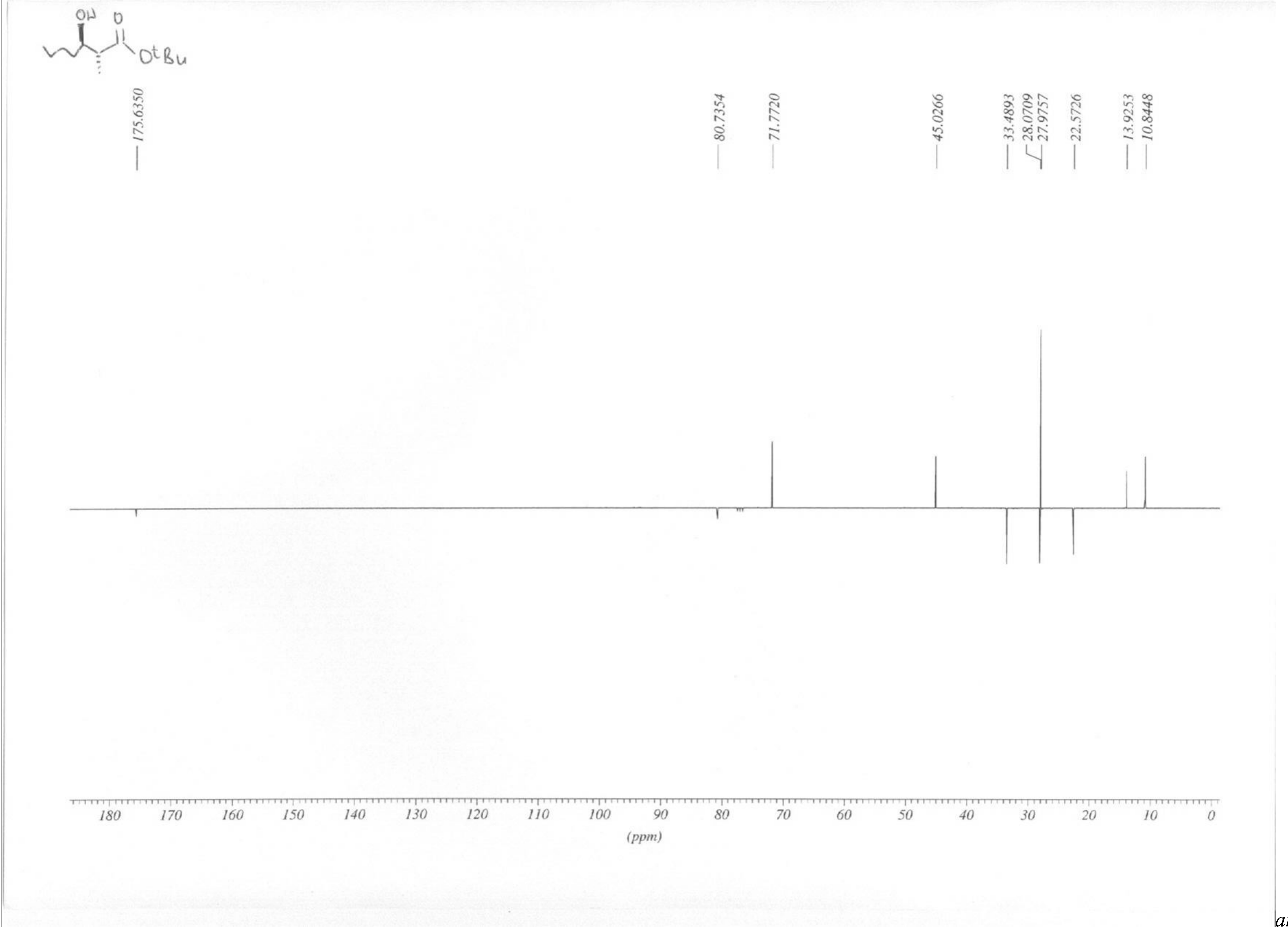
175.6350
80.7354
71.7720
45.0266
33.4893
28.0709
27.9757
22.5726
13.9253
10.8448
180 170 160 150 140 130 120 110 100 90 80 70 60 50 40 30 20 10 0
(ppm)

anti-9a

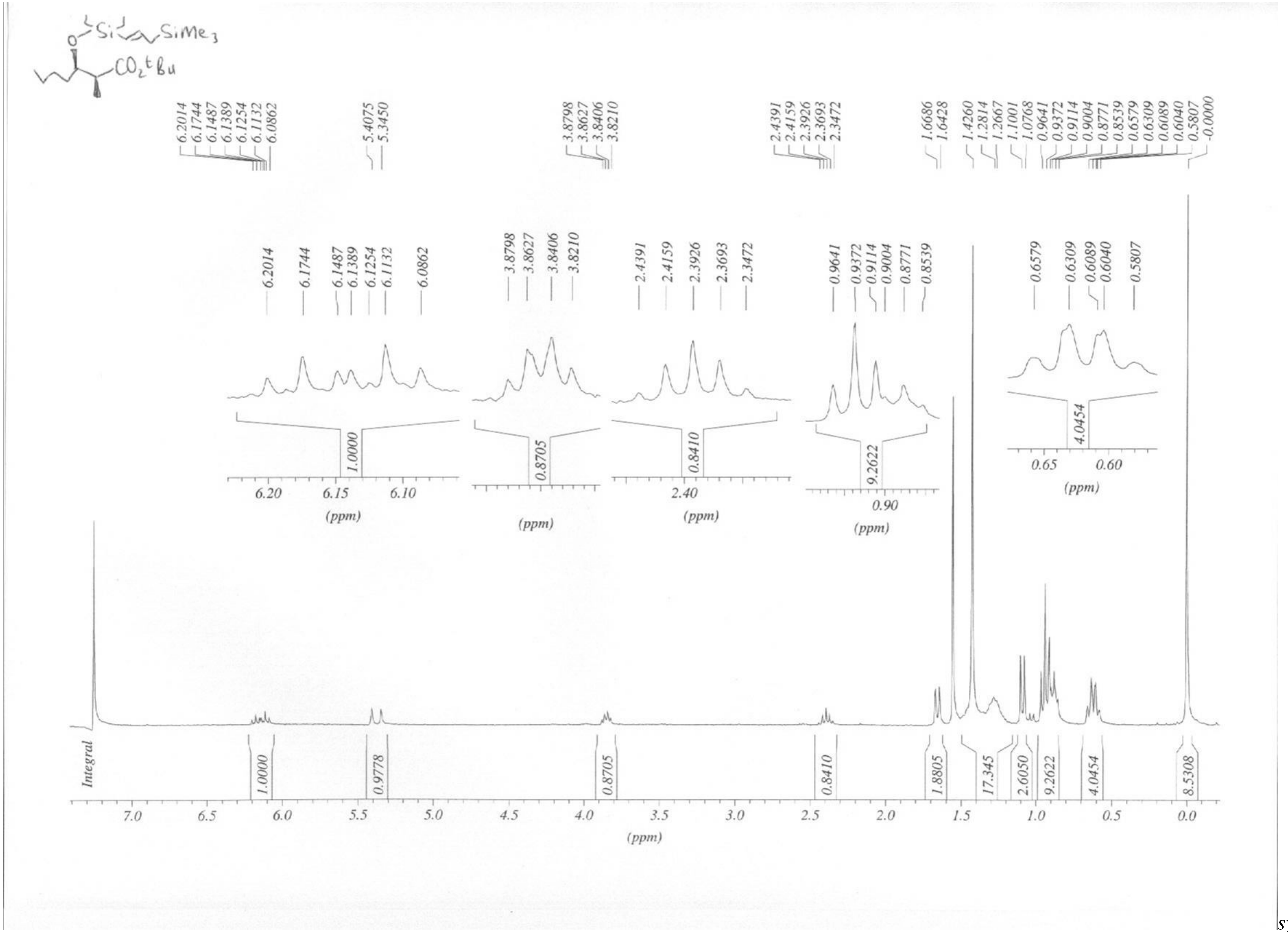

syn-12a

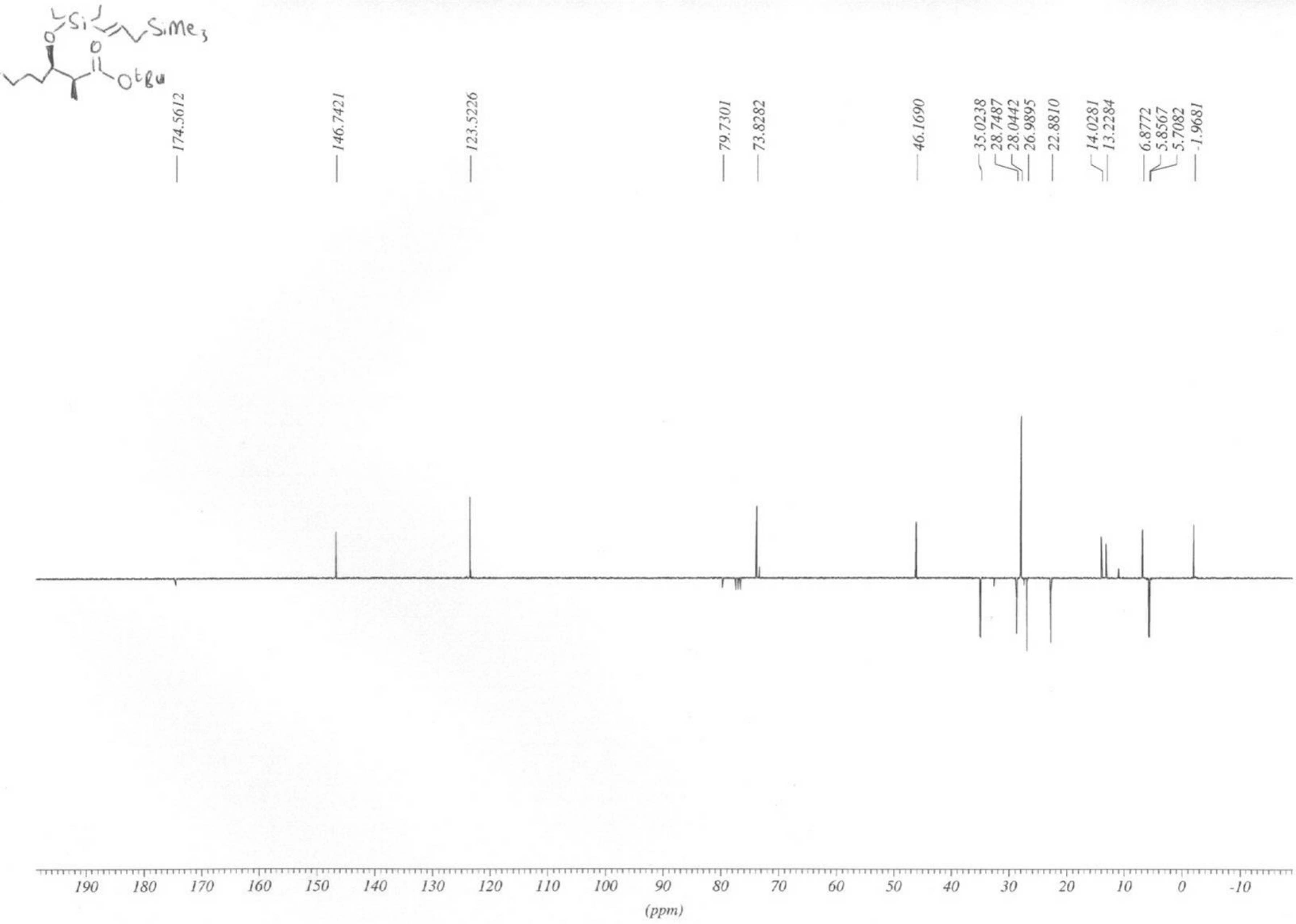
SiMe3
OtBu
174.5612
146.7421
123.5226
79.7301
73.8282
46.1690
35.0238
28.7487
28.0442
26.9895
22.8810
14.0281
13.2284
6.8772
5.8567
5.7082
-1.9681
190 180 170 160 150 140 130 120 110 100 90 80 70 60 50 40 30 20 10 0 -10
(ppm)

syn-12a

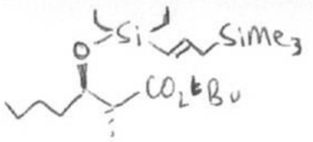
SiMe3
CO2tBu

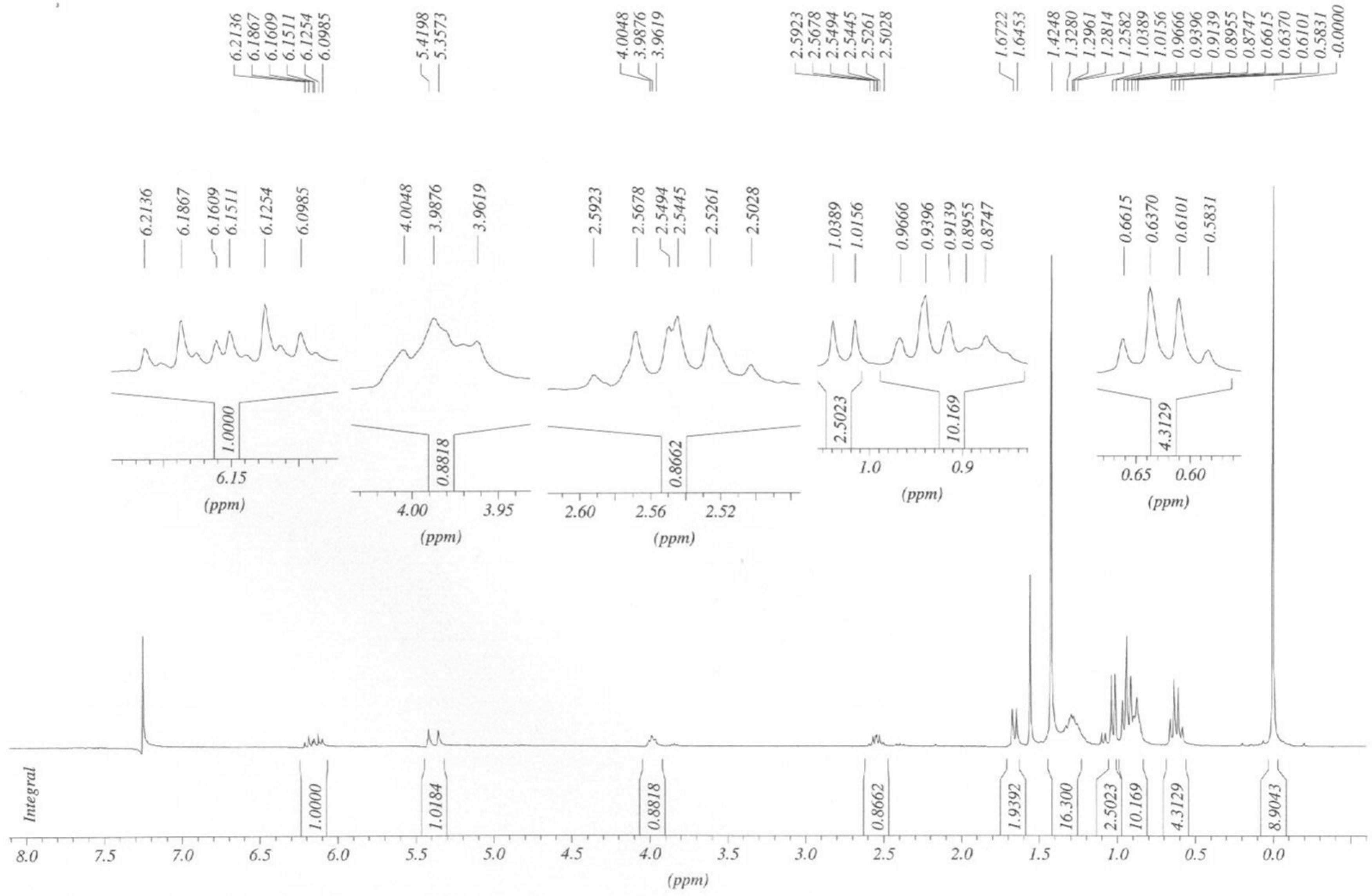
6.2136
6.1867
6.1609
6.1511
6.1254
6.0985
5.4198
5.3573
4.0048
3.9876
3.9619
2.5923
2.5678
2.5494
2.5445
2.5261
2.5028
1.6722
1.6453
1.4248
1.3280
1.2961
1.2814
1.2582
1.0389
1.0156
0.9666
0.9396
0.9139
0.8955
0.8747
0.6615
0.6370
0.6101
0.5831
-0.0000
Integral
1.0000
1.0184
0.8818
0.8662
1.9392
16.300
2.5023
10.169
4.3129
8.9043
(ppm)

anti-12a

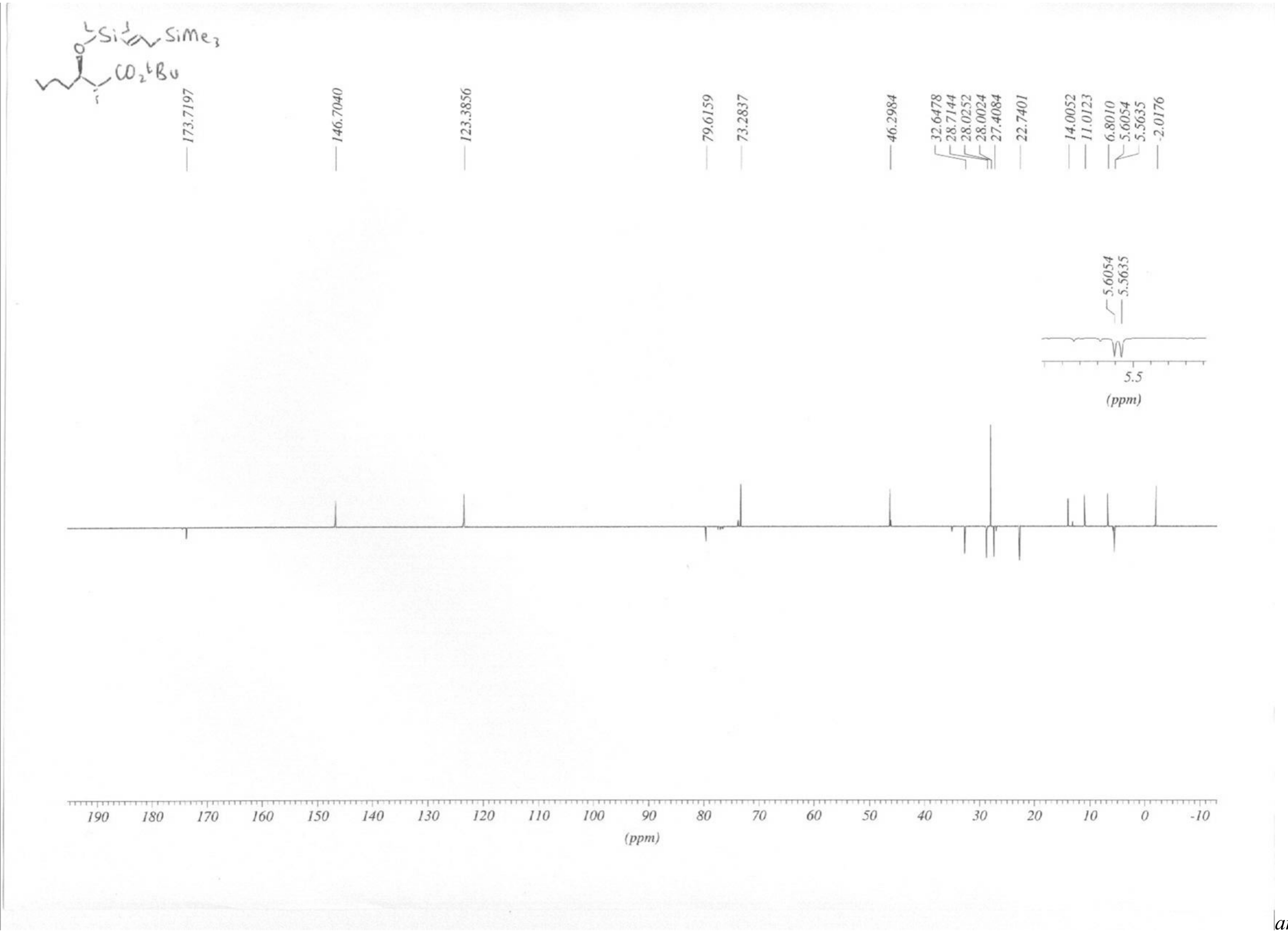
SiMe3
CO2tBu
173.7197
146.7040
123.3856
79.6159
73.2837
46.2984
32.6478
28.7144
28.0252
28.0024
27.4084
22.7401
14.0052
11.0123
6.8010
5.6054
5.5635
-2.0176
5.6054
5.5635
5.5
(ppm)
190
180
170
160
150
140
130
120
110
100
90
80
70
60
50
40
30
20
10
0
-10
(ppm)

anti-12a

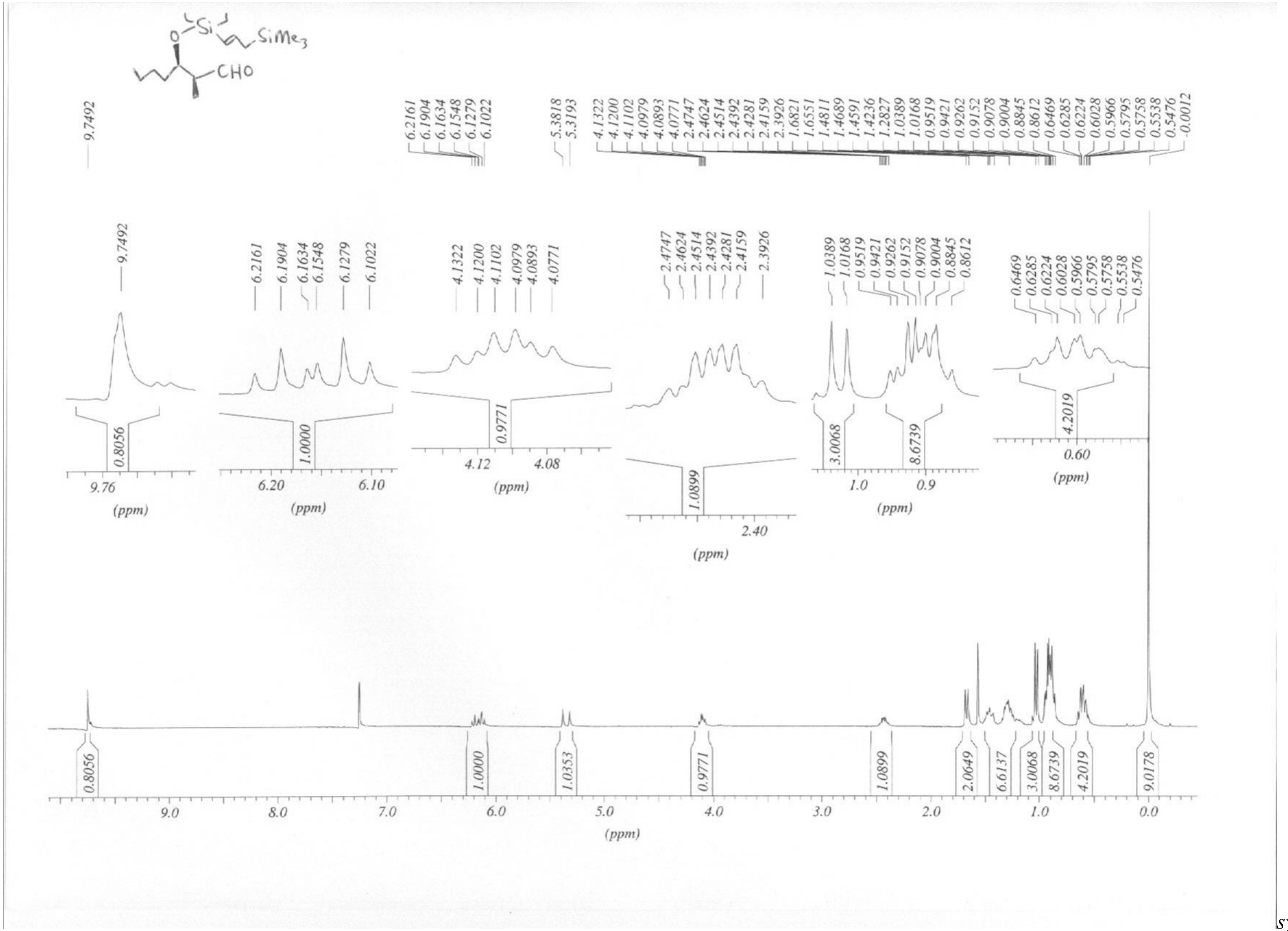

syn-4a

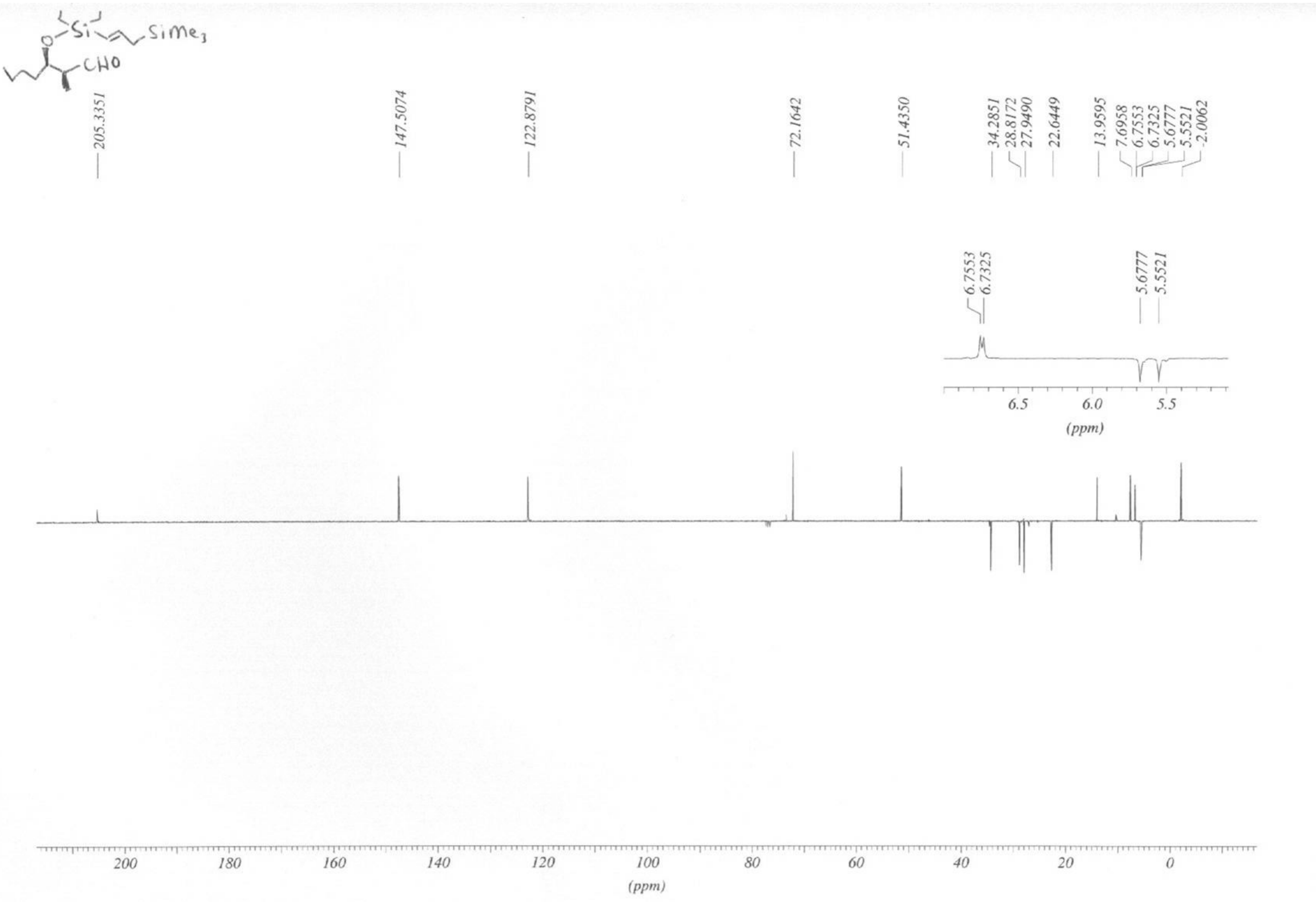
SiMe3
CHO
205.3351
147.5074
122.8791
72.1642
51.4350
34.2851
28.8172
27.9490
22.6449
13.9595
7.6958
6.7553
6.7325
5.6777
5.5521
-2.0062
6.7553
6.7325
5.6777
5.5521
6.5
6.0
5.5
(ppm)
200
180
160
140
120
100
80
60
40
20
0
(ppm)

syn-4a

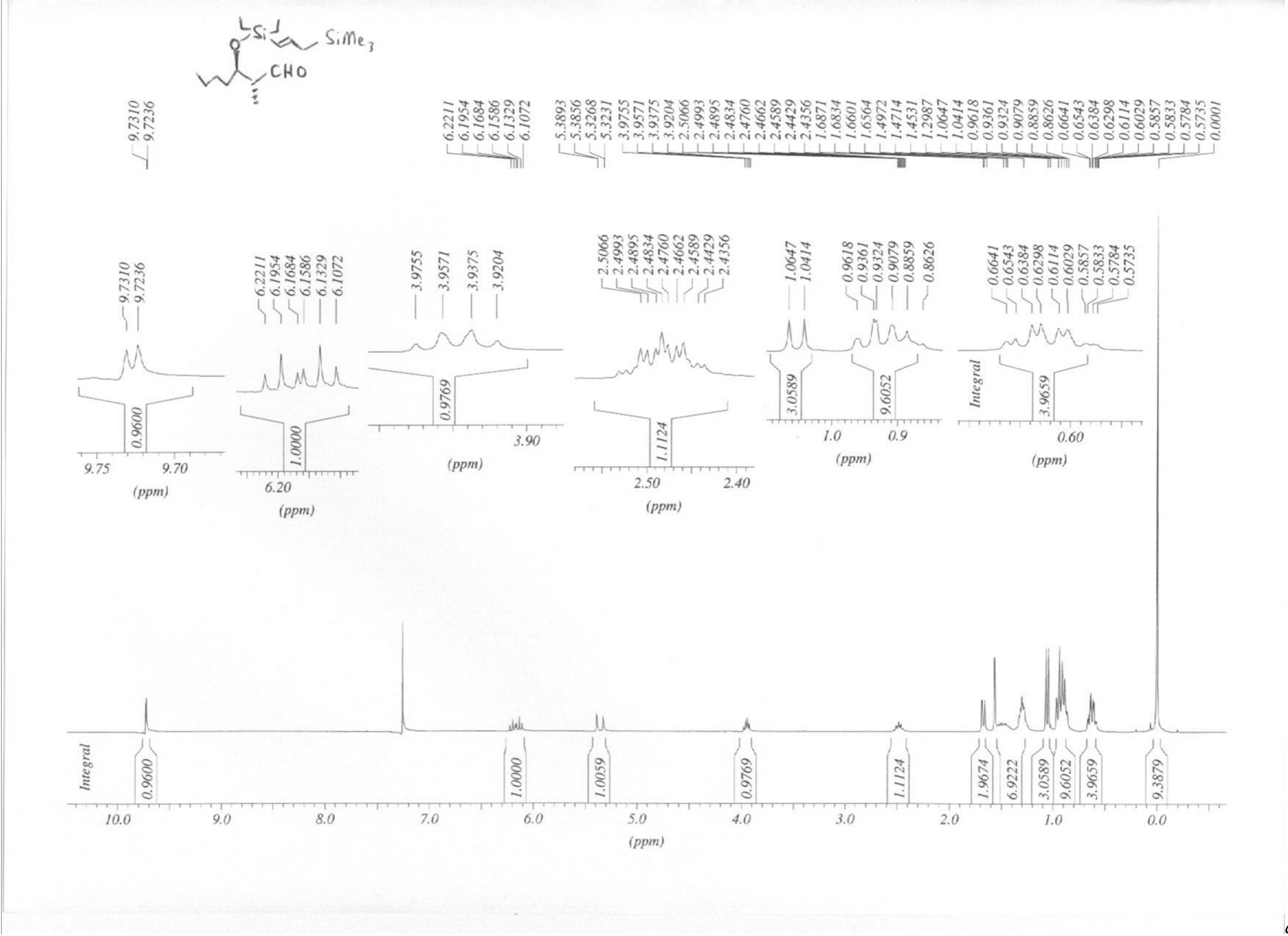

SiMe3
CHO
9.7310
9.7236
6.2211
6.1954
6.1684
6.1586
6.1329
6.1072
5.3893
5.3856
5.3268
5.3231
3.9755
3.9571
3.9375
3.9204
2.5066
2.4993
2.4895
2.4834
2.4760
2.4662
2.4589
2.4429
2.4356
1.6871
1.6834
1.6601
1.6564
1.4972
1.4714
1.4531
1.2987
1.0647
1.0414
0.9618
0.9361
0.9324
0.9079
0.8859
0.8626
0.6641
0.6543
0.6384
0.6298
0.6114
0.6029
0.5857
0.5833
0.5784
0.5735
0.0001
Integral
0.9600
1.0000
1.0059
0.9769
1.1124
1.9674
6.9222
3.0589
9.6052
3.9659
9.3879
(ppm)

anti-4a

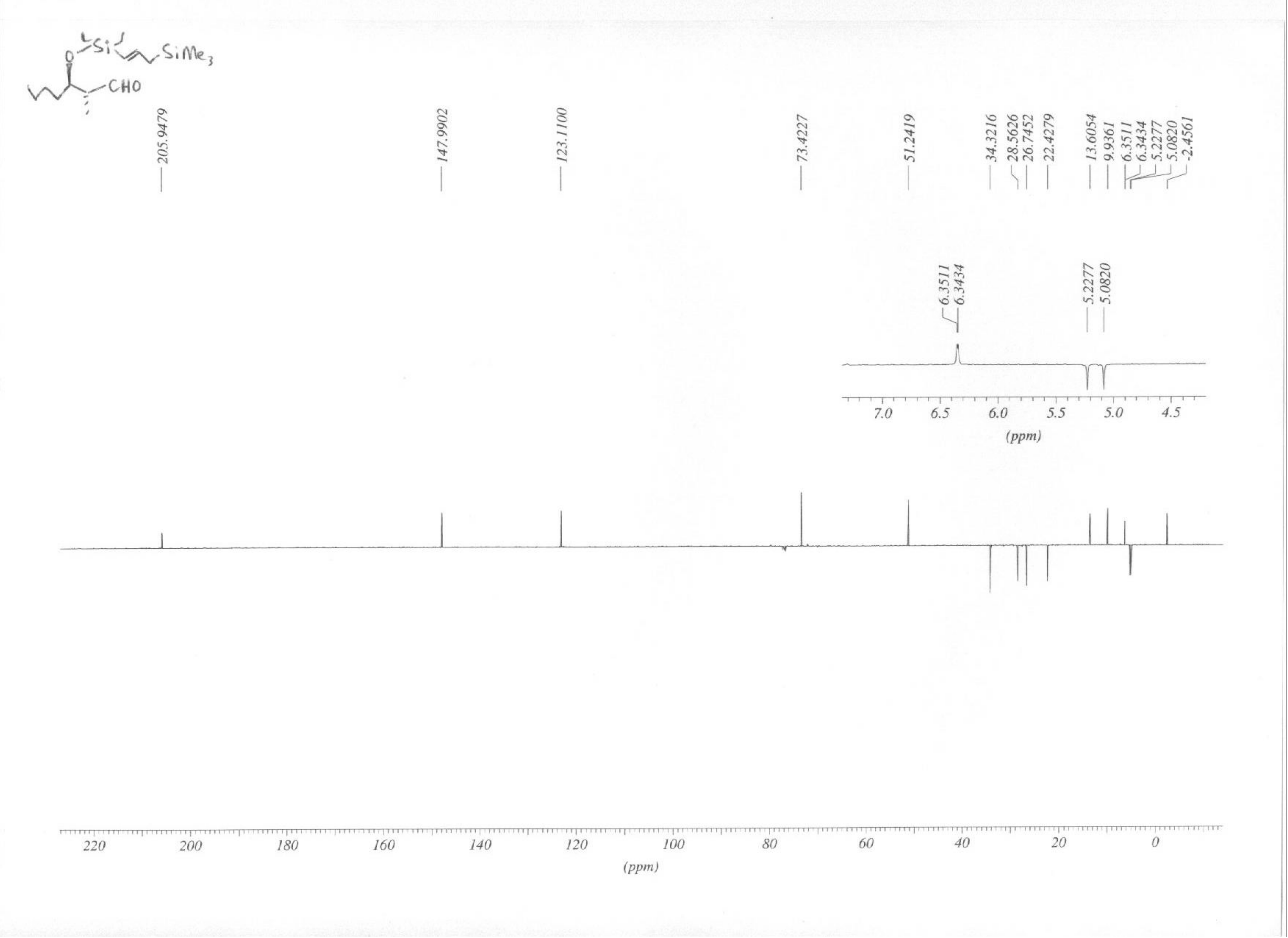
SiMe3
CHO
205.9479
147.9902
123.1100
73.4227
51.2419
34.3216
28.5626
26.7452
22.4279
13.6054
9.9361
6.3511
6.3434
5.2277
5.0820
-2.4561
(ppm)

anti-4a

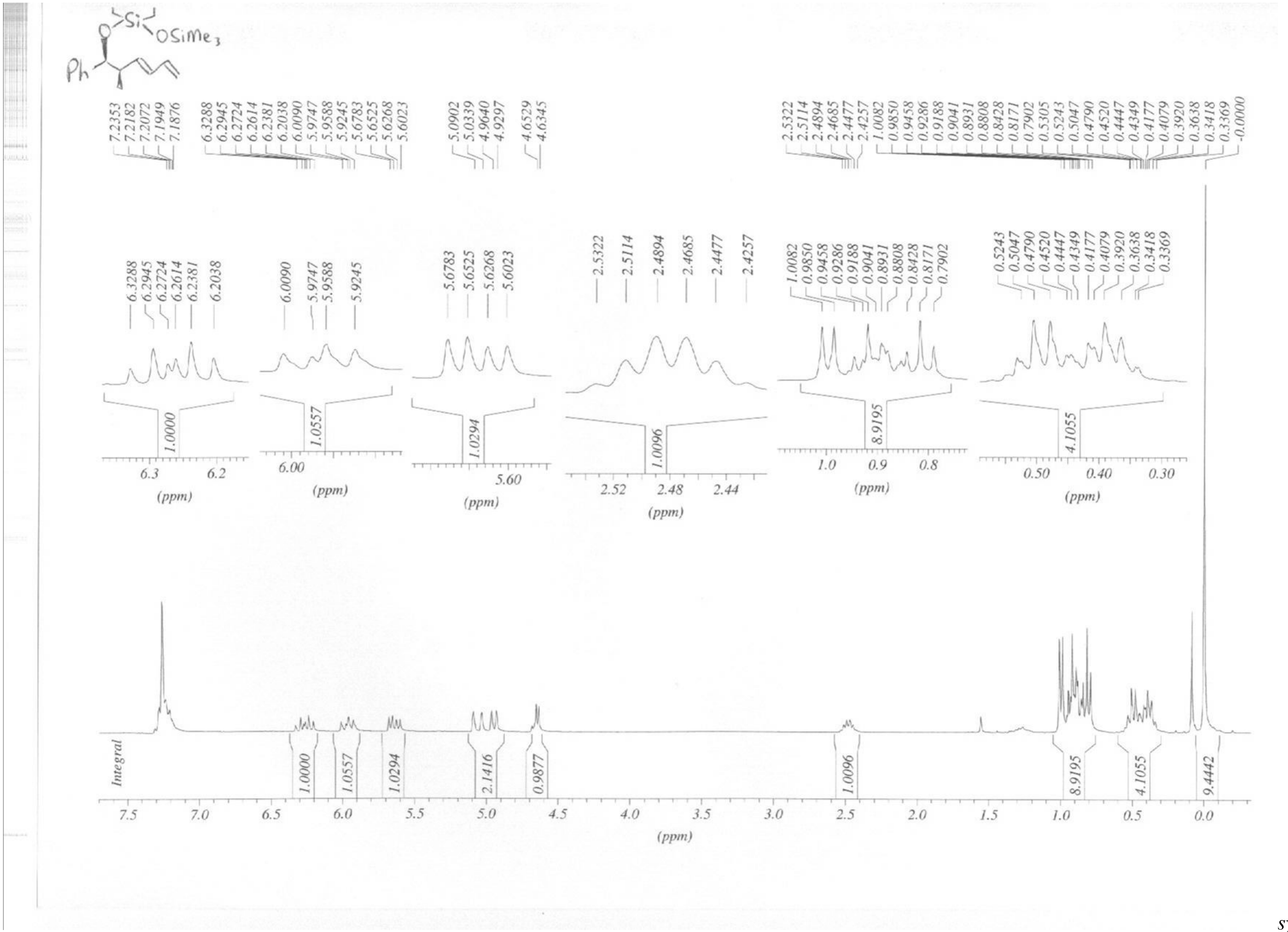
OSiMe3
Ph
7.2353 7.2182 7.2072 7.1949 7.1876
6.3288 6.2945 6.2724 6.2614 6.2381 6.2038 6.0090 5.9747 5.9588 5.9245 5.6783 5.6525 5.6268 5.6023
5.0902 5.0339 4.9640 4.9297
4.6529 4.6345
2.5322 2.5114 2.4894 2.4685 2.4477 2.4257
1.0082 0.9850 0.9458 0.9286 0.9188 0.9041 0.8931 0.8808 0.8428 0.8171 0.7902
0.5305 0.5243 0.5047 0.4790 0.4520 0.4447 0.4349 0.4177 0.4079 0.3920 0.3638 0.3418 0.3369
-0.0000
1.0000
1.0557
1.0294
1.0096
8.9195
4.1055
(ppm)
Integral
1.0000 1.0557 1.0294 2.1416 0.9877 1.0096 8.9195 4.1055 9.4442
7.5 7.0 6.5 6.0 5.5 5.0 4.5 4.0 3.5 3.0 2.5 2.0 1.5 1.0 0.5 0.0
(ppm)

syn-13b

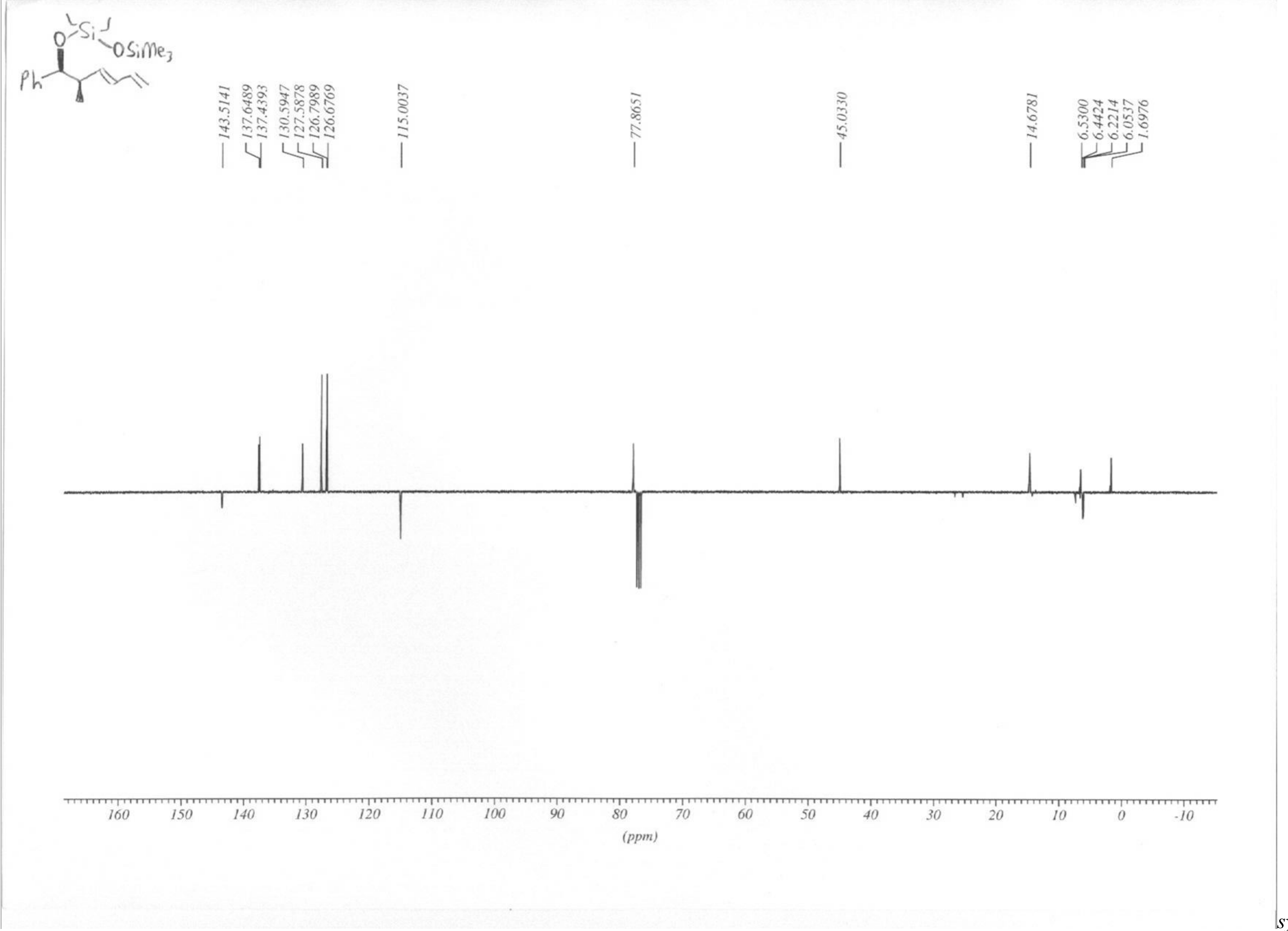

143.5141
137.6489
137.4393
130.5947
127.5878
126.7989
126.6769
115.0037
77.8651
45.0330
14.6781
6.5300
6.4424
6.2214
6.0537
1.6976
160 150 140 130 120 110 100 90 80 70 60 50 40 30 20 10 0 -10
(ppm)

syn-13b

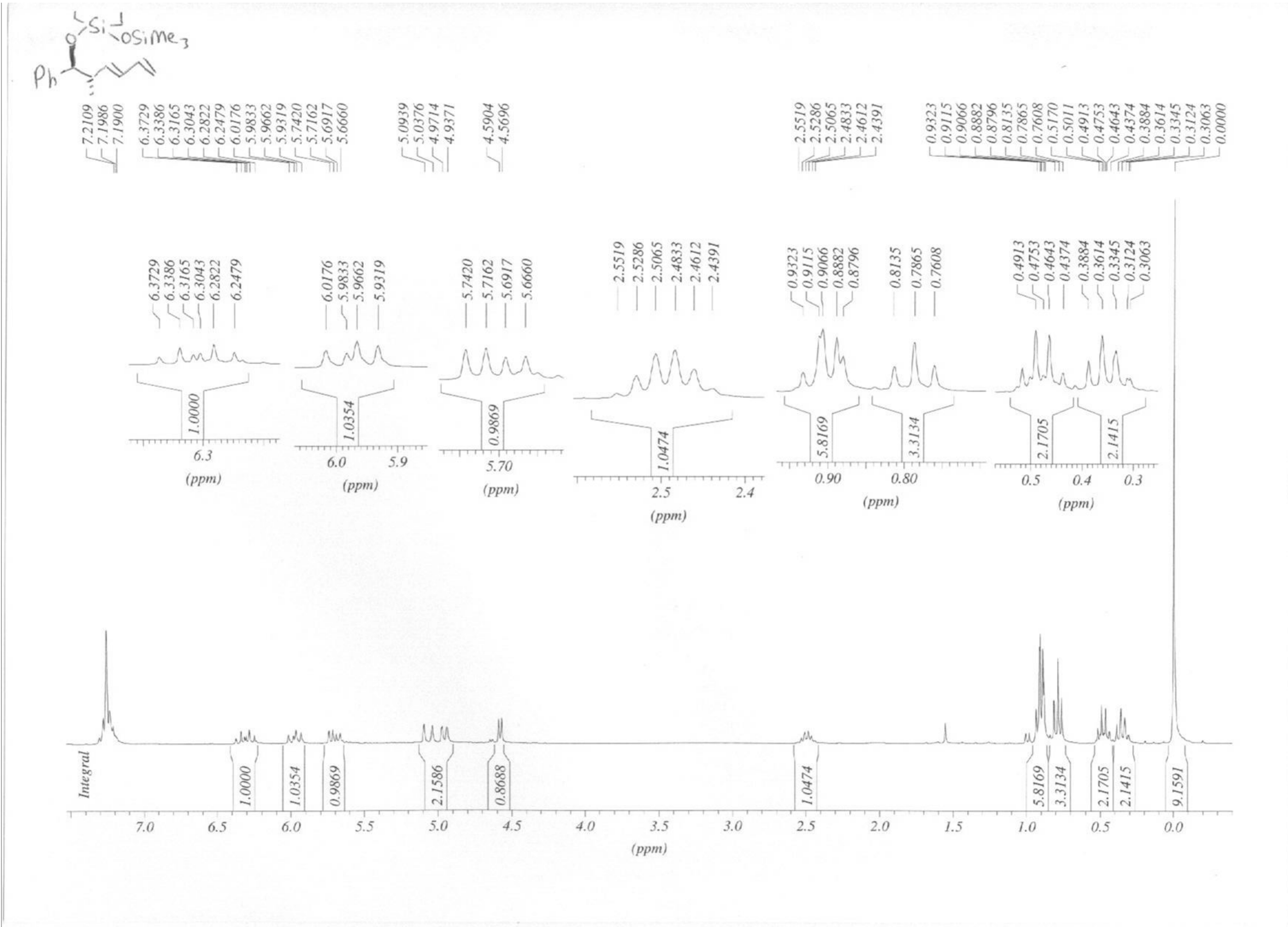

OSiMe3
Ph
7.2109
7.1986
7.1900
6.3729
6.3386
6.3165
6.3043
6.2822
6.2479
6.0176
5.9833
5.9662
5.9319
5.7420
5.7162
5.6917
5.6660
5.0939
5.0376
4.9714
4.9371
4.5904
4.5696
2.5519
2.5286
2.5065
2.4833
2.4612
2.4391
0.9323
0.9115
0.9066
0.8882
0.8796
0.8135
0.7865
0.7608
0.5170
0.5011
0.4913
0.4753
0.4643
0.4374
0.3884
0.3614
0.3345
0.3124
0.3063
0.0000
1.0000
1.0354
0.9869
1.0474
5.8169
3.1134
2.1705
2.1415
6.3
6.0
5.9
5.70
2.5
2.4
0.90
0.80
0.5
0.4
0.3
(ppm)
Integral
1.0000
1.0354
0.9869
2.1586
0.8688
1.0474
5.8169
3.3134
2.1705
2.1415
9.1591
7.0
6.5
6.0
5.5
5.0
4.5
4.0
3.5
3.0
2.5
2.0
1.5
1.0
0.5
0.0
(ppm)

anti-13b

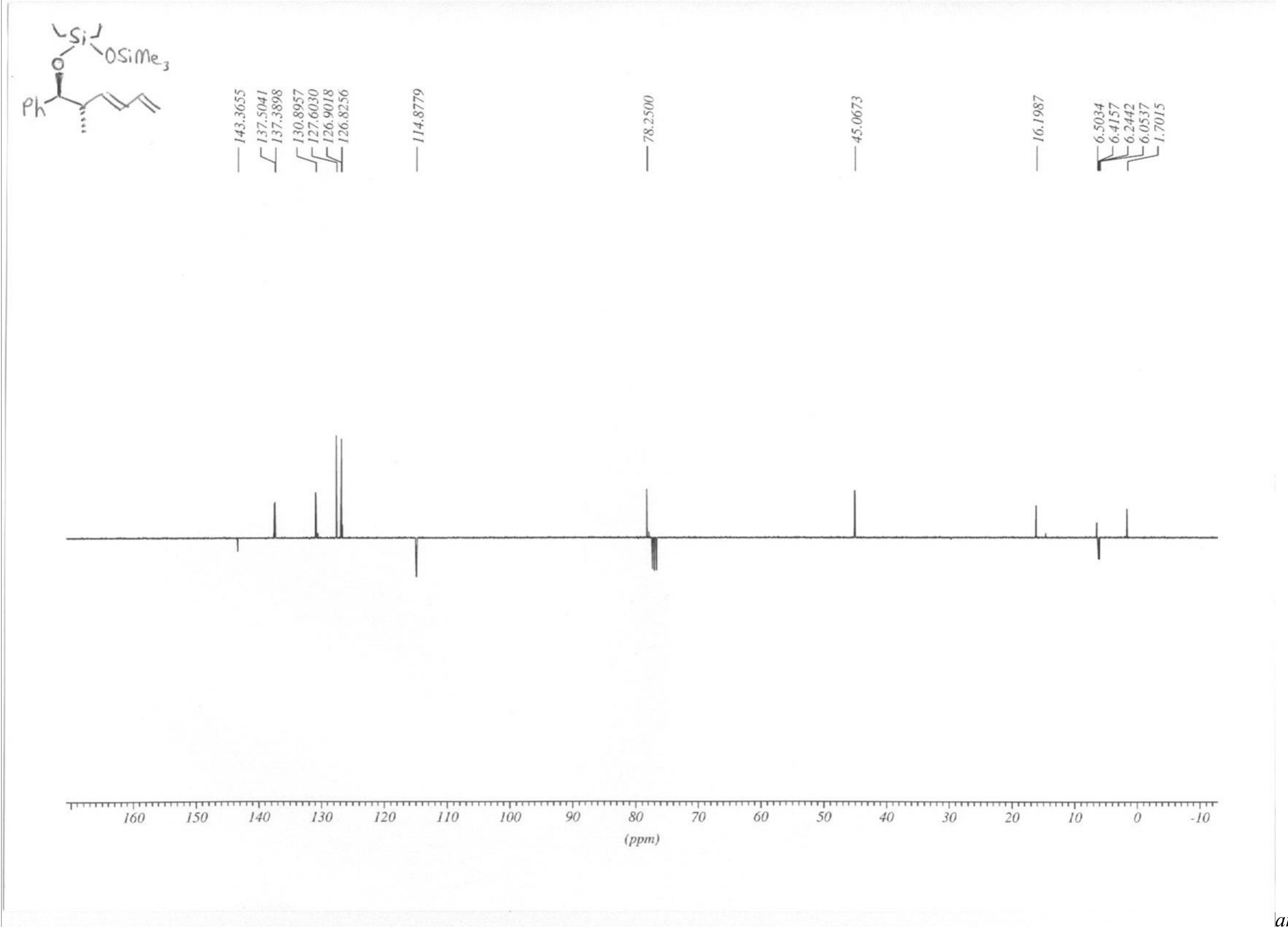
OSiMe3
Ph
143.3655
137.5041
137.3898
130.8957
127.6030
126.9018
126.8256
114.8779
78.2500
45.0673
16.1987
6.5034
6.4157
6.2442
6.0537
1.7015
160 150 140 130 120 110 100 90 80 70 60 50 40 30 20 10 0 -10
(ppm)

anti-13b

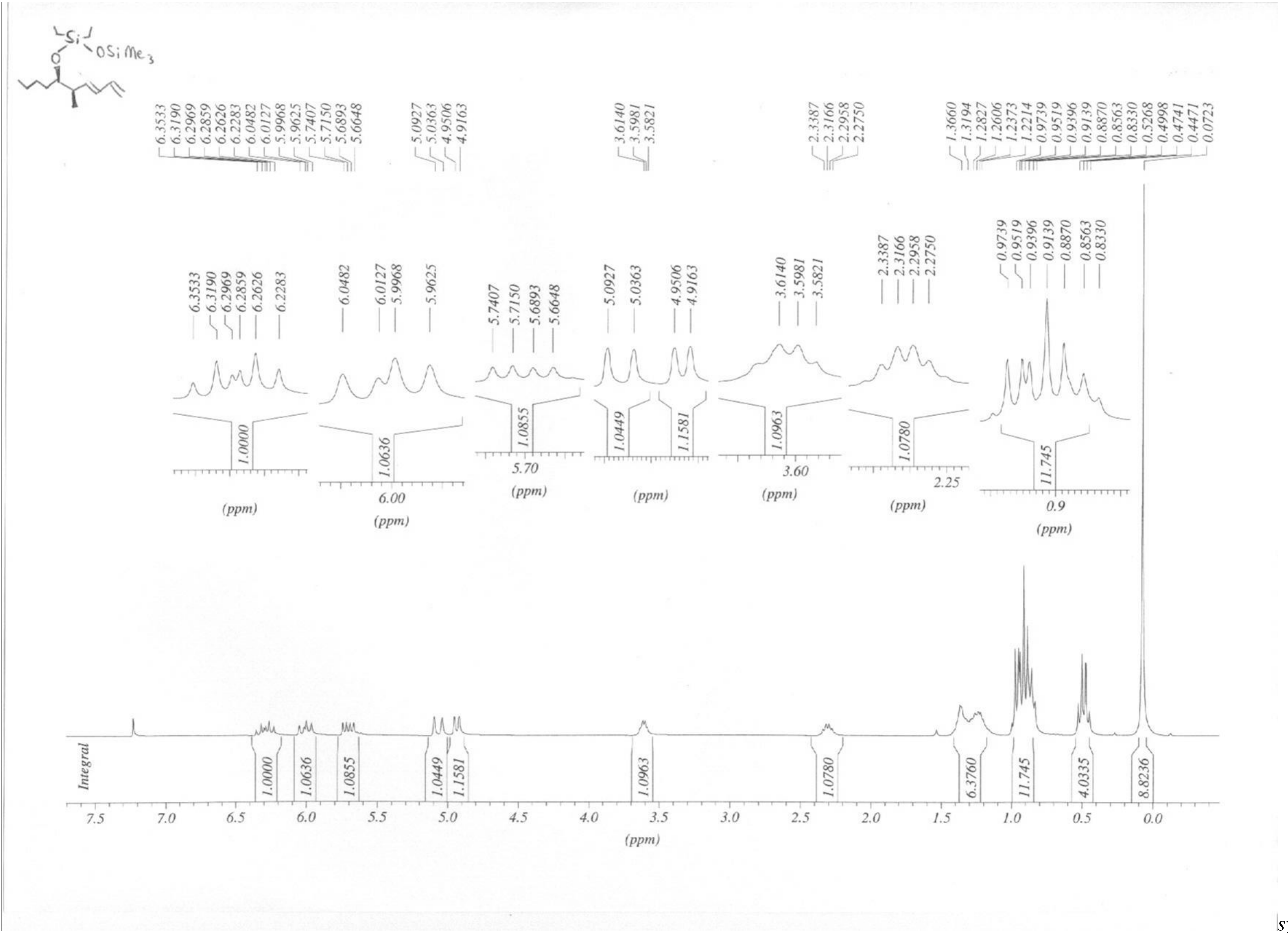
OSiMe3
6.3533 6.3190 6.2969 6.2859 6.2626 6.2283 6.0482 6.0127 5.9968 5.9625 5.7407 5.7150 5.6893 5.6648
5.0927 5.0363 4.9506 4.9163
3.6140 3.5981 3.5821
2.3387 2.3166 2.2958 2.2750
1.3660 1.3194 1.2827 1.2606 1.2373 1.2214 0.9739 0.9519 0.9396 0.9139 0.8870 0.8563 0.8330 0.5268 0.4998 0.4741 0.4471 0.0723
1.0000
1.0636
6.00
1.0855
5.70
1.0449
1.1581
1.0963
3.60
1.0780
2.25
11.745
0.9
(ppm)
Integral
1.0000 1.0636 1.0855 1.0449 1.1581 1.0963 1.0780 6.3760 11.745 4.0335 8.8236
7.5 7.0 6.5 6.0 5.5 5.0 4.5 4.0 3.5 3.0 2.5 2.0 1.5 1.0 0.5 0.0
(ppm)

syn-13a

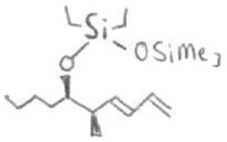
OSiMe3

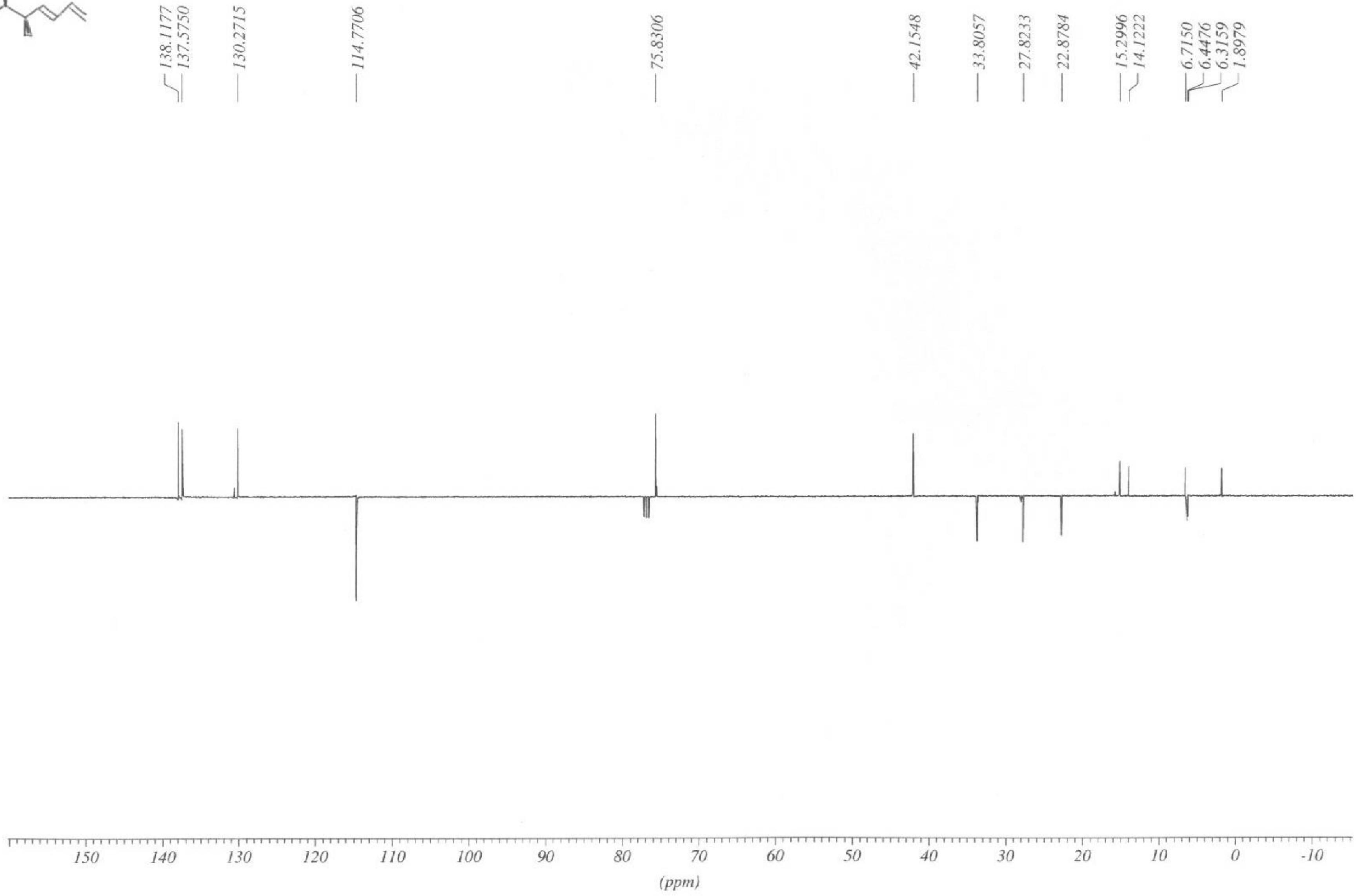
138.1177
137.5750
130.2715
114.7706
75.8306
42.1548
33.8057
27.8233
22.8784
15.2996
14.1222
6.7150
6.4476
6.3159
1.8979
150 140 130 120 110 100 90 80 70 60 50 40 30 20 10 0 -10
(ppm)

syn-13a

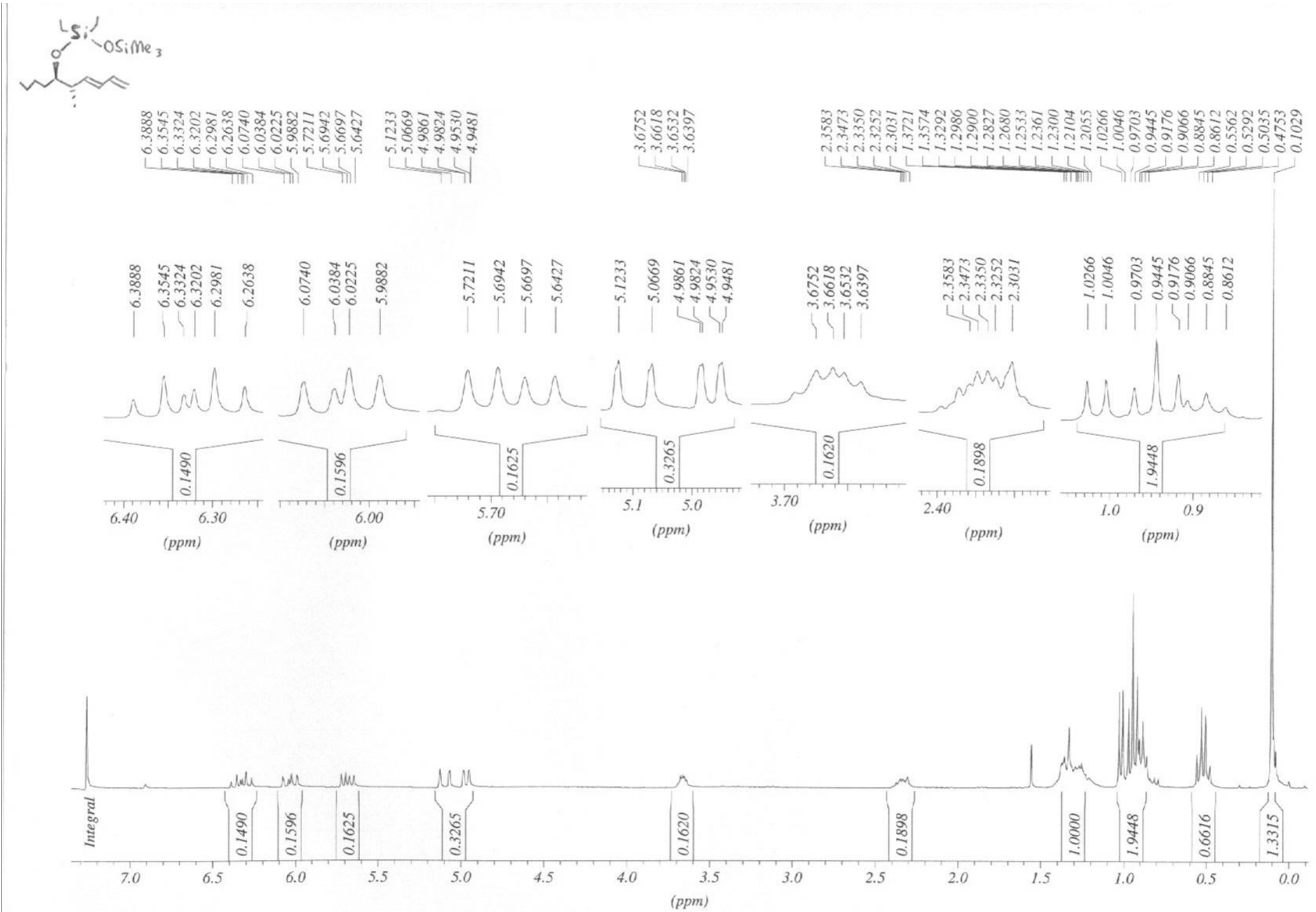
OSiMe3
6.3888
6.3545
6.3324
6.3202
6.2981
6.2638
6.0740
6.0384
6.0225
5.9882
5.7211
5.6942
5.6697
5.6427
5.1233
5.0669
4.9861
4.9824
4.9530
4.9481
3.6752
3.6618
3.6532
3.6397
2.3583
2.3473
2.3350
2.3252
2.3031
1.3721
1.3574
1.3292
1.2986
1.2900
1.2827
1.2680
1.2533
1.2361
1.2300
1.2104
1.2055
1.0266
1.0046
0.9703
0.9445
0.9176
0.9066
0.8845
0.8612
0.5562
0.5292
0.5035
0.4753
0.1029
0.1490
0.1596
0.1625
0.3265
0.1620
0.1898
1.9448
(ppm)
Integral
1.0000
0.6616
1.3315

anti-13a

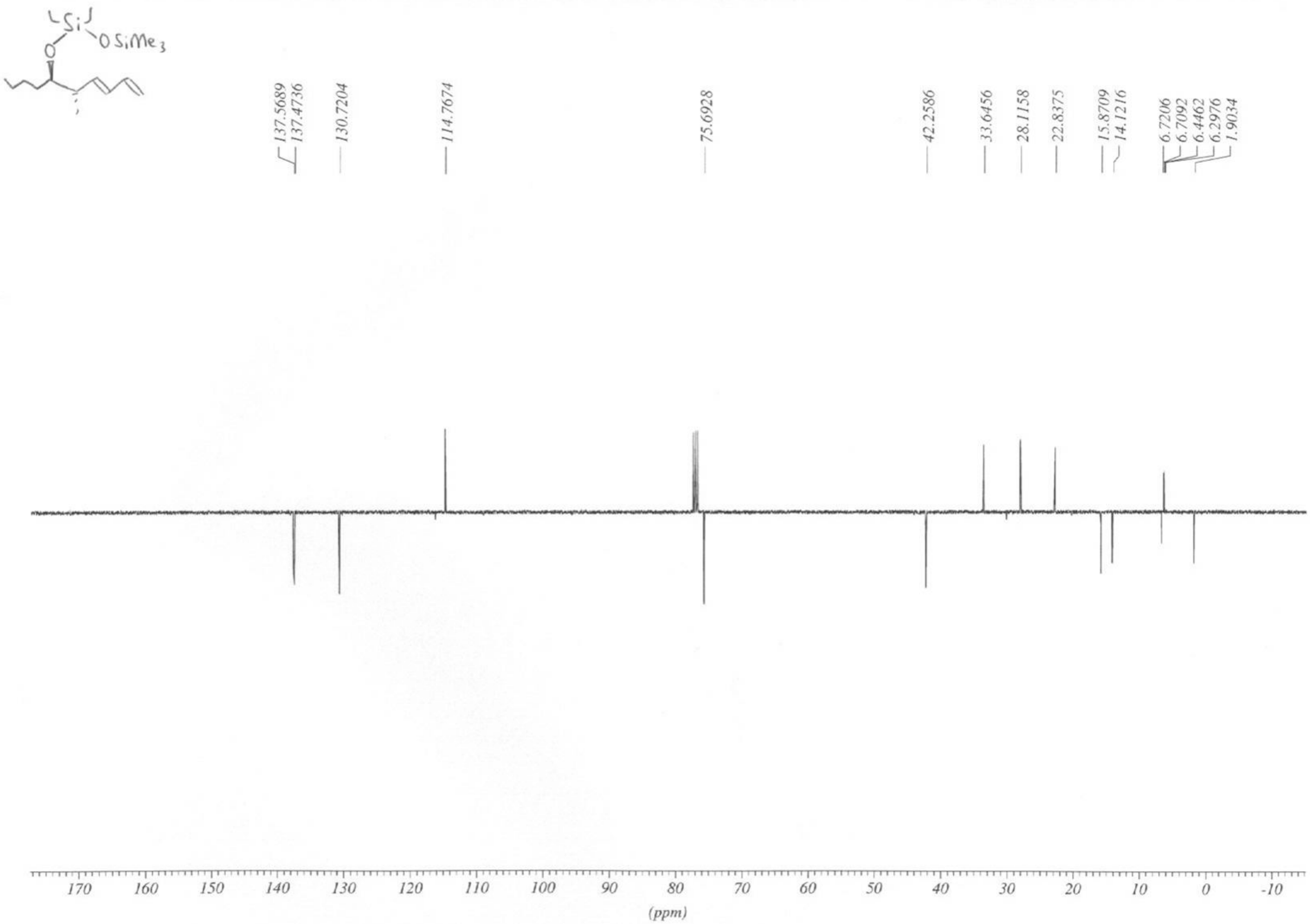

OSiMe3
137.5689
137.4736
130.7204
114.7674
75.6928
42.2586
33.6456
28.1158
22.8375
15.8709
14.1216
6.7206
6.7092
6.4462
6.2976
1.9034
170 160 150 140 130 120 110 100 90 80 70 60 50 40 30 20 10 0 -10
(ppm)

anti-13a
