# Supplementary material for: Tether-directed synthesis of highly substituted oxasilacycles via an intramolecular allylation employing allylsilanes
Source: Beilstein J Org Chem. 2007 Feb 8;3:6. doi: 10.1186/1860-5397-3-6 (PMC1810303; doi:10.1186/1860-5397-3-6)
Supplement: File 3 — 1H-NMR and 13C-NMR Spectra for the following compounds: 16a, 17a. [file Beilstein_J_Org_Chem-03-06-s003.pdf]

Additional File 3

Tether-directed synthesis of highly substituted oxasilacycles *via* an intramolecular allylation employing allylsilanes

Peter J. Jervis and Liam R. Cox*

email: l.r.cox@bham.ac.uk

^{1}H-NMR and ^{13}C-NMR Spectra for the following compounds:

16a, 17a

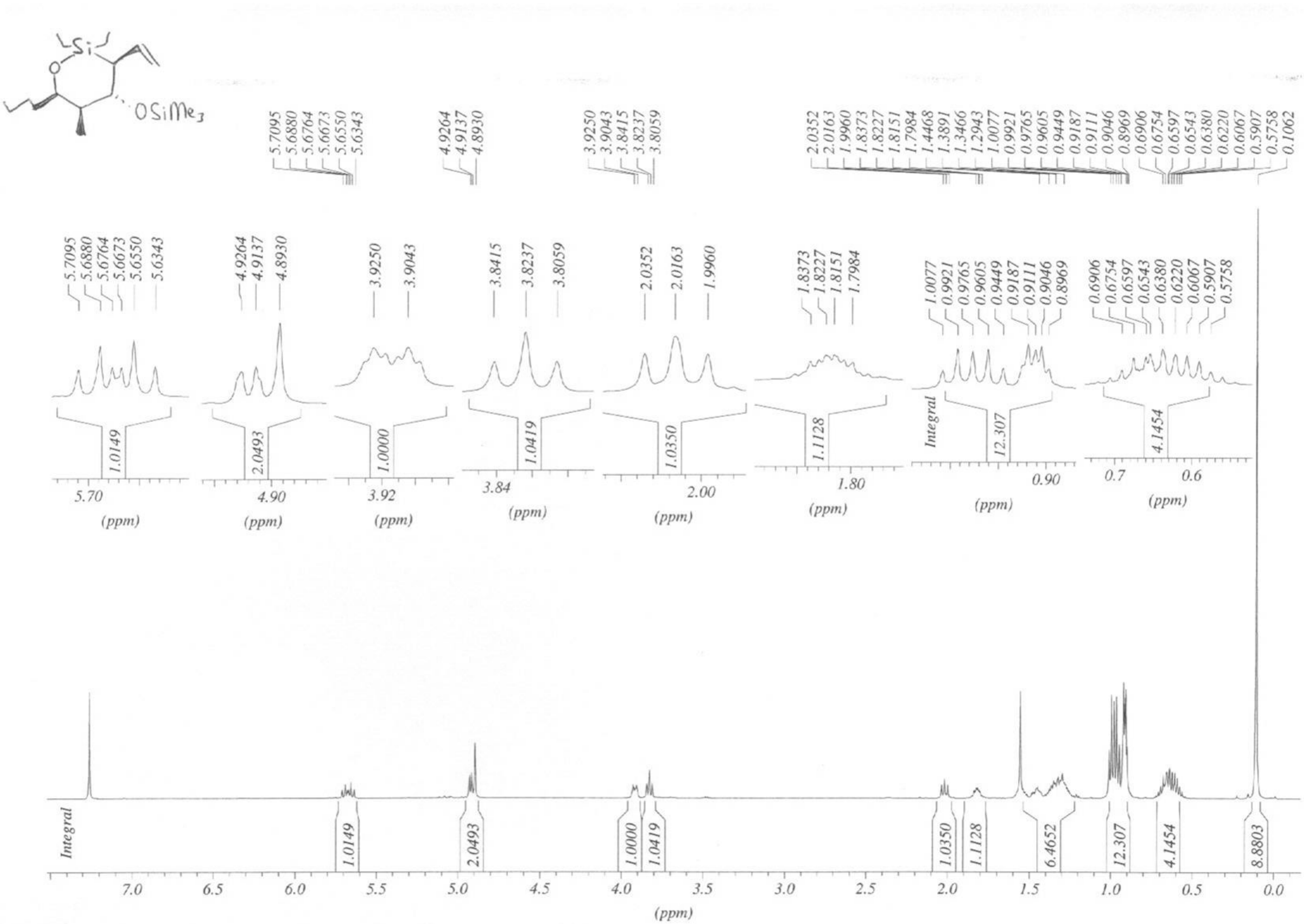
OSiMe3
Si
Integral
(ppm)
1.0149
2.0493
1.0000
1.0419
1.0350
1.1128
6.4652
12.307
4.1454
8.8803

16a

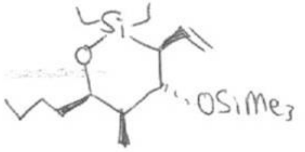
OSiMe3
Si
O

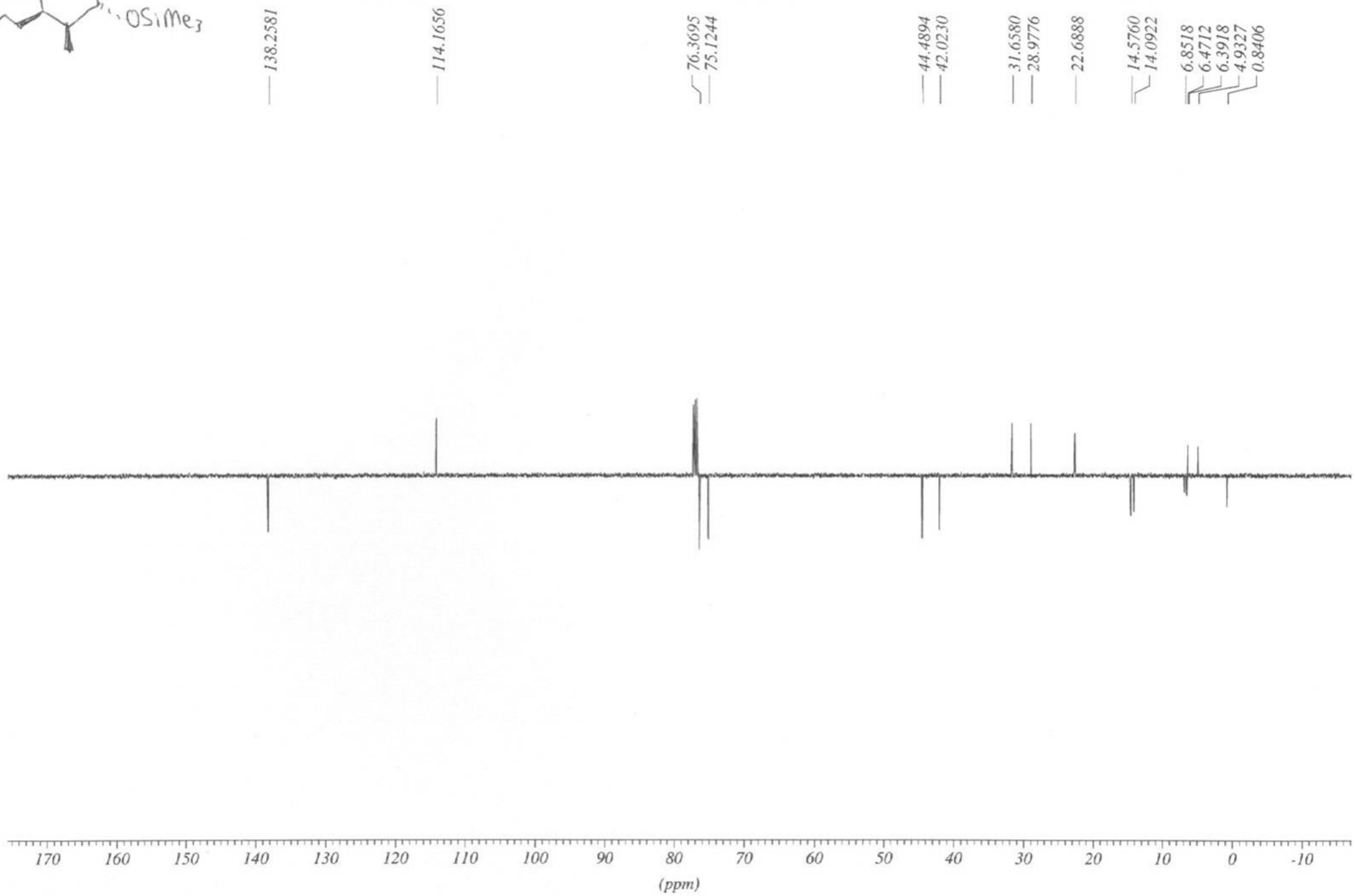
138.2581
114.1656
76.3695
75.1244
44.4894
42.0230
31.6580
28.9776
22.6888
14.5760
14.0922
6.8518
6.4712
6.3918
4.9327
0.8406
170 160 150 140 130 120 110 100 90 80 70 60 50 40 30 20 10 0 -10
(ppm)

16a

Peter Jervis Sample 2, 08/11/05 in CDCl3 at +27C, set temp
drx500, Gradient COSY90

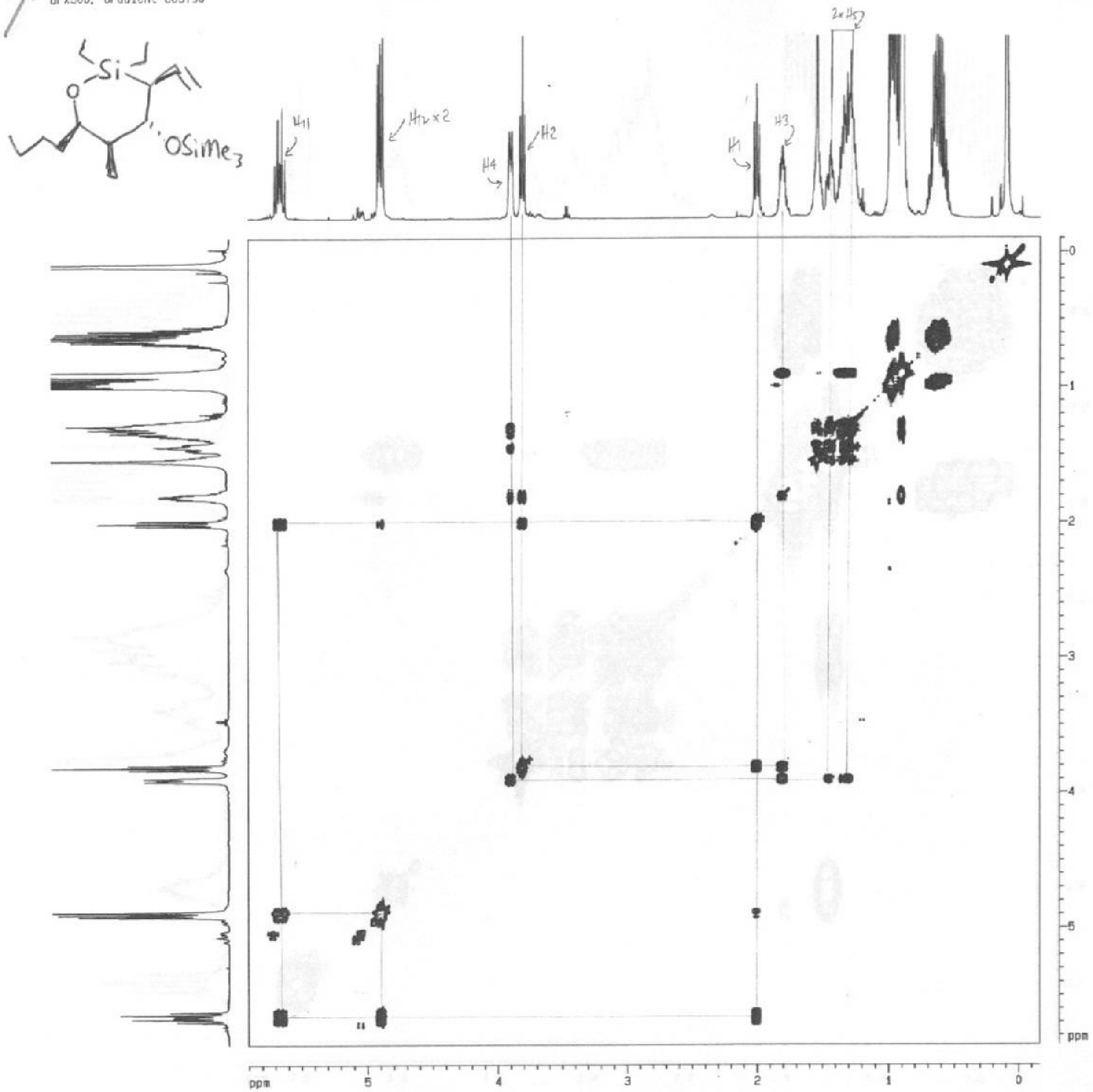

Current Data Parameters	
NAME	nv08pj1d
EXPNO	4
PROCNO	1

F2 - Acquisition Parameters	
Date_	20051108
Time	22.47
INSTRUM	drx500
PROBHD	5 mm TBI H/C
PULPROG	cosygp
TD	2048
SOLVENT	CDCl3
NS	8
DS	16
SWH	4310.345 Hz
FIDRES	2.104661 Hz
AQ	0.2376180 sec
RG	256
DW	116.000 usec
DE	5.50 usec
TE	300.0 K
d0	0.00000300 sec
D1	2.00000000 sec
d13	0.00000300 sec
D16	0.00010000 sec
IN0	0.00023200 sec

============ CHANNEL f1 ============	
NUC1	1H
P0	10.70 usec
P1	10.70 usec
PL1	1.00 dB
SFO1	500.1318867 MHz

============ GRADIENT CHANNEL ========	
GPNAM1	SINE.100
GPNAM2	SINE.100
GPX1	0.00 %
GPX2	0.00 %
GPY1	0.00 %
GPY2	0.00 %
GPZ1	10.00 %
GPZ2	10.00 %
P16	1000.00 usec

F1 - Acquisition parameters	
ND0	1
TD	512
SFO1	500.1319 MHz
FIDRES	8.418642 Hz
SW	8.618 ppm

F2 - Processing parameters	
SI	2048
SF	500.1300233 MHz
WDW	SINE
SSB	0
LB	0.00 Hz
GB	0
PC	1.00

F1 - Processing parameters	
SI	1024
MC2	QF
SF	500.1300233 MHz
WDW	SINE
SSB	0
LB	0.00 Hz
GB	0

2D NMR plot parameters	
CX2	20.00 cm
CX1	20.00 cm
F2PLO	5.906 ppm
F2LO	2953.67 Hz
F2PHI	-0.162 ppm
F2HI	-81.25 Hz
F1PLO	5.872 ppm
F1LO	2936.84 Hz
F1PHI	-0.104 ppm
F1HI	-51.78 Hz
F2PPMCM	0.30341 ppm/cm
F2HZCM	151.74603 Hz/cm
F1PPMCM	0.29878 ppm/cm
F1HZCM	149.43089 Hz/cm

16a

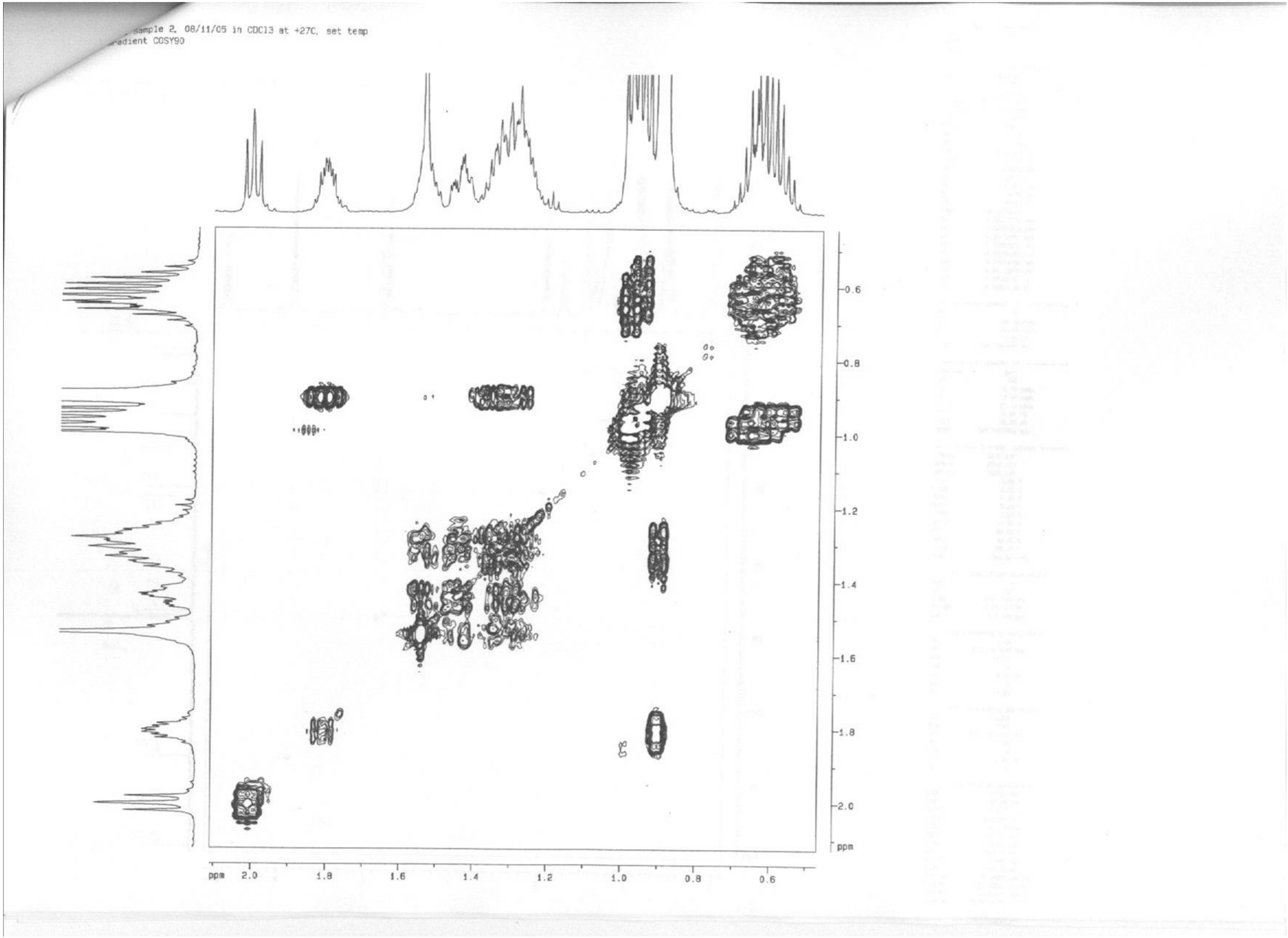
sample 2, 08/11/05 in CDCl3 at +27C, set temp
radient COSY90
ppm
2.0
1.8
1.6
1.4
1.2
1.0
0.8
0.6

16a

Peter Jervis Sample 2, 08/11/05 in CDCl3 at +27C, set temp
drx500, Gradient HSGC

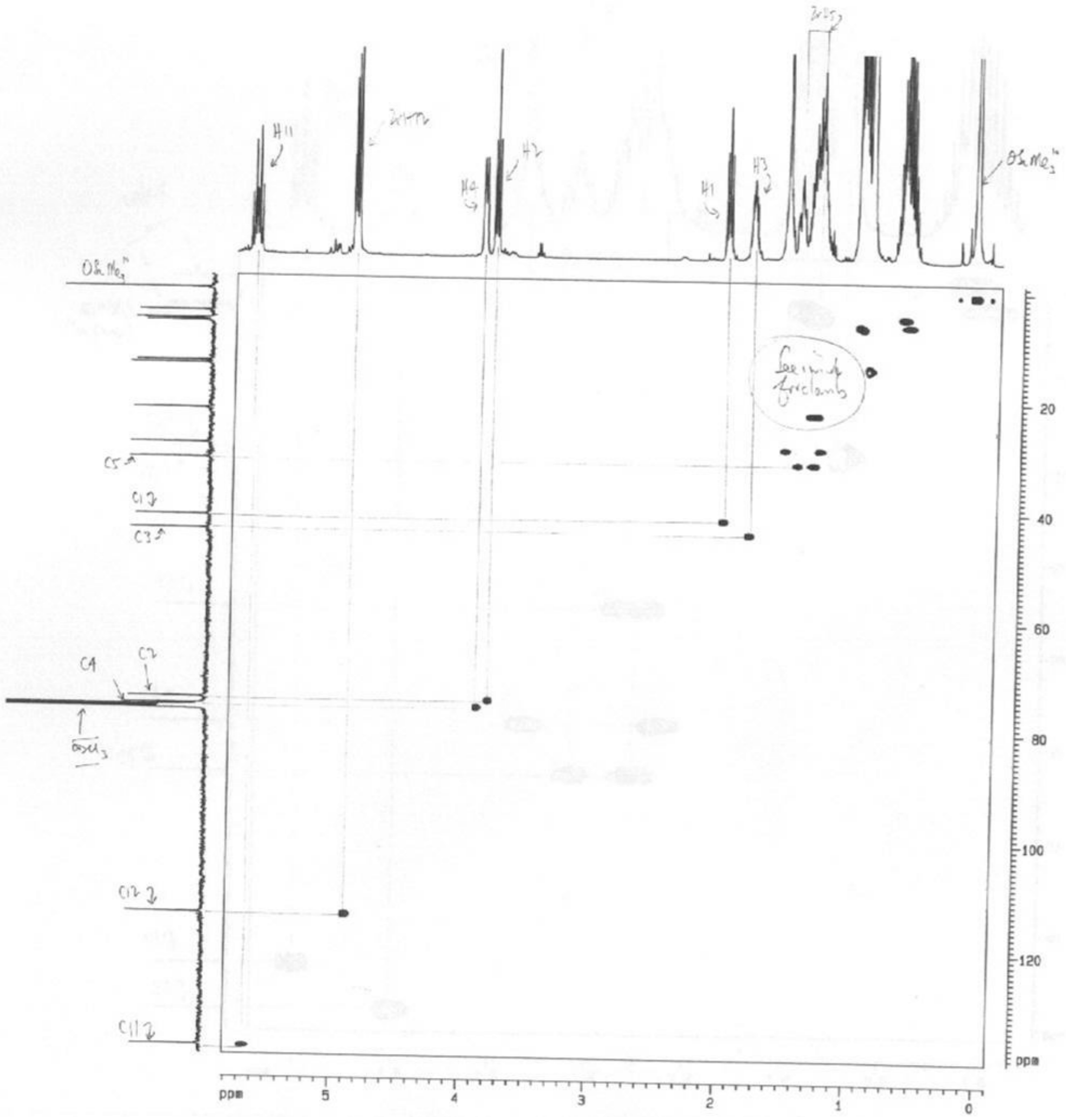

Current Data Parameters

NAME	nv08pj1d
EXPNO	2
PROCNO	1

F2 - Acquisition Parameters

Date_	20051108
Time	17.31
INSTRUM	drx500
PROBHD	5 mm TBI H/C
PULPROG	invigptp
TD	2048
SOLVENT	CDCl3
NS	8
DS	16
SWH	4310.345 Hz
FIDRES	2.104661 Hz
AQ	0.2376180 sec
RG	32768
DW	116.000 usec
DE	5.50 usec
TE	300.0 K
CNST2	145.0000000
d0	0.00000300 sec
D1	2.00000000 sec
d4	0.00172414 sec
d11	0.03000000 sec
d13	0.00000300 sec
D16	0.00010000 sec
d20	0.00110000 sec
d21	0.00081714 sec
IN0	0.00001140 sec

======== CHANNEL f1 ========

NUC1	1H
P1	10.70 usec
p2	21.40 usec
PL1	1.00 dB
SFO1	500.1318887 MHz

======== CHANNEL f2 ========

CPDPRG2	garp
NUC2	13C
P3	12.00 usec
p4	24.00 usec
PCPD2	78.00 usec
PL2	-1.00 dB
PL12	15.00 dB
SFO2	125.7667893 MHz

======== GRADIENT CHANNEL ========

GPNAM1	SINE.100
GPNAM2	SINE.100
GPNAM3	SINE.100
GPX1	0.00 %
GPX2	0.00 %
GPX3	0.00 %
GPY1	0.00 %
GPY2	0.00 %
GPY3	0.00 %
GPZ1	80.00 %
GPZ2	30.00 %
GPZ3	20.10 %
P16	1000.00 usec

F1 - Acquisition parameters

ND0	4
TD	512
SFO1	125.7688 MHz
FIDRES	42.831698 Hz
SW	174.369 ppm

F2 - Processing parameters

SI	2048
SF	500.1300233 MHz
WDW	QSINE
SSB	2
LB	0.00 Hz
GB	0
PC	1.00

F1 - Processing parameters

SI	1024
MC2	TPPI
SF	125.7577910 MHz
WDW	QSINE
SSB	2
LB	0.00 Hz
GB	0

2D NMR plot parameters

CX2	17.00 cm
CX1	17.00 cm
F2PLO	5.813 ppm
F2LO	2907.37 Hz
F2PHI	-0.104 ppm
F2HI	-51.78 Hz
F1PLO	139.841 ppm
F1LO	17586.09 Hz
F1PHI	-1.503 ppm
F1HI	-189.06 Hz
F2PPMCM	0.34805 ppm/cm
F2HZCM	174.06781 Hz/cm
F1PPMCM	8.31437 ppm/cm
F1HZCM	[illegible] Hz/cm

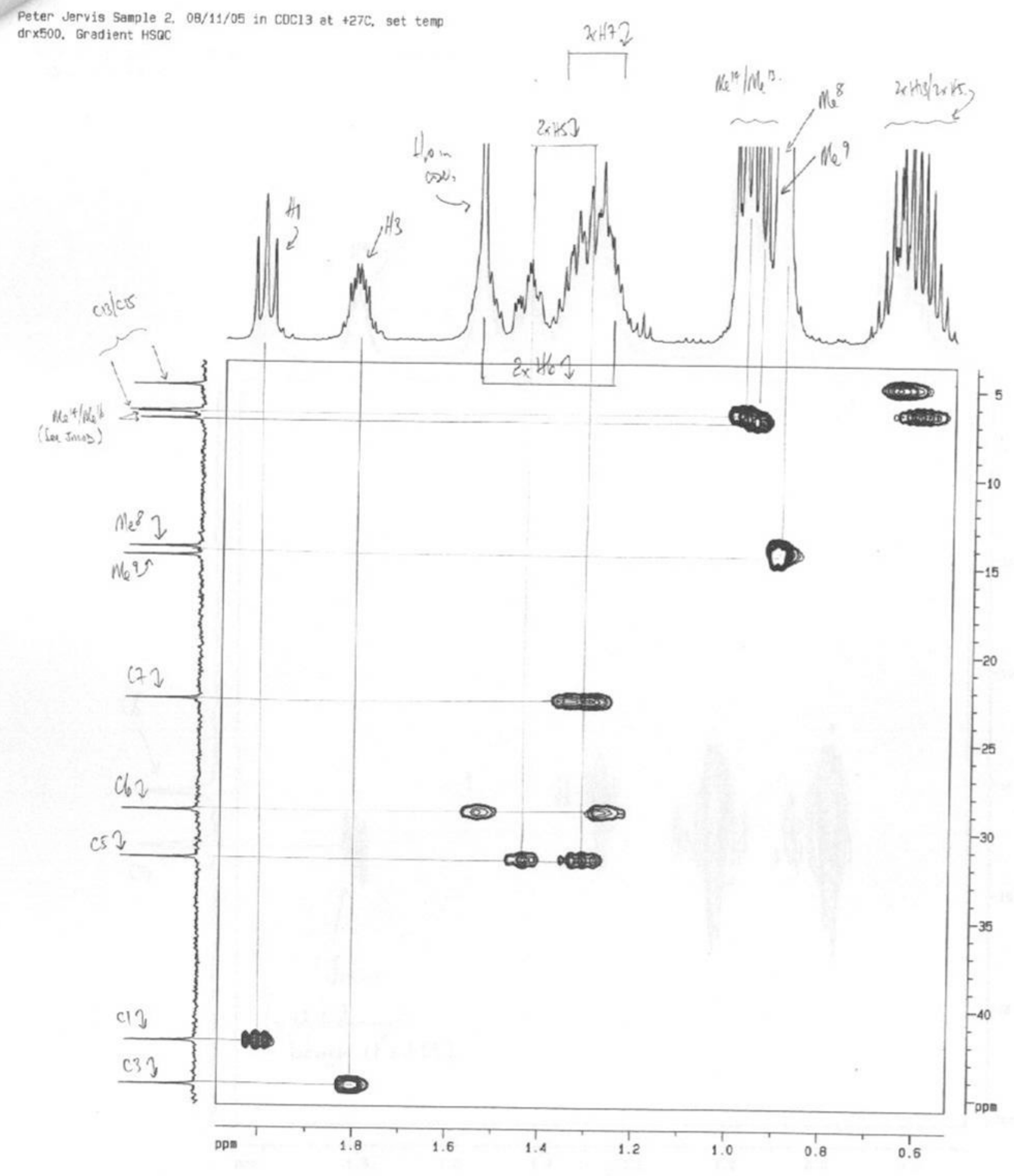
Peter Jervis Sample 2, 08/11/05 in CDCl3 at +27C, set temp
drx500, Gradient HSQC
H1
H3
2xH7
2xH5
2xH6
Me8
Me9
C7
C6
C5
C1
C3
ppm
1.8
1.6
1.4
1.2
1.0
0.8
0.6
5
10
15
20
25
30
35
40

Peter Jervis Sample 2, 08/11/05 in CDCl3 at +27C, set temp
drx500, Gradient HMBC

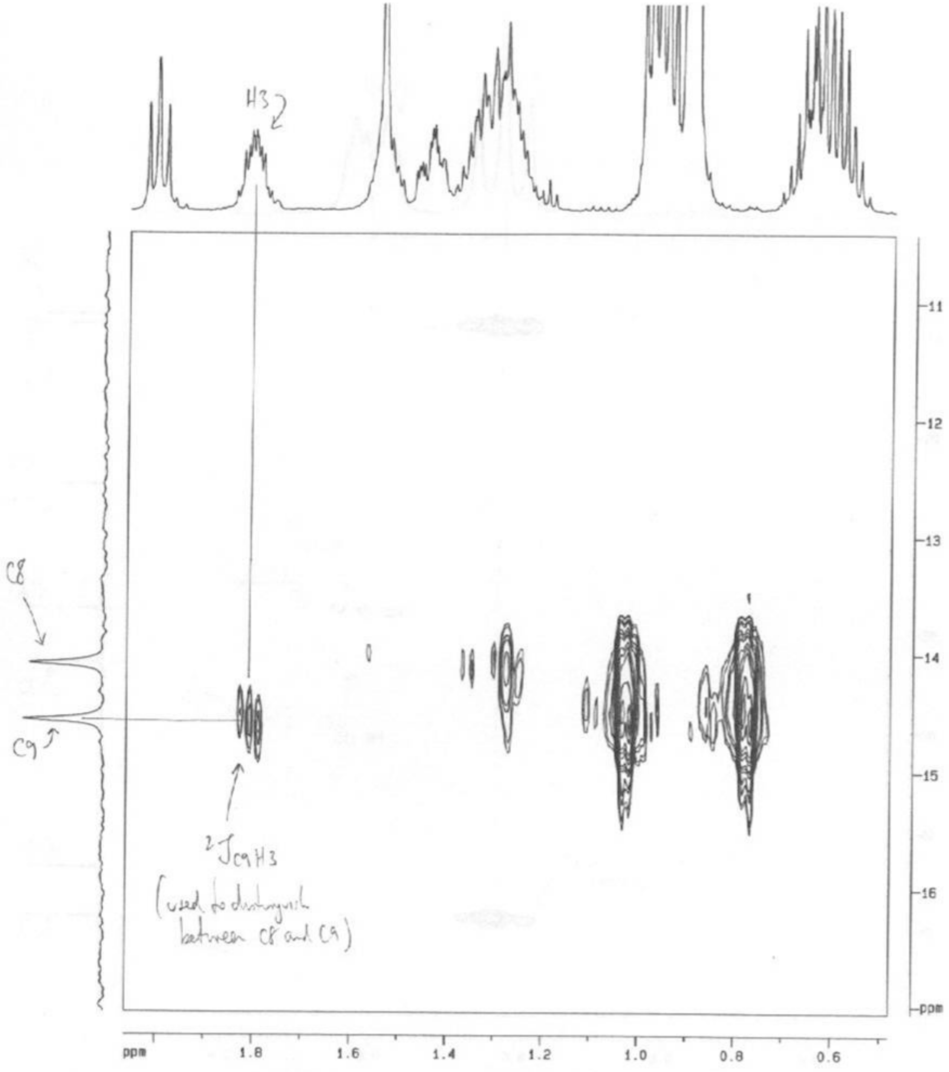

Current Data Parameters

NAME	nv08pj1d
EXPNO	3
PROCNO	1

F2 - Acquisition Parameters

Date_	20051108
Time	20.05
INSTRUM	drx500
PROBHD	5 mm TBI H/C
PULPROG	inv4gplplrnd
TD	2048
SOLVENT	CDCl3
NS	8
DS	16
SWH	4310.345 Hz
FIDRES	2.104661 Hz
AQ	0.2376180 sec
RG	32768
DW	116.000 usec
DE	5.50 usec
TE	300.0 K
CNST2	160.0000000
d0	0.00000300 sec
D1	2.00000000 sec
d2	0.00312500 sec
D6	0.10000000 sec
d13	0.00000300 sec
D16	0.00010000 sec
IN0	0.00002280 sec

============ CHANNEL f1 ============

NUC1	1H
P1	10.70 usec
p2	21.40 usec
PL1	1.00 dB
SFO1	500.1318867 MHz

============ CHANNEL f2 ============

NUC2	13C
P3	12.00 usec
PL2	-1.00 dB
SFO2	125.7667893 MHz

============ GRADIENT CHANNEL ========

GPNAM1	SINE.100
GPNAM2	SINE.100
GPNAM3	SINE.100
GPX1	0.00 %
GPX2	0.00 %
GPX3	0.00 %
GPY1	0.00 %
GPY2	0.00 %
GPY3	0.00 %
GPZ1	50.00 %
GPZ2	30.00 %
GPZ3	40.10 %
P16	1000.00 usec

F1 - Acquisition parameters

ND0	2
TD	512
SFO1	125.7668 MHz
FIDRES	42.831688 Hz
SW	174.369 ppm

F2 - Processing parameters

SI	2048
SF	500.1300233 MHz
WDW	QSINE
SSB	2
LB	0.00 Hz
GB	0
PC	0.20

F1 - Processing parameters

SI	1024
MC2	QF
SF	125.7577910 MHz
WDW	QSINE
SSB	2
LB	0.00 Hz
GB	0

2D NMR plot parameters

CX2	17.00 cm
CX1	17.00 cm
F2PLO	2.064 ppm
F2LO	1032.12 Hz
F2PHI	0.481 ppm
F2HI	240.77 Hz
F1PLO	17.059 ppm
F1LO	2145.26 Hz
F1PHI	10.417 ppm
F1HI	1310.04 Hz
F2PPMCM	0.09308 ppm/cm
F2HZCM	46.55014 Hz/cm
F1PPMCM	0.39068 ppm/cm
F1HZCM	49.13047 Hz/cm

16a

Peter Jervis Sample 2, 08/11/05 in CDCl3 at +27C, set temp
drx500, Gradient HMBC

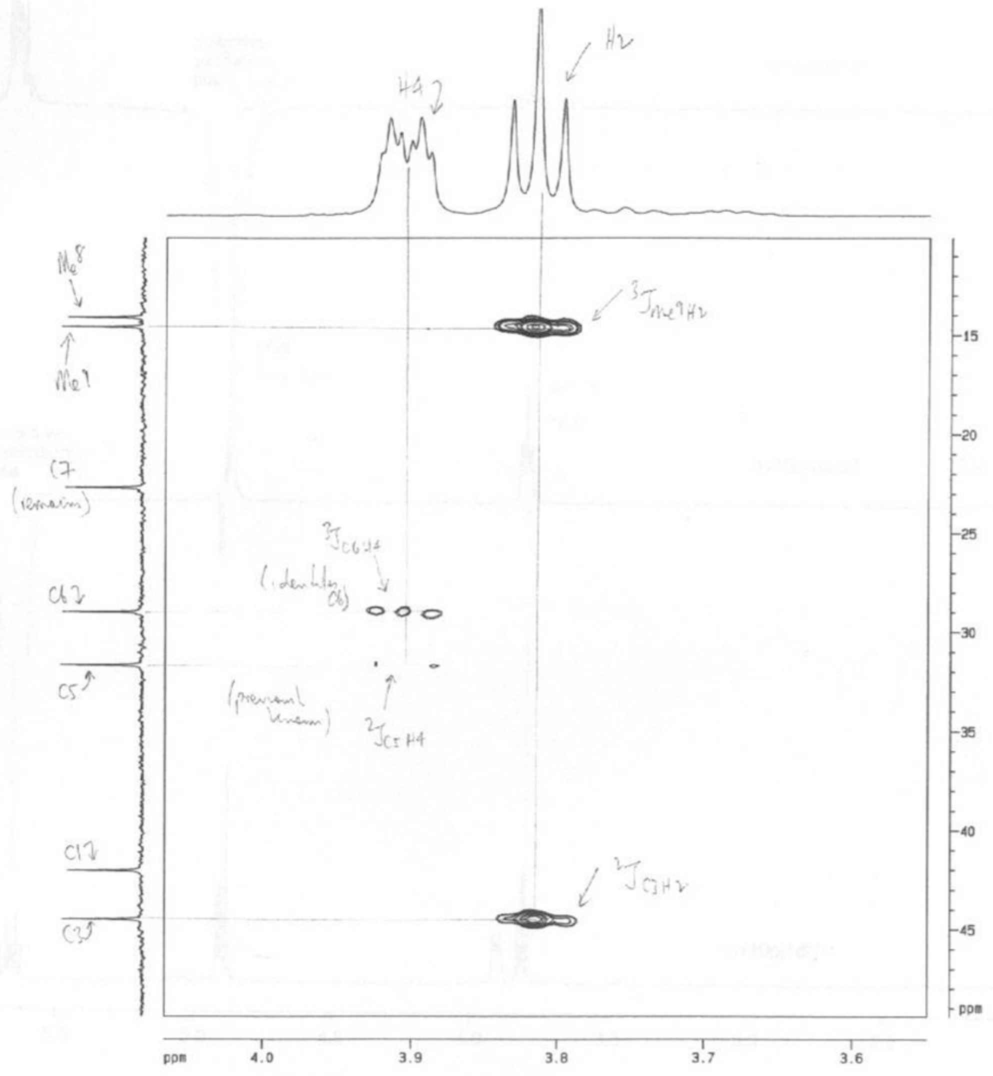

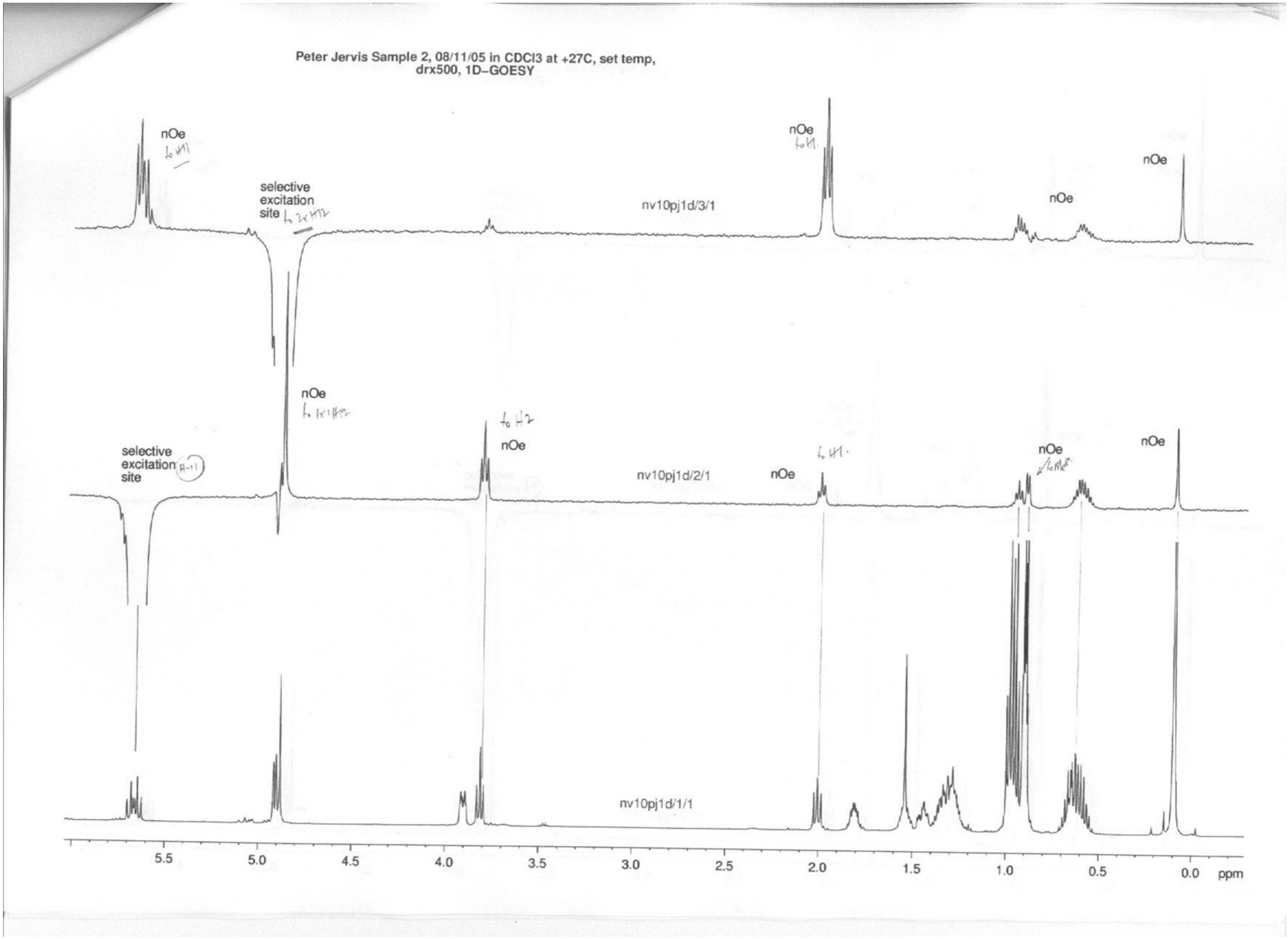
Peter Jervis Sample 2, 08/11/05 in CDCl3 at +27C, set temp,
drx500, 1D−GOESY
nOe
selective
excitation
site
nv10pj1d/3/1
nOe
nOe
nOe
nOe
selective
excitation
site
nOe
nv10pj1d/2/1
nOe
nOe
nOe
nv10pj1d/1/1
5.5
5.0
4.5
4.0
3.5
3.0
2.5
2.0
1.5
1.0
0.5
0.0
ppm

16a

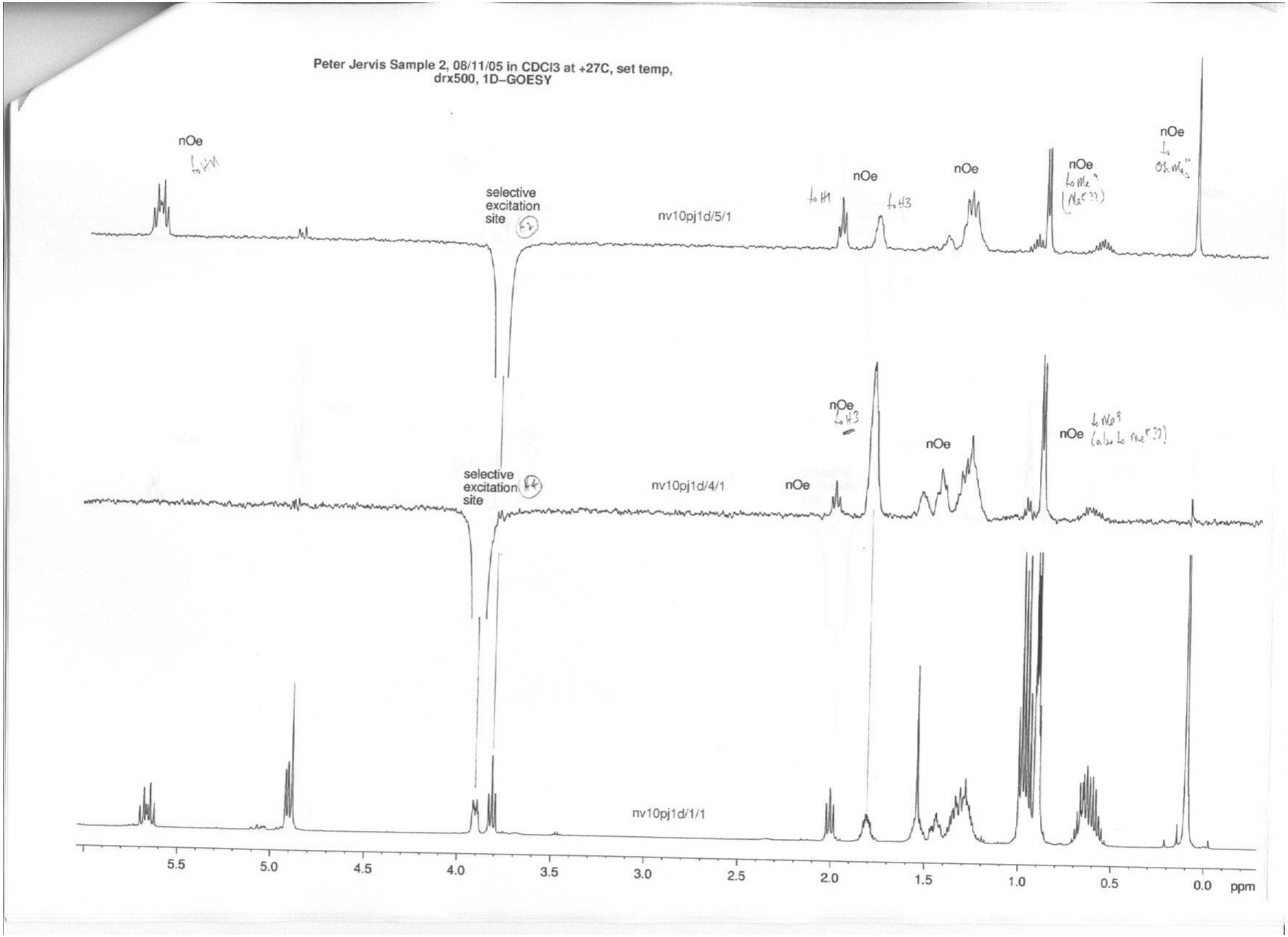

Peter Jervis Sample 2, 08/11/05 in CDCl3 at +27C, set temp,
drx500, 1D–GOESY
nOe
selective
excitation
site
nv10pj1d/5/1
nOe
nOe
nOe
nOe
selective
excitation
site
nv10pj1d/4/1
nOe
nOe
nOe
nOe
nv10pj1d/1/1
5.5
5.0
4.5
4.0
3.5
3.0
2.5
2.0
1.5
1.0
0.5
0.0
ppm

16a

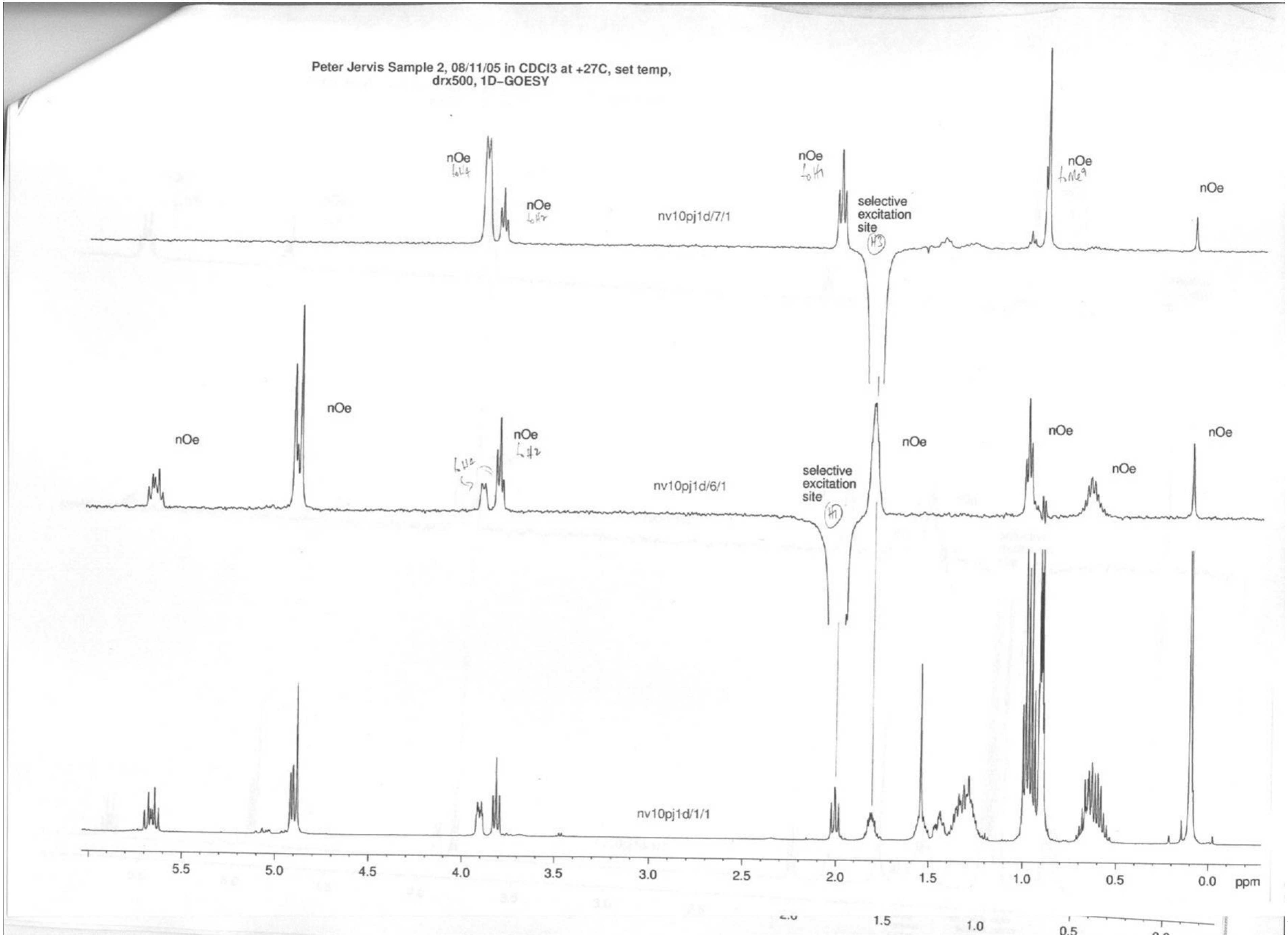
Peter Jervis Sample 2, 08/11/05 in CDCl3 at +27C, set temp,
drx500, 1D-GOESY
nOe
nOe
nOe
selective excitation site
nOe
nOe
nv10pj1d/7/1
nOe
nOe
nOe
nOe
selective excitation site
nOe
nOe
nOe
nv10pj1d/6/1
nv10pj1d/1/1
5.5
5.0
4.5
4.0
3.5
3.0
2.5
2.0
1.5
1.0
0.5
0.0
ppm

16a

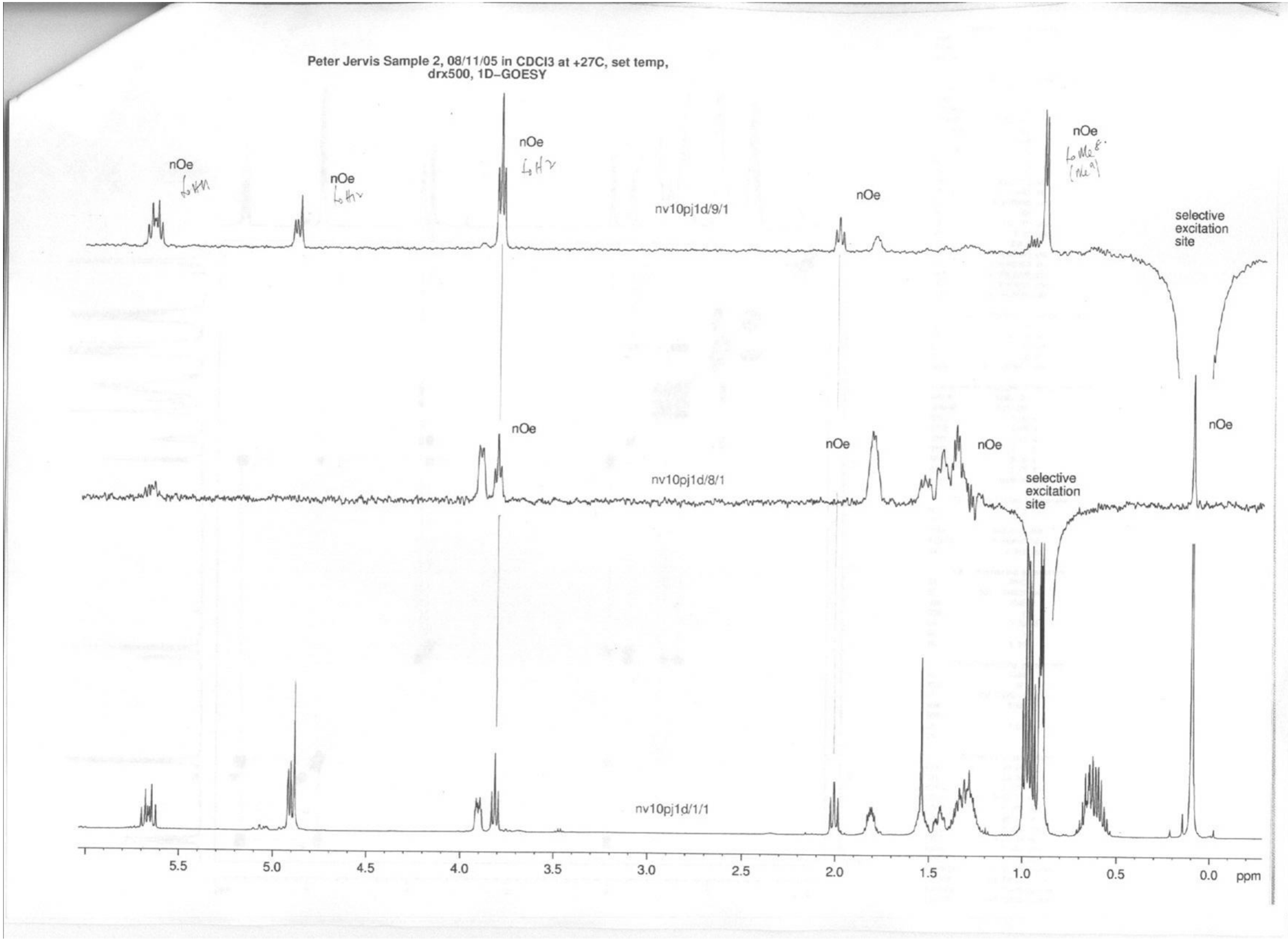
Peter Jervis Sample 2, 08/11/05 in CDCl3 at +27C, set temp,
drx500, 1D-GOESY
nOe
nOe
nOe
nOe
nOe
nv10pj1d/9/1
selective
excitation
site
nOe
nOe
nOe
nOe
nv10pj1d/8/1
selective
excitation
site
nv10pj1d/1/1
5.5
5.0
4.5
4.0
3.5
3.0
2.5
2.0
1.5
1.0
0.5
0.0
ppm

16a

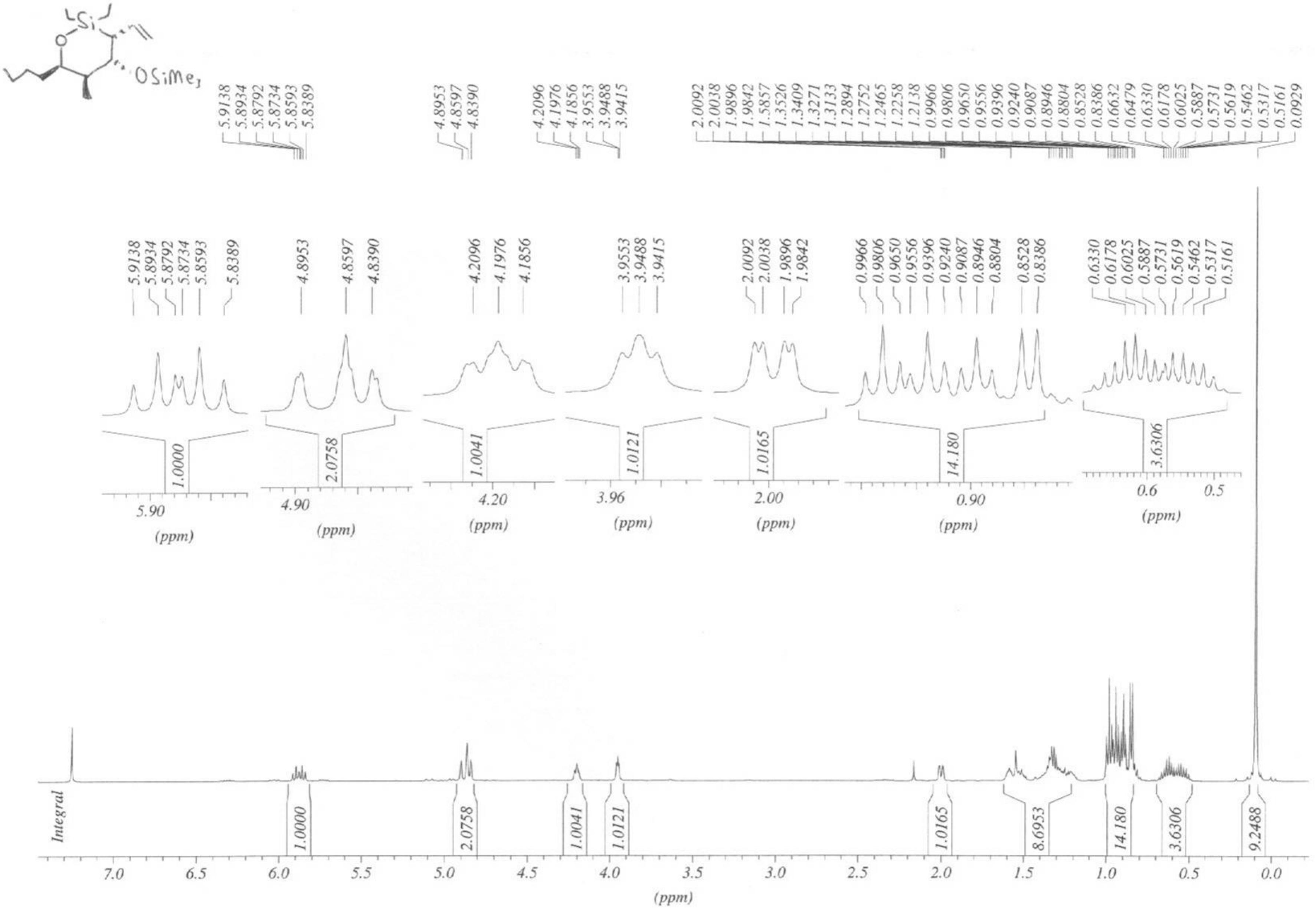

17a

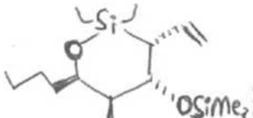
Si
O
OSiMe3

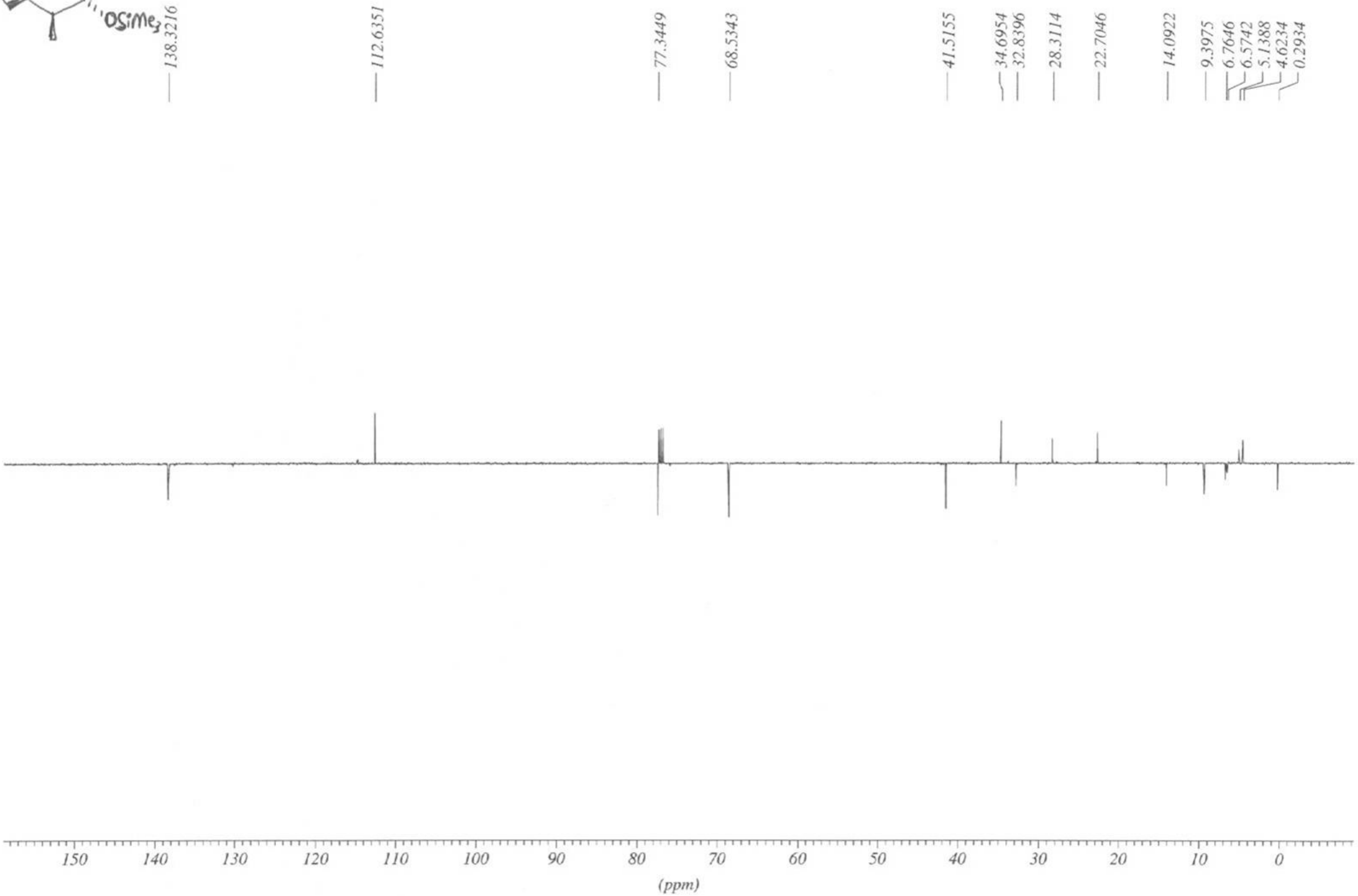
138.3216
112.6351
77.3449
68.5343
41.5155
34.6954
32.8396
28.3114
22.7046
14.0922
9.3975
6.7646
6.5742
5.1388
4.6234
0.2934
150
140
130
120
110
100
90
80
70
60
50
40
30
20
10
0
(ppm)

17a

Peter Jervis Sample 4, 14/11/05 in CDCl3 at +27C, set temp
drx500, Gradient COSY90

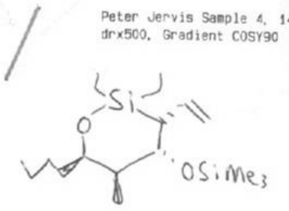

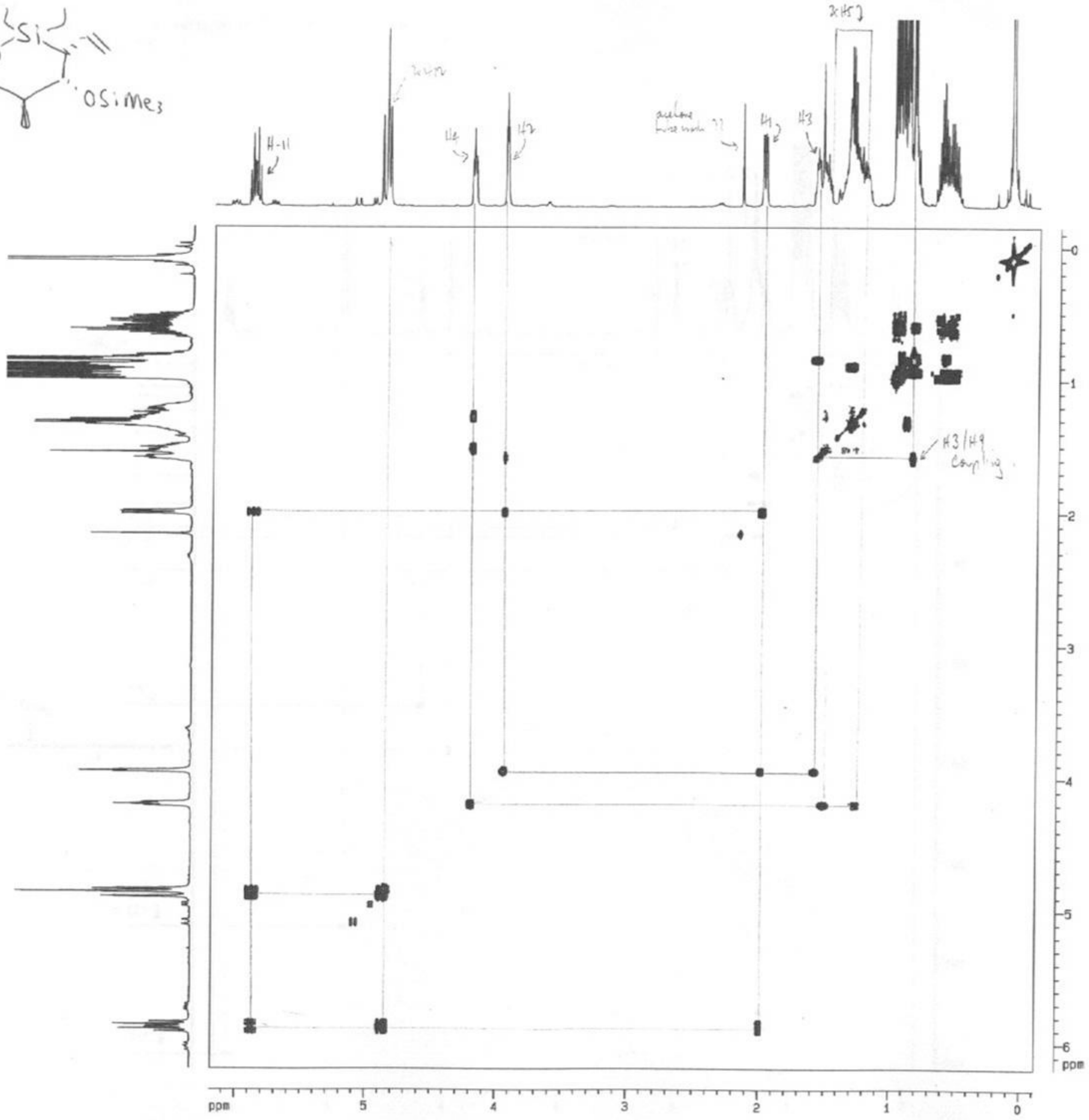

Current Data Parameters

NAME	nv14pj1d
EXPNO	4
PROCNO	1

F2 - Acquisition Parameters

Date_	20051114
Time	21.08
INSTRUM	drx500
PROBHD	5 mm TBI H/C
PULPROG	cosygp
TD	2048
SOLVENT	CDCl3
NS	8
DS	16
SWH	4310.345 Hz
FIDRES	2.104661 Hz
AQ	0.2376180 sec
RG	128
DW	116.000 usec
DE	5.50 usec
TE	300.0 K
d0	0.00000300 sec
D1	2.00000000 sec
d13	0.00000300 sec
D16	0.00010000 sec
IN0	0.00023200 sec

============ CHANNEL f1 ============

NUC1	1H
P0	10.70 usec
P1	10.70 usec
PL1	1.00 dB
SFO1	500.1318867 MHz

============ GRADIENT CHANNEL ========

GPNAM1	SINE.100
GPNAM2	SINE.100
GPX1	0.00 %
GPX2	0.00 %
GPY1	0.00 %
GPY2	0.00 %
GPZ1	10.00 %
GPZ2	10.00 %
P16	1000.00 usec

F1 - Acquisition parameters

ND0	1
TD	512
SFO1	500.1319 MHz
FIDRES	8.418642 Hz
SW	8.618 ppm

F2 - Processing parameters

SI	2048
SF	500.1300233 MHz
WDW	SINE
SSB	0
LB	0.00 Hz
GB	0
PC	1.00

F1 - Processing parameters

SI	1024
MC2	QF
SF	500.1300233 MHz
WDW	SINE
SSB	0
LB	0.00 Hz
GB	0

2D NMR plot parameters

CX2	20.00 cm
CX1	20.00 cm
F2PLO	6.188 ppm
F2LO	3094.69 Hz
F2PHI	-0.116 ppm
F2HI	-58.10 Hz
F1PLO	6.192 ppm
F1LO	3096.79 Hz
F1PHI	-0.154 ppm
F1HI	-77.04 Hz
F2PPMCM	0.31520 ppm/cm
F2HZCM	157.63908 Hz/cm
F1PPMCM	0.31730 ppm/cm
F1HZCM	158.69141 Hz/cm

Peter Jervis Sample 4, 14/11/05 in CDCl3 at +27C, set temp
drx500, Gradient HSQC

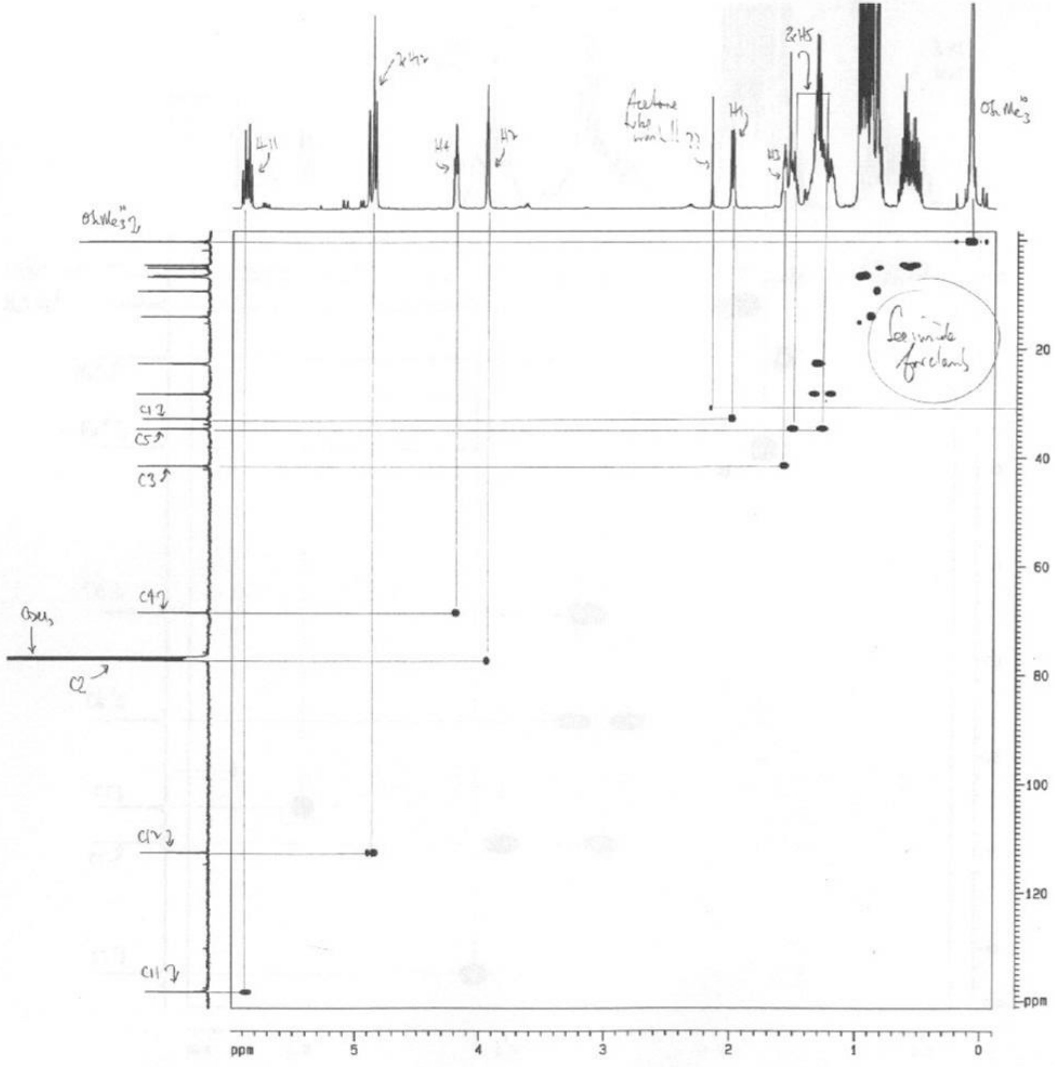

Current Data Parameters

NAME	nv14pjid
EXPNO	2
PROCNO	1

F2 - Acquisition Parameters

Date_	20051114
Time	15.52
INSTRUM	drx500
PROBHD	5 mm TBI H/C
PULPROG	invigptp
TD	2048
SOLVENT	CDCl3
NS	8
DS	16
SWH	4310.345 Hz
FIDRES	2.104661 Hz
AQ	0.2376180 sec
RG	32768
DW	116.000 usec
DE	6.50 usec
TE	300.0 K
CNST2	145.0000000
d0	0.00000300 sec
D1	2.00000000 sec
d4	0.00172414 sec
d11	0.03000000 sec
d13	0.00000300 sec
D16	0.00010000 sec
d20	0.00110000 sec
d21	0.00061714 sec
IN0	0.00001140 sec

============ CHANNEL f1 ============

NUC1	1H
P1	10.70 usec
p2	21.40 usec
PL1	1.00 dB
SFO1	500.1318867 MHz

============ CHANNEL f2 ============

CPDPRG2	garp
NUC2	13C
P3	12.00 usec
p4	24.00 usec
PCPD2	76.00 usec
PL2	-1.00 dB
PL12	15.00 dB
SFO2	125.7667893 MHz

============ GRADIENT CHANNEL ============

GPNAM1	SINE.100
GPNAM2	SINE.100
GPNAM3	SINE.100
GPX1	0.00 %
GPX2	0.00 %
GPX3	0.00 %
GPY1	0.00 %
GPY2	0.00 %
GPY3	0.00 %
GPZ1	80.00 %
GPZ2	30.00 %
GPZ3	20.10 %
P16	1000.00 usec

F1 - Acquisition parameters

ND0	4
TD	512
SFO1	125.7668 MHz
FIDRES	42.831688 Hz
SW	174.369 ppm

F2 - Processing parameters

SI	2048
SF	500.1300233 MHz
WDW	QSINE
SSB	2
LB	0.00 Hz
GB	0
PC	1.00

F1 - Processing parameters

SI	1024
MC2	TPPI
SF	125.7577910 MHz
WDW	QSINE
SSB	2
LB	0.00 Hz
GB	0

2D NMR plot parameters

CX2	17.00 cm
CX1	17.00 cm
F2PLO	5.890 ppm
F2LO	2995.77 Hz
F2PHI	-0.104 ppm
F2HI	-51.78 Hz
F1PLO	141.374 ppm
F1LO	17778.83 Hz
F1PHI	-1.674 ppm
F1HI	-210.47 Hz
F2PPMCM	0.35844 ppm/cm
F2HZCM	179.26756 Hz/cm
F1PPMCM	8.41455 ppm/cm
F1HZCM	1058.19456 Hz/cm

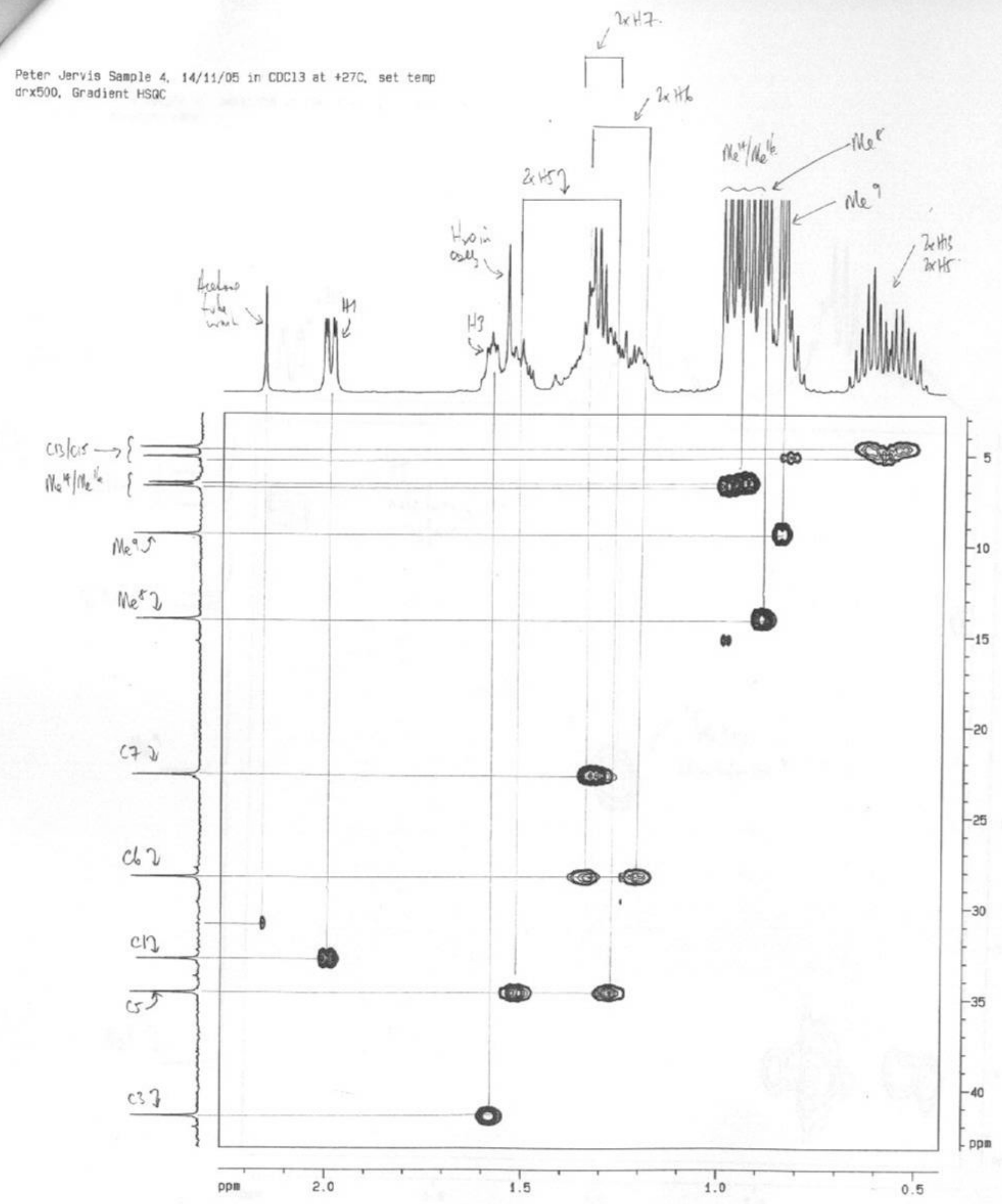

Peter Jervis Sample 4, 14/11/05 in CDCl3 at +27C, set temp
drx500, Gradient HSQC
2xH7
2xH6
2xH5
Me8
Me9
H1
H3
C7
C6
C1
C5
C3
ppm
2.0
1.5
1.0
0.5
5
10
15
20
25
30
35
40

Peter Jervis Sample 4, 14/11/05 in CDCl3 at +27C, set temp
drx500, Gradient HMBC

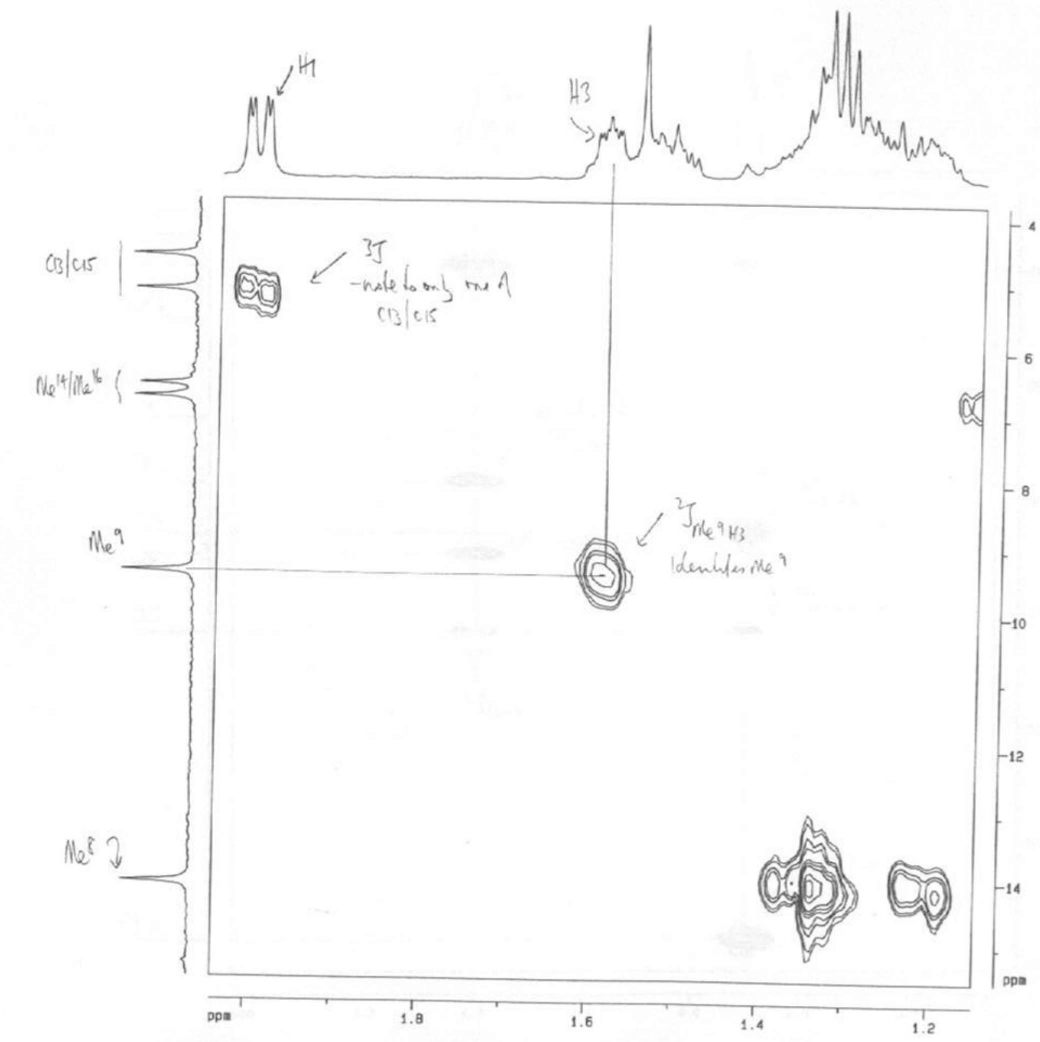

Current Data Parameters

NAME	nvi4pj1d
EXPNO	3
PROCNO	1

F2 - Acquisition Parameters

Date_	20051114
Time	18.26
INSTRUM	drx500
PROBHD	5 mm TBI H/C
PULPROG	inv4gplplrnd
TD	2048
SOLVENT	CDCl3
NS	8
DS	16
SWH	4310.345 Hz
FIDRES	2.104661 Hz
AQ	0.2376180 sec
RG	32768
DW	116.000 usec
DE	5.50 usec
TE	300.0 K
CNST2	160.0000000
d0	0.00000300 sec
D1	2.00000000 sec
d2	0.00312500 sec
D6	0.10000000 sec
d13	0.00000300 sec
D16	0.00010000 sec
IN0	0.00002280 sec

======== CHANNEL f1 ========

NUC1	1H
P1	10.70 usec
p2	21.40 usec
PL1	1.00 dB
SFO1	500.1318867 MHz

======== CHANNEL f2 ========

NUC2	13C
P3	12.00 usec
PL2	-1.00 dB
SFO2	125.7667893 MHz

======== GRADIENT CHANNEL ========

GPNAM1	SINE.100
GPNAM2	SINE.100
GPNAM3	SINE.100
GPX1	0.00 %
GPX2	0.00 %
GPX3	0.00 %
GPY1	0.00 %
GPY2	0.00 %
GPY3	0.00 %
GPZ1	50.00 %
GPZ2	30.00 %
GPZ3	40.10 %
P16	1000.00 usec

F1 - Acquisition parameters

ND0	2
TD	512
SFO1	125.7668 MHz
FIDRES	42.831688 Hz
SW	174.369 ppm

F2 - Processing parameters

SI	2048
SF	500.1300233 MHz
WDW	QSINE
SSB	2
LB	0.00 Hz
GB	0
PC	0.20

F1 - Processing parameters

SI	1024
MC2	QF
SF	125.7577910 MHz
WDW	QSINE
SSB	2
LB	0.00 Hz
GB	0

2D NMR plot parameters

CX2	17.00 cm
CX1	17.00 cm
F2PLO	2.038 ppm
F2LO	1019.49 Hz
F2PHI	1.146 ppm
F2HI	573.30 Hz
F1PLO	15.526 ppm
F1LO	1952.52 Hz
F1PHI	3.776 ppm
F1HI	474.83 Hz
F2PPMCM	0.05248 ppm/cm
F2HZCM	26.24636 Hz/cm
F1PPMCM	0.69119 ppm/cm
F1HZCM	[illegible]

Peter Jervis Sample 4, 14/11/05 in CDCl3 at +27C, set temp
drx500, Gradient HMBC

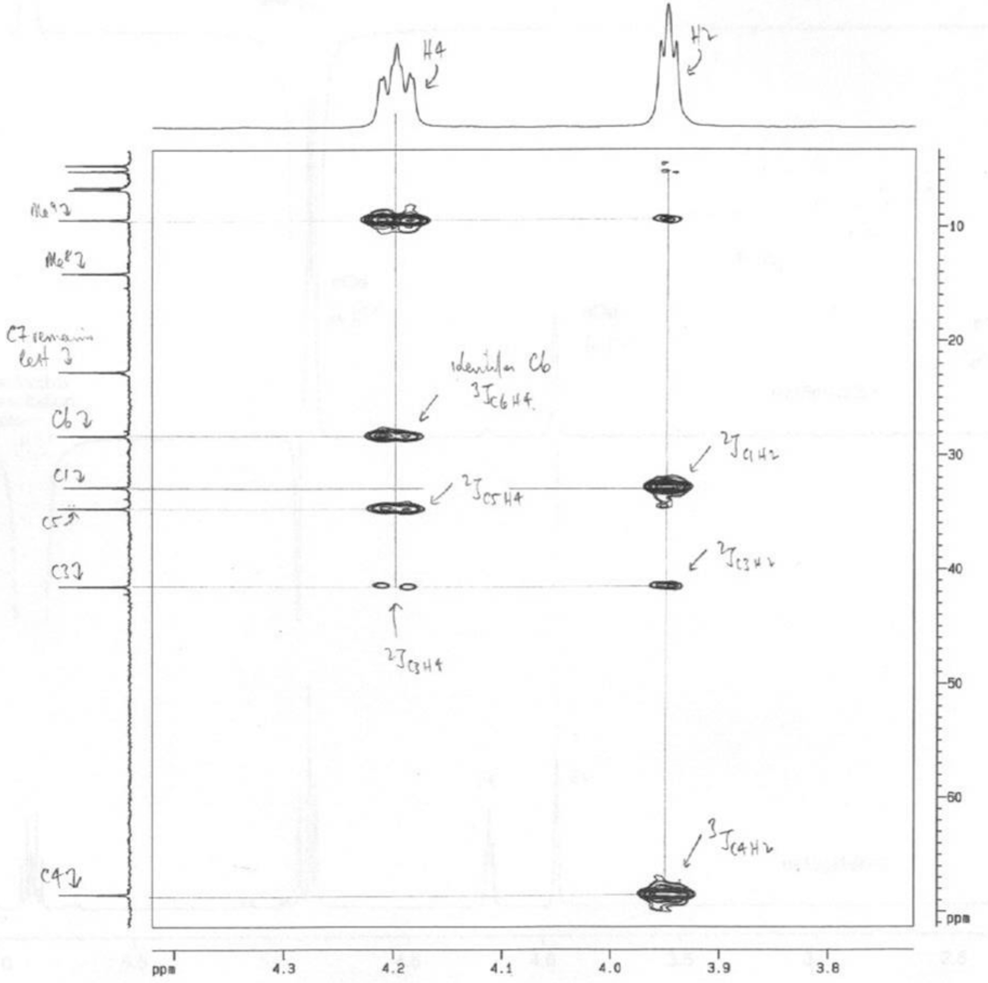

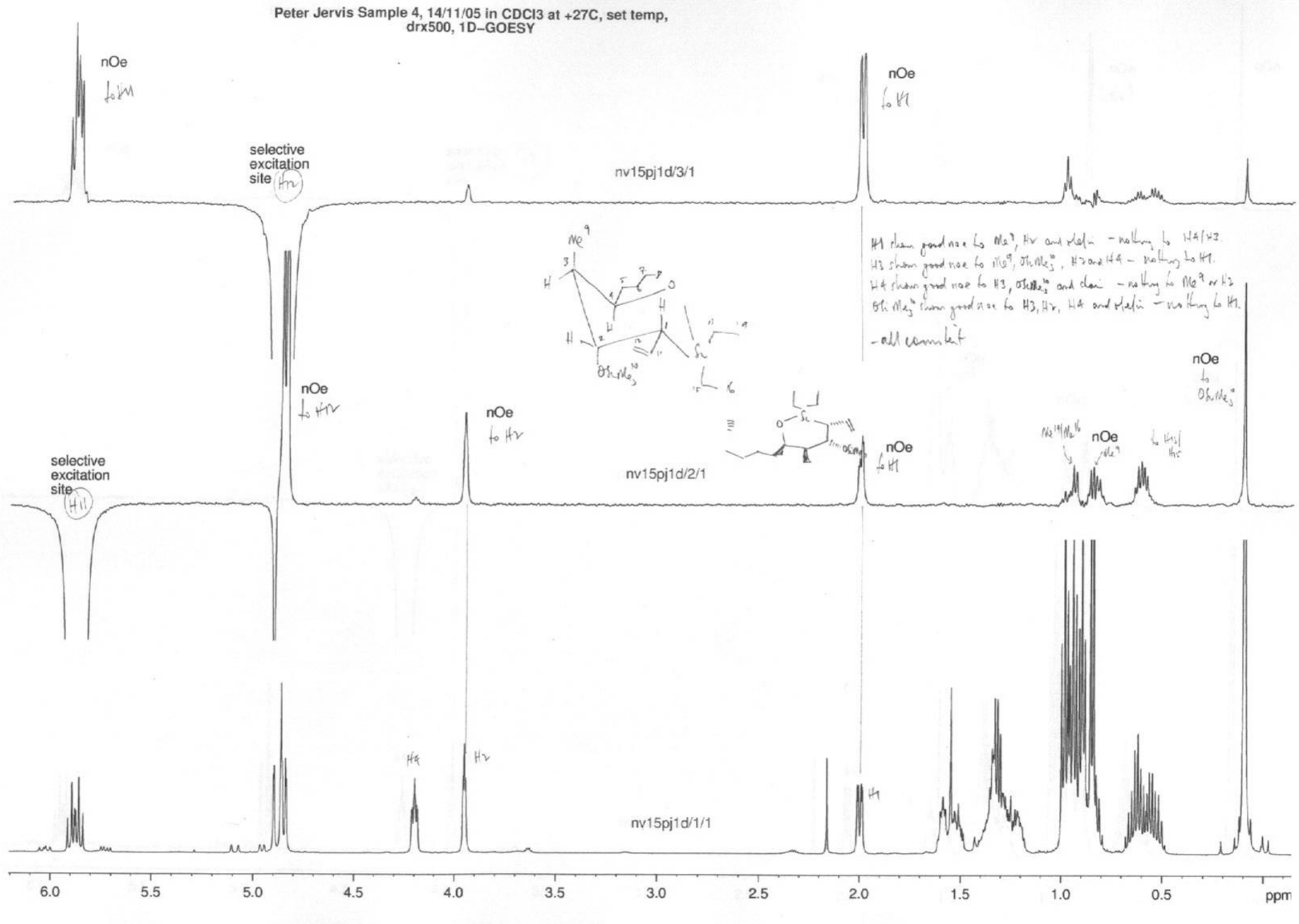
Peter Jervis Sample 4, 14/11/05 in CDCl3 at +27C, set temp,
drx500, 1D-GOESY
nOe
selective
excitation
site
nv15pj1d/3/1
nOe
nv15pj1d/2/1
nOe
nOe
nOe
nOe
nv15pj1d/1/1
6.0
5.5
5.0
4.5
4.0
3.5
3.0
2.5
2.0
1.5
1.0
0.5
ppm

17a

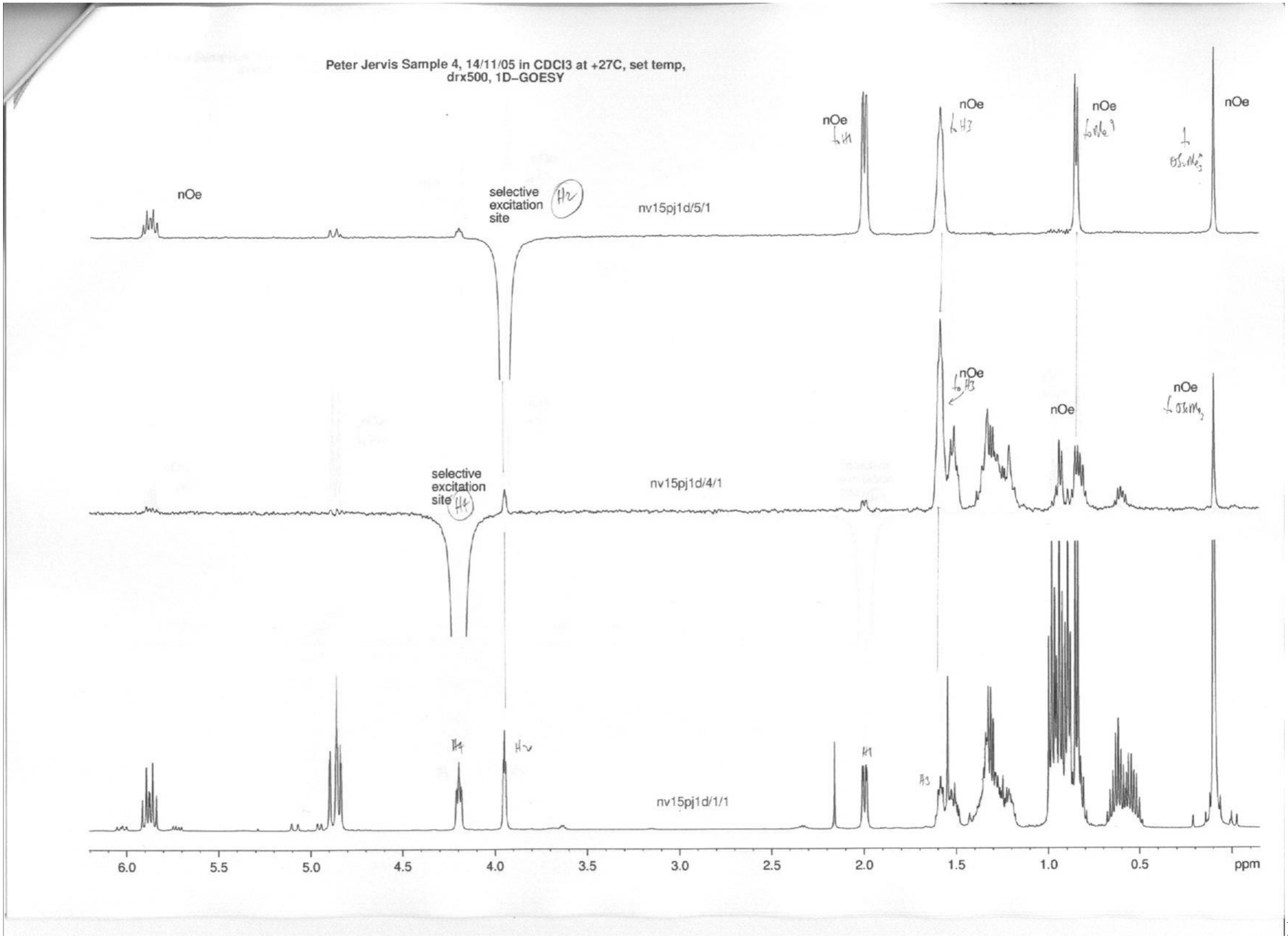

Peter Jervis Sample 4, 14/11/05 in CDCl3 at +27C, set temp,
drx500, 1D-GOESY
nOe
selective
excitation
site
nv15pj1d/5/1
nOe
nOe
nOe
nOe
selective
excitation
site
nv15pj1d/4/1
nOe
nOe
nOe
nv15pj1d/1/1
6.0
5.5
5.0
4.5
4.0
3.5
3.0
2.5
2.0
1.5
1.0
0.5
ppm

17a

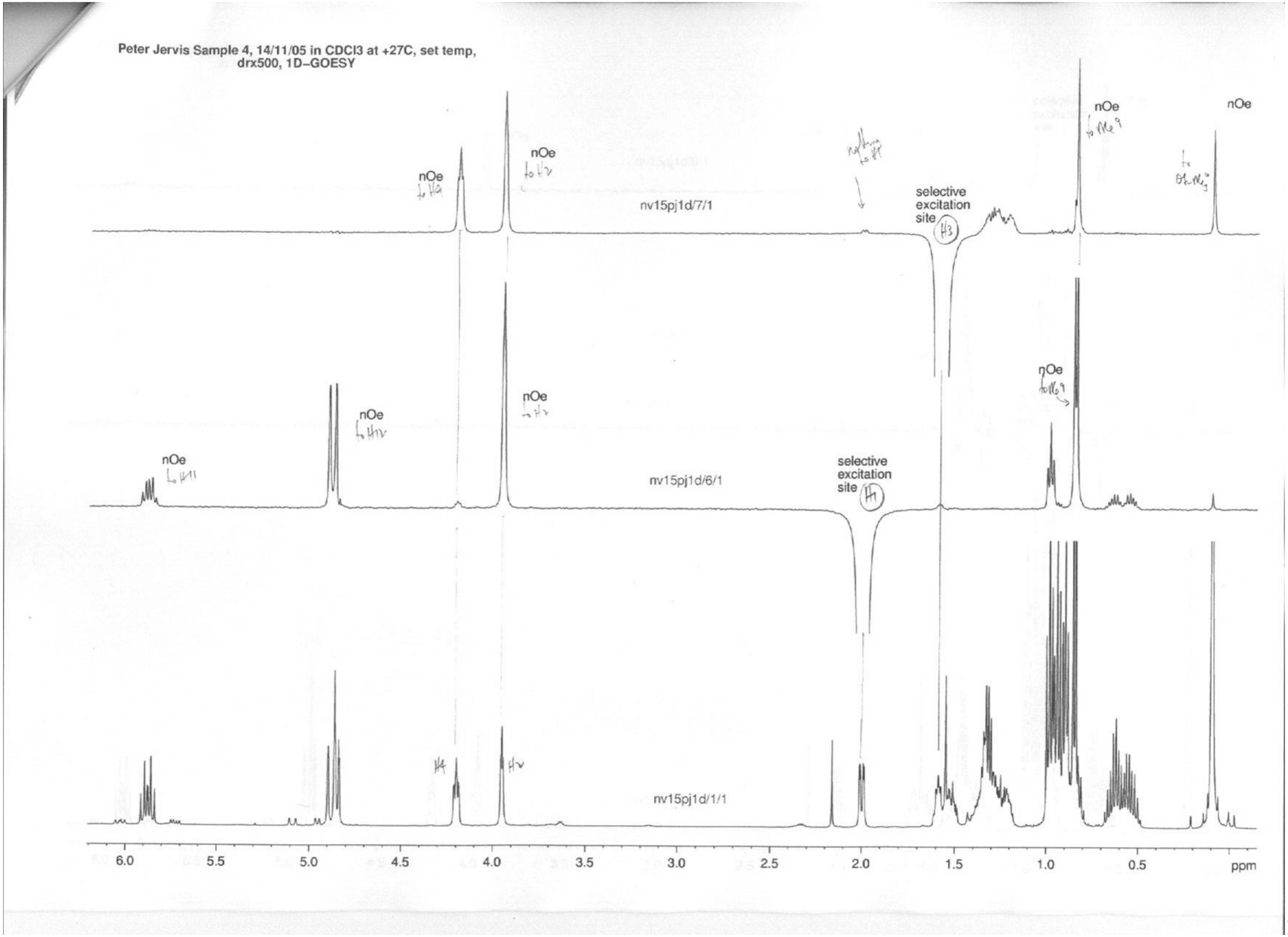

Peter Jervis Sample 4, 14/11/05 in CDCl3 at +27C, set temp,
drx500, 1D–GOESY
nOe
nOe
nv15pj1d/7/1
selective
excitation
site
H3
nOe
nOe
nOe
nOe
nOe
nv15pj1d/6/1
selective
excitation
site
H1
nOe
nv15pj1d/1/1
6.0
5.5
5.0
4.5
4.0
3.5
3.0
2.5
2.0
1.5
1.0
0.5
ppm

17a

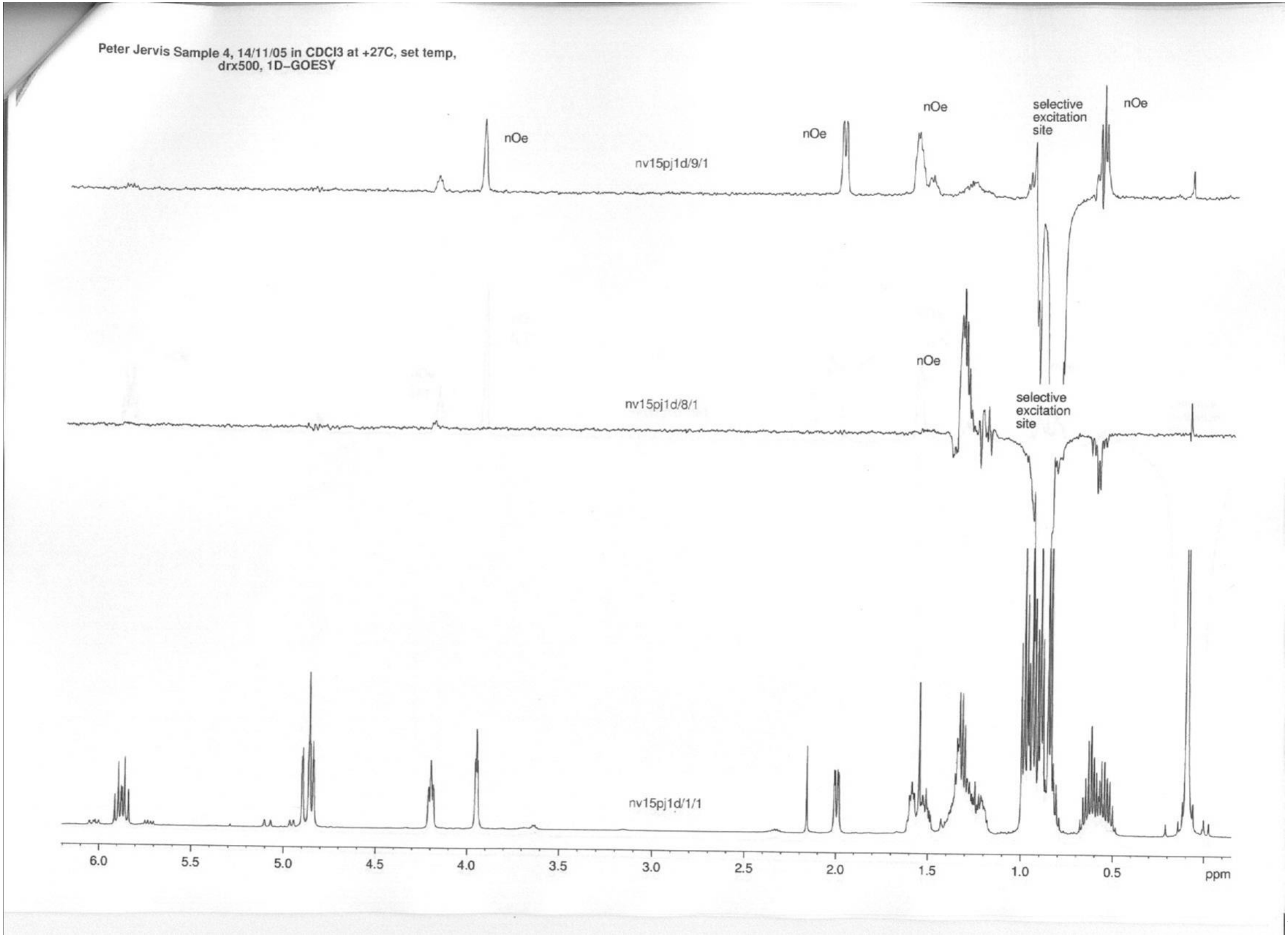
Peter Jervis Sample 4, 14/11/05 in CDCl3 at +27C, set temp,
drx500, 1D-GOESY
nOe
nOe
nOe
selective
excitation
site
nOe
nv15pj1d/9/1
nOe
selective
excitation
site
nv15pj1d/8/1
nv15pj1d/1/1
6.0
5.5
5.0
4.5
4.0
3.5
3.0
2.5
2.0
1.5
1.0
0.5
ppm

17a
