# Supplementary material for: Tether-directed synthesis of highly substituted oxasilacycles via an intramolecular allylation employing allylsilanes
Source: Beilstein J Org Chem. 2007 Feb 8;3:6. doi: 10.1186/1860-5397-3-6 (PMC1810303; doi:10.1186/1860-5397-3-6)
Supplement: File 4 — 1H-NMR and 13C-NMR Spectra for the following compounds: 18a, 19a. [file Beilstein_J_Org_Chem-03-06-s004.pdf]

Additional File 4

Tether-directed synthesis of highly substituted oxasilacycles *via* an intramolecular allylation employing allylsilanes

Peter J. Jervis and Liam R. Cox*

email: l.r.cox@bham.ac.uk

^{1}H-NMR and ^{13}C-NMR Spectra for the following compounds:

18a, 19a

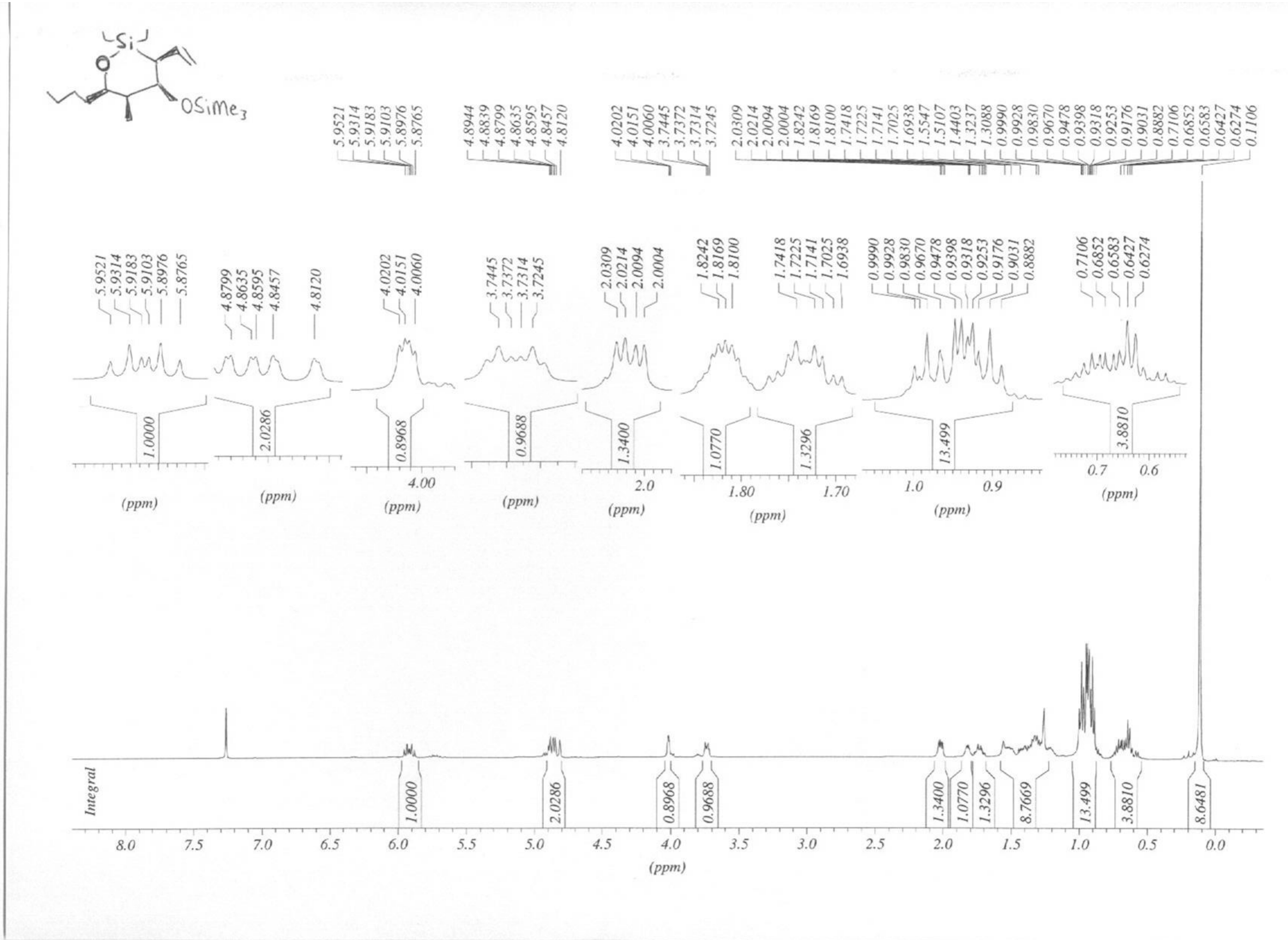
Si
O
OSiMe3
5.9521 5.9314 5.9183 5.9103 5.8976 5.8765
4.8944 4.8839 4.8799 4.8635 4.8595 4.8457 4.8120
4.0202 4.0151 4.0060 3.7445 3.7372 3.7314 3.7245
2.0309 2.0214 2.0094 2.0004 1.8242 1.8169 1.8100 1.7418 1.7225 1.7141 1.7025 1.6938 1.5547 1.5107 1.4403 1.3237 1.3088 0.9990 0.9928 0.9830 0.9670 0.9478 0.9398 0.9318 0.9253 0.9176 0.9031 0.8882 0.7106 0.6852 0.6583 0.6427 0.6274 0.1106
1.0000
2.0286
0.8968
4.00
0.9688
1.3400
2.0
1.0770
1.3296
1.80
1.70
13.499
1.0
0.9
3.8810
0.7
0.6
(ppm)
Integral
1.0000 2.0286 0.8968 0.9688 1.3400 1.0770 1.3296 8.7669 13.499 3.8810 8.6481
8.0 7.5 7.0 6.5 6.0 5.5 5.0 4.5 4.0 3.5 3.0 2.5 2.0 1.5 1.0 0.5 0.0
(ppm)

18a

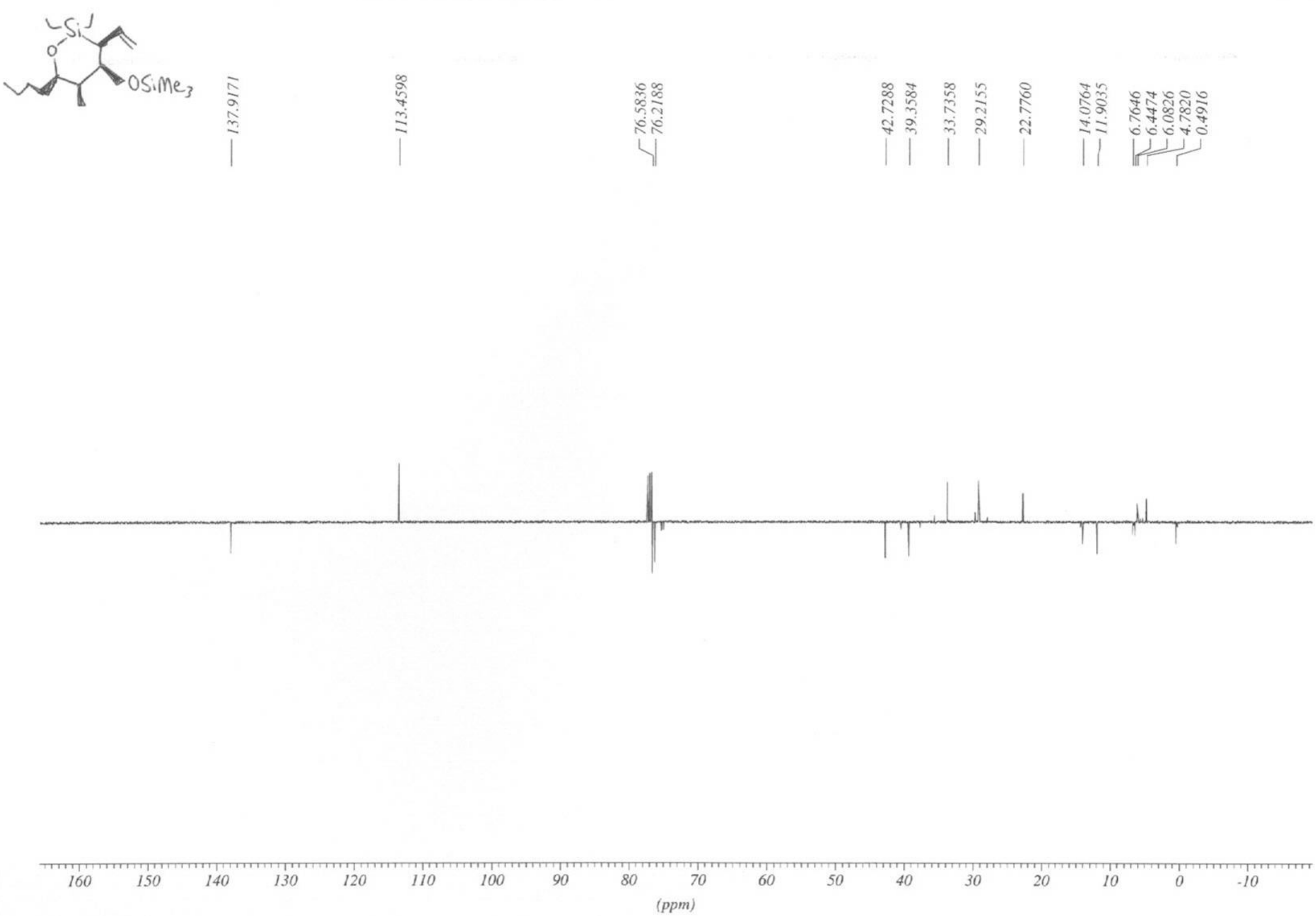
OSiMe3
137.9171
113.4598
76.5836
76.2188
42.7288
39.3584
33.7358
29.2155
22.7760
14.0764
11.9035
6.7646
6.4474
6.0826
4.7820
0.4916
160 150 140 130 120 110 100 90 80 70 60 50 40 30 20 10 0 -10
(ppm)

18a

Peter Jervis Sample 3, 22/11/05 in CDCl3 at +27C, set temp
drx500, Gradient COSY90

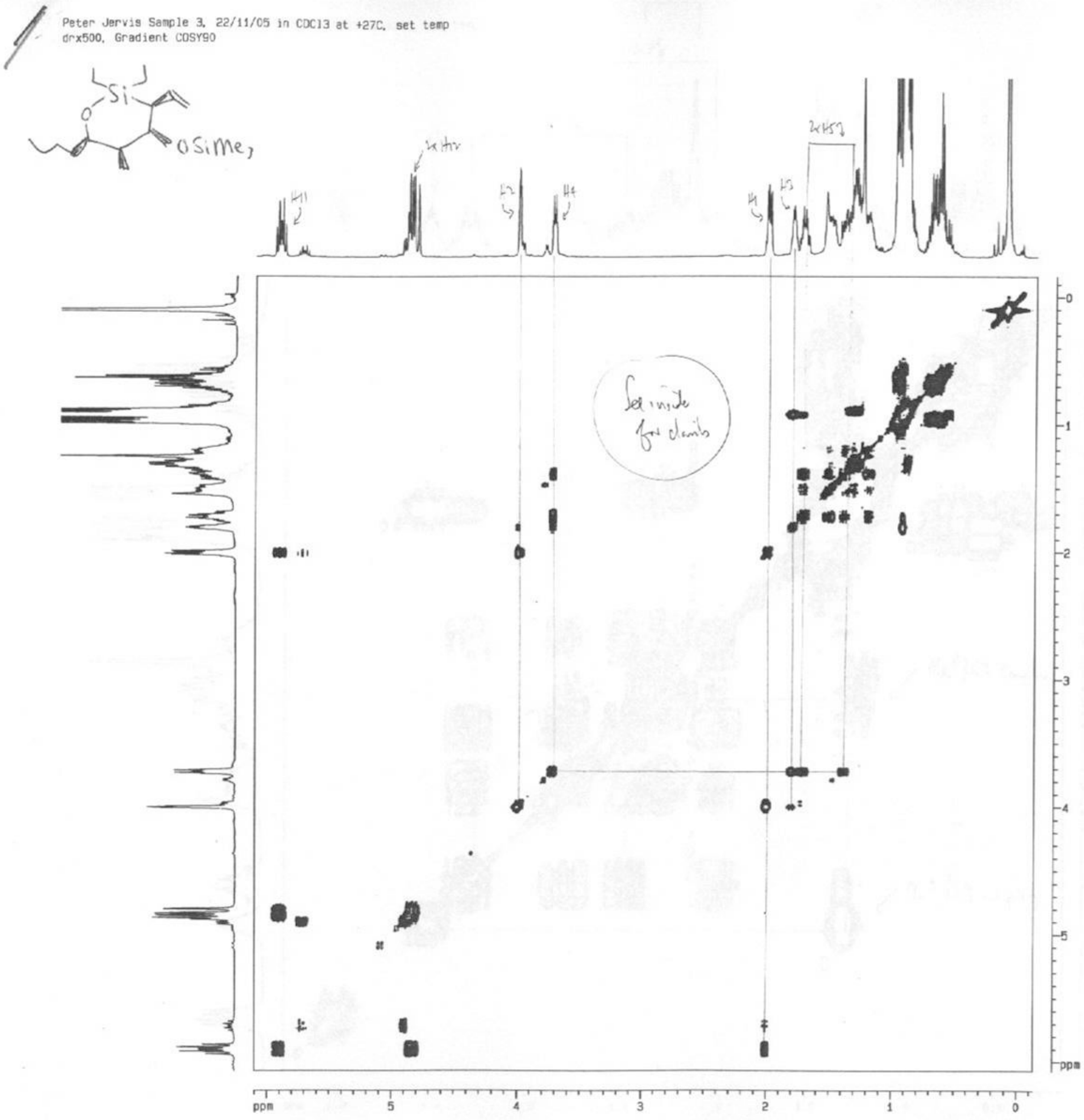

Current Data Parameters

NAME	nv22pjid
EXPNO	4
PROCNO	1

F2 - Acquisition Parameters

Date_	20051122
Time	21.24
INSTRUM	drx500
PROBHD	5 mm TBI H/C
PULPROG	cosygp
TD	2048
SOLVENT	CDCl3
NS	8
DS	16
SWH	4310.345 Hz
FIDRES	2.104661 Hz
AQ	0.2376180 sec
RG	114
DW	116.000 usec
DE	5.50 usec
TE	300.0 K
d0	0.00000300 sec
D1	2.00000000 sec
d13	0.00000300 sec
D16	0.00010000 sec
IN0	0.00023200 sec

============ CHANNEL f1 ============

NUC1	1H
P0	10.70 usec
P1	10.70 usec
PL1	1.00 dB
SFO1	500.1318867 MHz

============ GRADIENT CHANNEL ========

GPNAM1	SINE.100
GPNAM2	SINE.100
GPX1	0.00 %
GPX2	0.00 %
GPY1	0.00 %
GPY2	0.00 %
GPZ1	10.00 %
GPZ2	10.00 %
P16	1000.00 usec

F1 - Acquisition parameters

ND0	1
TD	512
SFO1	500.1319 MHz
FIDRES	8.418642 Hz
SW	8.618 ppm

F2 - Processing parameters

SI	2048
SF	500.1300233 MHz
WDW	SINE
SSB	0
LB	0.00 Hz
GB	0
PC	1.00

F1 - Processing parameters

SI	1024
MC2	QF
SF	500.1300233 MHz
WDW	SINE
SSB	0
LB	0.00 Hz
GB	0

2D NMR plot parameters

CX2	20.00 cm
CX1	20.00 cm
F2PLO	6.104 ppm
F2LO	3052.59 Hz
F2PHI	-0.129 ppm
F2HI	-64.41 Hz
F1PLO	6.074 ppm
F1LO	3037.86 Hz
F1PHI	-0.162 ppm
F1HI	-81.25 Hz
F2PPMCM	0.31162 ppm/cm
F2HZCM	155.85011 Hz/cm
F1PPMCM	0.31183 ppm/cm
F1HZCM	155.95535 Hz/cm

18a

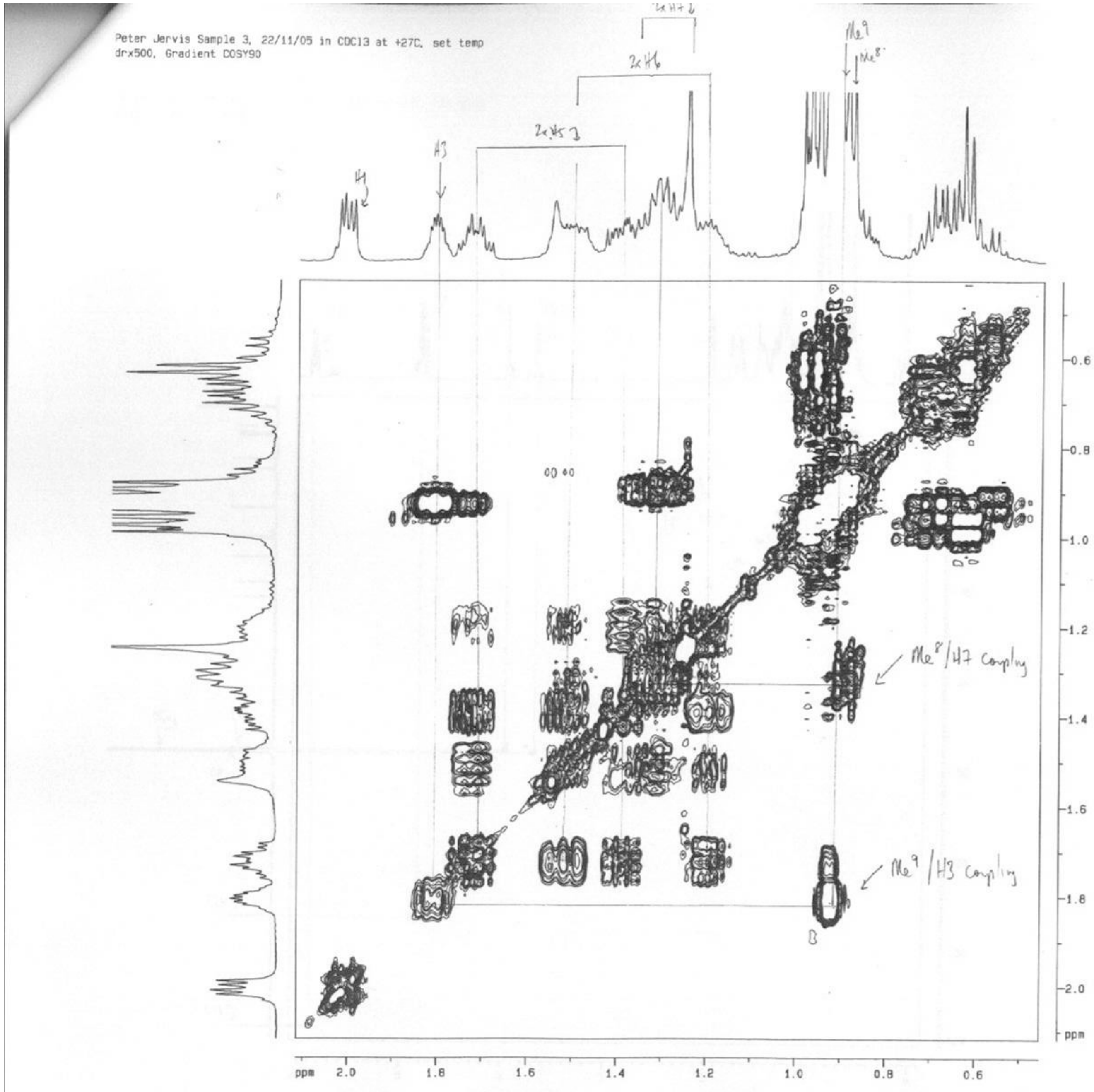
Peter Jervis Sample 3, 22/11/05 in CDCl3 at +27C, set temp
drx500, Gradient COSY90
H4
H3
2xH5
2xH6
2xH7
Me9
Me8
Me8/H7 Coupling
Me9/H3 Coupling
ppm
2.0
1.8
1.6
1.4
1.2
1.0
0.8
0.6

18a

Peter Jervis Sample 3, 22/11/05 in CDCl3 at +27C, set temp
drx500, Gradient HSGC

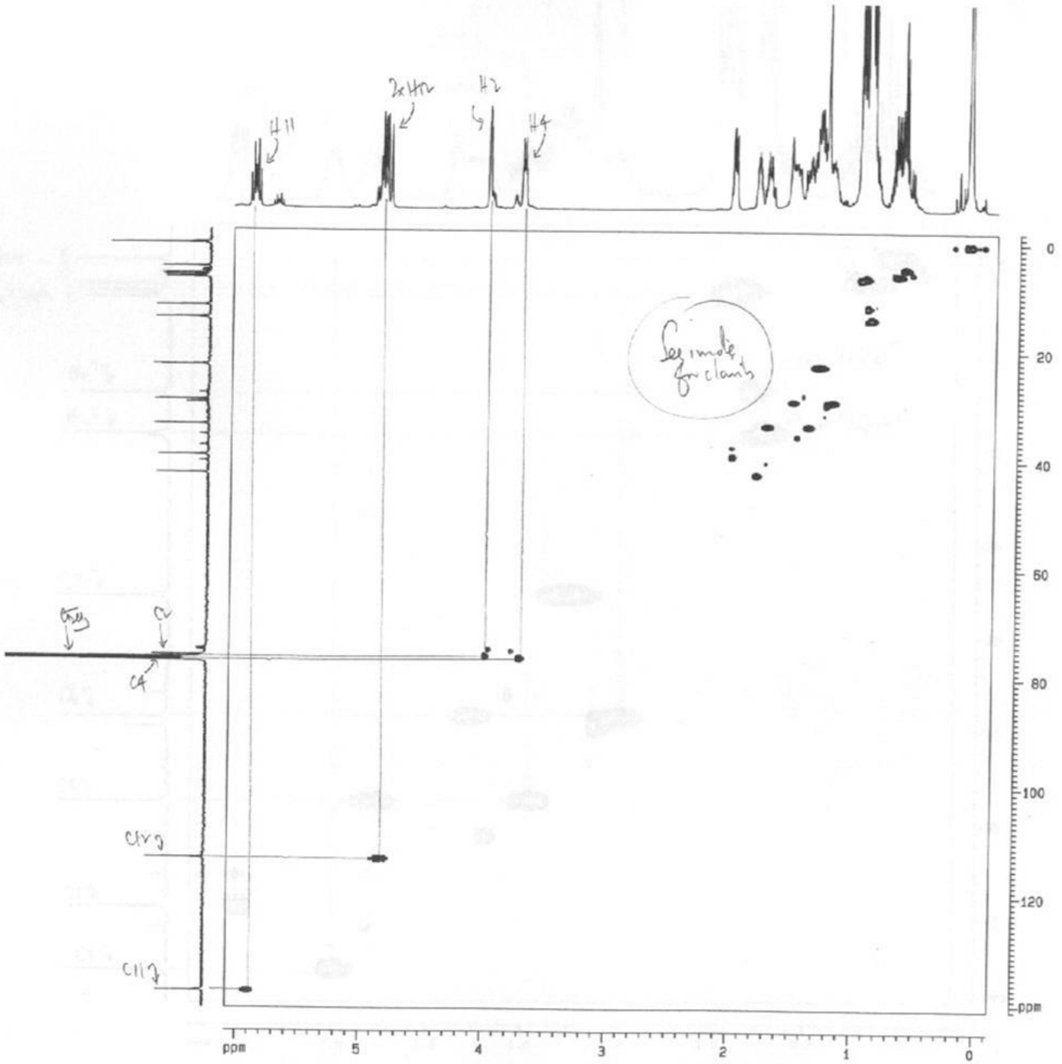

Current Data Parameters

NAME	nv22pj1d
EXPNO	2
PROCNO	1

F2 - Acquisition Parameters

Date_	20051122
Time	16.07
INSTRUM	drx500
PROBHD	5 mm TBI H/C
PULPROG	invigptp
TD	2048
SOLVENT	CDCl3
NS	8
DS	16
SWH	4310.345 Hz
FIDRES	2.104661 Hz
AQ	0.2376180 sec
RG	32768
DW	116.000 usec
DE	5.50 usec
TE	300.0 K
CNST2	145.0000000
d0	0.00000300 sec
D1	2.00000000 sec
d4	0.00172414 sec
d11	0.03000000 sec
d13	0.00000300 sec
D16	0.00010000 sec
d20	0.00110000 sec
d21	0.00061714 sec
IN0	0.00001140 sec

============ CHANNEL f1 ============

NUC1	1H
P1	10.70 usec
p2	21.40 usec
PL1	1.00 dB
SFO1	500.1318867 MHz

============ CHANNEL f2 ============

CPDPRG2	garp
NUC2	13C
P3	12.00 usec
p4	24.00 usec
PCPD2	76.00 usec
PL2	-1.00 dB
PL12	15.00 dB
SFO2	125.7667893 MHz

============ GRADIENT CHANNEL ============

GPNAM1	SINE.100
GPNAM2	SINE.100
GPNAM3	SINE.100
GPX1	0.00 %
GPX2	0.00 %
GPX3	0.00 %
GPY1	0.00 %
GPY2	0.00 %
GPY3	0.00 %
GPZ1	80.00 %
GPZ2	30.00 %
GPZ3	20.10 %
P16	1000.00 usec

F1 - Acquisition parameters

ND0	4
TD	512
SFO1	125.7668 MHz
FIDRES	42.831888 Hz
SW	174.369 ppm

F2 - Processing parameters

SI	2048
SF	500.1300233 MHz
WDW	QSINE
SSB	2
LB	0.00 Hz
GB	0
PC	1.00

F1 - Processing parameters

SI	1024
MC2	TPPI
SF	125.7577910 MHz
WDW	QSINE
SSB	2
LB	0.00 Hz
GB	0

2D NMR plot parameters

CX2	17.00 cm
CX1	17.00 cm
F2PLO	6.083 ppm
F2LO	3042.07 Hz
F2PHI	-0.129 ppm
F2HI	-64.41 Hz
F1PLO	141.033 ppm
F1LO	17736.00 Hz
F1PHI	-2.014 ppm
F1HI	-253.31 Hz
F2PPMCM	0.36537 ppm/cm
F2HZCM	182.73405 Hz/cm
F1PPMCM	8.41455 ppm/cm
F1HZCM	1058.19458 Hz/cm

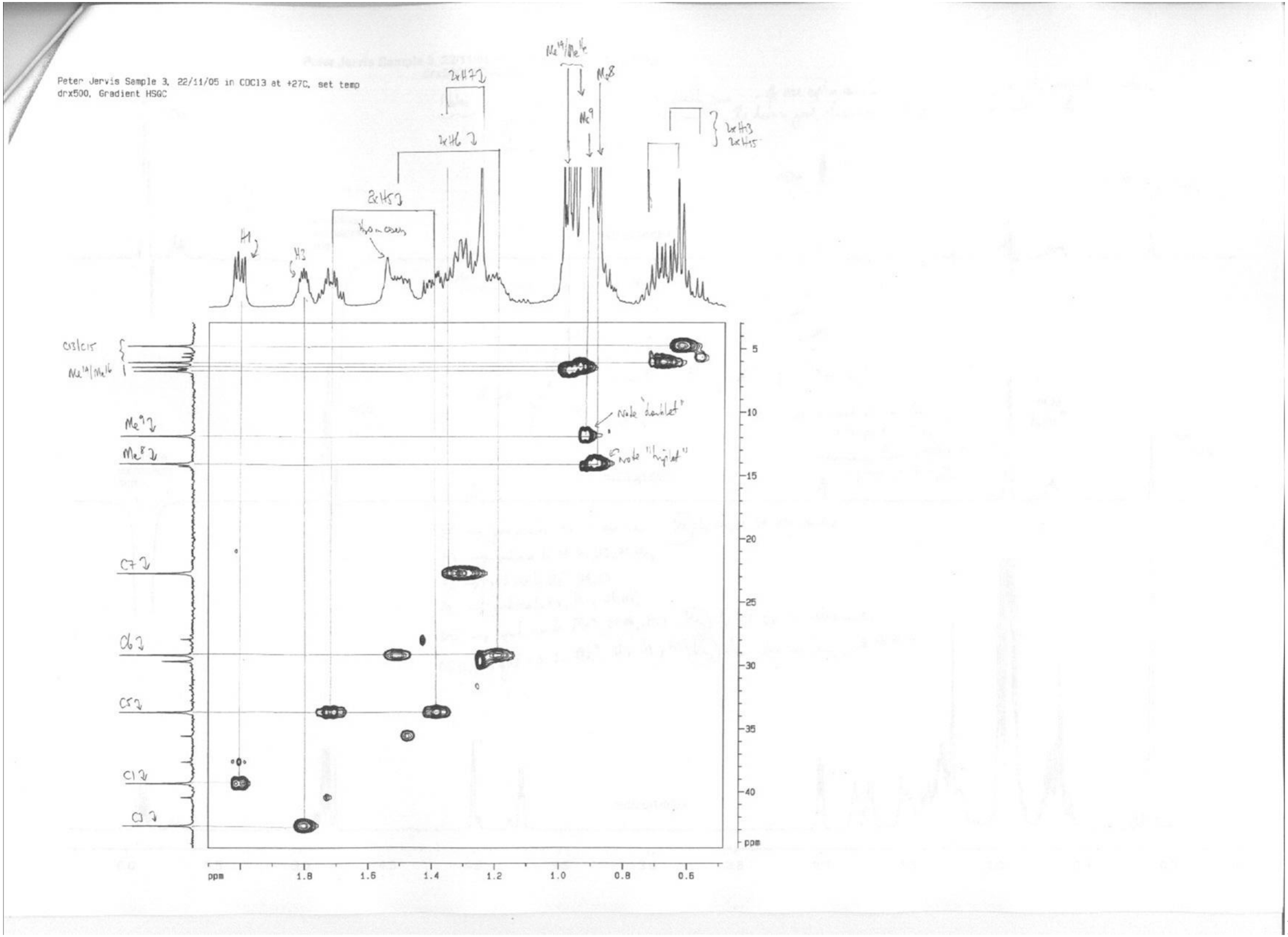
Peter Jervis Sample 3, 22/11/05 in CDCl3 at +27C, set temp
drx500, Gradient HSGC
H1
H3
2xH5
2xH6
2xH7
Me14/Me16
Me9
Me8
2xH13
2xH15
C13/C15
Me14/Me16
Me9
Me8
C7
C6
C5
C1
C3
Note "doublet"
Note "triplet"
ppm
1.8
1.6
1.4
1.2
1.0
0.8
0.6
5
10
15
20
25
30
35
40

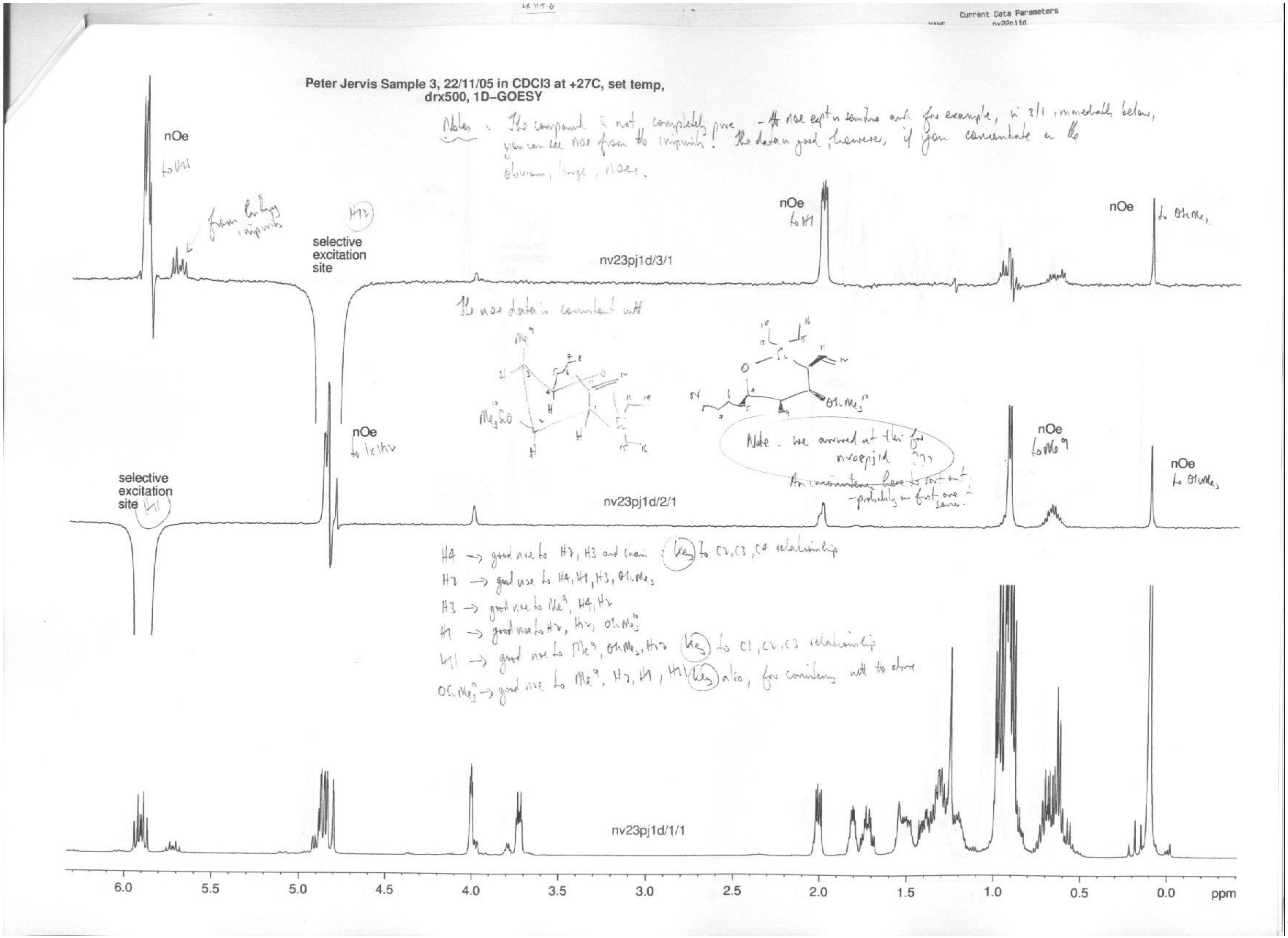

Current Data Parameters
Peter Jervis Sample 3, 22/11/05 in CDCl3 at +27C, set temp,
drx500, 1D–GOESY
nOe
selective excitation site
nv23pj1d/3/1
nOe
nOe
selective excitation site
nOe
nv23pj1d/2/1
nOe
nOe
nv23pj1d/1/1
6.0
5.5
5.0
4.5
4.0
3.5
3.0
2.5
2.0
1.5
1.0
0.5
0.0
ppm

18a

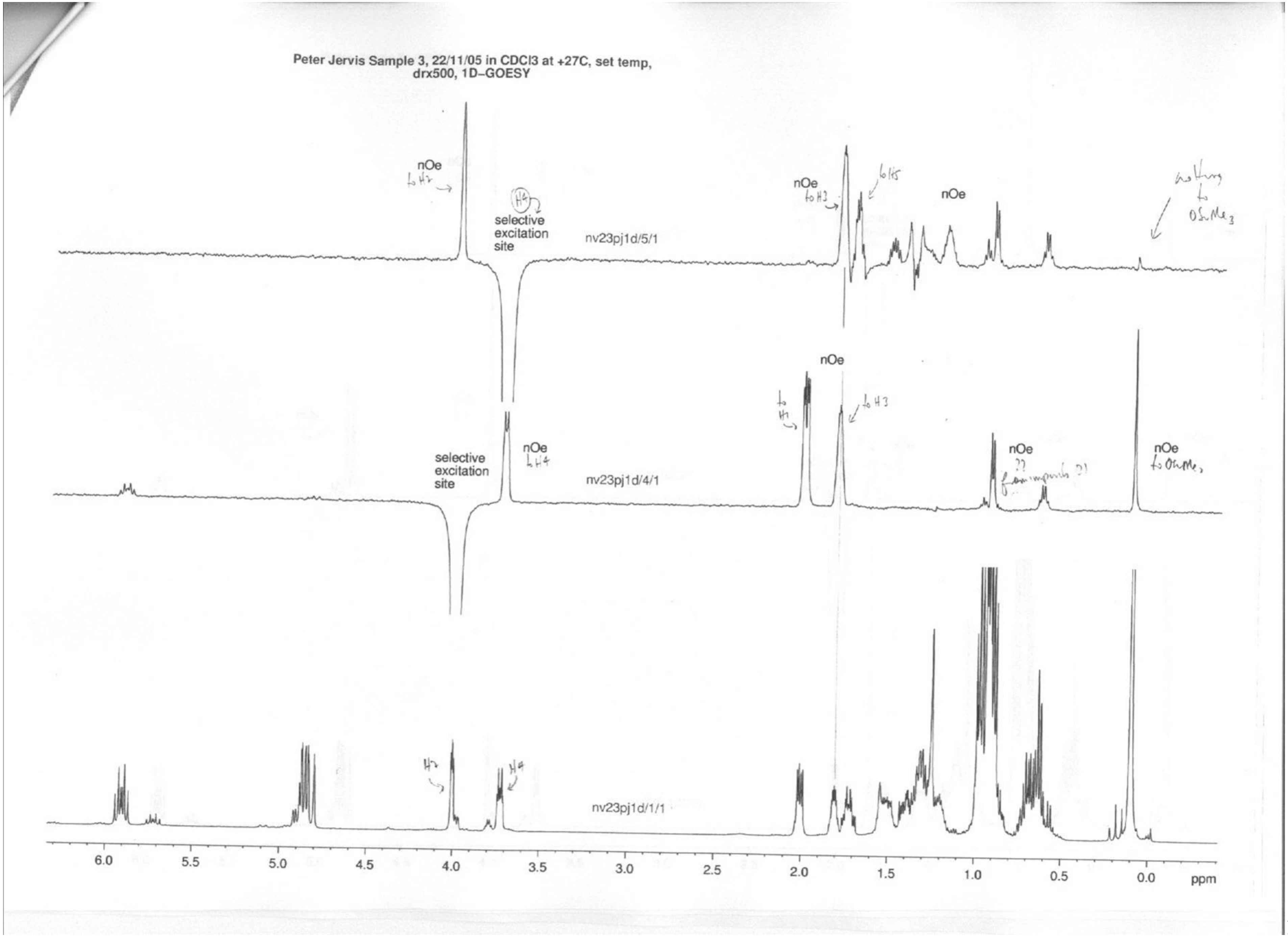
Peter Jervis Sample 3, 22/11/05 in CDCl3 at +27C, set temp,
drx500, 1D–GOESY
nOe
selective
excitation
site
nv23pj1d/5/1
nOe
nOe
nOe
selective
excitation
site
nOe
nv23pj1d/4/1
nOe
nOe
nv23pj1d/1/1
6.0
5.5
5.0
4.5
4.0
3.5
3.0
2.5
2.0
1.5
1.0
0.5
0.0
ppm

18a

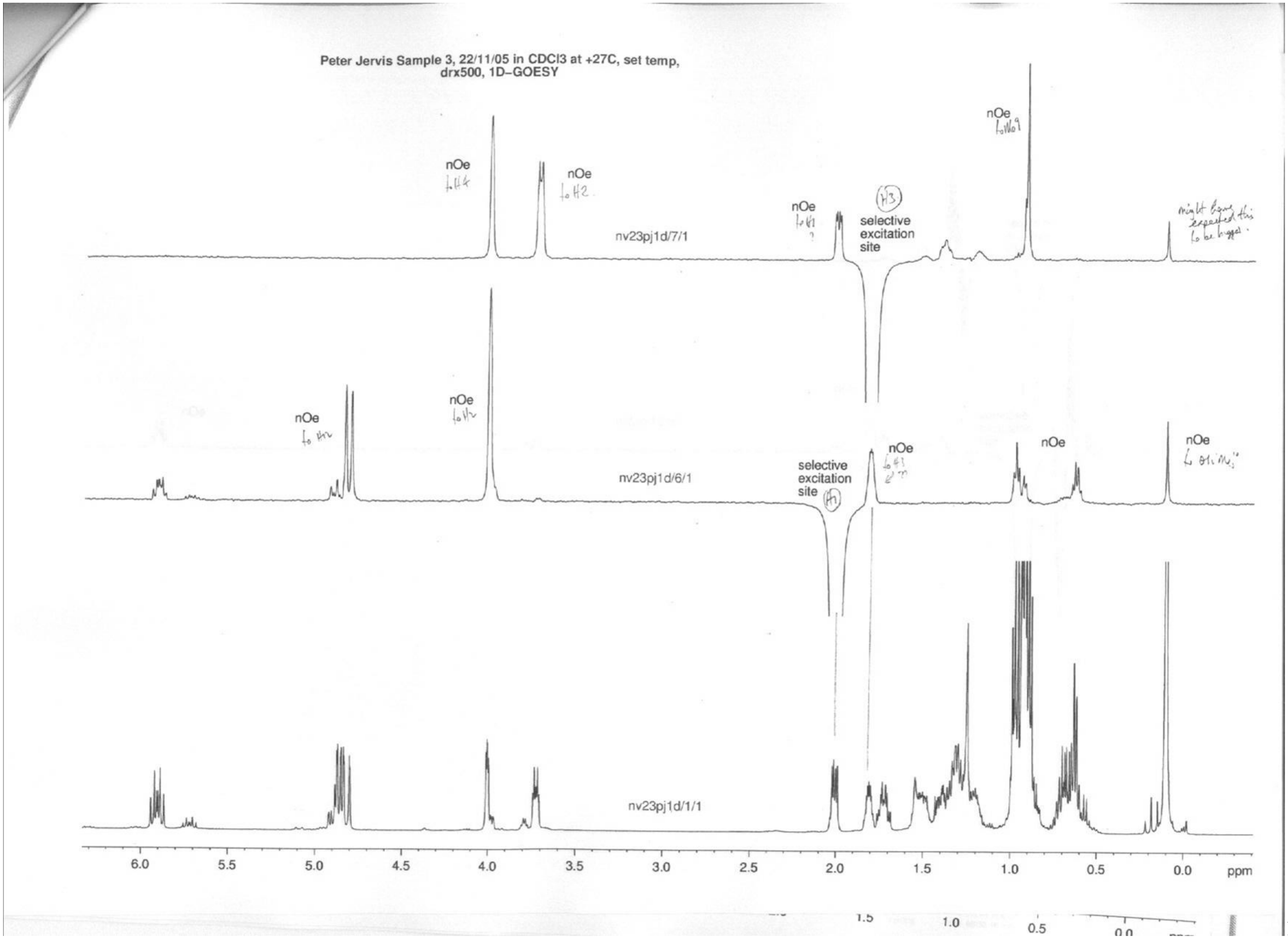
Peter Jervis Sample 3, 22/11/05 in CDCl3 at +27C, set temp, drx500, 1D-GOESY
nOe
nOe
nOe
nOe
selective excitation site
nv23pj1d/7/1
nOe
nOe
nOe
nOe
nOe
selective excitation site
nv23pj1d/6/1
nv23pj1d/1/1
6.0
5.5
5.0
4.5
4.0
3.5
3.0
2.5
2.0
1.5
1.0
0.5
0.0
ppm

18a

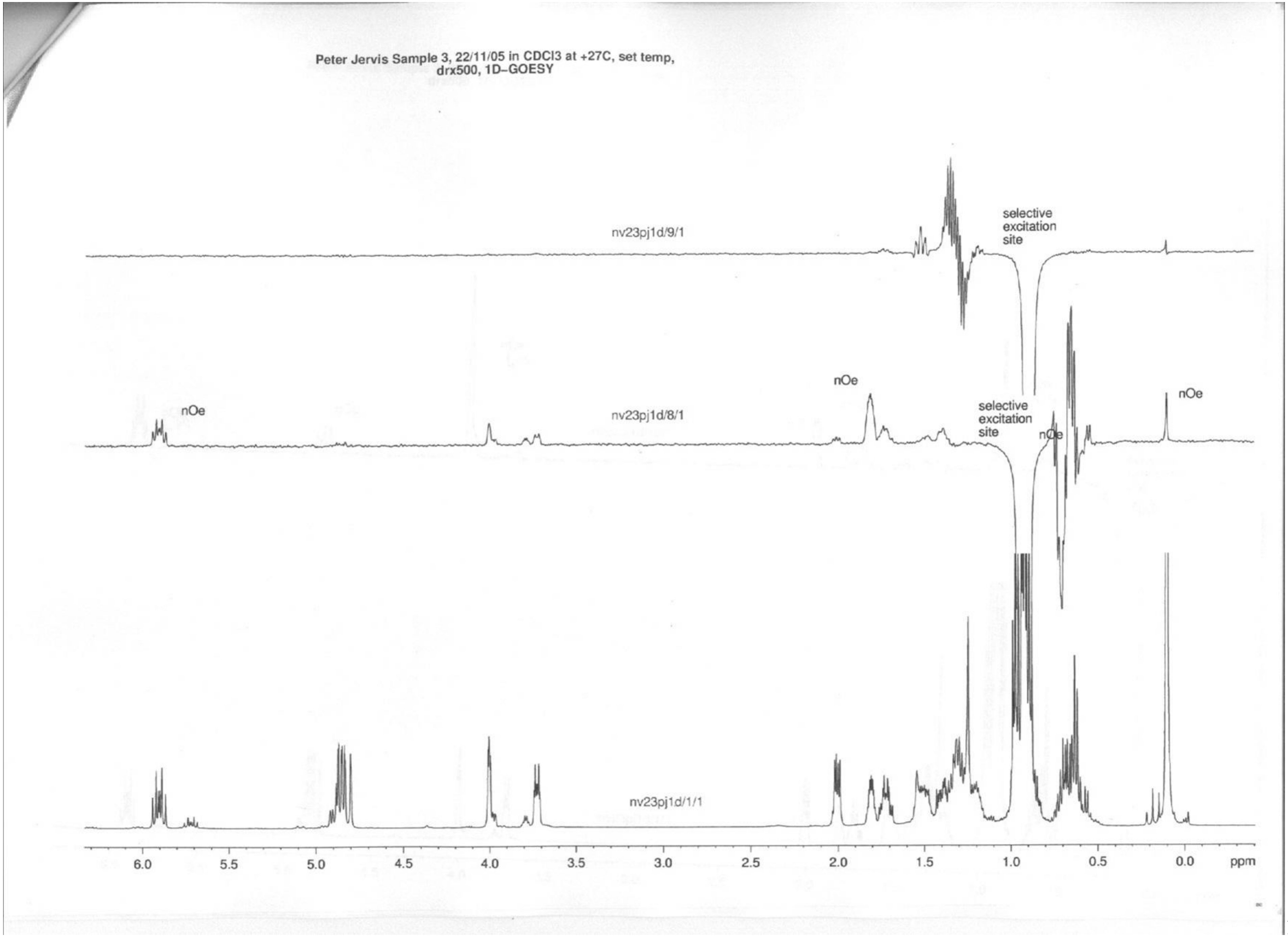
Peter Jervis Sample 3, 22/11/05 in CDCl3 at +27C, set temp,
drx500, 1D-GOESY
nv23pj1d/9/1
selective
excitation
site
nOe
nOe
nv23pj1d/8/1
selective
excitation
site
nOe
nOe
nv23pj1d/1/1
6.0
5.5
5.0
4.5
4.0
3.5
3.0
2.5
2.0
1.5
1.0
0.5
0.0
ppm

18a

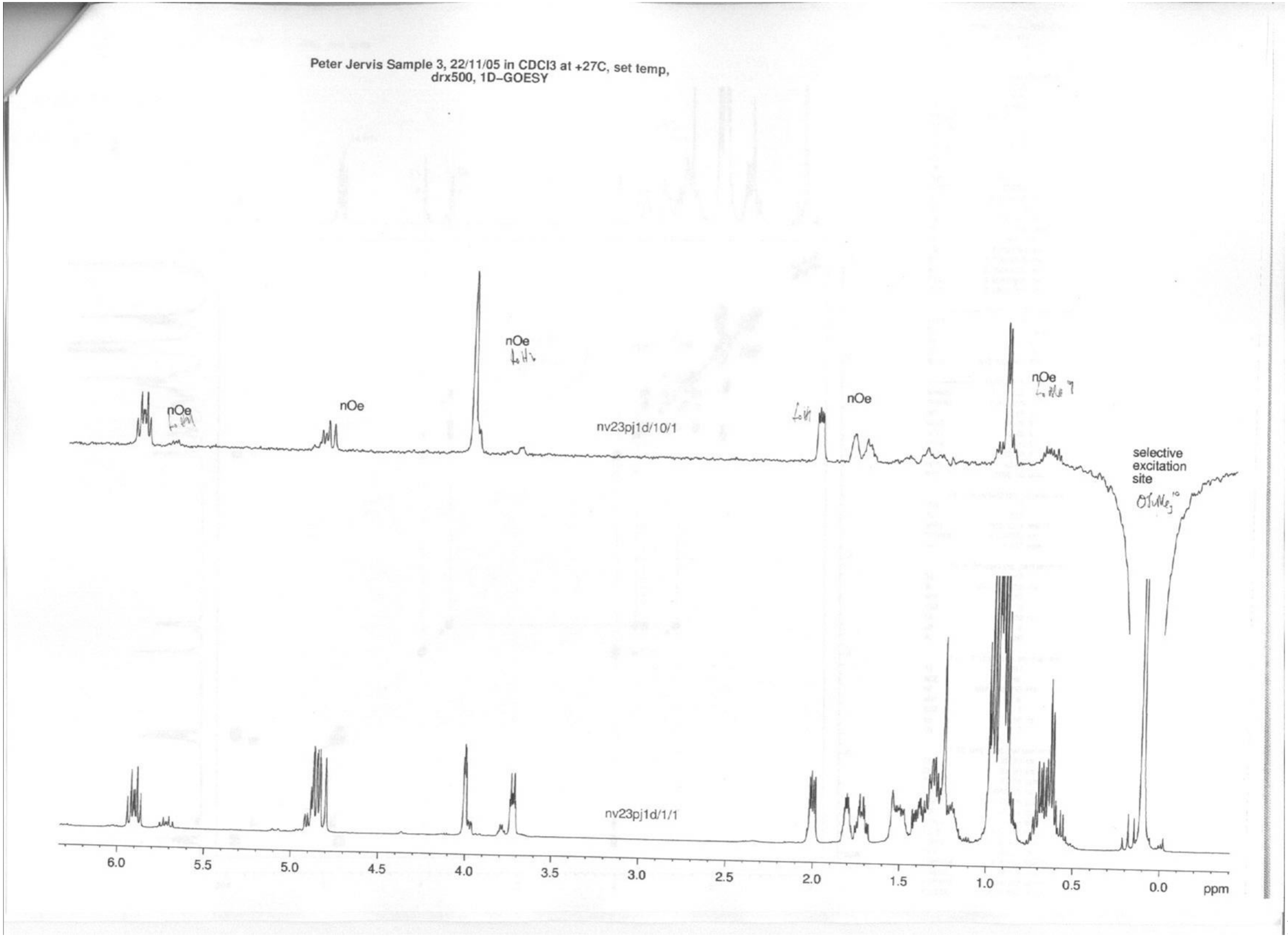
Peter Jervis Sample 3, 22/11/05 in CDCl3 at +27C, set temp,
drx500, 1D-GOESY
nOe
nOe
nOe
nOe
nOe
nv23pj1d/10/1
selective
excitation
site
nv23pj1d/1/1
6.0
5.5
5.0
4.5
4.0
3.5
3.0
2.5
2.0
1.5
1.0
0.5
0.0
ppm

18a

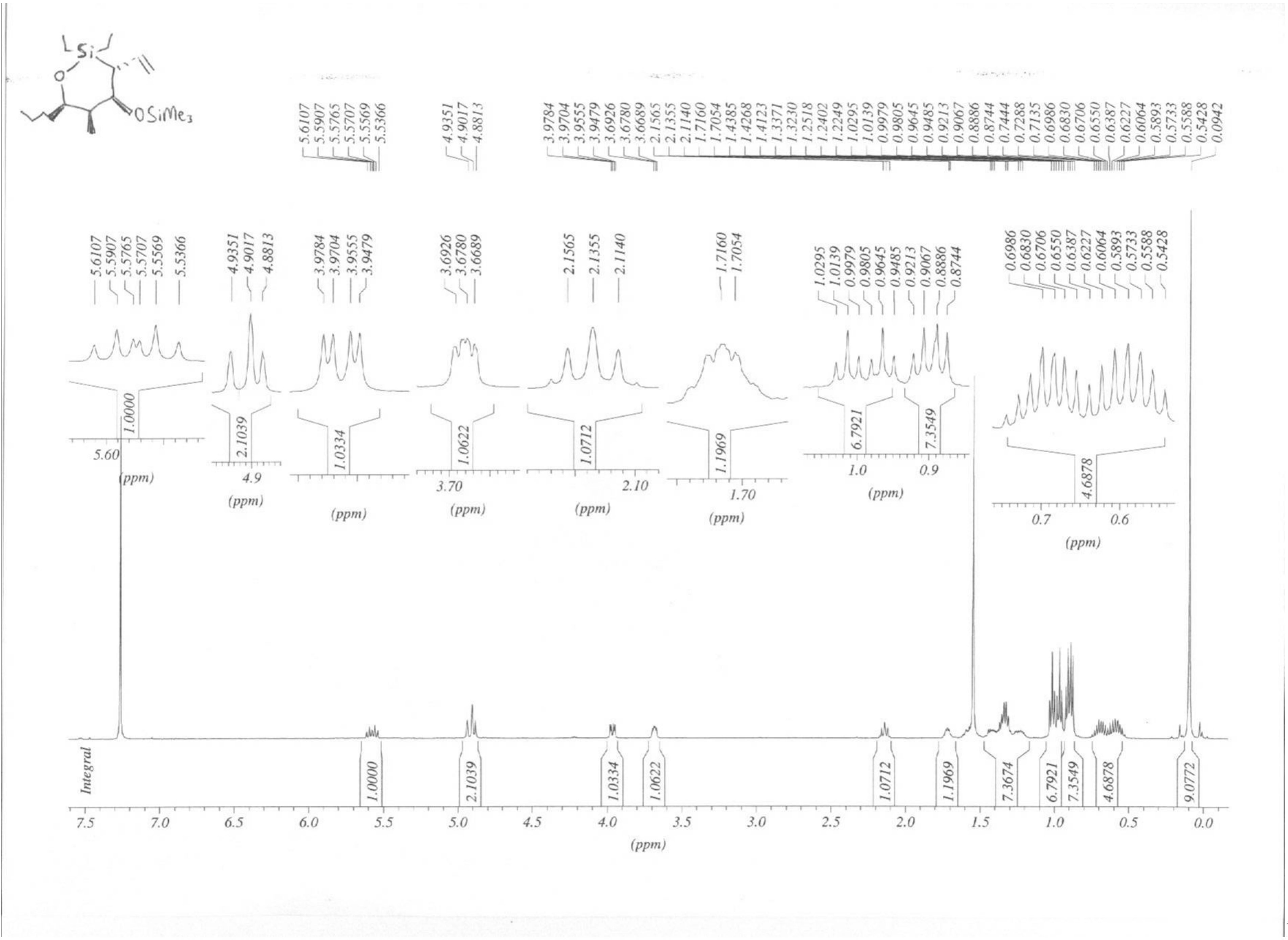
Si
O
OSiMe3
5.6107
5.5907
5.5765
5.5707
5.5569
5.5366
4.9351
4.9017
4.8813
3.9784
3.9704
3.9555
3.9479
3.6926
3.6780
3.6689
2.1565
2.1355
2.1140
1.7160
1.7054
1.4385
1.4268
1.4123
1.3371
1.3230
1.2518
1.2402
1.2249
1.0295
1.0139
0.9979
0.9805
0.9645
0.9485
0.9213
0.9067
0.8886
0.8744
0.7444
0.7288
0.7135
0.6986
0.6830
0.6706
0.6550
0.6387
0.6227
0.6064
0.5893
0.5733
0.5588
0.5428
0.0942
1.0000
2.1039
1.0334
1.0622
1.0712
1.1969
7.3674
6.7921
7.3549
4.6878
9.0772
Integral
5.60
4.9
3.70
2.10
1.70
1.0
0.9
0.7
0.6
7.5
7.0
6.5
6.0
5.5
5.0
4.5
4.0
3.5
3.0
2.5
2.0
1.5
1.0
0.5
0.0
(ppm)

19a

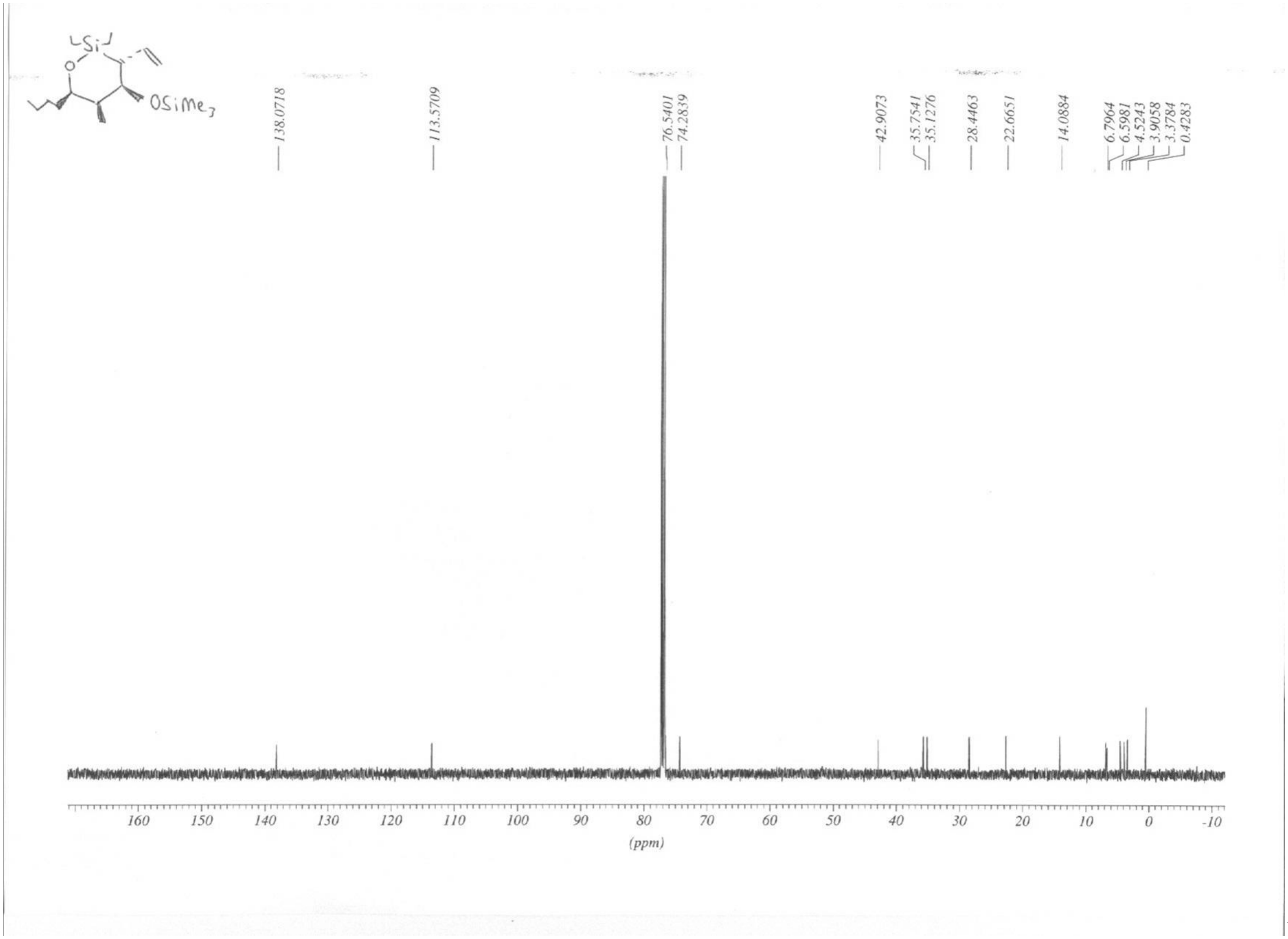

OSiMe3
Si
138.0718
113.5709
76.5401
74.2839
42.9073
35.7541
35.1276
28.4463
22.6651
14.0884
6.7964
6.5981
4.5243
3.9058
3.3784
0.4283
160
150
140
130
120
110
100
90
80
70
60
50
40
30
20
10
0
-10
(ppm)

19a

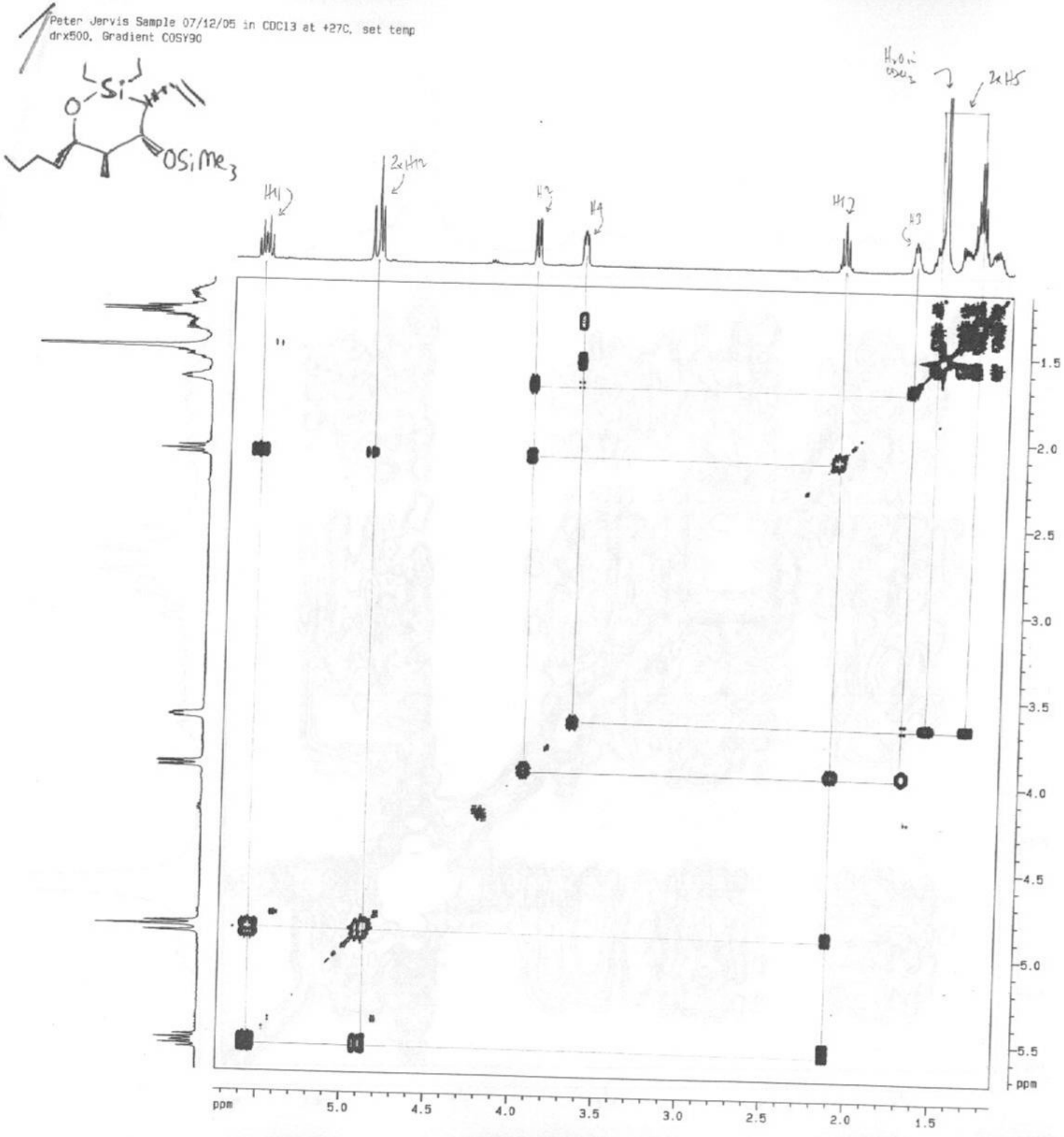

Current Data Parameters

NAME	dc07pj1d
EXPNO	2
PROCNO	1

F2 - Acquisition Parameters

Date_	20051207
Time	12.06
INSTRUM	drx500
PROBHD	5 mm TBI H/C
PULPROG	cosygp
TD	2048
SOLVENT	CDCl3
NS	8
DS	16
SWH	3415.301 Hz
FIDRES	1.667627 Hz
AQ	0.2998772 sec
RG	812.7
DW	146.400 usec
DE	5.50 usec
TE	300.0 K
d0	0.00000300 sec
D1	2.00000000 sec
d13	0.00000300 sec
D16	0.00010000 sec
IN0	0.00029280 sec

============ CHANNEL f1 ============

NUC1	1H
P0	10.70 usec
P1	10.70 usec
PL1	1.00 dB
SFO1	500.1322908 MHz

============ GRADIENT CHANNEL ========

GPNAM1	SINE.100
GPNAM2	SINE.100
GPX1	0.00 %
GPX2	0.00 %
GPY1	0.00 %
GPY2	0.00 %
GPZ1	10.00 %
GPZ2	10.00 %
P16	1000.00 usec

F1 - Acquisition parameters

ND0	1
TD	512
SFO1	500.1323 MHz
FIDRES	6.670509 Hz
SW	6.829 ppm

F2 - Processing parameters

SI	2048
SF	500.1300238 MHz
WDW	SINE
SSB	0
LB	0.00 Hz
GB	0
PC	1.00

F1 - Processing parameters

SI	1024
MC2	QF
SF	500.1300238 MHz
WDW	SINE
SSB	0
LB	0.00 Hz
GB	0

2D NMR plot parameters

CX2	20.00 cm
CX1	20.00 cm
F2PLO	5.740 ppm
F2LO	2870.70 Hz
F2PHI	1.125 ppm
F2HI	562.71 Hz
F1PLO	5.733 ppm
F1LO	2867.37 Hz
F1PHI	1.132 ppm
F1HI	566.04 Hz
F2PPMCM	0.23074 ppm/cm
F2HZCM	115.39980 Hz/cm
F1PPMCM	0.23007 ppm/cm

19a

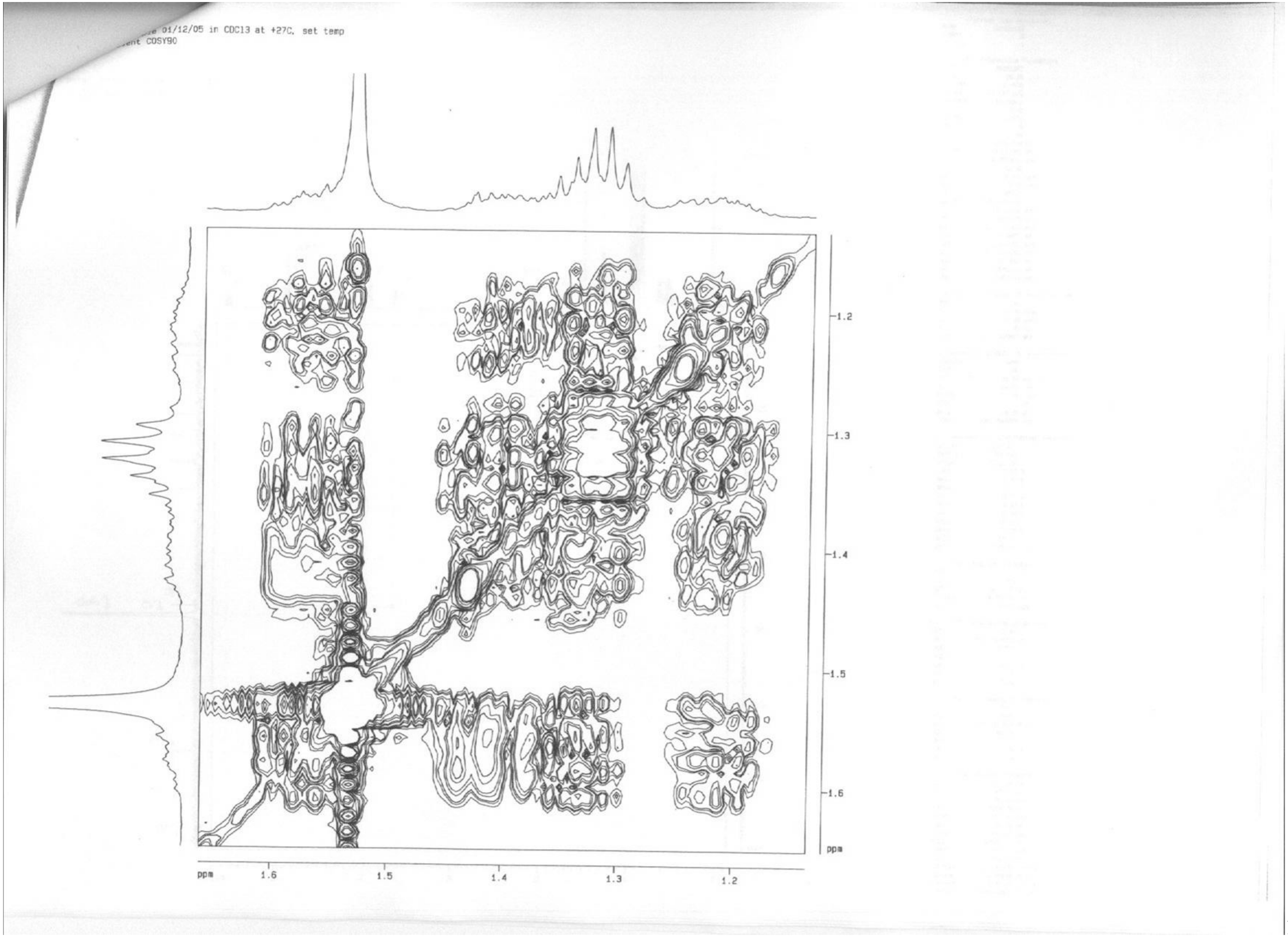
01/12/05 in CDCl3 at +27C, set temp
COSY90
1.2
1.3
1.4
1.5
1.6
ppm
1.6
1.5
1.4
1.3
1.2
ppm

19a

Peter Jervis Sample 07/12/05 in CDCl3 at +27C, set temp
drx500, Gradient HSGC

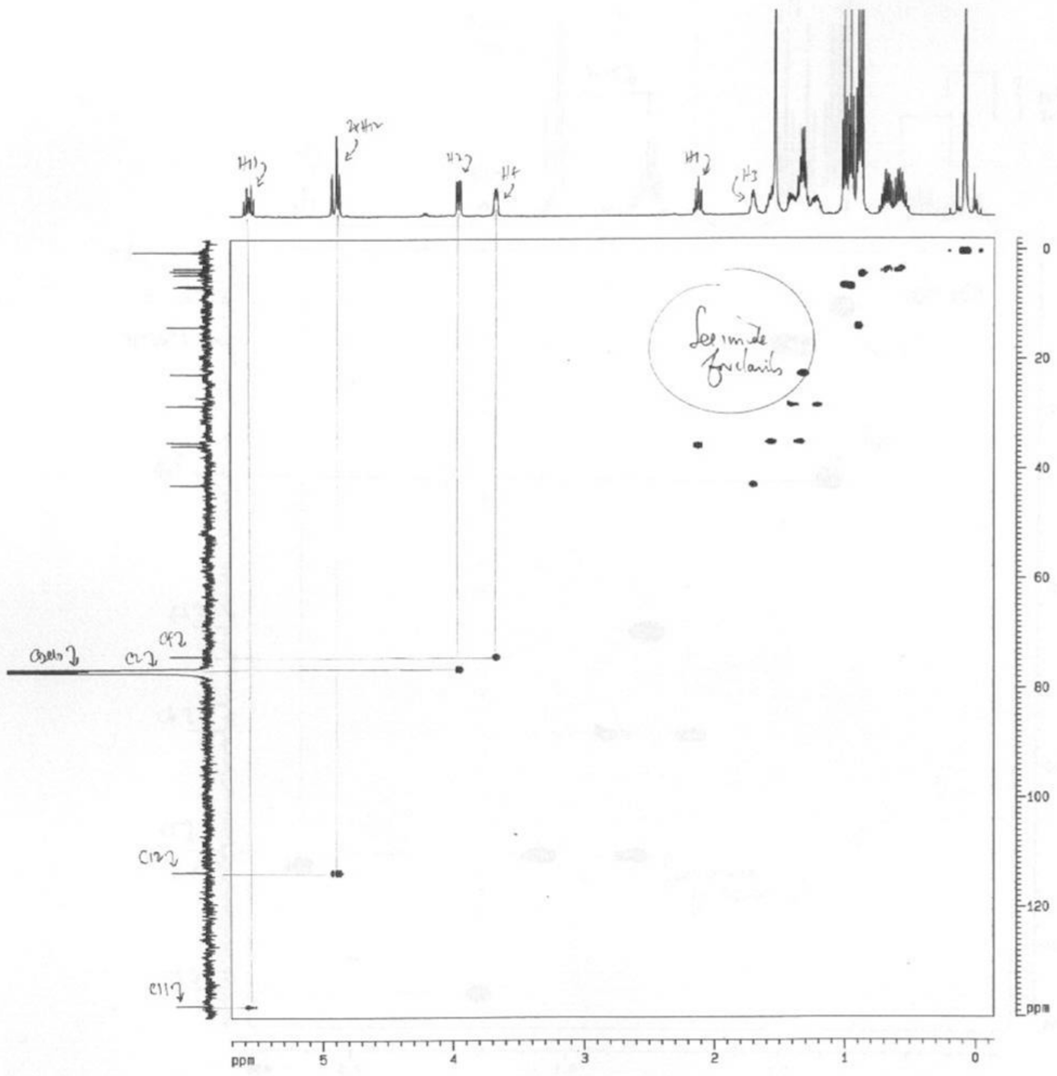

Current Data Parameters

NAME	dc07pjid
EXPNO	3
PROCNO	1

F2 - Acquisition Parameters

Date_	20051207
Time	16.32
INSTRUM	drx500
PROBHD	5 mm TBI H/C
PULPROG	invigptp
TD	2048
SOLVENT	CDCl3
NS	8
DS	16
SWH	4310.345 Hz
FIDRES	2.104661 Hz
AQ	0.2376180 sec
RG	32768
DW	116.000 usec
DE	5.50 usec
TE	300.0 K
CNST2	145.0000000
d0	0.00000300 sec
D1	2.00000000 sec
d4	0.00172414 sec
d11	0.03000000 sec
d13	0.00000300 sec
D16	0.00010000 sec
d20	0.00110000 sec
d21	0.00081714 sec
IN0	0.00001140 sec

============ CHANNEL f1 ============

NUC1	1H
P1	10.70 usec
p2	21.40 usec
PL1	1.00 dB
SFO1	500.1318867 MHz

============ CHANNEL f2 ============

CPDPRG2	garp
NUC2	13C
P3	12.00 usec
p4	24.00 usec
PCPD2	78.00 usec
PL2	-1.00 dB
PL12	15.00 dB
SFO2	125.7667893 MHz

============ GRADIENT CHANNEL ========

GPNAM1	SINE.100
GPNAM2	SINE.100
GPNAM3	SINE.100
GPX1	0.00 %
GPX2	0.00 %
GPX3	0.00 %
GPY1	0.00 %
GPY2	0.00 %
GPY3	0.00 %
GPZ1	80.00 %
GPZ2	30.00 %
GPZ3	20.10 %
P16	1000.00 usec

F1 - Acquisition parameters

ND0	4
TD	512
SFO1	125.7668 MHz
FIDRES	42.831688 Hz
SW	174.369 ppm

F2 - Processing parameters

SI	2048
SF	500.1300233 MHz
WDW	QSINE
SSB	2
LB	0.00 Hz
GB	0
PC	1.00

F1 - Processing parameters

SI	1024
MC2	TPPI
SF	125.7577910 MHz
WDW	QSINE
SSB	2
LB	0.00 Hz
GB	0

2D NMR plot parameters

CX2	17.00 cm
CX1	17.00 cm
F2PLO	5.708 ppm
F2LO	2854.76 Hz
F2PHI	-0.141 ppm
F2HI	-70.72 Hz
F1PLO	140.182 ppm
F1LO	17628.92 Hz
F1PHI	-2.014 ppm
F1HI	-253.31 Hz
F2PPMCM	0.34408 ppm/cm
F2HZCM	172.08694 Hz/cm
F1PPMCM	8.36446 ppm/cm
F1HZCM	1051.89587 Hz/cm

19a

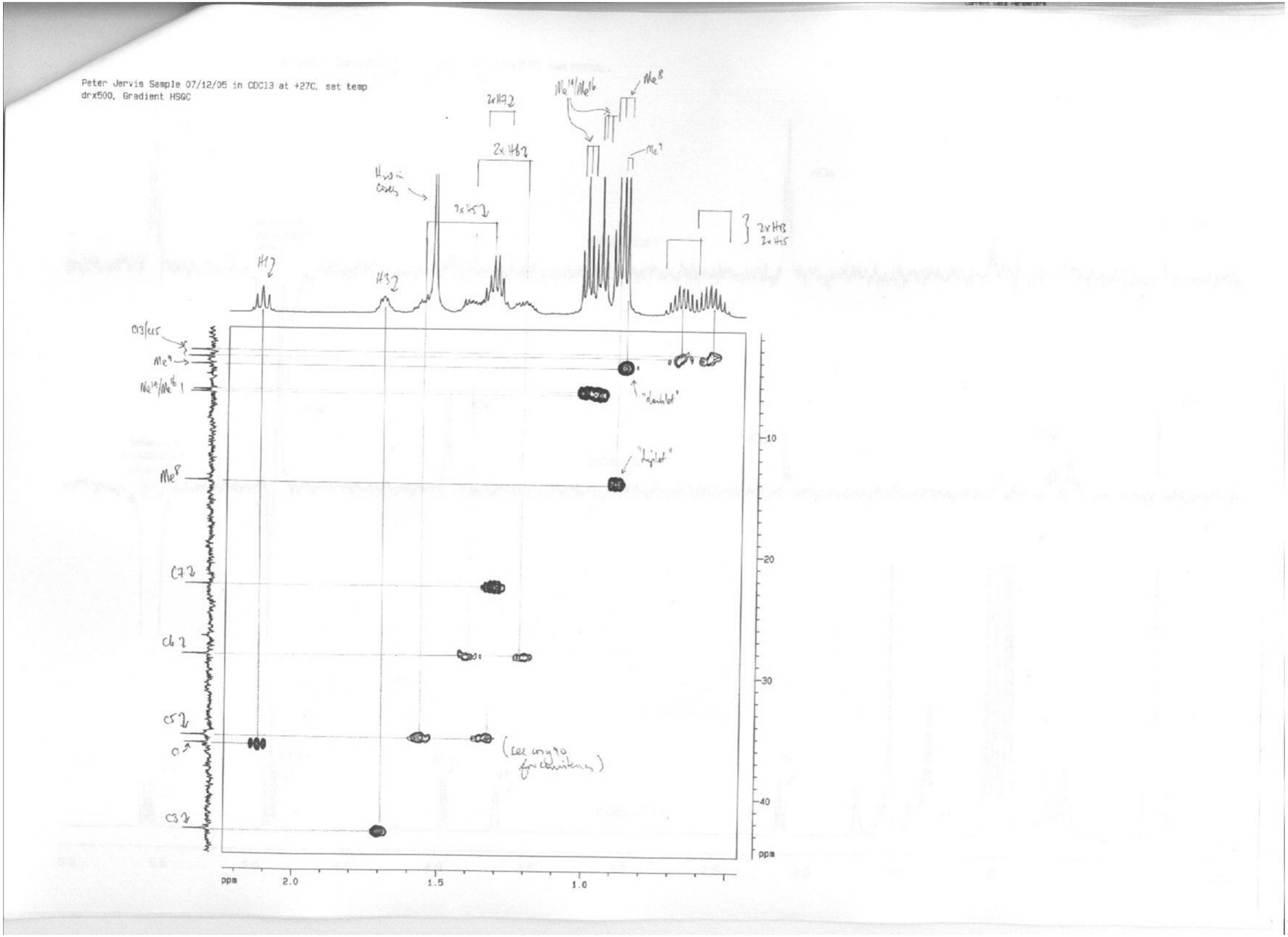
Peter Jervis Sample 07/12/05 in CDCl3 at +27C, set temp
drx500, Gradient HSGC
ppm
2.0
1.5
1.0
10
20
30
40
ppm

19a

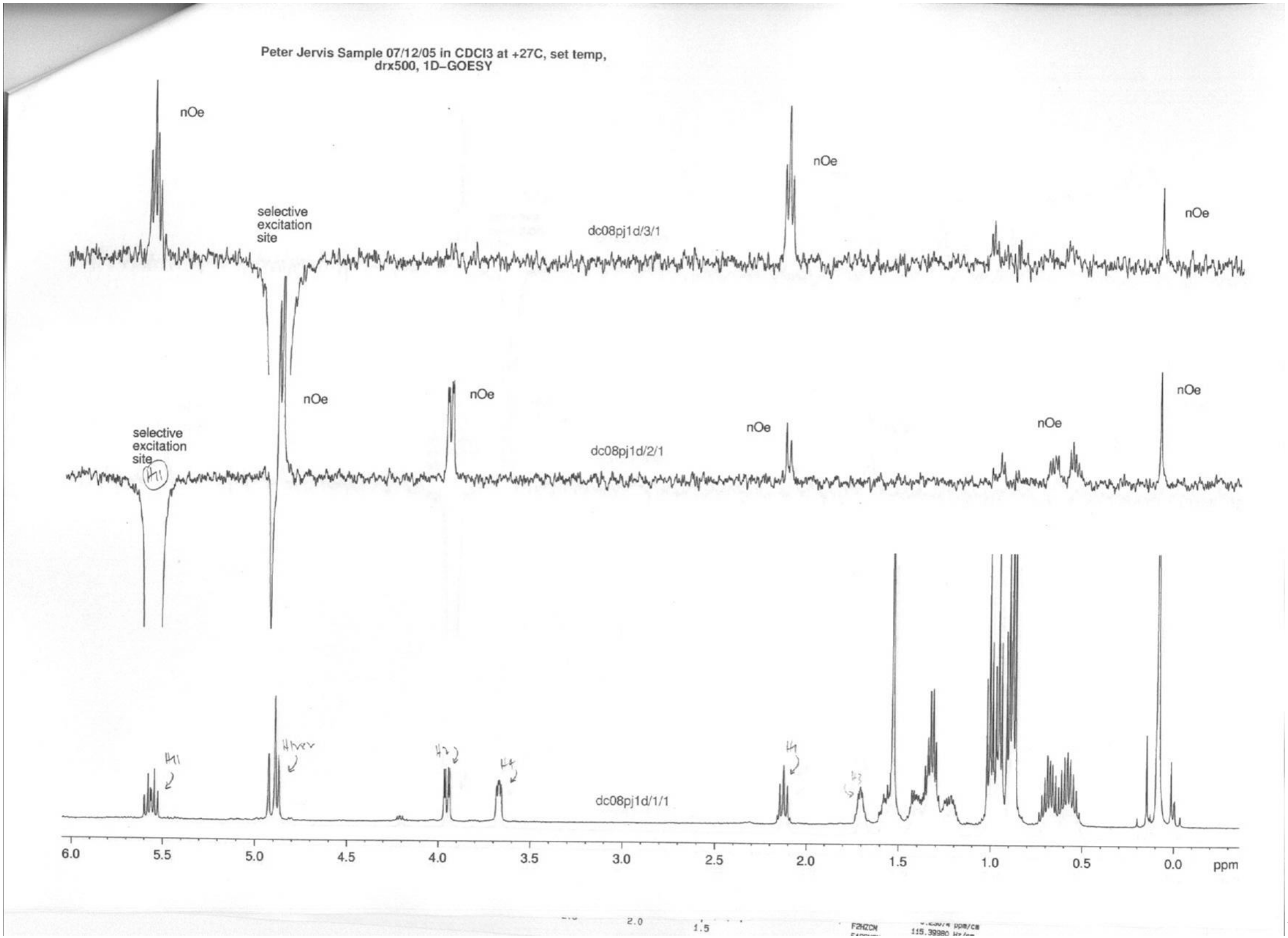
Peter Jervis Sample 07/12/05 in CDCl3 at +27C, set temp,
drx500, 1D–GOESY
nOe
selective
excitation
site
dc08pj1d/3/1
dc08pj1d/2/1
dc08pj1d/1/1
ppm

19a

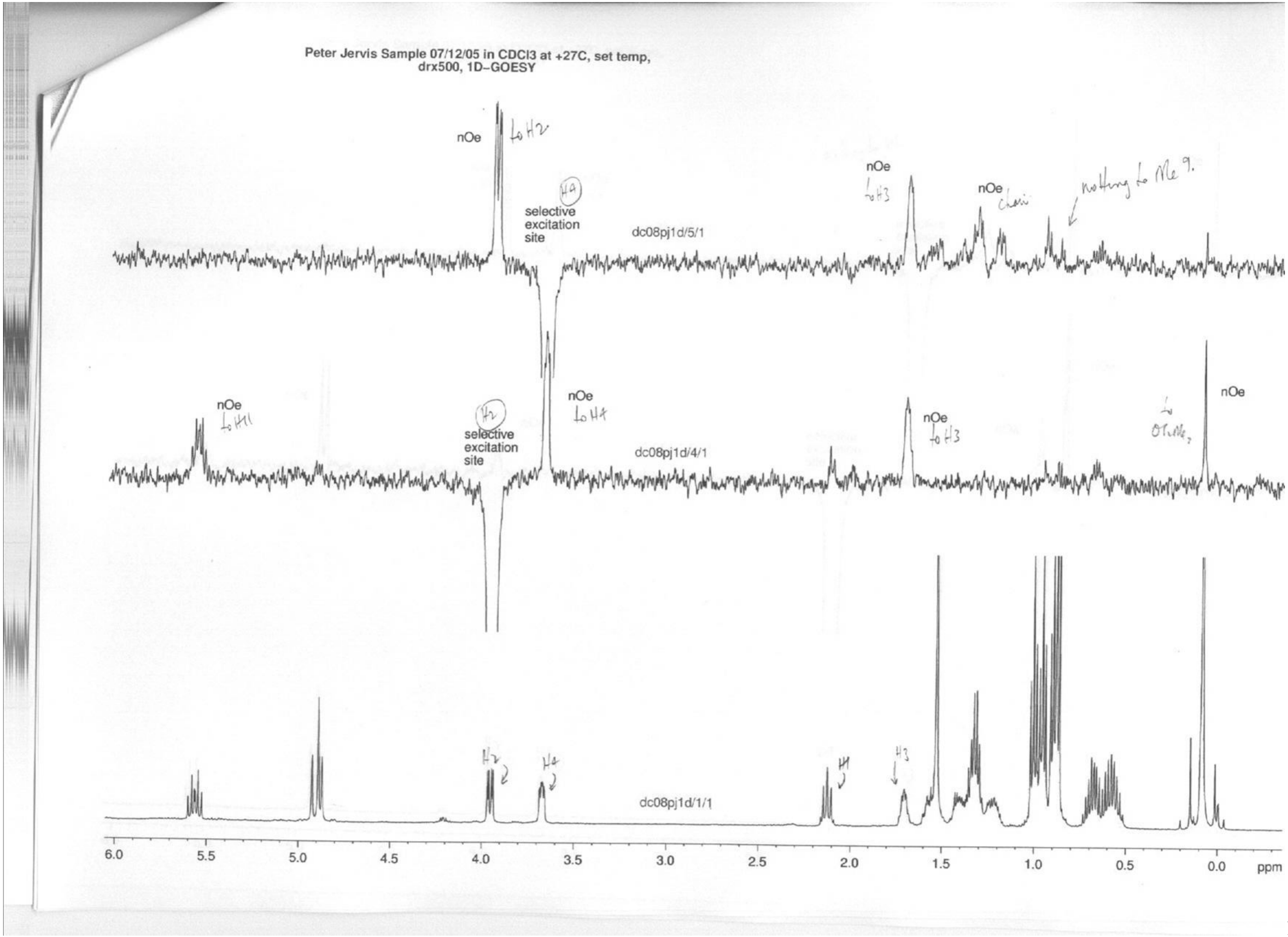
Peter Jervis Sample 07/12/05 in CDCl3 at +27C, set temp,
drx500, 1D-GOESY
nOe
to H2
H4
selective
excitation
site
dc08pj1d/5/1
nOe
to H3
nOe
nothing to Me 9.
nOe
to H1
H2
selective
excitation
site
nOe
to H4
dc08pj1d/4/1
nOe
to H3
nOe
H2
H4
dc08pj1d/1/1
H1
H3
6.0
5.5
5.0
4.5
4.0
3.5
3.0
2.5
2.0
1.5
1.0
0.5
0.0
ppm

19a

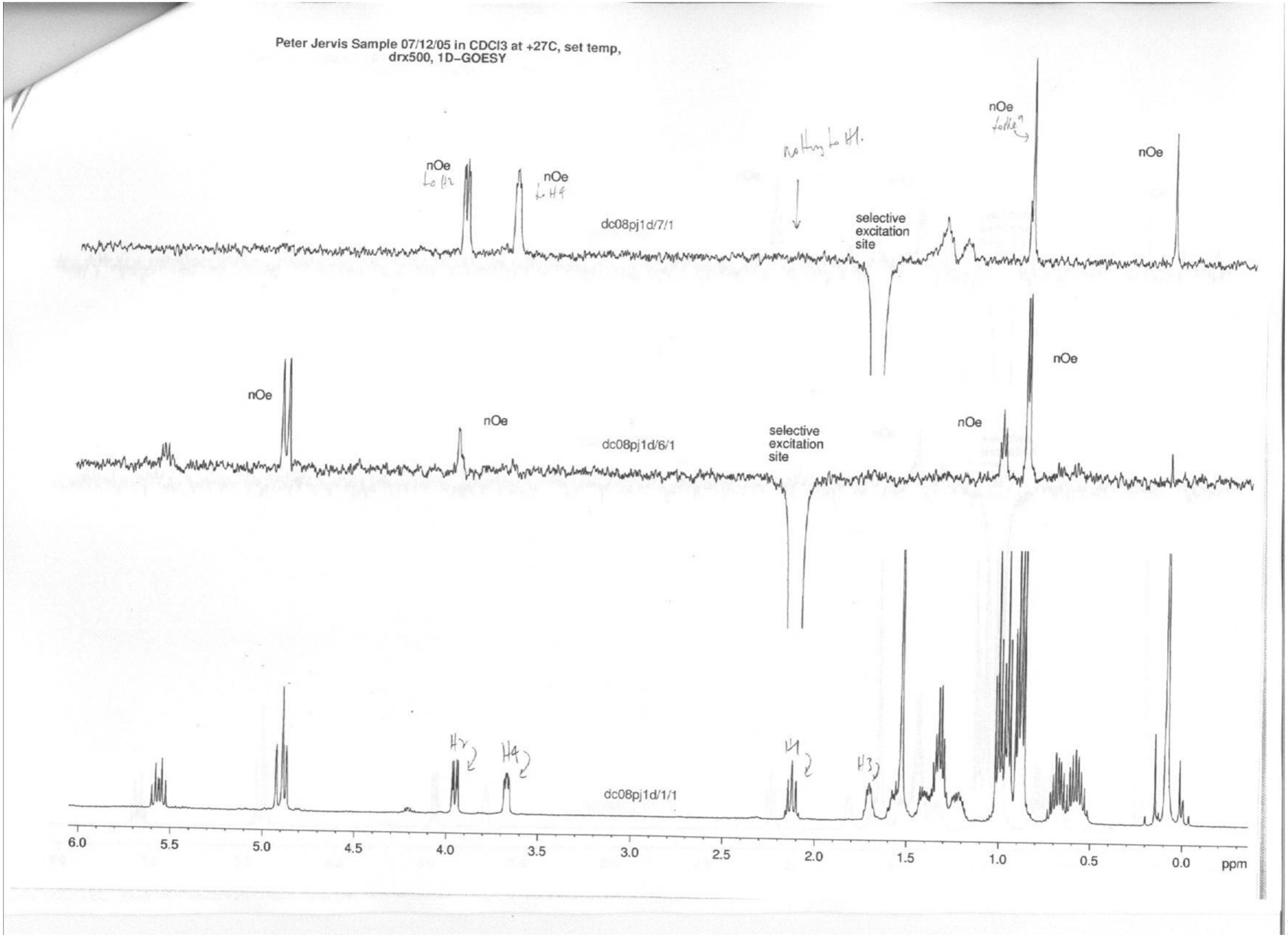
Peter Jervis Sample 07/12/05 in CDCl3 at +27C, set temp, drx500, 1D-GOESY
nOe
nOe
nOe
nOe
selective excitation site
dc08pj1d/7/1
nOe
nOe
nOe
nOe
selective excitation site
dc08pj1d/6/1
dc08pj1d/1/1
6.0
5.5
5.0
4.5
4.0
3.5
3.0
2.5
2.0
1.5
1.0
0.5
0.0
ppm

19a

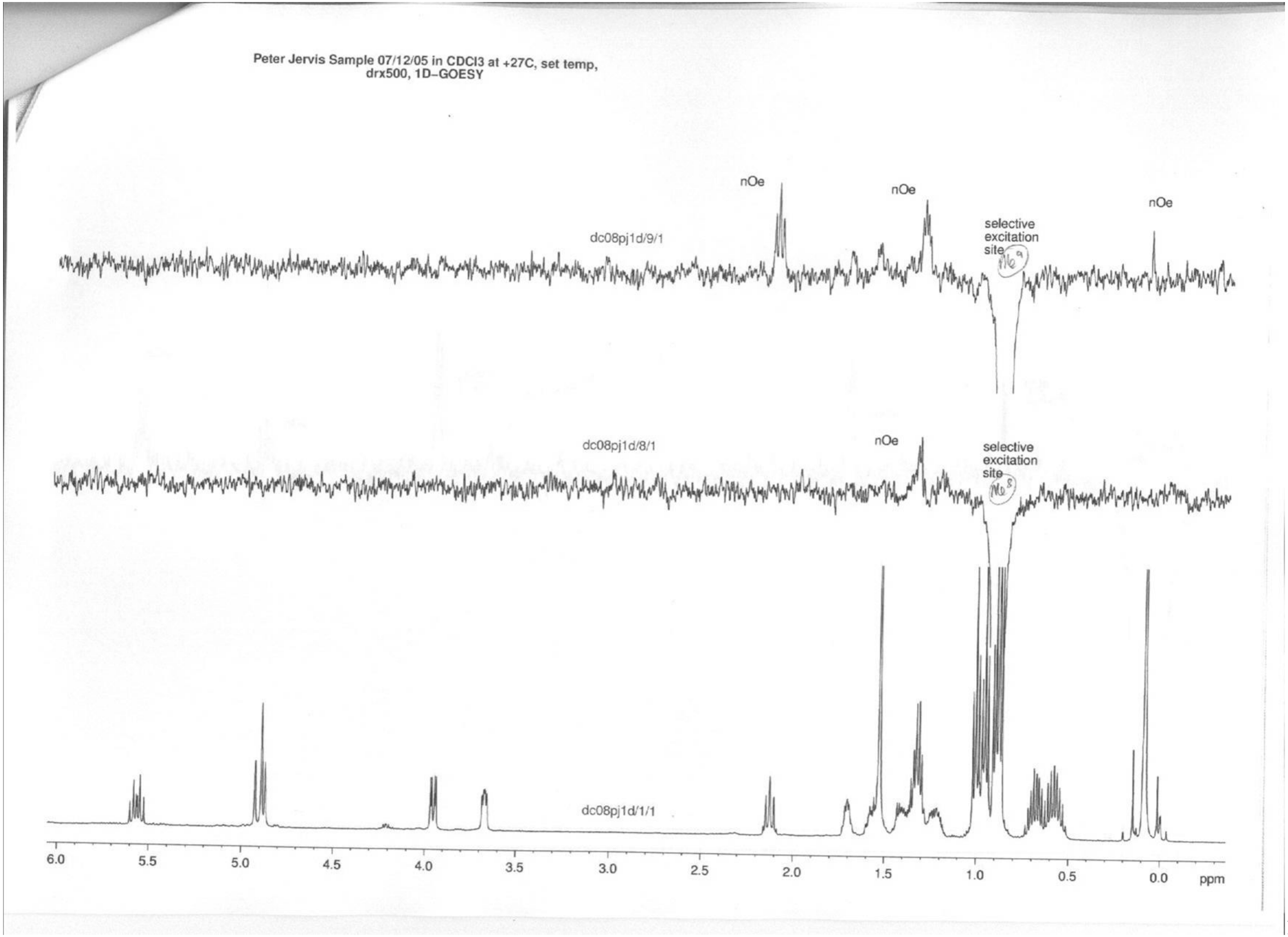
Peter Jervis Sample 07/12/05 in CDCl3 at +27C, set temp,
drx500, 1D-GOESY
nOe
nOe
nOe
dc08pj1d/9/1
selective
excitation
site
dc08pj1d/8/1
nOe
selective
excitation
site
dc08pj1d/1/1
6.0
5.5
5.0
4.5
4.0
3.5
3.0
2.5
2.0
1.5
1.0
0.5
0.0
ppm

19a

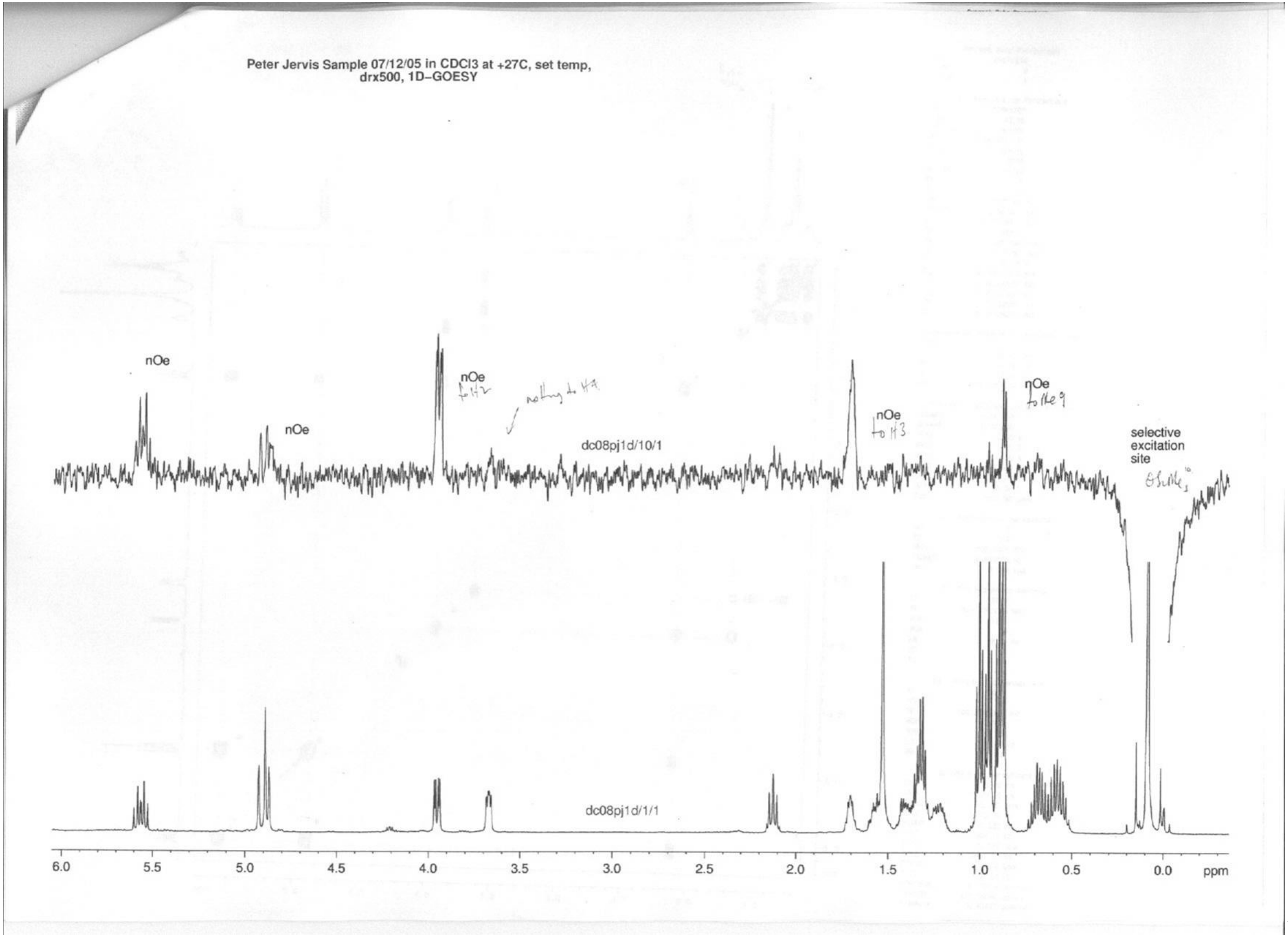

Peter Jervis Sample 07/12/05 in CDCl3 at +27C, set temp,
drx500, 1D–GOESY
nOe
nOe
nOe
nOe
nOe
selective
excitation
site
dc08pj1d/10/1
dc08pj1d/1/1
6.0
5.5
5.0
4.5
4.0
3.5
3.0
2.5
2.0
1.5
1.0
0.5
0.0
ppm

19a
