# Supplementary material for: Tether-directed synthesis of highly substituted oxasilacycles via an intramolecular allylation employing allylsilanes
Source: Beilstein J Org Chem. 2007 Feb 8;3:6. doi: 10.1186/1860-5397-3-6 (PMC1810303; doi:10.1186/1860-5397-3-6)
Supplement: File 5 — 1H-NMR and 13C-NMR Spectra for the following compounds: 14a, 15a. [file Beilstein_J_Org_Chem-03-06-s005.pdf]

Additional File 5

Tether-directed synthesis of highly substituted oxasilacycles *via* an intramolecular allylation employing allylsilanes

Peter J. Jervis and Liam R. Cox*

email: l.r.cox@bham.ac.uk

^{1}H-NMR and ^{13}C-NMR Spectra for the following compounds:

14a, 15a

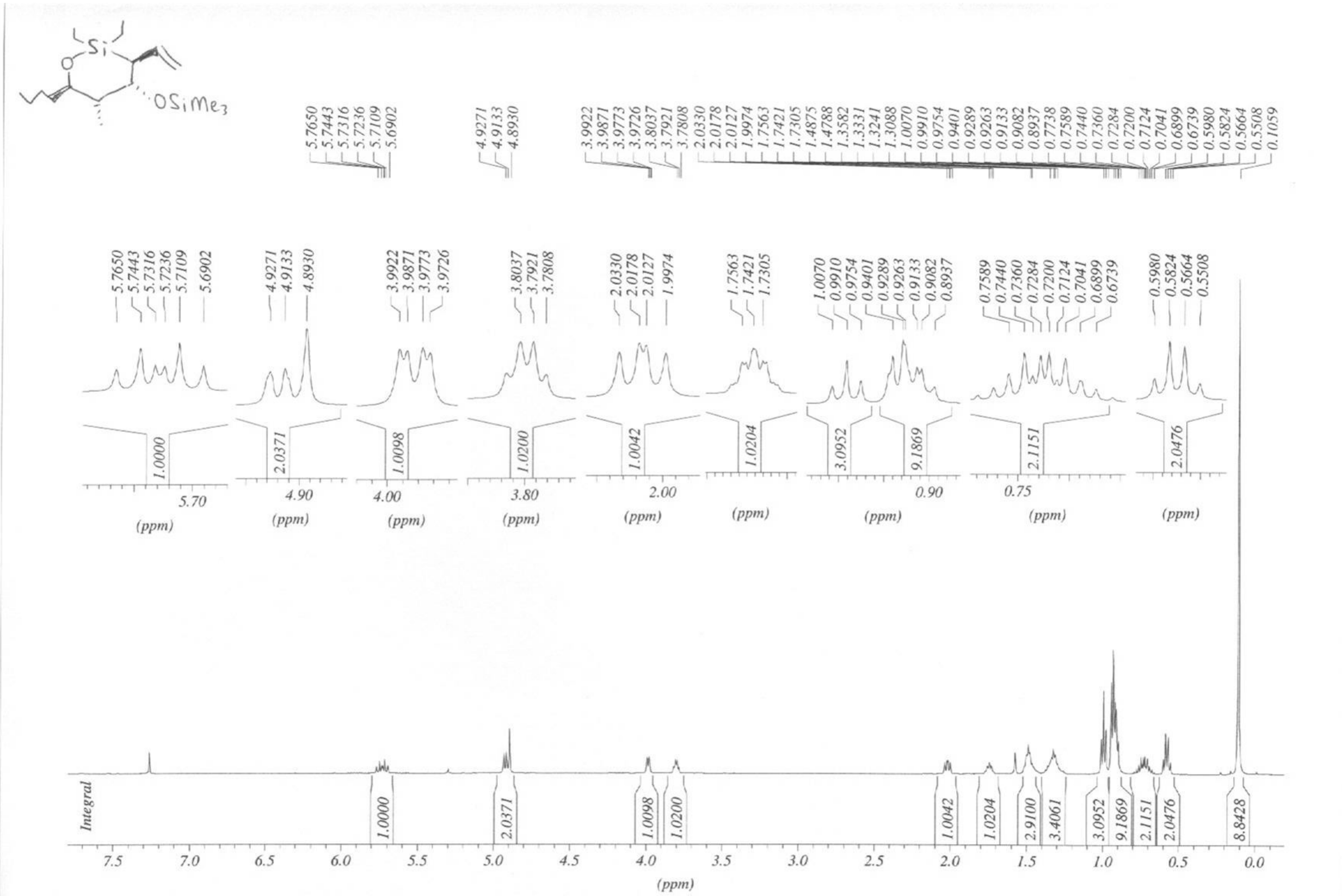
OSiMe3
Si
O
5.7650 5.7443 5.7316 5.7236 5.7109 5.6902
4.9271 4.9133 4.8930
3.9922 3.9871 3.9773 3.9726 3.8037 3.7921 3.7808
2.0330 2.0178 2.0127 1.9974 1.7563 1.7421 1.7305 1.4875 1.4788 1.3582 1.3331 1.3241 1.3088 1.0070 0.9910 0.9754 0.9401 0.9289 0.9263 0.9133 0.9082 0.8937 0.7738 0.7589 0.7440 0.7360 0.7284 0.7200 0.7124 0.7041 0.6899 0.6739 0.5980 0.5824 0.5664 0.5508 0.1059
1.0000
2.0371
1.0098
1.0200
1.0042
1.0204
3.0952
9.1869
2.1151
2.0476
5.70
4.90
4.00
3.80
2.00
0.90
0.75
(ppm)
Integral
1.0000 2.0371 1.0098 1.0200 1.0042 1.0204 2.9100 3.4061 3.0952 9.1869 2.1151 2.0476 8.8428
7.5 7.0 6.5 6.0 5.5 5.0 4.5 4.0 3.5 3.0 2.5 2.0 1.5 1.0 0.5 0.0
(ppm)

14a

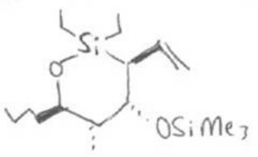
Si
O
OSiMe3

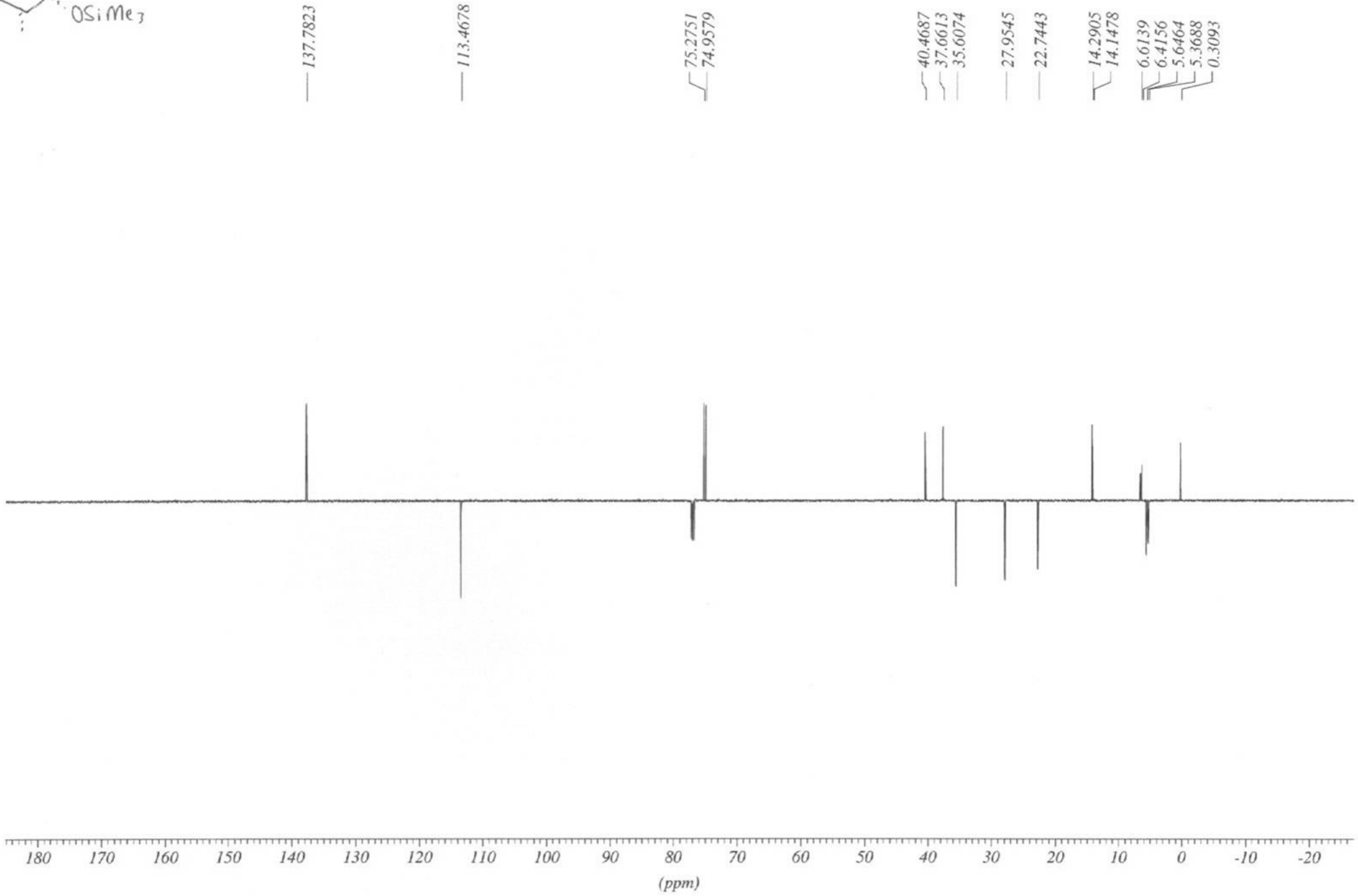
137.7823
113.4678
75.2751
74.9579
40.4687
37.6613
35.6074
27.9545
22.7443
14.2905
14.1478
6.6139
6.4156
5.6464
5.3688
0.3093
180 170 160 150 140 130 120 110 100 90 80 70 60 50 40 30 20 10 0 -10 -20
(ppm)

14a

Peter Jervis Sample 8, 13/12/05 in CDCl3 at +27C, set temp
drx500, Gradient COSY90

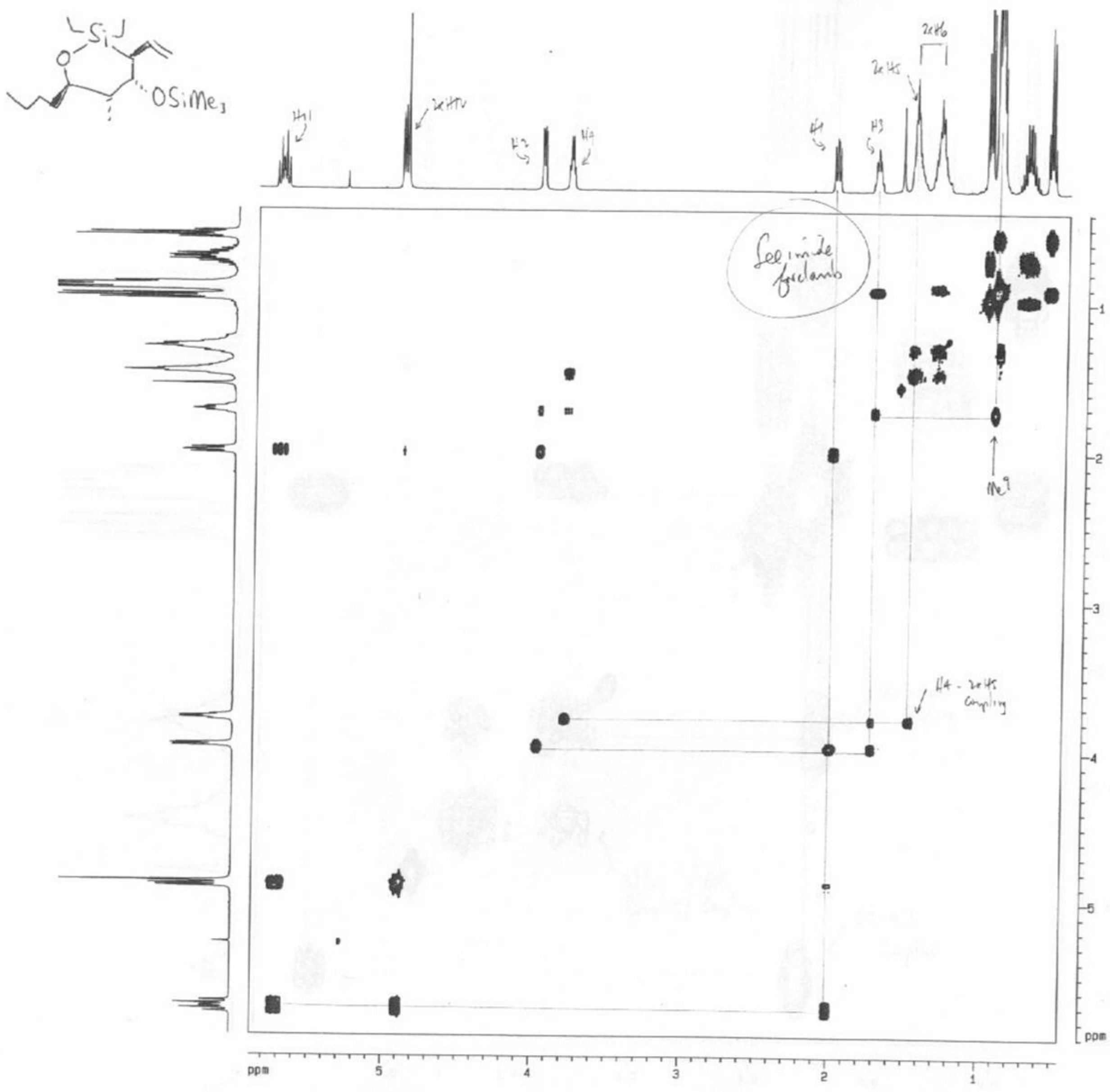

**Current Data Parameters**	
NAME	dc13pj1d
EXPNO	4
PROCNO	1
**F2 - Acquisition Parameters**	
Date_	20051212
Time	22.50
INSTRUM	drx500
PROBHD	5 mm TBI H/C
PULPROG	cosygp
TD	2048
SOLVENT	CDCl3
NS	8
DS	16
SWH	4310.345 Hz
FIDRES	2.104661 Hz
AQ	0.2376180 sec
RG	57
DW	116.000 usec
DE	5.50 usec
TE	300.0 K
d0	0.00000300 sec
D1	2.00000000 sec
d13	0.00000300 sec
D16	0.00010000 sec
IN0	0.00023200 sec
**======== CHANNEL f1 ========**	
NUC1	1H
P0	10.70 usec
P1	10.70 usec
PL1	1.00 dB
SFO1	500.1318867 MHz
**======== GRADIENT CHANNEL ========**	
GPNAM1	SINE.100
GPNAM2	SINE.100
GPX1	0.00 %
GPX2	0.00 %
GPY1	0.00 %
GPY2	0.00 %
GPZ1	10.00 %
GPZ2	10.00 %
P16	1000.00 usec
**F1 - Acquisition parameters**	
ND0	1
TD	512
SFO1	500.1319 MHz
FIDRES	8.418642 Hz
SW	8.618 ppm
**F2 - Processing parameters**	
SI	2048
SF	500.1300233 MHz
WDW	SINE
SSB	0
LB	0.00 Hz
GB	0
PC	1.00
**F1 - Processing parameters**	
SI	1024
MC2	QF
SF	500.1300233 MHz
WDW	SINE
SSB	0
LB	0.00 Hz
GB	0
**2D NMR plot parameters**	
CX2	20.00 cm
CX1	20.00 cm
F2PLO	5.881 ppm
F2LO	2941.05 Hz
F2PHI	0.444 ppm
F2HI	221.82 Hz
F1PLO	5.906 ppm
F1LO	2953.67 Hz
F1PHI	0.393 ppm
F1HI	196.57 Hz
F2PPMCM	0.27185 ppm/cm
F2HZCM	135.96109 Hz/cm
F1PPMCM	0.27564 ppm/cm
F1HZCM	137.85527 Hz/cm

14a

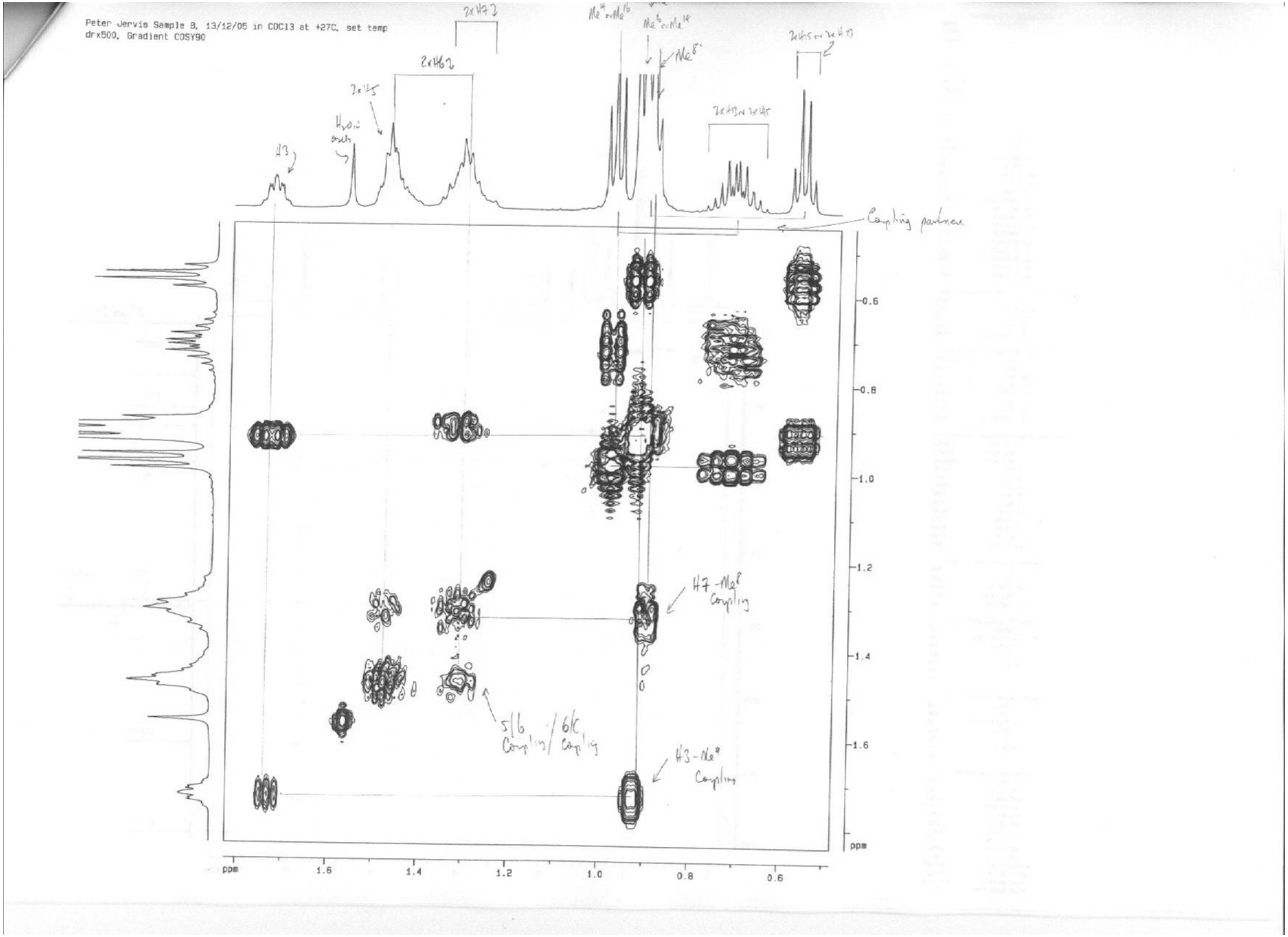
Peter Jervis Sample B, 13/12/05 in CDCl3 at +27C, set temp
drx500, Gradient COSY90
H3
2xH5
Coupling partners
H7-Me8 Coupling
5/6 Coupling / 6/C Coupling
H3-Me9 Coupling
ppm
1.6
1.4
1.2
1.0
0.8
0.6

14a

Peter Jervis Sample B, 13/12/05 in CDCl3 at +27C, set temp
drx500, Gradient HSQC

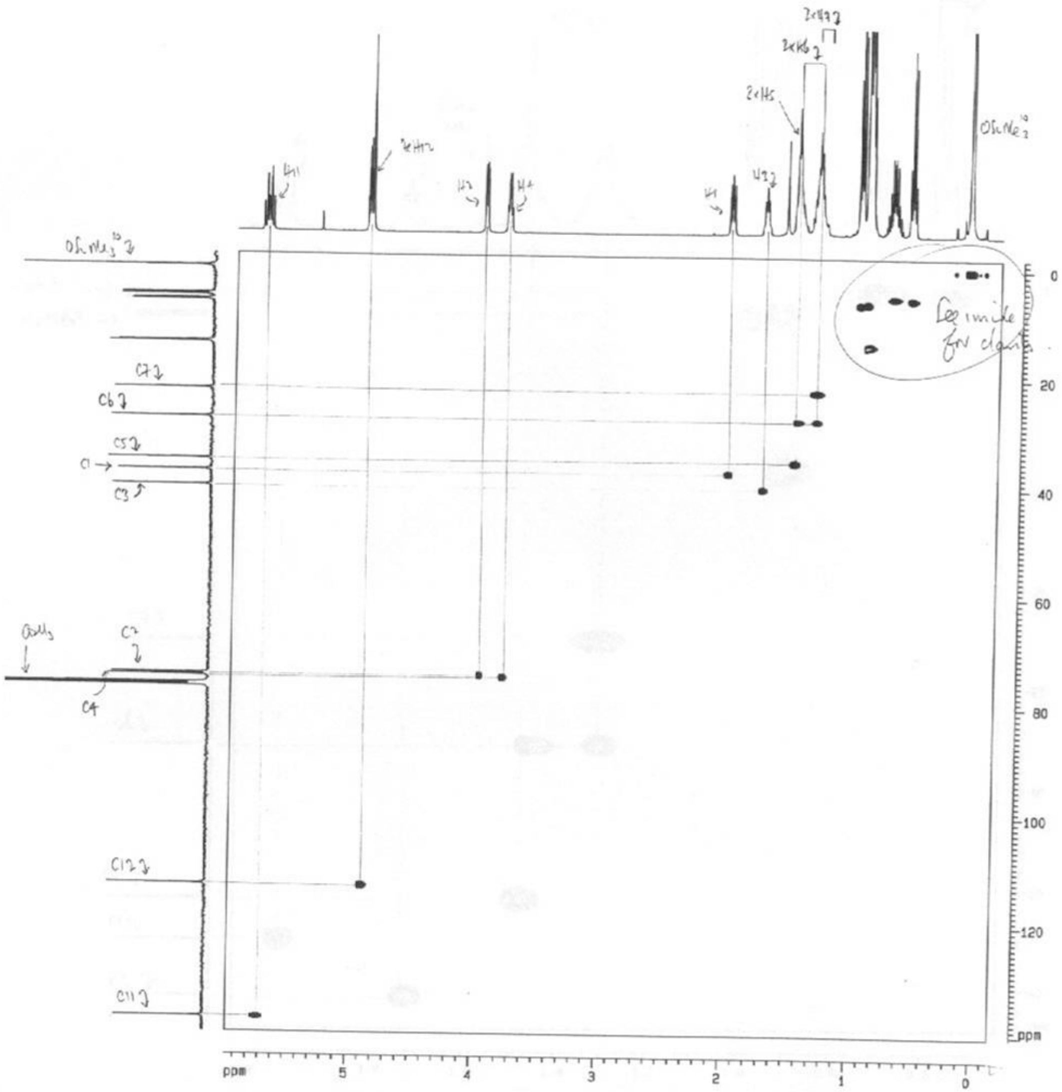

Current Data Parameters

NAME	dc13pjid
EXPNO	2
PROCNO	1

F2 - Acquisition Parameters

Date_	20051212
Time	17.34
INSTRUM	drx500
PROBHD	5 mm TBI H/C
PULPROG	invigptp
TD	2048
SOLVENT	CDCl3
NS	8
DS	16
SWH	4310.345 Hz
FIDRES	2.104661 Hz
AQ	0.2376180 sec
RG	32768
DW	116.000 usec
DE	5.50 usec
TE	300.0 K
CNST2	145.0000000
d0	0.00000300 sec
D1	2.00000000 sec
d4	0.00172414 sec
d11	0.03000000 sec
d13	0.00000300 sec
D16	0.00010000 sec
d20	0.00110000 sec
d21	0.00061714 sec
IN0	0.00001140 sec

============ CHANNEL f1 ============

NUC1	1H
P1	10.70 usec
p2	21.40 usec
PL1	1.00 dB
SFO1	500.1318667 MHz

============ CHANNEL f2 ============

CPDPRG2	garp
NUC2	13C
P3	12.00 usec
p4	24.00 usec
PCPD2	76.00 usec
PL2	-1.00 dB
PL12	15.00 dB
SFO2	125.7667893 MHz

============ GRADIENT CHANNEL ======

GPNAM1	SINE.100
GPNAM2	SINE.100
GPNAM3	SINE.100
GPX1	0.00 %
GPX2	0.00 %
GPX3	0.00 %
GPY1	0.00 %
GPY2	0.00 %
GPY3	0.00 %
GPZ1	80.00 %
GPZ2	30.00 %
GPZ3	20.10 %
P16	1000.00 usec

F1 - Acquisition parameters

ND0	4
TD	512
SFO1	125.7668 MHz
FIDRES	42.831688 Hz
SW	174.369 ppm

F2 - Processing parameters

SI	2048
SF	500.1300233 MHz
WDW	QSINE
SSB	2
LB	0.00 Hz
GB	0
PC	1.00

F1 - Processing parameters

SI	1024
MC2	TPPI
SF	125.7577910 MHz
WDW	QSINE
SSB	2
LB	0.00 Hz
GB	0

2D NMR plot parameters

CX2	17.00 cm
CX1	17.00 cm
F2PLO	5.985 ppm
F2LO	2983.14 Hz
F2PHI	-0.154 ppm
F2HI	-77.04 Hz
F1PLO	140.522 ppm
F1LO	17671.75 Hz
F1PHI	-2.014 ppm
F1HI	-253.31 Hz
F2PPMCM	0.35893 ppm/cm
F2HZCM	180.01039 Hz/cm
F1PPMCM	8.38449 ppm/cm
F1HZCM	1054.41563 Hz/cm

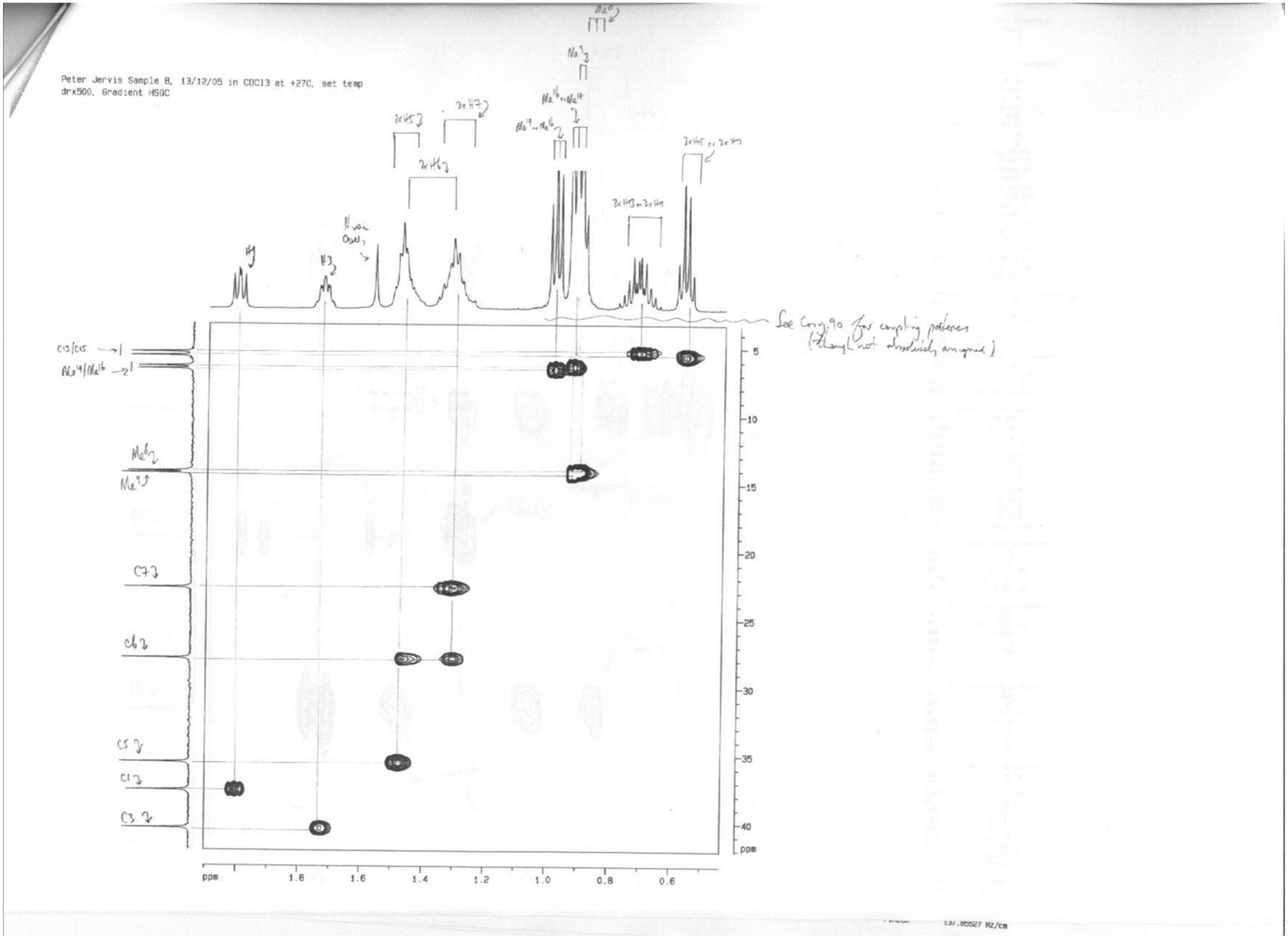
Peter Jervis Sample B, 13/12/05 in CDCl3 at +27C, set temp
drx500, Gradient HSQC
ppm
1.8
1.6
1.4
1.2
1.0
0.8
0.6
5
10
15
20
25
30
35
40
ppm
C13/C15
C7
C6
C5
C1
C3

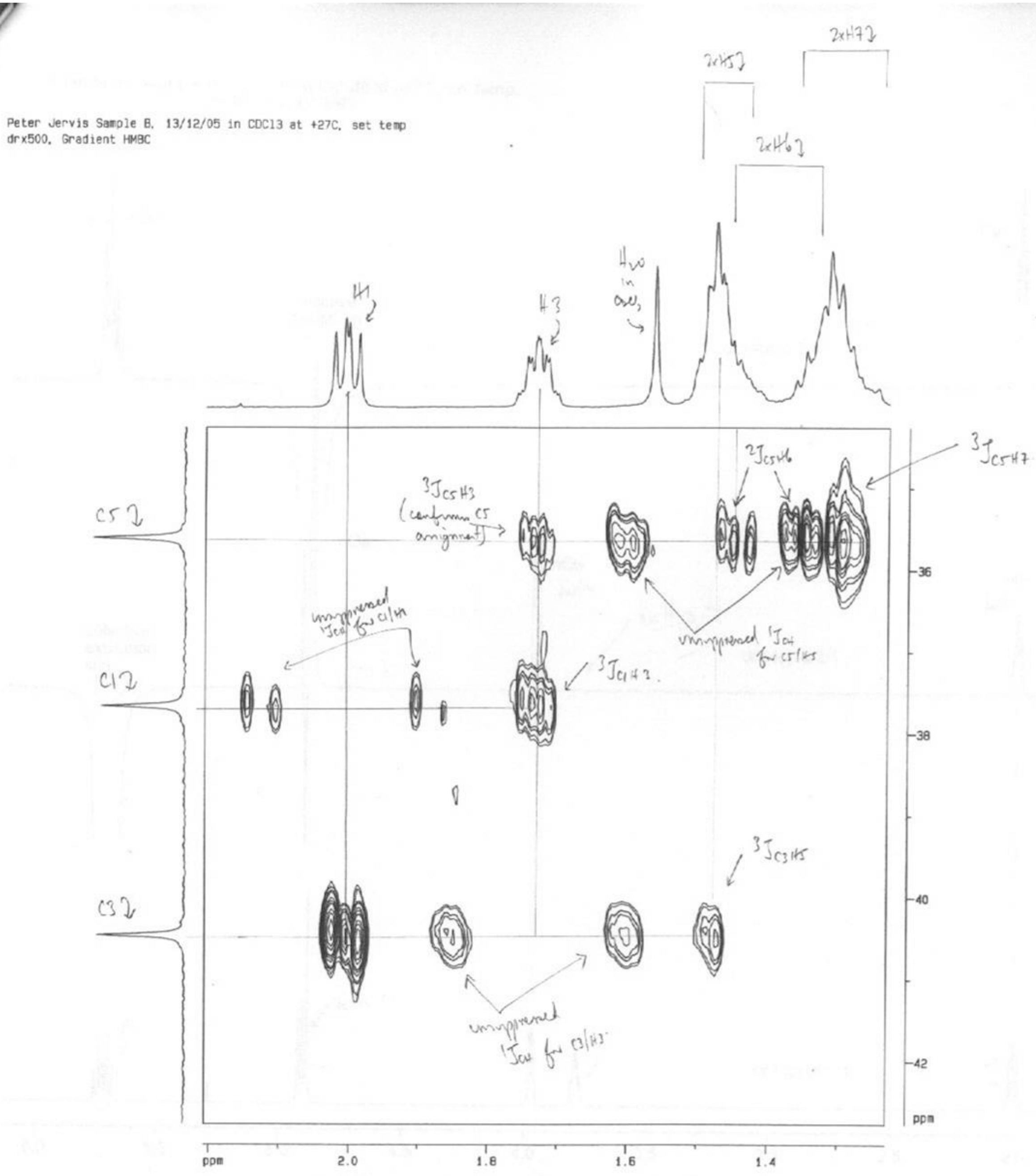

Current Data Parameters

NAME	dc13pj1d
EXPNO	3
PROCNO	1

F2 - Acquisition Parameters

Date_	20051212
Time	20.09
INSTRUM	drx500
PROBHD	5 mm TBI H/C
PULPROG	inv4gplplrnd
TD	2048
SOLVENT	CDCl3
NS	8
DS	16
SWH	4310.345 Hz
FIDRES	2.104661 Hz
AQ	0.2376180 sec
RG	32768
DW	116.000 usec
DE	5.50 usec
TE	300.0 K
CNST2	160.0000000
d0	0.00000300 sec
D1	2.00000000 sec
d2	0.00312500 sec
D6	0.10000000 sec
d13	0.00000300 sec
D16	0.00010000 sec
IN0	0.00002280 sec

======== CHANNEL f1 ========

NUC1	1H
P1	10.70 usec
p2	21.40 usec
PL1	1.00 dB
SFO1	500.1318867 MHz

======== CHANNEL f2 ========

NUC2	13C
P3	12.00 usec
PL2	-1.00 dB
SFO2	125.7667893 MHz

======== GRADIENT CHANNEL ========

GPNAM1	SINE.100
GPNAM2	SINE.100
GPNAM3	SINE.100
GPX1	0.00 %
GPX2	0.00 %
GPX3	0.00 %
GPY1	0.00 %
GPY2	0.00 %
GPY3	0.00 %
GPZ1	50.00 %
GPZ2	30.00 %
GPZ3	40.10 %
P16	1000.00 usec

F1 - Acquisition parameters

ND0	2
TD	512
SFO1	125.7668 MHz
FIDRES	42.831668 Hz
SW	174.369 ppm

F2 - Processing parameters

SI	2048
SF	500.1300233 MHz
WDW	QSINE
SSB	2
LB	0.00 Hz
GB	0
PC	0.20

F1 - Processing parameters

SI	1024
MC2	QF
SF	125.7577910 MHz
WDW	QSINE
SSB	2
LB	0.00 Hz
GB	0

2D NMR plot parameters

CX2	17.00 cm
CX1	17.00 cm
F2PLO	2.207 ppm
F2LO	1103.68 Hz
F2PHI	1.230 ppm
F2HI	615.40 Hz
F1PLO	42.773 ppm
F1LO	5379.06 Hz
F1PHI	34.258 ppm
F1HI	4308.26 Hz
F2PPMCM	0.05743 ppm/cm
F2HZCM	28.72243 Hz/cm
F1PPMCM	0.50087 ppm/cm
F1HZCM	62.98777 Hz/cm

14a

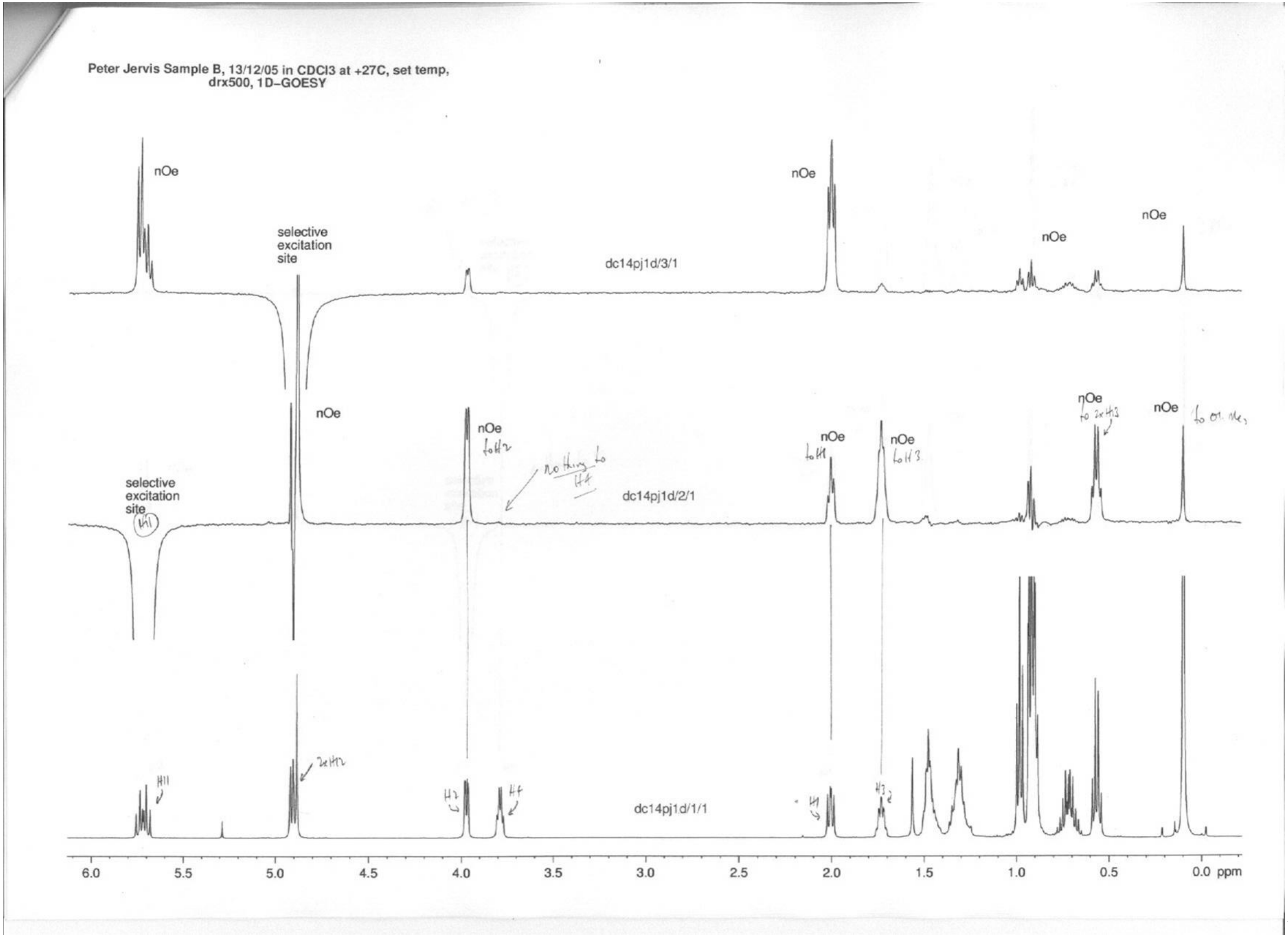
Peter Jervis Sample B, 13/12/05 in CDCl3 at +27C, set temp,
drx500, 1D-GOESY
nOe
selective
excitation
site
dc14pj1d/3/1
nOe
nOe
nOe
nOe
selective
excitation
site
nOe
nOe
dc14pj1d/2/1
nOe
nOe
nOe
nOe
dc14pj1d/1/1
6.0
5.5
5.0
4.5
4.0
3.5
3.0
2.5
2.0
1.5
1.0
0.5
0.0 ppm

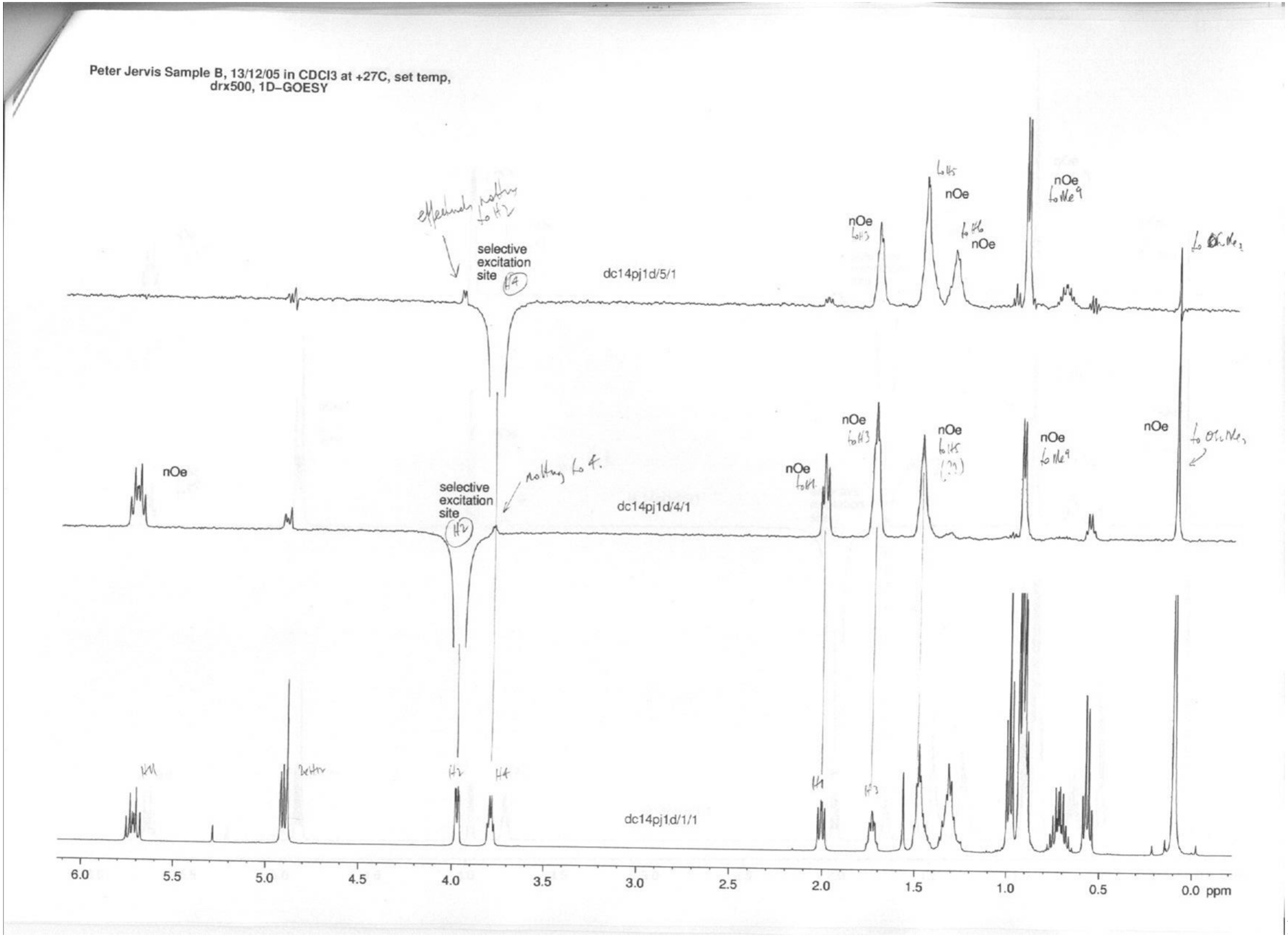
Peter Jervis Sample B, 13/12/05 in CDCl3 at +27C, set temp,
drx500, 1D–GOESY
selective excitation site
dc14pj1d/5/1
nOe
nOe
nOe
nOe
nOe
selective excitation site
dc14pj1d/4/1
nOe
nOe
nOe
nOe
nOe
nOe
dc14pj1d/1/1
6.0 5.5 5.0 4.5 4.0 3.5 3.0 2.5 2.0 1.5 1.0 0.5 0.0 ppm

14a

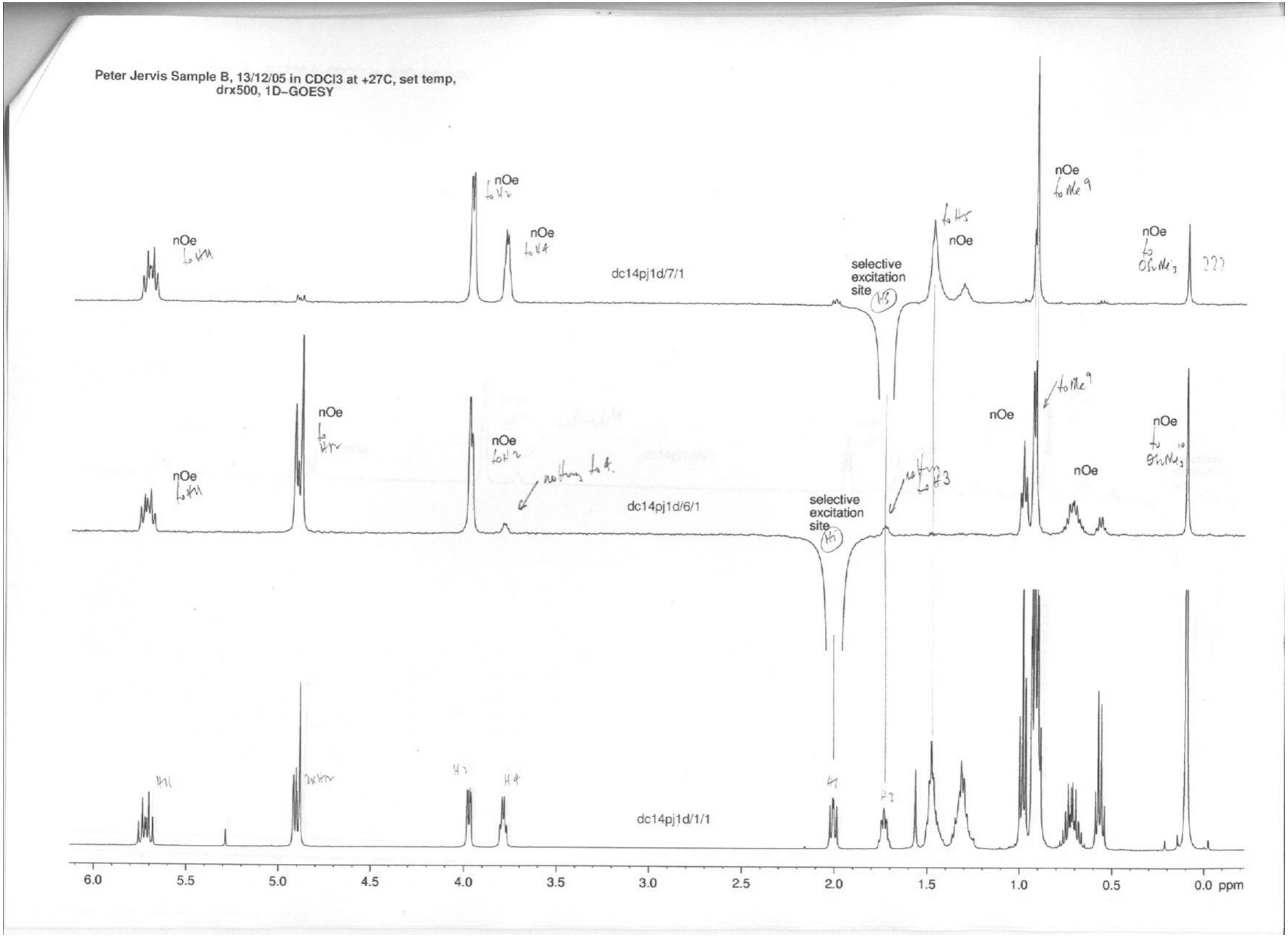
Peter Jervis Sample B, 13/12/05 in CDCl3 at +27C, set temp,
drx500, 1D-GOESY
nOe
dc14pj1d/7/1
selective
excitation
site
dc14pj1d/6/1
dc14pj1d/1/1
6.0
5.5
5.0
4.5
4.0
3.5
3.0
2.5
2.0
1.5
1.0
0.5
0.0 ppm

14a

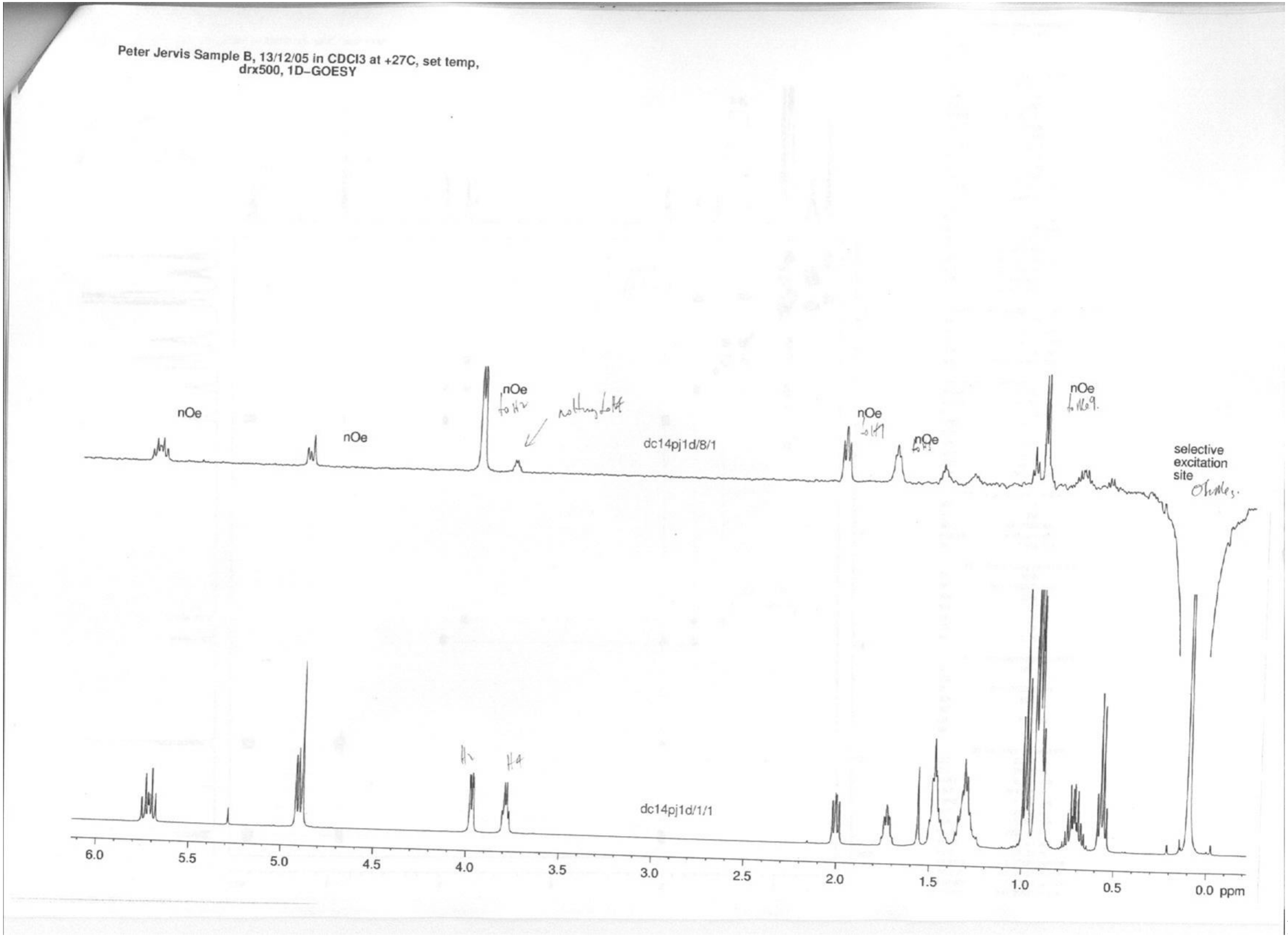
Peter Jervis Sample B, 13/12/05 in CDCl3 at +27C, set temp,
drx500, 1D–GOESY
nOe
nOe
nOe
nOe
nOe
nOe
dc14pj1d/8/1
selective
excitation
site
dc14pj1d/1/1
6.0
5.5
5.0
4.5
4.0
3.5
3.0
2.5
2.0
1.5
1.0
0.5
0.0 ppm

14a

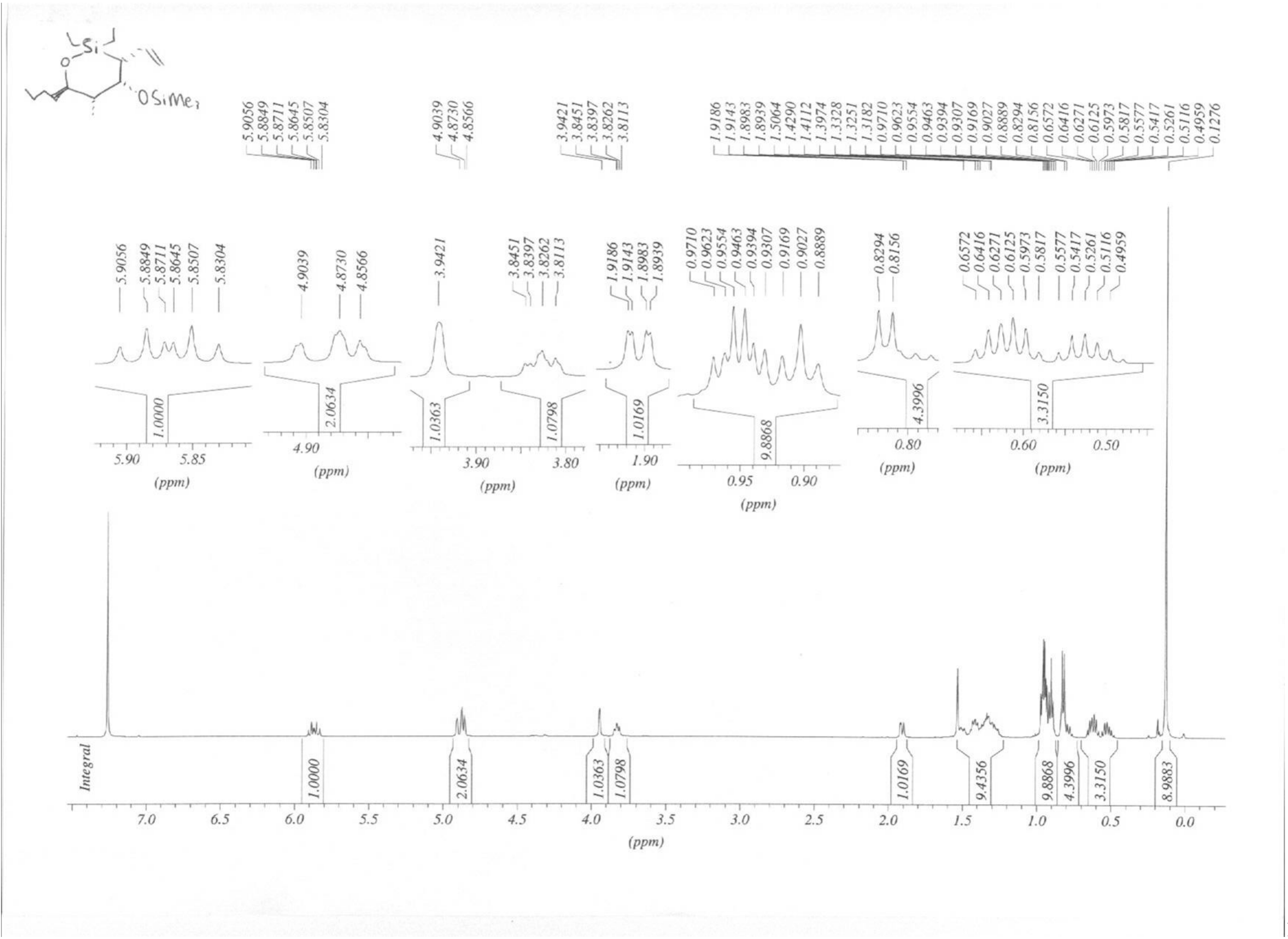
OSiMe3
5.9056 5.8849 5.8711 5.8645 5.8507 5.8304
4.9039 4.8730 4.8566
3.9421 3.8451 3.8397 3.8262 3.8113
1.9186 1.9143 1.8983 1.8939 1.5064 1.4290 1.4112 1.3974 1.3328 1.3251 1.3182 0.9710 0.9623 0.9554 0.9463 0.9394 0.9307 0.9169 0.9027 0.8889 0.8294 0.8156 0.6572 0.6416 0.6271 0.6125 0.5973 0.5817 0.5577 0.5417 0.5261 0.5116 0.4959 0.1276
1.0000
2.0634
1.0363
1.0798
1.0169
9.8868
4.3996
3.3150
9.4356
8.9883
Integral
(ppm)

15a

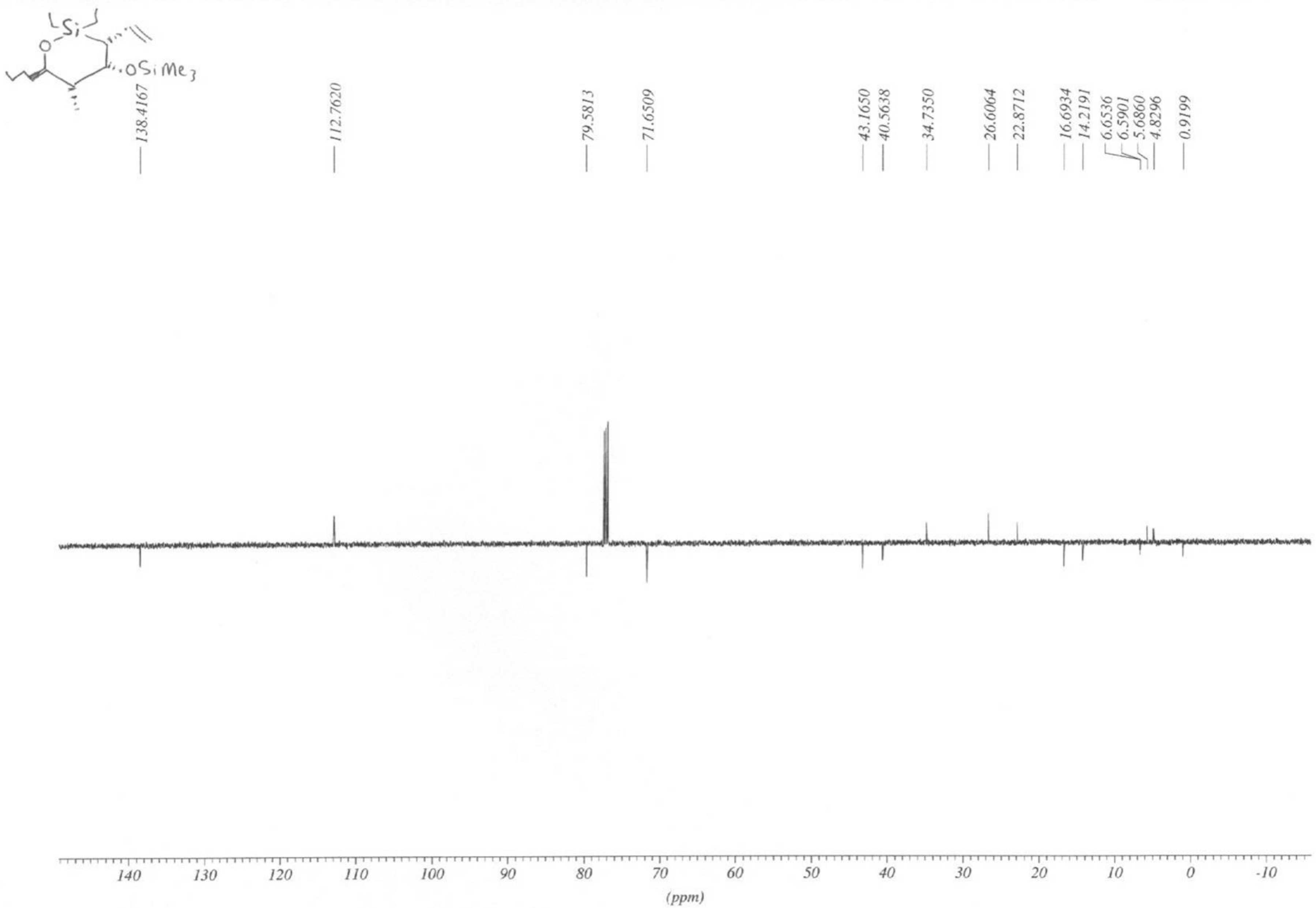
Si
O
OSiMe3
138.4167
112.7620
79.5813
71.6509
43.1650
40.5638
34.7350
26.6064
22.8712
16.6934
14.2191
6.6536
6.5901
5.6860
4.8296
0.9199
140 130 120 110 100 90 80 70 60 50 40 30 20 10 0 -10
(ppm)

15a

Peter Jervis 09/01/06 in CDCl3 at +27C, set temp
drx500, Gradient COSY90

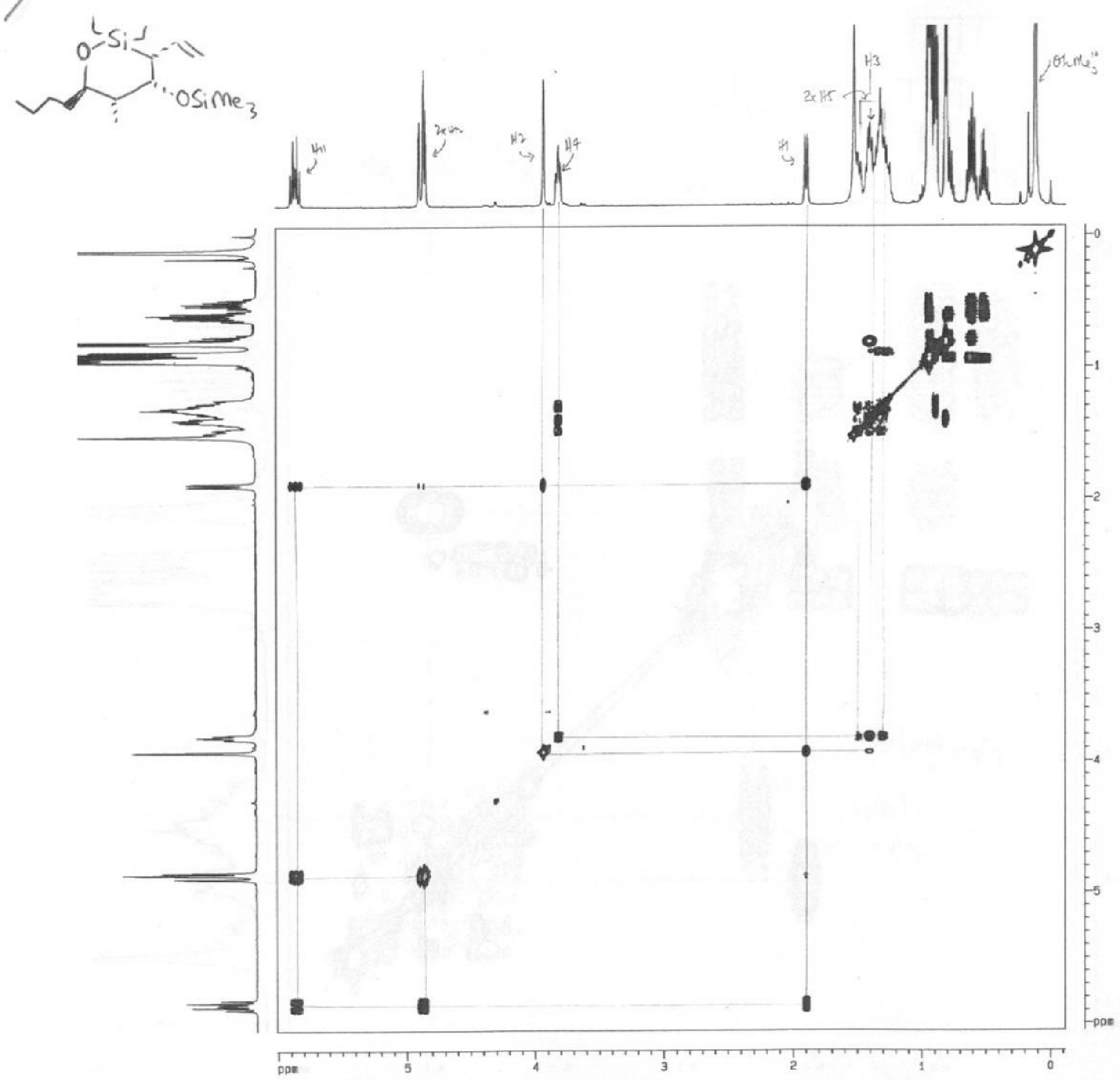

Current Data Parameters

NAME	ja09pj1d
EXPNO	4
PROCNO	1

F2 - Acquisition Parameters

Date_	20060109
Time	21.49
INSTRUM	drx500
PROBHD	5 mm TBI H/C
PULPROG	cosygp
TD	2048
SOLVENT	CDCl3
NS	8
DS	16
SWH	4310.345 Hz
FIDRES	2.104661 Hz
AQ	0.2376180 sec
RG	574.7
DW	116.000 usec
DE	5.50 usec
TE	300.0 K
d0	0.00000300 sec
D1	2.00000000 sec
d13	0.00000300 sec
D16	0.00010000 sec
IN0	0.00023200 sec

============ CHANNEL f1 ============

NUC1	1H
P0	10.70 usec
P1	10.70 usec
PL1	1.00 dB
SFO1	500.1318867 MHz

============ GRADIENT CHANNEL ========

GPNAM1	SINE.100
GPNAM2	SINE.100
GPX1	0.00 %
GPX2	0.00 %
GPY1	0.00 %
GPY2	0.00 %
GPZ1	10.00 %
GPZ2	10.00 %
P16	1000.00 usec

F1 - Acquisition parameters

ND0	1
TD	512
SFO1	500.1319 MHz
FIDRES	8.418642 Hz
SW	8.618 ppm

F2 - Processing parameters

SI	2048
SF	500.1300233 MHz
WDW	SINE
SSB	0
LB	0.00 Hz
GB	0
PC	1.00

F1 - Processing parameters

SI	1024
MC2	QF
SF	500.1300233 MHz
WDW	SINE
SSB	0
LB	0.00 Hz
GB	0

2D NMR plot parameters

CX2	20.00 cm
CX1	20.00 cm
F2PLO	6.011 ppm
F2LO	3006.29 Hz
F2PHI	-0.116 ppm
F2HI	-58.10 Hz
F1PLO	6.057 ppm
F1LO	3029.44 Hz
F1PHI	-0.070 ppm
F1HI	-34.94 Hz
F2PPMCM	0.30636 ppm/cm
F2HZCM	153.21930 Hz/cm
F1PPMCM	0.30636 ppm/cm
F1HZCM	153.21930 Hz/cm

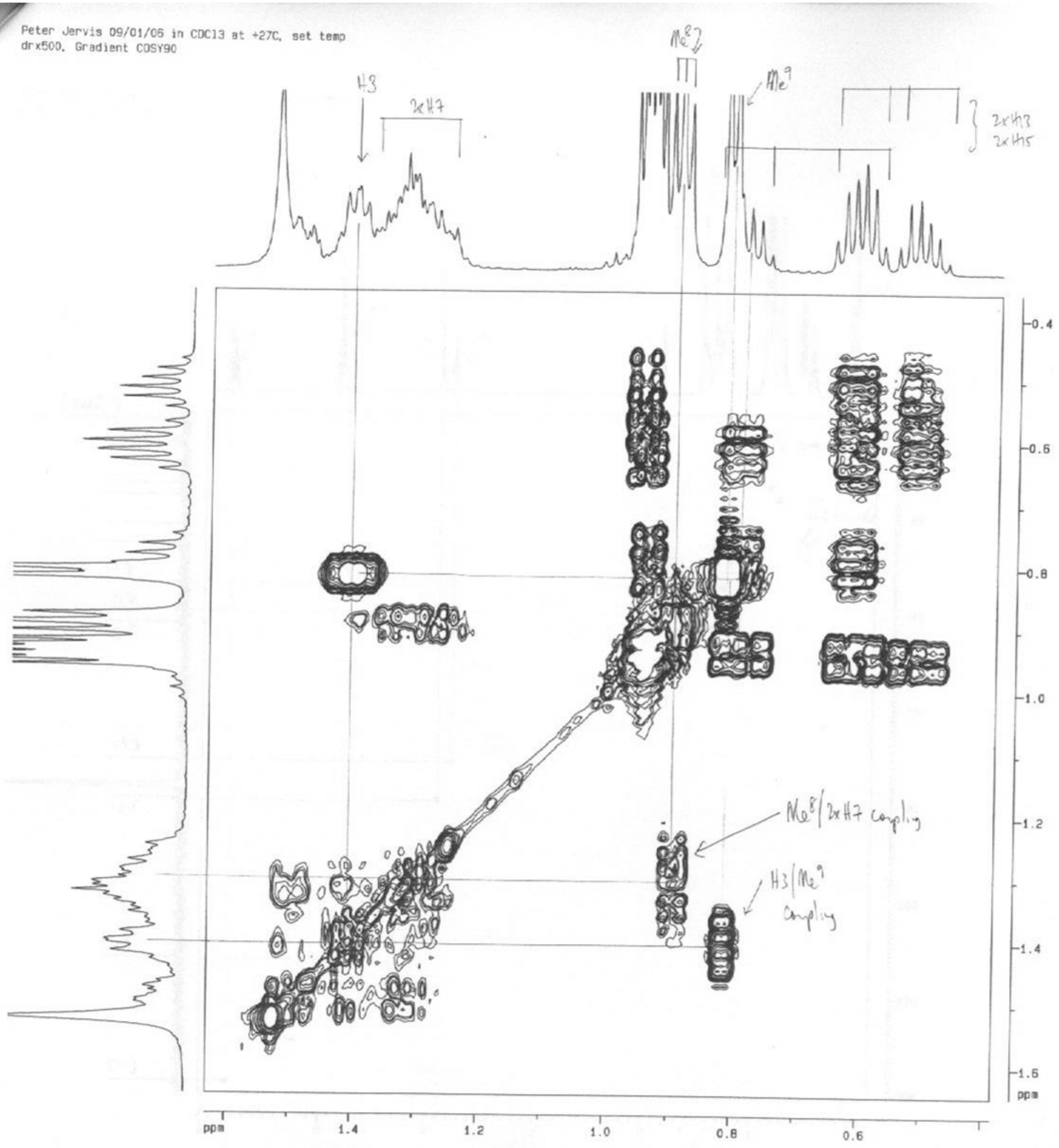
Peter Jervis 09/01/06 in CDCl3 at +27C, set temp
drx500. Gradient COSY90
H3
2xH7
Me9
2xH13
2xH15
Me8/2xH7 coupling
H3/Me9 coupling
ppm
1.4
1.2
1.0
0.8
0.6
0.4
1.6

Peter Jervis 09/01/06 in CDCl3 at +27C, set temp
drx500, Gradient HSGC

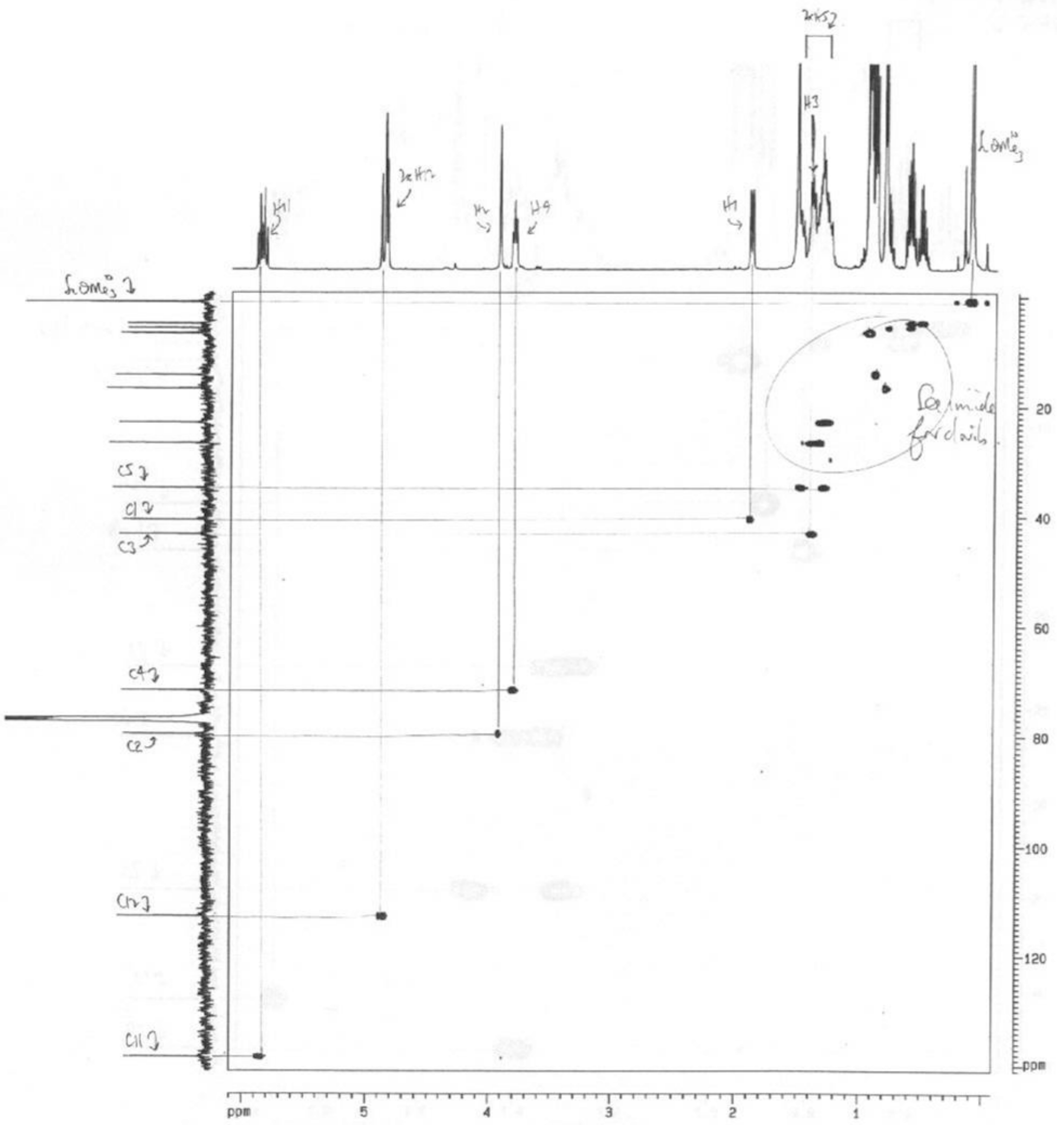

Current Data Parameters

NAME	ja09pj1d
EXPNO	2
PROCNO	1

F2 - Acquisition Parameters

Date_	20060109
Time	16.32
INSTRUM	drx500
PROBHD	5 mm TBI H/C
PULPROG	invigptp
TD	2048
SOLVENT	CDCl3
NS	8
DS	16
SWH	4310.345 Hz
FIDRES	2.104661 Hz
AQ	0.2376180 sec
RG	32768
DW	116.000 usec
DE	5.50 usec
TE	300.0 K
CNST2	145.0000000
d0	0.00000300 sec
D1	2.00000000 sec
d4	0.00172414 sec
d11	0.03000000 sec
d13	0.00000300 sec
D16	0.00010000 sec
d20	0.00110000 sec
d21	0.00061714 sec
IN0	0.00001140 sec

============ CHANNEL f1 ============

NUC1	1H
P1	10.70 usec
p2	21.40 usec
PL1	1.00 dB
SFO1	500.1318867 MHz

============ CHANNEL f2 ============

CPDPRG2	garp
NUC2	13C
P3	12.00 usec
p4	24.00 usec
PCPD2	76.00 usec
PL2	-1.00 dB
PL12	15.00 dB
SFO2	125.7667893 MHz

============ GRADIENT CHANNEL ========

GPNAM1	SINE.100
GPNAM2	SINE.100
GPNAM3	SINE.100
GPX1	0.00 %
GPX2	0.00 %
GPX3	0.00 %
GPY1	0.00 %
GPY2	0.00 %
GPY3	0.00 %
GPZ1	80.00 %
GPZ2	30.00 %
GPZ3	20.10 %
P16	1000.00 usec

F1 - Acquisition parameters

ND0	4
TD	512
SFO1	125.7668 MHz
FIDRES	42.831688 Hz
SW	174.369 ppm

F2 - Processing parameters

SI	2048
SF	500.1300233 MHz
WDW	QSINE
SSB	2
LB	0.00 Hz
GB	0
PC	1.00

F1 - Processing parameters

SI	1024
MC2	TPPI
SF	125.7577910 MHz
WDW	QSINE
SSB	2
LB	0.00 Hz
GB	0

2D NMR plot parameters

CX2	17.00 cm
CX1	17.00 cm
F2PLO	6.104 ppm
F2LO	3052.59 Hz
F2PHI	-0.082 ppm
F2HI	-41.26 Hz
F1PLO	141.033 ppm
F1LO	17736.00 Hz
F1PHI	-0.822 ppm
F1HI	-103.40 Hz
F2PPMCM	0.36389 ppm/cm
F2HZCM	181.99124 Hz/cm
F1PPMCM	8.34442 ppm/cm
F1HZCM	1049.37634 Hz/cm

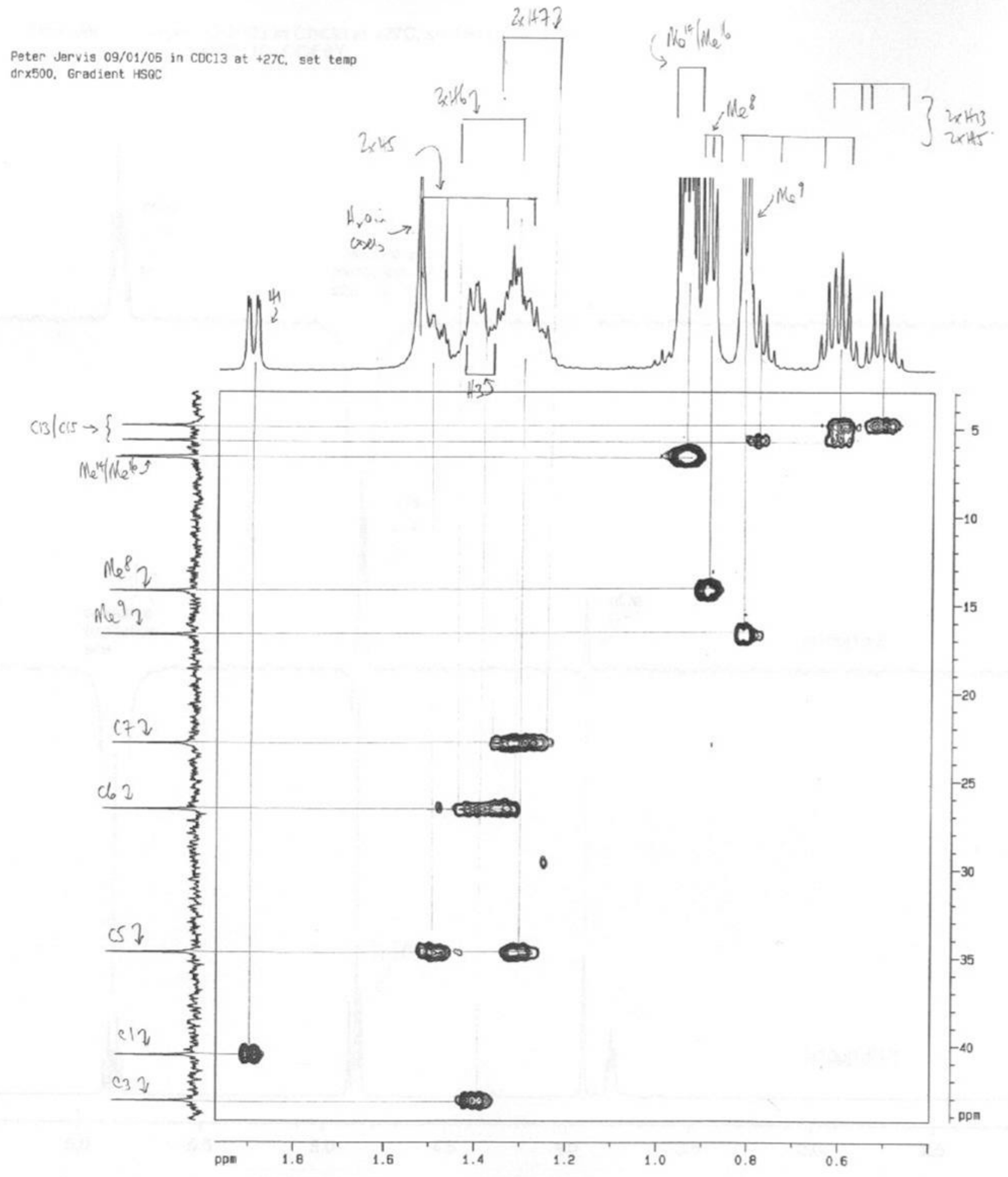
Peter Jervis 09/01/05 in CDCl3 at +27C, set temp
drx500, Gradient HSQC
ppm
1.8
1.6
1.4
1.2
1.0
0.8
0.6
5
10
15
20
25
30
35
40
ppm
C1
C3
C5
C6
C7
Me8
Me9
C13/C15
Me14/Me16
2xH5
2xH6
2xH7
H3
H1
Me8
Me9
Me14/Me16
2xH13
2xH15
H2O in CDCl3

15a

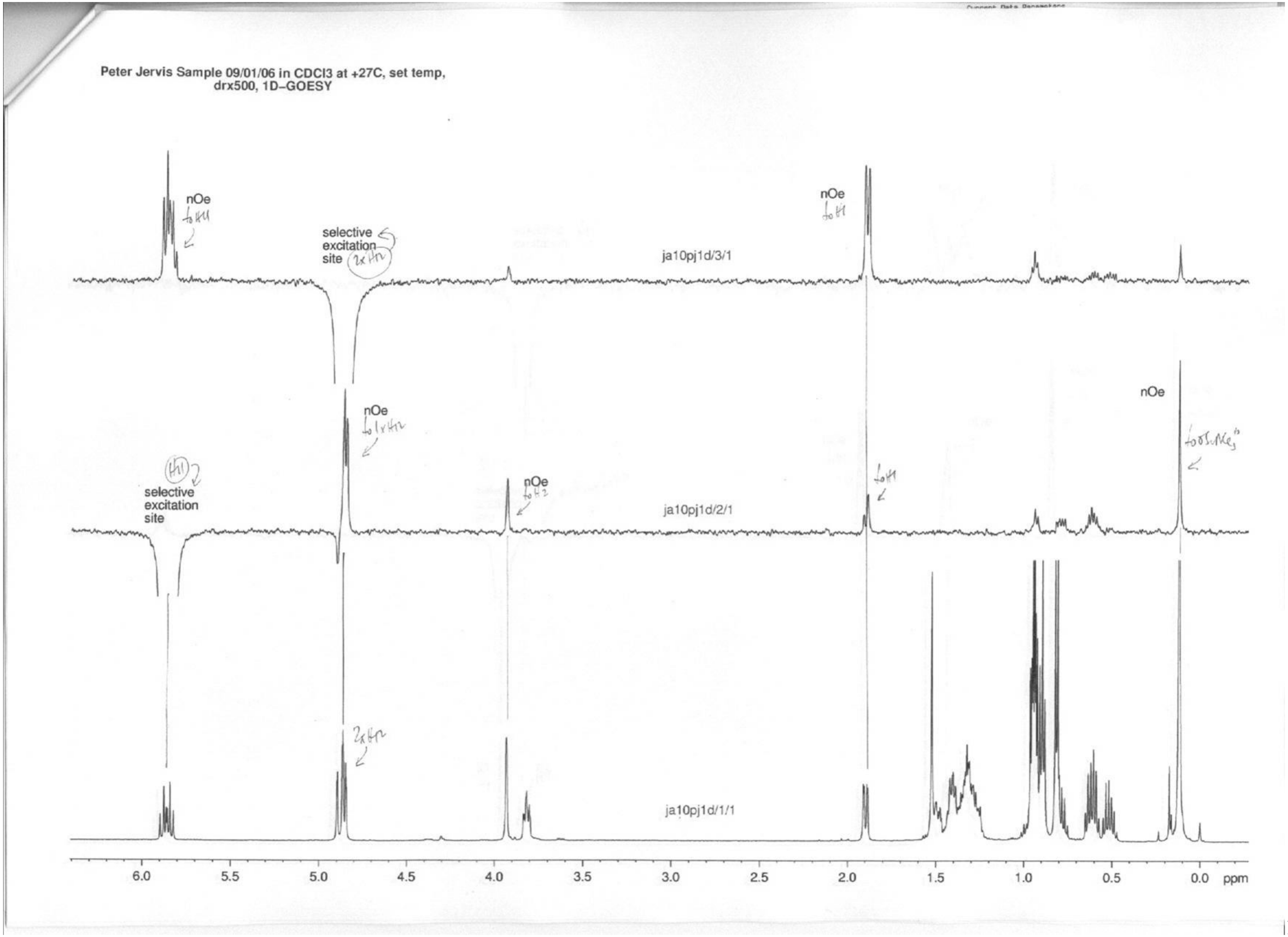
Peter Jervis Sample 09/01/06 in CDCl3 at +27C, set temp,
drx500, 1D-GOESY
nOe
selective
excitation
site
nOe
ja10pj1d/3/1
nOe
selective
excitation
site
nOe
nOe
ja10pj1d/2/1
ja10pj1d/1/1
6.0
5.5
5.0
4.5
4.0
3.5
3.0
2.5
2.0
1.5
1.0
0.5
0.0
ppm

15a

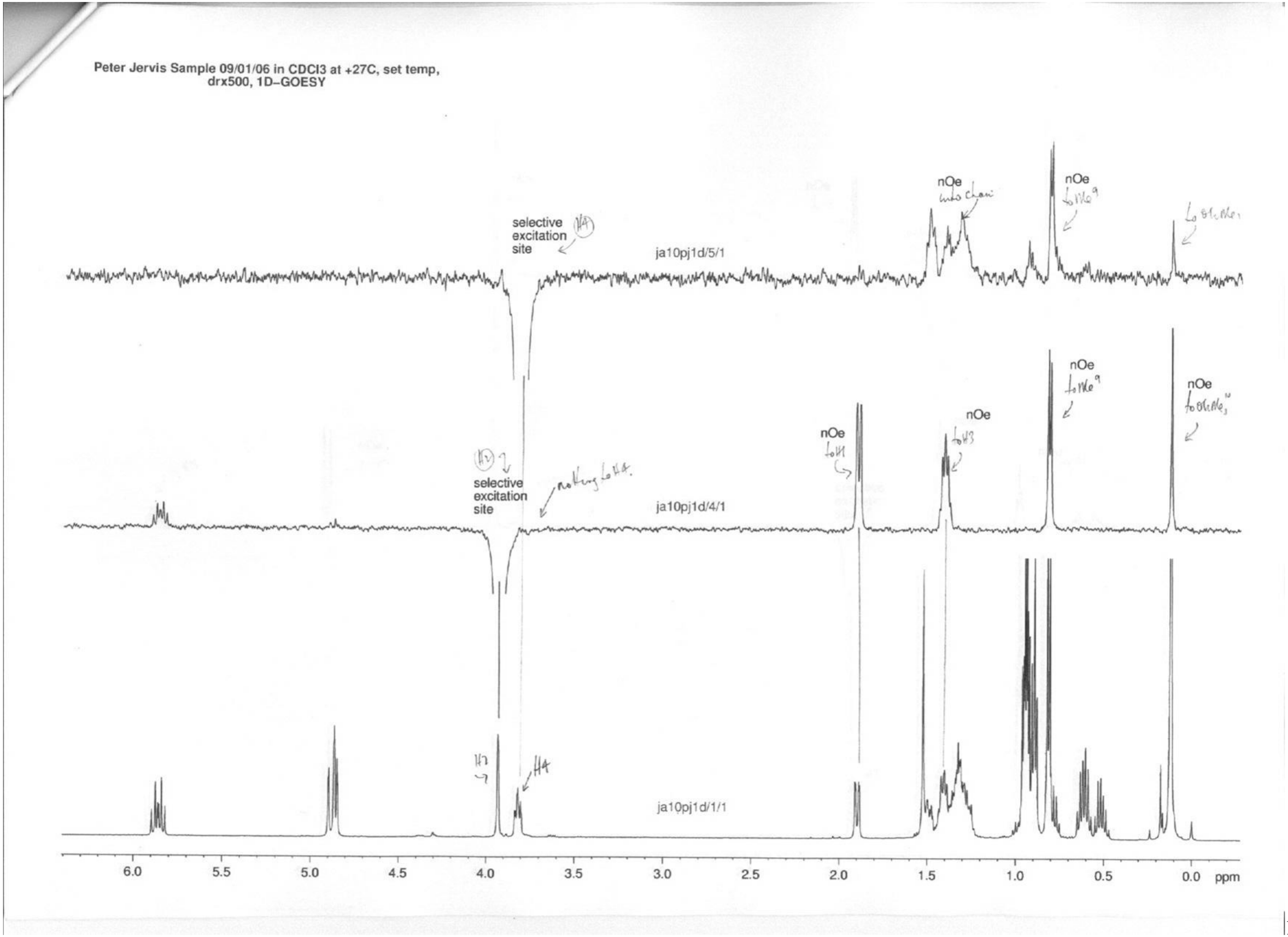
Peter Jervis Sample 09/01/06 in CDCl3 at +27C, set temp,
drx500, 1D–GOESY
selective excitation site
ja10pj1d/5/1
nOe
nOe
selective excitation site
ja10pj1d/4/1
nOe
nOe
nOe
nOe
ja10pj1d/1/1
6.0
5.5
5.0
4.5
4.0
3.5
3.0
2.5
2.0
1.5
1.0
0.5
0.0
ppm

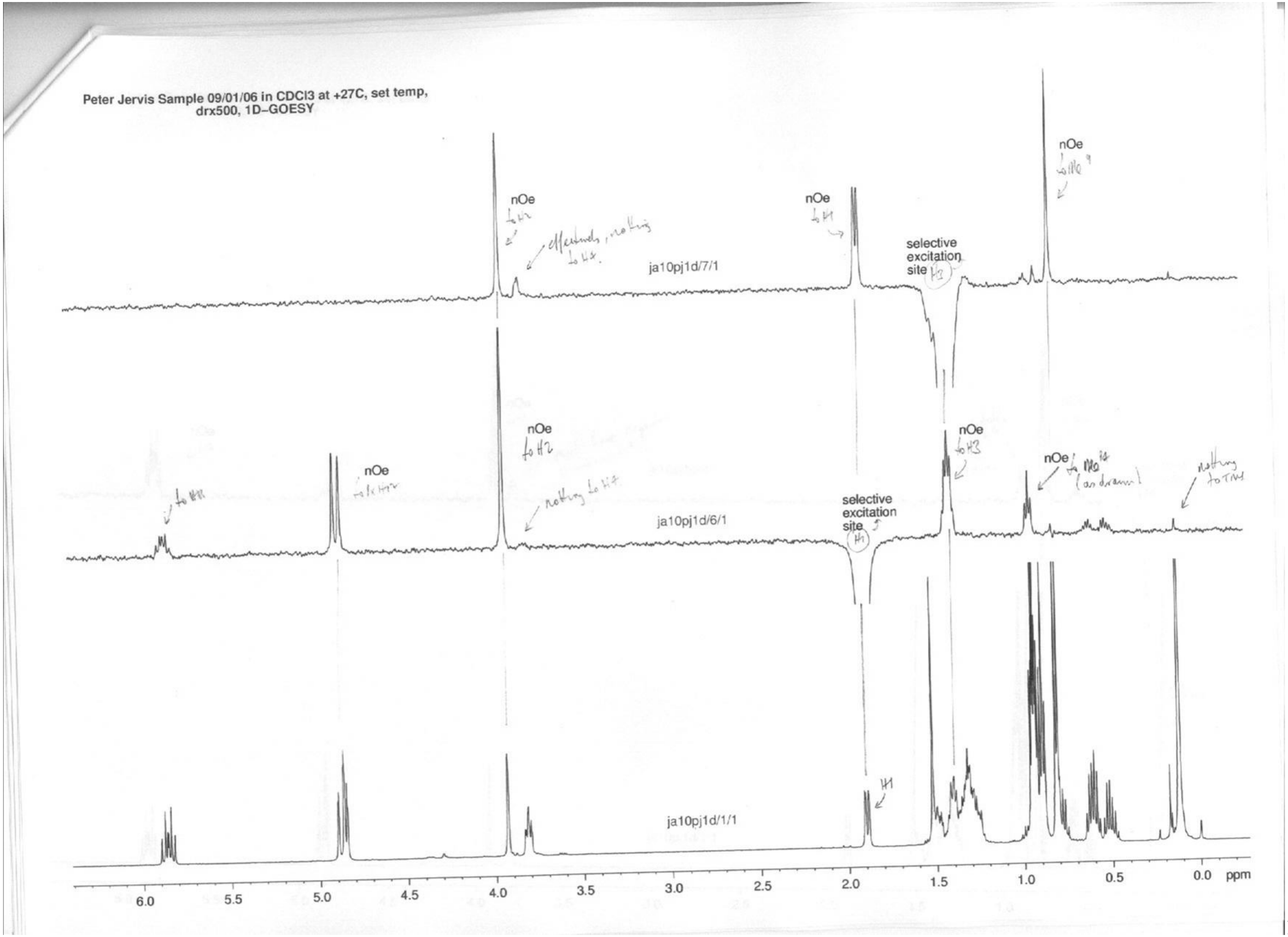
Peter Jervis Sample 09/01/06 in CDCl3 at +27C, set temp,
drx500, 1D-GOESY
nOe
nOe
nOe
selective
excitation
site
ja10pj1d/7/1
nOe
nOe
nOe
nOe
selective
excitation
site
ja10pj1d/6/1
ja10pj1d/1/1
6.0
5.5
5.0
4.5
4.0
3.5
3.0
2.5
2.0
1.5
1.0
0.5
0.0
ppm

15a

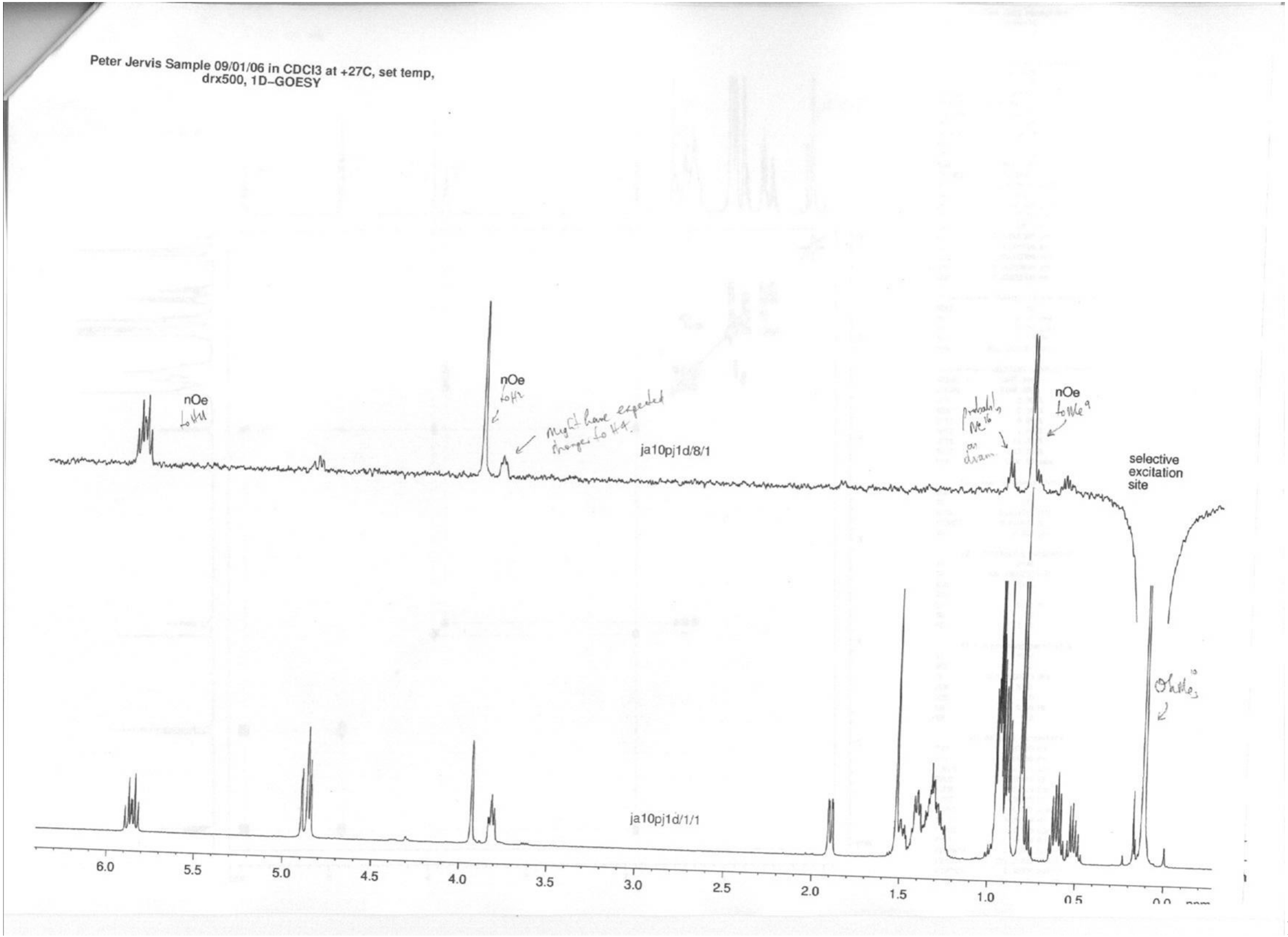
Peter Jervis Sample 09/01/06 in CDCl3 at +27C, set temp,
drx500, 1D–GOESY
nOe
nOe
nOe
ja10pj1d/8/1
selective
excitation
site
ja10pj1d/1/1
6.0
5.5
5.0
4.5
4.0
3.5
3.0
2.5
2.0
1.5
1.0
0.5

15a
