# Supplementary material for: Tether-directed synthesis of highly substituted oxasilacycles via an intramolecular allylation employing allylsilanes
Source: Beilstein J Org Chem. 2007 Feb 8;3:6. doi: 10.1186/1860-5397-3-6 (PMC1810303; doi:10.1186/1860-5397-3-6)
Supplement: File 6 — 1H-NMR and 13C-NMR Spectra for the following compounds: 16b, 17b. [file Beilstein_J_Org_Chem-03-06-s006.pdf]

Additional File 6

Tether-directed synthesis of highly substituted oxasilacycles *via* an intramolecular allylation employing allylsilanes

Peter J. Jervis and Liam R. Cox*

email: l.r.cox@bham.ac.uk

^{1}H-NMR and ^{13}C-NMR Spectra for the following compounds:

16b, 17b

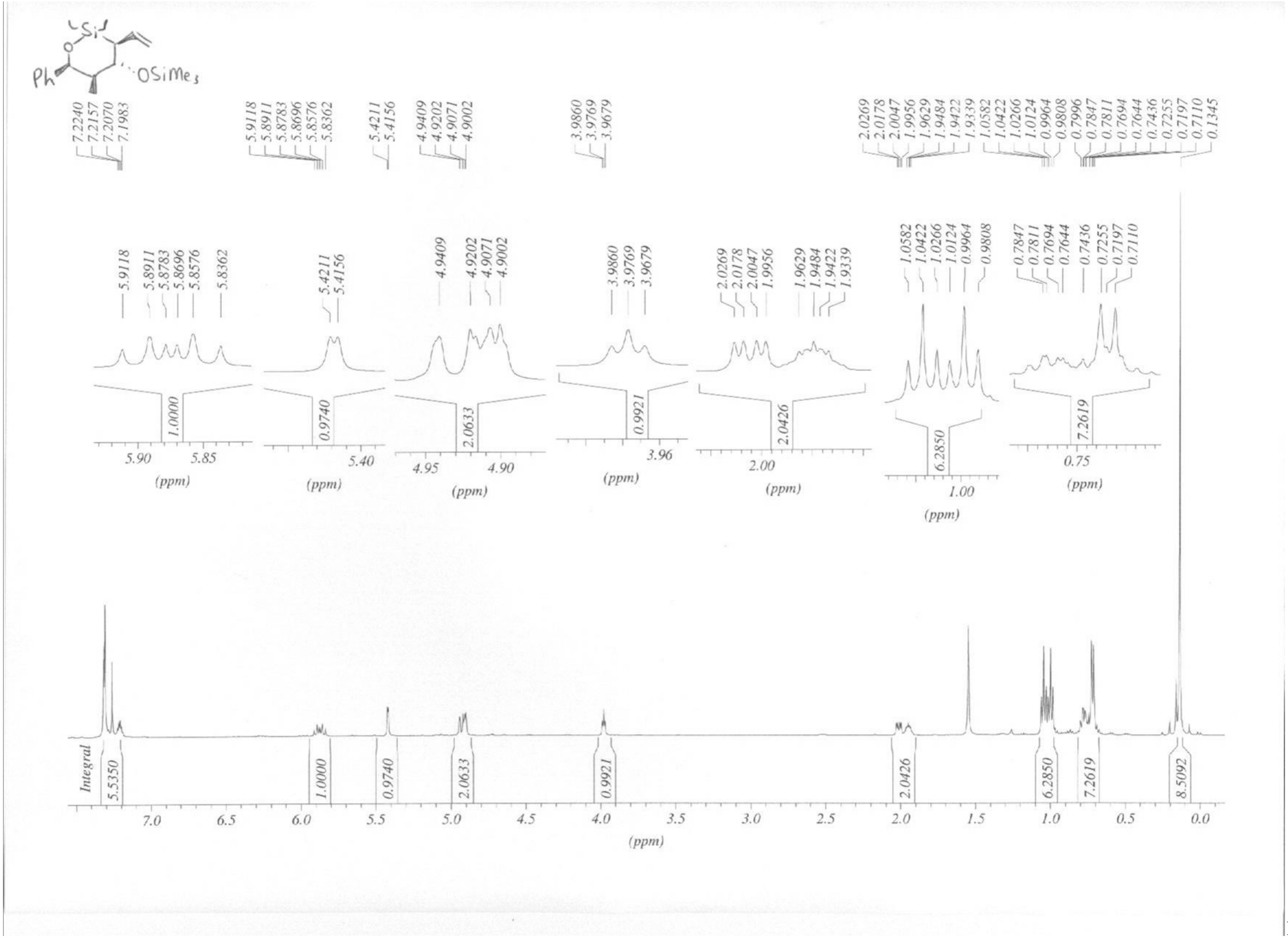

16b

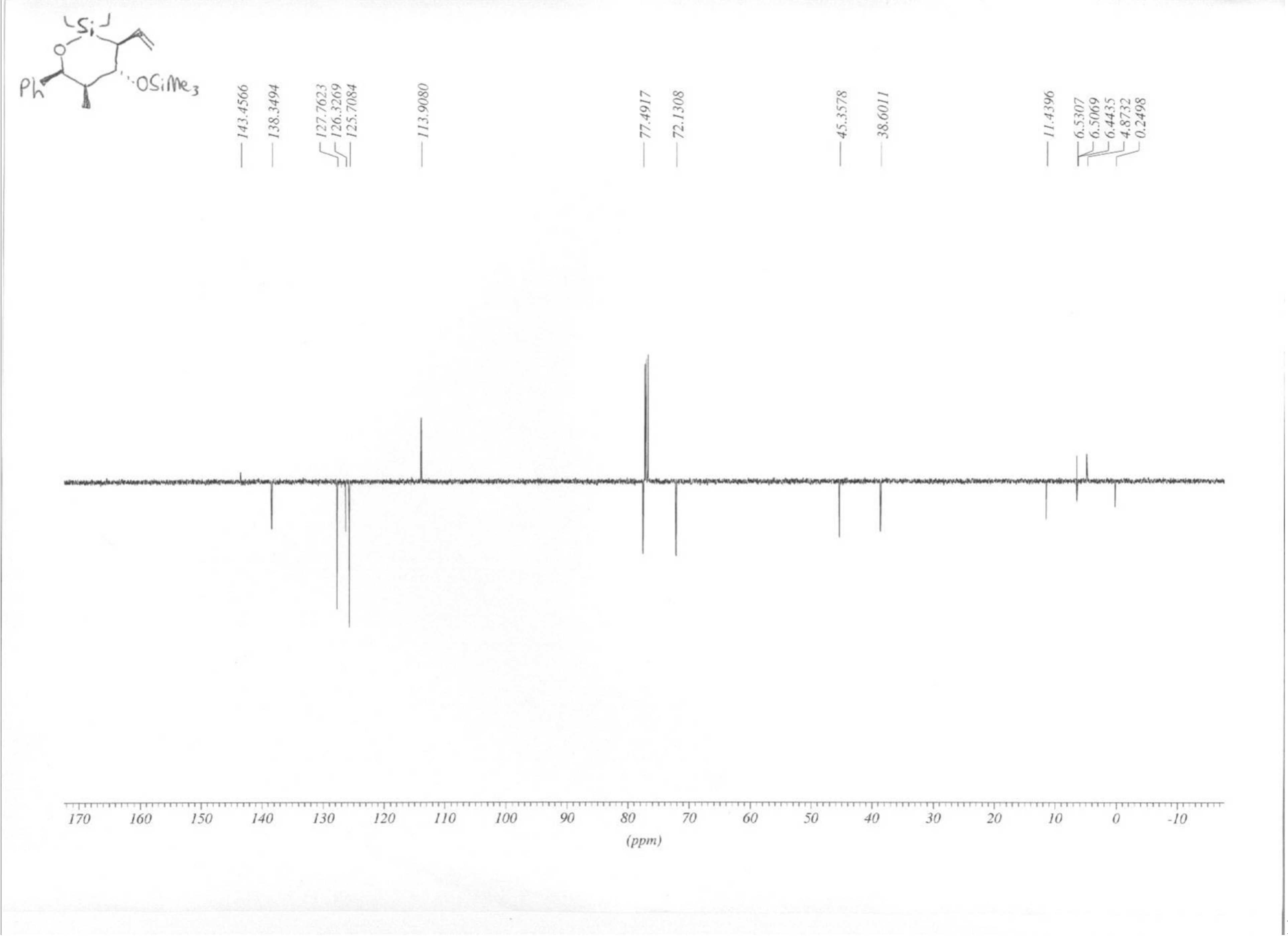
Si
O
Ph
OSiMe3
143.4566
138.3494
127.7623
126.3269
125.7084
113.9080
77.4917
72.1308
45.3578
38.6011
11.4396
6.5307
6.5069
6.4435
4.8732
0.2498
170 160 150 140 130 120 110 100 90 80 70 60 50 40 30 20 10 0 -10
(ppm)

16b

Peter Jervis Barcode 3902 in CDCl3 at +27C, set temp
drx500, Gradient COSY90

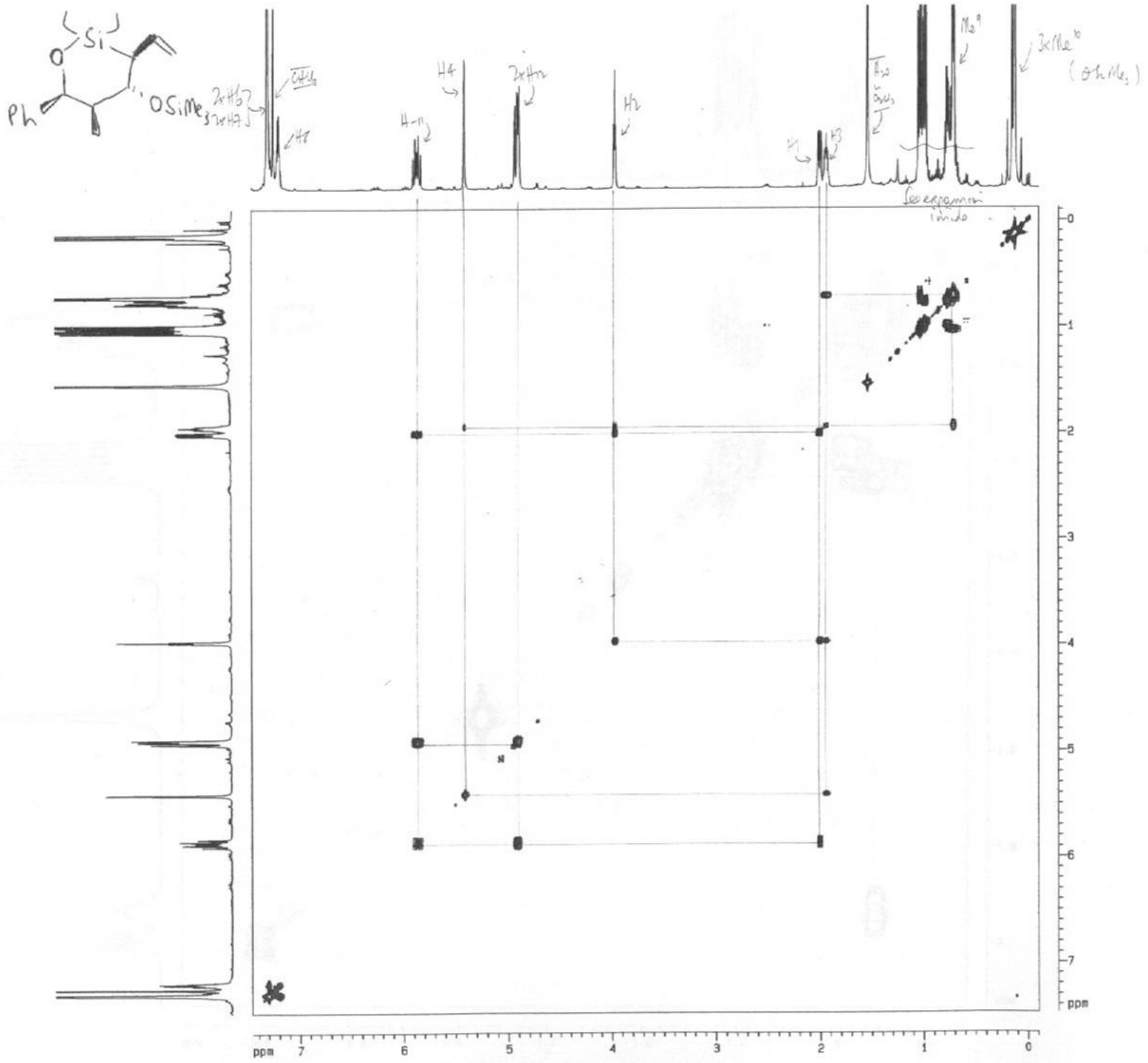

Current Data Parameters

NAME	j128pj1d
EXPNO	3
PROCNO	1

F2 - Acquisition Parameters

Date_	20050726
Time	19.26
INSTRUM	drx500
PROBHD	5 mm TBI H/C
PULPROG	cosygp
TD	2048
SOLVENT	CDCl3
NS	8
DS	16
SWH	4310.345 Hz
FIDRES	2.104661 Hz
AQ	0.2376180 sec
RG	362
DW	116.000 usec
DE	5.50 usec
TE	300.0 K
d0	0.00000300 sec
D1	2.00000000 sec
d13	0.00000300 sec
D16	0.00010000 sec
IN0	0.00023200 sec

============ CHANNEL f1 =============

NUC1	1H
P0	10.60 usec
P1	10.60 usec
PL1	1.00 dB
SFO1	500.1318867 MHz

============ GRADIENT CHANNEL ========

GPNAM1	SINE.100
GPNAM2	SINE.100
GPX1	0.00 %
GPX2	0.00 %
GPY1	0.00 %
GPY2	0.00 %
GPZ1	10.00 %
GPZ2	10.00 %
P16	1000.00 usec

F1 - Acquisition parameters

ND0	1
TD	512
SFO1	500.1319 MHz
FIDRES	8.418642 Hz
SW	8.618 ppm

F2 - Processing parameters

SI	2048
SF	500.1300233 MHz
WDW	SINE
SSB	0
LB	0.00 Hz
GB	0
PC	1.00

F1 - Processing parameters

SI	1024
MC2	QF
SF	500.1300233 MHz
WDW	SINE
SSB	0
LB	0.00 Hz
GB	0

2D NMR plot parameters

CX2	20.00 cm
CX1	20.00 cm
F2PLO	7.463 ppm
F2LO	3732.40 Hz
F2PHI	-0.104 ppm
F2HI	-51.78 Hz
F1PLO	7.480 ppm
F1LO	3740.82 Hz
F1PHI	-0.120 ppm
F1HI	-60.20 Hz
F2PPMCM	0.37832 ppm/cm
F2HZCM	189.20898 Hz/cm
F1PPMCM	0.38000 ppm/cm
F1HZCM	190.05087 Hz/cm

16b

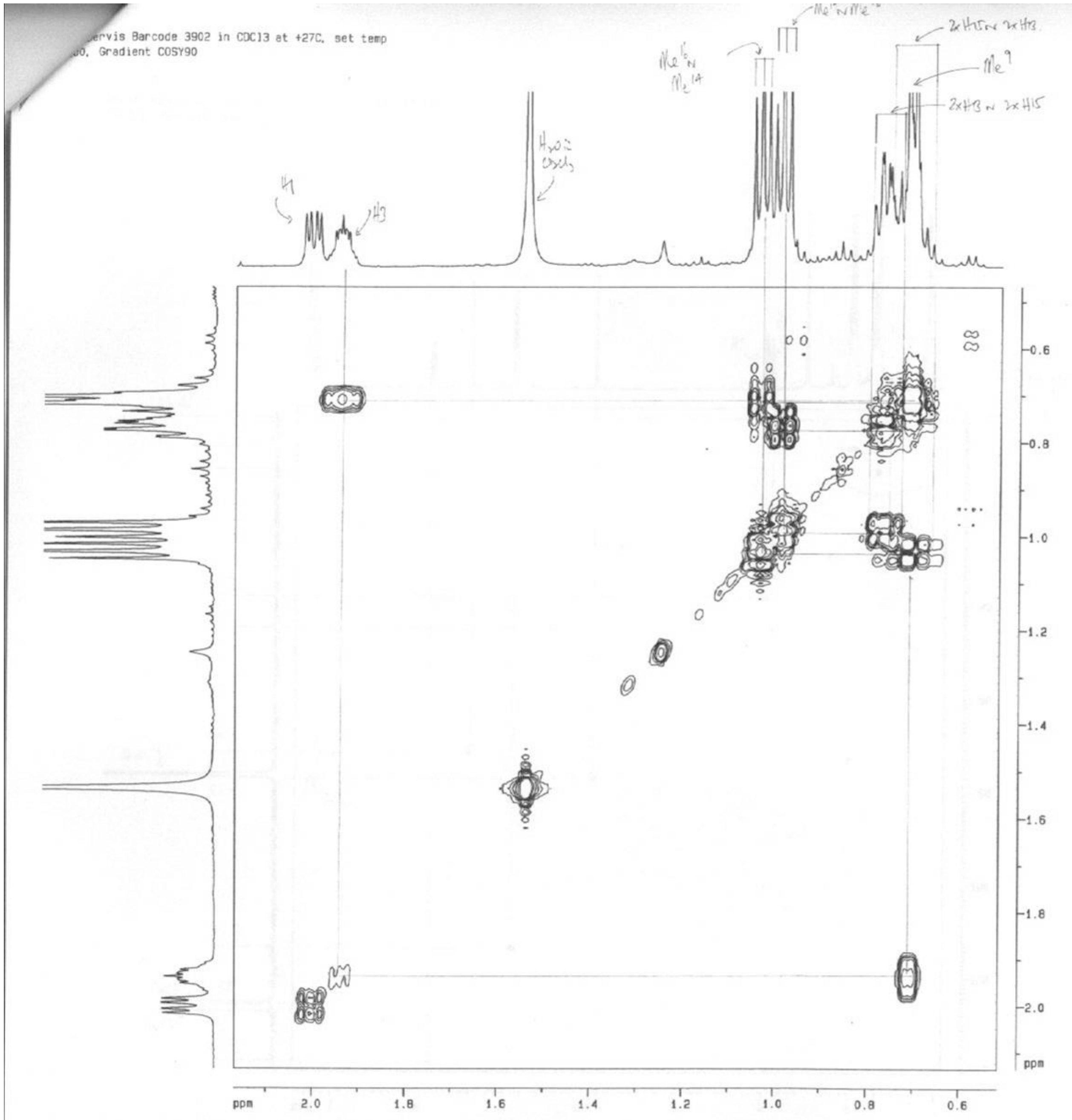
ervis Barcode 3902 in CDCl3 at +27C, set temp
00, Gradient COSY90
H1
H3
Me9
2xH15 or 2xH13
2xH13 or 2xH15
ppm
2.0
1.8
1.6
1.4
1.2
1.0
0.8
0.6

Peter Jervis Barcode 3902 in CDCl3 at +27C, set temp
drx500, Gradient HSGC

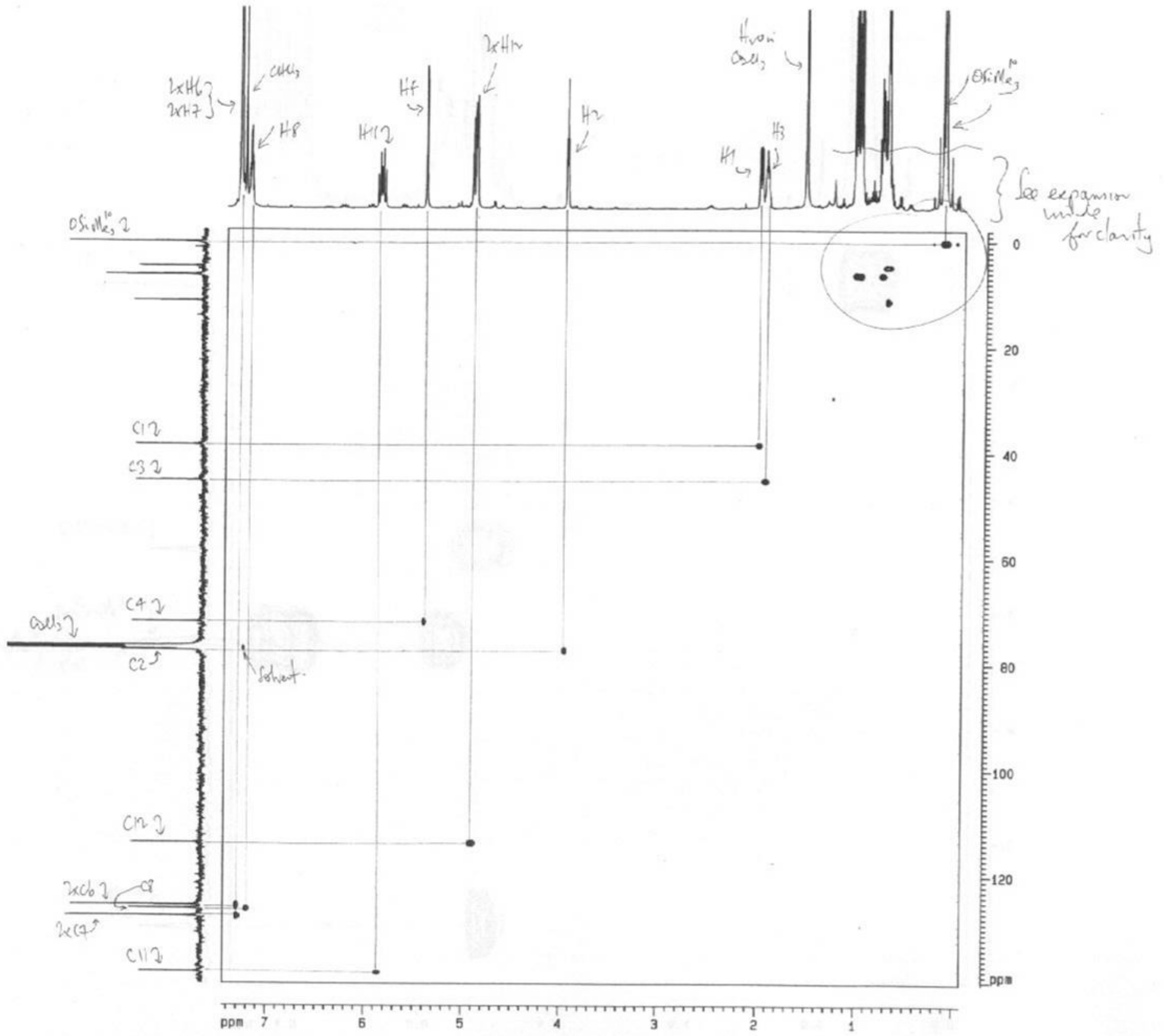

Current Data Parameters

NAME	j128pj1d
EXPNO	2
PROCNO	1

F2 - Acquisition Parameters

Date_	20060726
Time	16.51
INSTRUM	drx500
PROBHD	5 mm TBI H/C
PULPROG	invigptp
TD	2048
SOLVENT	CDCl3
NS	8
DS	16
SWH	4310.345 Hz
FIDRES	2.104661 Hz
AQ	0.2376180 sec
RG	32768
DW	116.000 usec
DE	5.50 usec
TE	300.0 K
CNST2	145.0000000
d0	0.00000300 sec
D1	2.00000000 sec
d4	0.00172414 sec
d11	0.03000000 sec
d13	0.00000300 sec
D16	0.00010000 sec
d20	0.00110000 sec
d21	0.00081714 sec
IN0	0.00001140 sec

============ CHANNEL f1 ============

NUC1	1H
P1	10.60 usec
p2	21.20 usec
PL1	1.00 dB
SFO1	500.1318867 MHz

============ CHANNEL f2 ============

CPDPRG2	garp
NUC2	13C
P3	12.00 usec
p4	24.00 usec
PCPD2	76.00 usec
PL2	-1.00 dB
PL12	15.00 dB
SFO2	125.7667893 MHz

============ GRADIENT CHANNEL ======

GPNAM1	SINE.100
GPNAM2	SINE.100
GPNAM3	SINE.100
GPX1	0.00 %
GPX2	0.00 %
GPX3	0.00 %
GPY1	0.00 %
GPY2	0.00 %
GPY3	0.00 %
GPZ1	80.00 %
GPZ2	30.00 %
GPZ3	20.10 %
P16	1000.00 usec

F1 - Acquisition parameters

ND0	4
TD	512
SFO1	125.7668 MHz
FIDRES	42.831688 Hz
SW	174.369 ppm

F2 - Processing parameters

SI	2048
SF	500.1300233 MHz
WDW	QSINE
SSB	2
LB	0.00 Hz
GB	0
PC	1.00

F1 - Processing parameters

SI	1024
MC2	TPPI
SF	125.7577945 MHz
WDW	QSINE
SSB	2
LB	0.00 Hz
GB	0

2D NMR plot parameters

CX2	17.00 cm
CX1	17.00 cm
F2PLO	7.442 ppm
F2LO	3721.88 Hz
F2PHI	-0.082 ppm
F2HI	-41.26 Hz
F1PLO	140.494 ppm
F1LO	17668.25 Hz
F1PHI	-2.042 ppm
F1HI	-256.81 Hz
F2PPMCM	0.44261 ppm/cm
F2HZCM	221.36076 Hz/cm
F1PPMCM	8.38449 ppm/cm
F1HZCM	1054.41553 Hz/cm

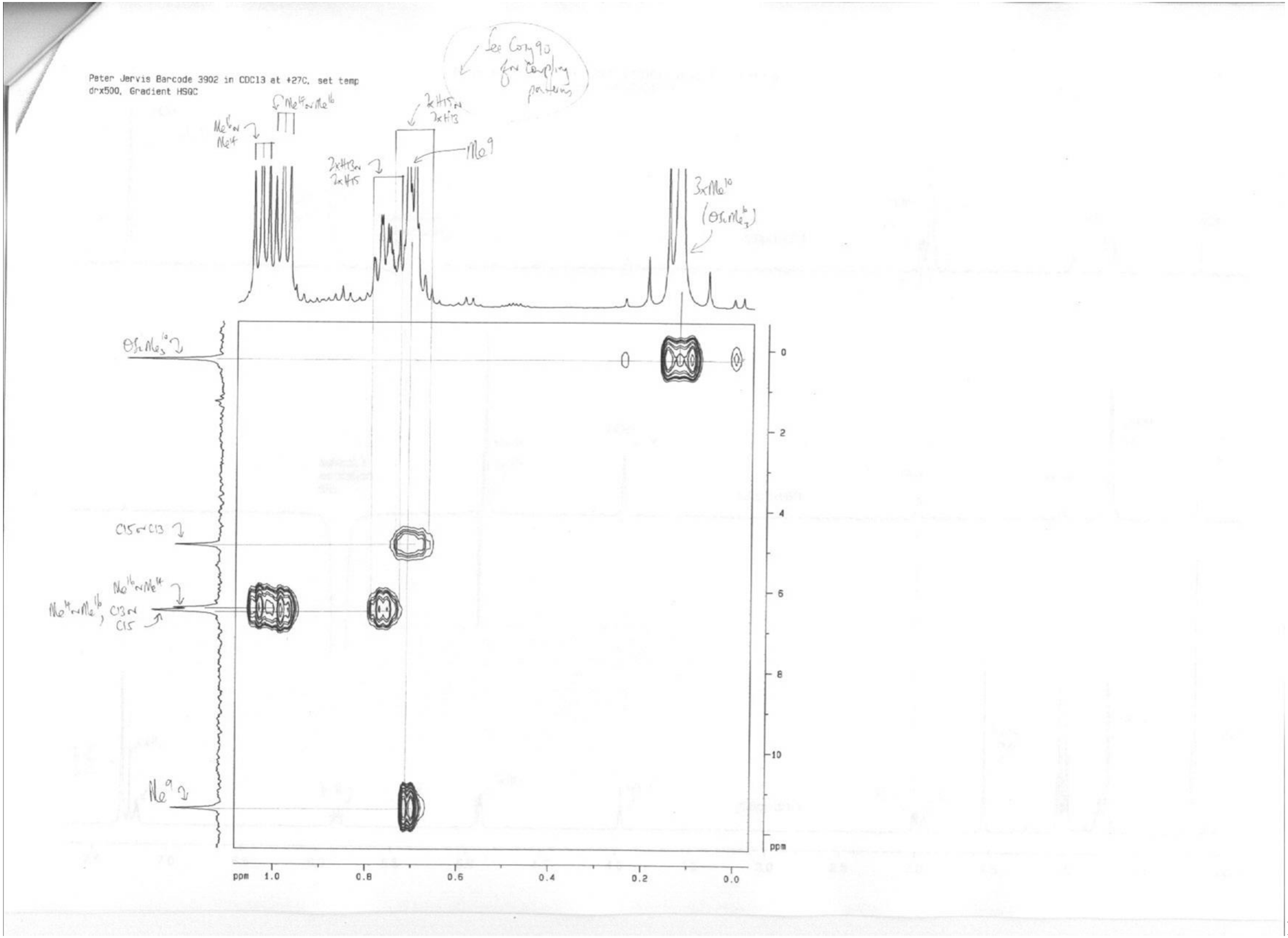
Peter Jervis Barcode 3902 in CDCl3 at +27C, set temp
drx500, Gradient HSQC
See Cosy 90 for coupling patterns
Me9
3xMe10
ppm
1.0
0.8
0.6
0.4
0.2
0.0
0
2
4
6
8
10
ppm

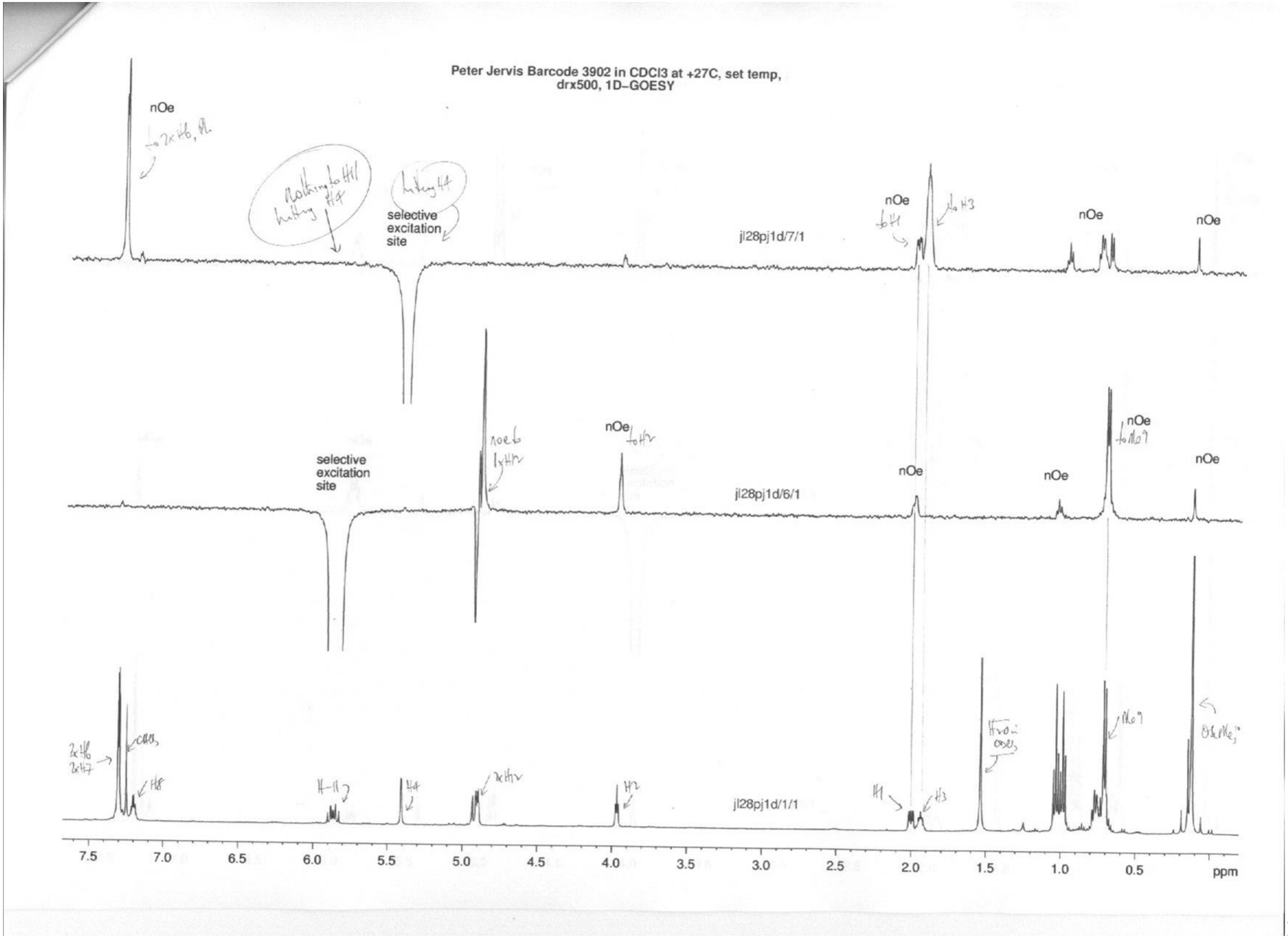
Peter Jervis Barcode 3902 in CDCl3 at +27C, set temp,
drx500, 1D-GOESY
nOe
selective
excitation
site
jl28pj1d/7/1
jl28pj1d/6/1
jl28pj1d/1/1
7.5
7.0
6.5
6.0
5.5
5.0
4.5
4.0
3.5
3.0
2.5
2.0
1.5
1.0
0.5
ppm

16b

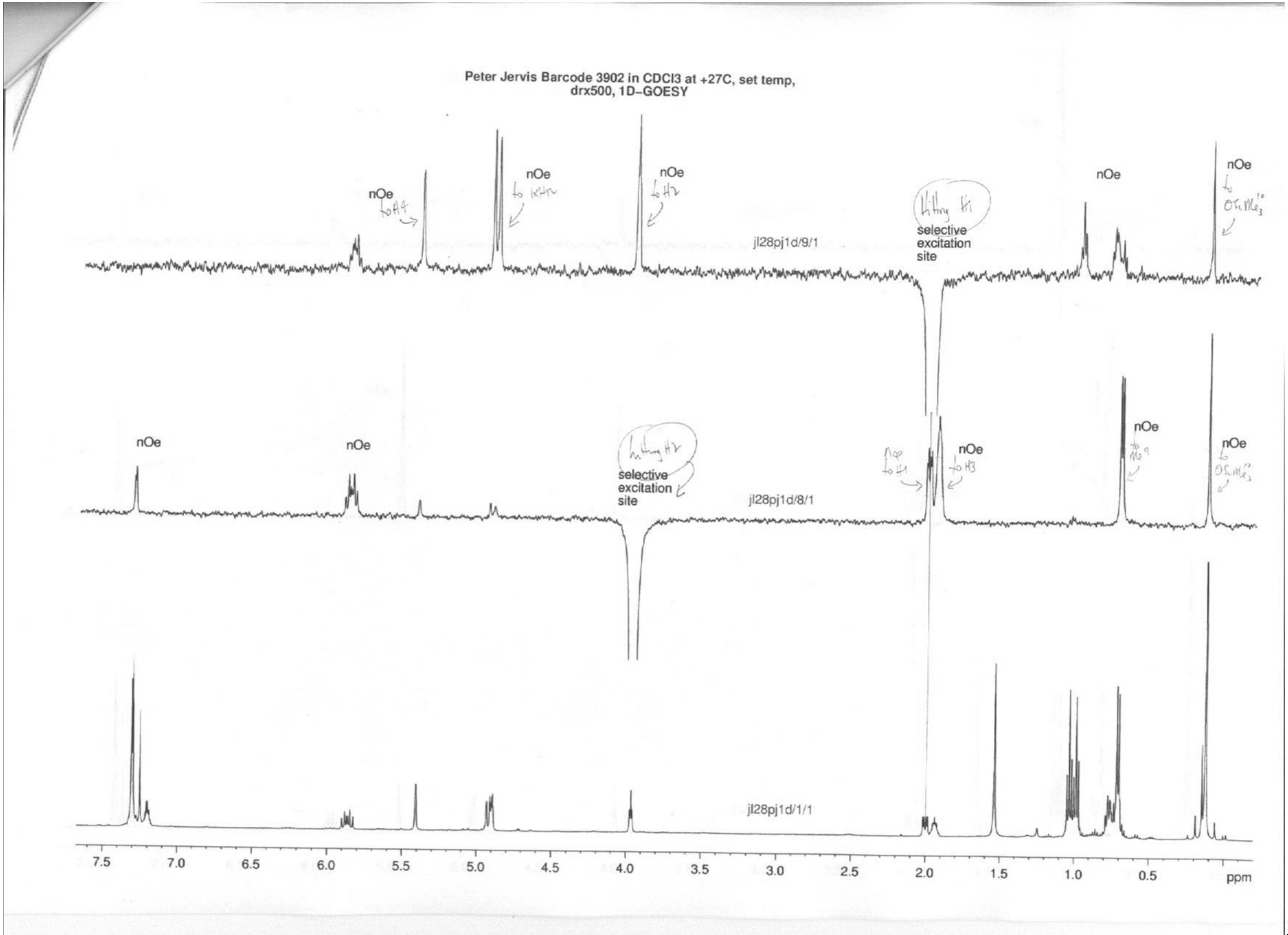
Peter Jervis Barcode 3902 in CDCl3 at +27C, set temp,
drx500, 1D-GOESY
nOe
nOe
nOe
nOe
nOe
selective
excitation
site
jl28pj1d/9/1
nOe
nOe
nOe
nOe
nOe
selective
excitation
site
jl28pj1d/8/1
jl28pj1d/1/1
7.5
7.0
6.5
6.0
5.5
5.0
4.5
4.0
3.5
3.0
2.5
2.0
1.5
1.0
0.5
ppm

16b

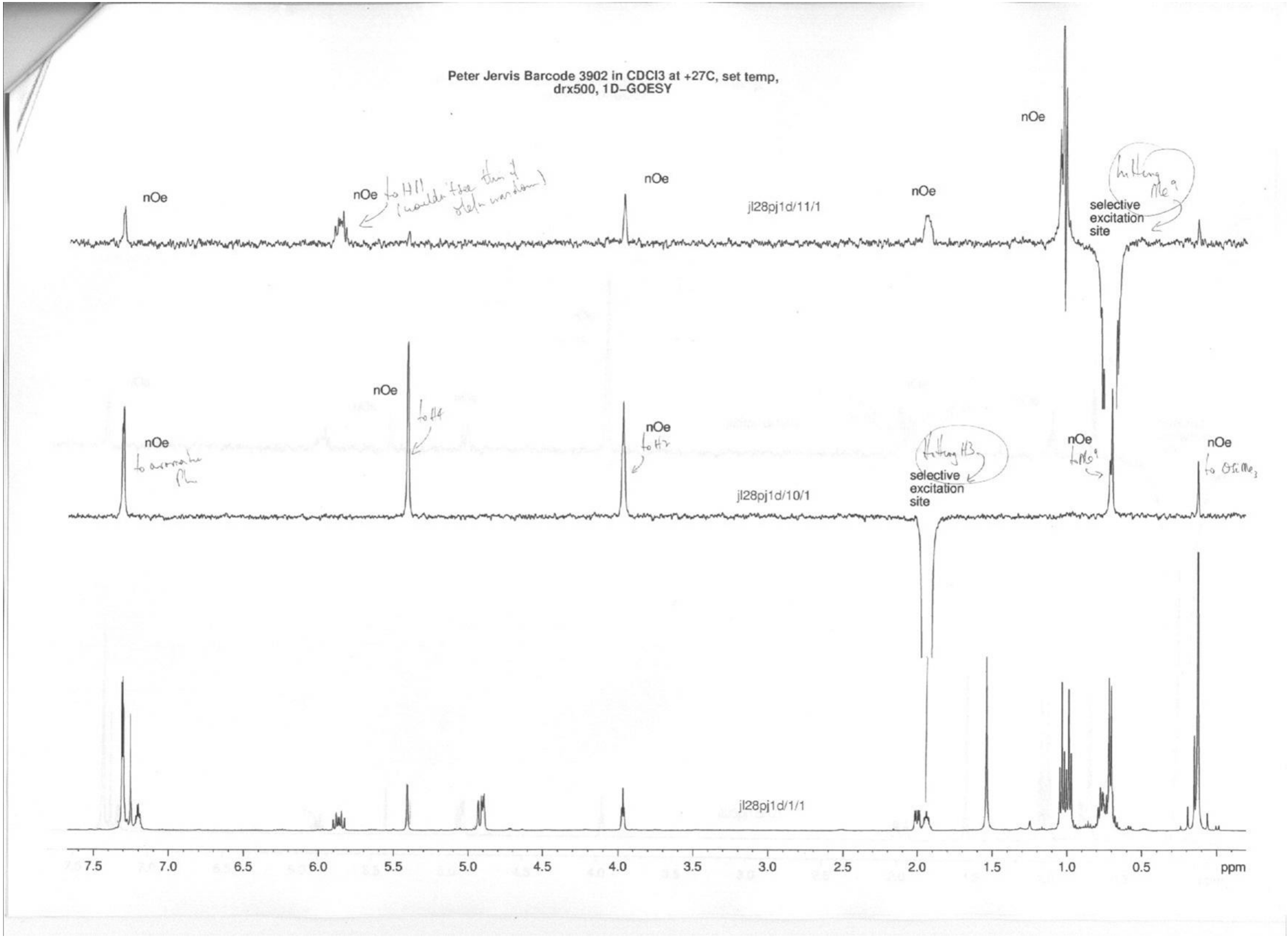
Peter Jervis Barcode 3902 in CDCl3 at +27C, set temp,
drx500, 1D-GOESY
nOe
nOe
to H11
nOe
nOe
nOe
jl28pj1d/11/1
selective
excitation
site
nOe
to H4
nOe
to H2
nOe
selective
excitation
site
nOe
nOe
jl28pj1d/10/1
jl28pj1d/1/1
7.5
7.0
6.5
6.0
5.5
5.0
4.5
4.0
3.5
3.0
2.5
2.0
1.5
1.0
0.5
ppm

16b

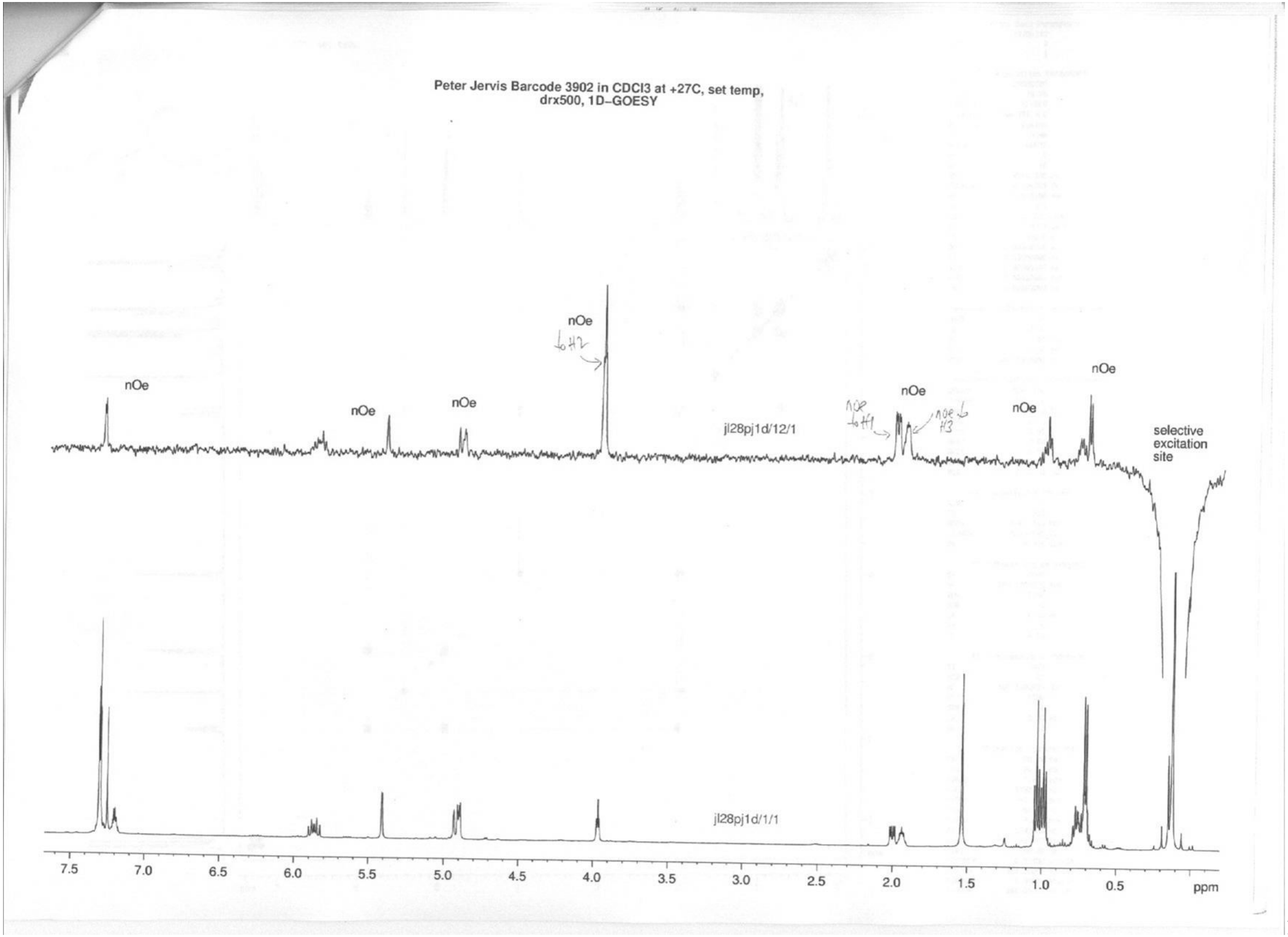
Peter Jervis Barcode 3902 in CDCl3 at +27C, set temp, drx500, 1D-GOESY
nOe
to H2
nOe
nOe
nOe
nOe
nOe
nOe
nOe to H1
nOe to H3
jl28pj1d/12/1
selective excitation site
jl28pj1d/1/1
7.5
7.0
6.5
6.0
5.5
5.0
4.5
4.0
3.5
3.0
2.5
2.0
1.5
1.0
0.5
ppm

16b

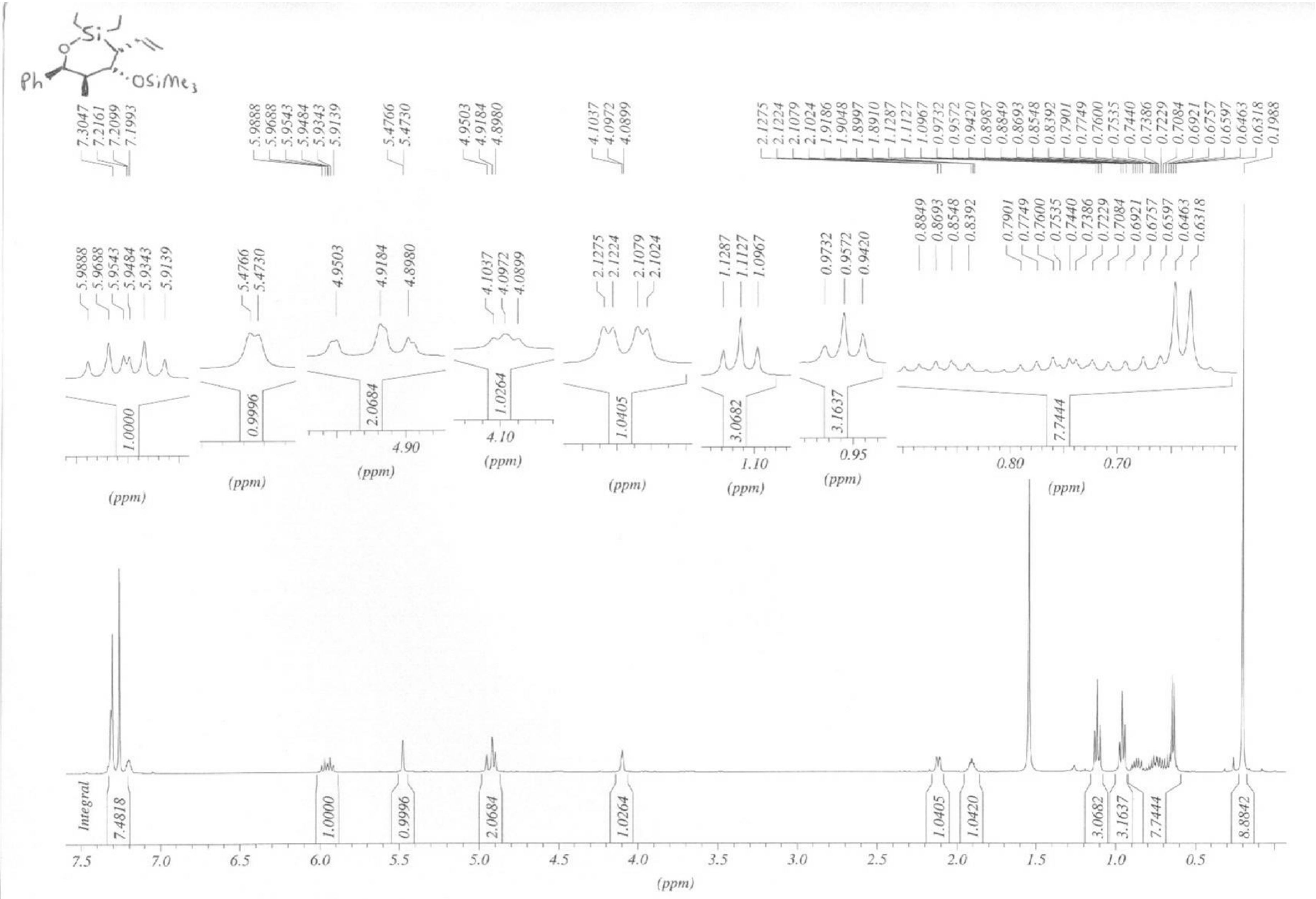
OSiMe3
Ph
Si
7.3047
7.2161
7.2099
7.1993
5.9888
5.9688
5.9543
5.9484
5.9343
5.9139
5.4766
5.4730
4.9503
4.9184
4.8980
4.1037
4.0972
4.0899
2.1275
2.1224
2.1079
2.1024
1.9186
1.9048
1.8997
1.8910
1.1287
1.1127
1.0967
0.9732
0.9572
0.9420
0.8987
0.8849
0.8693
0.8548
0.8392
0.7901
0.7749
0.7600
0.7535
0.7440
0.7386
0.7229
0.7084
0.6921
0.6757
0.6597
0.6463
0.6318
0.1988
1.0000
0.9996
2.0684
4.90
1.0264
4.10
1.0405
3.0682
1.10
3.1637
0.95
7.7444
0.80
0.70
(ppm)
Integral
7.4818
1.0000
0.9996
2.0684
1.0264
1.0405
1.0420
3.0682
3.1637
7.7444
8.8842
7.5
7.0
6.5
6.0
5.5
5.0
4.5
4.0
3.5
3.0
2.5
2.0
1.5
1.0
0.5
(ppm)

17b

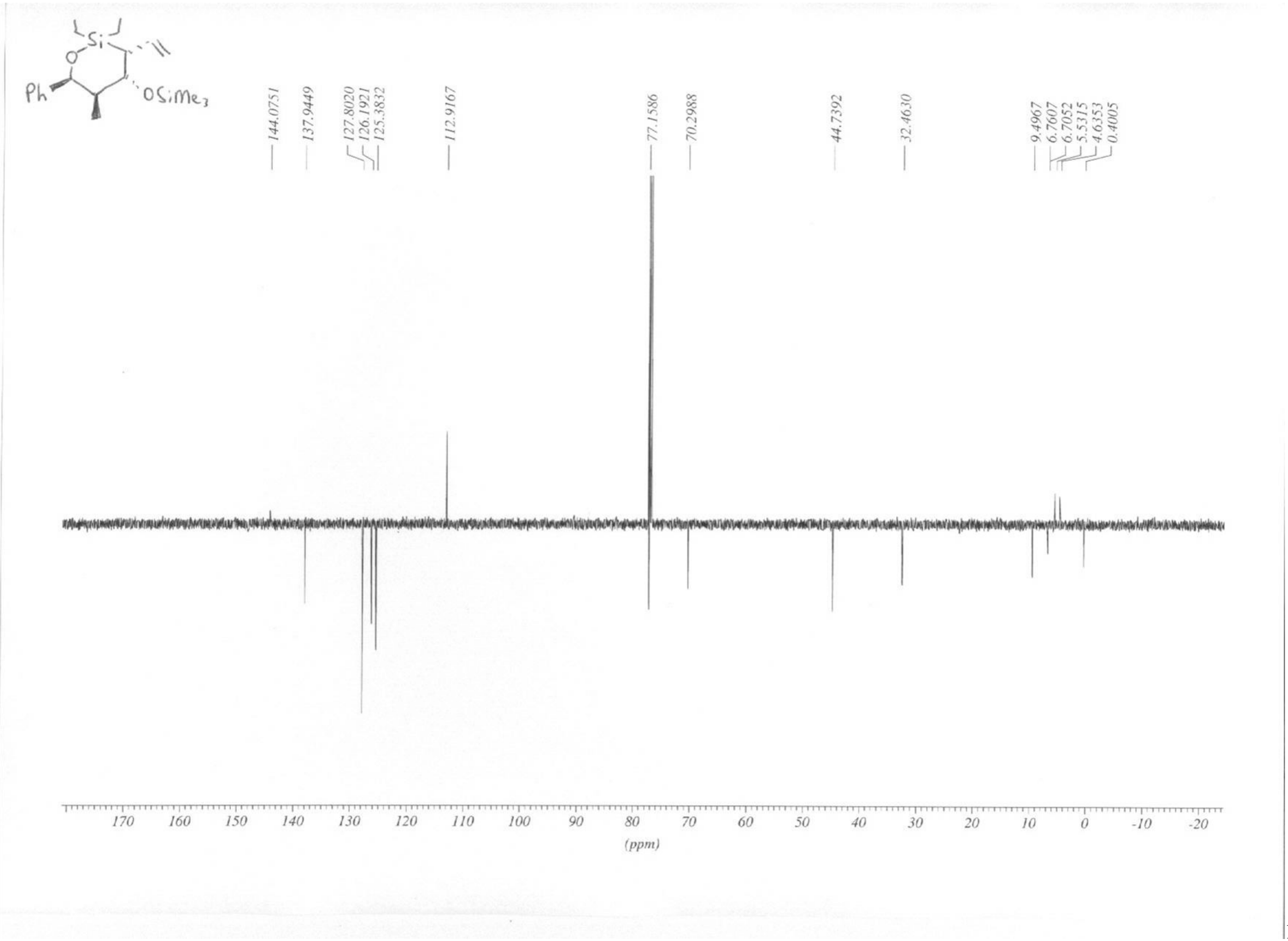
Ph
OSiMe3
144.0751
137.9449
127.8020
126.1921
125.3832
112.9167
77.1586
70.2988
44.7392
32.4630
9.4967
6.7607
6.7052
5.5315
4.6353
0.4005
170 160 150 140 130 120 110 100 90 80 70 60 50 40 30 20 10 0 -10 -20
(ppm)

17b

Peter Jervis Sample 09/09/05 in CDC13 at +27C, set temp
drx500, Gradient COSY90

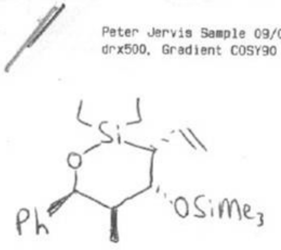

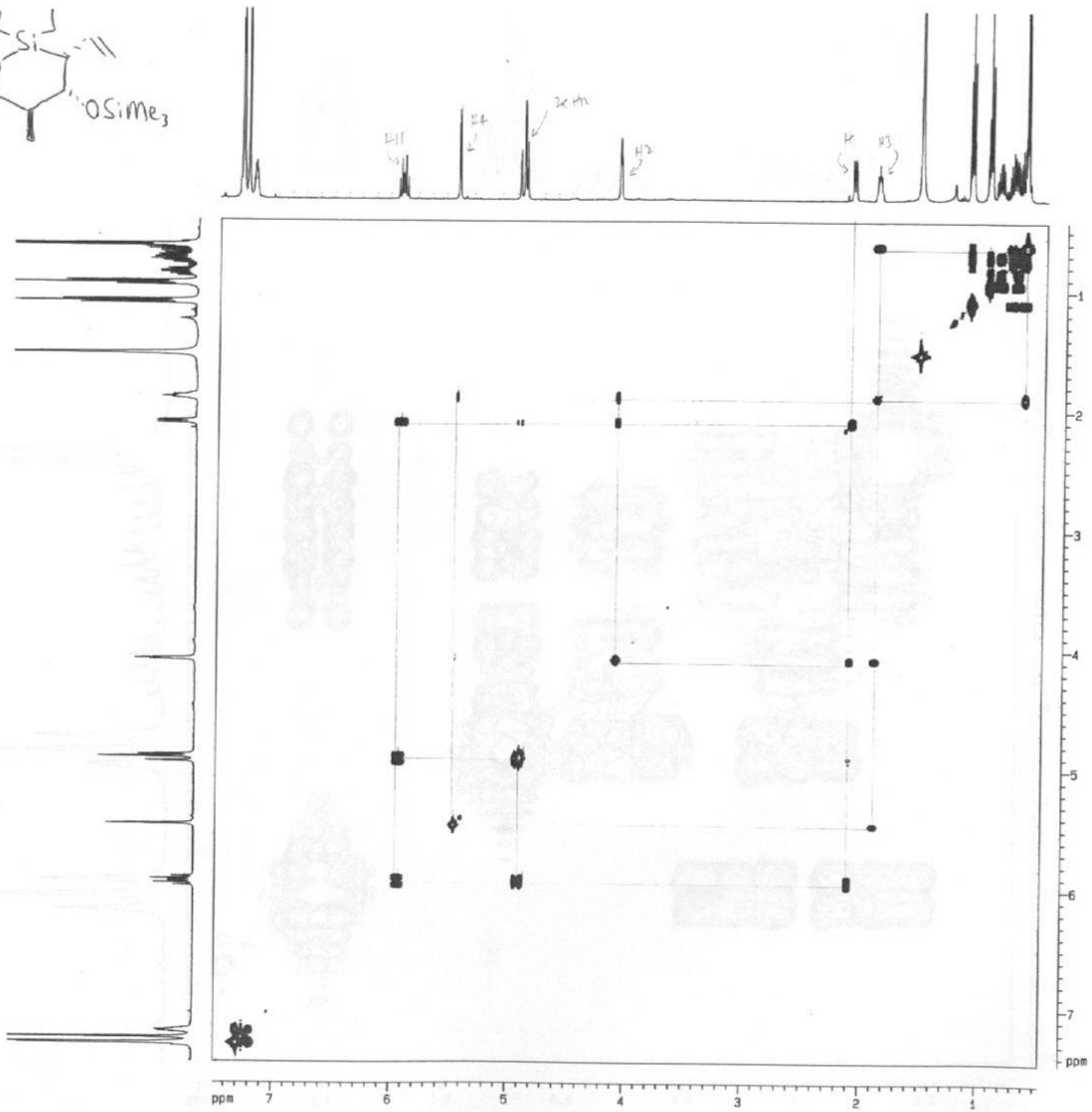

Current Data Parameters

NAME	sp09pj1d	
EXPNO	10	
PROCNO	1	

F2 - Acquisition Parameters

Date_	20050909	
Time	20.33	
INSTRUM	drx500	
PROBHD	5 mm TBI H/C	
PULPROG	cosygp	
TD	2048	
SOLVENT	CDC13	
NS	8	
DS	16	
SWH	4310.345	Hz
FIDRES	2.104661	Hz
AQ	0.2376180	sec
RG	1024	
DW	116.000	usec
DE	5.50	usec
TE	300.0	K
d0	0.00000300	sec
D1	2.00000000	sec
d13	0.00000300	sec
D16	0.00010000	sec
IN0	0.00023200	sec

============ CHANNEL f1 ============

NUC1	1H	
P0	10.70	usec
P1	10.70	usec
PL1	1.00	dB
SFO1	500.1318867	MHz

============ GRADIENT CHANNEL ========

GPNAM1	SINE.100	
GPNAM2	SINE.100	
GPX1	0.00	%
GPX2	0.00	%
GPY1	0.00	%
GPY2	0.00	%
GPZ1	10.00	%
GPZ2	10.00	%
P16	1000.00	usec

F1 - Acquisition parameters

ND0	1	
TD	512	
SFO1	500.1319	MHz
FIDRES	8.418642	Hz
SW	8.618	ppm

F2 - Processing parameters

SI	2048	
SF	500.1300233	MHz
WDW	SINE	
SSB	0	
LB	0.00	Hz
GB	0	
PC	1.00	

F1 - Processing parameters

SI	1024	
MC2	QF	
SF	500.1300233	MHz
WDW	SINE	
SSB	0	
LB	0.00	Hz
GB	0	

2D NMR plot parameters

CX2	20.00	cm
CX1	20.00	cm
F2PLO	7.488	ppm
F2LO	3745.03	Hz
F2PHI	0.456	ppm
F2HI	228.14	Hz
F1PLO	7.454	ppm
F1LO	3728.19	Hz
F1PHI	0.418	ppm
F1HI	209.20	Hz
F2PPMCM	0.35160	ppm/cm
F2HZCM	175.84441	Hz/cm
F1PPMCM	0.35181	ppm/cm
F1HZCM	175.94962	Hz/cm

17b

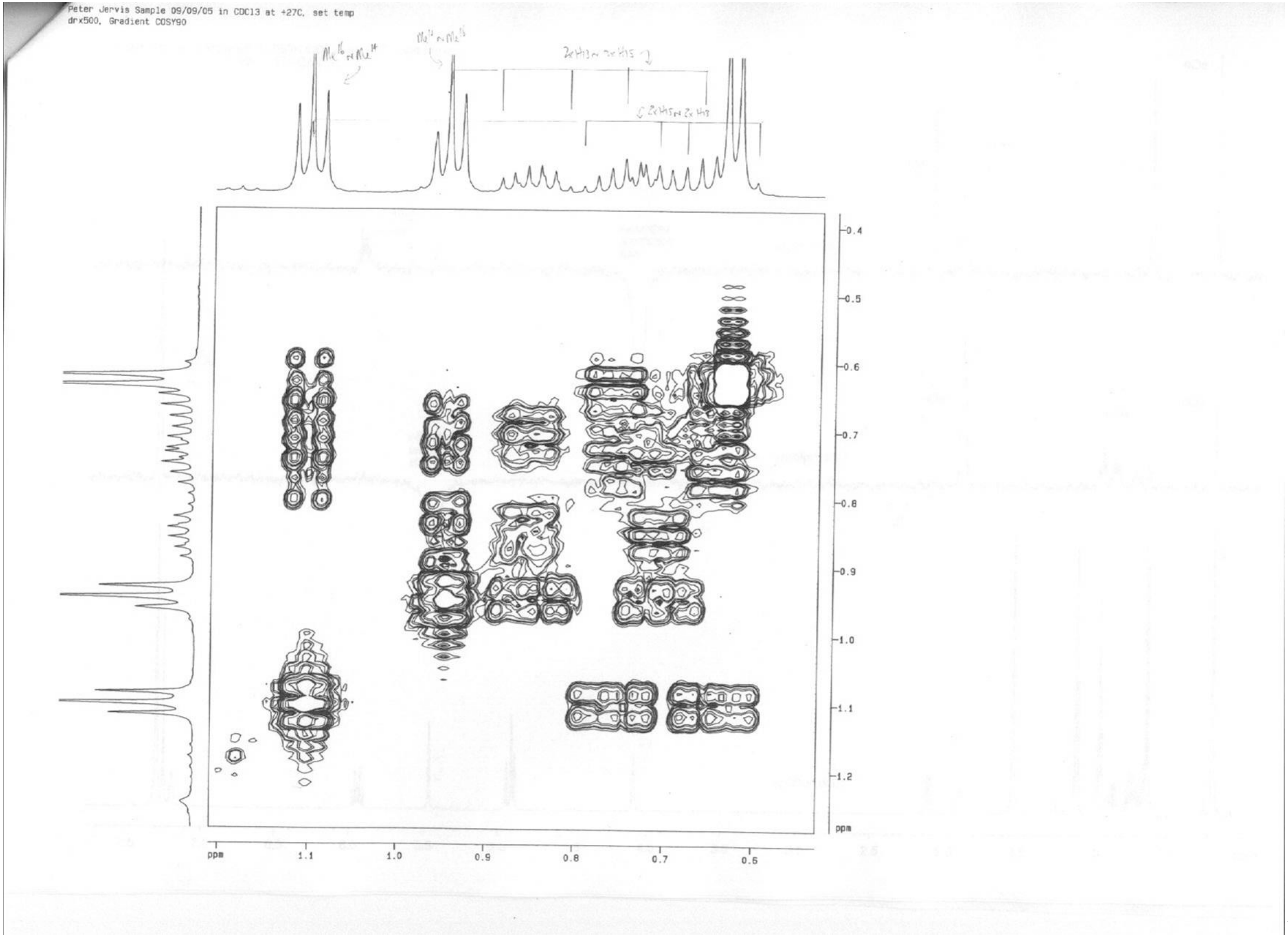
Peter Jervis Sample 09/09/05 in CDCl3 at +27C, set temp
drx500, Gradient COSY90
ppm
1.1
1.0
0.9
0.8
0.7
0.6
0.5
0.4
1.2

17b

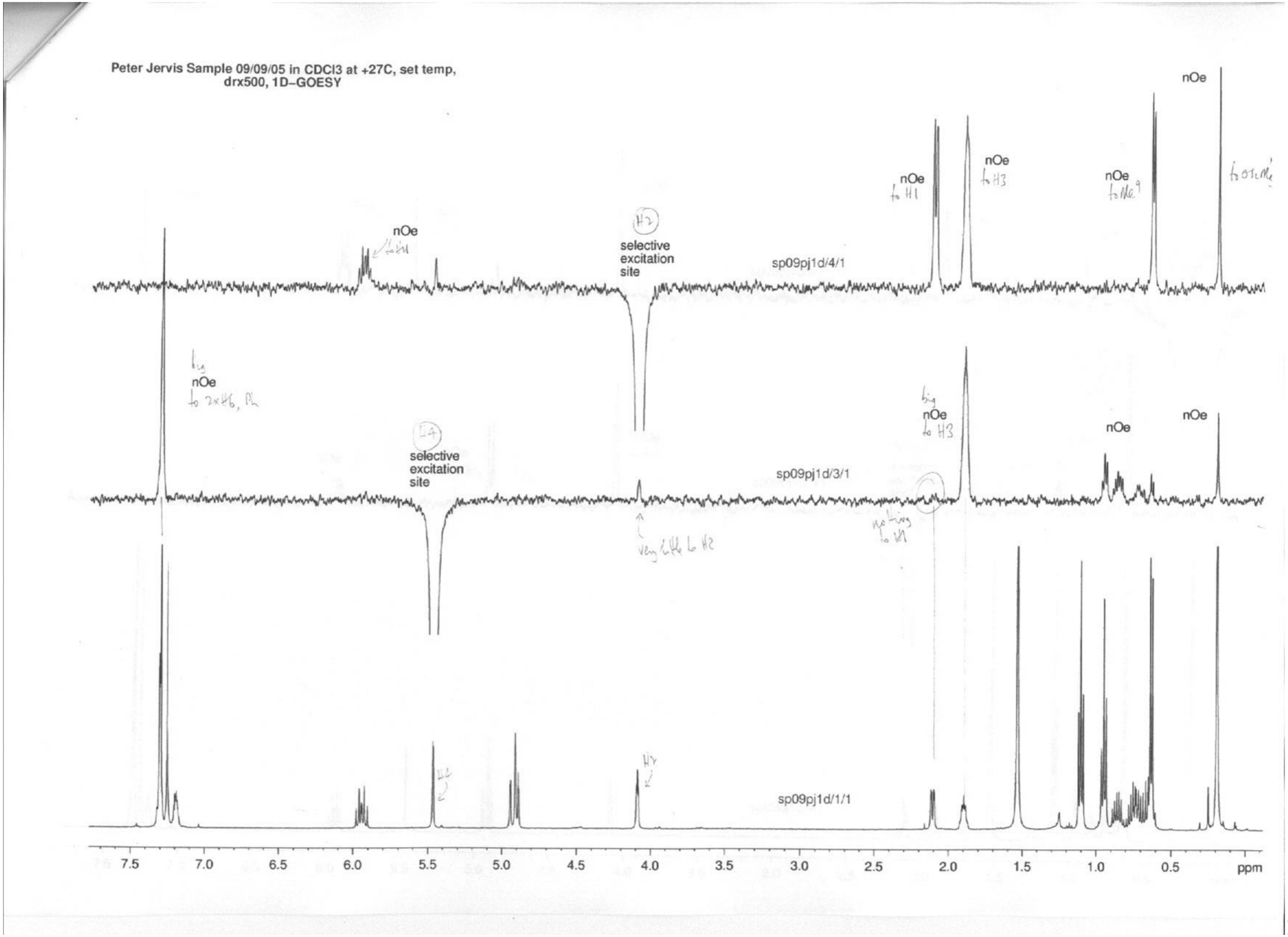
Peter Jervis Sample 09/09/05 in CDCl3 at +27C, set temp,
drx500, 1D-GOESY
nOe
selective
excitation
site
sp09pj1d/4/1
nOe
nOe
nOe
nOe
nOe
selective
excitation
site
sp09pj1d/3/1
nOe
nOe
nOe
sp09pj1d/1/1
7.5 7.0 6.5 6.0 5.5 5.0 4.5 4.0 3.5 3.0 2.5 2.0 1.5 1.0 0.5 ppm

17b

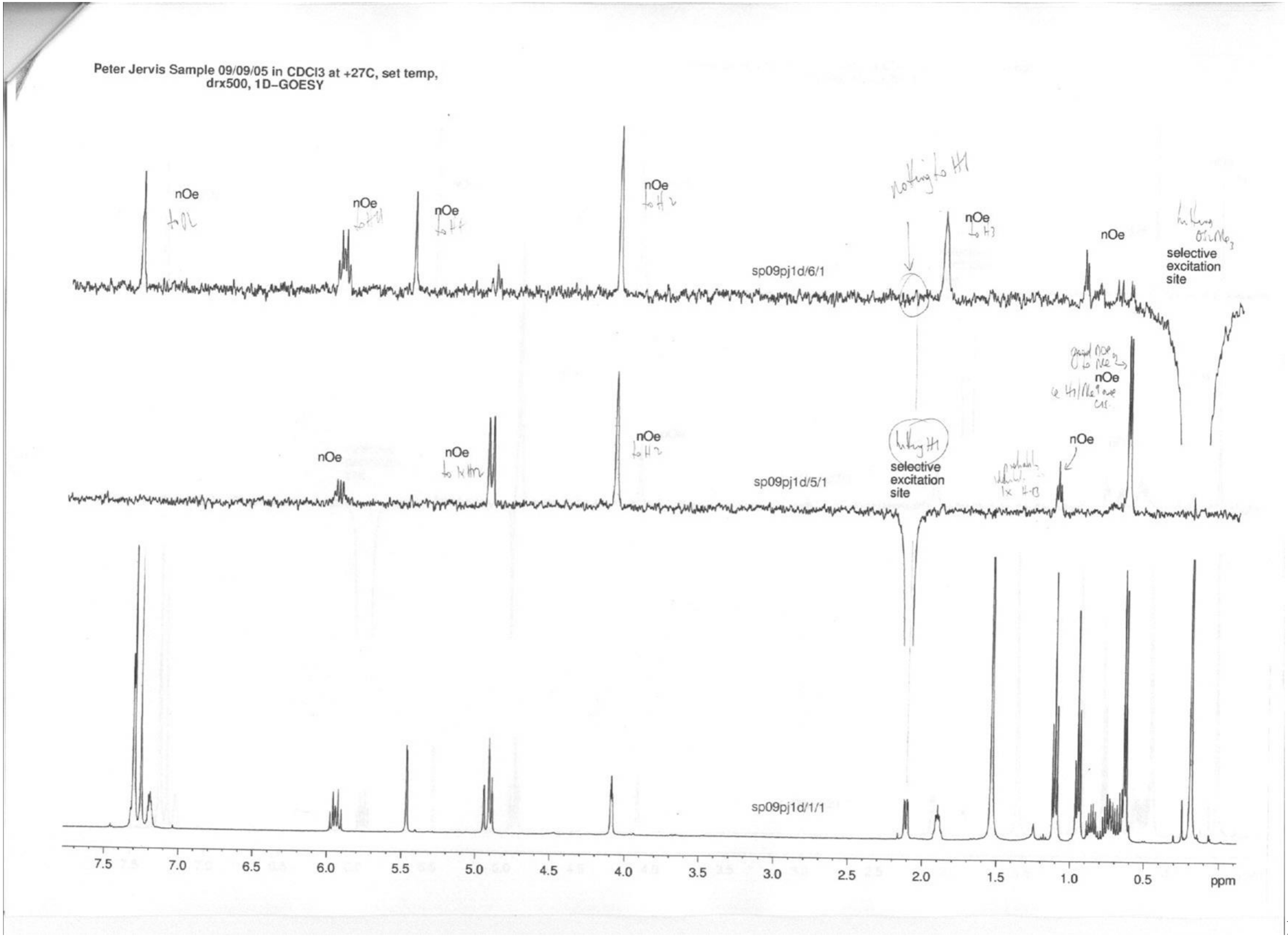
Peter Jervis Sample 09/09/05 in CDCl3 at +27C, set temp,
drx500, 1D-GOESY
nOe
nOe
nOe
nOe
nOe
nOe
selective
excitation
site
sp09pj1d/6/1
nOe
nOe
nOe
nOe
nOe
selective
excitation
site
sp09pj1d/5/1
sp09pj1d/1/1
7.5
7.0
6.5
6.0
5.5
5.0
4.5
4.0
3.5
3.0
2.5
2.0
1.5
1.0
0.5
ppm

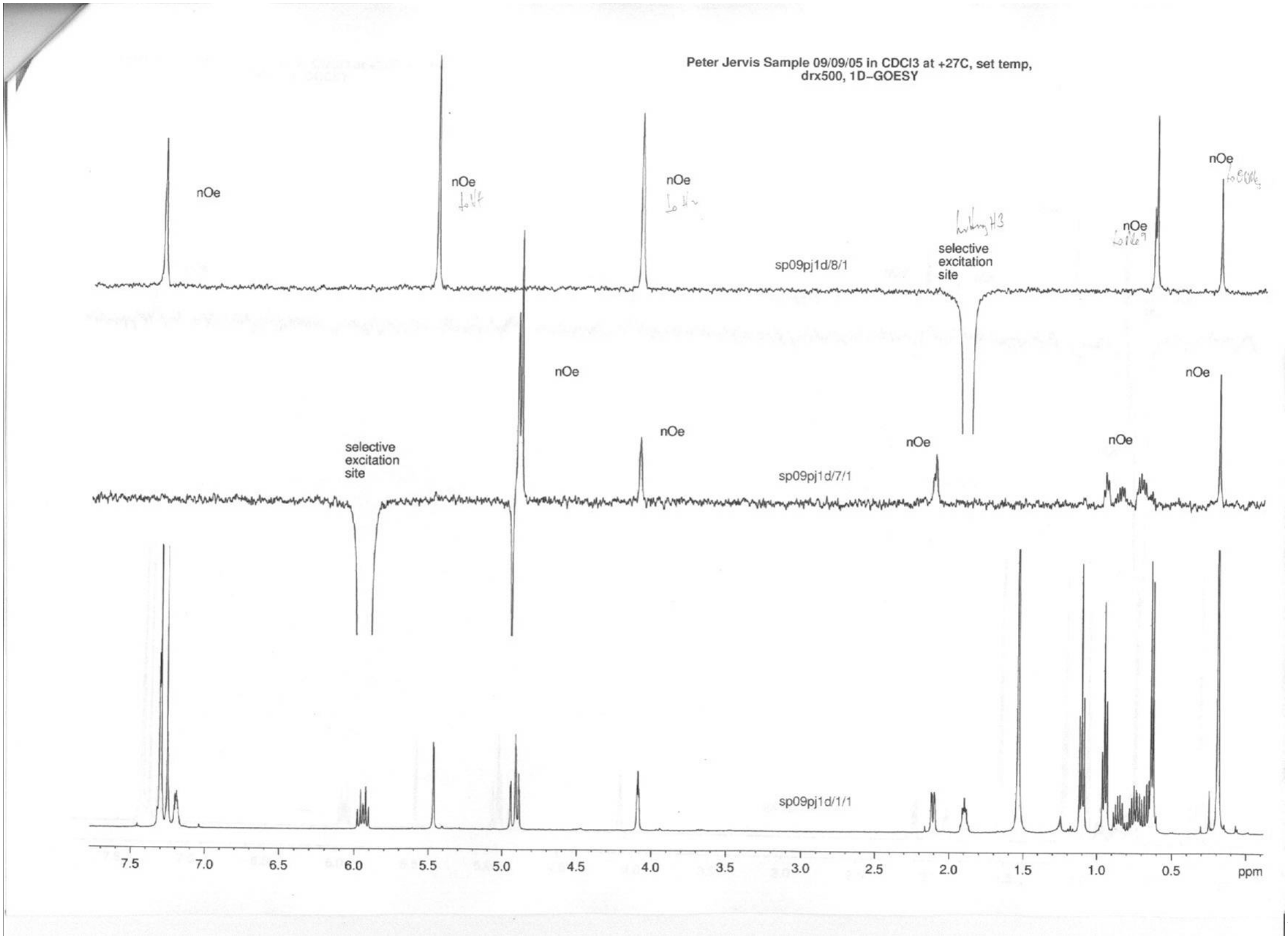
Peter Jervis Sample 09/09/05 in CDCl3 at +27C, set temp,
drx500, 1D-GOESY
nOe
nOe
nOe
selective
excitation
site
sp09pj1d/8/1
nOe
nOe
nOe
nOe
nOe
nOe
nOe
selective
excitation
site
sp09pj1d/7/1
sp09pj1d/1/1
7.5
7.0
6.5
6.0
5.5
5.0
4.5
4.0
3.5
3.0
2.5
2.0
1.5
1.0
0.5
ppm

17b

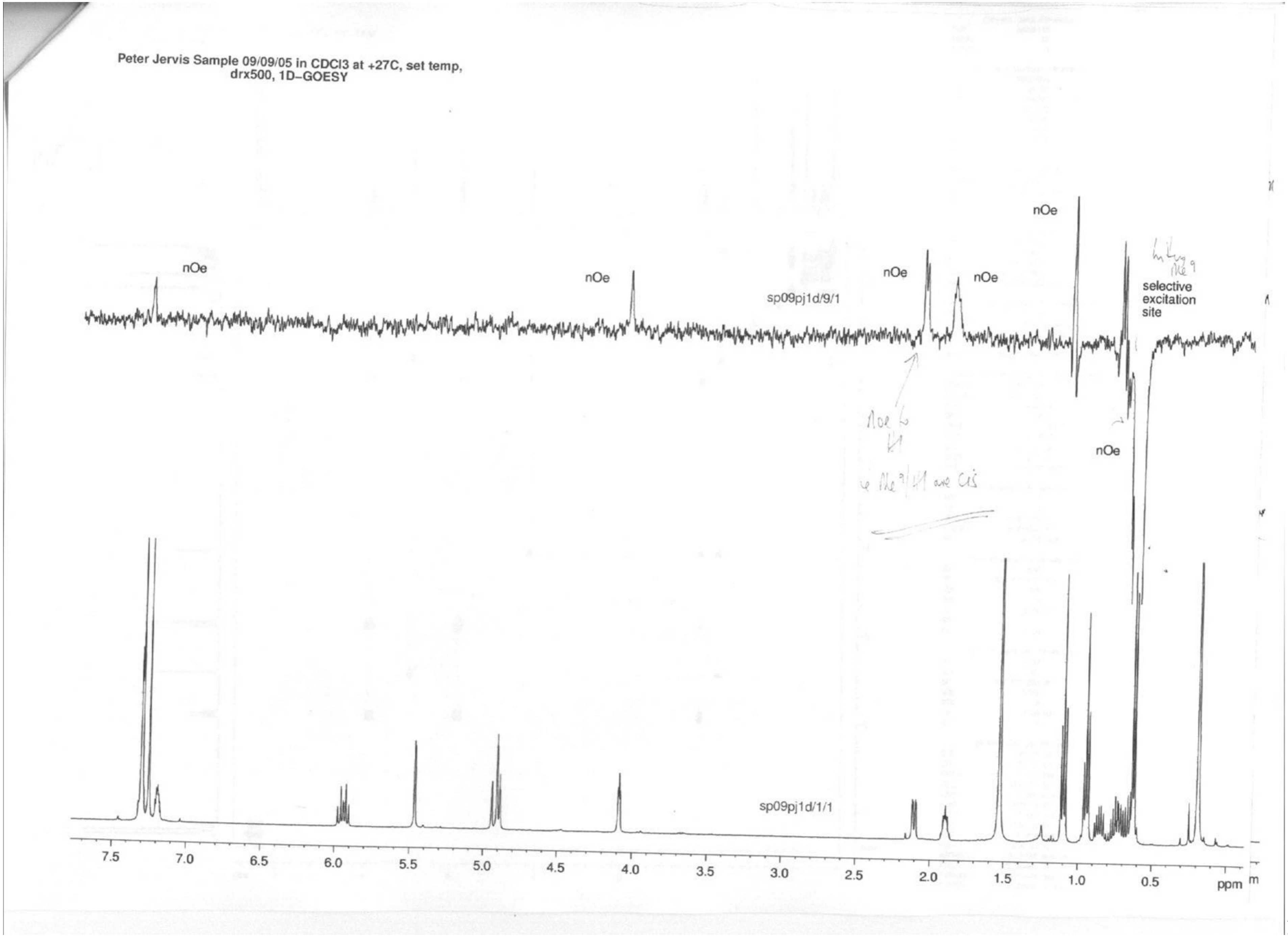
Peter Jervis Sample 09/09/05 in CDCl3 at +27C, set temp, drx500, 1D-GOESY
nOe
nOe
sp09pj1d/9/1
nOe
nOe
nOe
selective
excitation
site
nOe
sp09pj1d/1/1
7.5
7.0
6.5
6.0
5.5
5.0
4.5
4.0
3.5
3.0
2.5
2.0
1.5
1.0
0.5
ppm

17b
