# Supplementary material for: Tether-directed synthesis of highly substituted oxasilacycles via an intramolecular allylation employing allylsilanes
Source: Beilstein J Org Chem. 2007 Feb 8;3:6. doi: 10.1186/1860-5397-3-6 (PMC1810303; doi:10.1186/1860-5397-3-6)
Supplement: File 7 — 1H-NMR and 13C-NMR Spectra for the following compounds: 18b, 19b. [file Beilstein_J_Org_Chem-03-06-s007.pdf]

Additional File 7

Tether-directed synthesis of highly substituted oxasilacycles *via* an intramolecular allylation employing allylsilanes

Peter J. Jervis and Liam R. Cox*

email: l.r.cox@bham.ac.uk

^{1}H-NMR and ^{13}C-NMR Spectra for the following compounds:

18b, 19b

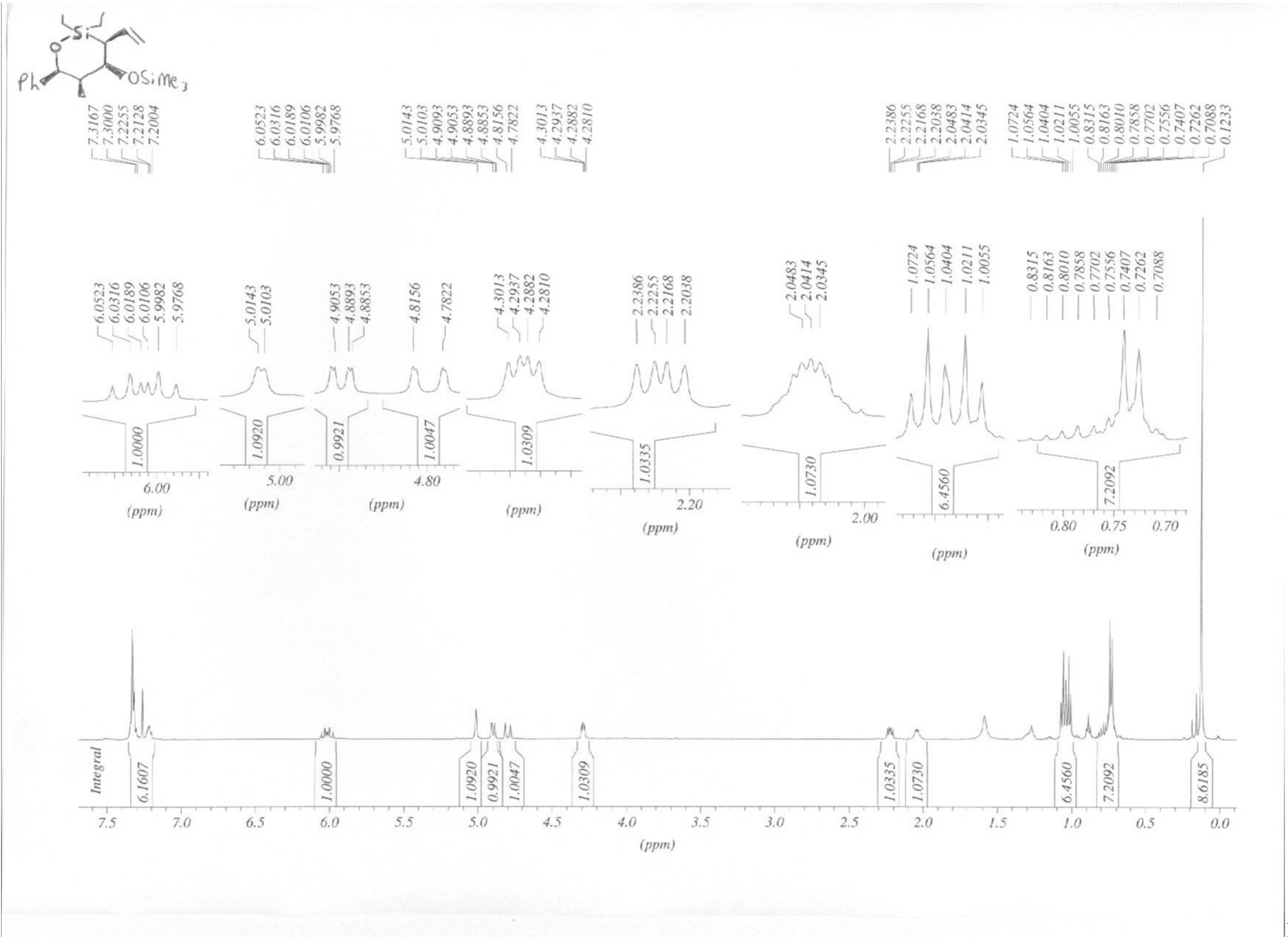

Ph
OSiMe3
7.3167
7.3000
7.2255
7.2128
7.2004
6.0523
6.0316
6.0189
6.0106
5.9982
5.9768
5.0143
5.0103
4.9093
4.9053
4.8893
4.8853
4.8156
4.7822
4.3013
4.2937
4.2882
4.2810
2.2386
2.2255
2.2168
2.2038
2.0483
2.0414
2.0345
1.0724
1.0564
1.0404
1.0211
1.0055
0.8315
0.8163
0.8010
0.7858
0.7702
0.7556
0.7407
0.7262
0.7088
0.1233
1.0000
1.0920
0.9921
1.0047
1.0309
1.0335
1.0730
6.4560
7.2092
(ppm)
Integral
6.1607
8.6185

18b

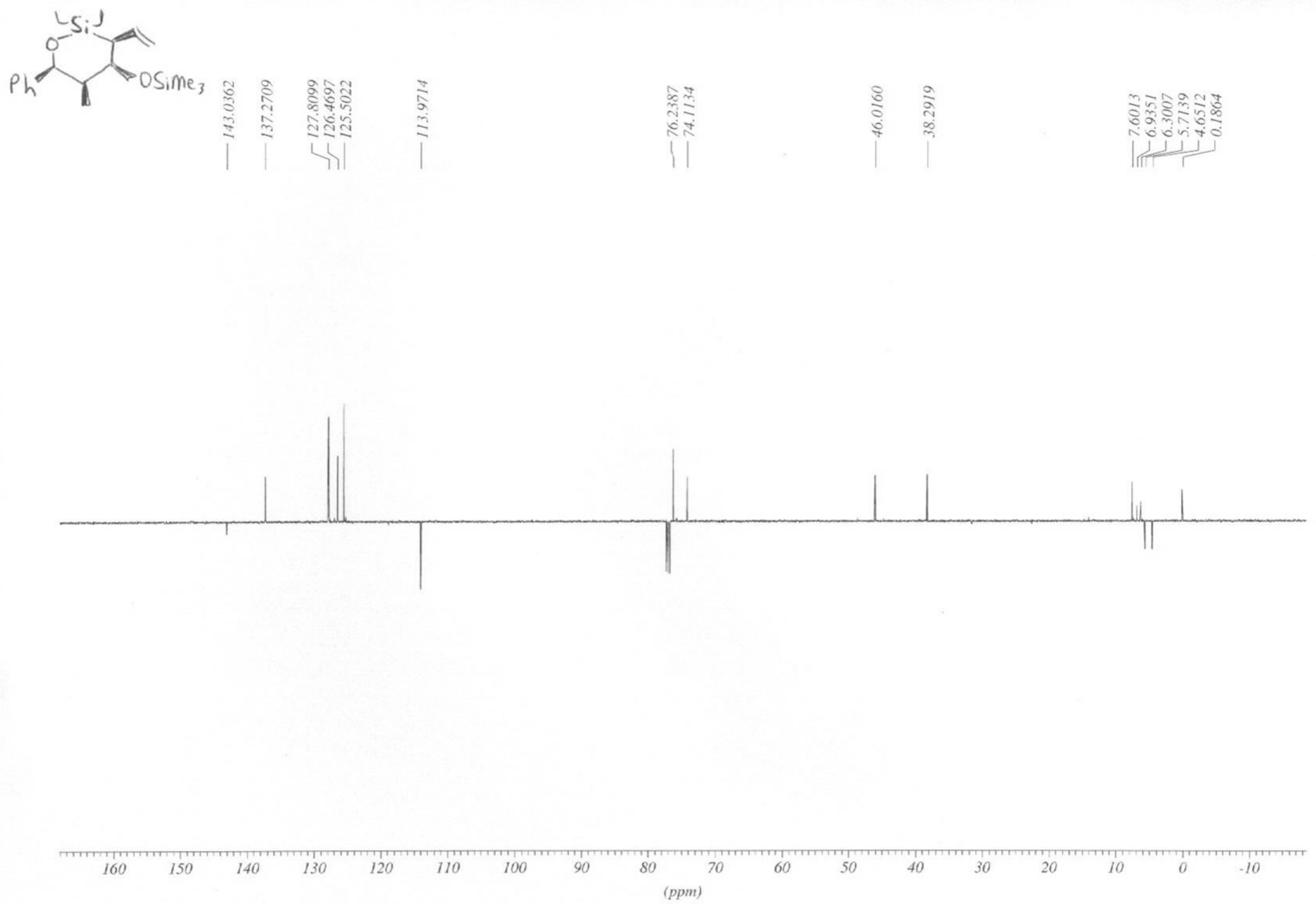

OSiMe3
Ph
143.0362
137.2709
127.8099
126.4697
125.5022
113.9714
76.2387
74.1134
46.0160
38.2919
7.6013
6.9351
6.3007
5.7139
4.6512
0.1864
160
150
140
130
120
110
100
90
80
70
60
50
40
30
20
10
0
-10
(ppm)

18b

Peter Jervis Sample 04/10/05 in CDCl3 at +27C, set temp
drx500, Gradient COSY90

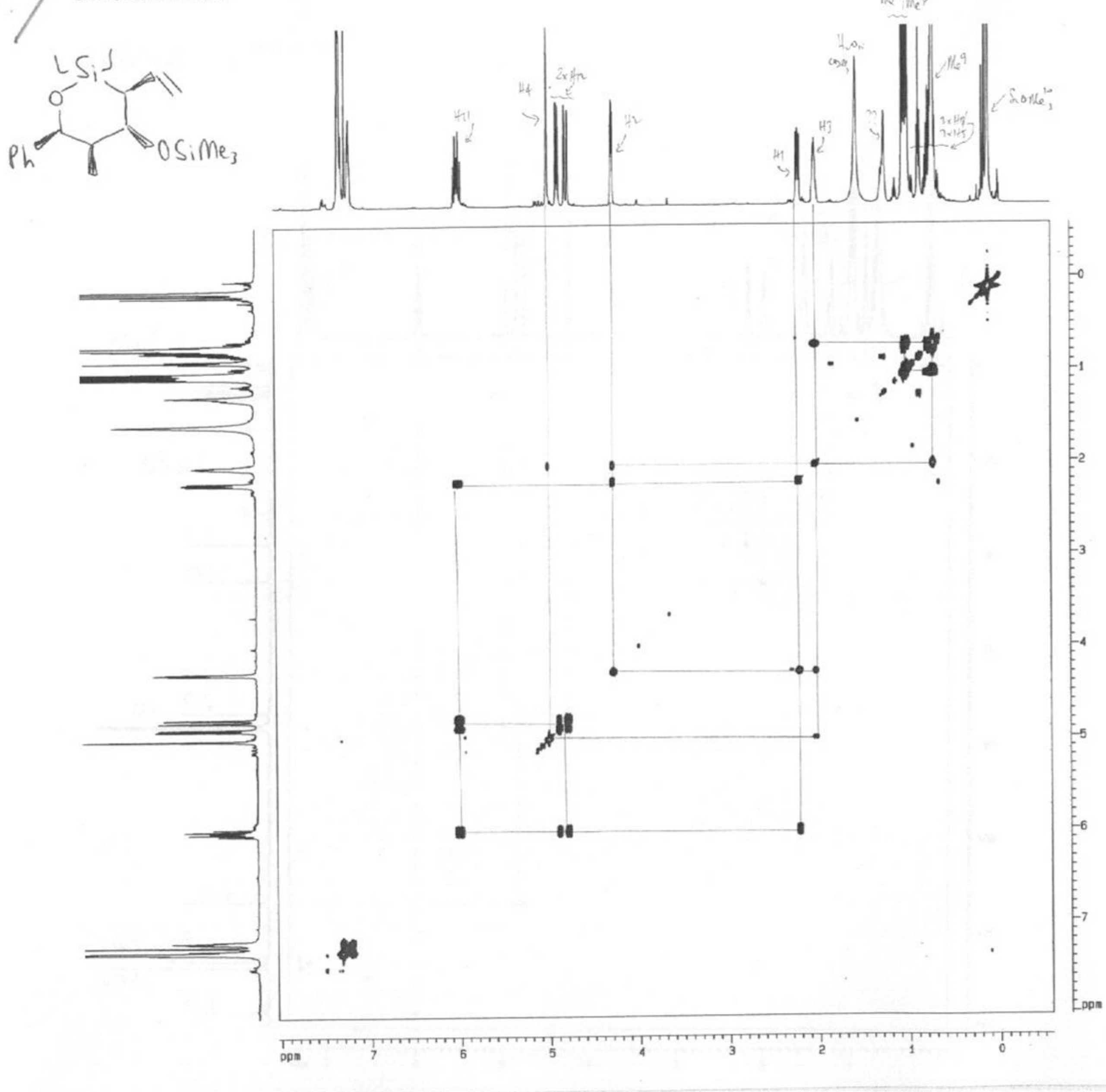

Current Data Parameters

NAME	oc04pj1d
EXPNO	10
PROCNO	1

F2 - Acquisition Parameters

Date_	20051005
Time	12.17
INSTRUM	drx500
PROBHD	5 mm TBI H/C
PULPROG	cosygp
TD	2048
SOLVENT	CDCl3
NS	8
DS	16
SWH	4310.345 Hz
FIDRES	2.104661 Hz
AQ	0.2376180 sec
RG	128
DW	116.000 usec
DE	5.50 usec
TE	300.0 K
d0	0.00000300 sec
D1	2.00000000 sec
d13	0.00000300 sec
D16	0.00010000 sec
IN0	0.00023200 sec

============ CHANNEL f1 ============

NUC1	1H
P0	10.70 usec
P1	10.70 usec
PL1	1.00 dB
SFO1	500.1318867 MHz

============ GRADIENT CHANNEL ========

GPNAM1	SINE.100
GPNAM2	SINE.100
GPX1	0.00 %
GPX2	0.00 %
GPY1	0.00 %
GPY2	0.00 %
GPZ1	10.00 %
GPZ2	10.00 %
P16	1000.00 usec

F1 - Acquisition parameters

ND0	1
TD	512
SFO1	500.1319 MHz
FIDRES	8.418642 Hz
SW	8.618 ppm

F2 - Processing parameters

SI	2048
SF	500.1300233 MHz
WDW	SINE
SSB	0
LB	0.00 Hz
GB	0
PC	1.00

F1 - Processing parameters

SI	1024
MC2	QF
SF	500.1300233 MHz
WDW	SINE
SSB	0
LB	0.00 Hz
GB	0

2D NMR plot parameters

CX2	20.00 cm
CX1	20.00 cm
F2PLO	8.035 ppm
F2LO	4018.63 Hz
F2PHI	-0.583 ppm
F2HI	-291.71 Hz
F1PLO	8.035 ppm
F1LO	4018.63 Hz
F1PHI	-0.583 ppm
F1HI	-291.71 Hz
F2PPMCM	0.43092 ppm/cm
F2HZCM	215.51724 Hz/cm
F1PPMCM	0.43092 ppm/cm
F1HZCM	215.51724 Hz/cm

18b

Peter Jervis Sample 04/10/05 in CDCl3 at +27C, set temp
drx500, Gradient HSGC

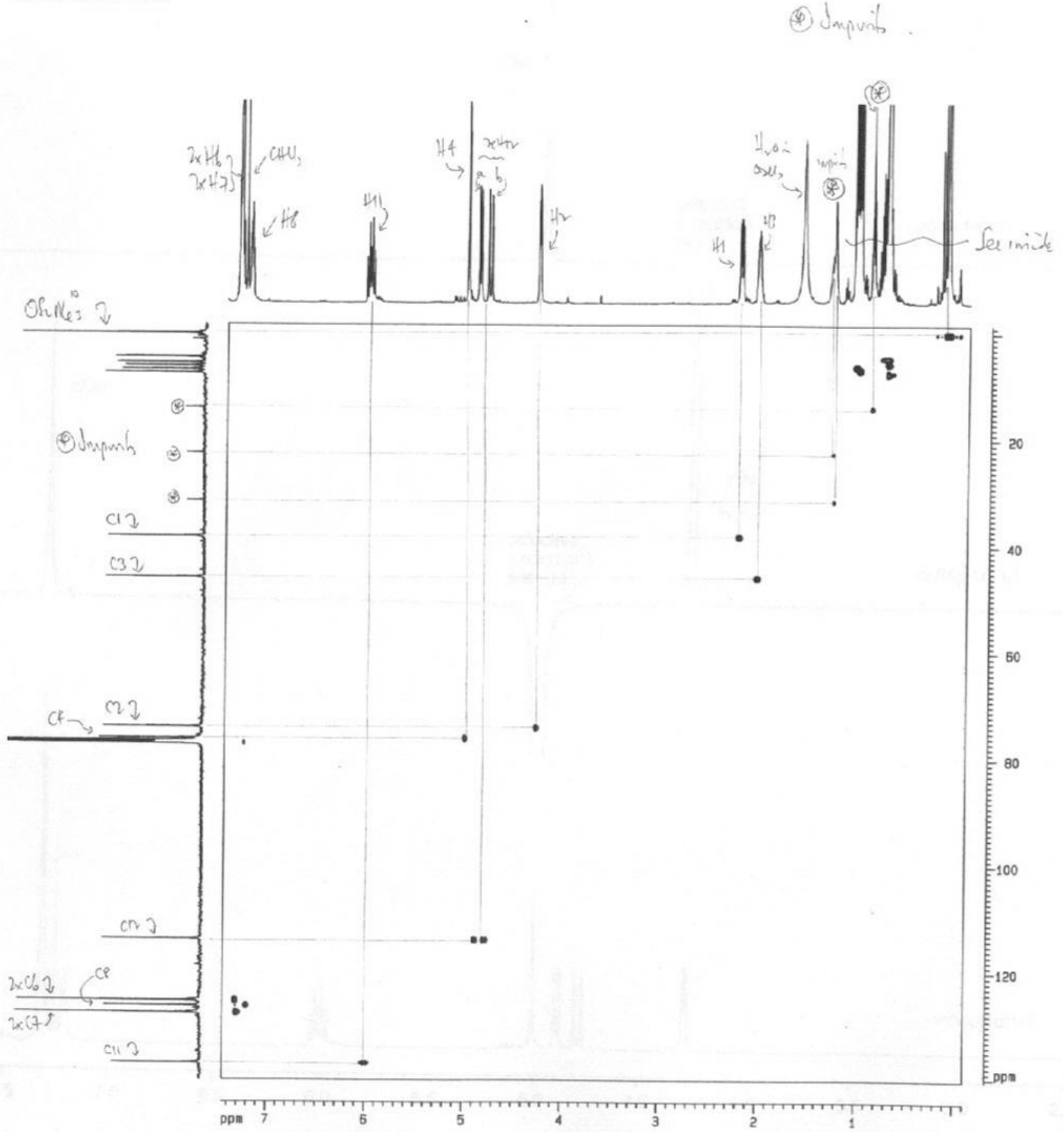

Current Data Parameters
NAME ocO4pj1d
EXPNO 2
PROCNO 1

F2 - Acquisition Parameters
Date_ 20051004
Time 16.39
INSTRUM drx500
PROBHD 5 mm TBI H/C
PULPROG invigptp
TD 2048
SOLVENT CDCl3
NS 8
DS 16
SWH 4310.345 Hz
FIDRES 2.104661 Hz
AQ 0.2376180 sec
RG 32768
DW 116.000 usec
DE 5.50 usec
TE 300.0 K
CNST2 145.0000000
d0 0.00000300 sec
D1 2.00000000 sec
d4 0.00172414 sec
d11 0.03000000 sec
d13 0.00000300 sec
D16 0.00010000 sec
d20 0.00110000 sec
d21 0.00061714 sec
IN0 0.00001140 sec

============ CHANNEL f1 ============
NUC1 1H
P1 10.70 usec
p2 21.40 usec
PL1 1.00 dB
SFO1 500.1318867 MHz

============ CHANNEL f2 ============
CPDPRG2 garp
NUC2 13C
P3 12.00 usec
p4 24.00 usec
PCPD2 76.00 usec
PL2 -1.00 dB
PL12 15.00 dB
SFO2 125.7687893 MHz

============ GRADIENT CHANNEL ========
GPNAM1 SINE.100
GPNAM2 SINE.100
GPNAM3 SINE.100
GPX1 0.00 %
GPX2 0.00 %
GPX3 0.00 %
GPY1 0.00 %
GPY2 0.00 %
GPY3 0.00 %
GPZ1 80.00 %
GPZ2 30.00 %
GPZ3 20.10 %
P16 1000.00 usec

F1 - Acquisition parameters
ND0 4
TD 512
SFO1 125.7688 MHz
FIDRES 42.831688 Hz
SW 174.369 ppm

F2 - Processing parameters
SI 2048
SF 500.1300233 MHz
WDW QSINE
SSB 2
LB 0.00 Hz
GB 0
PC 1.00

F1 - Processing parameters
SI 1024
MC2 TPPI
SF 125.7577945 MHz
WDW QSINE
SSB 2
LB 0.00 Hz
GB 0

2D NMR plot parameters
CX2 17.00 cm
CX1 17.00 cm
F2PLO 7.450 ppm
F2LO 3726.08 Hz
F2PHI -0.104 ppm
F2HI -51.78 Hz
F1PLO 140.494 ppm
F1LO 17668.25 Hz
F1PHI -1.531 ppm
F1HI -192.56 Hz
F2PPMCM 0.44434 ppm/cm
F2HZCM 222.22740 Hz/cm
F1PPMCM 8.35444 ppm/cm
F1HZCM 1050.63611 Hz/cm

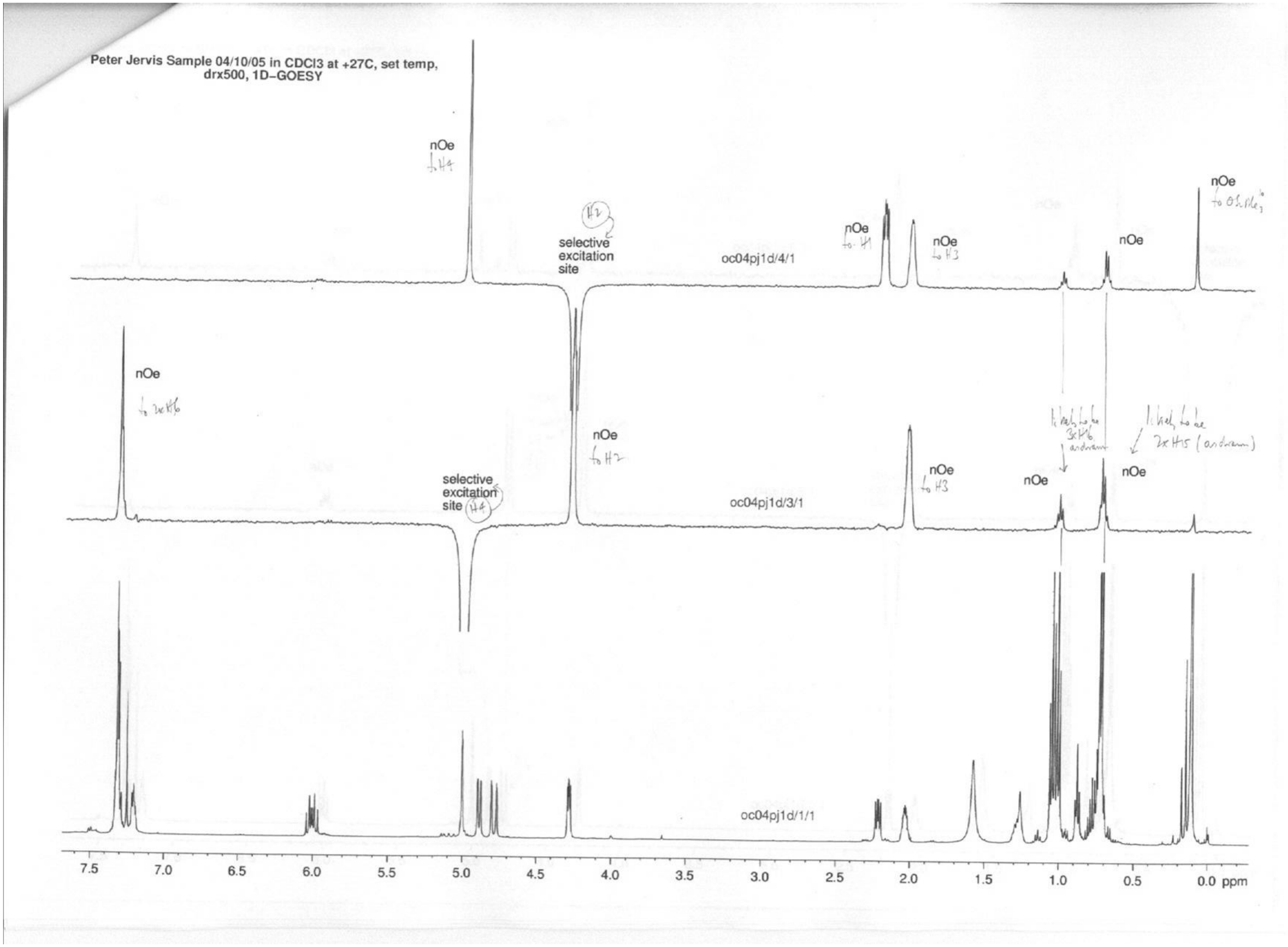
Peter Jervis Sample 04/10/05 in CDCl3 at +27C, set temp,
drx500, 1D-GOESY
nOe
selective
excitation
site
oc04pj1d/4/1
nOe
nOe
nOe
nOe
nOe
nOe
selective
excitation
site
oc04pj1d/3/1
nOe
nOe
nOe
oc04pj1d/1/1
7.5 7.0 6.5 6.0 5.5 5.0 4.5 4.0 3.5 3.0 2.5 2.0 1.5 1.0 0.5 0.0 ppm

18b

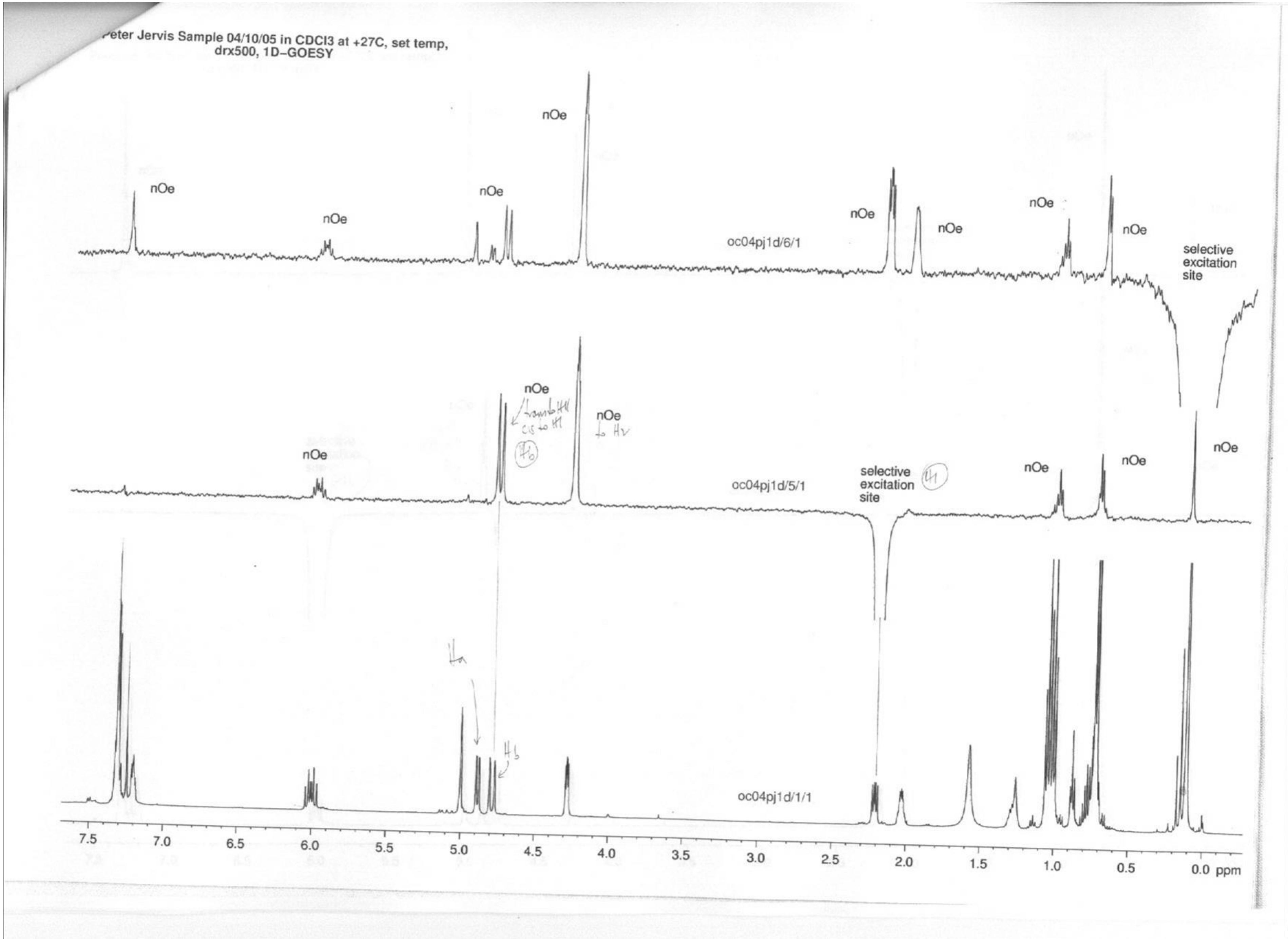

Peter Jervis Sample 04/10/05 in CDCl3 at +27C, set temp,
drx500, 1D–GOESY
nOe
nOe
nOe
nOe
oc04pj1d/6/1
nOe
nOe
nOe
nOe
selective
excitation
site
nOe
nOe
to H2
nOe
oc04pj1d/5/1
selective
excitation
site
nOe
nOe
nOe
Ha
Hb
oc04pj1d/1/1
7.5
7.0
6.5
6.0
5.5
5.0
4.5
4.0
3.5
3.0
2.5
2.0
1.5
1.0
0.5
0.0 ppm

18b

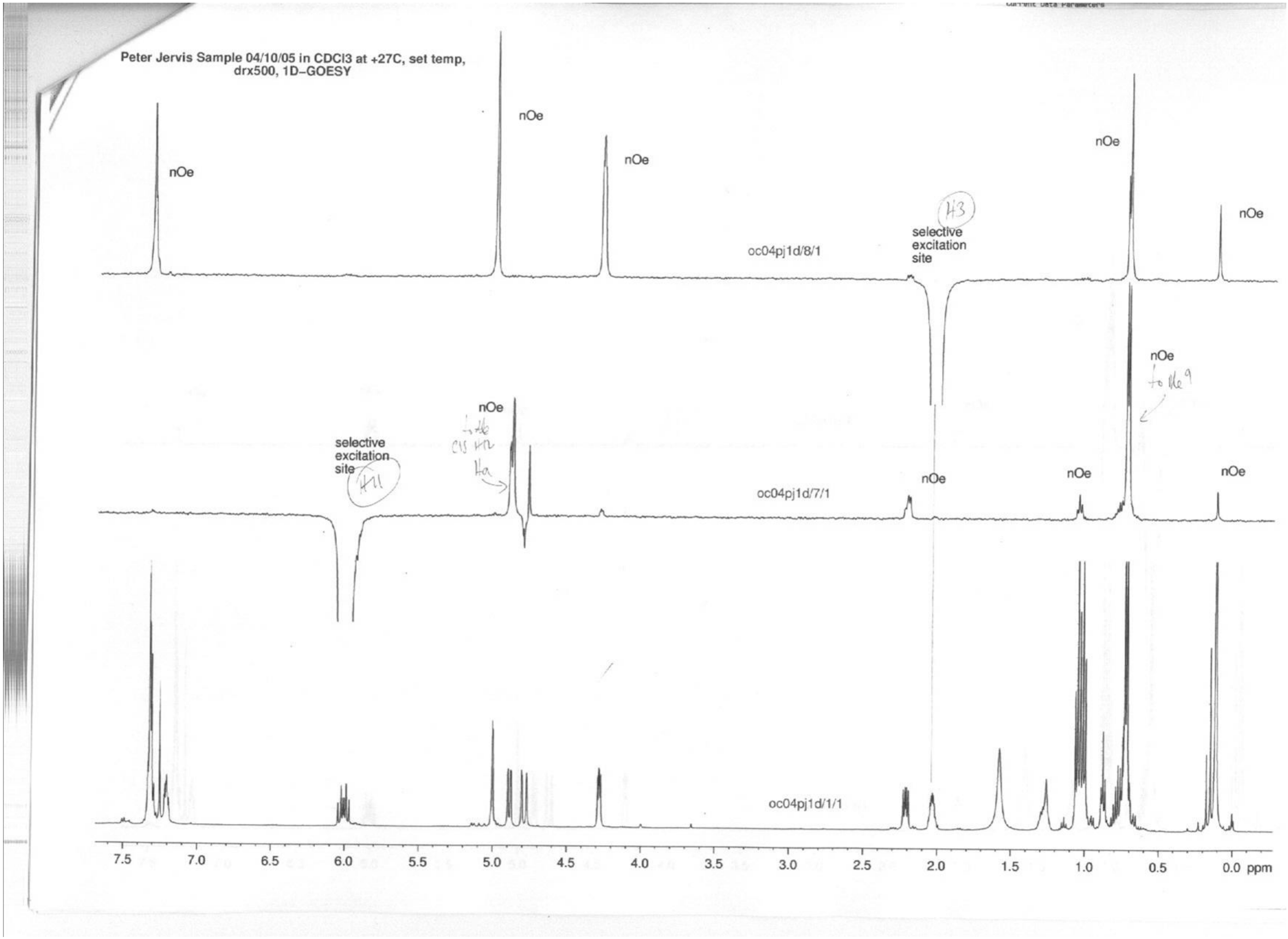
Peter Jervis Sample 04/10/05 in CDCl3 at +27C, set temp,
drx500, 1D–GOESY
nOe
nOe
nOe
nOe
nOe
H3
selective
excitation
site
oc04pj1d/8/1
nOe
nOe
selective
excitation
site
oc04pj1d/7/1
nOe
nOe
nOe
oc04pj1d/1/1
7.5
7.0
6.5
6.0
5.5
5.0
4.5
4.0
3.5
3.0
2.5
2.0
1.5
1.0
0.5
0.0 ppm

18b

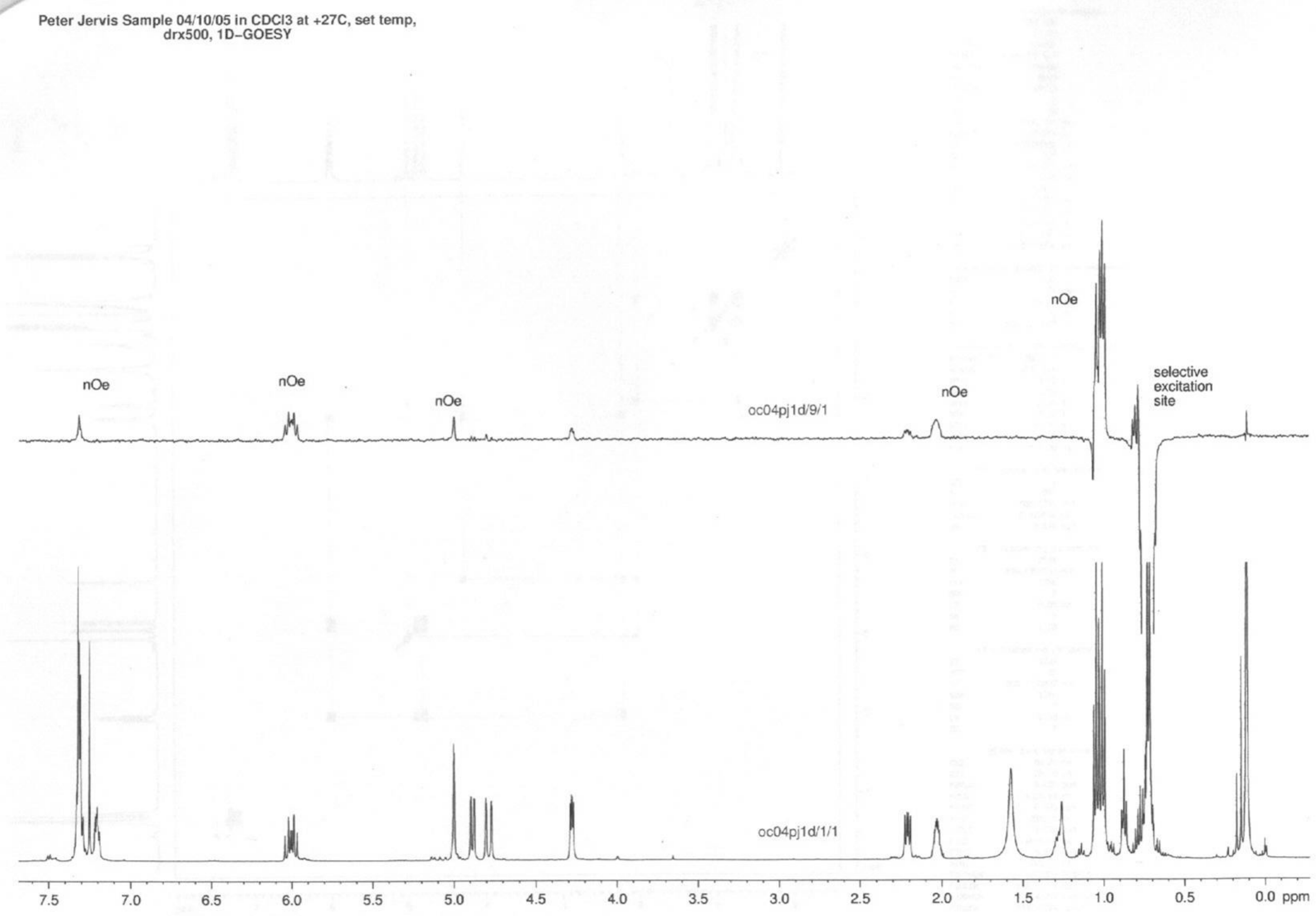
Peter Jervis Sample 04/10/05 in CDCl3 at +27C, set temp,
drx500, 1D-GOESY
nOe
nOe
nOe
oc04pj1d/9/1
nOe
nOe
selective
excitation
site
oc04pj1d/1/1
7.5
7.0
6.5
6.0
5.5
5.0
4.5
4.0
3.5
3.0
2.5
2.0
1.5
1.0
0.5
0.0 ppm

18b

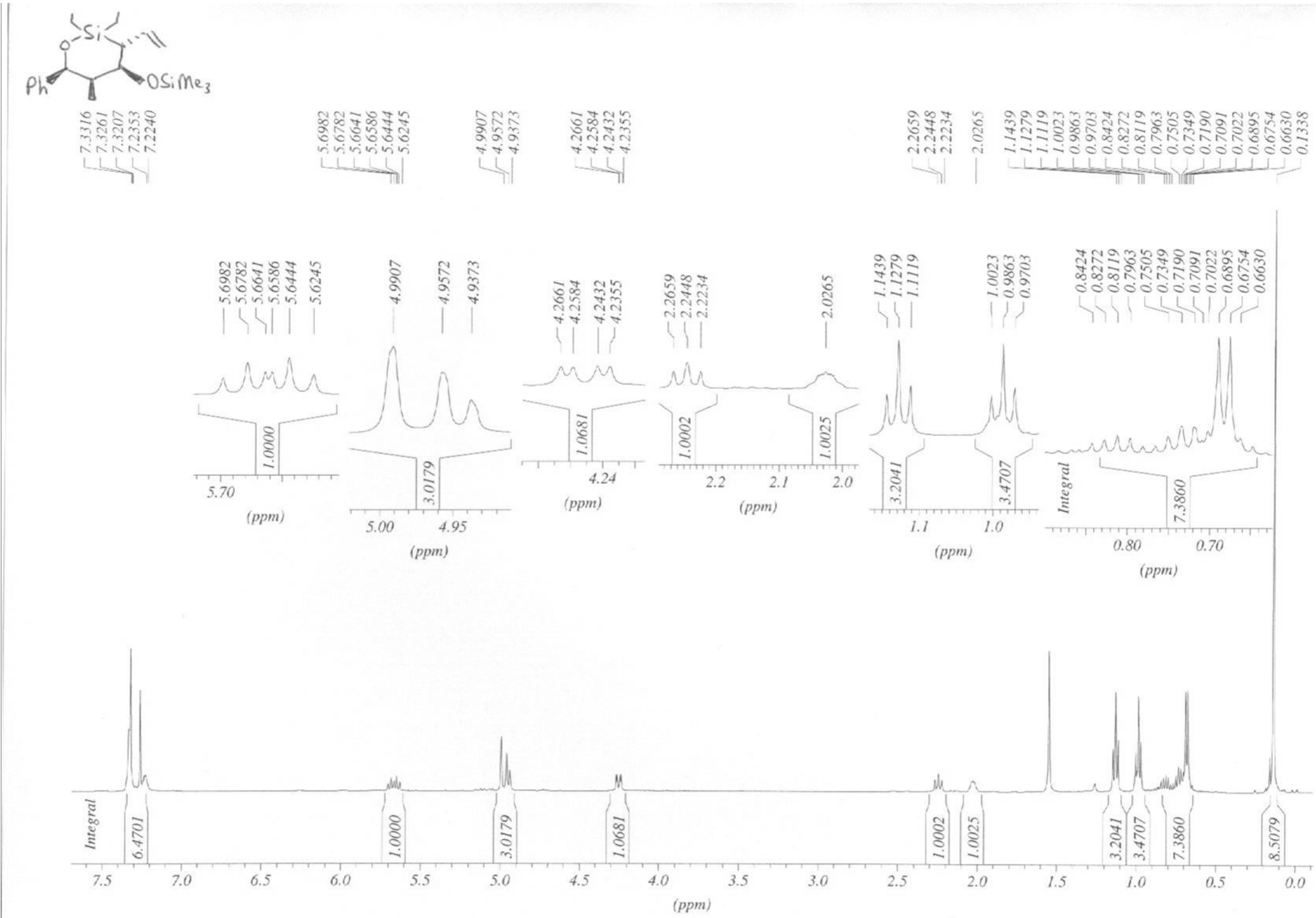
Ph
OSiMe3
7.3316
7.3261
7.3207
7.2353
7.2240
5.6982
5.6782
5.6641
5.6586
5.6444
5.6245
4.9907
4.9572
4.9373
4.2661
4.2584
4.2432
4.2355
2.2659
2.2448
2.2234
2.0265
1.1439
1.1279
1.1119
1.0023
0.9863
0.9703
0.8424
0.8272
0.8119
0.7963
0.7505
0.7349
0.7190
0.7091
0.7022
0.6895
0.6754
0.6630
0.1338
Integral
6.4701
1.0000
3.0179
1.0681
1.0002
1.0025
3.2041
3.4707
7.3860
8.5079
(ppm)

19b

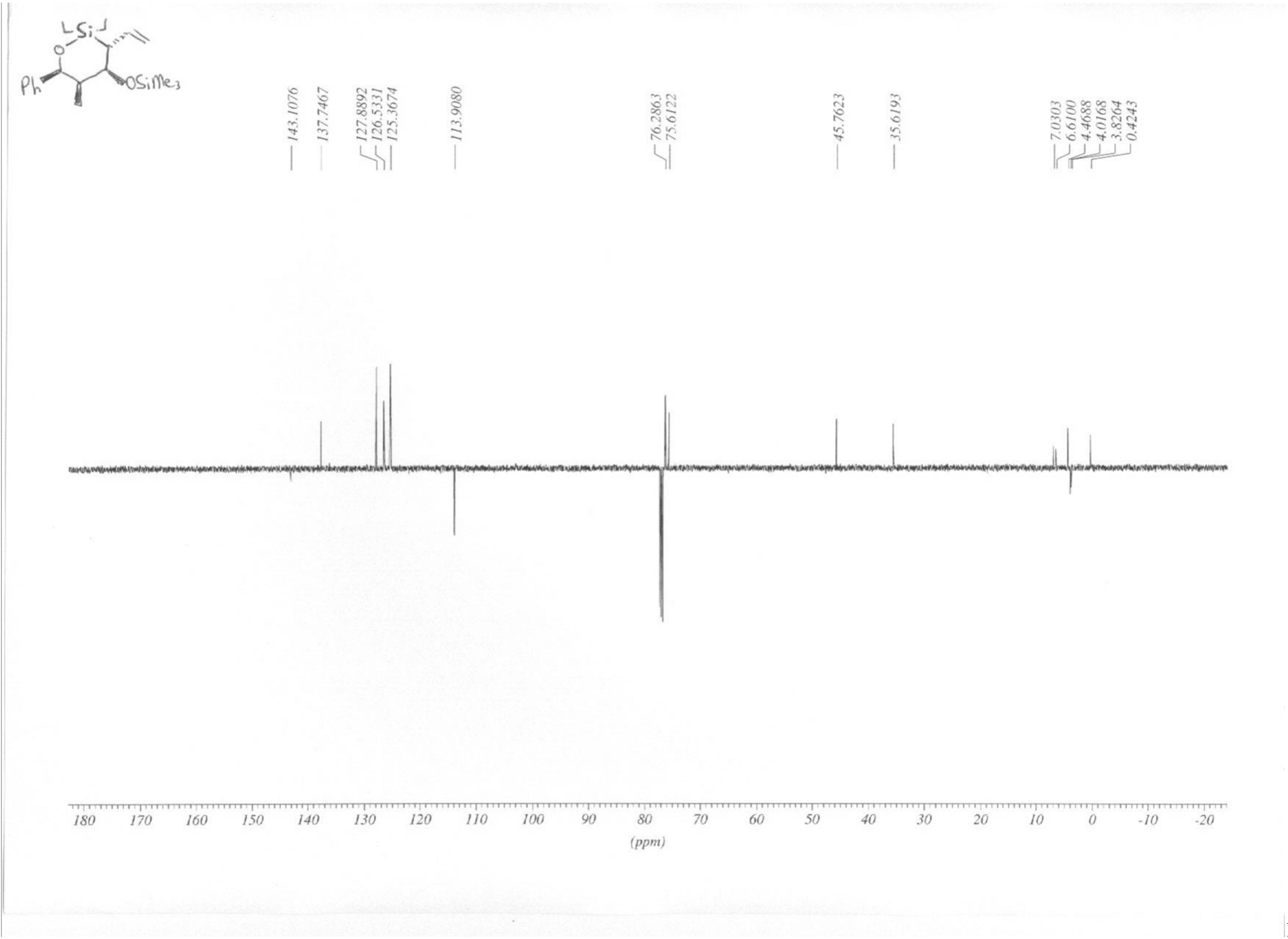
Ph
OSiMe3
143.1076
137.7467
127.8892
126.5331
125.3674
113.9080
76.2863
75.6122
45.7623
35.6193
7.0303
6.6100
4.4688
4.0168
3.8264
0.4243
180 170 160 150 140 130 120 110 100 90 80 70 60 50 40 30 20 10 0 -10 -20
(ppm)

19b

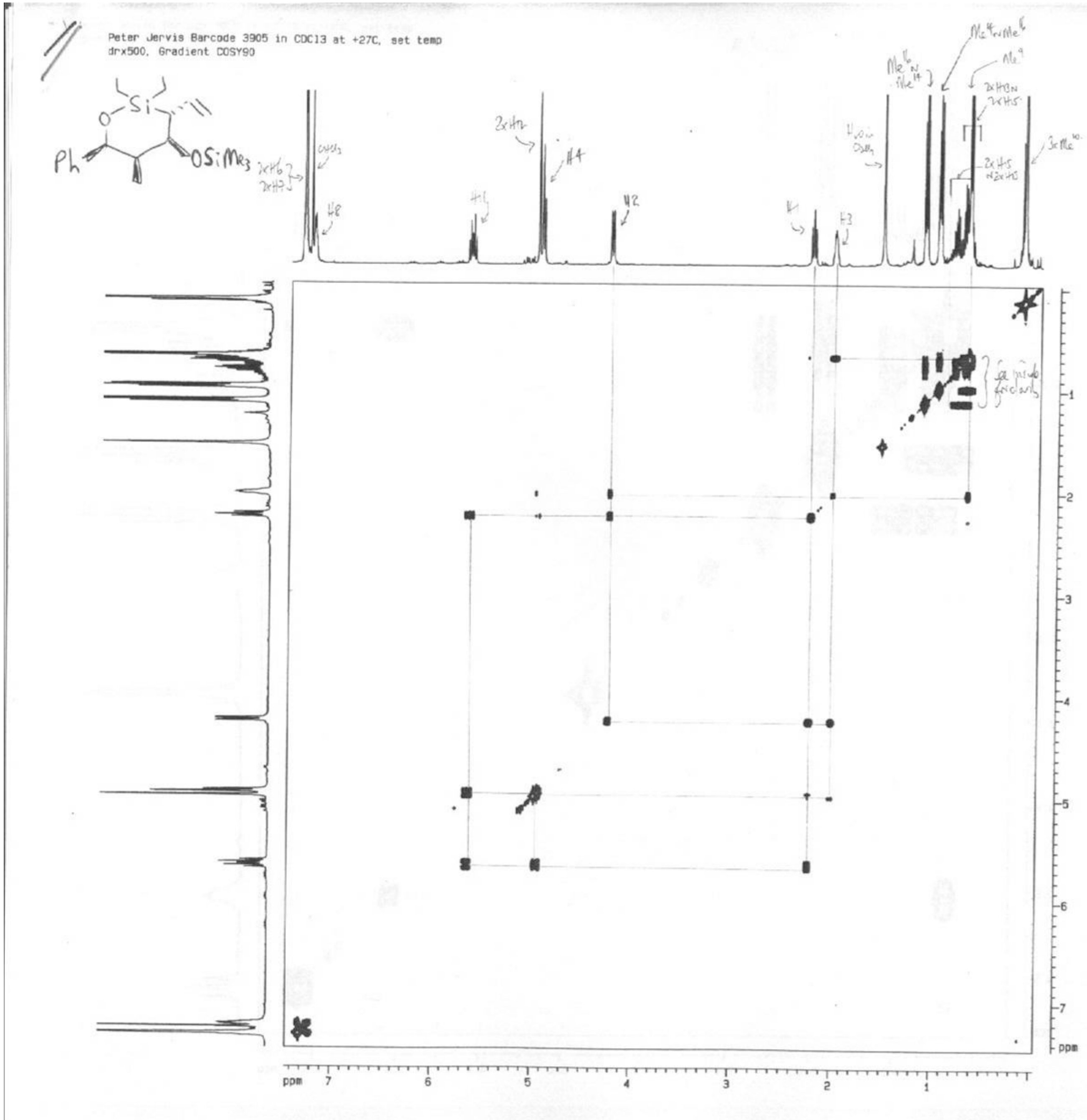

Current Data Parameters

NAME	j126pj1d
EXPNO	3
PROCNO	1

F2 - Acquisition Parameters

Date_	20050725
Time	19.47
INSTRUM	drx500
PROBHD	5 mm TBI H/C
PULPROG	cosygp
TD	2048
SOLVENT	CDCl3
NS	8
DS	16
SWH	4310.345 Hz
FIDRES	2.104661 Hz
AQ	0.2376180 sec
RG	456.1
DW	116.000 usec
DE	5.50 usec
TE	300.0 K
d0	0.00000300 sec
D1	2.00000000 sec
d13	0.00000300 sec
D16	0.00010000 sec
IN0	0.00023200 sec

============ CHANNEL f1 ============

NUC1	1H
P0	10.60 usec
P1	10.60 usec
PL1	1.00 dB
SFO1	500.1318867 MHz

============ GRADIENT CHANNEL ========

GPNAM1	SINE.100
GPNAM2	SINE.100
GPX1	0.00 %
GPX2	0.00 %
GPY1	0.00 %
GPY2	0.00 %
GPZ1	10.00 %
GPZ2	10.00 %
P16	1000.00 usec

F1 - Acquisition parameters

ND0	1
TD	512
SFO1	500.1319 MHz
FIDRES	8.418642 Hz
SW	8.618 ppm

F2 - Processing parameters

SI	2048
SF	500.1300233 MHz
WDW	SINE
SSB	0
LB	0.00 Hz
GB	0
PC	1.00

F1 - Processing parameters

SI	1024
MC2	QF
SF	500.1300233 MHz
WDW	SINE
SSB	0
LB	0.00 Hz
GB	0

2D NMR plot parameters

CX2	20.00 cm
CX1	20.00 cm
F2PLO	7.450 ppm
F2LO	3726.08 Hz
F2PHI	-0.049 ppm
F2HI	-24.42 Hz
F1PLO	7.454 ppm
F1LO	3728.19 Hz
F1PHI	-0.036 ppm
F1HI	-18.11 Hz
F2PPMCM	0.37495 ppm/cm
F2HZCM	187.52527 Hz/cm
F1PPMCM	0.37453 ppm/cm
F1HZCM	187.31480 Hz/cm

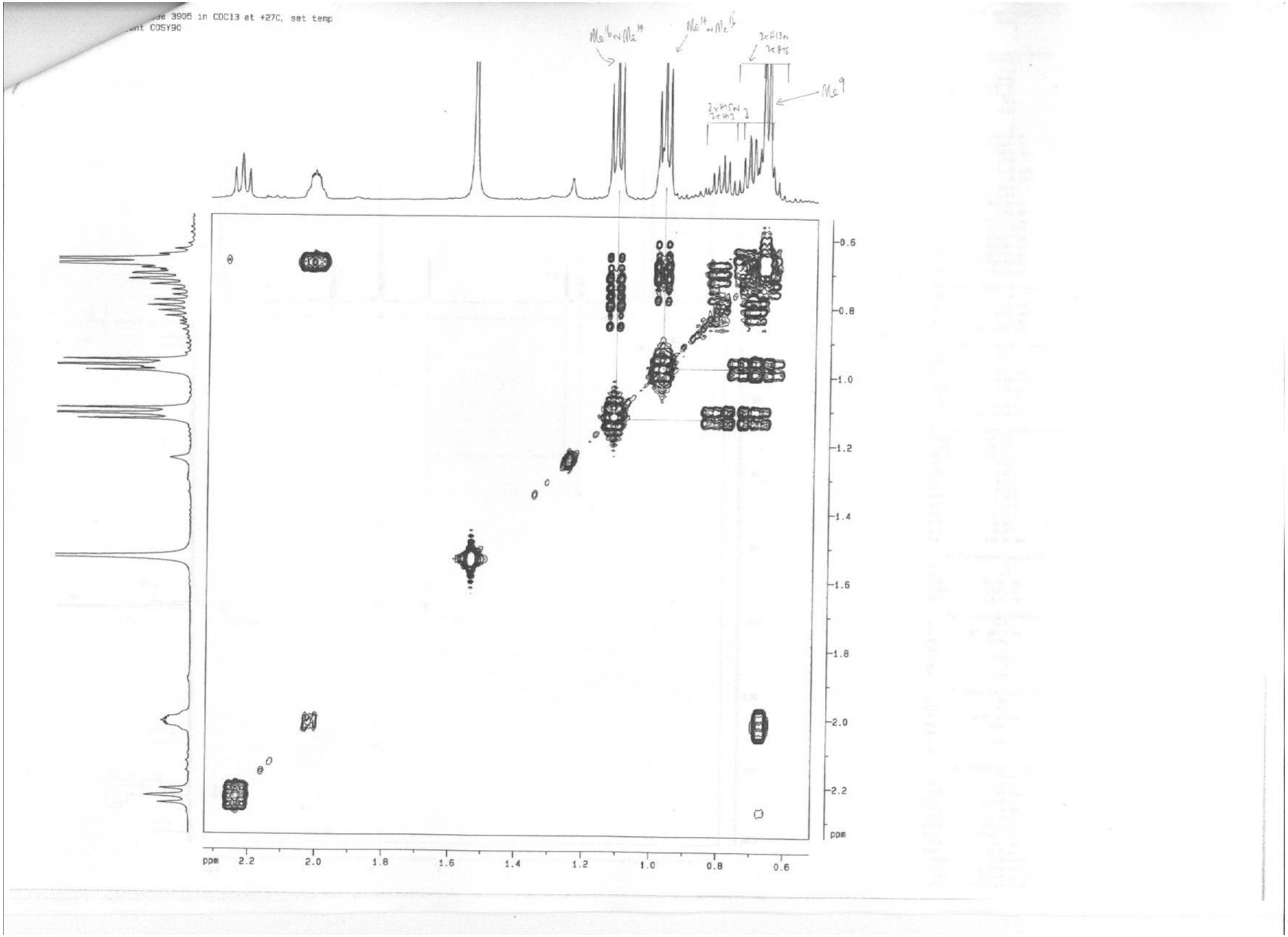
de 3905 in CDCl3 at +27C, set temp
nt COSY90
Me9
ppm
2.2 2.0 1.8 1.6 1.4 1.2 1.0 0.8 0.6
0.6 0.8 1.0 1.2 1.4 1.6 1.8 2.0 2.2

19b

Peter Jervis Barcode 3905 in CDCl3 at +27C, set temp
drx500, Gradient HSGC

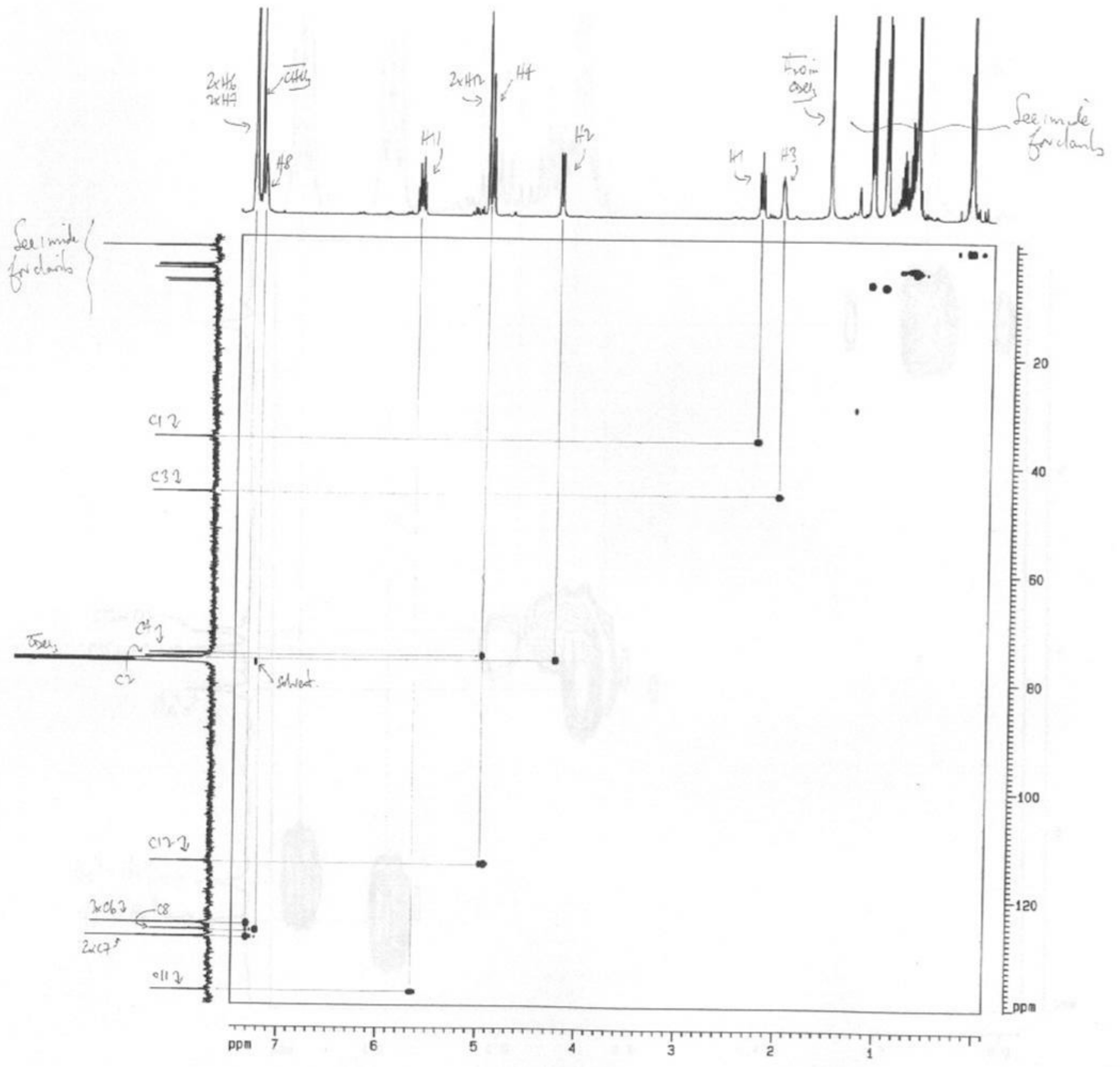

Current Data Parameters

NAME	j126pjid
EXPNO	2
PROCNO	1

F2 - Acquisition Parameters

Date_	20050726
Time	17.12
INSTRUM	drx500
PROBHD	5 mm TBI H/C
PULPROG	invigptp
TD	2048
SOLVENT	CDCl3
NS	8
DS	16
SWH	4310.345 Hz
FIDRES	2.104661 Hz
AQ	0.2376180 sec
RG	32768
DW	116.000 usec
DE	5.50 usec
TE	300.0 K
CNST2	145.0000000
d0	0.00000300 sec
D1	2.00000000 sec
d4	0.00172414 sec
d11	0.03000000 sec
d13	0.00000300 sec
D16	0.00010000 sec
d20	0.00110000 sec
d21	0.00061714 sec
IN0	0.00001140 sec

============ CHANNEL f1 ============

NUC1	1H
P1	10.60 usec
p2	21.20 usec
PL1	1.00 dB
SFO1	500.1318867 MHz

============ CHANNEL f2 ============

CPDPRG2	garp
NUC2	13C
P3	12.00 usec
p4	24.00 usec
PCPD2	76.00 usec
PL2	-1.00 dB
PL12	15.00 dB
SFO2	125.7667893 MHz

============ GRADIENT CHANNEL ========

GPNAM1	SINE.100
GPNAM2	SINE.100
GPNAM3	SINE.100
GPX1	0.00 %
GPX2	0.00 %
GPX3	0.00 %
GPY1	0.00 %
GPY2	0.00 %
GPY3	0.00 %
GPZ1	80.00 %
GPZ2	30.00 %
GPZ3	20.10 %
P16	1000.00 usec

F1 - Acquisition parameters

ND0	4
TD	512
SFO1	125.7668 MHz
FIDRES	42.831688 Hz
SW	174.369 ppm

F2 - Processing parameters

SI	2048
SF	500.1300233 MHz
WDW	QSINE
SSB	2
LB	0.00 Hz
GB	0
PC	1.00

F1 - Processing parameters

SI	1024
MC2	TPPI
SF	125.7577945 MHz
WDW	QSINE
SSB	2
LB	0.00 Hz
GB	0

2D NMR plot parameters

CX2	17.00 cm
CX1	17.00 cm
F2PLO	7.463 ppm
F2LO	3732.40 Hz
F2PHI	-0.104 ppm
F2HI	-51.78 Hz
F1PLO	140.324 ppm
F1LO	17648.84 Hz
F1PHI	-1.531 ppm
F1HI	-192.56 Hz
F2PPMCM	0.44508 ppm/cm
F2HZCM	222.59880 Hz/cm
F1PPMCM	8.34442 ppm/cm
F1HZCM	1049.37634 Hz/cm

19b

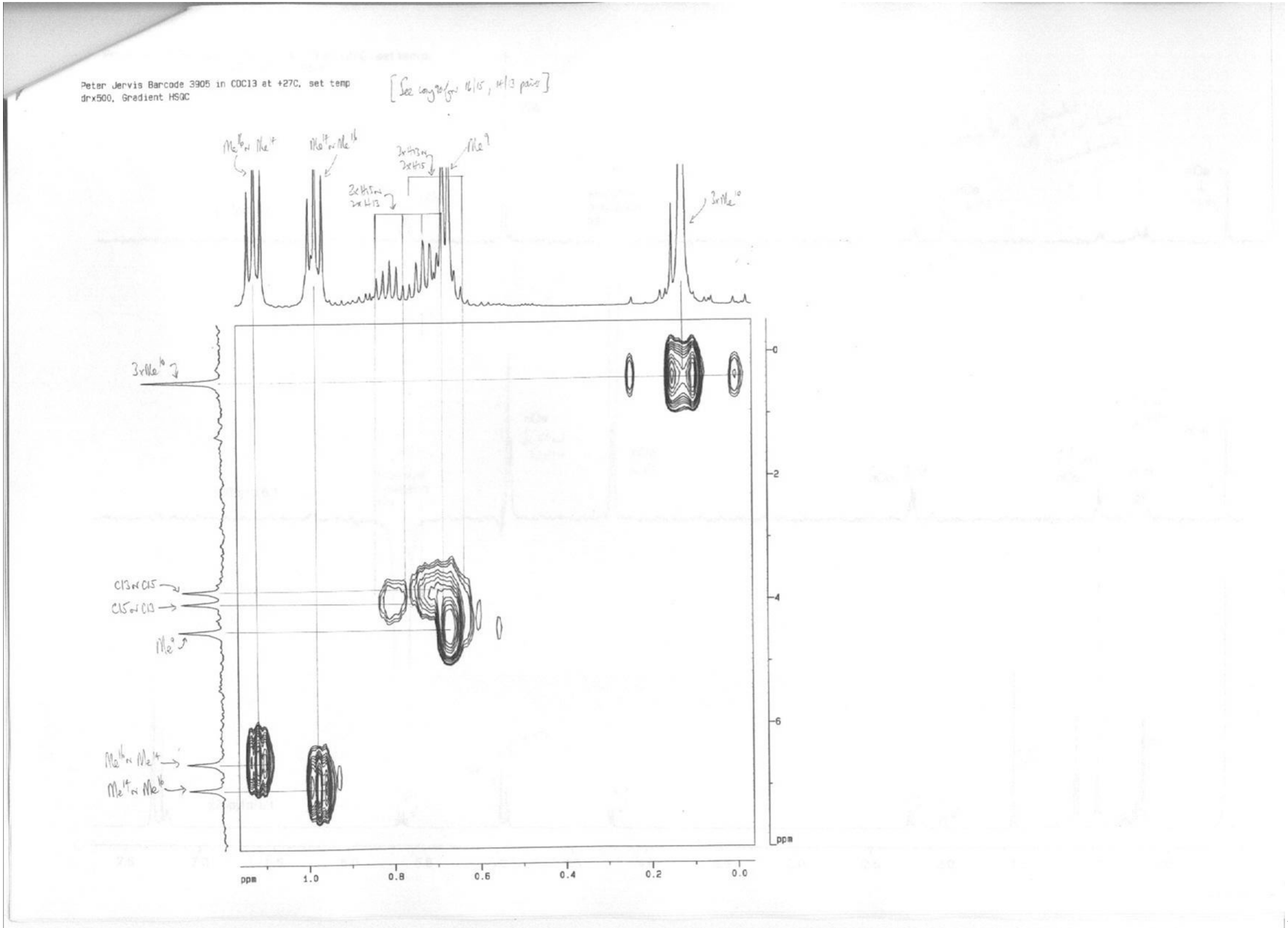

Peter Jervis Barcode 3905 in CDCl3 at +27C, set temp
drx500, Gradient HSQC
ppm
1.0
0.8
0.6
0.4
0.2
0.0
0
2
4
6
ppm

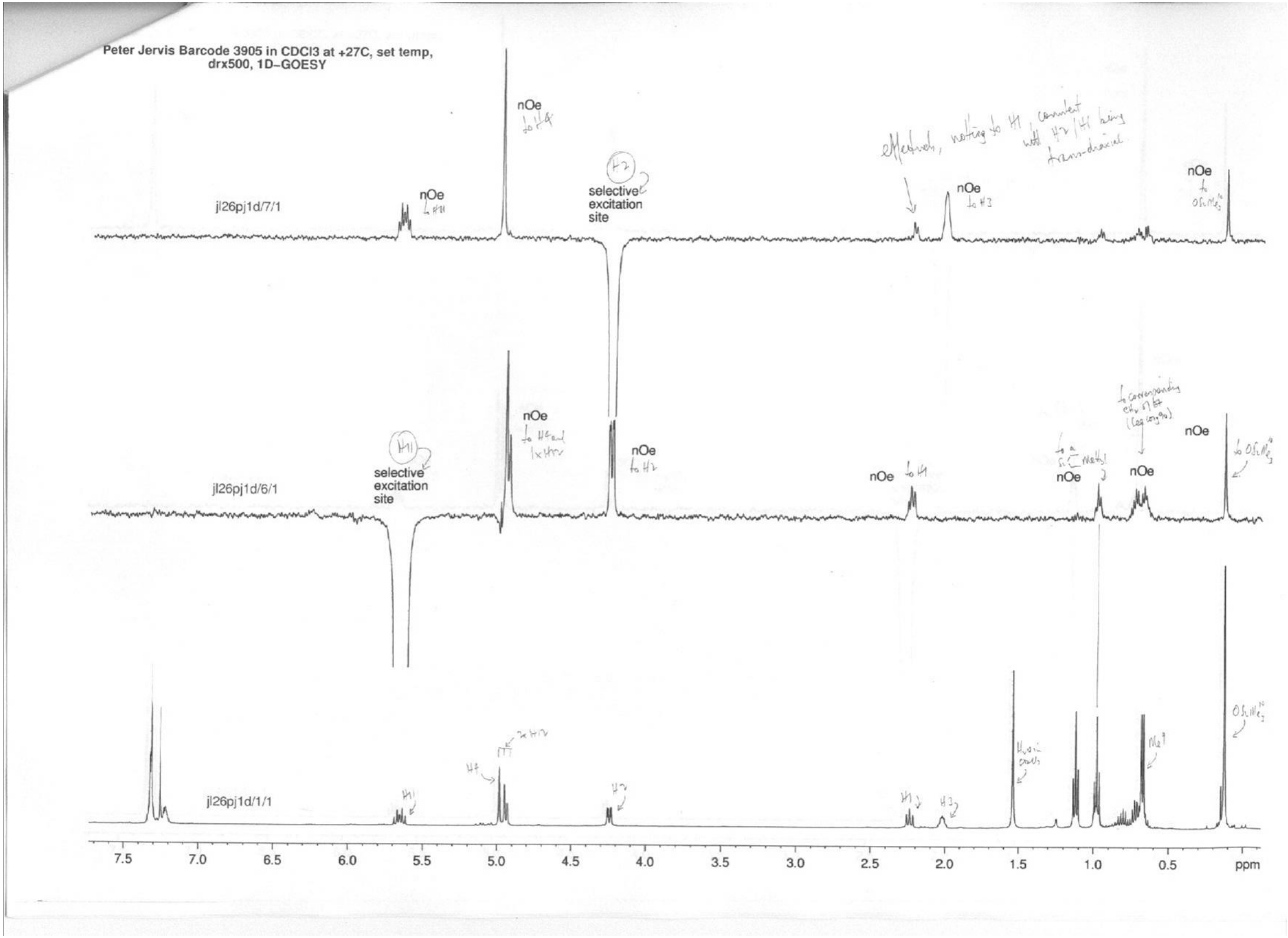

Peter Jervis Barcode 3905 in CDCl3 at +27C, set temp, drx500, 1D-GOESY
jl26pj1d/7/1
nOe
selective excitation site
nOe
nOe
jl26pj1d/6/1
selective excitation site
nOe
nOe
nOe
nOe
nOe
nOe
jl26pj1d/1/1
7.5
7.0
6.5
6.0
5.5
5.0
4.5
4.0
3.5
3.0
2.5
2.0
1.5
1.0
0.5
ppm

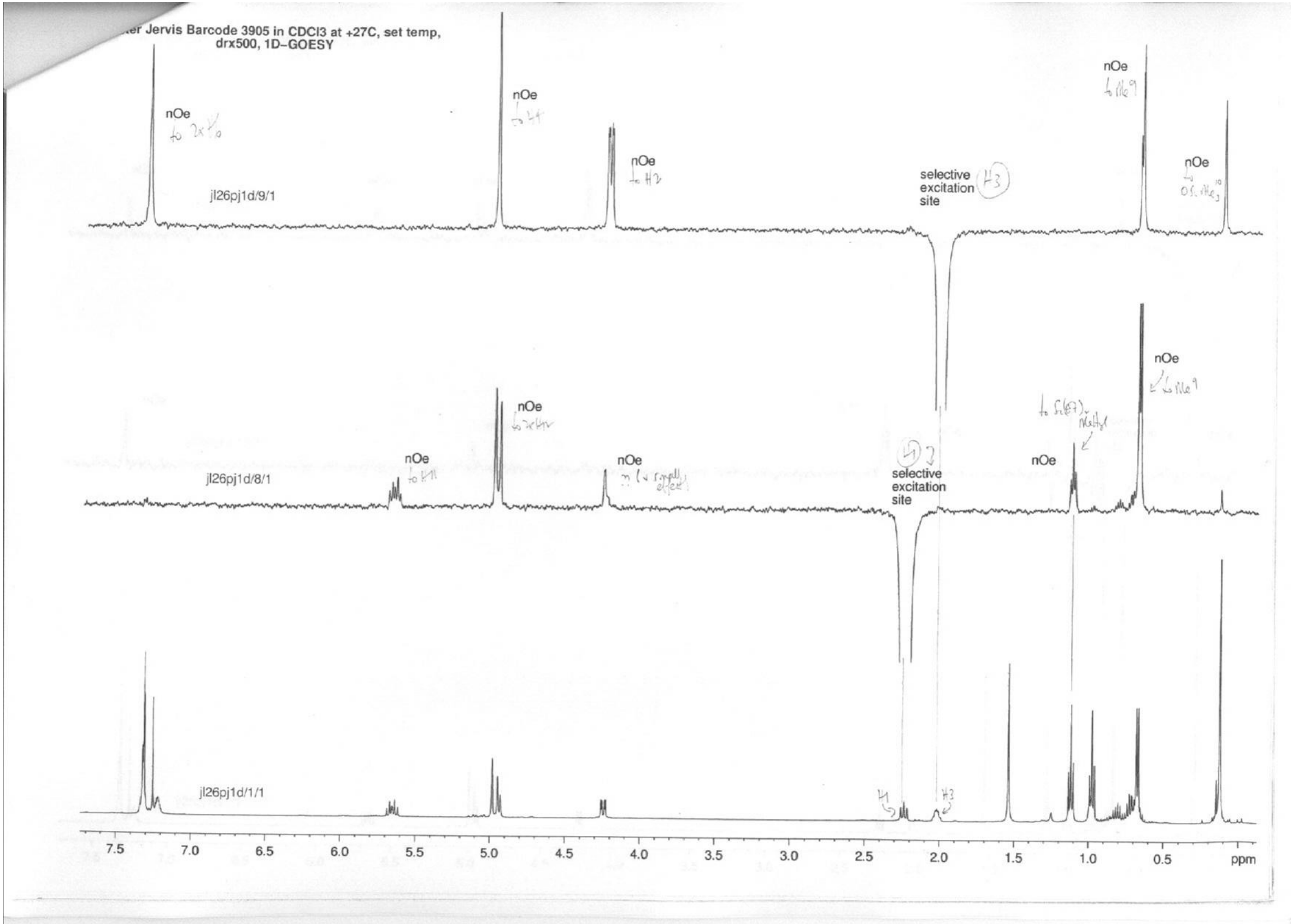
...ker Jervis Barcode 3905 in CDCl3 at +27C, set temp, drx500, 1D-GOESY
nOe
nOe
nOe
nOe
nOe
selective excitation site
jl26pj1d/9/1
nOe
nOe
nOe
nOe
nOe
selective excitation site
jl26pj1d/8/1
jl26pj1d/1/1
7.5
7.0
6.5
6.0
5.5
5.0
4.5
4.0
3.5
3.0
2.5
2.0
1.5
1.0
0.5
ppm

19b

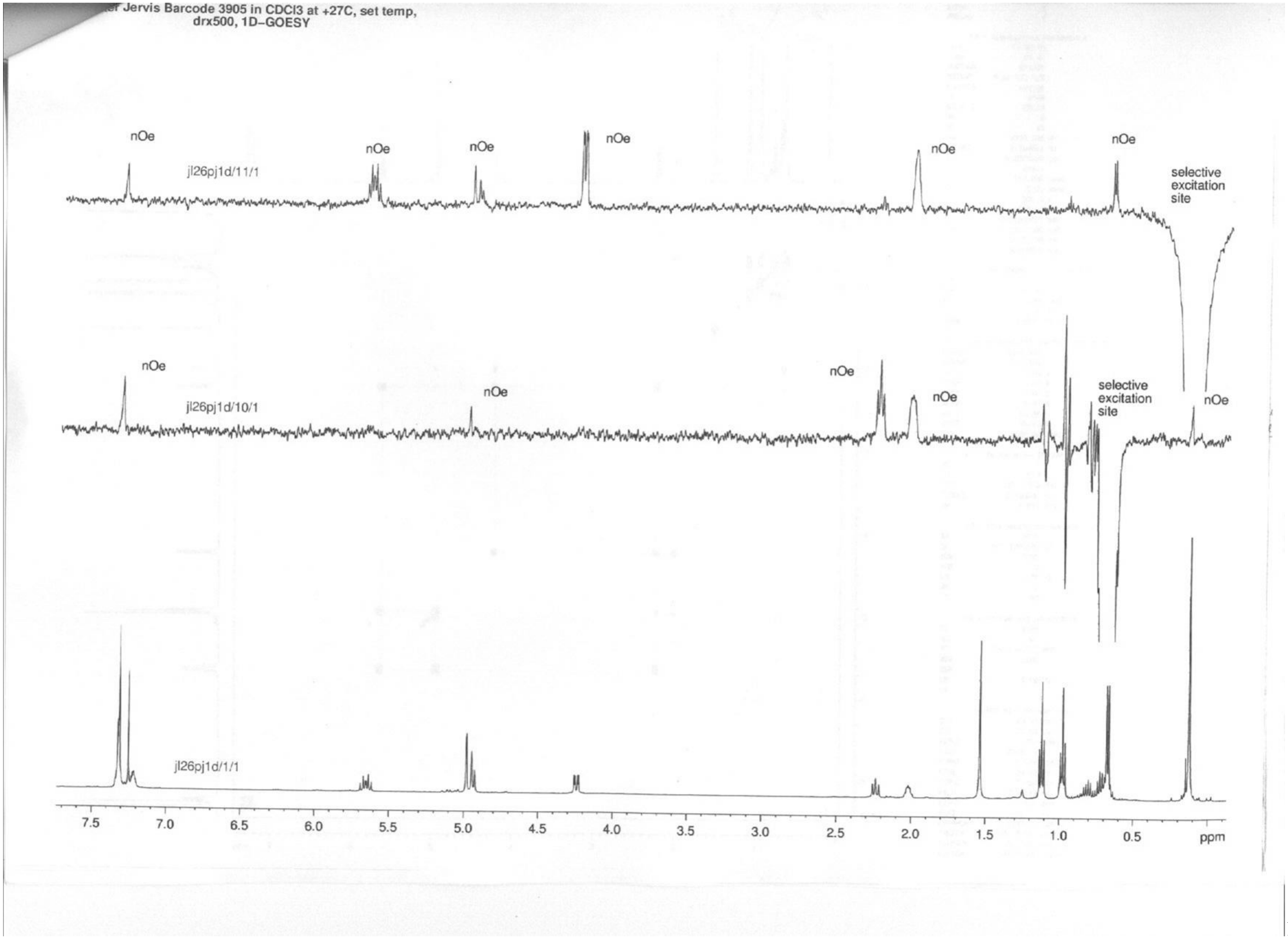
...er Jervis Barcode 3905 in CDCl3 at +27C, set temp,
drx500, 1D-GOESY
nOe
jl26pj1d/11/1
nOe
nOe
nOe
nOe
nOe
selective excitation site
nOe
jl26pj1d/10/1
nOe
nOe
nOe
selective excitation site
nOe
jl26pj1d/1/1
7.5
7.0
6.5
6.0
5.5
5.0
4.5
4.0
3.5
3.0
2.5
2.0
1.5
1.0
0.5
ppm

19b
