# Supplementary material for: Tether-directed synthesis of highly substituted oxasilacycles via an intramolecular allylation employing allylsilanes
Source: Beilstein J Org Chem. 2007 Feb 8;3:6. doi: 10.1186/1860-5397-3-6 (PMC1810303; doi:10.1186/1860-5397-3-6)
Supplement: File 8 — 1H-NMR and 13C-NMR Spectra for the following compounds: 14b, 15b. [file Beilstein_J_Org_Chem-03-06-s008.pdf]

Additional File 8

Tether-directed synthesis of highly substituted oxasilacycles *via* an intramolecular allylation employing allylsilanes

Peter J. Jervis and Liam R. Cox*

email: l.r.cox@bham.ac.uk

^{1}H-NMR and ^{13}C-NMR Spectra for the following compounds:

14b, 15b

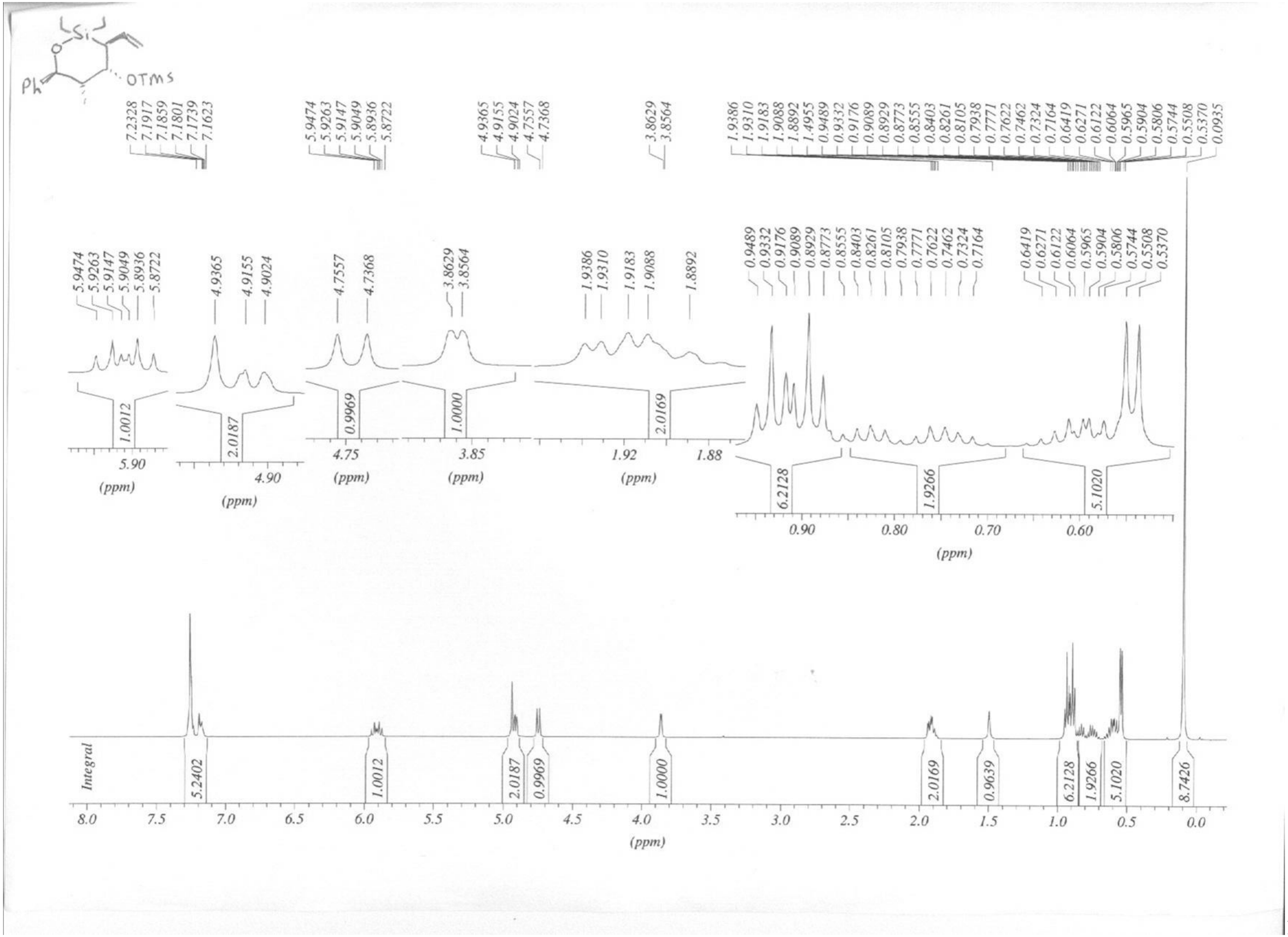
Ph
OTMS
Si
7.2328
7.1917
7.1859
7.1801
7.1739
7.1623
5.9474
5.9263
5.9147
5.9049
5.8936
5.8722
4.9365
4.9155
4.9024
4.7557
4.7368
3.8629
3.8564
1.9386
1.9310
1.9183
1.9088
1.8892
1.4955
0.9489
0.9332
0.9176
0.9089
0.8929
0.8773
0.8555
0.8403
0.8261
0.8105
0.7938
0.7771
0.7622
0.7462
0.7324
0.7164
0.6419
0.6271
0.6122
0.6064
0.5965
0.5904
0.5806
0.5744
0.5508
0.5370
0.0935
1.0012
2.0187
0.9969
1.0000
2.0169
6.2128
1.9266
5.1020
(ppm)
Integral
5.2402
0.9639
8.7426

14b

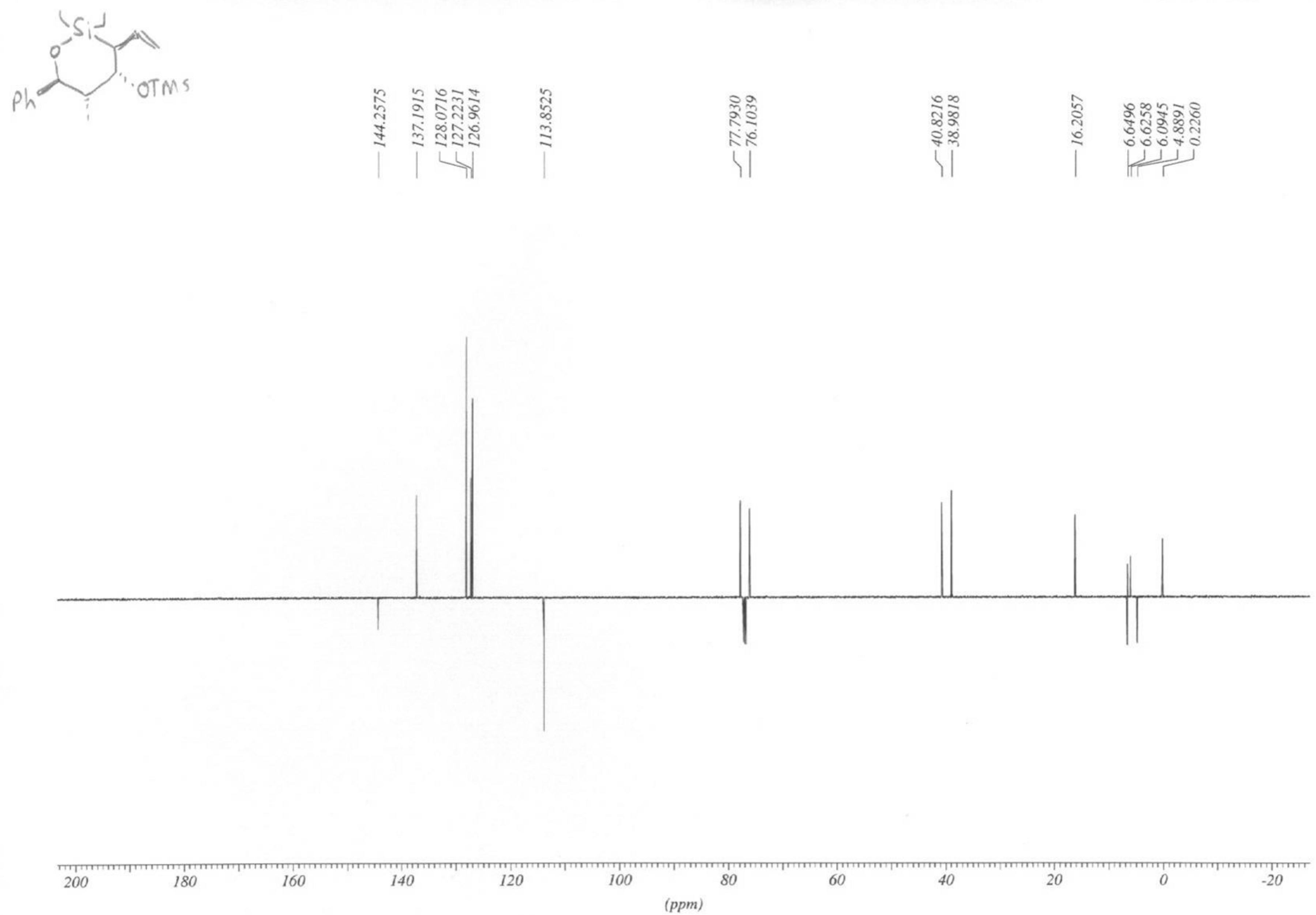
OTMS
Ph
Si
144.2575
137.1915
128.0716
127.2231
126.9614
113.8525
77.7930
76.1039
40.8216
38.9818
16.2057
6.6496
6.6258
6.0945
4.8891
0.2260
200
180
160
140
120
100
80
60
40
20
0
-20
(ppm)

14b

Peter Jervis Sample 1,Barcode 3902 in CDCl3 at +27C, set temp
drx500, Gradient COSY90

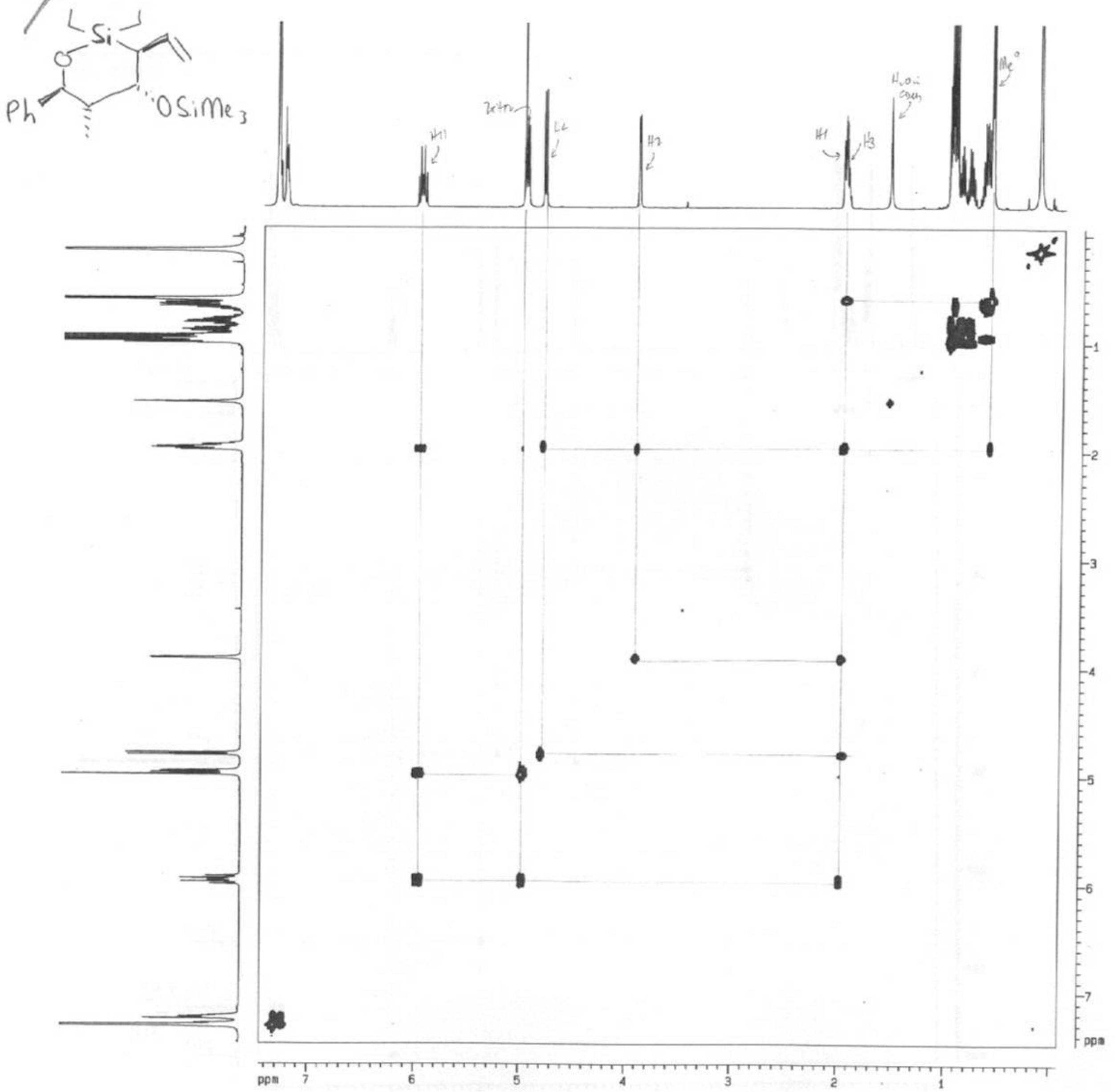

Current Data Parameters

NAME	j105pj1d
EXPNO	4
PROCNO	1

F2 - Acquisition Parameters

Date_	20050705
Time	21.43
INSTRUM	drx500
PROBHD	5 mm TBI H/C
PULPROG	cosygp
TD	2048
SOLVENT	CDCl3
NS	8
DS	16
SWH	4310.345 Hz
FIDRES	2.104661 Hz
AQ	0.2376180 sec
RG	71.8
DW	116.000 usec
DE	5.50 usec
TE	300.0 K
d0	0.00000300 sec
D1	2.00000000 sec
d13	0.00000300 sec
D16	0.00010000 sec
IN0	0.00023200 sec

============ CHANNEL f1 ============

NUC1	1H
P0	10.70 usec
P1	10.70 usec
PL1	1.00 dB
SFO1	500.1318867 MHz

============ GRADIENT CHANNEL ========

GPNAM1	SINE.100
GPNAM2	SINE.100
GPX1	0.00 %
GPX2	0.00 %
GPY1	0.00 %
GPY2	0.00 %
GPZ1	10.00 %
GPZ2	10.00 %
P16	1000.00 usec

F1 - Acquisition parameters

ND0	1
TD	512
SFO1	500.1319 MHz
FIDRES	8.418642 Hz
SW	8.618 ppm

F2 - Processing parameters

SI	2048
SF	500.1300233 MHz
WDW	SINE
SSB	0
LB	0.00 Hz
GB	0
PC	1.00

F1 - Processing parameters

SI	1024
MC2	QF
SF	500.1300233 MHz
WDW	SINE
SSB	0
LB	0.00 Hz
GB	0

2D NMR plot parameters

CX2	20.00 cm
CX1	20.00 cm
F2PLO	7.450 ppm
F2LO	3726.08 Hz
F2PHI	-0.082 ppm
F2HI	-41.26 Hz
F1PLO	7.480 ppm
F1LO	3740.82 Hz
F1PHI	-0.061 ppm
F1HI	-30.73 Hz
F2PPMCM	0.37664 ppm/cm
F2HZCM	188.36713 Hz/cm
F1PPMCM	0.37706 ppm/cm
F1HZCM	188.57759 Hz/cm

14b

Peter Jervis Sample 1.Barcode 3902 in CDCl3 at +27C, set temp
drx500, Gradient HSQC

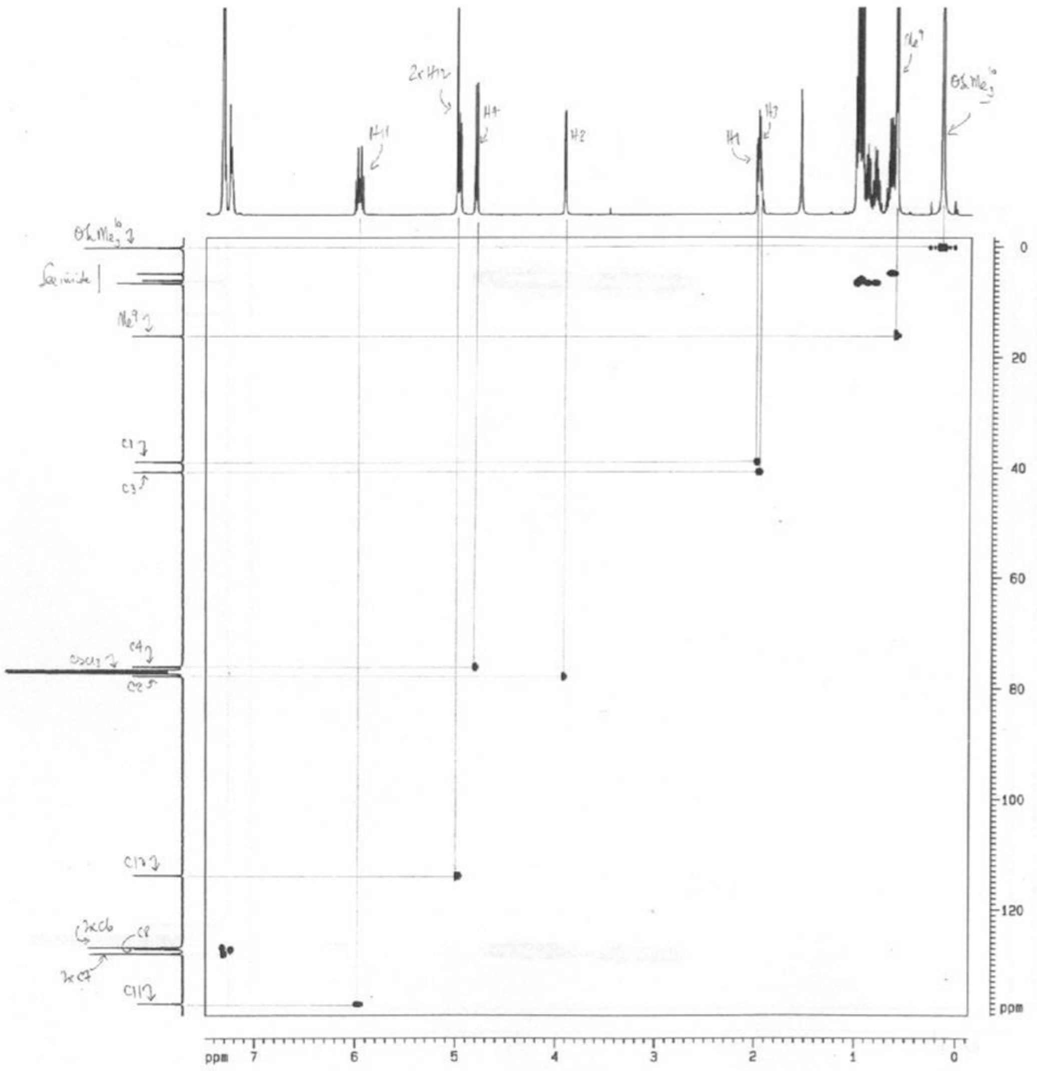

Current Data Parameters

NAME	j105pjid
EXPNO	2
PROCNO	1

F2 - Acquisition Parameters

Date_	20050705
Time	16.26
INSTRUM	drx500
PROBHD	5 mm TBI H/C
PULPROG	invigptp
TD	2048
SOLVENT	CDCl3
NS	8
DS	16
SWH	4310.345 Hz
FIDRES	2.104661 Hz
AQ	0.2376180 sec
RG	32768
DW	116.000 usec
DE	5.50 usec
TE	300.0 K
CNST2	145.0000000
d0	0.00000300 sec
D1	2.00000000 sec
d4	0.00172414 sec
d11	0.03000000 sec
d13	0.00000300 sec
D16	0.00010000 sec
d20	0.00110000 sec
d21	0.00061714 sec
IN0	0.00001140 sec

============ CHANNEL f1 ============

NUC1	1H
P1	10.70 usec
p2	21.40 usec
PL1	1.00 dB
SFO1	500.1318867 MHz

============ CHANNEL f2 ============

CPDPRG2	garp
NUC2	13C
P3	12.00 usec
p4	24.00 usec
PCPD2	76.00 usec
PL2	-1.00 dB
PL12	15.00 dB
SFO2	125.7667893 MHz

============ GRADIENT CHANNEL ============

GPNAM1	SINE.100
GPNAM2	SINE.100
GPNAM3	SINE.100
GPX1	0.00 %
GPX2	0.00 %
GPX3	0.00 %
GPY1	0.00 %
GPY2	0.00 %
GPY3	0.00 %
GPZ1	80.00 %
GPZ2	30.00 %
GPZ3	20.10 %
P16	1000.00 usec

F1 - Acquisition parameters

ND0	4
TD	512
SFO1	125.7668 MHz
FIDRES	42.831688 Hz
SW	174.369 ppm

F2 - Processing parameters

SI	2048
SF	500.1300233 MHz
WDW	QSINE
SSB	2
LB	0.00 Hz
GB	0
PC	1.00

F1 - Processing parameters

SI	1024
MC2	TPPI
SF	125.7577945 MHz
WDW	QSINE
SSB	2
LB	0.00 Hz
GB	0

2D NMR plot parameters

CX2	17.00 cm
CX1	17.00 cm
F2PLO	7.488 ppm
F2LO	3745.03 Hz
F2PHI	-0.129 ppm
F2HI	-64.41 Hz
F1PLO	139.302 ppm
F1LO	17518.34 Hz
F1PHI	-1.701 ppm
F1HI	-213.88 Hz
F2PPMCM	0.44805 ppm/cm
F2HZCM	224.08446 Hz/cm
F1PPMCM	8.29434 ppm/cm
F1HZCM	1043.07764 Hz/cm

Peter Jervis Sample 1.Barcode 3902 in CDC13 at +27C, set temp
drx500, Gradient HMBC

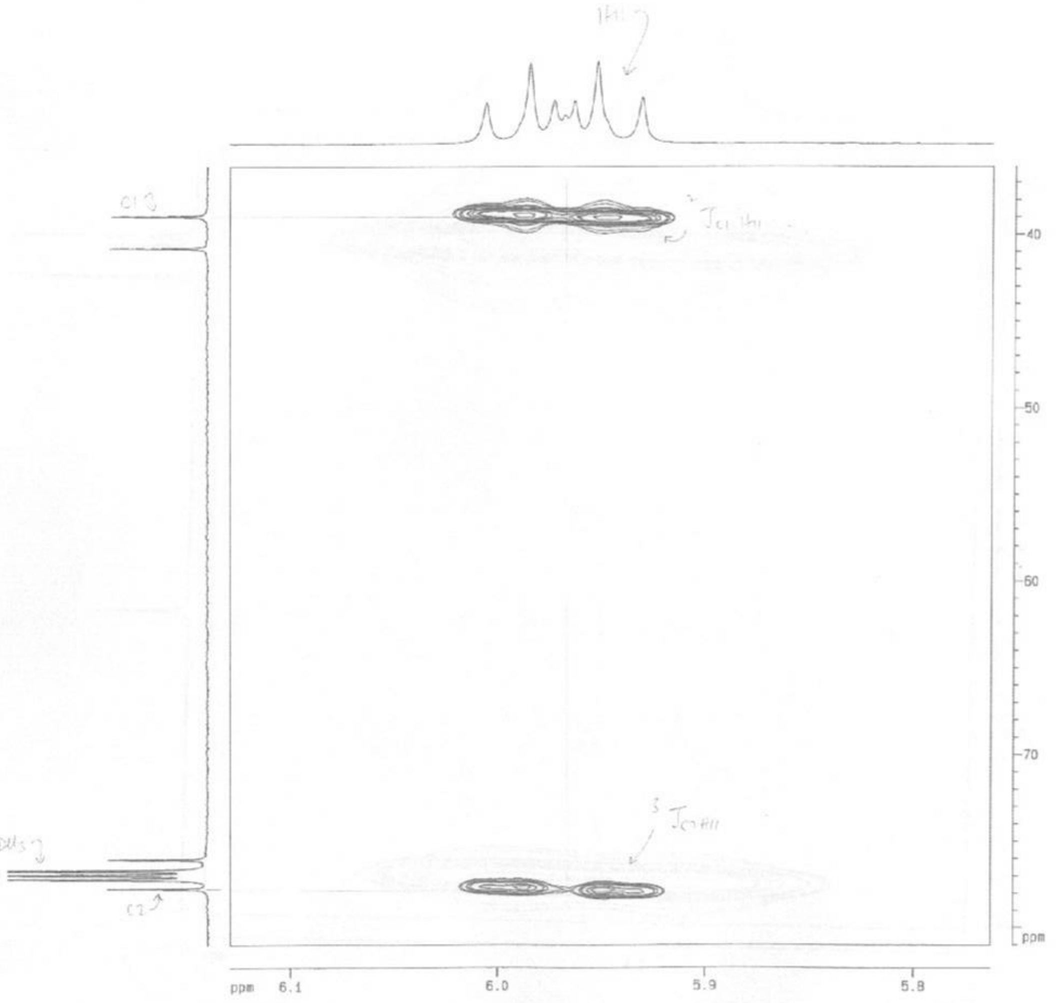

Current Data Parameters

NAME	j105pj1d
EXPNO	3
PROCNO	1

F2 - Acquisition Parameters

Date_	20050705
Time	19.01
INSTRUM	drx500
PROBHD	5 mm TBI H/C
PULPROG	inv4gp1plrnd
TD	2048
SOLVENT	CDC13
NS	8
DS	16
SWH	4310.345 Hz
FIDRES	2.104661 Hz
AQ	0.2376180 sec
RG	32768
DW	116.000 usec
DE	6.50 usec
TE	300.0 K
CNST2	160.0000000
d0	0.00000300 sec
D1	2.00000000 sec
d2	0.00312500 sec
D6	0.10000000 sec
d13	0.00000300 sec
D16	0.00010000 sec
IN0	0.00002260 sec

======== CHANNEL f1 ========

NUC1	1H
P1	10.70 usec
p2	21.40 usec
PL1	1.00 dB
SFO1	500.1318867 MHz

======== CHANNEL f2 ========

NUC2	13C
P3	12.00 usec
PL2	-1.00 dB
SFO2	125.7667893 MHz

======== GRADIENT CHANNEL ========

GPNAM1	SINE.100
GPNAM2	SINE.100
GPNAM3	SINE.100
GPX1	0.00 %
GPX2	0.00 %
GPX3	0.00 %
GPY1	0.00 %
GPY2	0.00 %
GPY3	0.00 %
GPZ1	50.00 %
GPZ2	30.00 %
GPZ3	40.10 %
P16	1000.00 usec

F1 - Acquisition parameters

ND0	2
TD	512
SFO1	125.7668 MHz
FIDRES	42.831688 Hz
SW	174.369 ppm

F2 - Processing parameters

SI	2048
SF	500.1300233 MHz
WDW	QSINE
SSB	2
LB	0.00 Hz
GB	0
PC	0.20

F1 - Processing parameters

SI	1024
MC2	QF
SF	125.7577945 MHz
WDW	QSINE
SSB	2
LB	0.00 Hz
GB	0

2D NMR plot parameters

CX2	17.00 cm
CX1	17.00 cm
F2PLO	6.129 ppm
F2LO	3065.22 Hz
F2PHI	5.763 ppm
F2HI	2882.12 Hz
F1PLO	81.062 ppm
F1LO	10194.12 Hz
F1PHI	36.104 ppm
F1HI	4540.34 Hz
F2PPMCM	0.02154 ppm/cm
F2HZCM	10.77091 Hz/cm
F1PPMCM	2.64457 ppm/cm
F1HZCM	332.57547 Hz/cm

14b

Peter Jervis Sample 1, Barcode 3902 in CDCl3 at +27C, set temp
drx500, Gradient HMBC

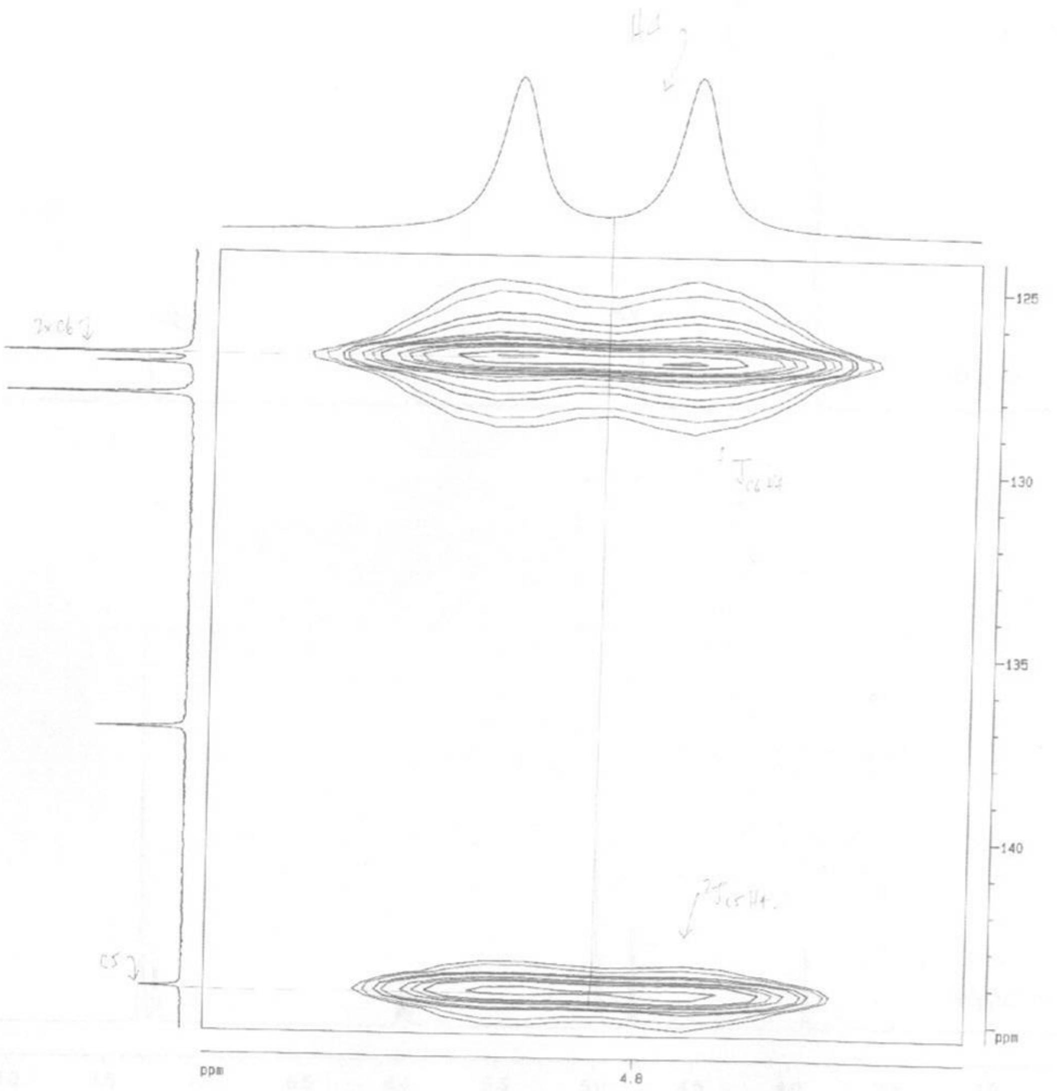

**Current Data Parameters**		
NAME	j106pj1d	
EXPNO	3	
PROCNO	1	
**F2 - Acquisition Parameters**		
Date_	20050705	
Time	19.01	
INSTRUM	drx500	
PROBHD	5 mm TBI H/C	
PULPROG	inv4gp3lplrnd	
TD	2048	
SOLVENT	CDCl3	
NS	8	
DS	16	
SWH	4310.345	Hz
FIDRES	2.104661	Hz
AQ	0.2376180	sec
RG	32768	
DW	116.000	usec
DE	5.80	usec
TE	300.0	K
CNST2	160.0000000	
d0	0.00000300	sec
D1	2.00000000	sec
d2	0.00312500	sec
D6	0.10000000	sec
d13	0.00000300	sec
D16	0.00010000	sec
IN0	0.00002280	sec
**======== CHANNEL f1 ========**		
NUC1	1H	
P1	10.70	usec
p2	21.40	usec
PL1	1.00	dB
SFO1	500.1318867	MHz
**======== CHANNEL f2 ========**		
NUC2	13C	
P3	12.00	usec
PL2	-1.00	dB
SFO2	125.7667893	MHz
**======== GRADIENT CHANNEL ========**		
GPNAM1	SINE.100	
GPNAM2	SINE.100	
GPNAM3	SINE.100	
GPX1	0.00	%
GPX2	0.00	%
GPX3	0.00	%
GPY1	0.00	%
GPY2	0.00	%
GPY3	0.00	%
GPZ1	50.00	%
GPZ2	30.00	%
GPZ3	40.10	%
P16	1000.00	usec
**F1 - Acquisition parameters**		
ND0	2	
TD	512	
SFO1	125.7668	MHz
FIDRES	42.831688	Hz
SW	174.369	ppm
**F2 - Processing parameters**		
SI	2048	
SF	500.1300233	MHz
WDW	QSINE	
SSB	2	
LB	0.00	Hz
GB	0	
PC	0.20	
**F1 - Processing parameters**		
SI	1024	
MC2	QF	
SF	125.7577945	MHz
WDW	QSINE	
SSB	2	
LB	0.00	Hz
GB	0	
**2D NMR plot parameters**		
CX2	17.00	cm
CX1	17.00	cm
F2PLO	4.845	ppm
F2LO	2423.30	Hz
F2PHI	4.765	ppm
F2HI	2383.31	Hz
F1PLO	145.433	ppm
F1LO	18289.31	Hz
F1PHI	124.146	ppm
F1HI	15612.33	Hz
F2PPMCM	0.00470	ppm/cm
F2HZCM	2.35227	Hz/cm
F1PPMCM	1.25216	ppm/cm
F1HZCM	157.46945	Hz/cm

14b

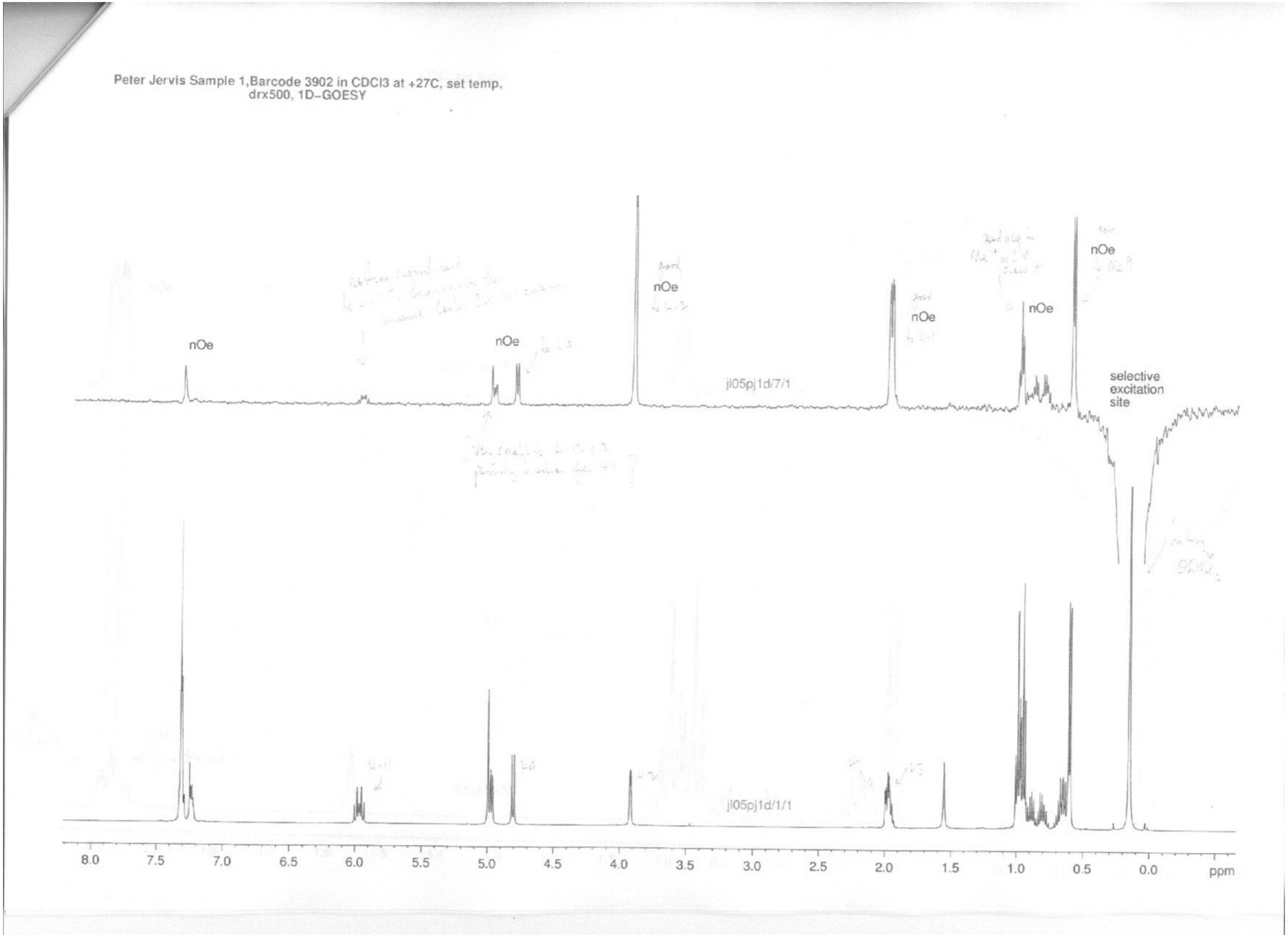
Peter Jervis Sample 1,Barcode 3902 in CDCl3 at +27C, set temp,
drx500, 1D-GOESY
nOe
nOe
nOe
nOe
nOe
nOe
selective
excitation
site
jl05pj1d/7/1
jl05pj1d/1/1
8.0 7.5 7.0 6.5 6.0 5.5 5.0 4.5 4.0 3.5 3.0 2.5 2.0 1.5 1.0 0.5 0.0 ppm

14b

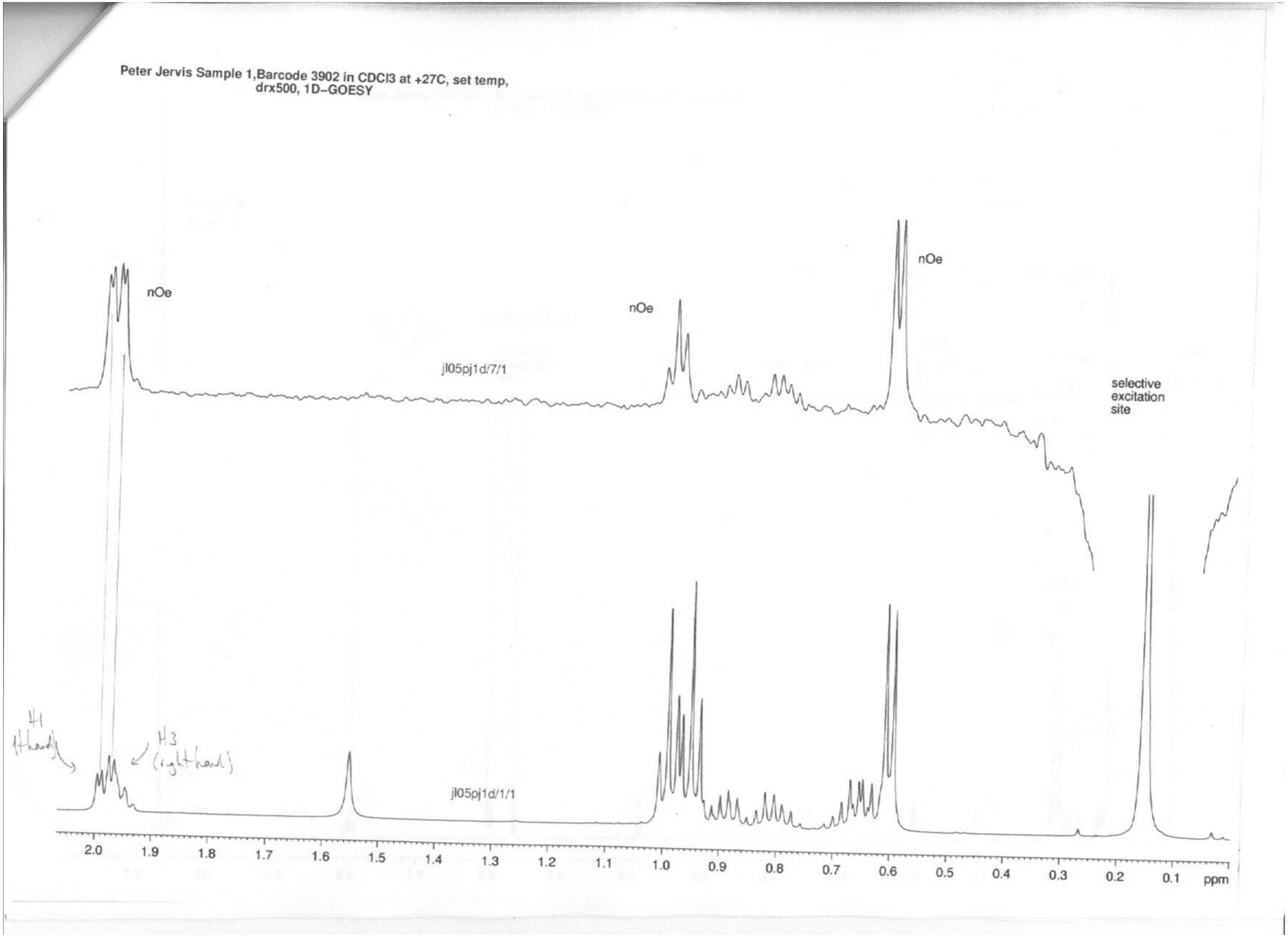
Peter Jervis Sample 1,Barcode 3902 in CDCl3 at +27C, set temp,
drx500, 1D-GOESY
nOe
nOe
nOe
jl05pj1d/7/1
selective
excitation
site
H1
H3
jl05pj1d/1/1
2.0 1.9 1.8 1.7 1.6 1.5 1.4 1.3 1.2 1.1 1.0 0.9 0.8 0.7 0.6 0.5 0.4 0.3 0.2 0.1 ppm

14b

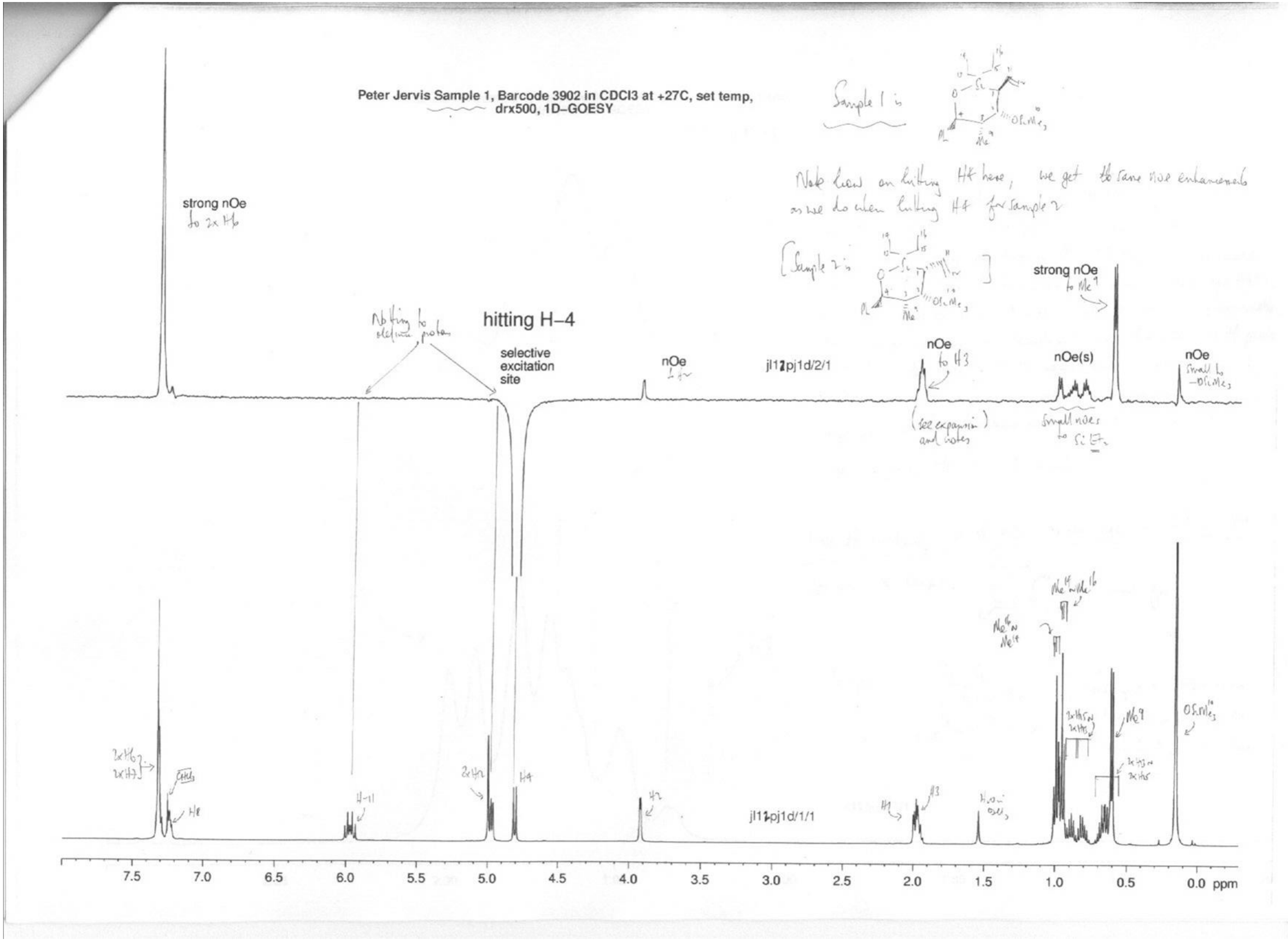
Peter Jervis Sample 1, Barcode 3902 in CDCl3 at +27C, set temp,
drx500, 1D-GOESY
Sample 1 is
strong nOe
to 2x H6
hitting H-4
selective
excitation
site
nOe
jl11pj1d/2/1
nOe
to H3
(see expansion
and notes)
nOe(s)
strong nOe
to Me9
nOe
jl11pj1d/1/1
H4
H-11
Me9
7.5
7.0
6.5
6.0
5.5
5.0
4.5
4.0
3.5
3.0
2.5
2.0
1.5
1.0
0.5
0.0 ppm

14b

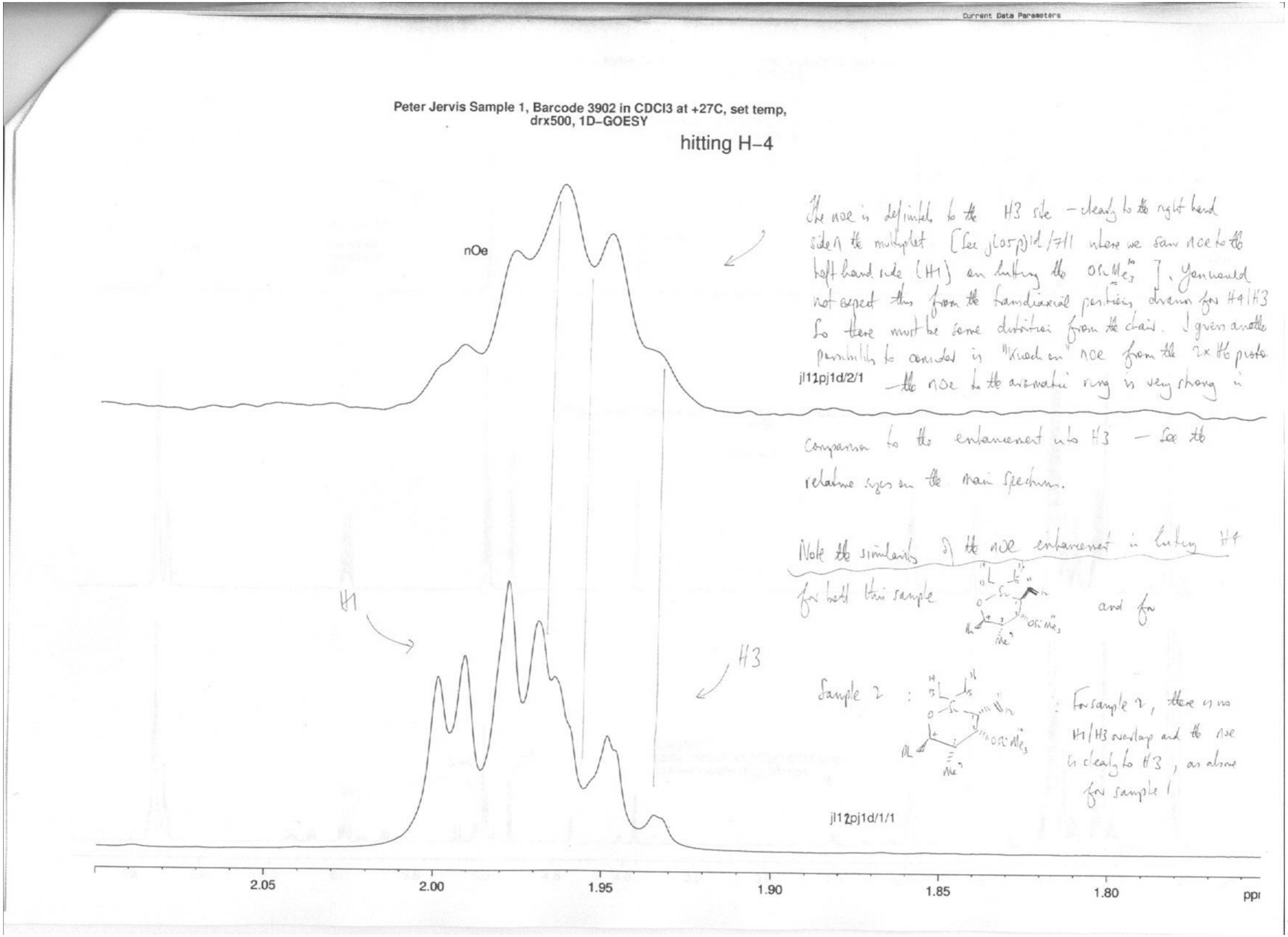
Current Data Parameters
Peter Jervis Sample 1, Barcode 3902 in CDCl3 at +27C, set temp,
drx500, 1D-GOESY
hitting H-4
nOe
jl11pj1d/2/1
H1
H3
jl12pj1d/1/1
2.05
2.00
1.95
1.90
1.85
1.80
ppm
The nOe is definitely to the H3 site – clearly to the right hand side of the multiplet [See jl05pj1d/7/1 where we saw nOe to the left hand side (H1) on hitting the OSiMe3]. You would not expect this from the trans-diaxial positions drawn for H4/H3 so there must be some distortion from the chair. A given another possibility to consider is "knock on" nOe from the 2x H6 protons – the nOe to the aromatic ring is very strong in comparison to the enhancement into H3 – see the relative sizes on the main spectrum.
Note the similarity of the nOe enhancement in hitting H4
for both this sample
and for
Sample 2 :
: For sample 2, there is no H1/H3 overlap and the nOe is clearly to H3, as above for sample 1
OSiMe3
Me

14b

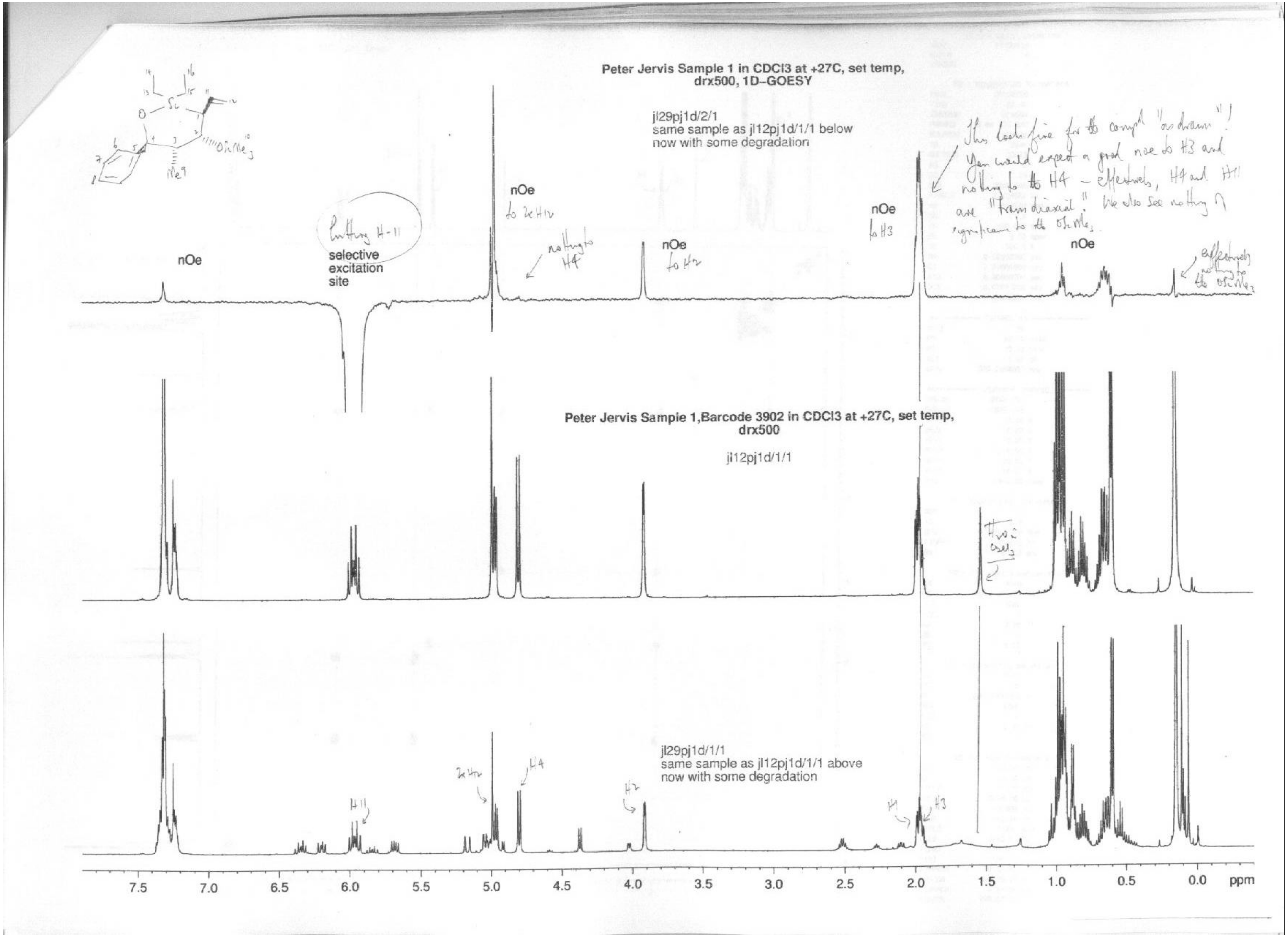

Peter Jervis Sample 1 in CDCl3 at +27C, set temp, drx500, 1D-GOESY
jl29pj1d/2/1
same sample as jl12pj1d/1/1 below
now with some degradation
nOe
selective
excitation
site
nOe
nOe
nOe
nOe
Peter Jervis Sample 1,Barcode 3902 in CDCl3 at +27C, set temp, drx500
jl12pj1d/1/1
jl29pj1d/1/1
same sample as jl12pj1d/1/1 above
now with some degradation
7.5
7.0
6.5
6.0
5.5
5.0
4.5
4.0
3.5
3.0
2.5
2.0
1.5
1.0
0.5
0.0
ppm

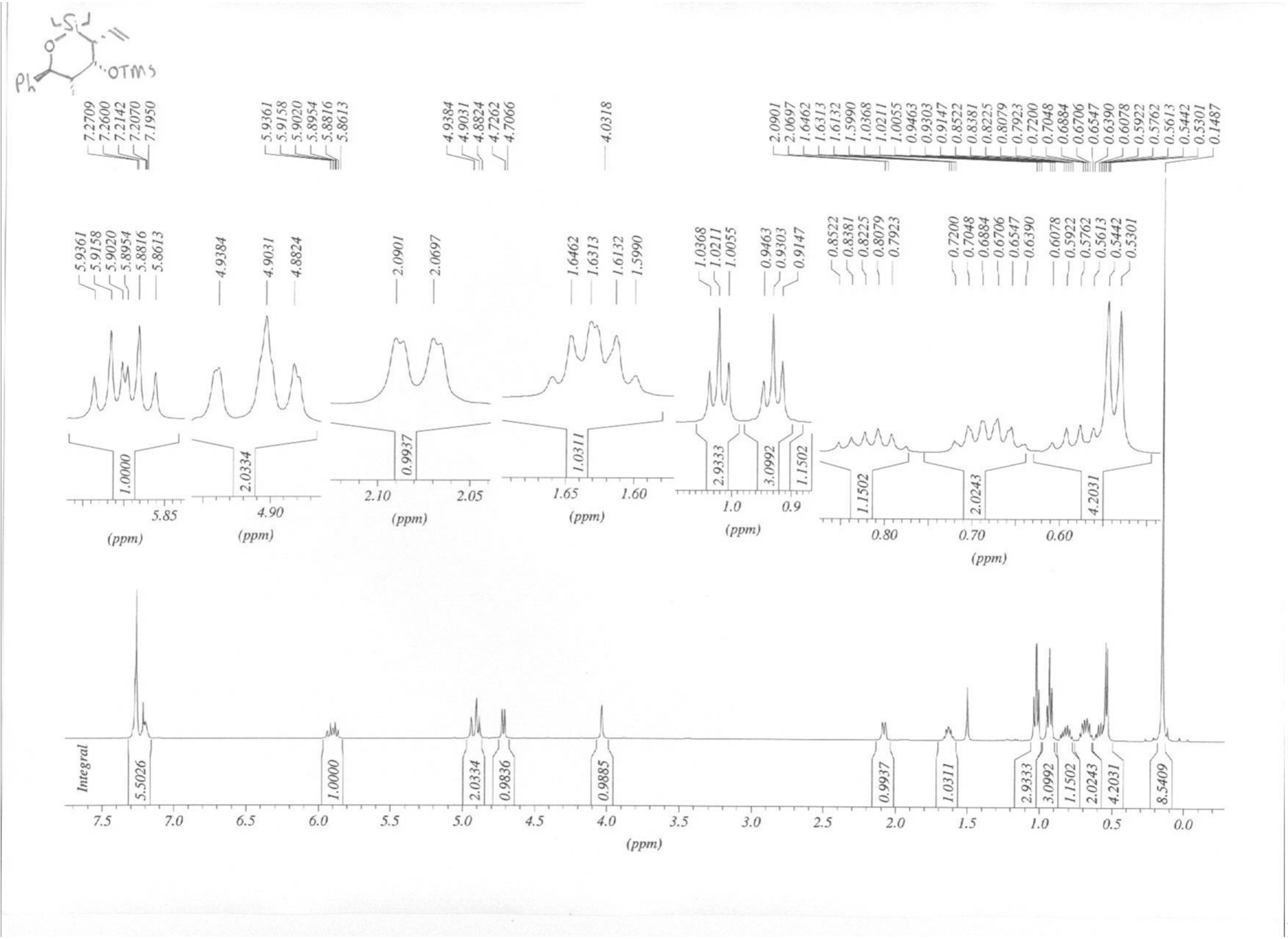

Ph
OTMS
Si
O
7.2709
7.2600
7.2142
7.2070
7.1950
5.9361
5.9158
5.9020
5.8954
5.8816
5.8613
4.9384
4.9031
4.8824
4.7262
4.7066
4.0318
2.0901
2.0697
1.6462
1.6313
1.6132
1.5990
1.0368
1.0211
1.0055
0.9463
0.9303
0.9147
0.8522
0.8381
0.8225
0.8079
0.7923
0.7200
0.7048
0.6884
0.6706
0.6547
0.6390
0.6078
0.5922
0.5762
0.5613
0.5442
0.5301
0.1487
Integral
5.5026
1.0000
2.0334
0.9836
0.9885
0.9937
1.0311
2.9333
3.0992
1.1502
2.0243
4.2031
8.5409
(ppm)

15b

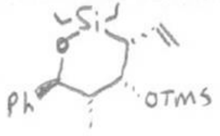
Ph
OTMS
Si
O

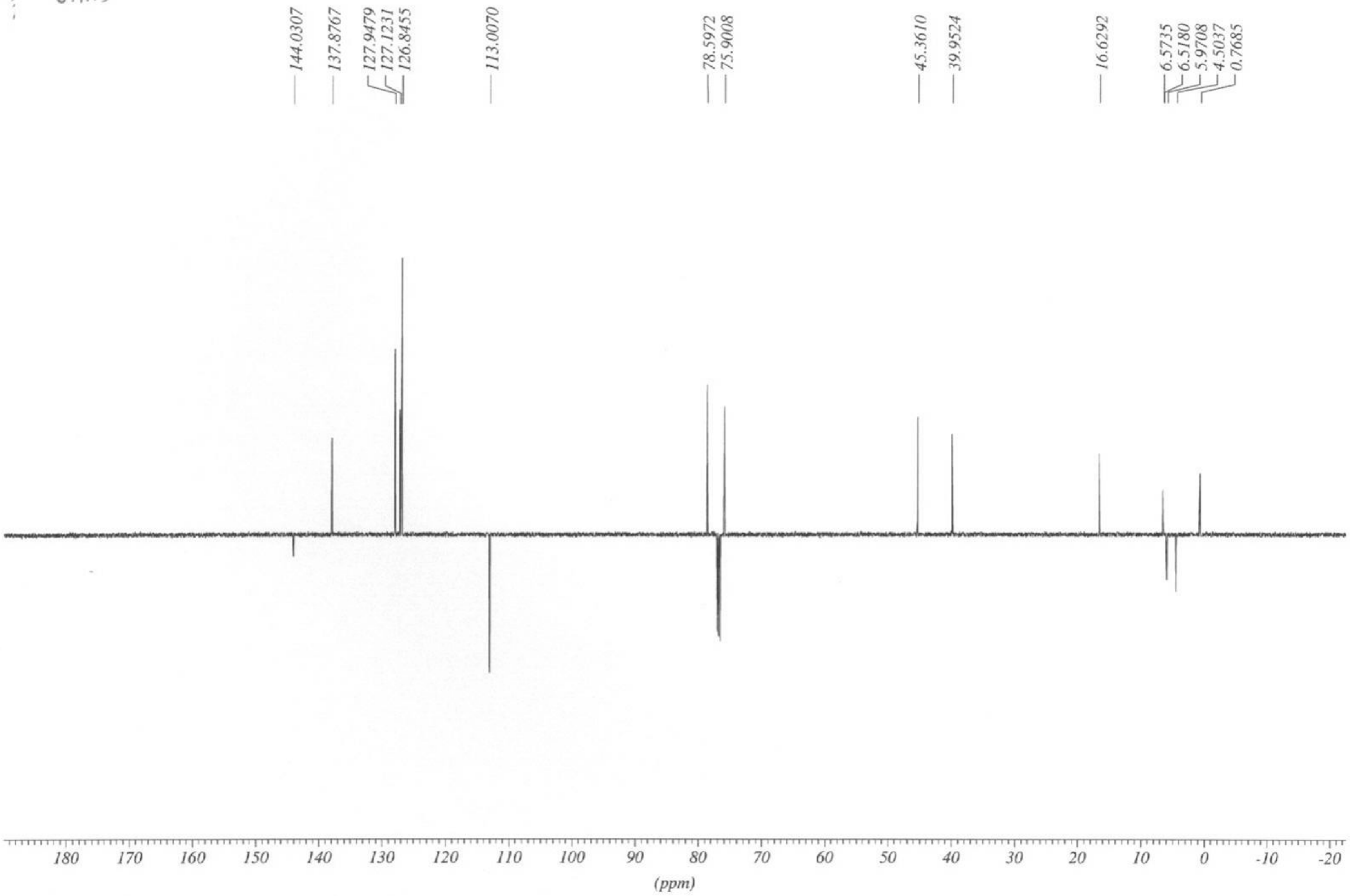
144.0307
137.8767
127.9479
127.1231
126.8455
113.0070
78.5972
75.9008
45.3610
39.9524
16.6292
6.5735
6.5180
5.9708
4.5037
0.7685
180
170
160
150
140
130
120
110
100
90
80
70
60
50
40
30
20
10
0
-10
-20
(ppm)

15b

Peter Jervis Sample 2, Barcode 3901 in CDCl3 at +27C, set temp
drx500, Gradient HSQC

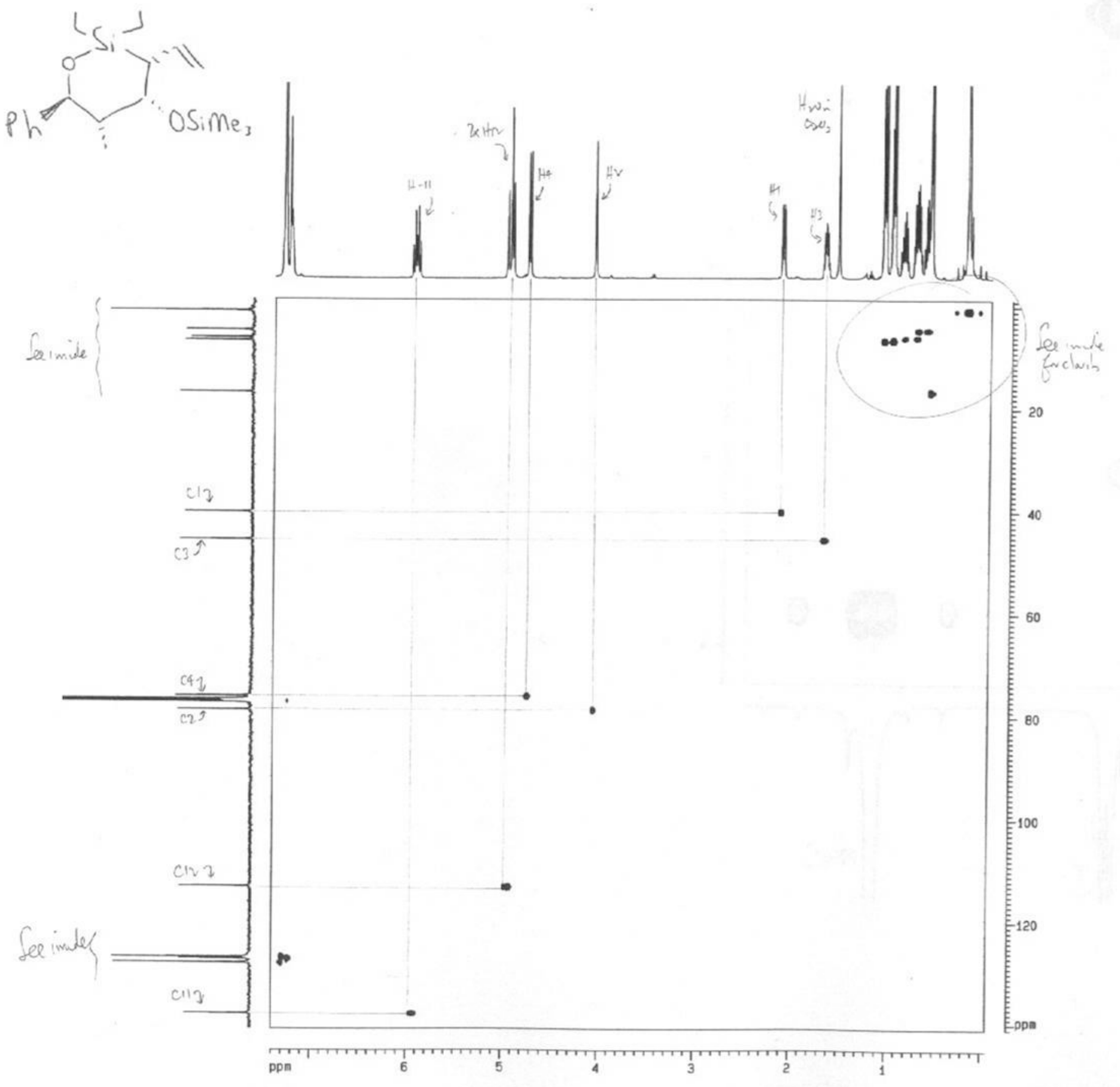

Current Data Parameters

NAME	j106pj1d
EXPNO	2
PROCNO	1

F2 - Acquisition Parameters

Date_	20050706
Time	17.09
INSTRUM	drx500
PROBHD	5 mm TBI H/C
PULPROG	invigptp
TD	2048
SOLVENT	CDCl3
NS	8
DS	16
SWH	4310.345 Hz
FIDRES	2.104661 Hz
AQ	0.2376180 sec
RG	32768
DW	116.000 usec
DE	5.50 usec
TE	300.0 K
CNST2	145.0000000
d0	0.00000300 sec
D1	2.00000000 sec
d4	0.00172414 sec
d11	0.03000000 sec
d13	0.00000300 sec
D16	0.00010000 sec
d20	0.00110000 sec
d21	0.00061714 sec
IN0	0.00001140 sec

CHANNEL f1

NUC1	1H
P1	10.70 usec
p2	21.40 usec
PL1	1.00 dB
SFO1	500.1318867 MHz

CHANNEL f2

CPDPRG2	garp
NUC2	13C
P3	12.00 usec
p4	24.00 usec
PCPD2	78.00 usec
PL2	-1.00 dB
PL12	15.00 dB
SFO2	125.7667893 MHz

GRADIENT CHANNEL

GPNAM1	SINE.100
GPNAM2	SINE.100
GPNAM3	SINE.100
GPX1	0.00 %
GPX2	0.00 %
GPX3	0.00 %
GPY1	0.00 %
GPY2	0.00 %
GPY3	0.00 %
GPZ1	80.00 %
GPZ2	30.00 %
GPZ3	20.10 %
P16	1000.00 usec

F1 - Acquisition parameters

ND0	4
TD	512
SFO1	125.7668 MHz
FIDRES	42.831688 Hz
SW	174.369 ppm

F2 - Processing parameters

SI	2048
SF	500.1300233 MHz
WDW	QSINE
SSB	2
LB	0.00 Hz
GB	0
PC	1.00

F1 - Processing parameters

SI	1024
MC2	TPPI
SF	125.7577945 MHz
WDW	QSINE
SSB	2
LB	0.00 Hz
GB	0

2D NMR plot parameters

CX2	17.00 cm
CX1	17.00 cm
F2PLO	7.404 ppm
F2LO	3702.93 Hz
F2PHI	-0.057 ppm
F2HI	-28.63 Hz
F1PLO	141.005 ppm
F1LO	17732.50 Hz
F1PHI	-1.361 ppm
F1HI	-171.14 Hz
F2PPMCM	0.43888 ppm/cm
F2HZCM	219.50372 Hz/cm
F1PPMCM	8.37448 ppm/cm
F1HZCM	1053.15564 Hz/cm

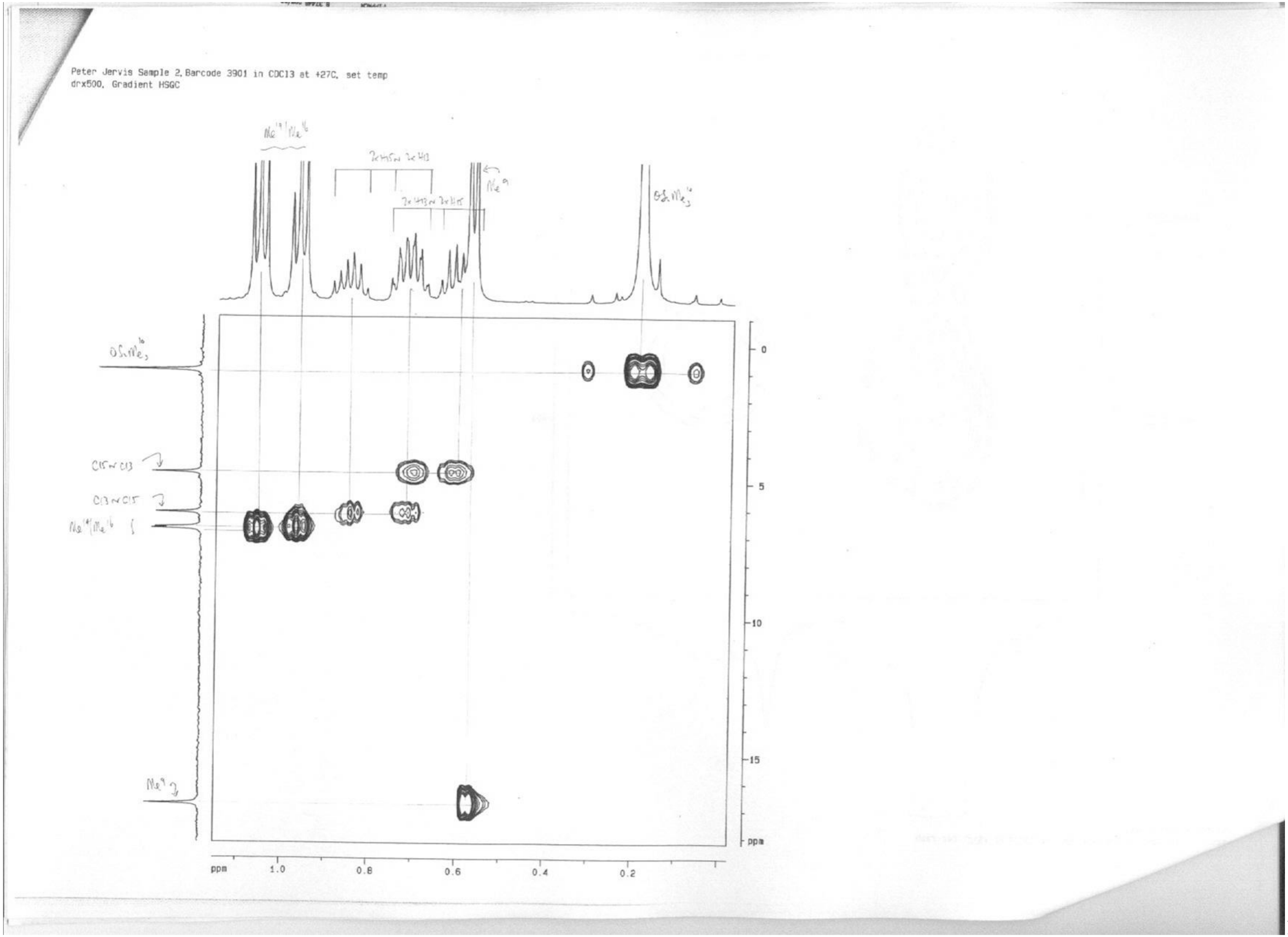
Peter Jervis Sample 2, Barcode 3901 in CDCl3 at +27C, set temp
drx500, Gradient HSGC
ppm
1.0
0.8
0.6
0.4
0.2
0
5
10
15
ppm

15b

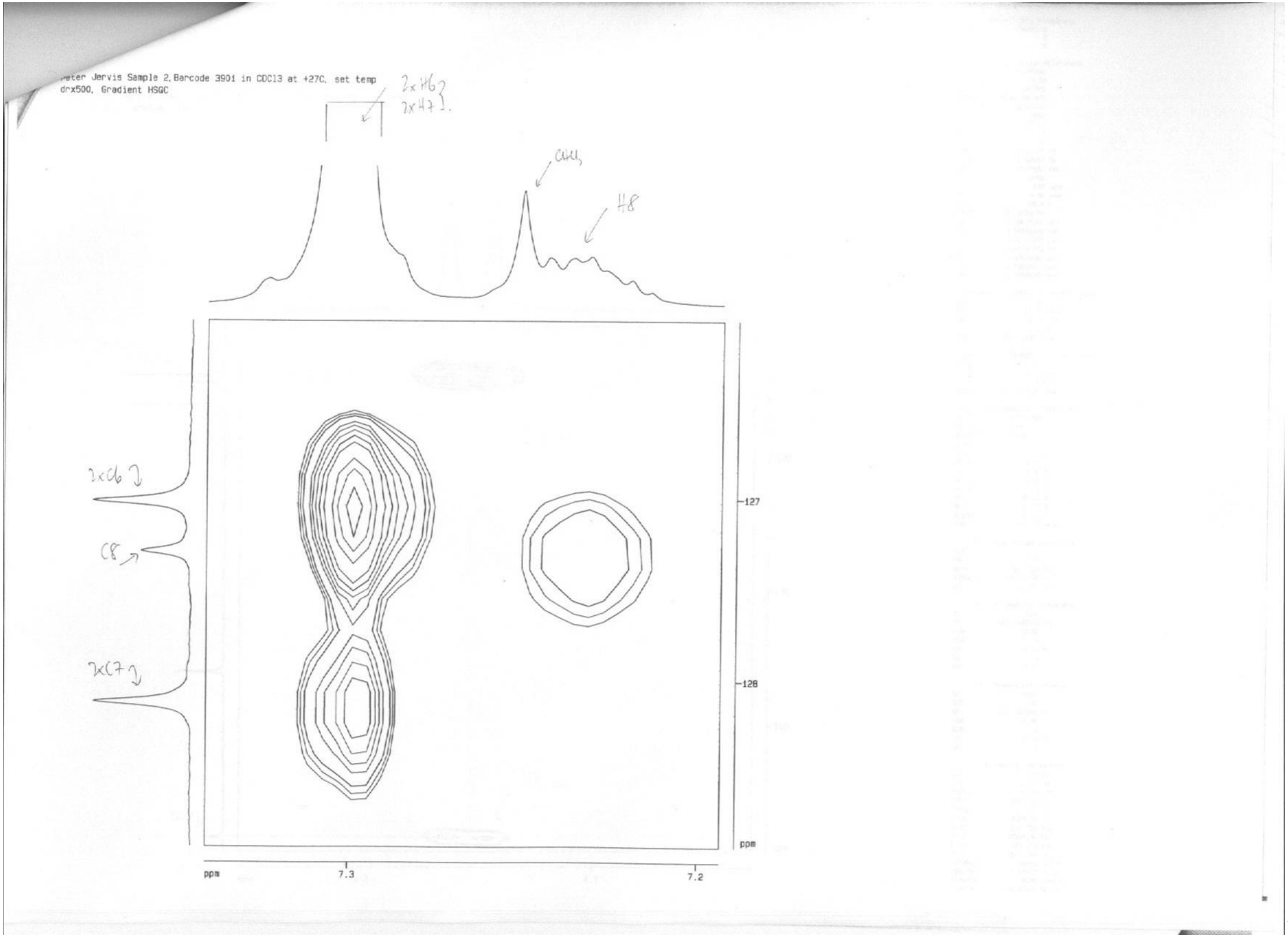
Peter Jervis Sample 2, Barcode 3901 in CDCl3 at +27C, set temp
drx500, Gradient HSQC
2xH6
2xH7
CH3
H8
2xC6
C8
2xC7
127
128
ppm
ppm
7.3
7.2

Peter Jervis Sample 2, Barcode 3901 in CDCl3 at +27C, set temp
drx500, Gradient HMBC

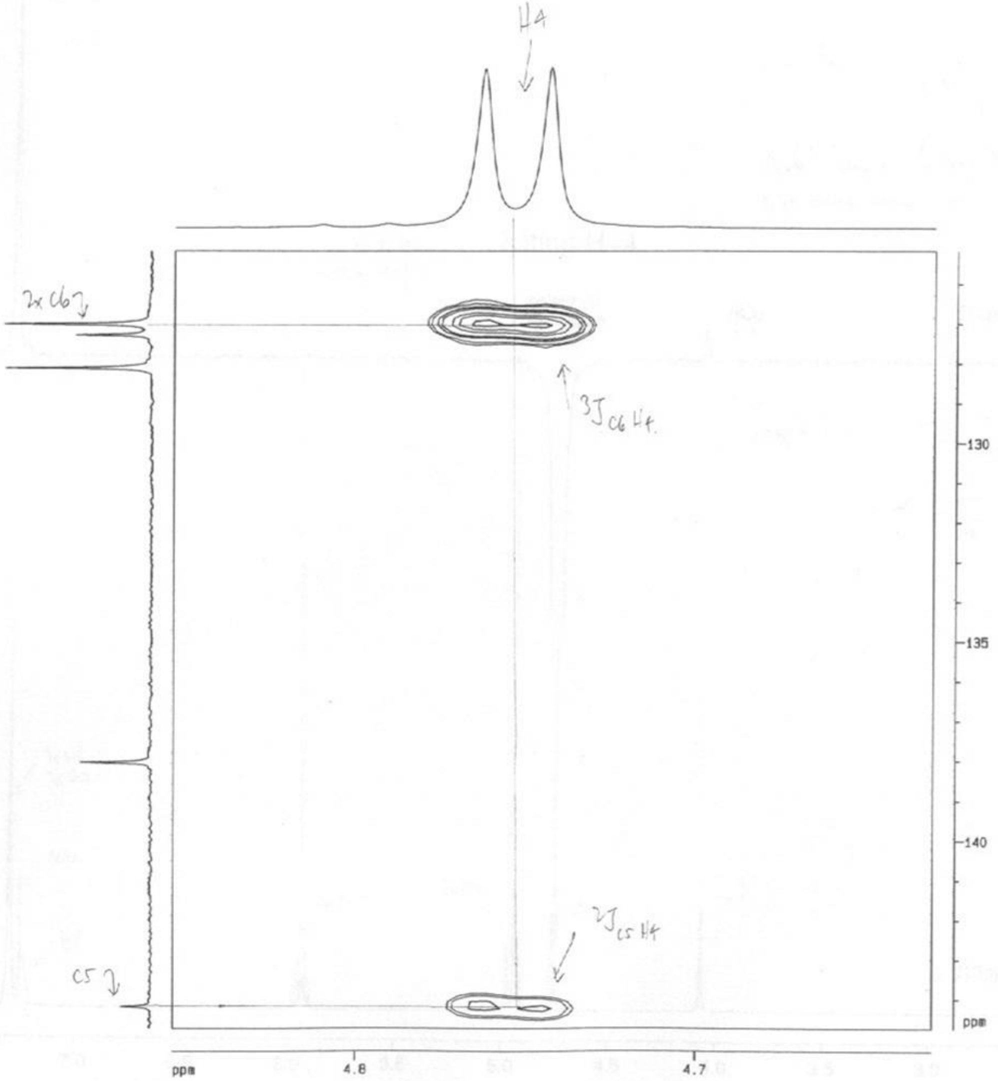

Current Data Parameters

NAME	j106pjid
EXPNO	3
PROCNO	1

F2 - Acquisition Parameters

Date_	20050706
Time	19.44
INSTRUM	drx500
PROBHD	5 mm TBI H/C
PULPROG	inv4gplplrnd
TD	2048
SOLVENT	CDCl3
NS	8
DS	16
SWH	4310.345 Hz
FIDRES	2.104661 Hz
AQ	0.2376180 sec
RG	32768
DW	116.000 usec
DE	5.50 usec
TE	300.0 K
CNST2	150.0000000
d0	0.00000300 sec
D1	2.00000000 sec
d2	0.00312500 sec
D6	0.10000000 sec
d13	0.00000300 sec
D16	0.00010000 sec
IN0	0.00002280 sec

============ CHANNEL f1 ============

NUC1	1H
P1	10.70 usec
p2	21.40 usec
PL1	1.00 dB
SFO1	500.1318867 MHz

============ CHANNEL f2 ============

NUC2	13C
P3	12.00 usec
PL2	-1.00 dB
SFO2	125.7667893 MHz

============ GRADIENT CHANNEL ============

GPNAM1	SINE.100
GPNAM2	SINE.100
GPNAM3	SINE.100
GPX1	0.00 %
GPX2	0.00 %
GPX3	0.00 %
GPY1	0.00 %
GPY2	0.00 %
GPY3	0.00 %
GPZ1	50.00 %
GPZ2	30.00 %
GPZ3	40.10 %
P16	1000.00 usec

F1 - Acquisition parameters

ND0	2
TD	512
SFO1	125.7668 MHz
FIDRES	42.831688 Hz
SW	174.369 ppm

F2 - Processing parameters

SI	2048
SF	500.1300233 MHz
WDW	QSINE
SSB	2
LB	0.00 Hz
GB	0
PC	0.20

F1 - Processing parameters

SI	1024
MC2	QF
SF	125.7577945 MHz
WDW	QSINE
SSB	2
LB	0.00 Hz
GB	0

2D NMR plot parameters

CX2	17.00 cm
CX1	17.00 cm
F2PLO	4.854 ppm
F2LO	2427.51 Hz
F2PHI	4.631 ppm
F2HI	2315.96 Hz
F1PLO	144.752 ppm
F1LO	18203.65 Hz
F1PHI	125.168 ppm
F1HI	15740.63 Hz
F2PPMCM	0.01312 ppm/cm
F2HZCM	6.56156 Hz/cm
F1PPMCM	1.15199 ppm/cm
F1HZCM	144.87190 Hz/cm

15b

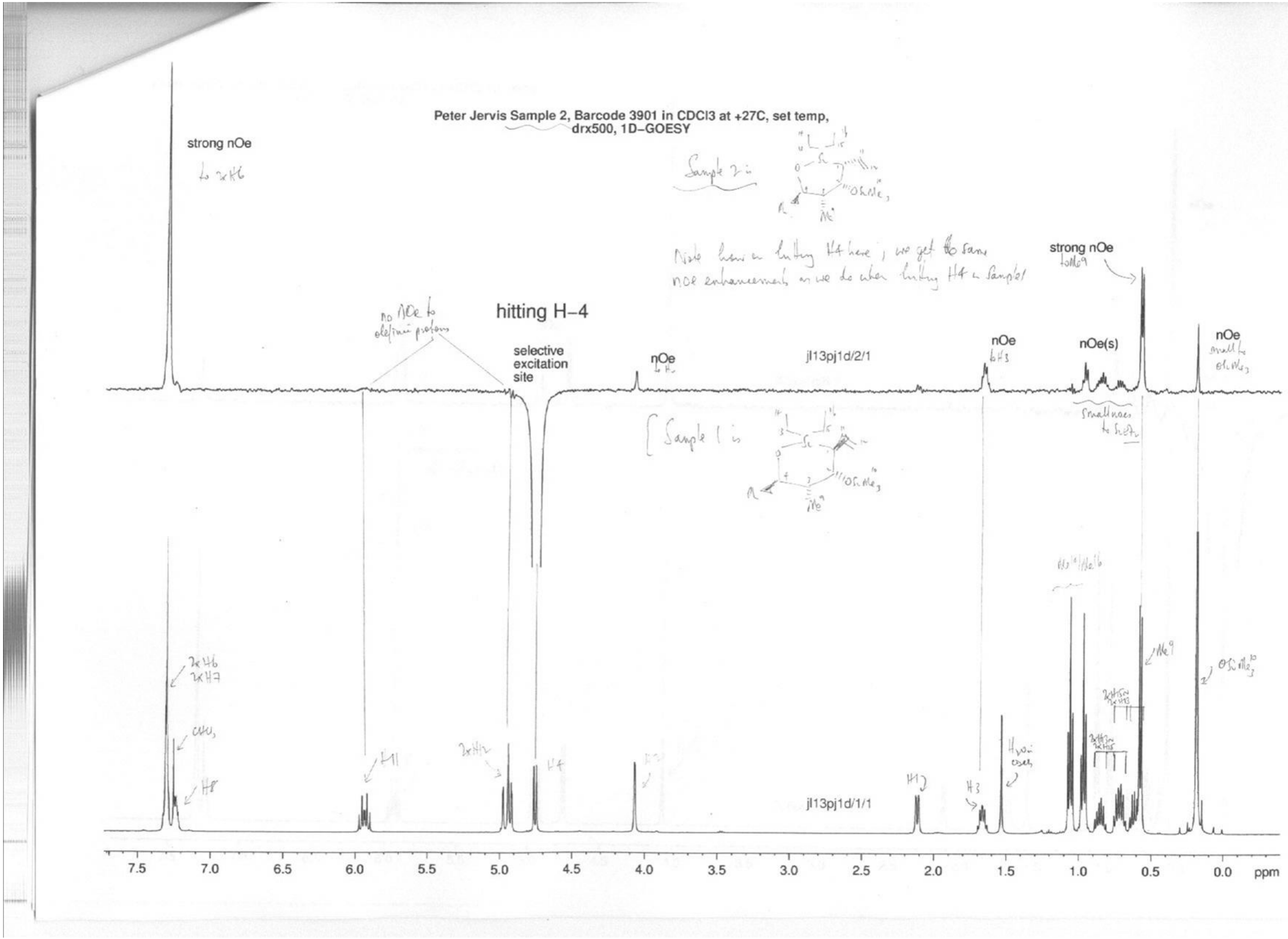

Peter Jervis Sample 2, Barcode 3901 in CDCl3 at +27C, set temp, drx500, 1D-GOESY
strong nOe
hitting H-4
selective excitation site
nOe
jl13pj1d/2/1
nOe
nOe(s)
strong nOe
nOe
jl13pj1d/1/1
7.5
7.0
6.5
6.0
5.5
5.0
4.5
4.0
3.5
3.0
2.5
2.0
1.5
1.0
0.5
0.0
ppm

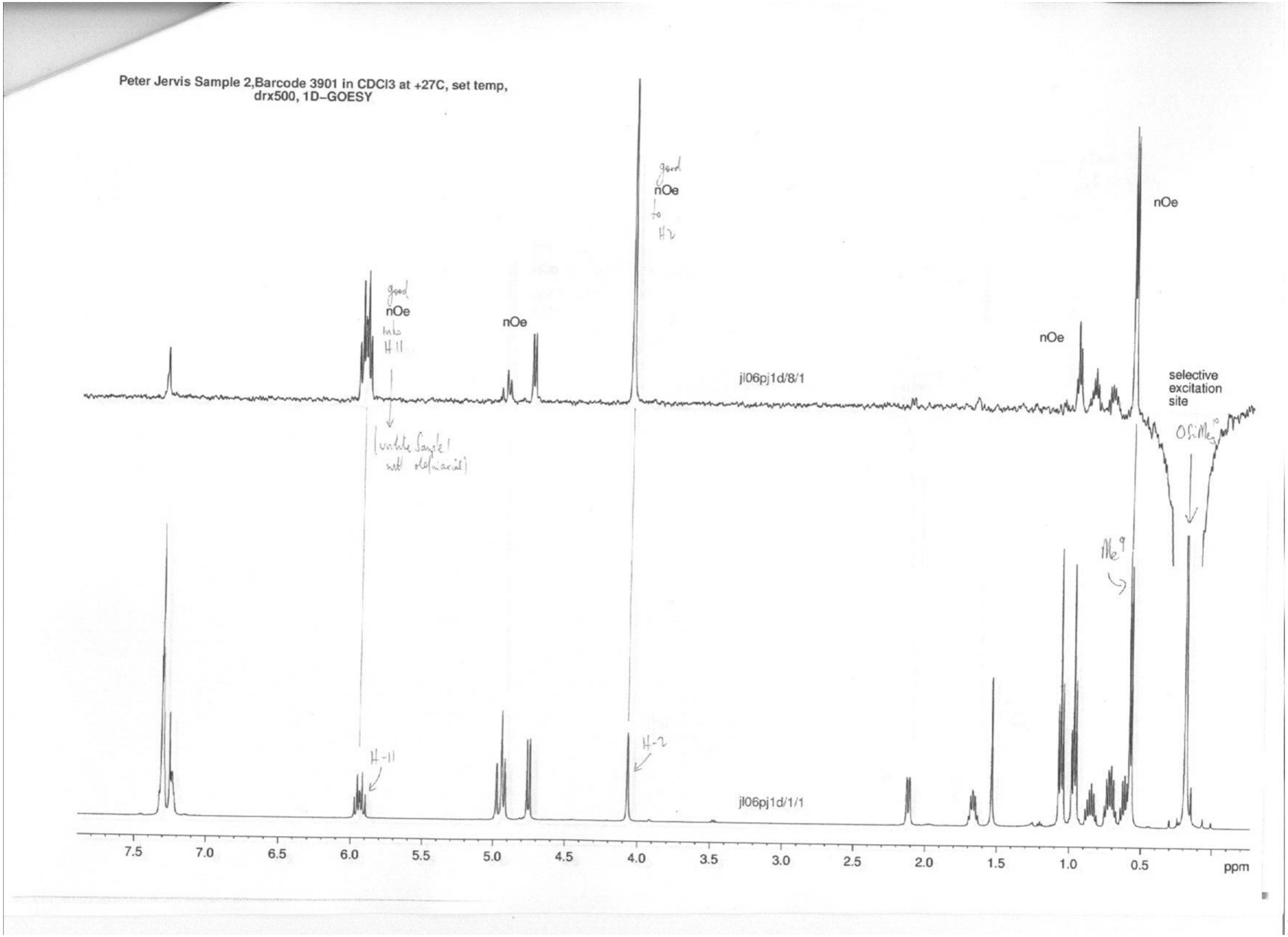
Peter Jervis Sample 2,Barcode 3901 in CDCl3 at +27C, set temp,
drx500, 1D-GOESY
good nOe to H2
good nOe into H11
nOe
nOe
nOe
jl06pj1d/8/1
selective excitation site
H-11
H-2
jl06pj1d/1/1
7.5
7.0
6.5
6.0
5.5
5.0
4.5
4.0
3.5
3.0
2.5
2.0
1.5
1.0
0.5
ppm

15b

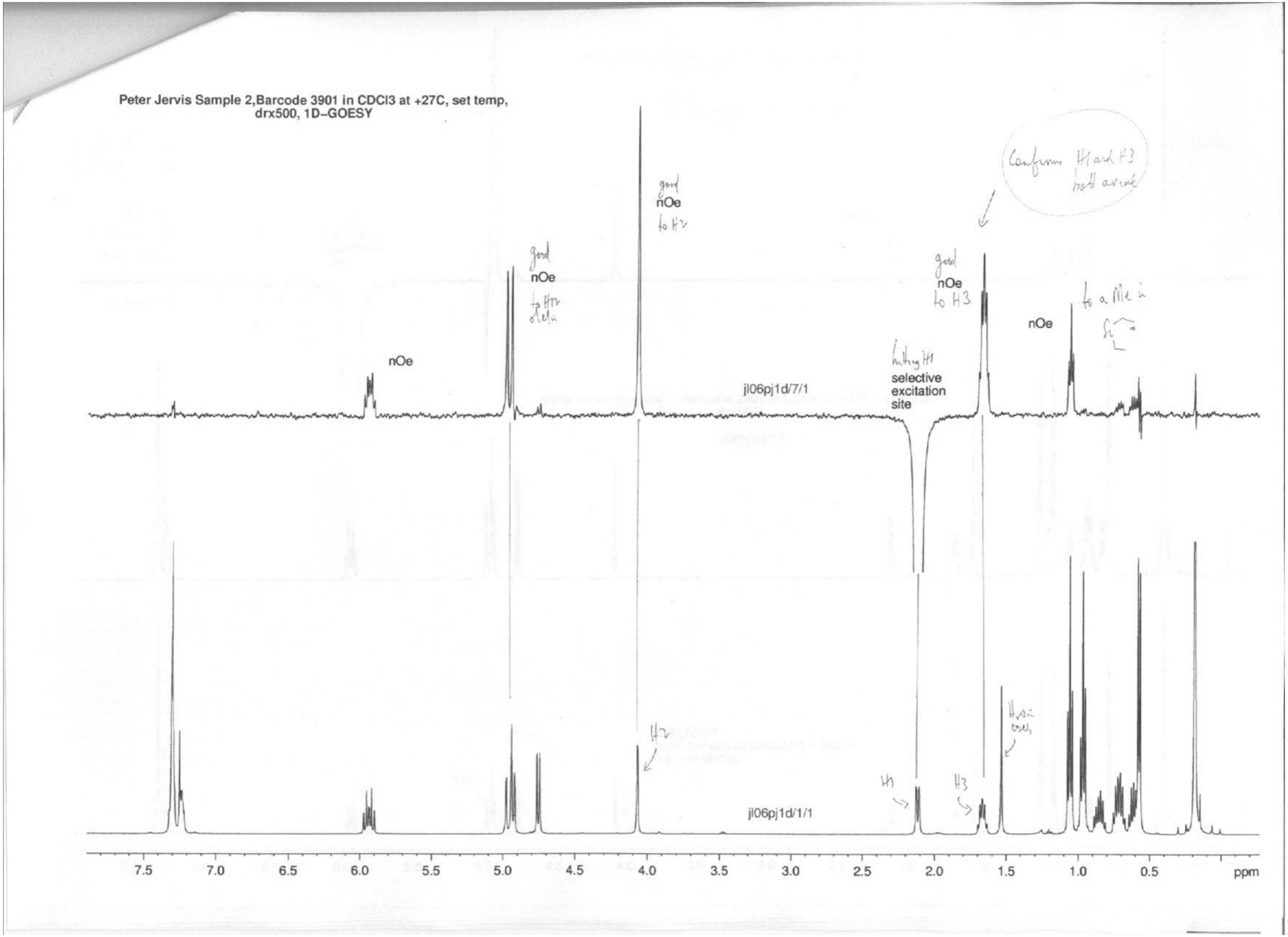
Peter Jervis Sample 2,Barcode 3901 in CDCl3 at +27C, set temp, drx500, 1D-GOESY
good nOe to H2
good nOe to H4 olefin
nOe
Confirm H1 and H3 both axial
good nOe to H3
nOe
to a Me in Si
hitting H1 selective excitation site
jl06pj1d/7/1
H2
H1
H3
jl06pj1d/1/1
7.5
7.0
6.5
6.0
5.5
5.0
4.5
4.0
3.5
3.0
2.5
2.0
1.5
1.0
0.5
ppm

15b

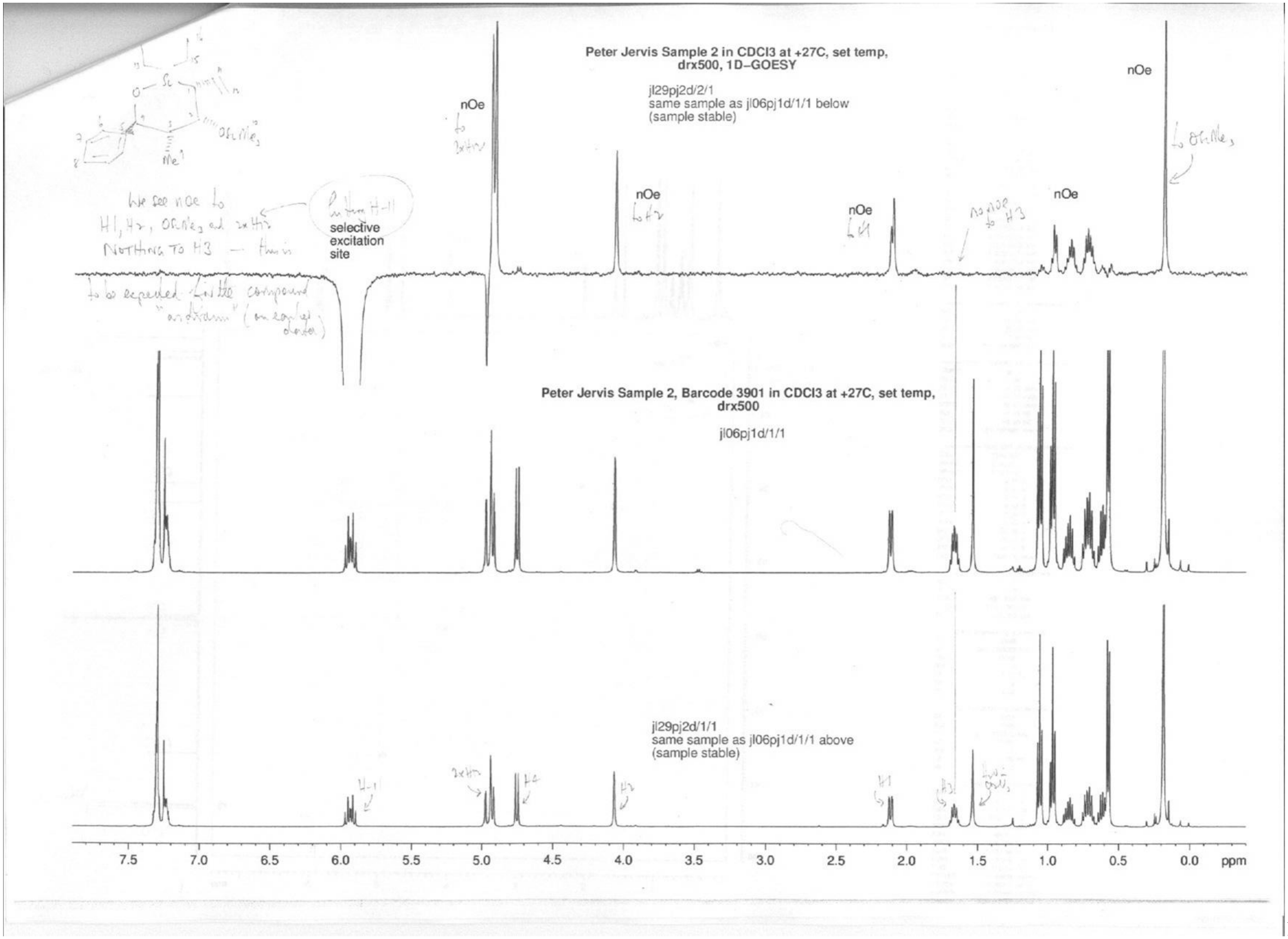
Peter Jervis Sample 2 in CDCl3 at +27C, set temp,
drx500, 1D-GOESY
jl29pj2d/2/1
same sample as jl06pj1d/1/1 below
(sample stable)
nOe
nOe
nOe
nOe
nOe
nOe
selective
excitation
site
Peter Jervis Sample 2, Barcode 3901 in CDCl3 at +27C, set temp,
drx500
jl06pj1d/1/1
jl29pj2d/1/1
same sample as jl06pj1d/1/1 above
(sample stable)
7.5
7.0
6.5
6.0
5.5
5.0
4.5
4.0
3.5
3.0
2.5
2.0
1.5
1.0
0.5
0.0
ppm

15b
